# Supplementary material for: Isoginkgetin antagonizes ALS pathologies in its animal and patient iPSC models via PINK1-Parkin-dependent mitophagy
Source: EMBO Mol Med. 2025 Oct 15;17(11):3139–73. doi: 10.1038/s44321-025-00323-2 (PMC12603167; doi:10.1038/s44321-025-00323-2)
Supplement: Supplementary file 1 — Appendix [file 44321_2025_323_MOESM1_ESM.pdf]

Appendix

Appendix Table S1: page 2 - 7

Appendix Table S1: Exact *P* values

Figure	Statistical method	Groups	Exact *P* value
Figure 1I	One-way ANOVA	ISO in *si-nc* vs ISO in *si-PINK1*	*P*<0.0001
Figure 1I	One-way ANOVA	CCCP in *si-nc* vs CCCP in *si-PINK1*	*P*<0.0001
Figure 2B	One-way ANOVA	Veh vs ISO (10 μM)	*P*=0.0463
Figure 2B	One-way ANOVA	CCCP (5 μM) vs ISO (5 μM)+CCCP	*P*=0.0002
Figure 2B	One-way ANOVA	CCCP (5 μM) vs ISO (10 μM)+CCCP	*P*<0.0001
Figure 3C	One-way ANOVA	CCCP (5 μM) vs ISO +CCCP(5+5 μM)	*P*=0.0407
Figure 3C	One-way ANOVA	CCCP (5 μM) vs ISO +CCCP(5+10 μM)	*P*=0.0006
Figure 3E	One-way ANOVA	Veh vs CCCP	*P*<0.0001
Figure 3E	One-way ANOVA	CCCP vs ISO+CCCP	*P*<0.0001
Figure 3G	One-way ANOVA	Veh vs CCCP	*P*=0.0104
Figure 3G	One-way ANOVA	CCCP vs ISO+CCCP	*P*=0.0271
Figure 3H	One-way ANOVA	Veh vs CCCP	*P*=0.0012
Figure 3H	One-way ANOVA	CCCP vs ISO+CCCP	*P*=0.0004
Figure 3K	Unpaired t test	Veh vs ISO	*P*=0.8144
Figure 3L	Unpaired t test	Veh vs ISO	*P*=0.7832
Figure 3M	Unpaired t test	Veh vs ISO	*P*=0.4822
Figure 4D	Unpaired t test	Ctrl vs ALS	*P*=0.0155
Figure 4F	Unpaired t test	Ctrl vs ALS	*P*=0.0253
Figure 4I	Unpaired t test	Ctrl vs ALS	*P*=0.0004
Figure 4K	Unpaired t test	Ctrl vs ALS	*P*<0.0001
Figure 4M	Unpaired t test	Ctrl vs ALS	*P*<0.0001
Figure 4N	Unpaired t test	Ctrl vs ALS	*P*<0.0001
Figure 4O	Unpaired t test	Ctrl vs ALS	*P*<0.0001
Figure 4Q	Unpaired t test	Ctrl vs ALS	*P*<0.0001
Figure 5B	Two-way ANOVA	C9-Veh vs C9-ISO	*P*<0.0001
Figure 5B	Two-way ANOVA	SOD1-Veh vs SOD1-ISO	*P*<0.0001
Figure 5B	Two-way ANOVA	TDP-43-Veh vs TDP-43-ISO	*P*<0.0001
Figure 5D	Two-way ANOVA	C9-Veh vs C9-ISO	*P*<0.0001
Figure 5D	Two-way ANOVA	SOD1-Veh vs SOD1-ISO	*P*<0.0001

Figure 5D	Two-way ANOVA	TDP-43-Veh vs TDP-43-ISO	*P*<0.0001
Figure 5F	Two-way ANOVA	C9-Veh vs C9-ISO	*P*=0.0003
Figure 5F	Two-way ANOVA	SOD1-Veh vs SOD1-ISO	*P*<0.0001
Figure 5F	Two-way ANOVA	TDP-43-Veh vs TDP-43-ISO	*P*<0.0001
Figure 5H	Two-way ANOVA	C9-Veh vs C9-ISO	*P*=0.0002
Figure 5H	Two-way ANOVA	SOD1-Veh vs SOD1-ISO	*P*=0.0002
Figure 5H	Two-way ANOVA	TDP-43-Veh vs TDP-43-ISO	*P*<0.0001
Figure 5J	Two-way ANOVA	C9-Veh vs C9-ISO	*P*<0.0001
Figure 5J	Two-way ANOVA	SOD1-Veh vs SOD1-ISO	*P*<0.0001
Figure 5J	Two-way ANOVA	TDP-43-Veh vs TDP-43-ISO	*P*<0.0001
Figure 5K	Two-way ANOVA	C9-Veh vs C9-ISO	*P*=0.0156
Figure 5K	Two-way ANOVA	SOD1-Veh vs SOD1-ISO	*P*=0.0014
Figure 5K	Two-way ANOVA	TDP-43-Veh vs TDP-43-ISO	*P*=0.0082
Figure 5M	Two-way ANOVA	C9-Veh vs C9-ISO	*P*=0.0473
Figure 5M	Two-way ANOVA	SOD1-Veh vs SOD1-ISO	*P*=0.0057
Figure 5M	Two-way ANOVA	TDP-43-Veh vs TDP-43-ISO	*P*=0.0241
Figure 6C	One-way ANOVA	Veh-WT vs Veh-G93A	*P*=0.0015
Figure 6C	One-way ANOVA	Veh-G93A vs ISO-G93A	*P*=0.0006
Figure 6D	Two-way ANOVA	Veh-WT vs ISO-WT	*P*=0.4499
Figure 6D	Two-way ANOVA	Veh-G93A vs ISO-G93A	*P*=0.0029
Figure 6E	Two-way ANOVA	Veh-WT vs ISO-WT	*P*=0.8441
Figure 6E	Two-way ANOVA	Veh-G93A vs ISO-G93A	*P*<0.0001
Figure 6G	Two-way ANOVA	DAY4: Veh-G93A GFP vs ISO-G93A GFP	*P*=0.0151
Figure 6G	Two-way ANOVA	DAY8: Veh-G93A GFP vs ISO-G93A GFP	*P*<0.0001

Figure 6H	Two-way ANOVA	DAY4: Veh-G93A GFP vs ISO-G93A GFP	*P*=0.0076
Figure 6H	Two-way ANOVA	DAY8: Veh-G93A GFP vs ISO-G93A GFP	*P*=0.0146
Figure 6I	Log-rank	Veh-WT vs ISO-WT	*P*=0.0659
Figure 6I	Log-rank	Veh-G93A vs ISO-G93A	*P*=0.0218
Figure 6K	One-way ANOVA	Veh-WT vs Veh-G93A	*P*=0.0159
Figure 6K	One-way ANOVA	Veh-G93A vs ISO-G93A	*P*<0.0001
Figure 6K	One-way ANOVA	ISO-G93A vs ISO+*pink1*RNAi-G93A	*P*=0.0003
Figure 6K	One-way ANOVA	ISO-G93A vs ISO+*pdr1*RNAi-G93A	*P*=0.0096
Figure 6L	One-way ANOVA	Veh-WT vs Veh-G93A	*P*<0.0001
Figure 6L	One-way ANOVA	Veh-G93A vs ISO-G93A	*P*=0.0010
Figure 6L	One-way ANOVA	ISO-G93A vs ISO+*pink1*RNAi-G93A	*P*=0.0010
Figure 6L	One-way ANOVA	ISO-G93A vs ISO+*pdr1*RNAi-G93A	*P*=0.0002
Figure 7D	Log-rank	Veh vs Nano-ISO	*P*=0.0381
Figure 7D	Log-rank	Veh vs Riluzole	*P*=0.5522
Figure 7E	One-way ANOVA	4-week: Veh vs Nano-ISO	*P*=0.0191
Figure 7E	One-way ANOVA	5-week: Veh vs Nano-ISO	*P*=0.0154
Figure 7F	Log-rank	Veh vs Nano-ISO	*P*=0.0343
Figure 7F	Log-rank	Veh vs Riluzole	*P*=0.7926
Figure 7H	One-way ANOVA	WT vs Veh	*P*=0.0002
Figure 7H	One-way ANOVA	Veh vs Nano-ISO	*P*=0.0374
Figure 7J	One-way ANOVA	WT vs Veh	*P*<0.0001
Figure 7J	One-way ANOVA	Veh vs Nano-ISO	*P*=0.0009
Figure 7K	One-way ANOVA	WT vs Veh	*P*<0.0001
Figure 7K	One-way ANOVA	Veh vs Nano-ISO	*P*=0.0027

Figure 7M	One-way ANOVA	WT vs Veh	*P*<0.0001
Figure 7M	One-way ANOVA	Veh vs Nano-ISO	*P*=0.0102
Figure 7O	One-way ANOVA	WT vs Veh	*P*=0.0113
Figure 7O	One-way ANOVA	Veh vs Nano-ISO	*P*=0.0020
Figure EV1D	One-way ANOVA	Veh vs ISO	*P*<0.0001
Figure EV1D	One-way ANOVA	Veh vs CCCP	*P*<0.0001
Figure EV1F	One-way ANOVA	Veh vs ISO	*P*<0.0001
Figure EV1F	One-way ANOVA	Veh vs CCCP	*P*<0.0001
Figure EV1I	One-way ANOVA	Veh vs ISO	*P*<0.0001
Figure EV1I	One-way ANOVA	ISO vs ISO+*si-PINK1*	*P*<0.0001
Figure EV2G	Unpaired t test	Veh vs ISO	*P*=0.4778
Figure EV2H	Two-way ANOVA	Basal: DMSO vs ISO	*P*=0.9794
Figure EV2H	Two-way ANOVA	CCCP: DMSO vs ISO	*P*=0.9394
Figure EV2H	Two-way ANOVA	Post washout: DMSO vs ISO	*P*=0.9930
Figure EV4A	One-way ANOVA	Veh vs 250 nM	*P*=0.9963
Figure EV4A	One-way ANOVA	Veh vs 500 nM	*P*=0.3897
Figure EV4A	One-way ANOVA	Veh vs 1000 nM	*P*<0.0001
Figure EV4C	Two-way ANOVA	C9-Veh vs C9-ISO	*P*<0.0001
Figure EV4C	Two-way ANOVA	C9-ISO vs C9-ISO+PRT	*P*<0.0001
Figure EV4C	Two-way ANOVA	SOD1-Veh vs SOD1-ISO	*P*=0.0008
Figure EV4C	Two-way ANOVA	SOD1-ISO vs SOD1-ISO+PRT	*P*=0.0002
Figure EV4C	Two-way ANOVA	TDP-43-Veh vs TDP-43-ISO	*P*=0.0004
Figure EV4C	Two-way ANOVA	TDP-43-ISO vs TDP-43-ISO+PRT	*P*=0.0063
Figure EV4E	Two-way ANOVA	C9-Veh vs C9-ISO	*P*=0.0121
Figure EV4E	Two-way ANOVA	C9-ISO vs C9-ISO+PRT	*P*=0.0163
Figure EV4E	Two-way ANOVA	SOD1-Veh vs SOD1-ISO	*P*=0.0064
Figure EV4E	Two-way ANOVA	SOD1-ISO vs SOD1-ISO+PRT	*P*=0.0032
Figure EV4E	Two-way ANOVA	TDP-43-Veh vs TDP-43-ISO	*P*=0.0020
Figure EV4E	Two-way ANOVA	TDP-43-ISO vs TDP-43-ISO+PRT	*P*=0.0031

Figure EV4G	Two-way ANOVA	C9-Veh vs C9-ISO	*P*=0.0011
Figure EV4G	Two-way ANOVA	C9-ISO vs C9-ISO+PRT	*P*=0.0087
Figure EV4G	Two-way ANOVA	SOD1-Veh vs SOD1-ISO	*P*<0.0001
Figure EV4G	Two-way ANOVA	SOD1-ISO vs SOD1-ISO+PRT	*P*=0.0019
Figure EV4G	Two-way ANOVA	TDP-43-Veh vs TDP-43-ISO	*P*=0.0004
Figure EV4G	Two-way ANOVA	TDP-43-ISO vs TDP-43-ISO+PRT	*P*<0.0001
Figure EV5A	One-way ANOVA	Veh vs ISO 150 μM	*P*<0.0001
Figure EV5A	One-way ANOVA	Veh vs ISO 150 μM	*P*=0.0003
Figure EV5A	One-way ANOVA	Veh vs ISO 150 μM	*P*<0.0001
Figure EV5A	One-way ANOVA	Veh vs ISO 150 μM	*P*<0.0001
Figure EV5C	One-way ANOVA	Veh vs ISO 15 μM	*P*<0.0001
Figure EV5D	One-way ANOVA	Veh vs ISO 5 μM	*P*=0.2228
Figure EV5D	One-way ANOVA	Veh vs ISO 15 μM	*P*<0.0001
Figure EV5E	Two-way ANOVA	No-kill: Veh-G93A vs ISO-G93A	*P*=0.0012
Figure EV5E	Two-way ANOVA	UV-kill: Veh-G93A vs ISO-G93A	*P*=0.0018
Figure EV5E	Two-way ANOVA	No-kill: ISO-G93A vs UV-kill: ISO G93A	*P*=0.5949
Figure EV5F	One-way ANOVA	DAY8: Veh-WT vs Veh-G93A	*P*=0.0022
Figure EV5F	One-way ANOVA	DAY8: Veh-G93A vs ISO-G93A	*P*=0.0266
Figure EV5F	One-way ANOVA	DAY10: Veh-WT vs Veh-G93A	*P*<0.0001
Figure EV5F	One-way ANOVA	DAY10: Veh-G93A vs ISO-G93A	*P*<0.0001
Figure EV5F	One-way ANOVA	DAY12: Veh-WT vs Veh-G93A	*P*<0.0001
Figure EV5F	One-way ANOVA	DAY12: Veh-G93A vs ISO-G93A	*P*<0.0001
Figure EV5F	One-way ANOVA	DAY14: Veh-WT vs Veh-G93A	*P*<0.0001
Figure EV5F	One-way ANOVA	DAY14: Veh-G93A vs ISO-G93A	*P*=0.0182
Figure EV7B	Unpaired t test	Wild type vs SOD1 G93A	*P*=0.6302
Figure EV7C	Unpaired t test	Wild type vs SOD1	*P*=0.0162

		G93A	
Figure EV7D	Unpaired t test	Wild type vs SOD1 G93A	*P*=0.0337
Figure EV7G	Unpaired t test	Wild type vs SOD1 G93A	*P*=0.0371
Figure EV7H	Unpaired t test	Wild type vs SOD1 G93A	*P*=0.0011
Figure EV7I	Unpaired t test	Wild type vs SOD1 G93A	*P*=0.2797
Figure EV7J	Unpaired t test	Wild type vs SOD1 G93A	*P*=0.7641
