# Supplementary material for: Isoginkgetin antagonizes ALS pathologies in its animal and patient iPSC models via PINK1-Parkin-dependent mitophagy
Source: EMBO Mol Med. 2025 Oct 15;17(11):3139–73. doi: 10.1038/s44321-025-00323-2 (PMC12603167; doi:10.1038/s44321-025-00323-2)
Supplement: Supplementary file 2 — Table EV1 [file 44321_2025_323_MOESM2_ESM.docx]

Table EV1 Detailed information of patients' spinal cord tissue samples.

| Diagnosis code^1^ | | Autopsy^1^ | Sex^1^ | Age^1^ | Braak^1,2^ | Amyloid^1^ | Region^1^ |
| --- | --- | --- | --- | --- | --- | --- | --- |
| Non-demented control | C6 | S18/058 | M | 87 | 1 | - | Spinal cord |
|  | C7 | S15/033 | M | 93 | 0 | A |  |
|  | C8 | S15/016 | F | 95 | 3 | B |  |
| ALS | A1 | S18/052 | M | 78 | 2 | - | Spinal cord |
|  | A2 | S16/088 | M | 57 | - | - |  |
|  | A3 | S15/086 | F | 67 | 1 | - |  |

^1^Provide from Netherlands Brain Bank

^2^Braak stage indicates
