# Supplementary figures and images for: Isoginkgetin antagonizes ALS pathologies in its animal and patient iPSC models via PINK1-Parkin-dependent mitophagy (part 1 of 3)
Source: EMBO Mol Med. 2025 Oct 15;17(11):3139–73. doi: 10.1038/s44321-025-00323-2 (PMC12603167; doi:10.1038/s44321-025-00323-2)

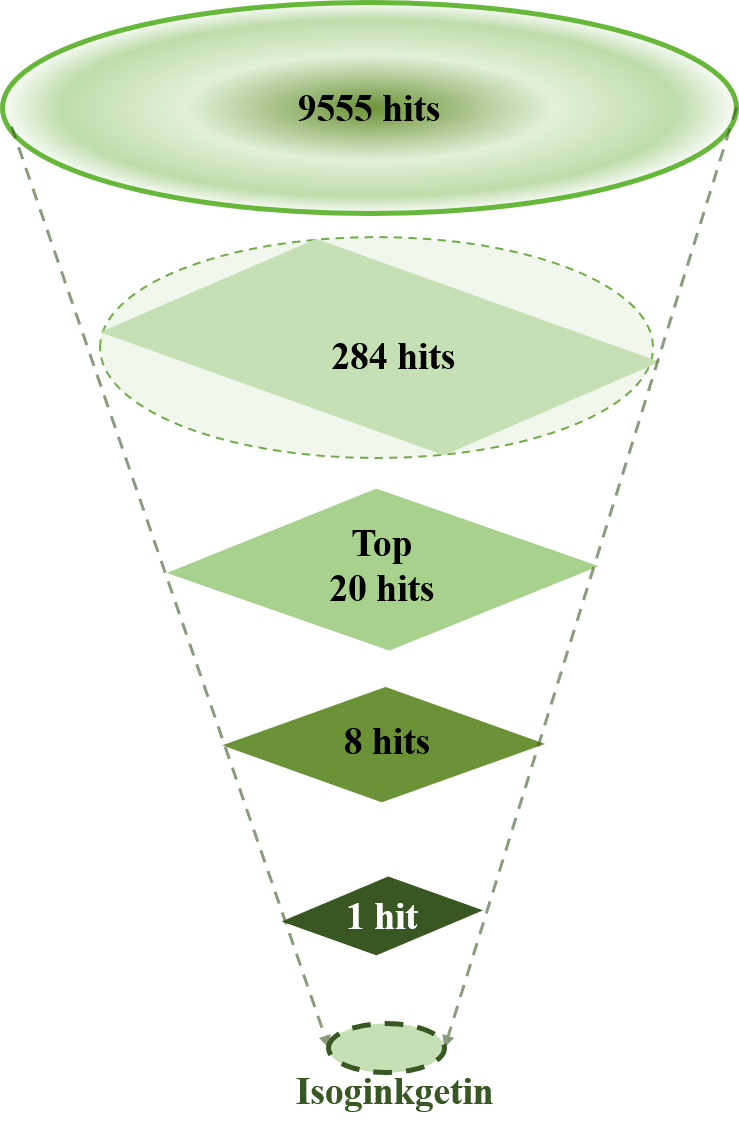

Supplement: Supplementary file 5 — Source data Fig. 1 [file 44321_2025_323_MOESM5_ESM.zip › Figure 1/1A/1A.tif]

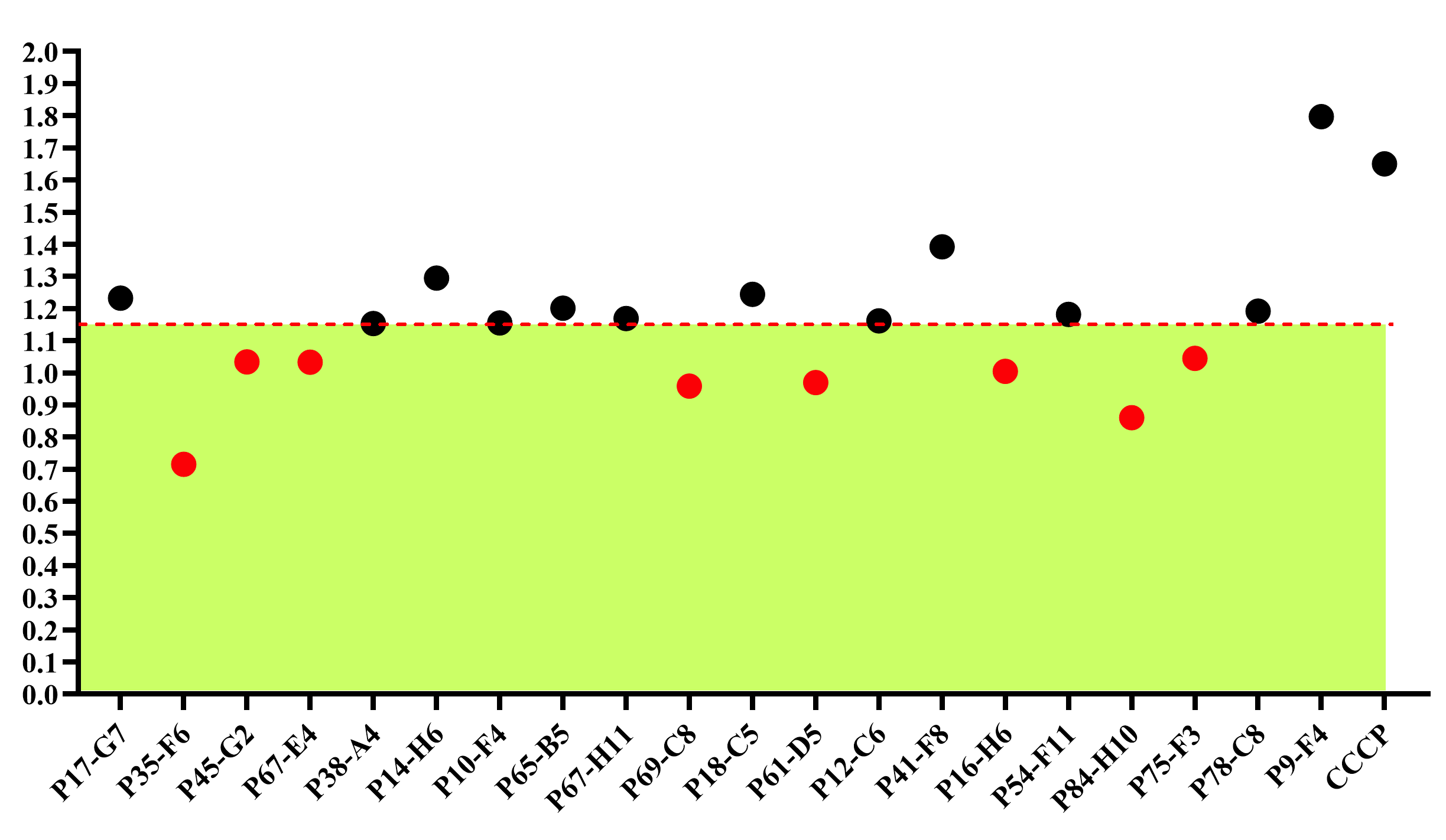

Supplement: Supplementary file 5 — Source data Fig. 1 [file 44321_2025_323_MOESM5_ESM.zip › Figure 1/1B/1B.tif]

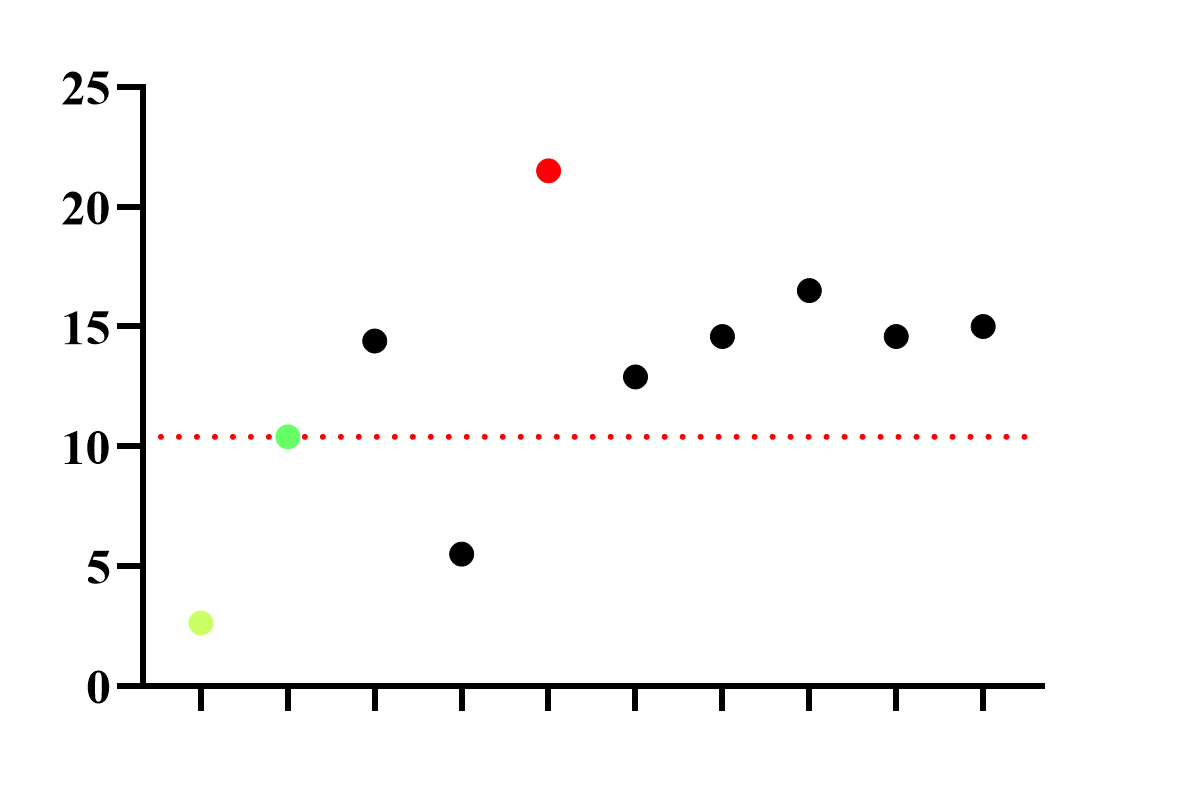

Supplement: Supplementary file 5 — Source data Fig. 1 [file 44321_2025_323_MOESM5_ESM.zip › Figure 1/1C/1C.tif]

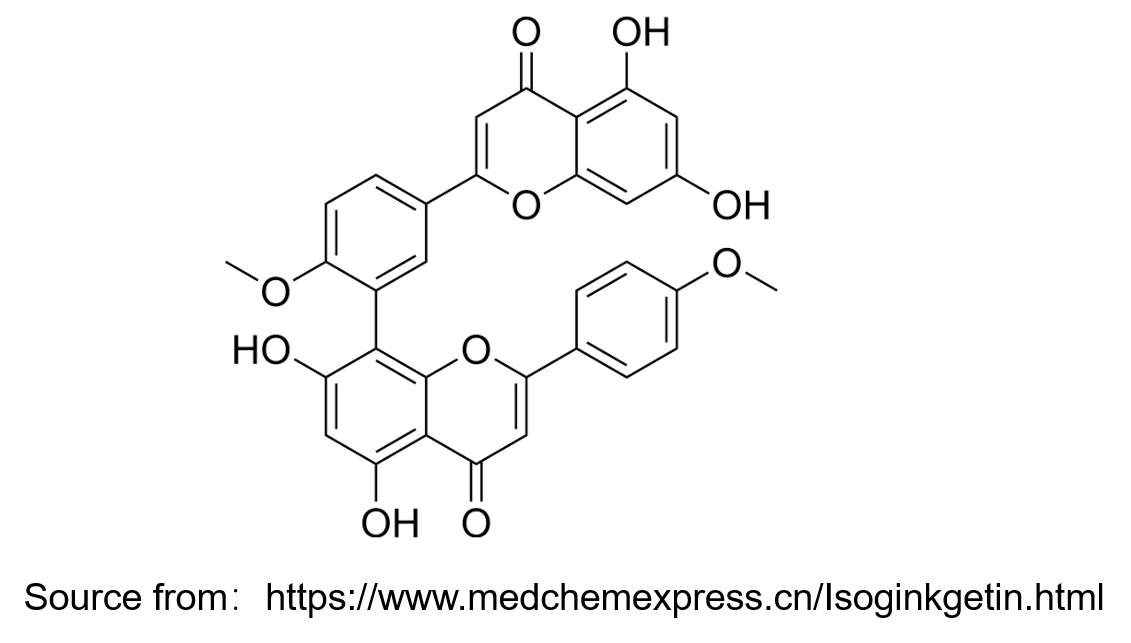

Supplement: Supplementary file 5 — Source data Fig. 1 [file 44321_2025_323_MOESM5_ESM.zip › Figure 1/1D/ISO.tif]

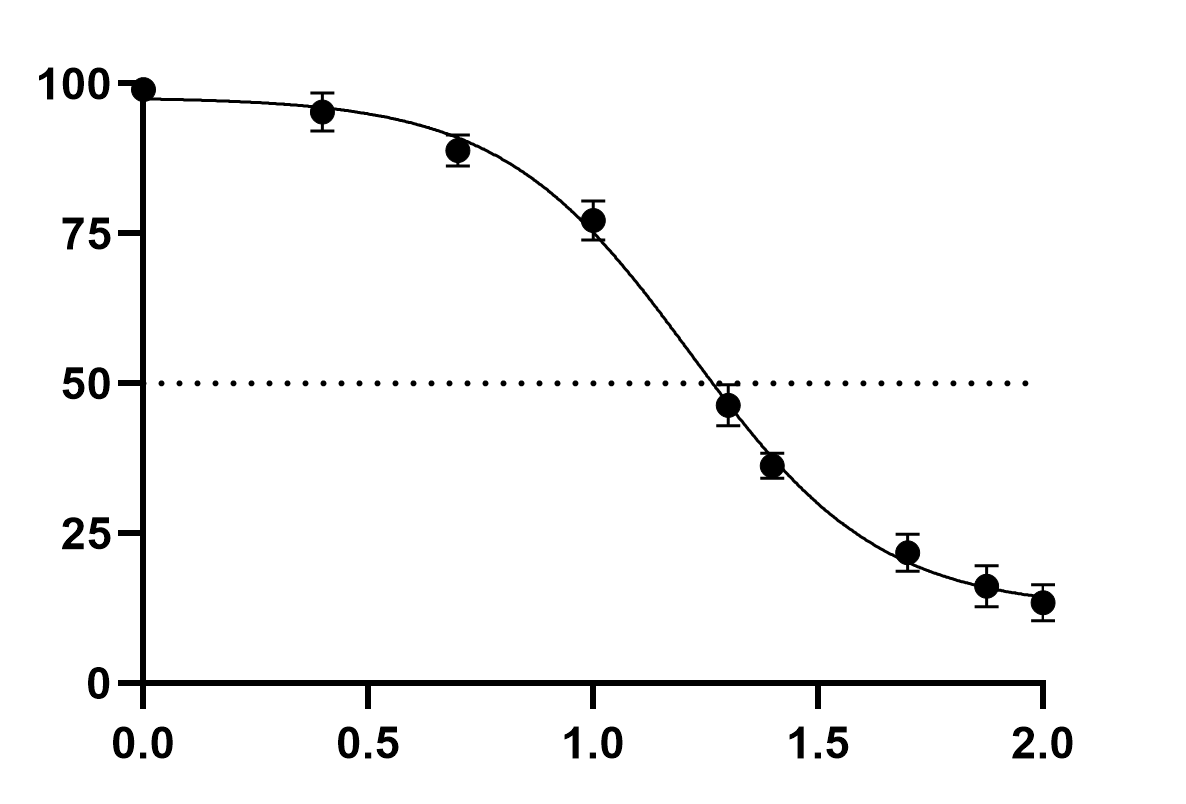

Supplement: Supplementary file 5 — Source data Fig. 1 [file 44321_2025_323_MOESM5_ESM.zip › Figure 1/1E/CC50.tif]

## Slide 1
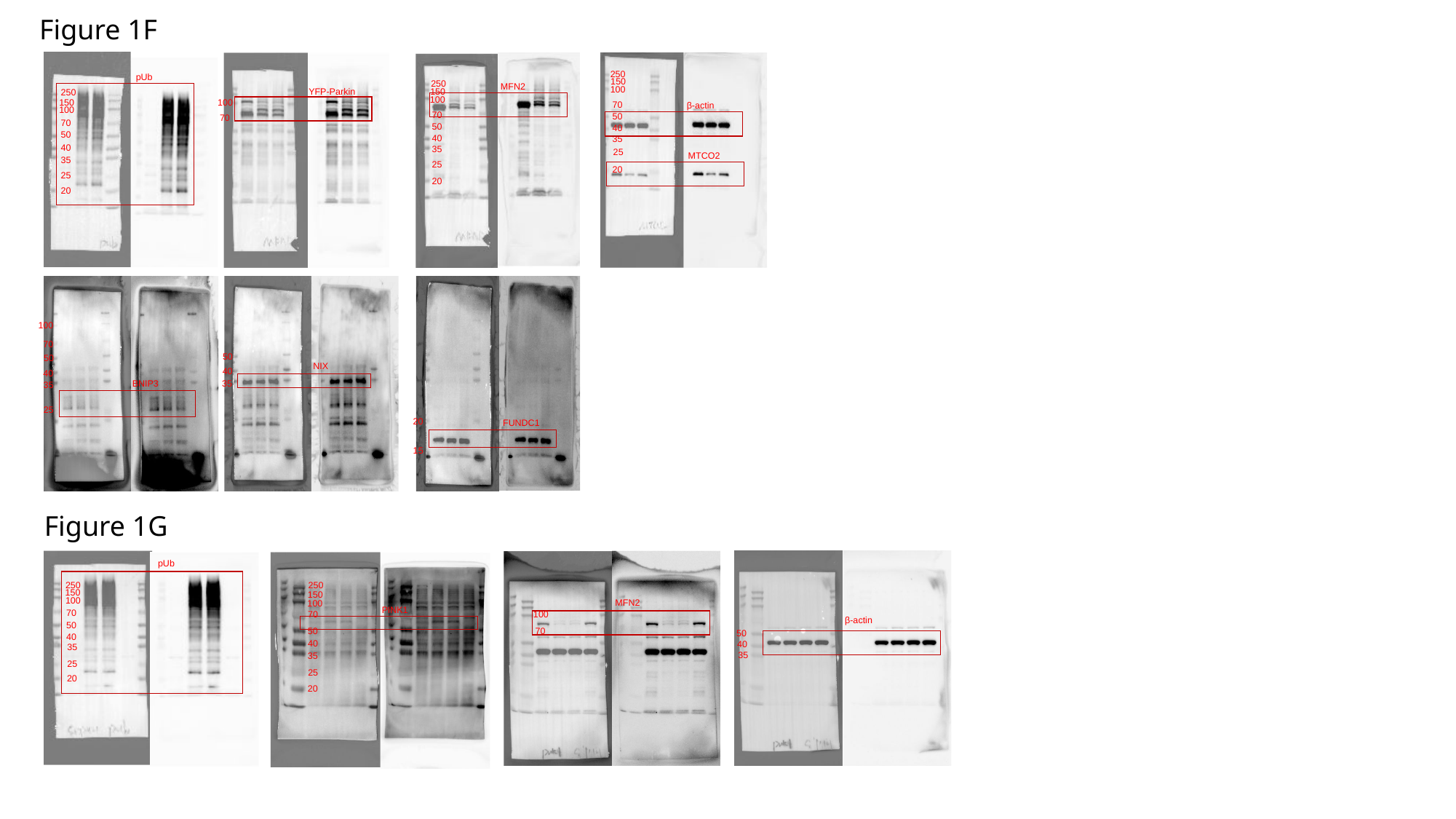

Figure 1F
pUb
250
150
100
70
50
40
35
25
20
250
150
100
70
β-actin
50
40
35
25
MTCO2
20
YFP-Parkin
100
70
250
MFN2
150
100
70
50
40
35
25
20
100
70
50
50
NIX
40
40
BNIP3
35
35
25
20
FUNDC1
15
Figure 1G
pUb
250
150
100
70
50
40
35
25
20
MFN2
100
70
β-actin
50
40
35
PINK1
25
20
250
150
100
70
50
40
35

Supplement: Supplementary file 5 — Source data Fig. 1 [file 44321_2025_323_MOESM5_ESM.zip › Figure 1/1F-G/WB.pptx]

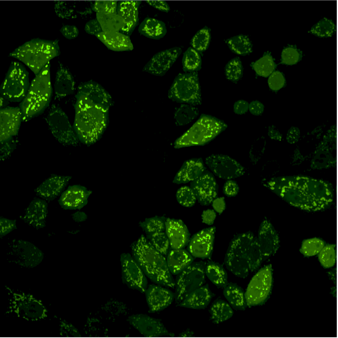

Supplement: Supplementary file 5 — Source data Fig. 1 [file 44321_2025_323_MOESM5_ESM.zip › Figure 1/1H/si-nc CCCP.tif]

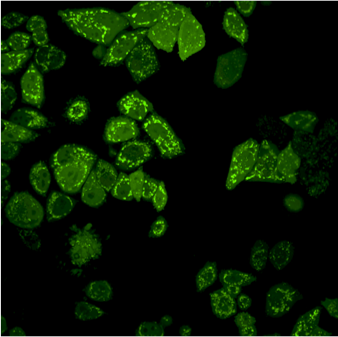

Supplement: Supplementary file 5 — Source data Fig. 1 [file 44321_2025_323_MOESM5_ESM.zip › Figure 1/1H/si-nc ISO.tif]

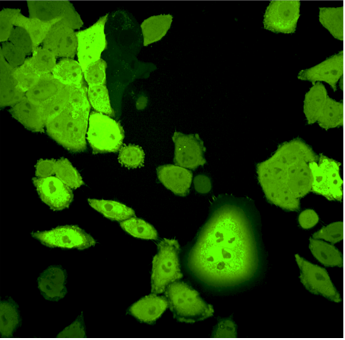

Supplement: Supplementary file 5 — Source data Fig. 1 [file 44321_2025_323_MOESM5_ESM.zip › Figure 1/1H/si-nc Veh.tif]

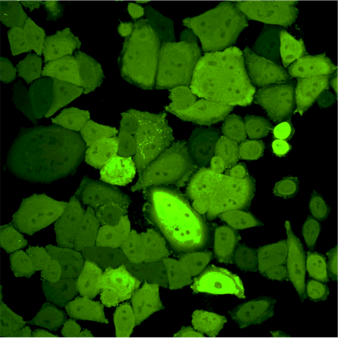

Supplement: Supplementary file 5 — Source data Fig. 1 [file 44321_2025_323_MOESM5_ESM.zip › Figure 1/1H/si-pink1 CCCP.tif]

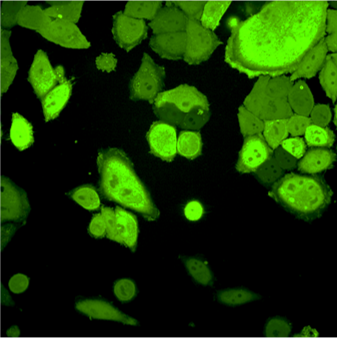

Supplement: Supplementary file 5 — Source data Fig. 1 [file 44321_2025_323_MOESM5_ESM.zip › Figure 1/1H/si-PINK1 ISO.tif]

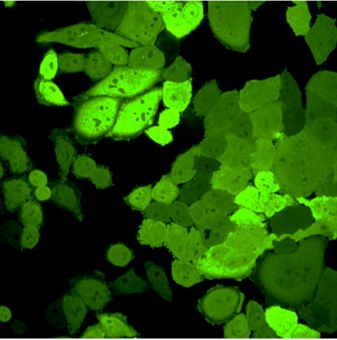

Supplement: Supplementary file 5 — Source data Fig. 1 [file 44321_2025_323_MOESM5_ESM.zip › Figure 1/1H/si-PINK1 Veh.tif]

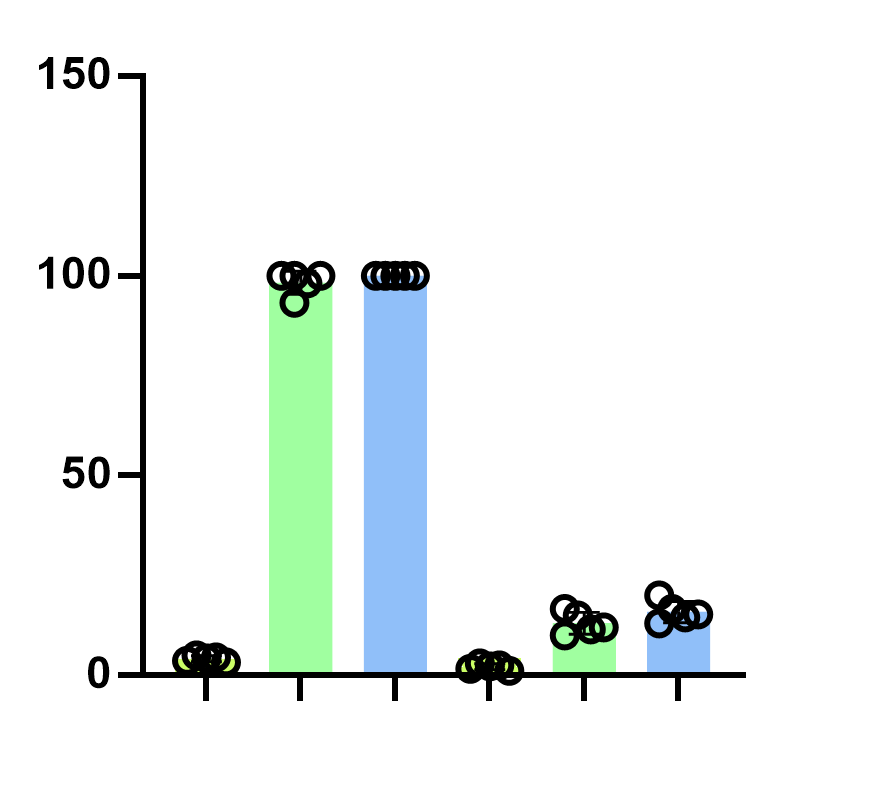

Supplement: Supplementary file 5 — Source data Fig. 1 [file 44321_2025_323_MOESM5_ESM.zip › Figure 1/1I/parkin.tif]

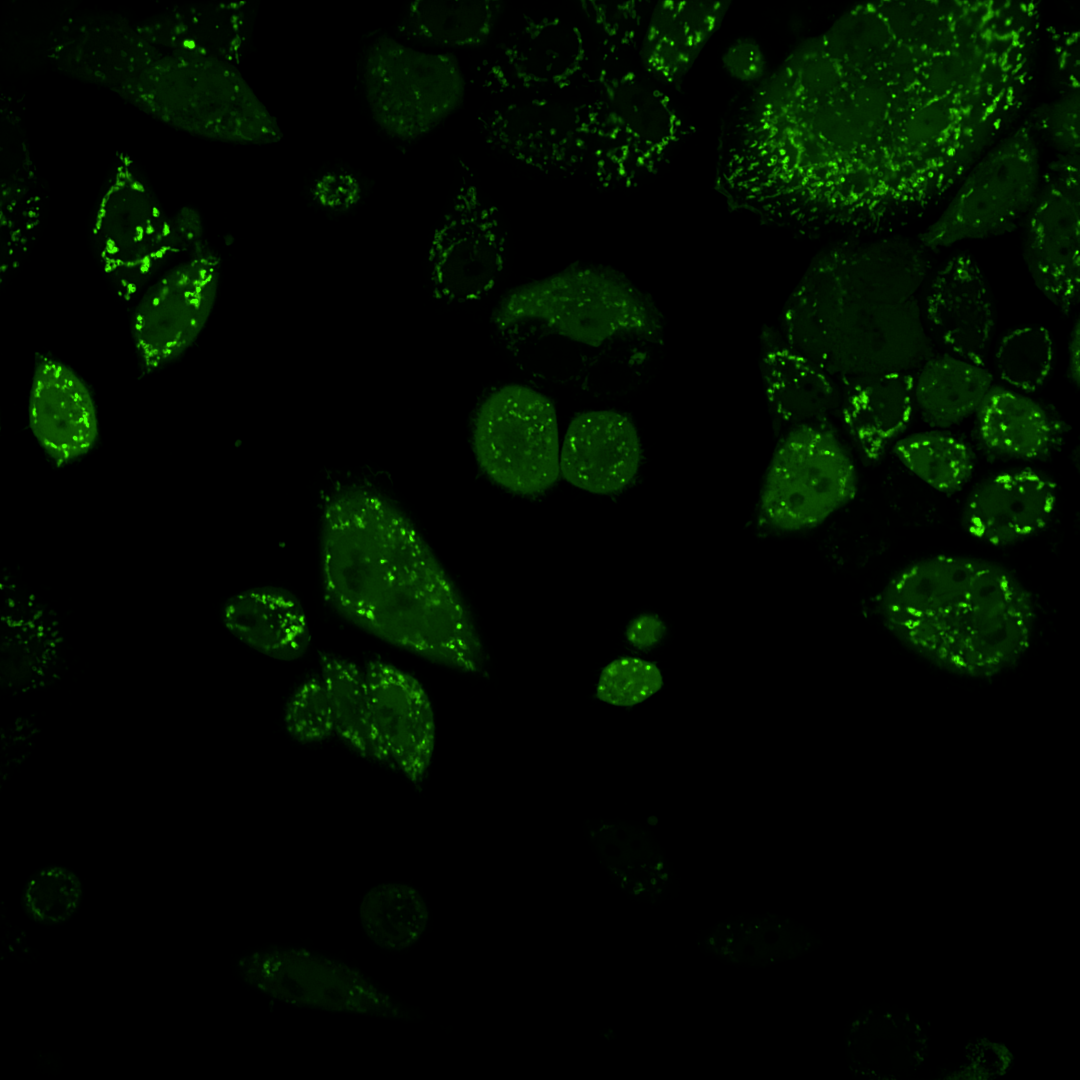

Supplement: Supplementary file 5 — Source data Fig. 1 [file 44321_2025_323_MOESM5_ESM.zip › Figure 1/1I/si-nc CCCP/1.png]

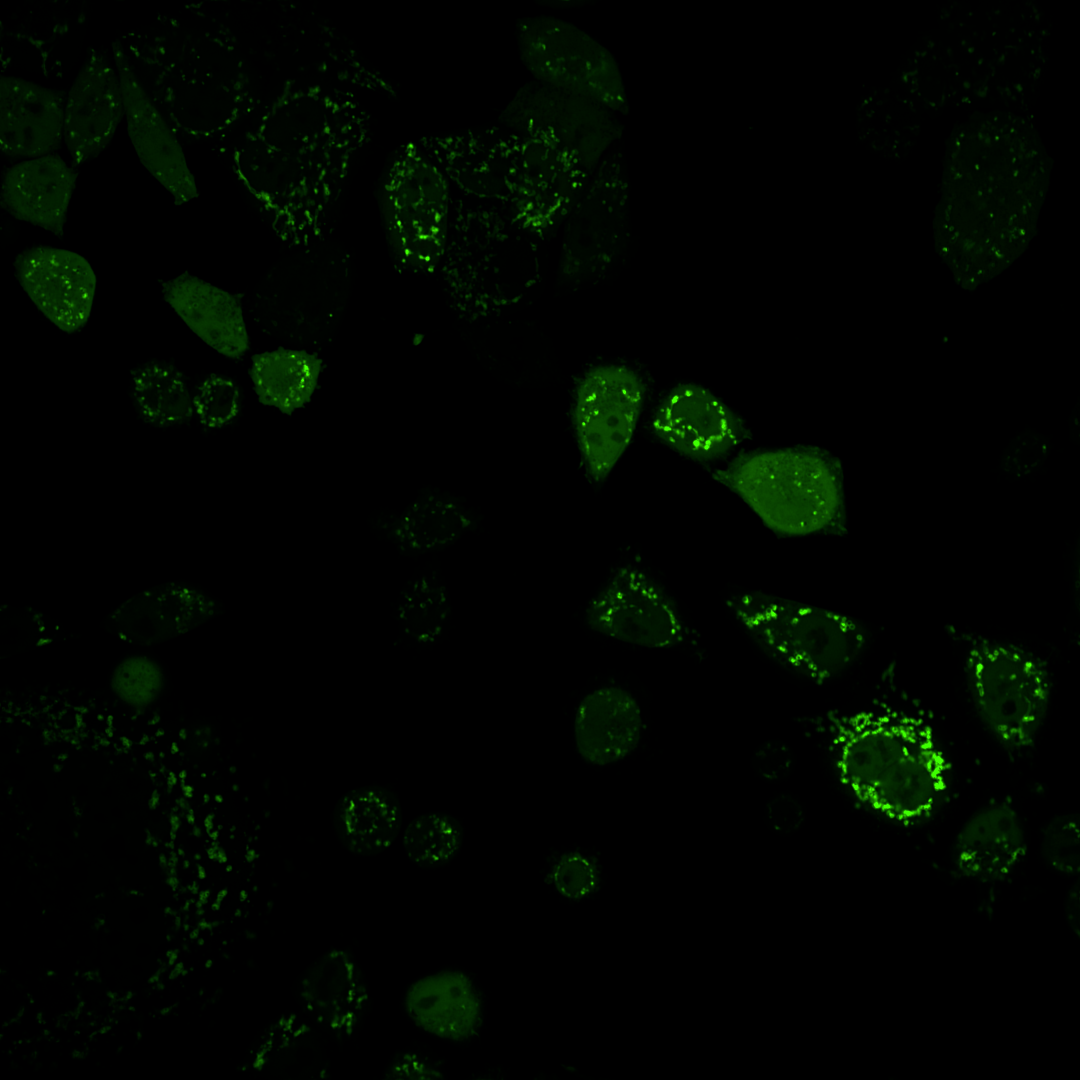

Supplement: Supplementary file 5 — Source data Fig. 1 [file 44321_2025_323_MOESM5_ESM.zip › Figure 1/1I/si-nc CCCP/2.png]

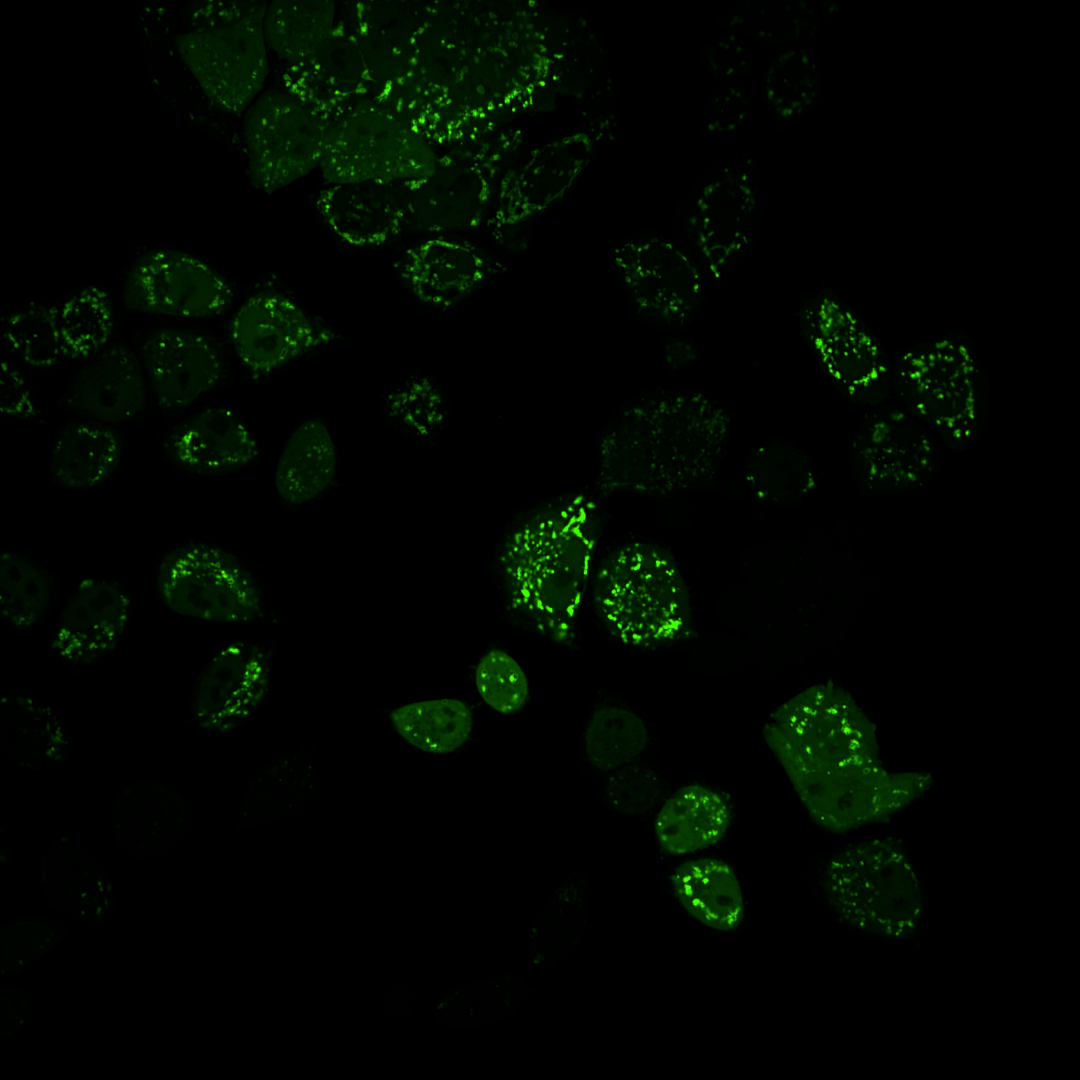

Supplement: Supplementary file 5 — Source data Fig. 1 [file 44321_2025_323_MOESM5_ESM.zip › Figure 1/1I/si-nc CCCP/3.png]

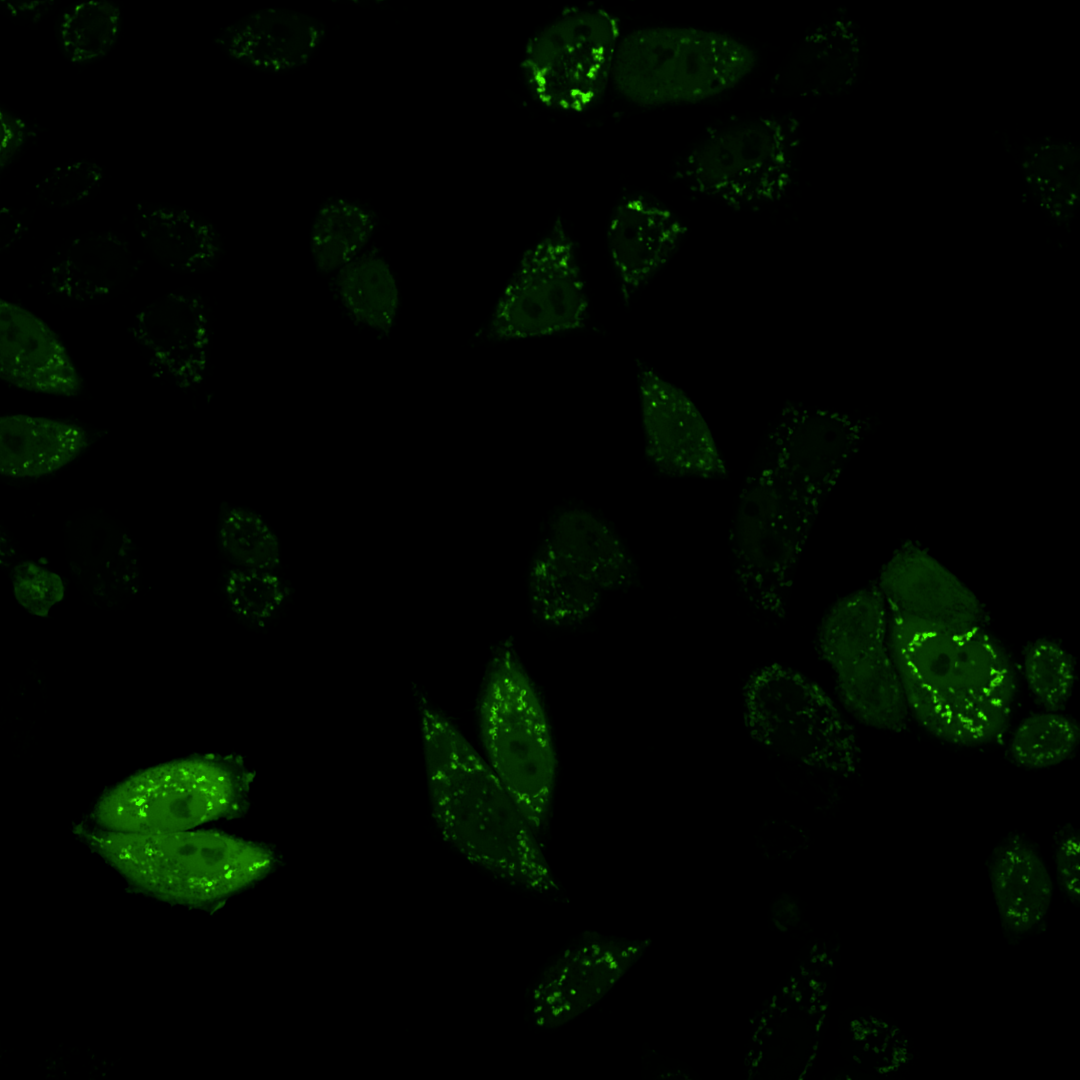

Supplement: Supplementary file 5 — Source data Fig. 1 [file 44321_2025_323_MOESM5_ESM.zip › Figure 1/1I/si-nc CCCP/4.png]

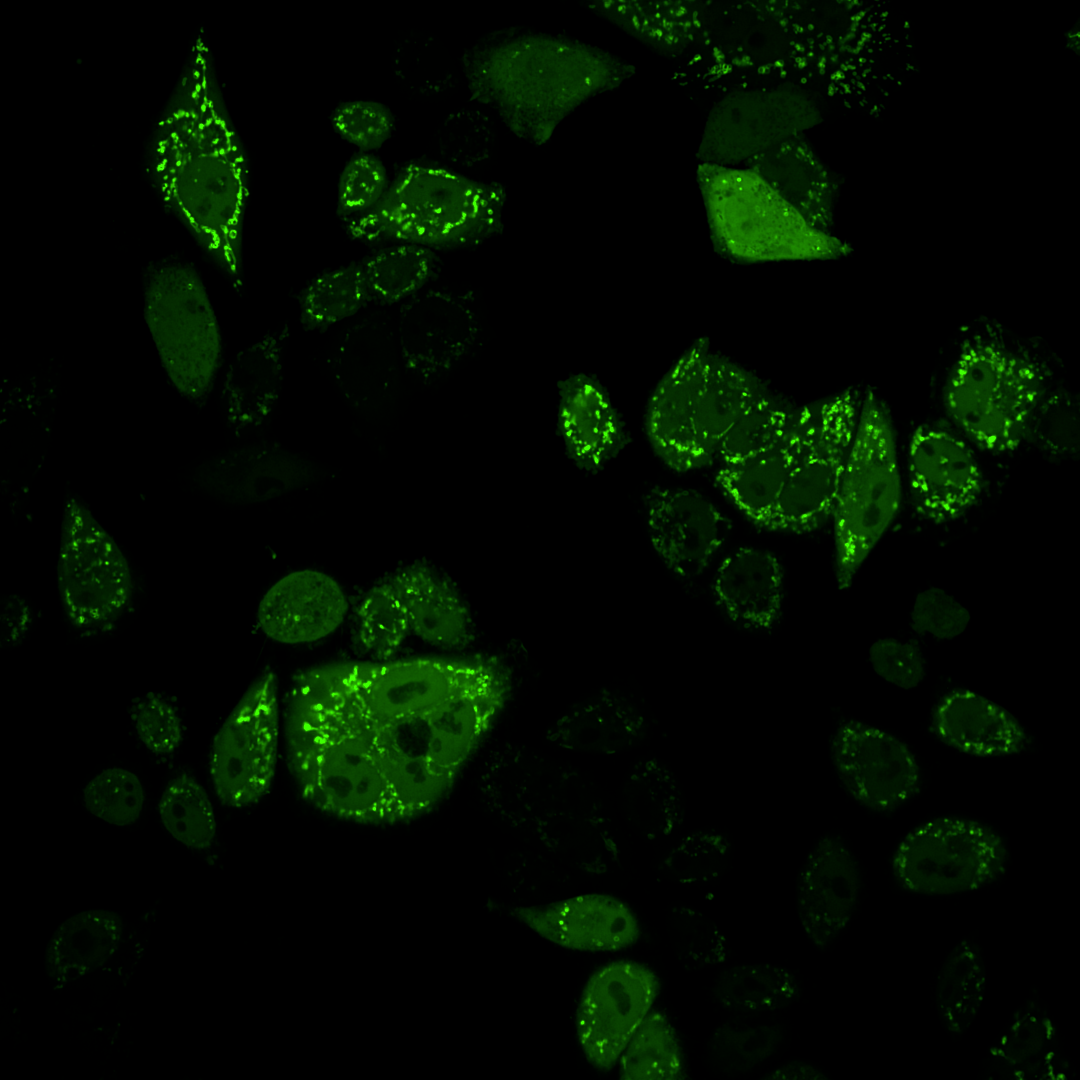

Supplement: Supplementary file 5 — Source data Fig. 1 [file 44321_2025_323_MOESM5_ESM.zip › Figure 1/1I/si-nc ISO/1.png]

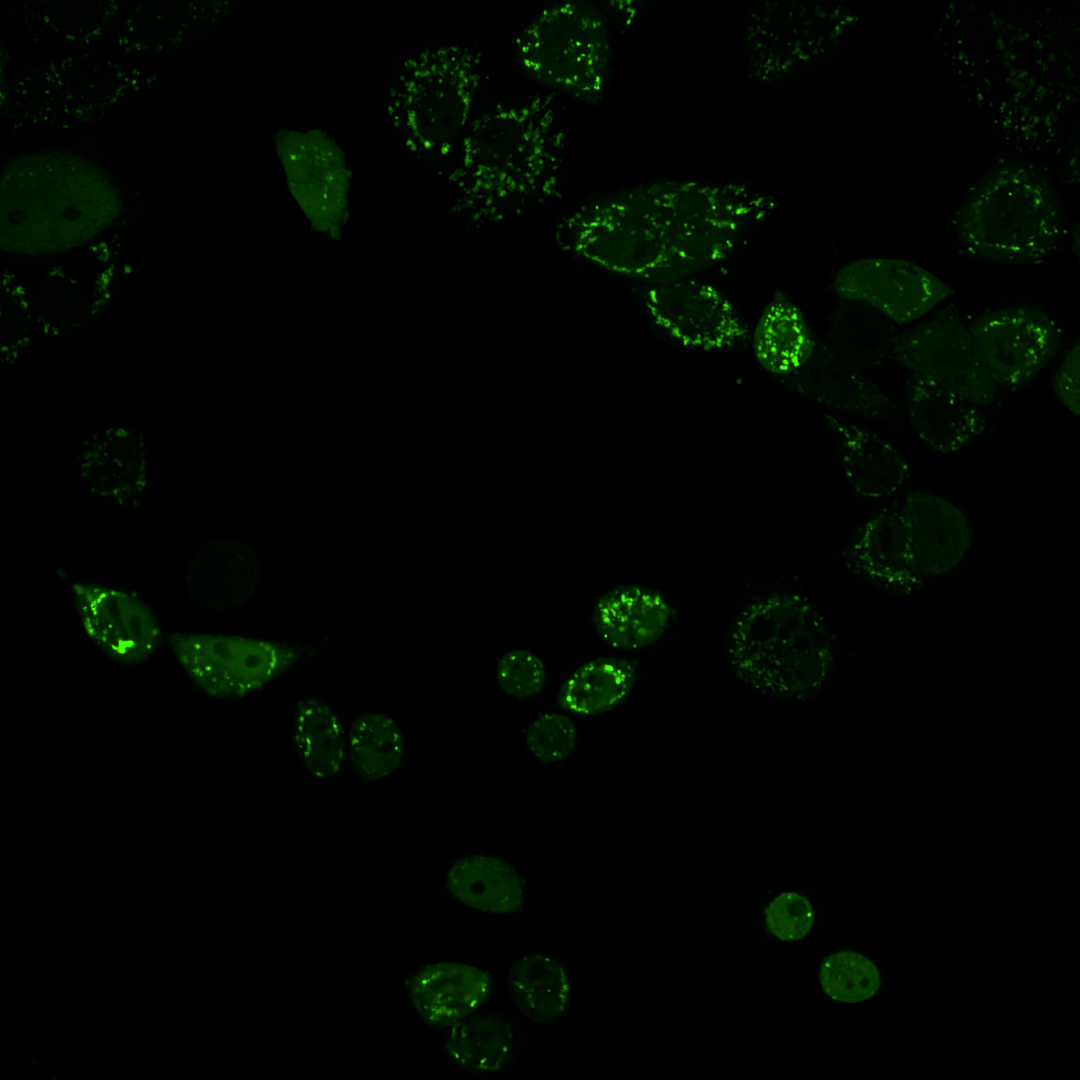

Supplement: Supplementary file 5 — Source data Fig. 1 [file 44321_2025_323_MOESM5_ESM.zip › Figure 1/1I/si-nc ISO/2.png]

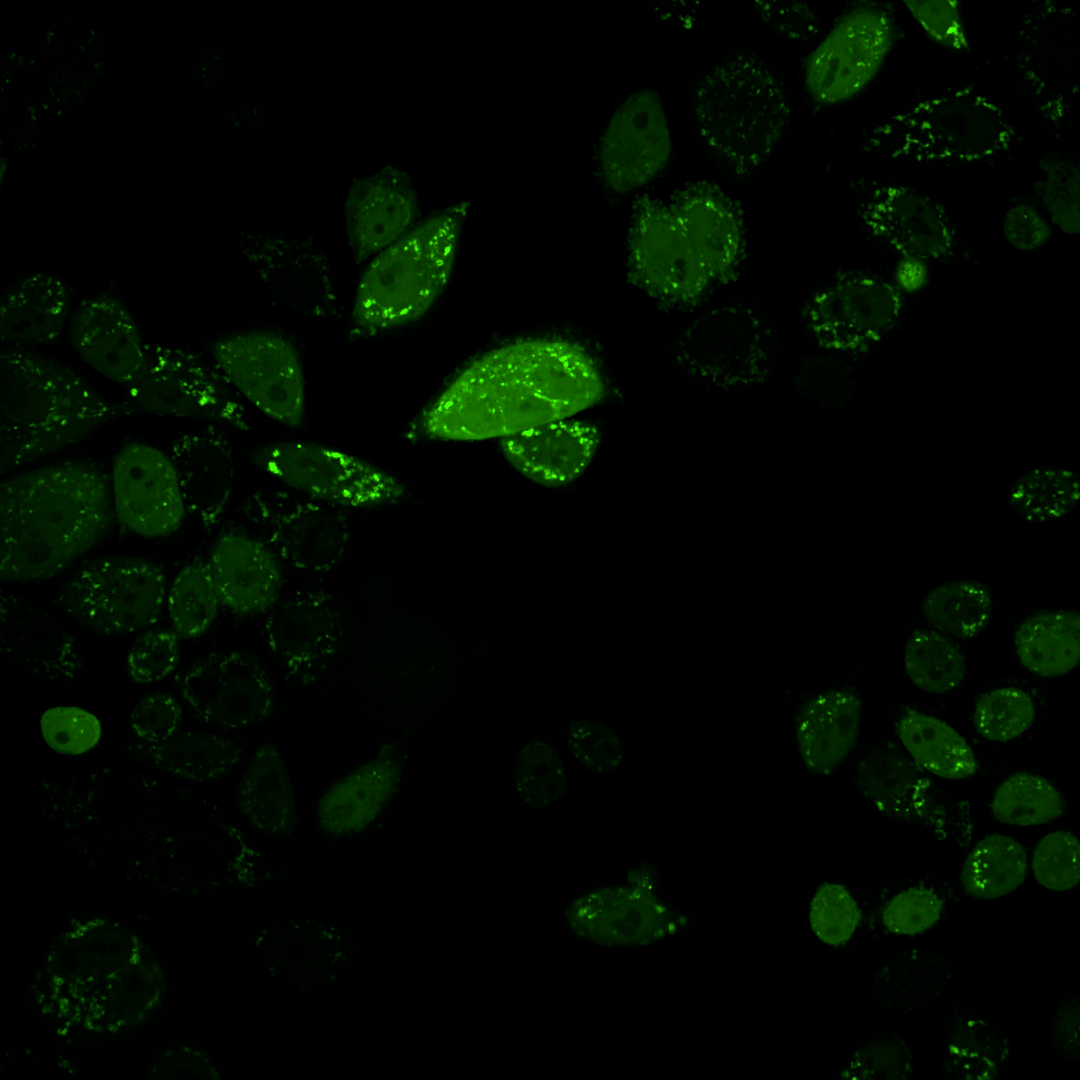

Supplement: Supplementary file 5 — Source data Fig. 1 [file 44321_2025_323_MOESM5_ESM.zip › Figure 1/1I/si-nc ISO/3.png]

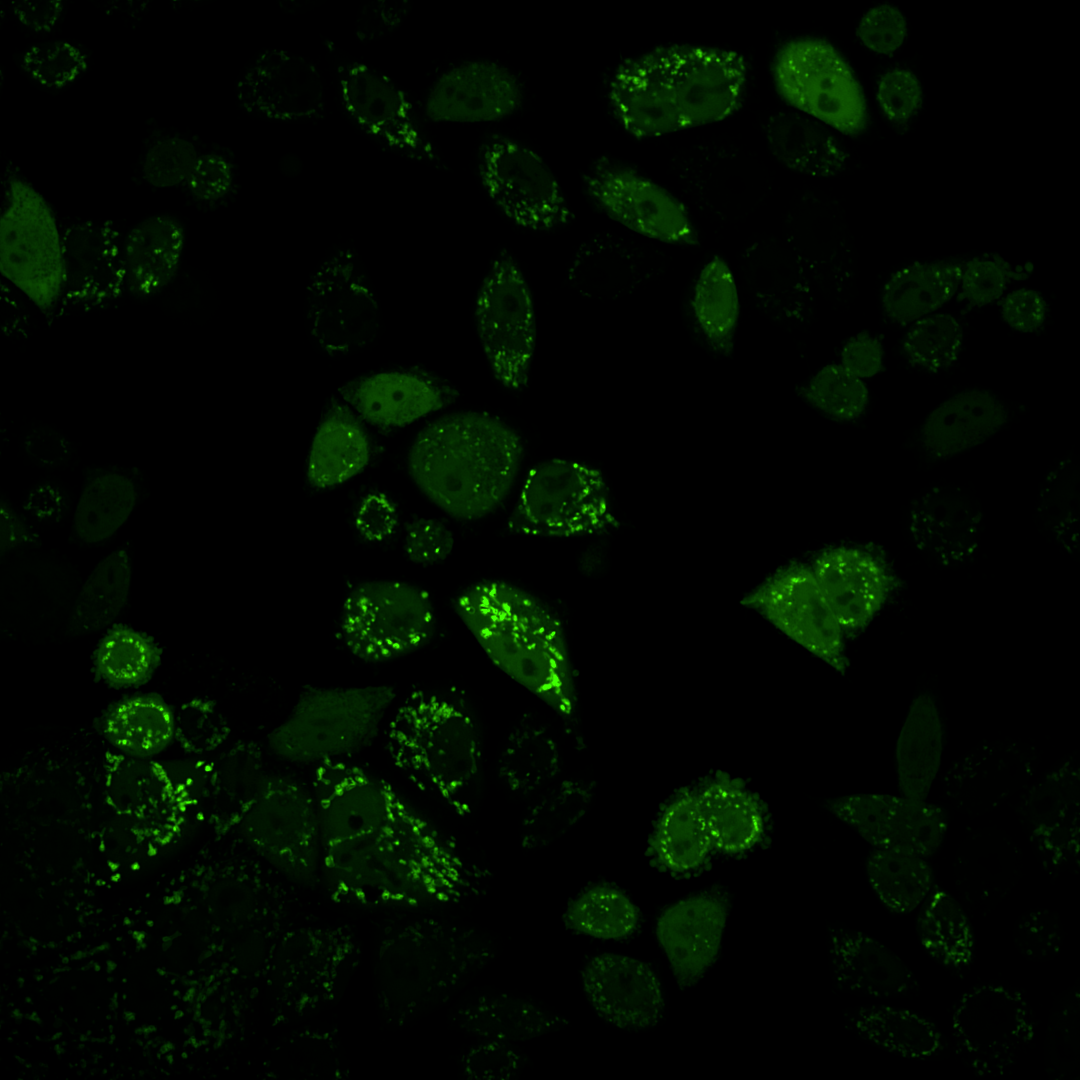

Supplement: Supplementary file 5 — Source data Fig. 1 [file 44321_2025_323_MOESM5_ESM.zip › Figure 1/1I/si-nc ISO/4.png]

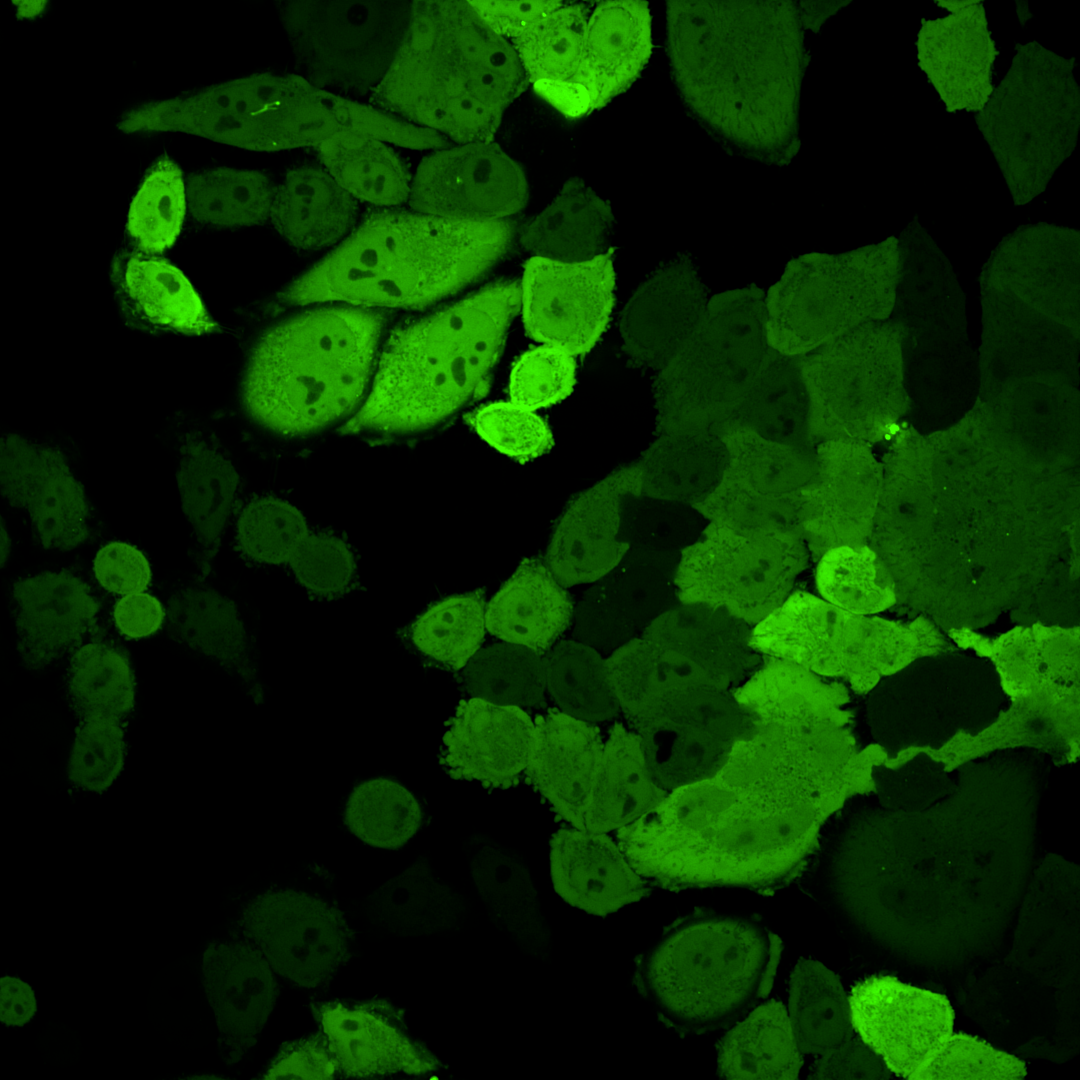

Supplement: Supplementary file 5 — Source data Fig. 1 [file 44321_2025_323_MOESM5_ESM.zip › Figure 1/1I/si-nc Veh/1.png]

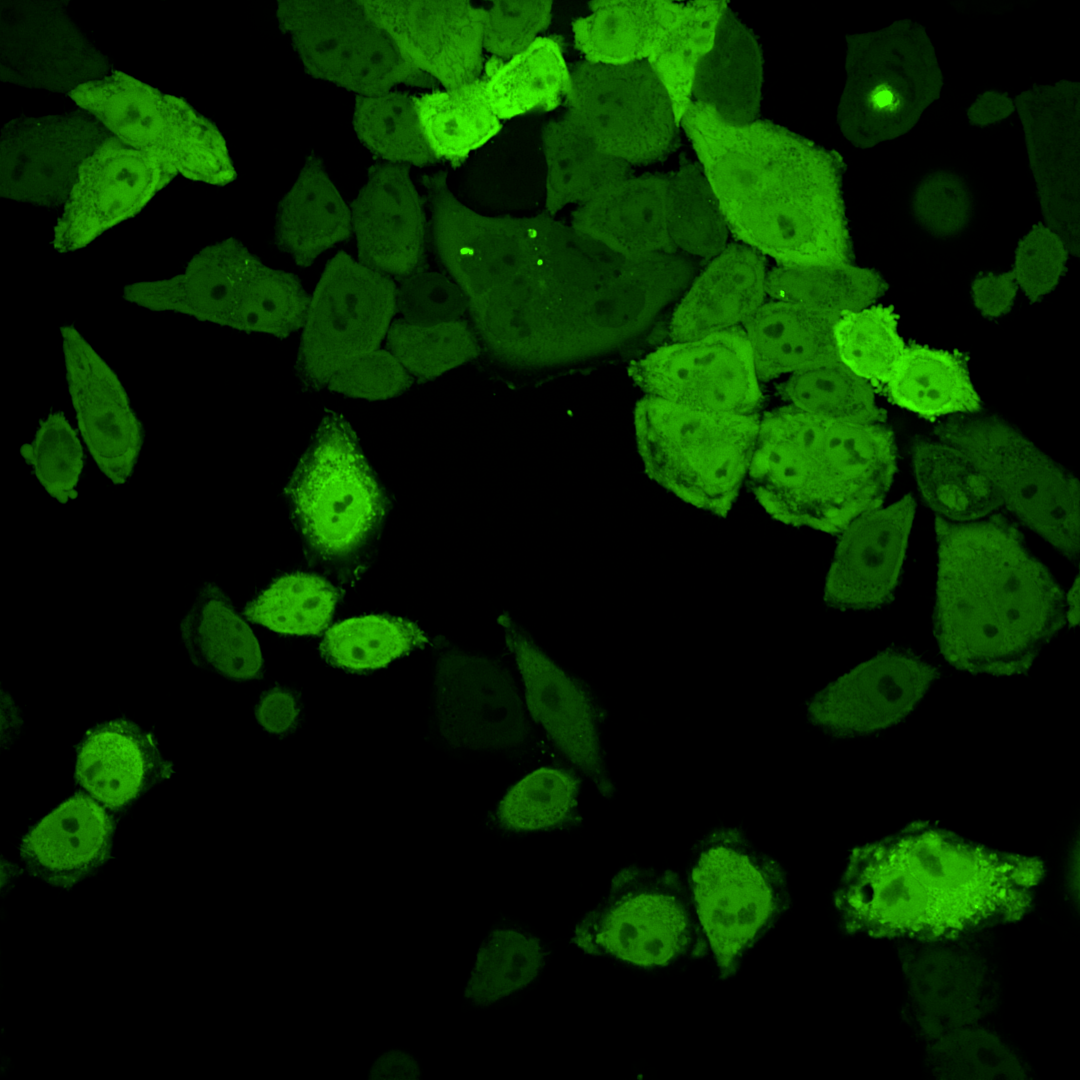

Supplement: Supplementary file 5 — Source data Fig. 1 [file 44321_2025_323_MOESM5_ESM.zip › Figure 1/1I/si-nc Veh/2.png]

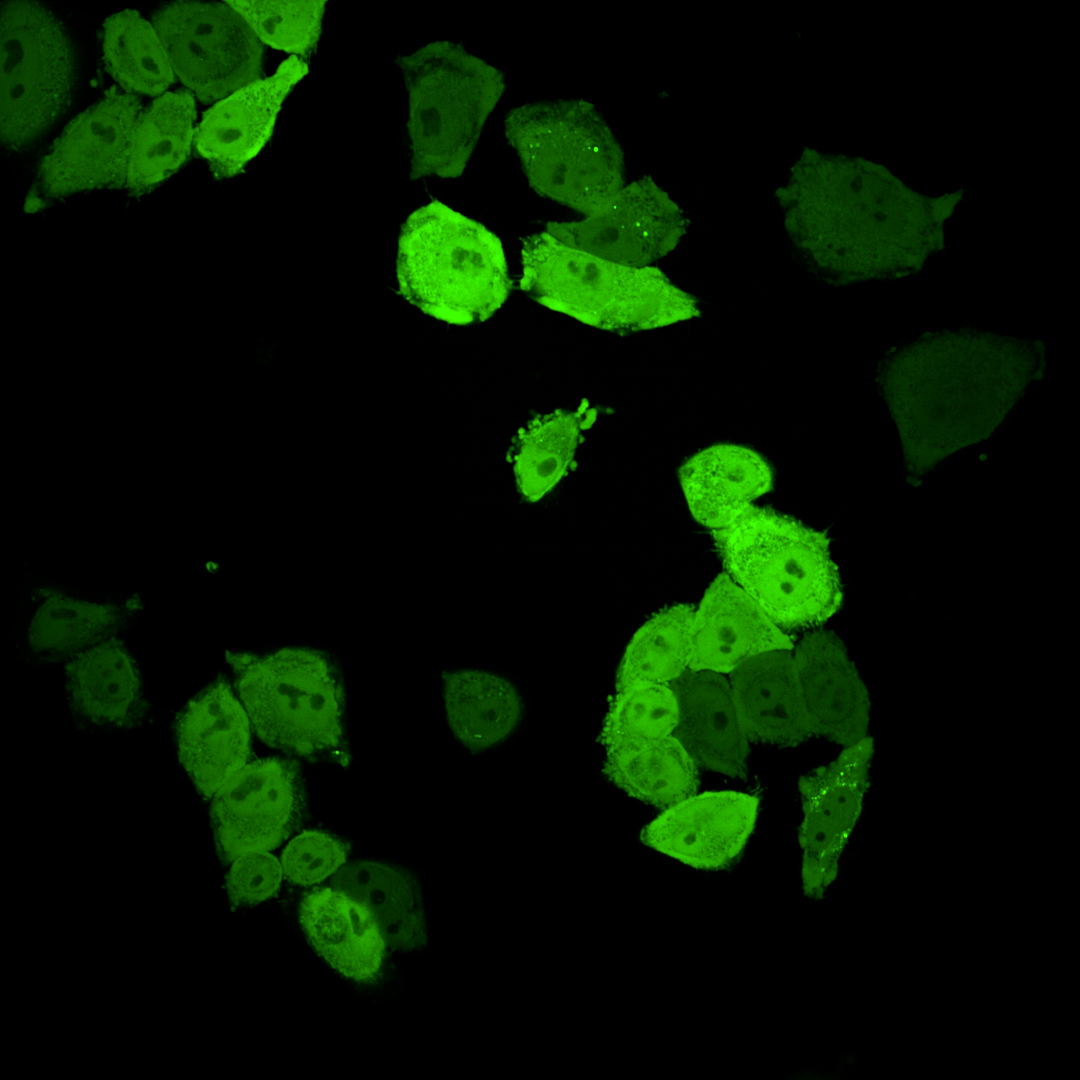

Supplement: Supplementary file 5 — Source data Fig. 1 [file 44321_2025_323_MOESM5_ESM.zip › Figure 1/1I/si-nc Veh/3.png]

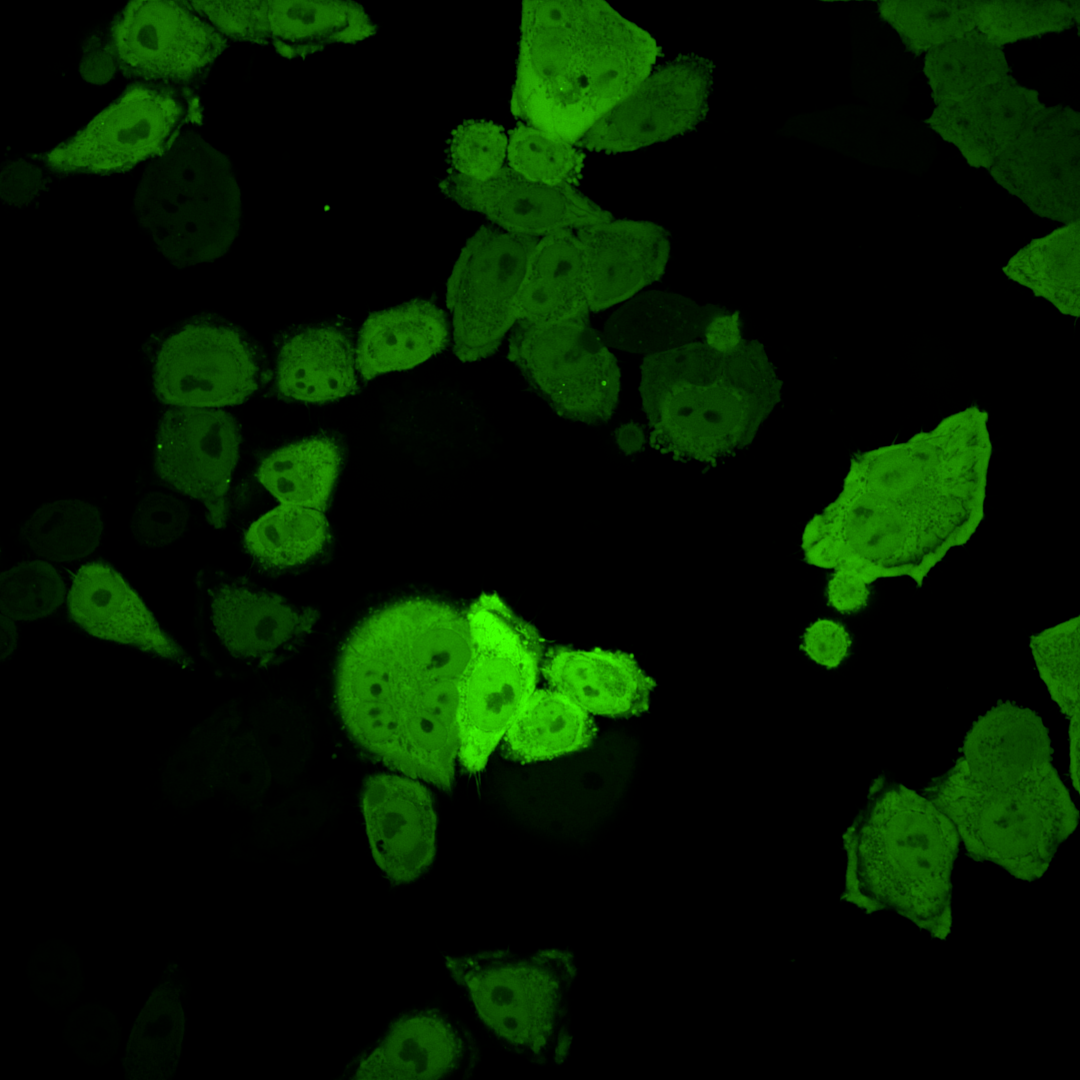

Supplement: Supplementary file 5 — Source data Fig. 1 [file 44321_2025_323_MOESM5_ESM.zip › Figure 1/1I/si-nc Veh/4.png]

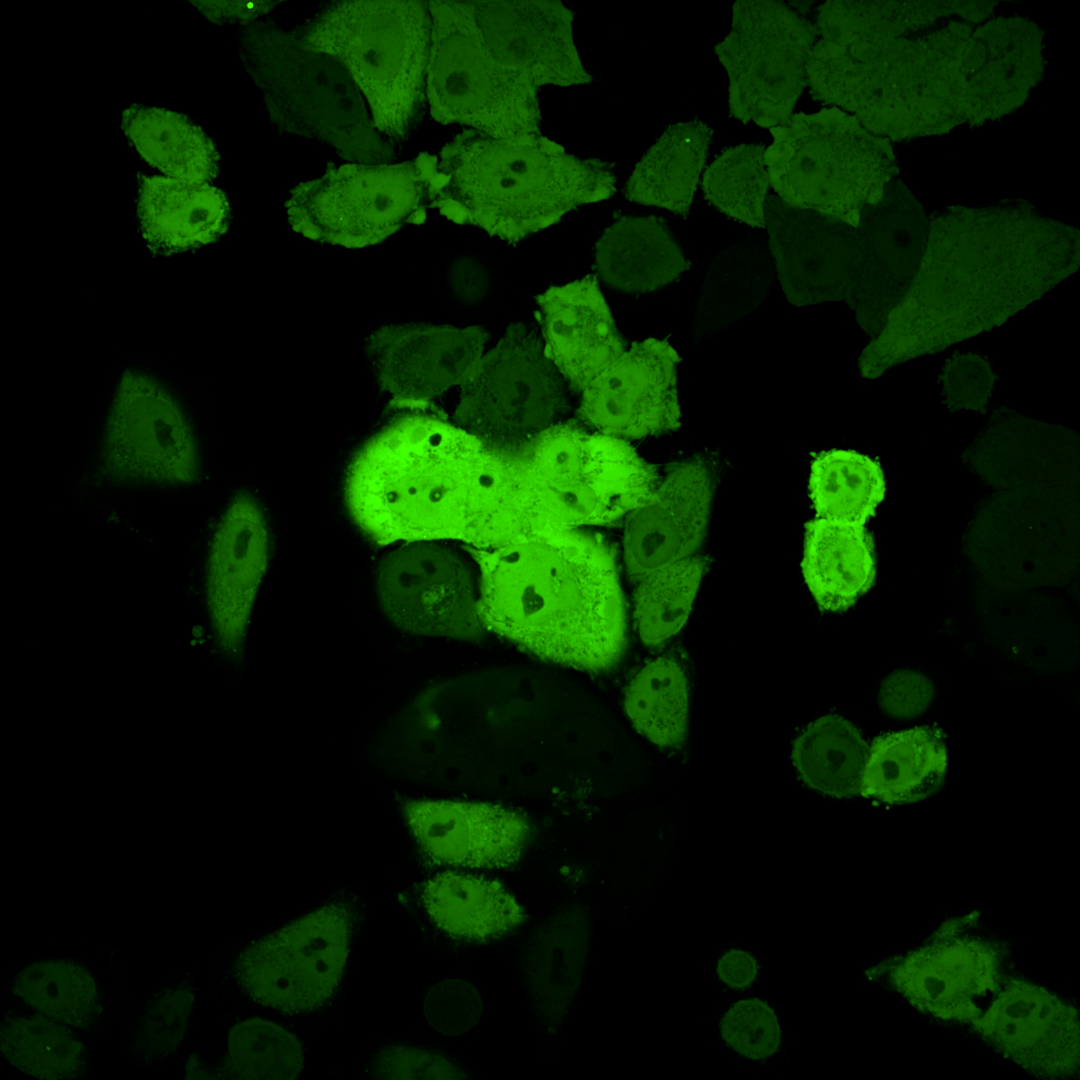

Supplement: Supplementary file 5 — Source data Fig. 1 [file 44321_2025_323_MOESM5_ESM.zip › Figure 1/1I/si-PINK1 CCCP/2.png]

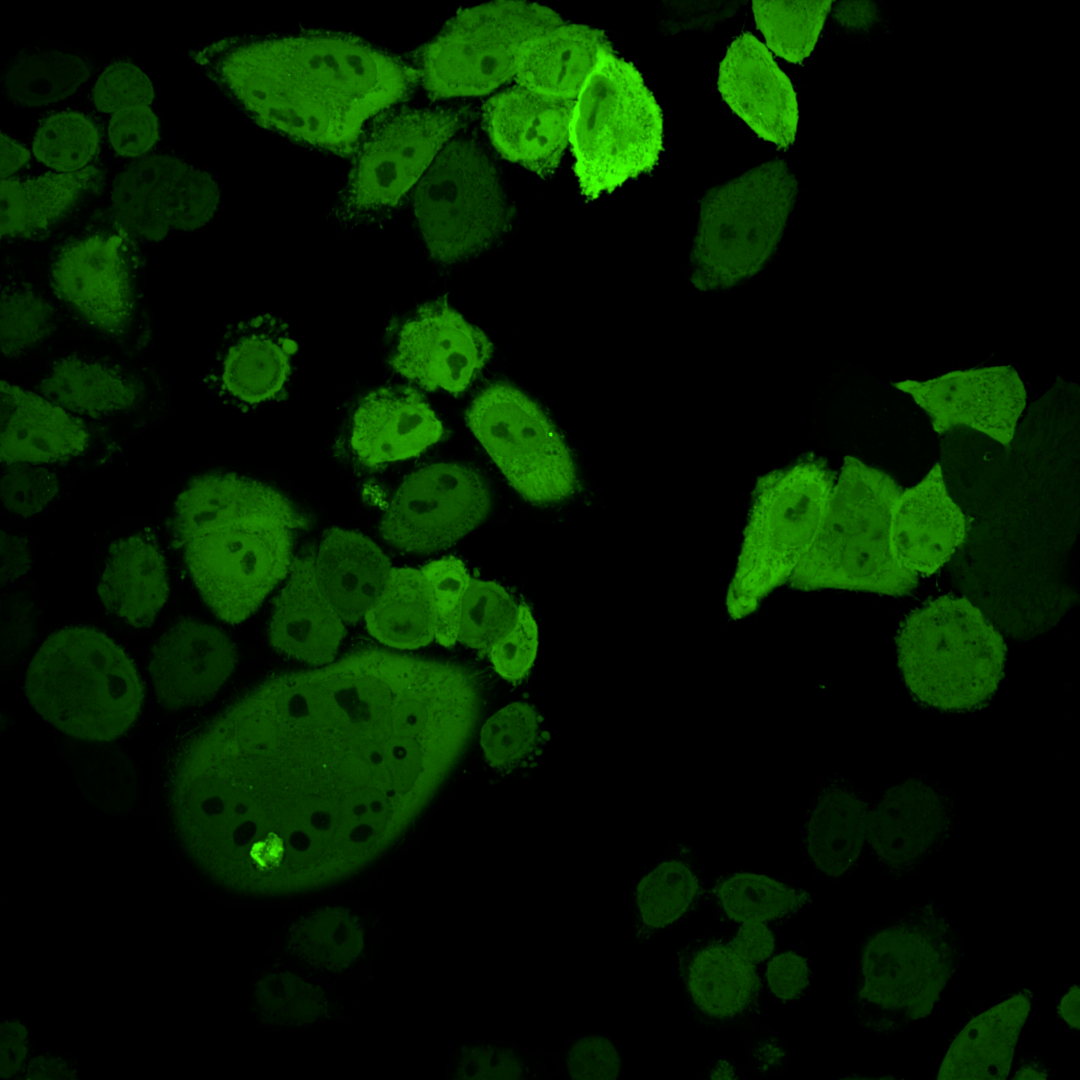

Supplement: Supplementary file 5 — Source data Fig. 1 [file 44321_2025_323_MOESM5_ESM.zip › Figure 1/1I/si-PINK1 CCCP/3.png]

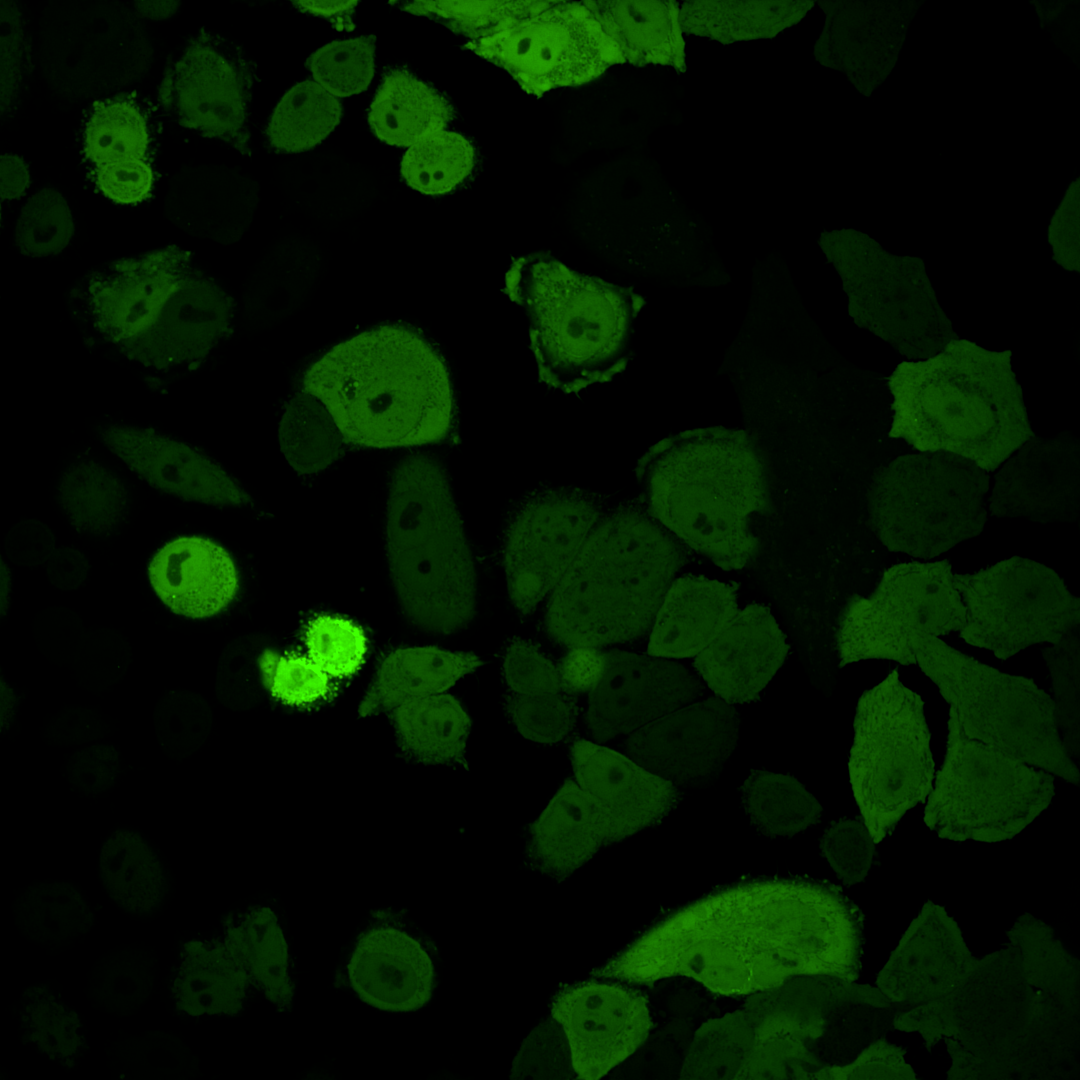

Supplement: Supplementary file 5 — Source data Fig. 1 [file 44321_2025_323_MOESM5_ESM.zip › Figure 1/1I/si-PINK1 CCCP/4.png]

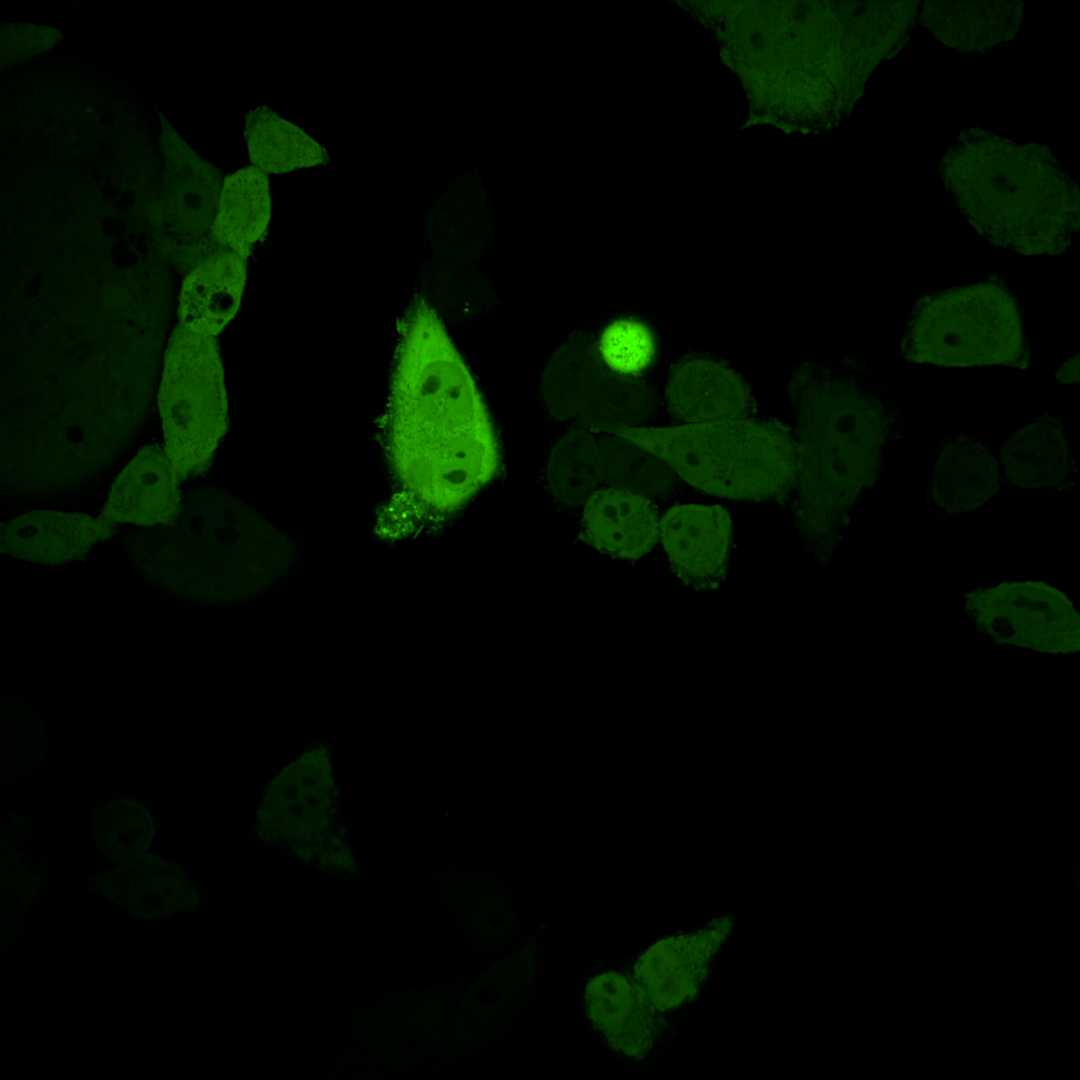

Supplement: Supplementary file 5 — Source data Fig. 1 [file 44321_2025_323_MOESM5_ESM.zip › Figure 1/1I/si-PINK1 ISO/1.png]

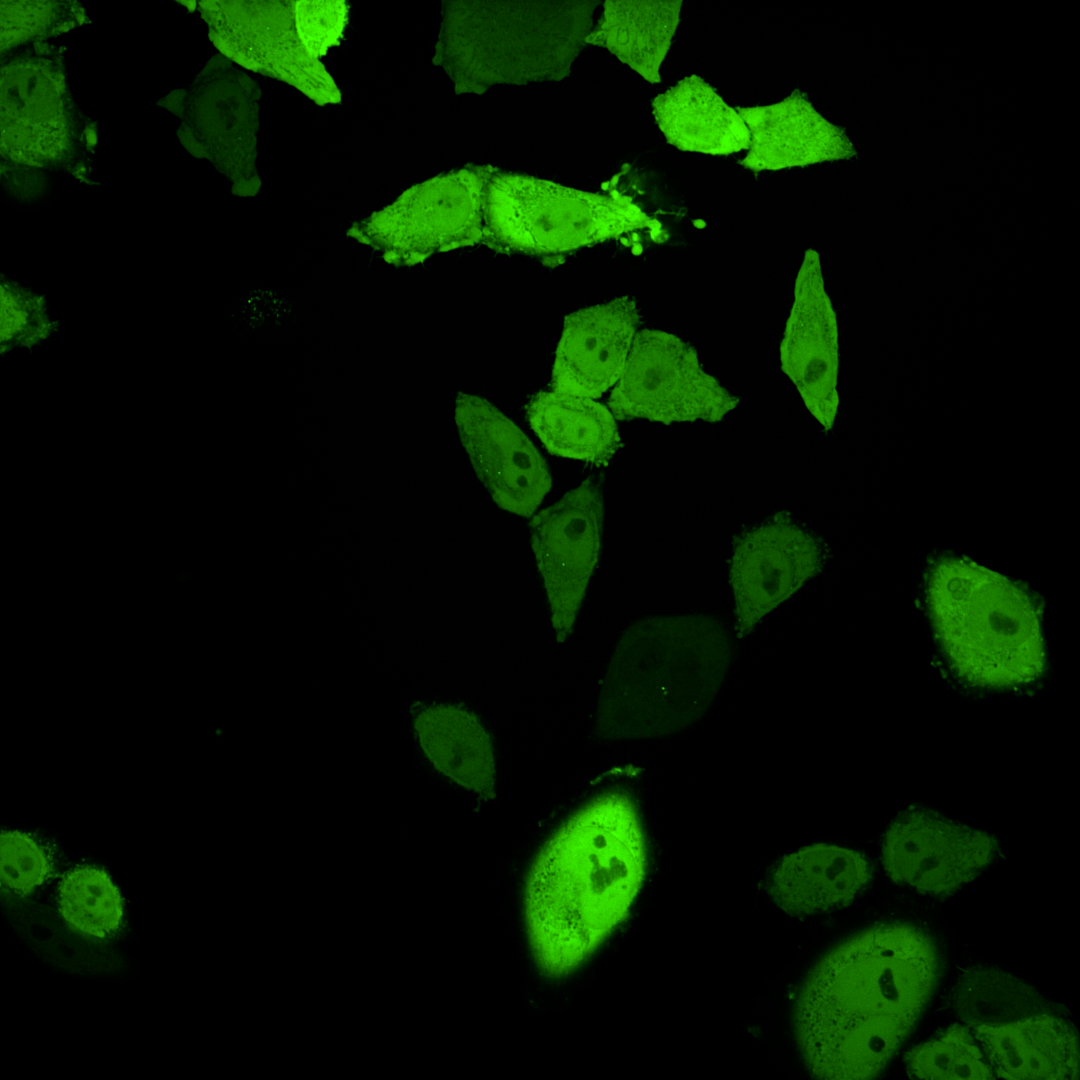

Supplement: Supplementary file 5 — Source data Fig. 1 [file 44321_2025_323_MOESM5_ESM.zip › Figure 1/1I/si-PINK1 ISO/2.png]

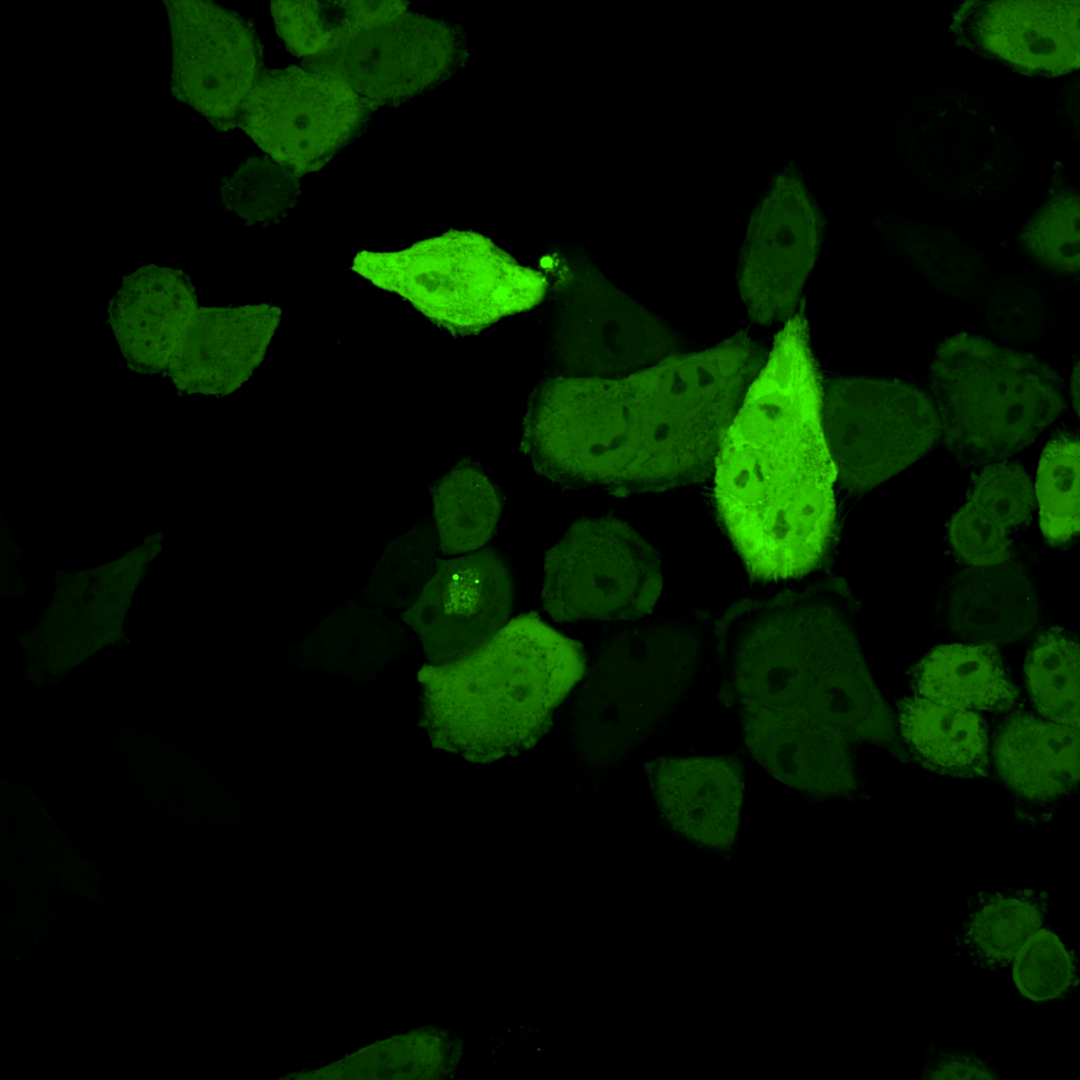

Supplement: Supplementary file 5 — Source data Fig. 1 [file 44321_2025_323_MOESM5_ESM.zip › Figure 1/1I/si-PINK1 ISO/3.png]

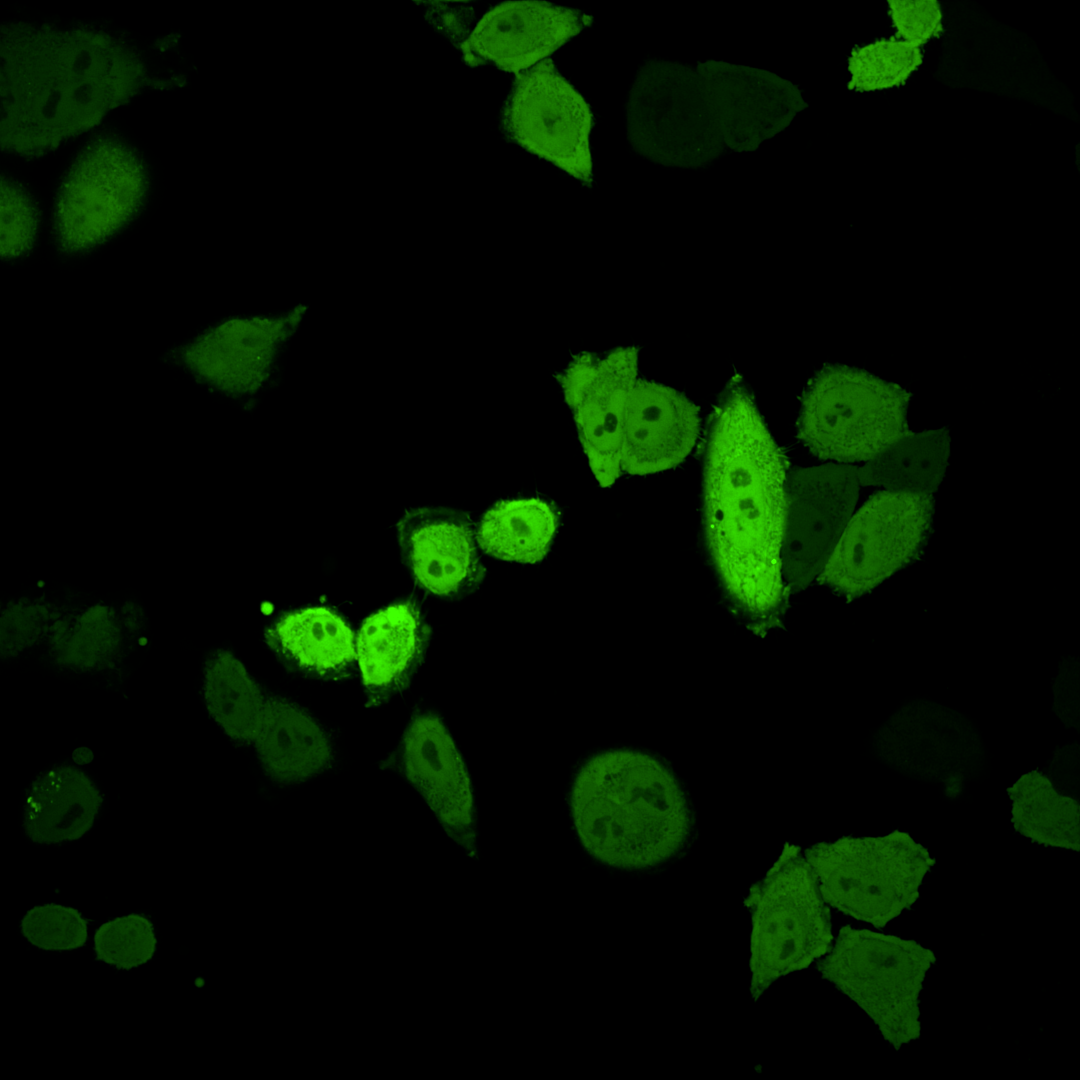

Supplement: Supplementary file 5 — Source data Fig. 1 [file 44321_2025_323_MOESM5_ESM.zip › Figure 1/1I/si-PINK1 ISO/4.png]

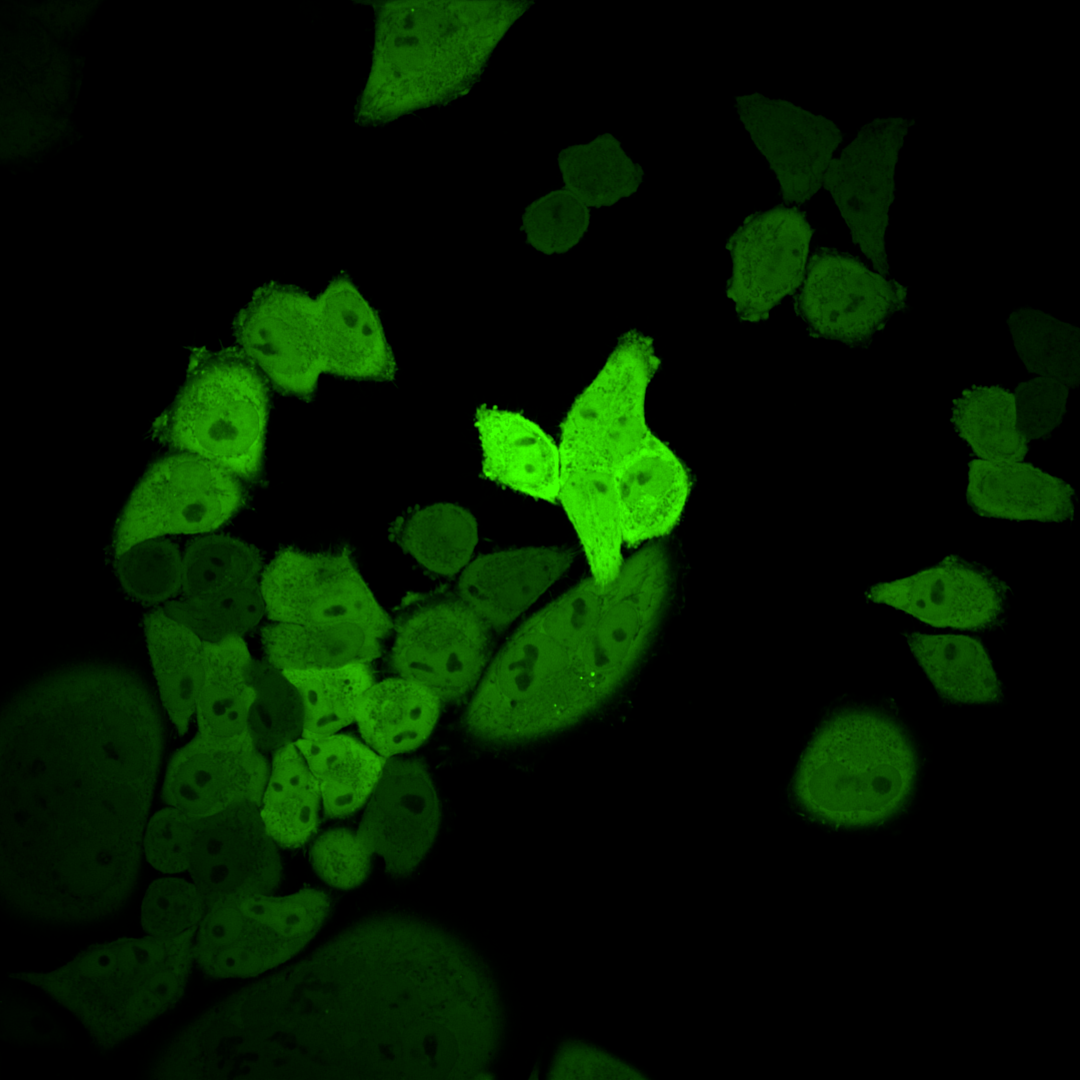

Supplement: Supplementary file 5 — Source data Fig. 1 [file 44321_2025_323_MOESM5_ESM.zip › Figure 1/1I/si-PINK1 Veh/1.png]

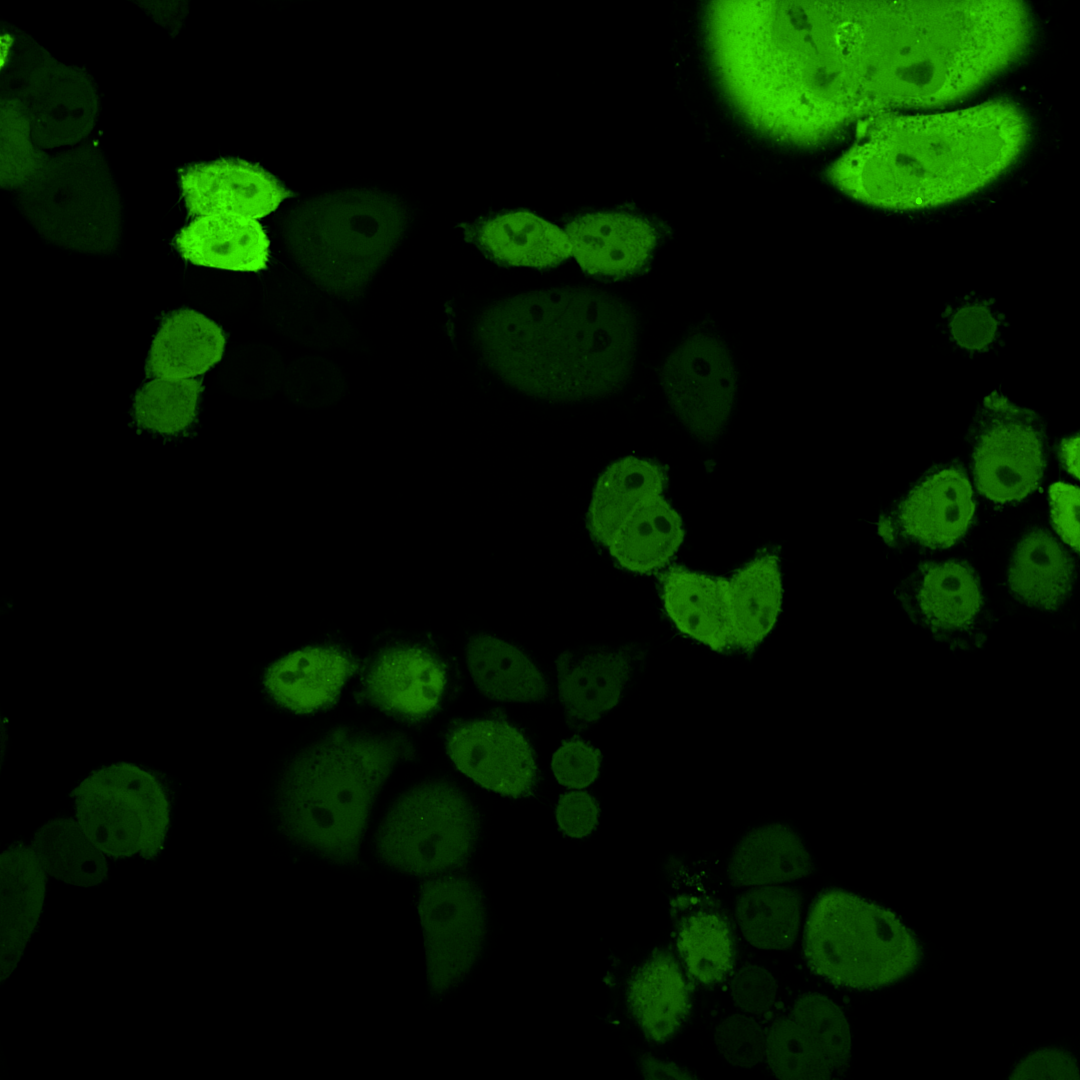

Supplement: Supplementary file 5 — Source data Fig. 1 [file 44321_2025_323_MOESM5_ESM.zip › Figure 1/1I/si-PINK1 Veh/2.png]

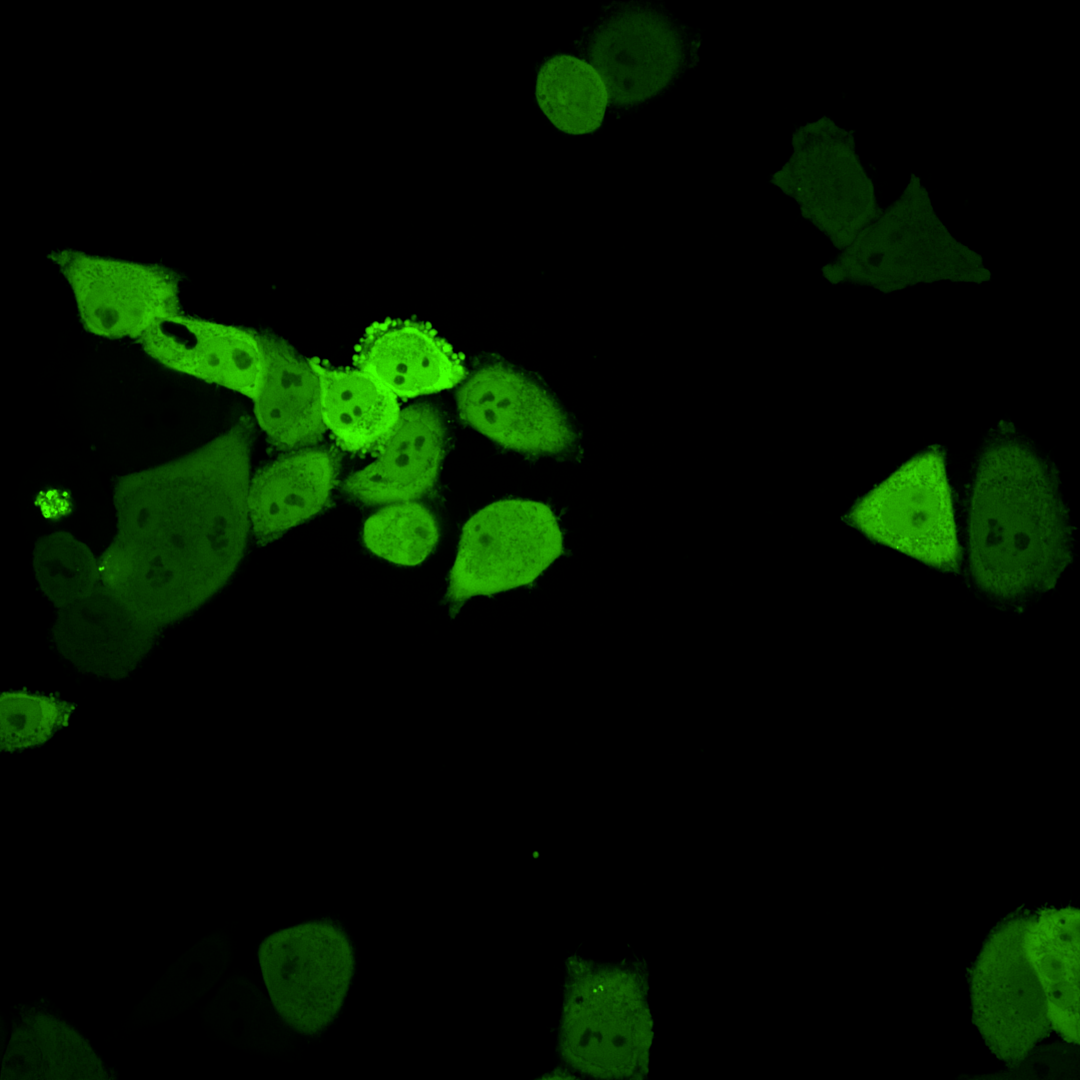

Supplement: Supplementary file 5 — Source data Fig. 1 [file 44321_2025_323_MOESM5_ESM.zip › Figure 1/1I/si-PINK1 Veh/3.png]

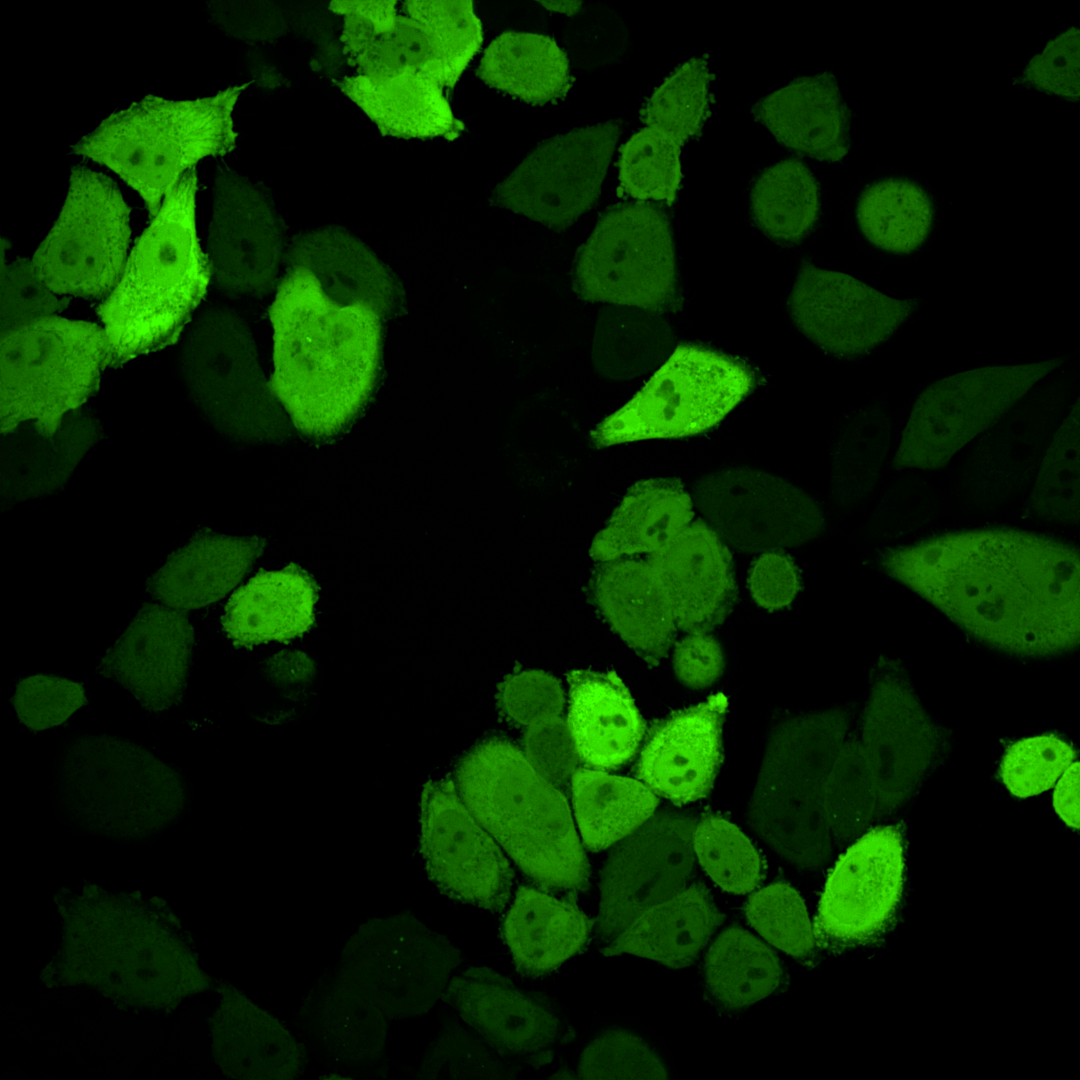

Supplement: Supplementary file 5 — Source data Fig. 1 [file 44321_2025_323_MOESM5_ESM.zip › Figure 1/1I/si-PINK1 Veh/4.png]

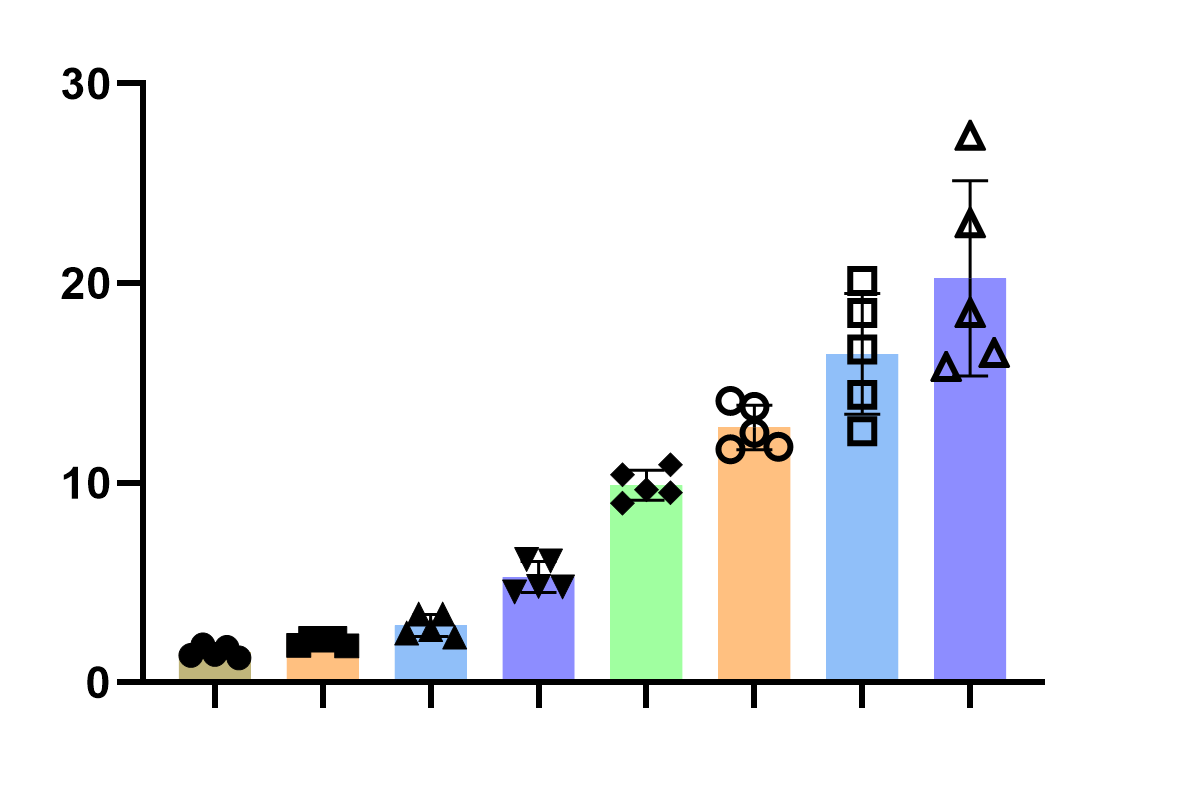

Supplement: Supplementary file 6 — Source data Fig. 2 [file 44321_2025_323_MOESM6_ESM.zip › Figure 2/2A-B/mkeima mitophagy.tif]

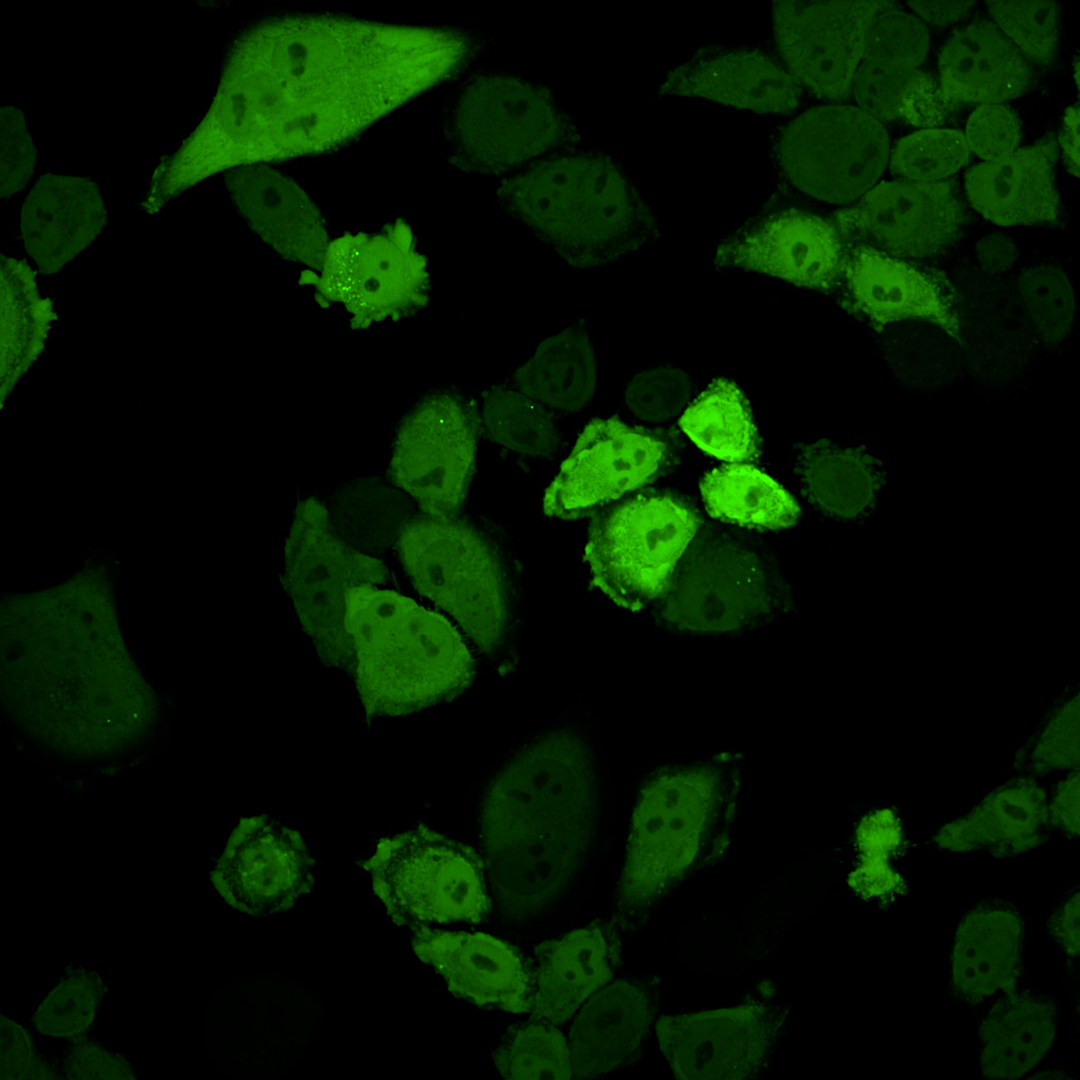

Supplement: Supplementary file 6 — Source data Fig. 2 [file 44321_2025_323_MOESM6_ESM.zip › Figure 2/2C/CCCP 0min.png]

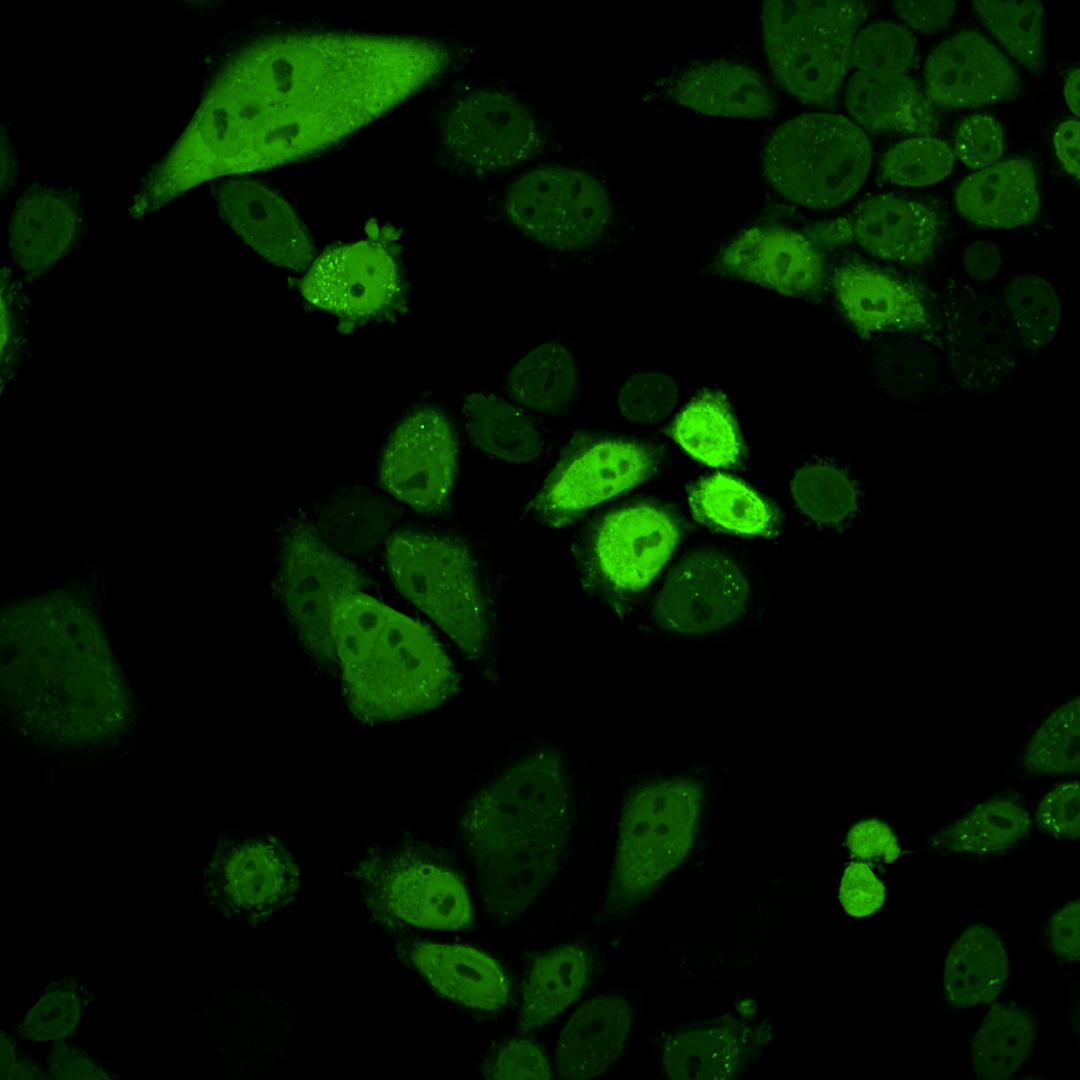

Supplement: Supplementary file 6 — Source data Fig. 2 [file 44321_2025_323_MOESM6_ESM.zip › Figure 2/2C/CCCP 15min.png]

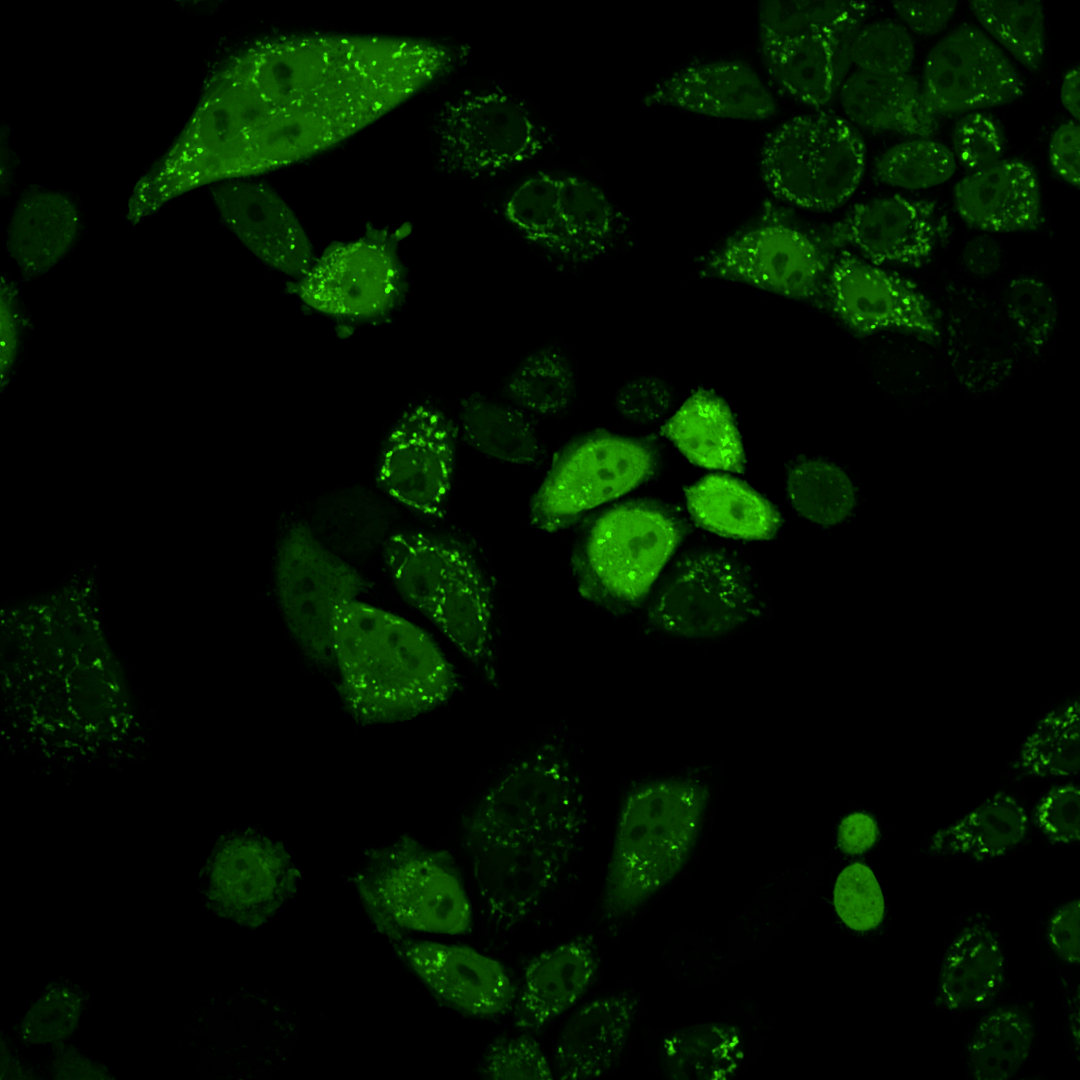

Supplement: Supplementary file 6 — Source data Fig. 2 [file 44321_2025_323_MOESM6_ESM.zip › Figure 2/2C/CCCP 30min.png]

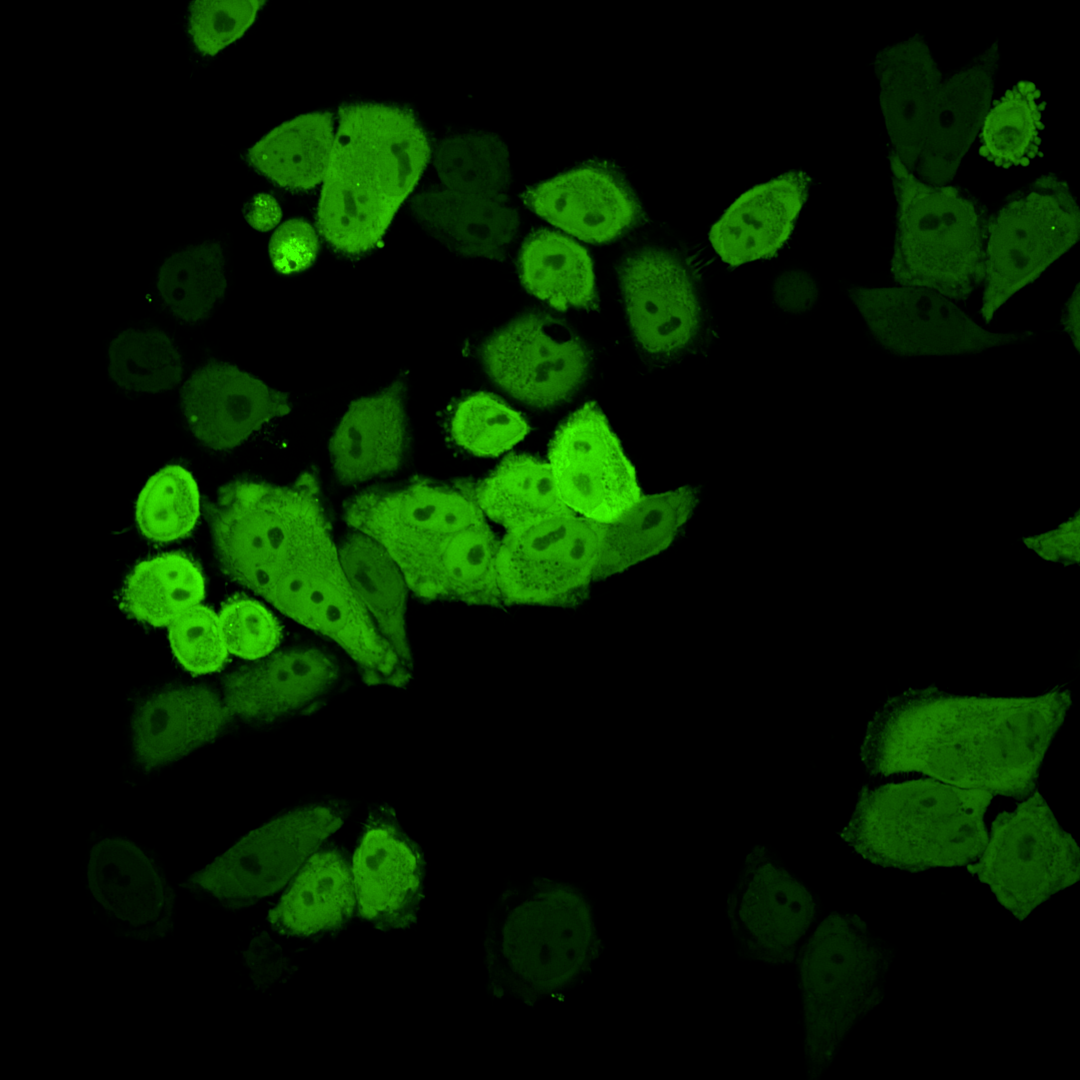

Supplement: Supplementary file 6 — Source data Fig. 2 [file 44321_2025_323_MOESM6_ESM.zip › Figure 2/2C/ISO 0min.png]

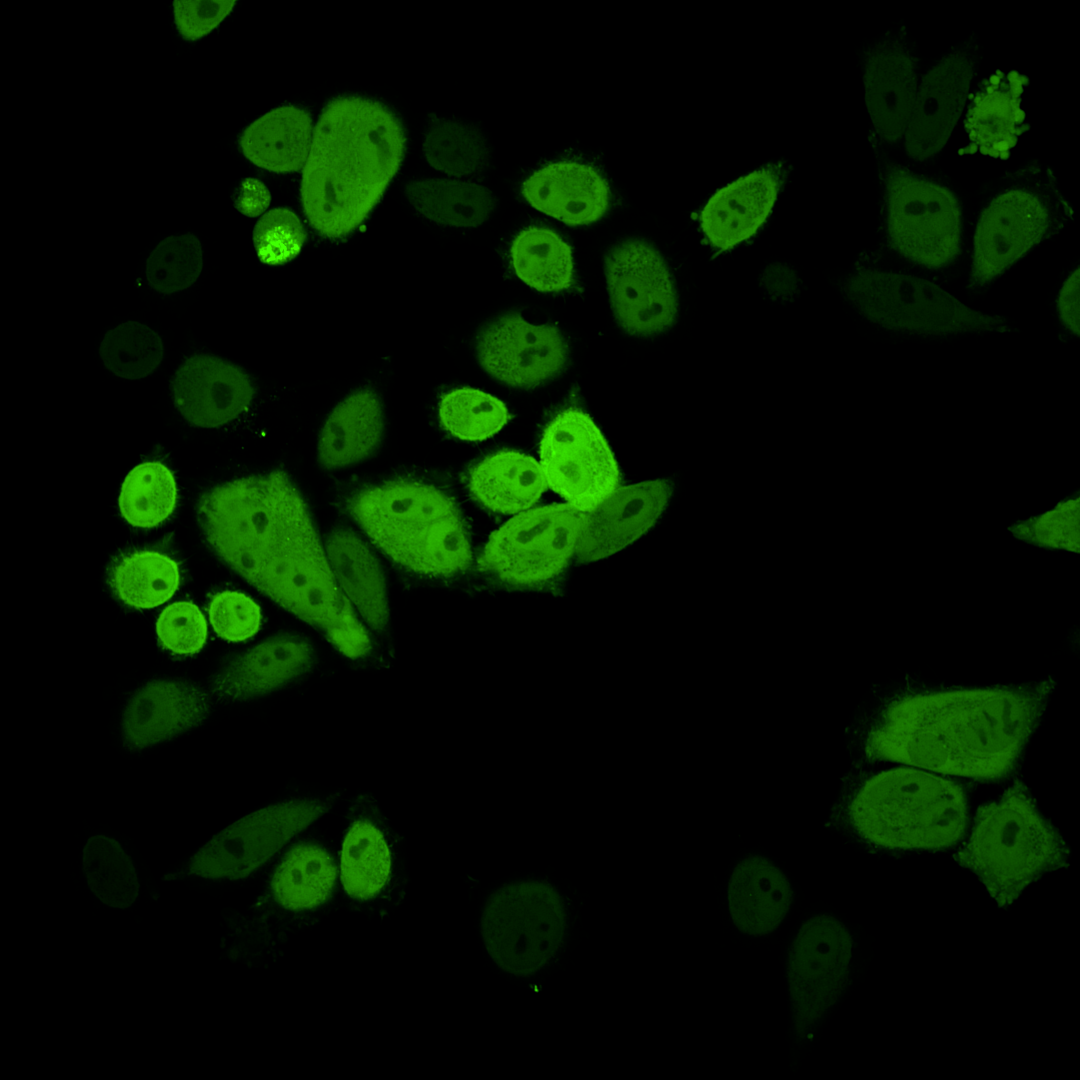

Supplement: Supplementary file 6 — Source data Fig. 2 [file 44321_2025_323_MOESM6_ESM.zip › Figure 2/2C/ISO 15min.png]

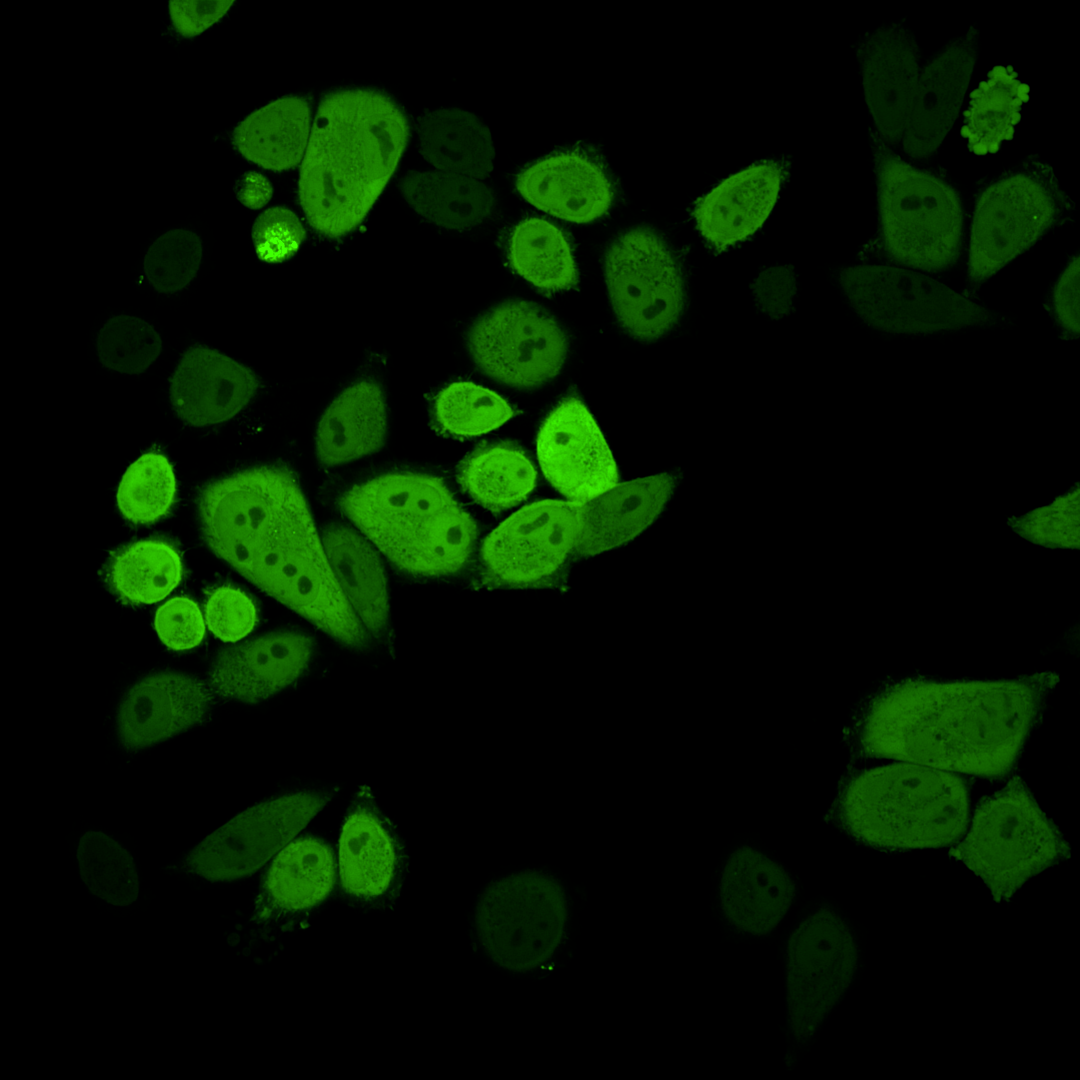

Supplement: Supplementary file 6 — Source data Fig. 2 [file 44321_2025_323_MOESM6_ESM.zip › Figure 2/2C/ISO 30min.png]

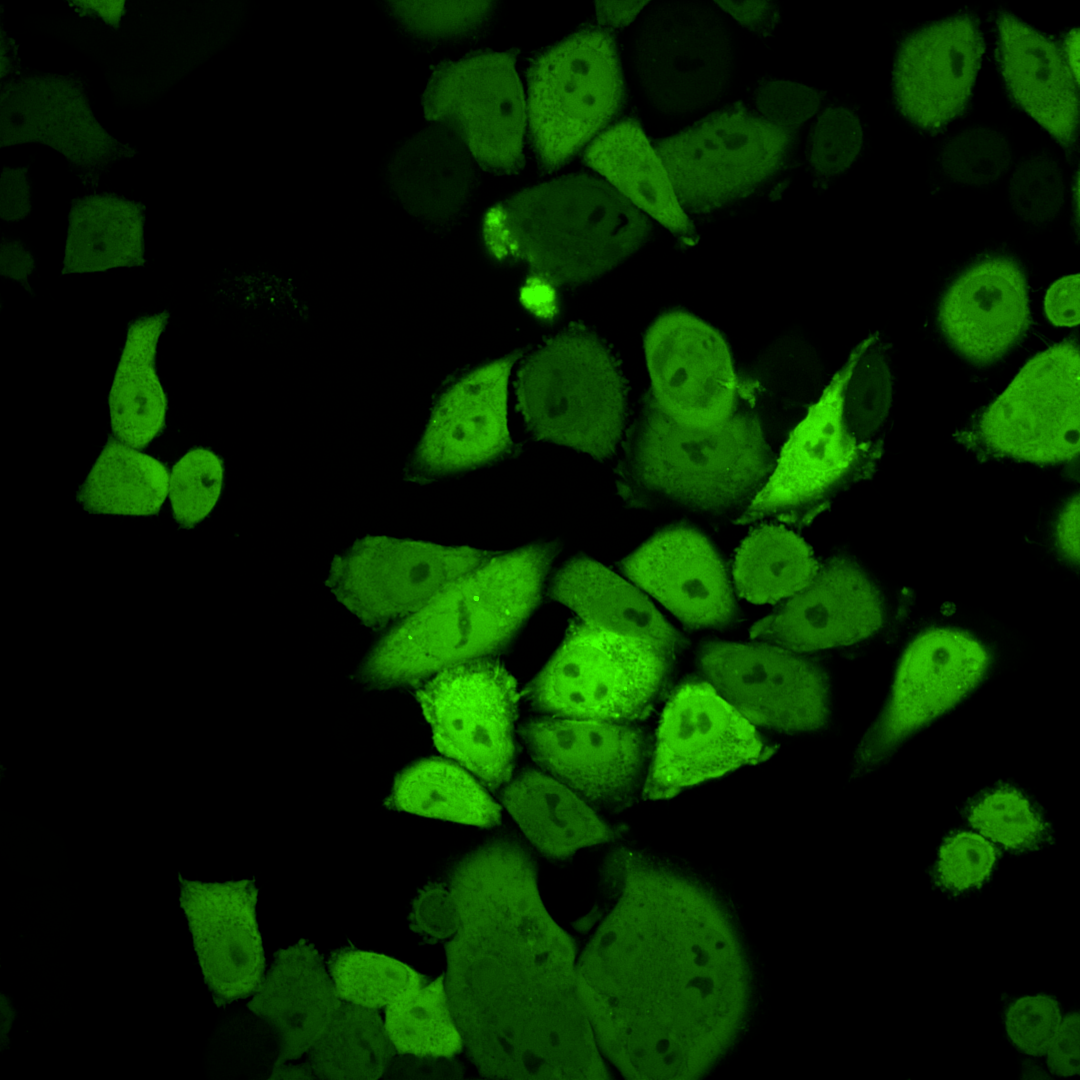

Supplement: Supplementary file 6 — Source data Fig. 2 [file 44321_2025_323_MOESM6_ESM.zip › Figure 2/2C/ISO+CCCP 0min.png]

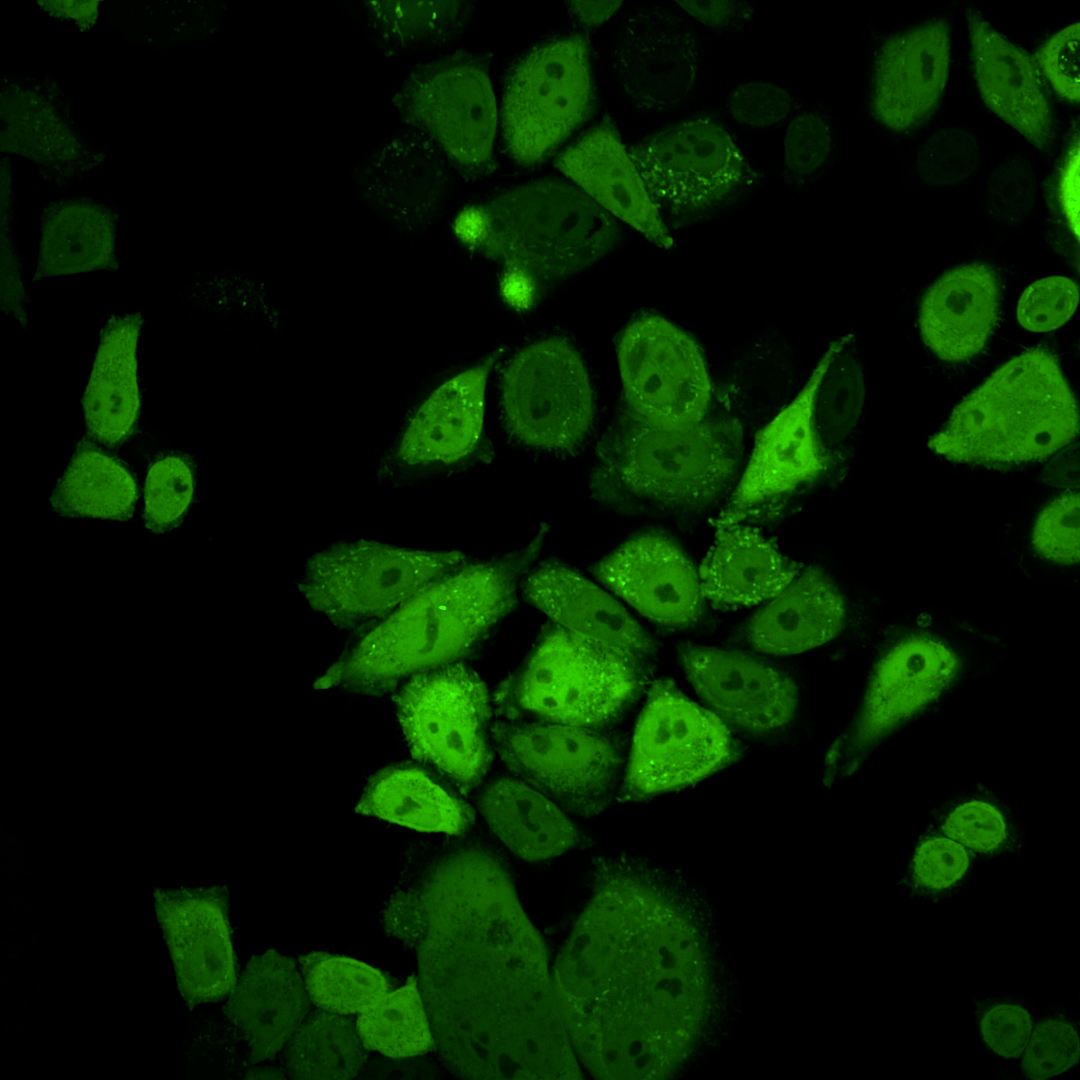

Supplement: Supplementary file 6 — Source data Fig. 2 [file 44321_2025_323_MOESM6_ESM.zip › Figure 2/2C/ISO+CCCP 15min.png]

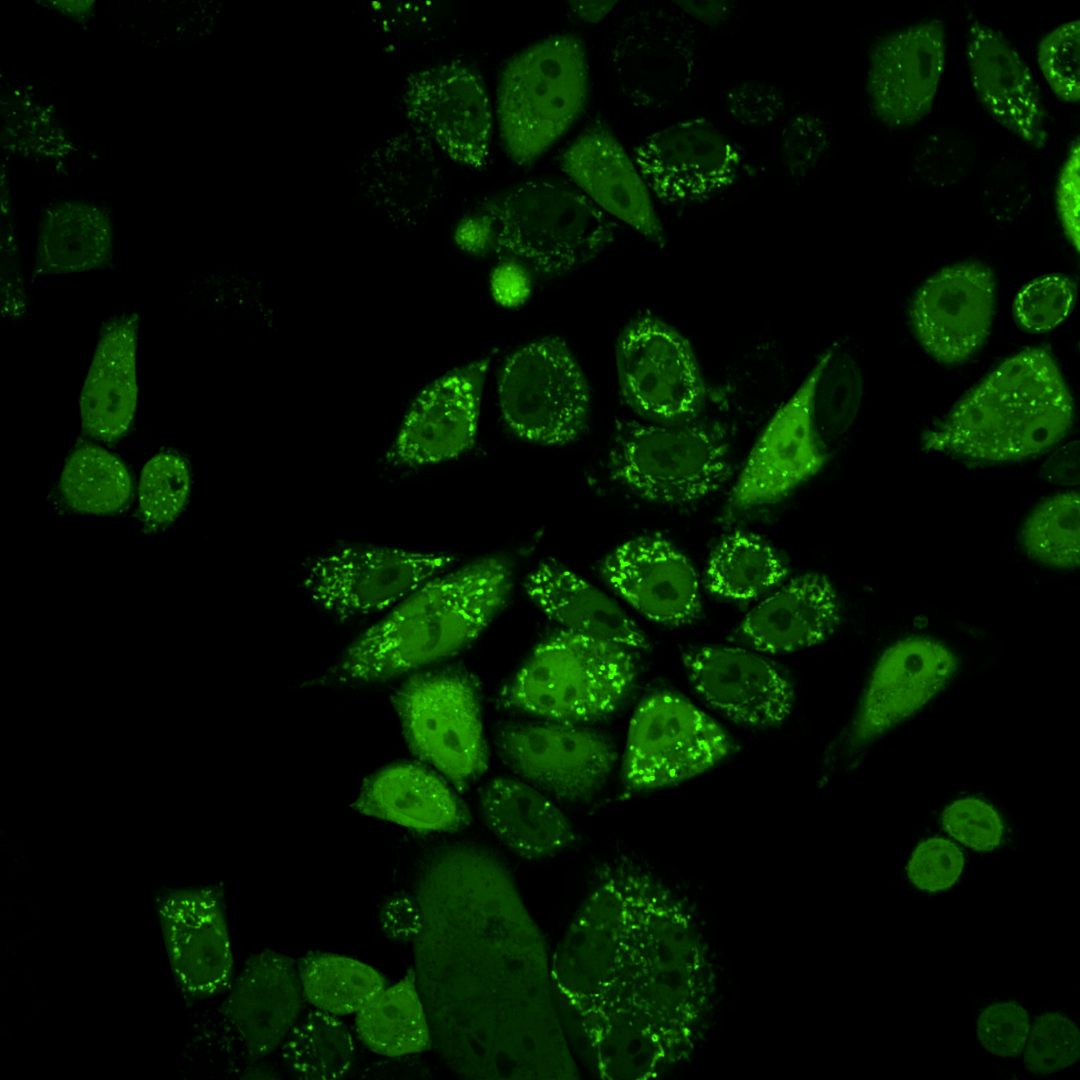

Supplement: Supplementary file 6 — Source data Fig. 2 [file 44321_2025_323_MOESM6_ESM.zip › Figure 2/2C/ISO+CCCP 30min.png]

## Slide 1
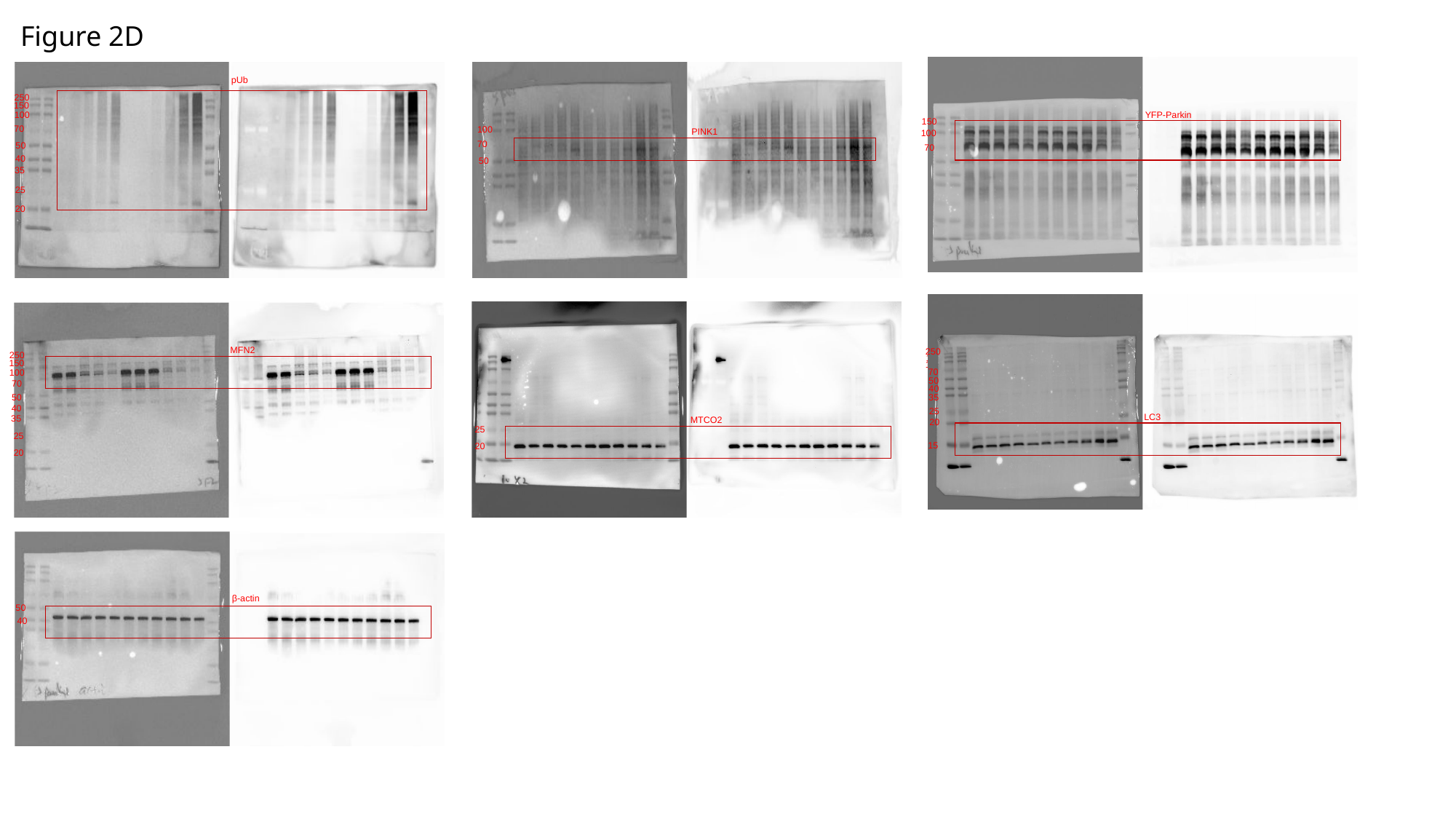

Figure 2D
YFP-Parkin
pUb
250
150
100
70
50
40
35
25
20
100
PINK1
70
50
150
100
70
70
50
40
35
25
LC3
20
15
250
150
100
MFN2
250
150
100
70
50
40
35
25
20
MTCO2
25
20
β-actin
50
40

## Slide 2
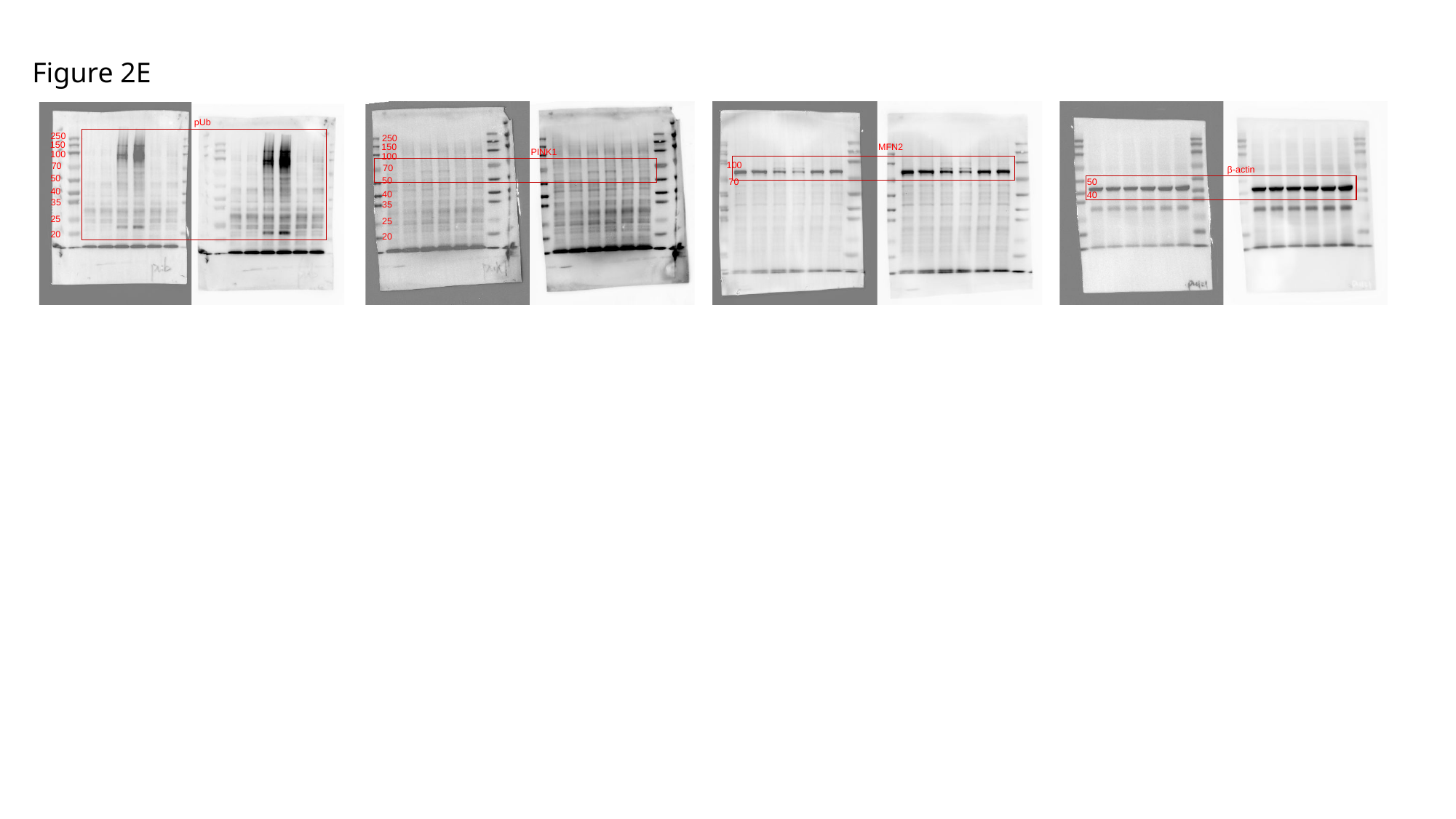

Figure 2E
250
150
PINK1
100
70
50
40
35
25
20
MFN2
100
70
β-actin
50
40
pUb
250
150
100
70
50
40
35
25
20

Supplement: Supplementary file 6 — Source data Fig. 2 [file 44321_2025_323_MOESM6_ESM.zip › Figure 2/2D-E/WB.pptx]

## Slide 1
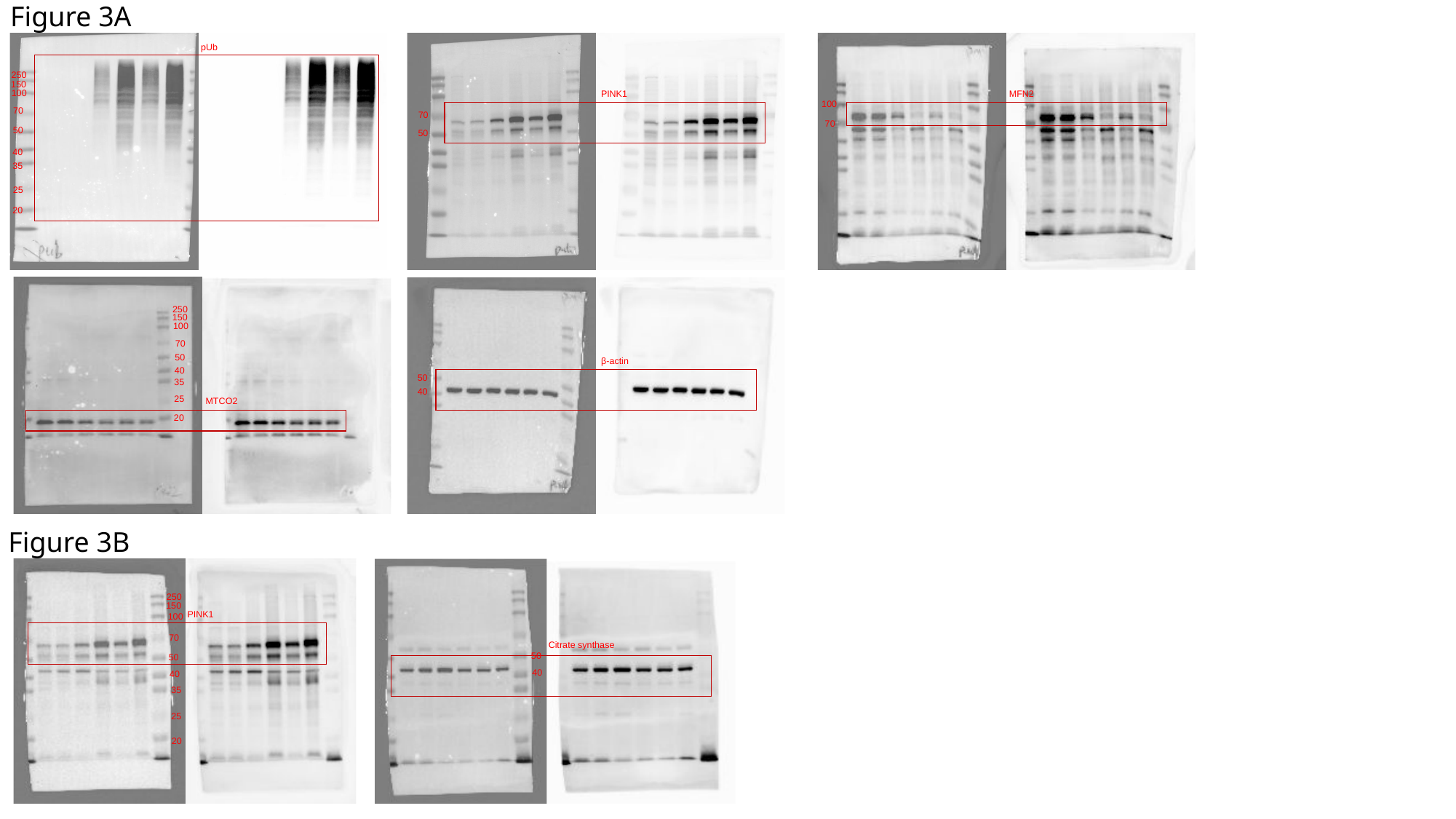

Figure 3A
pUb
250
150
100
PINK1
MFN2
100
70
70
70
50
50
40
35
25
20
250
150
100
70
50
β-actin
40
50
35
40
25
MTCO2
20
Figure 3B
250
150
PINK1
100
70
Citrate synthase
50
50
40
40
35
25
20

Supplement: Supplementary file 7 — Source data Fig. 3 [file 44321_2025_323_MOESM7_ESM.zip › Figure 3/3A-B/WB.pptx]

## Slide 1
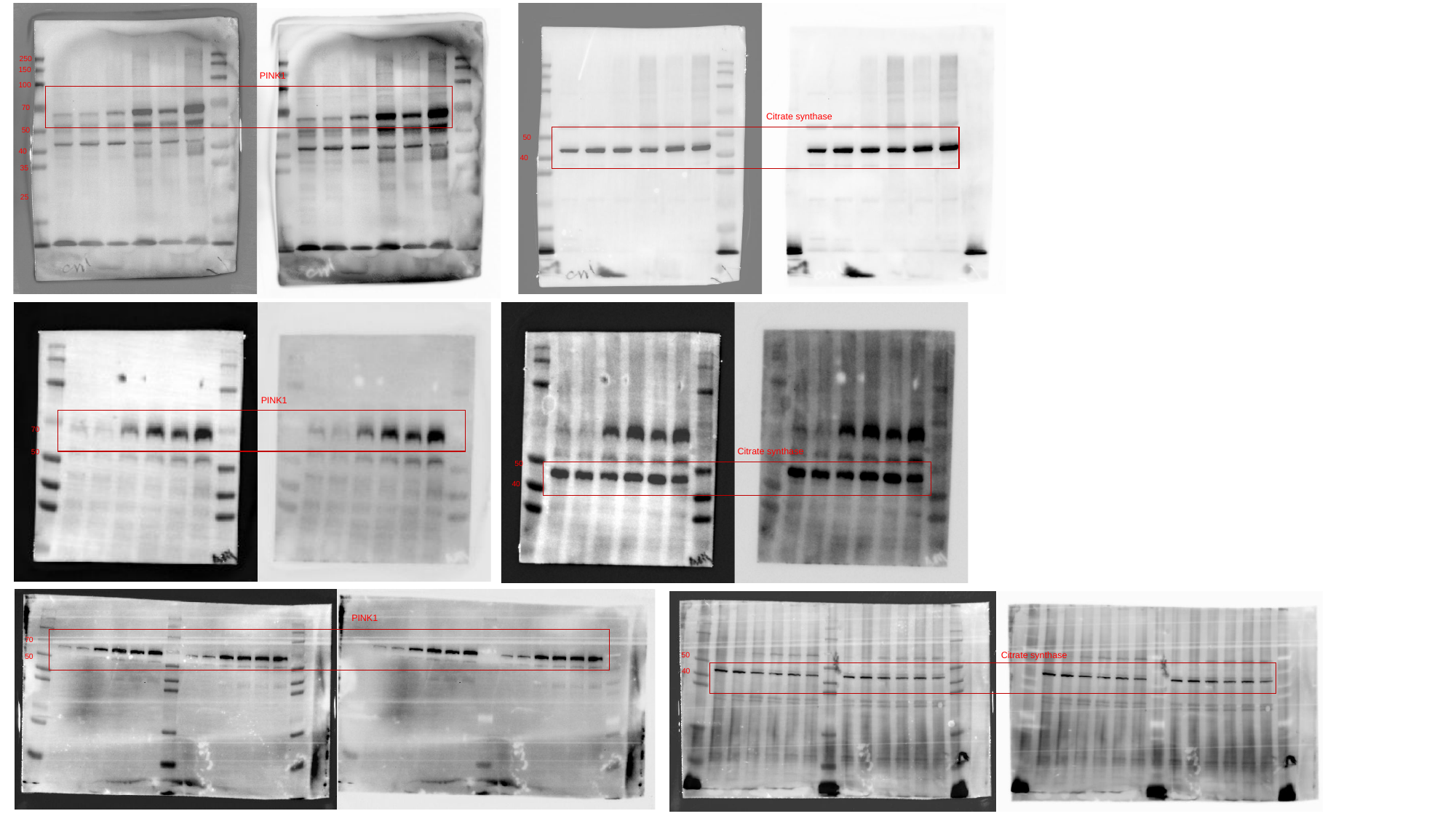

250
150
PINK1
100
70
Citrate synthase
50
50
40
40
35
25
PINK1
70
Citrate synthase
50
50
40
PINK1
70
Citrate synthase
50
50
40

Supplement: Supplementary file 7 — Source data Fig. 3 [file 44321_2025_323_MOESM7_ESM.zip › Figure 3/3C/biological repeat WB for analysis.pptx]

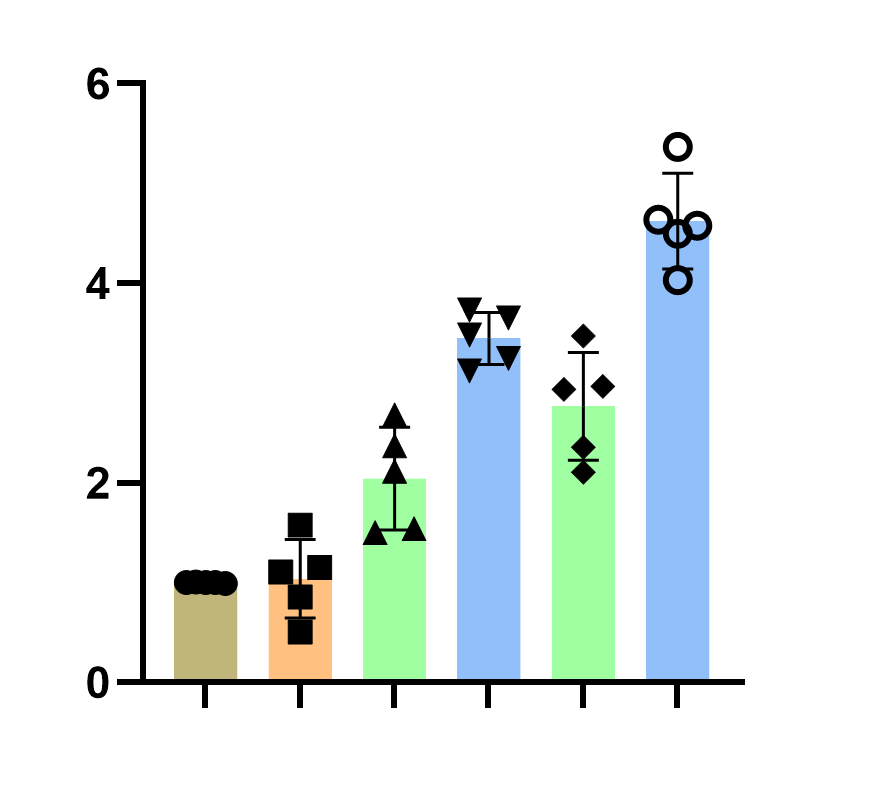

Supplement: Supplementary file 7 — Source data Fig. 3 [file 44321_2025_323_MOESM7_ESM.zip › Figure 3/3C/PINK1 expression.tif]

## Slide 1
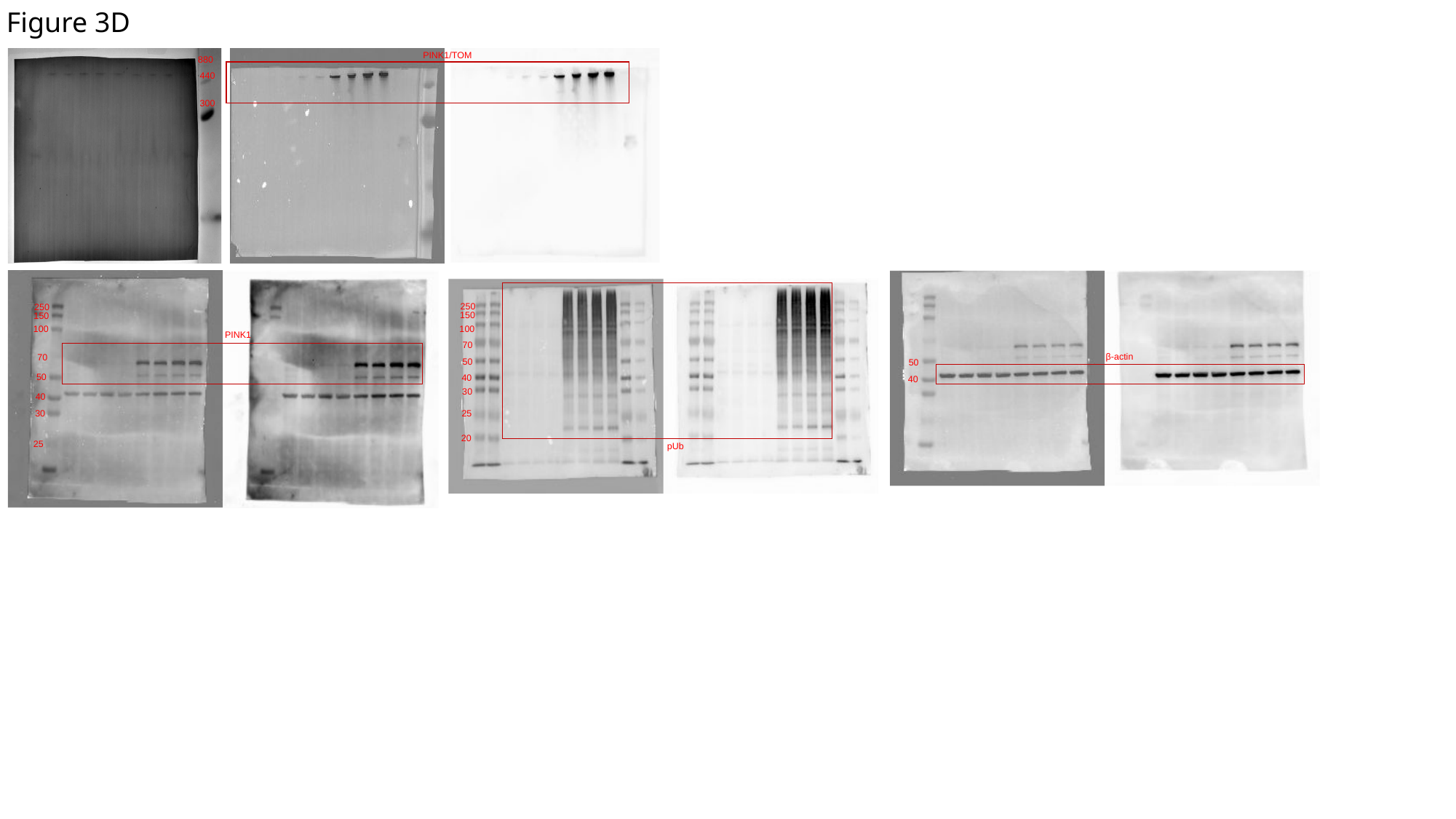

Figure 3D
PINK1/TOM
880
440
300
250
250
150
150
100
100
PINK1
70
β-actin
70
50
50
50
40
40
30
40
25
30
20
25
pUb

Supplement: Supplementary file 7 — Source data Fig. 3 [file 44321_2025_323_MOESM7_ESM.zip › Figure 3/3D/WB.pptx]

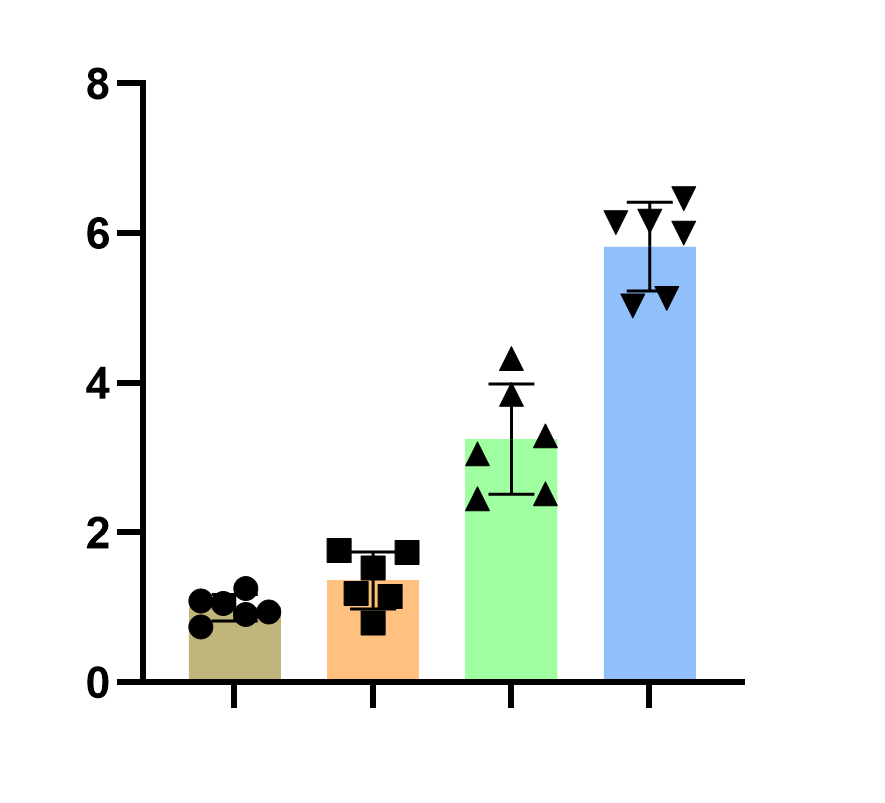

Supplement: Supplementary file 7 — Source data Fig. 3 [file 44321_2025_323_MOESM7_ESM.zip › Figure 3/3E/BN-PAGE.tif]

## Slide 1
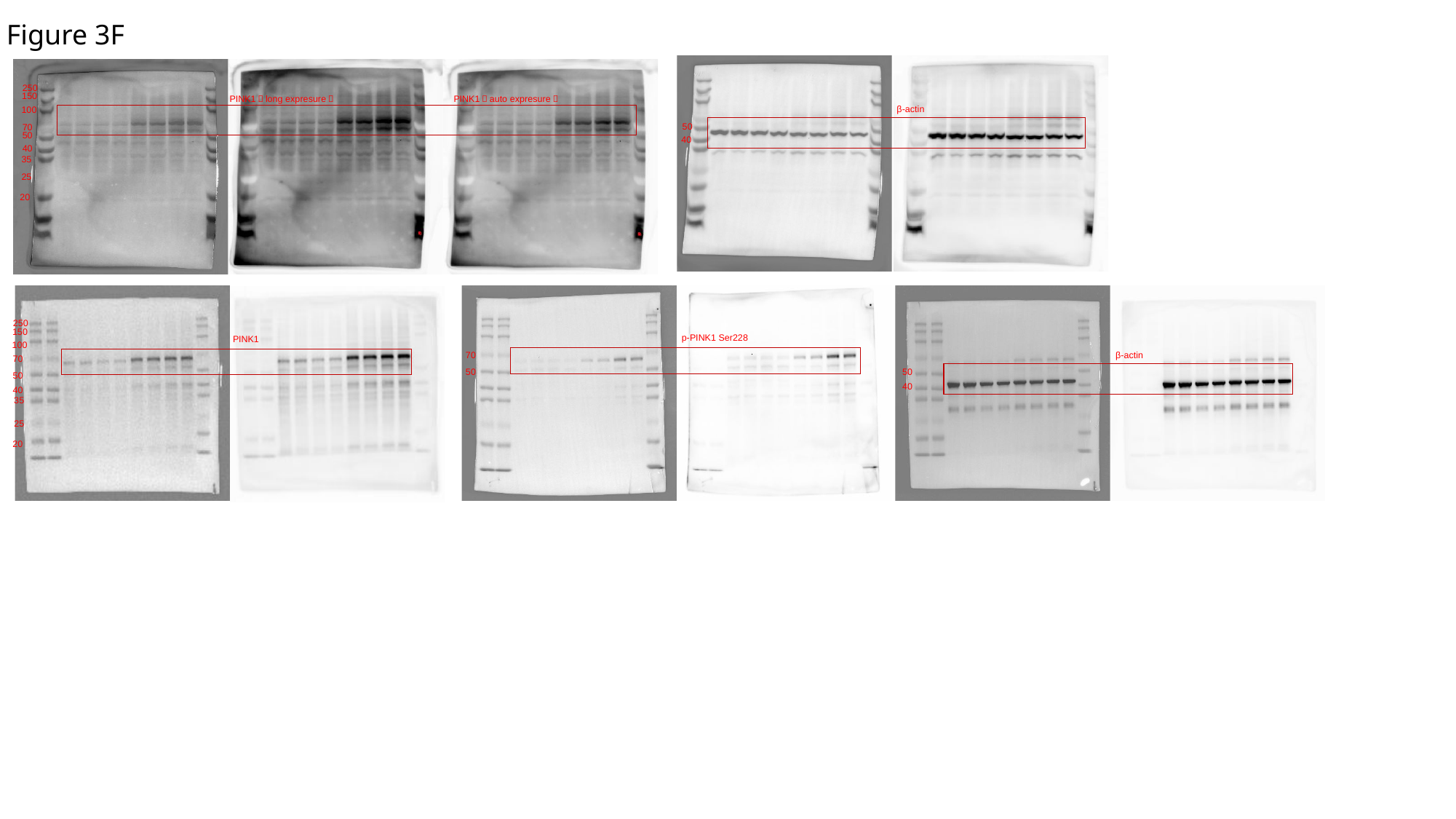

Figure 3F
250
150
PINK1（long expresure）
PINK1（auto expresure）
β-actin
100
50
70
50
40
40
35
25
20
250
150
p-PINK1 Ser228
PINK1
100
70
β-actin
70
50
50
50
40
40
35
25
20

Supplement: Supplementary file 7 — Source data Fig. 3 [file 44321_2025_323_MOESM7_ESM.zip › Figure 3/3F/WB.pptx]

## Slide 1
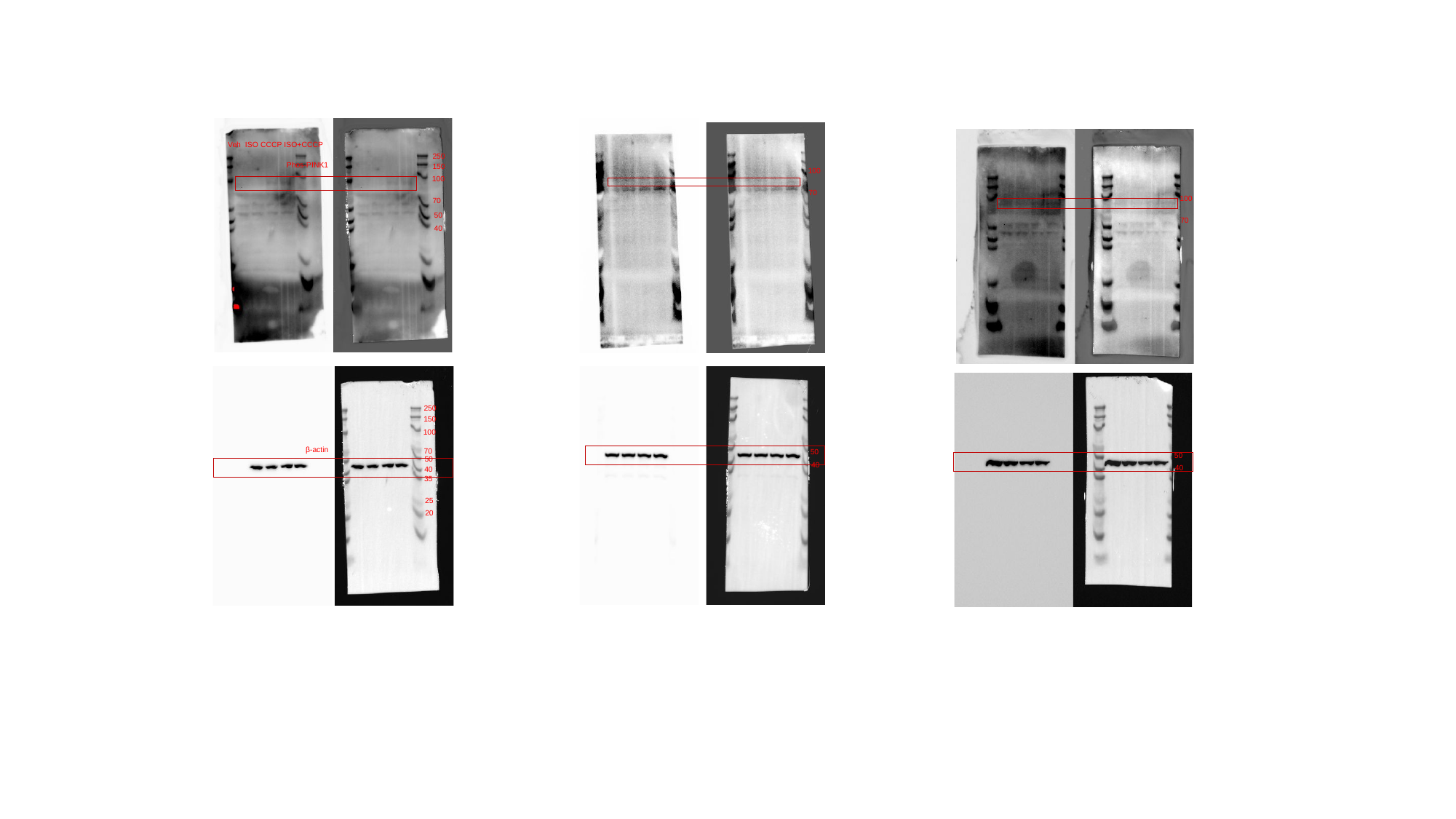

100
70
50
40
Veh ISO CCCP ISO+CCCP
250
Phos-PINK1
150
100
70
50
40
250
150
100
β-actin
70
50
40
35
25
20
100
70
50
40

Supplement: Supplementary file 7 — Source data Fig. 3 [file 44321_2025_323_MOESM7_ESM.zip › Figure 3/3G/biological repeat WB for analysis.pptx]

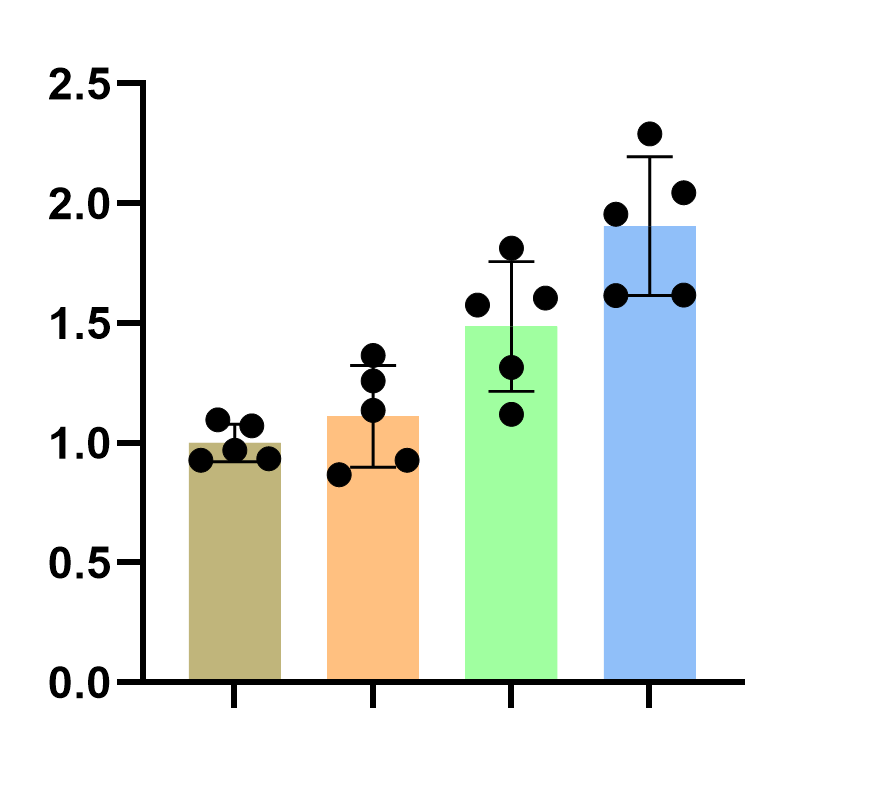

Supplement: Supplementary file 7 — Source data Fig. 3 [file 44321_2025_323_MOESM7_ESM.zip › Figure 3/3G/PHOS-PINK1.tif]

## Slide 1
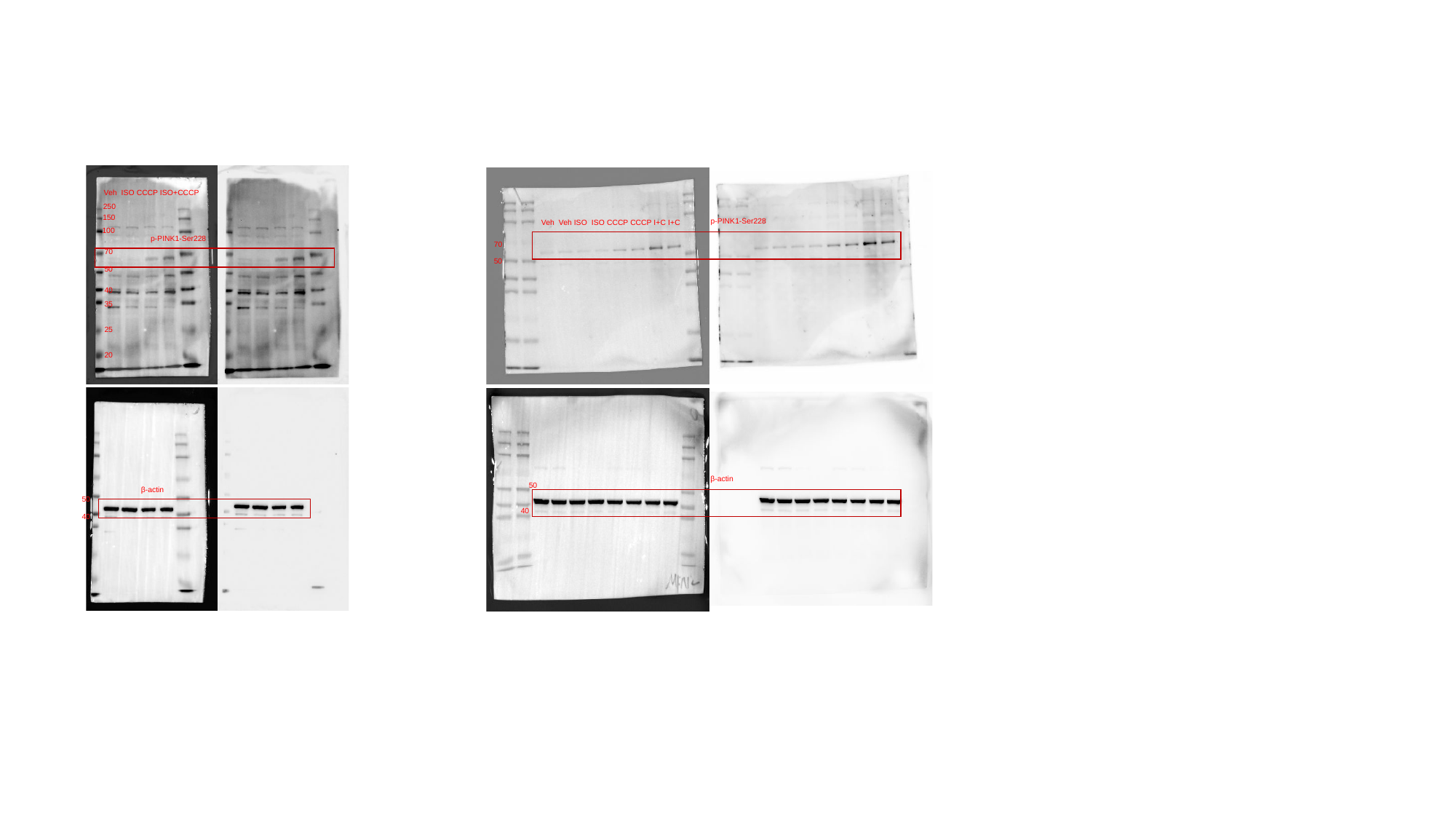

Veh ISO CCCP ISO+CCCP
250
150
100
p-PINK1-Ser228
70
50
40
35
25
20
β-actin
50
40
p-PINK1-Ser228
Veh Veh ISO ISO CCCP CCCP I+C I+C
70
50
β-actin
50
40

Supplement: Supplementary file 7 — Source data Fig. 3 [file 44321_2025_323_MOESM7_ESM.zip › Figure 3/3H/biological repeat WB for analysis.pptx]

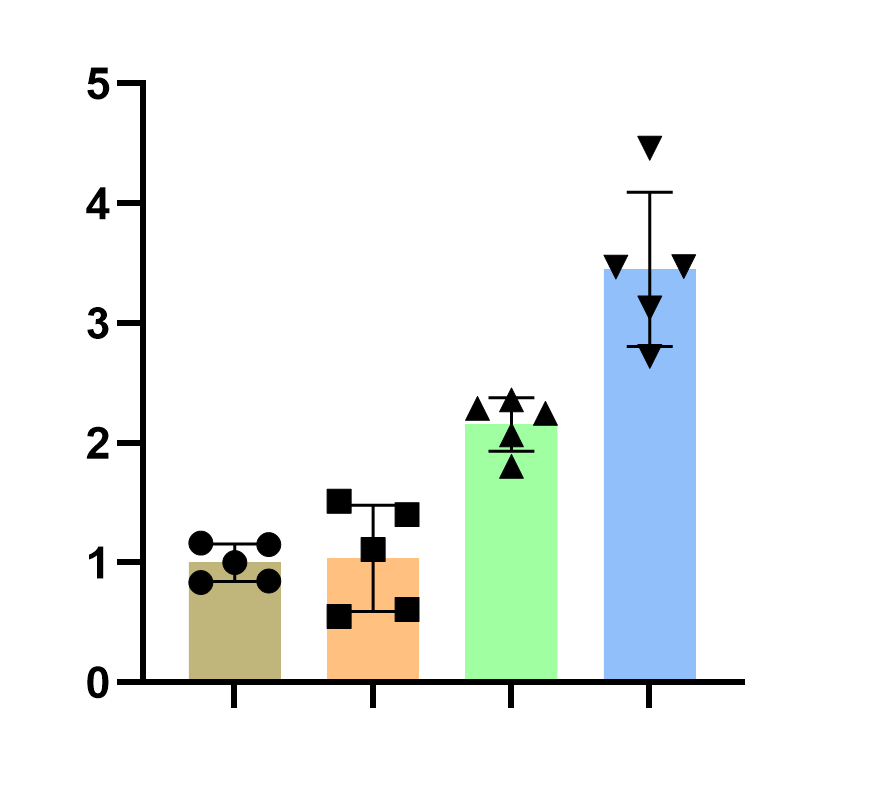

Supplement: Supplementary file 7 — Source data Fig. 3 [file 44321_2025_323_MOESM7_ESM.zip › Figure 3/3H/P-PINK1-228.tif]

## Slide 1
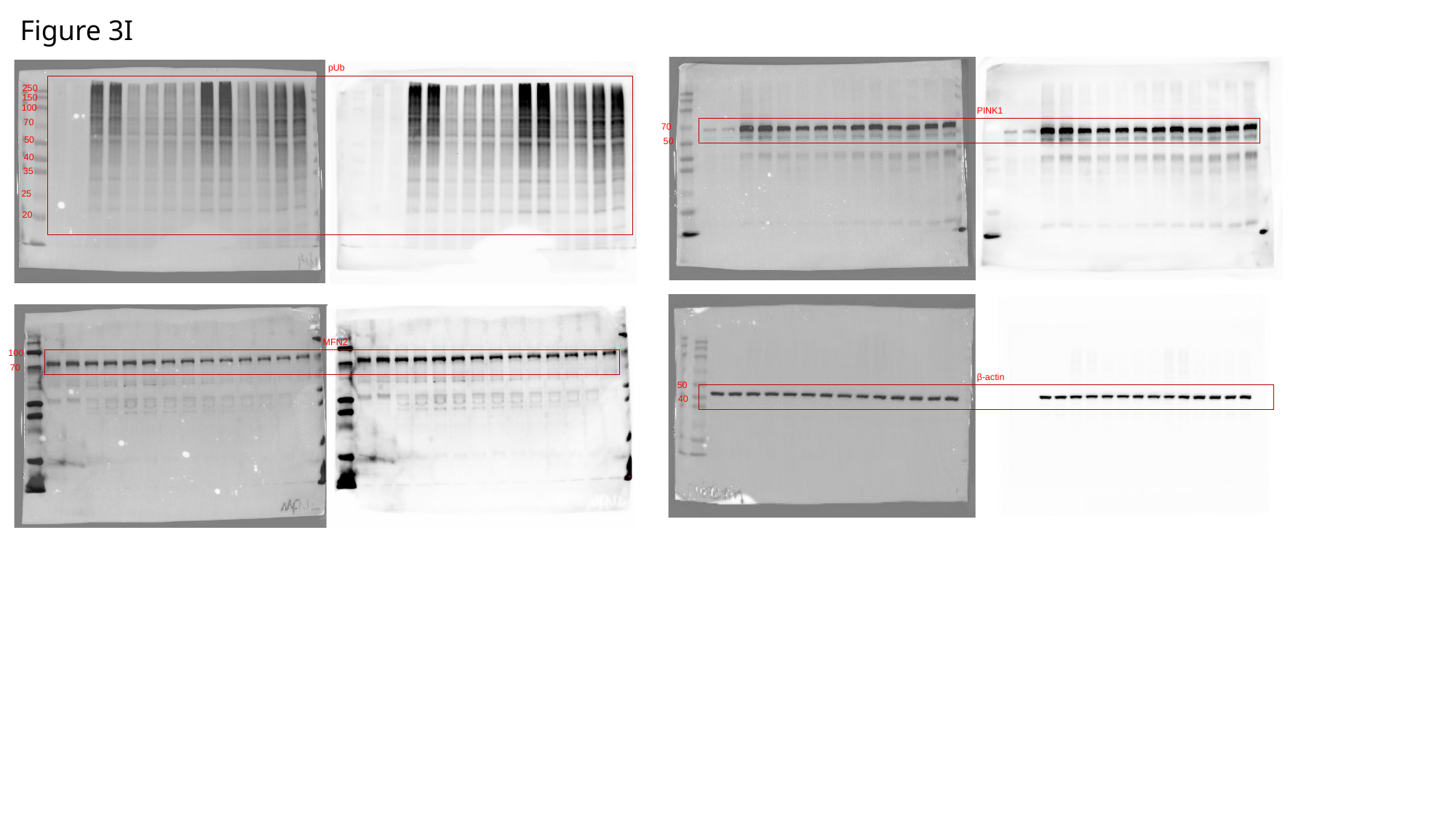

Figure 3I
pUb
250
150
100
70
50
40
35
25
20
PINK1
70
50
β-actin
50
40
MFN2
100
70

Supplement: Supplementary file 7 — Source data Fig. 3 [file 44321_2025_323_MOESM7_ESM.zip › Figure 3/3I/WB.pptx]

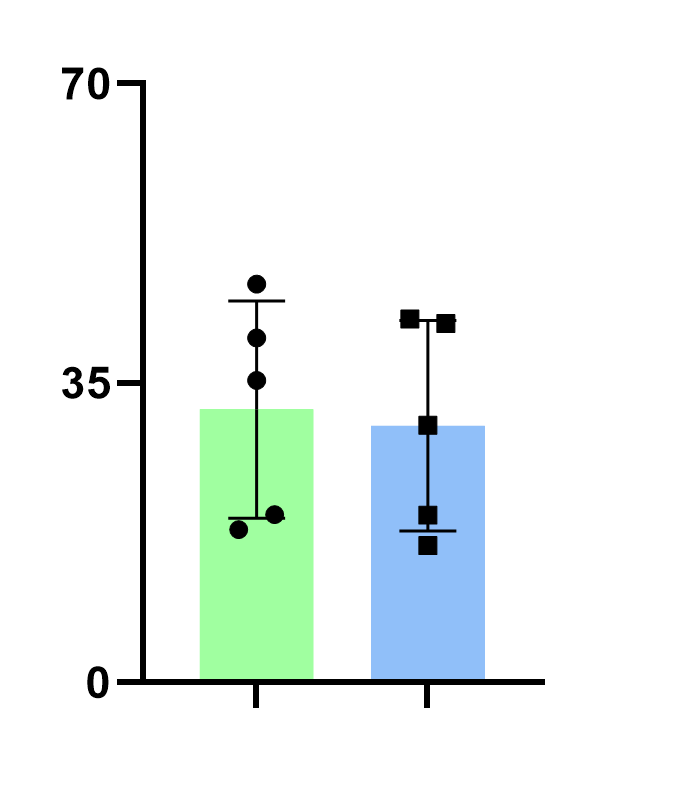

Supplement: Supplementary file 7 — Source data Fig. 3 [file 44321_2025_323_MOESM7_ESM.zip › Figure 3/3J-K/maximal respiration.tif]

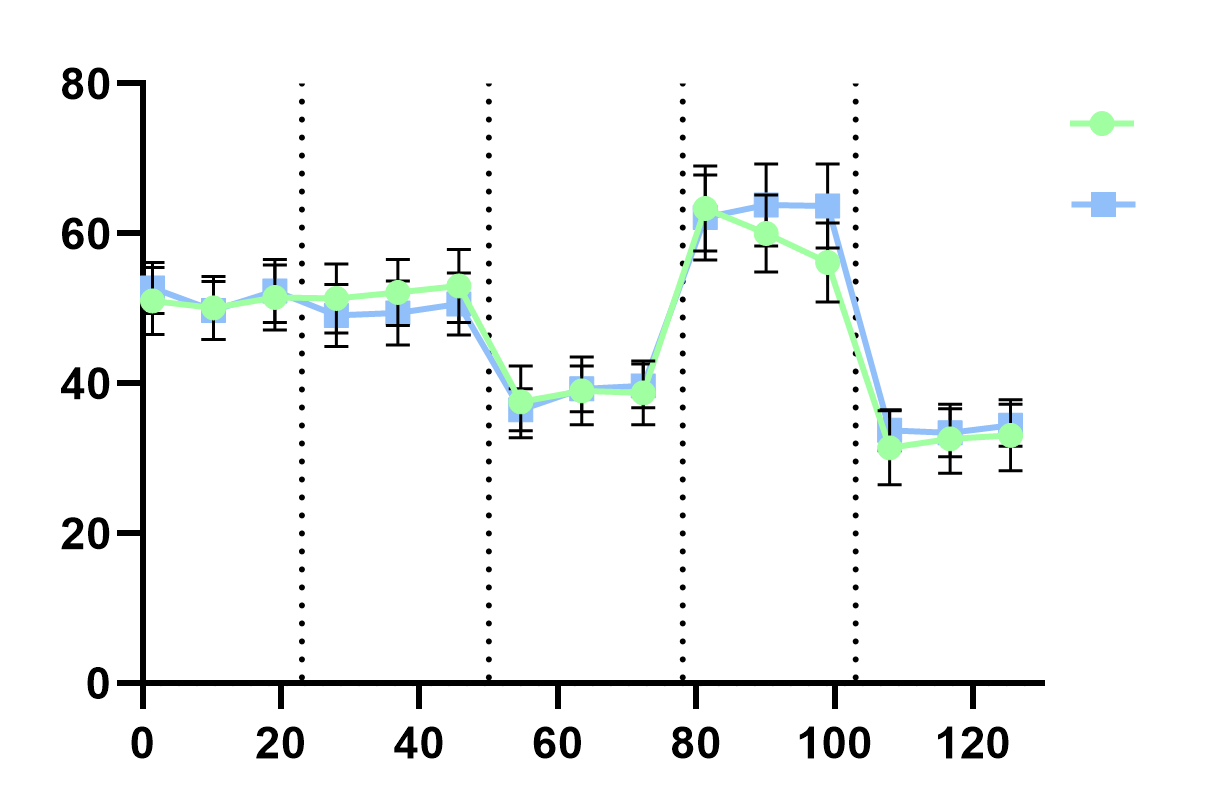

Supplement: Supplementary file 7 — Source data Fig. 3 [file 44321_2025_323_MOESM7_ESM.zip › Figure 3/3J-K/seahorse.tif]

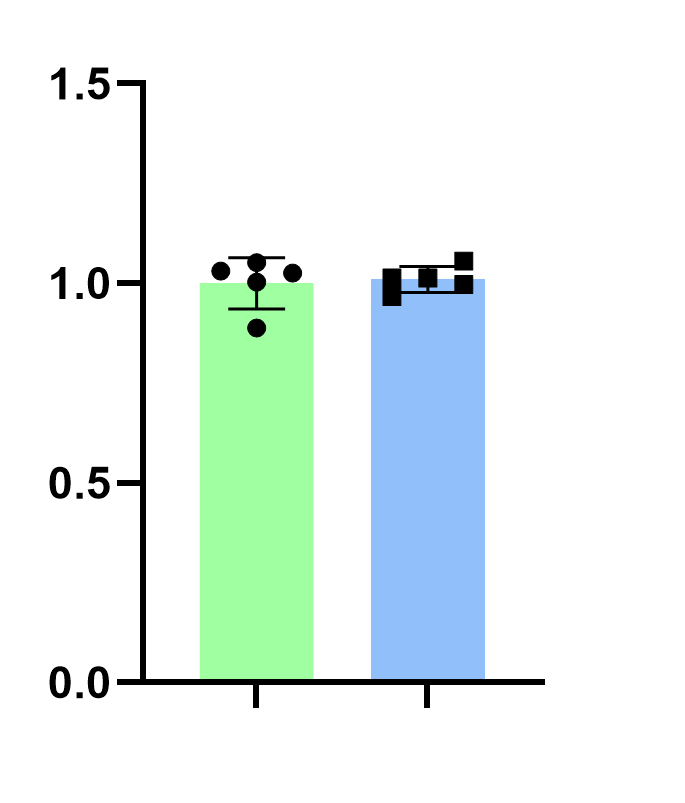

Supplement: Supplementary file 7 — Source data Fig. 3 [file 44321_2025_323_MOESM7_ESM.zip › Figure 3/3L/MTDNA COPY.tif]

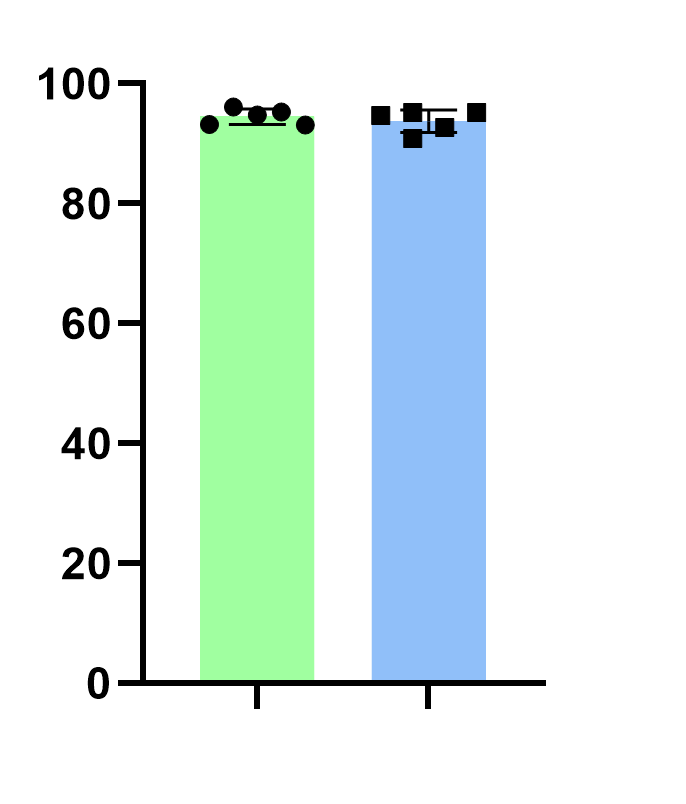

Supplement: Supplementary file 7 — Source data Fig. 3 [file 44321_2025_323_MOESM7_ESM.zip › Figure 3/3M/MMP.tif]

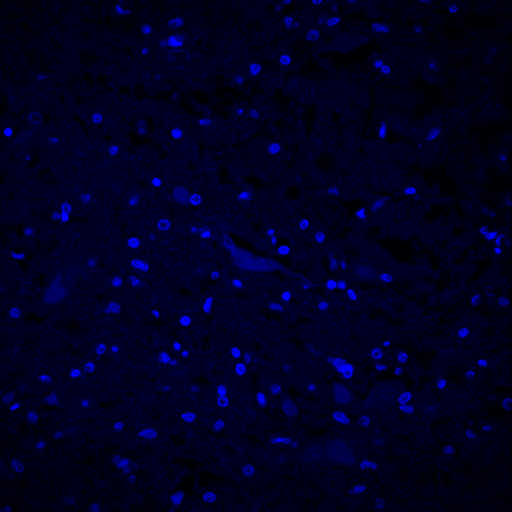

Supplement: Supplementary file 8 — Source data Fig. 4 [file 44321_2025_323_MOESM8_ESM.zip › Figure 4/4C/ALS DAPI.tif]

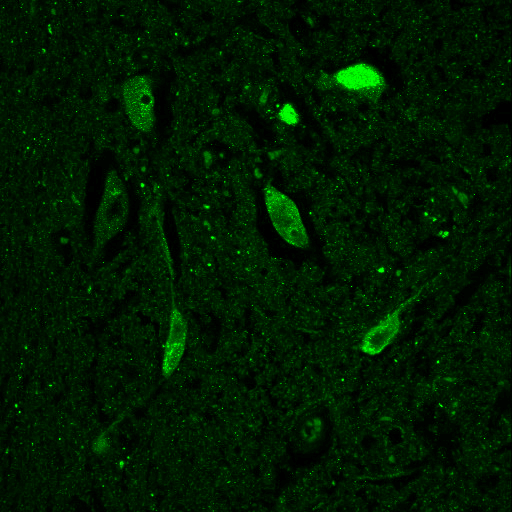

Supplement: Supplementary file 8 — Source data Fig. 4 [file 44321_2025_323_MOESM8_ESM.zip › Figure 4/4C/ALS LAMP2.tif]

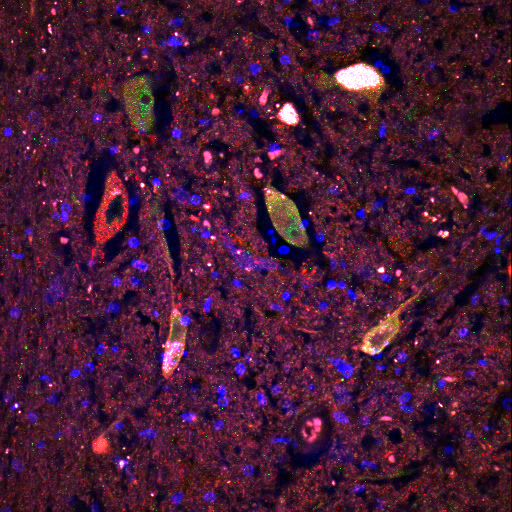

Supplement: Supplementary file 8 — Source data Fig. 4 [file 44321_2025_323_MOESM8_ESM.zip › Figure 4/4C/ALS Merge.tif]

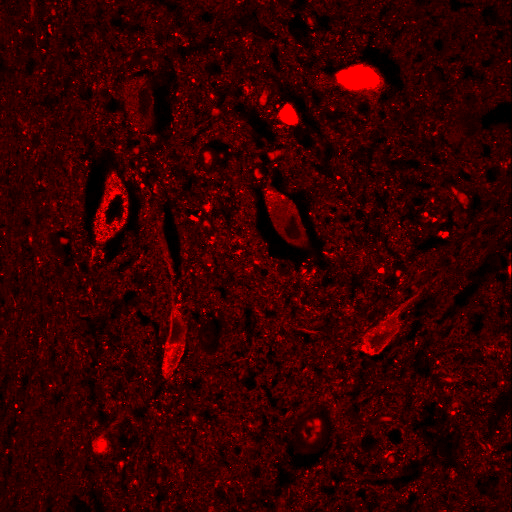

Supplement: Supplementary file 8 — Source data Fig. 4 [file 44321_2025_323_MOESM8_ESM.zip › Figure 4/4C/ALS MTCO2.tif]

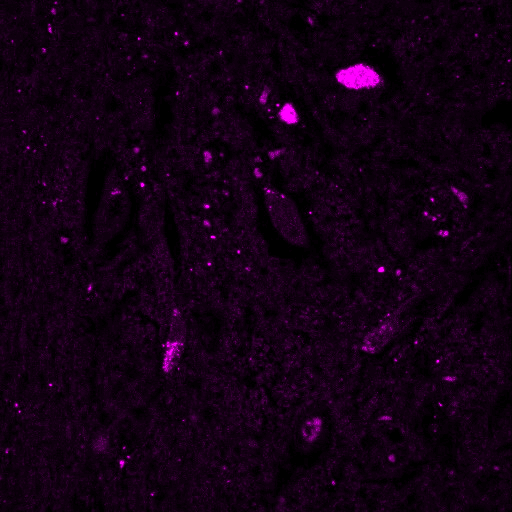

Supplement: Supplementary file 8 — Source data Fig. 4 [file 44321_2025_323_MOESM8_ESM.zip › Figure 4/4C/ALS NeuN.tif]

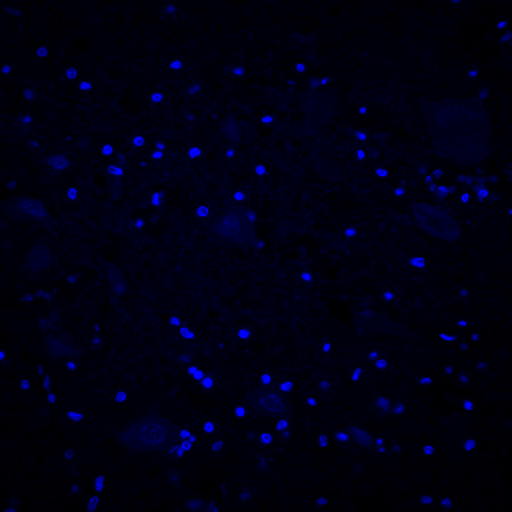

Supplement: Supplementary file 8 — Source data Fig. 4 [file 44321_2025_323_MOESM8_ESM.zip › Figure 4/4C/Health DAPI.tif]

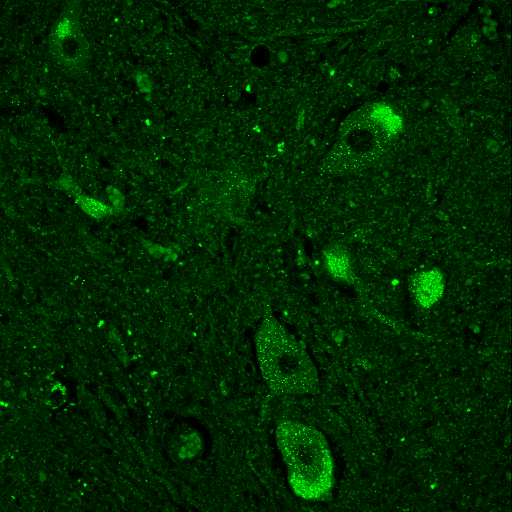

Supplement: Supplementary file 8 — Source data Fig. 4 [file 44321_2025_323_MOESM8_ESM.zip › Figure 4/4C/Health LAMP2.tif]

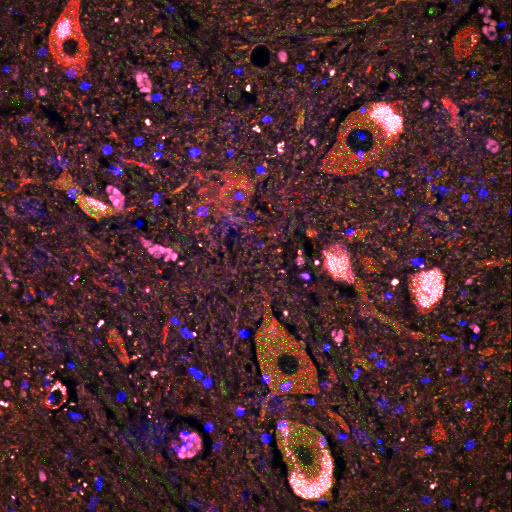

Supplement: Supplementary file 8 — Source data Fig. 4 [file 44321_2025_323_MOESM8_ESM.zip › Figure 4/4C/Health Merge.tif]

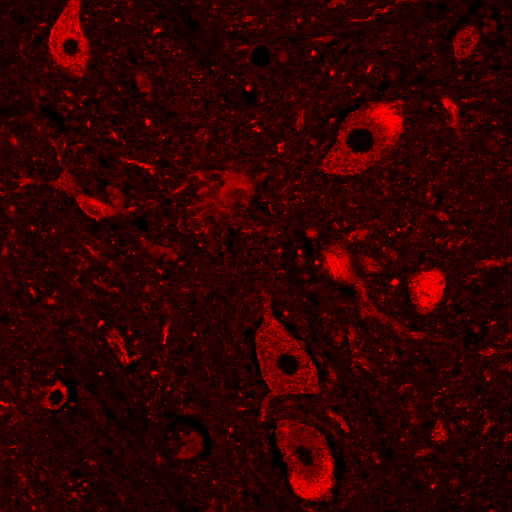

Supplement: Supplementary file 8 — Source data Fig. 4 [file 44321_2025_323_MOESM8_ESM.zip › Figure 4/4C/Health MTOC2.tif]

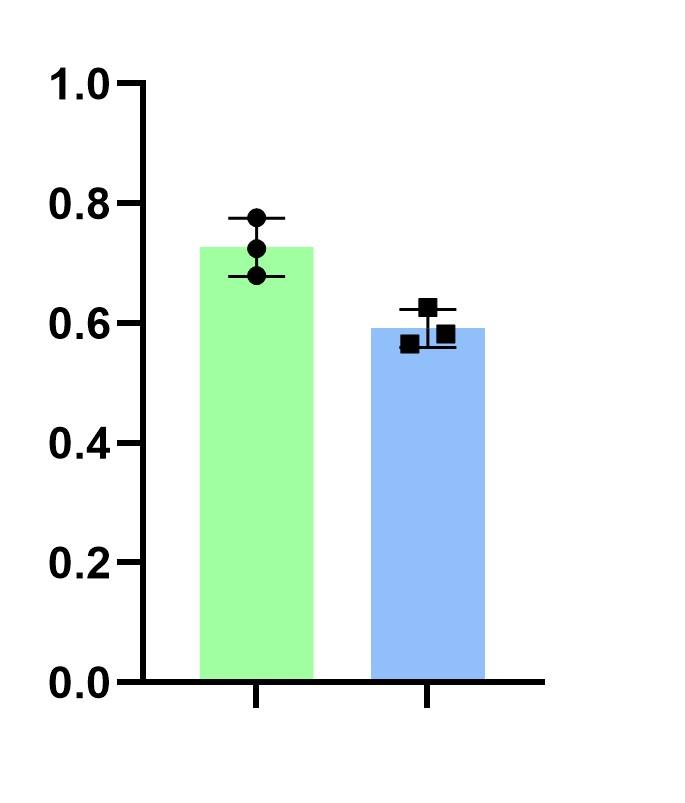

Supplement: Supplementary file 8 — Source data Fig. 4 [file 44321_2025_323_MOESM8_ESM.zip › Figure 4/4D/Pearson correlation coefficient.tif]

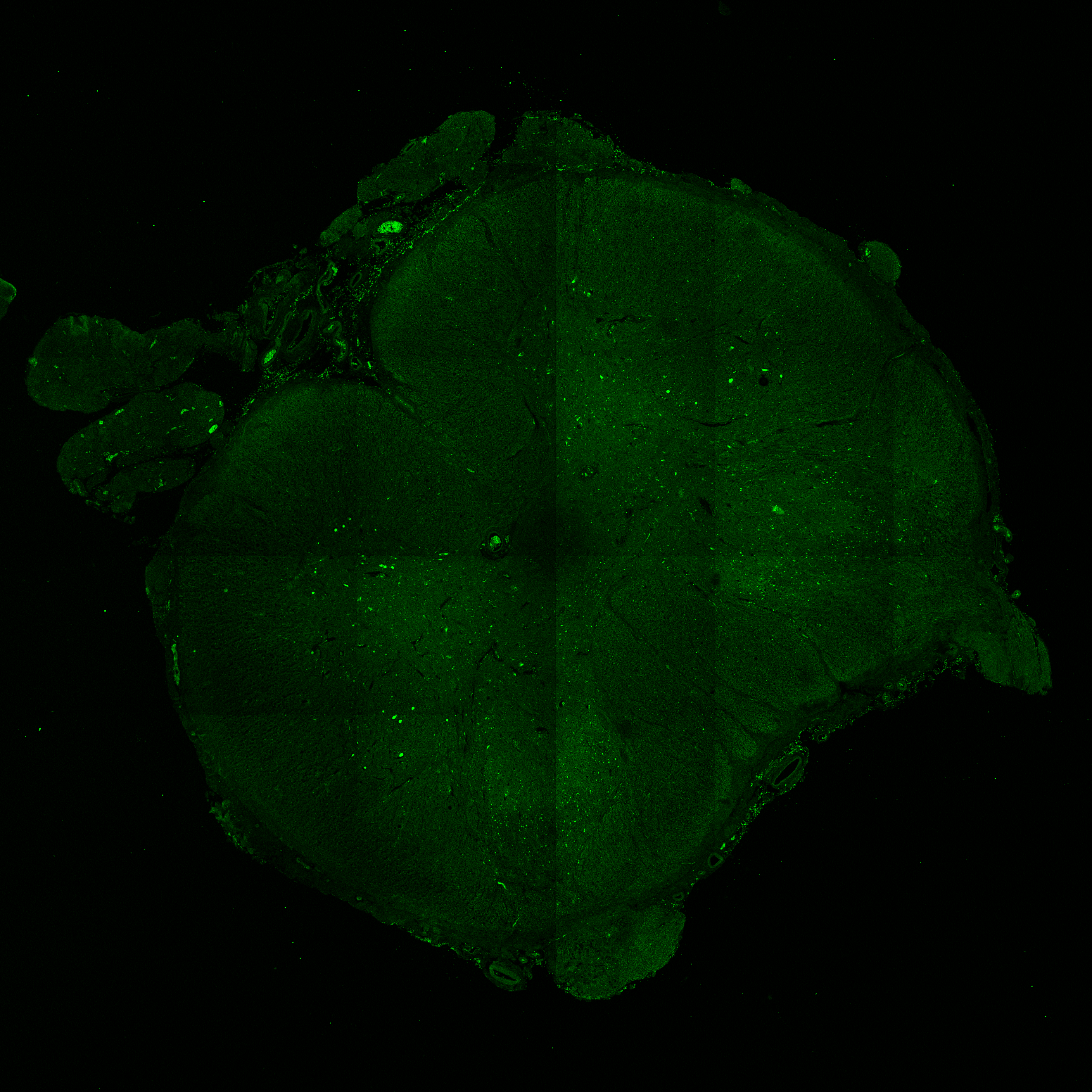

Supplement: Supplementary file 8 — Source data Fig. 4 [file 44321_2025_323_MOESM8_ESM.zip › Figure 4/4E/ALS3 pUbs65 NeuN NeuN.tif]

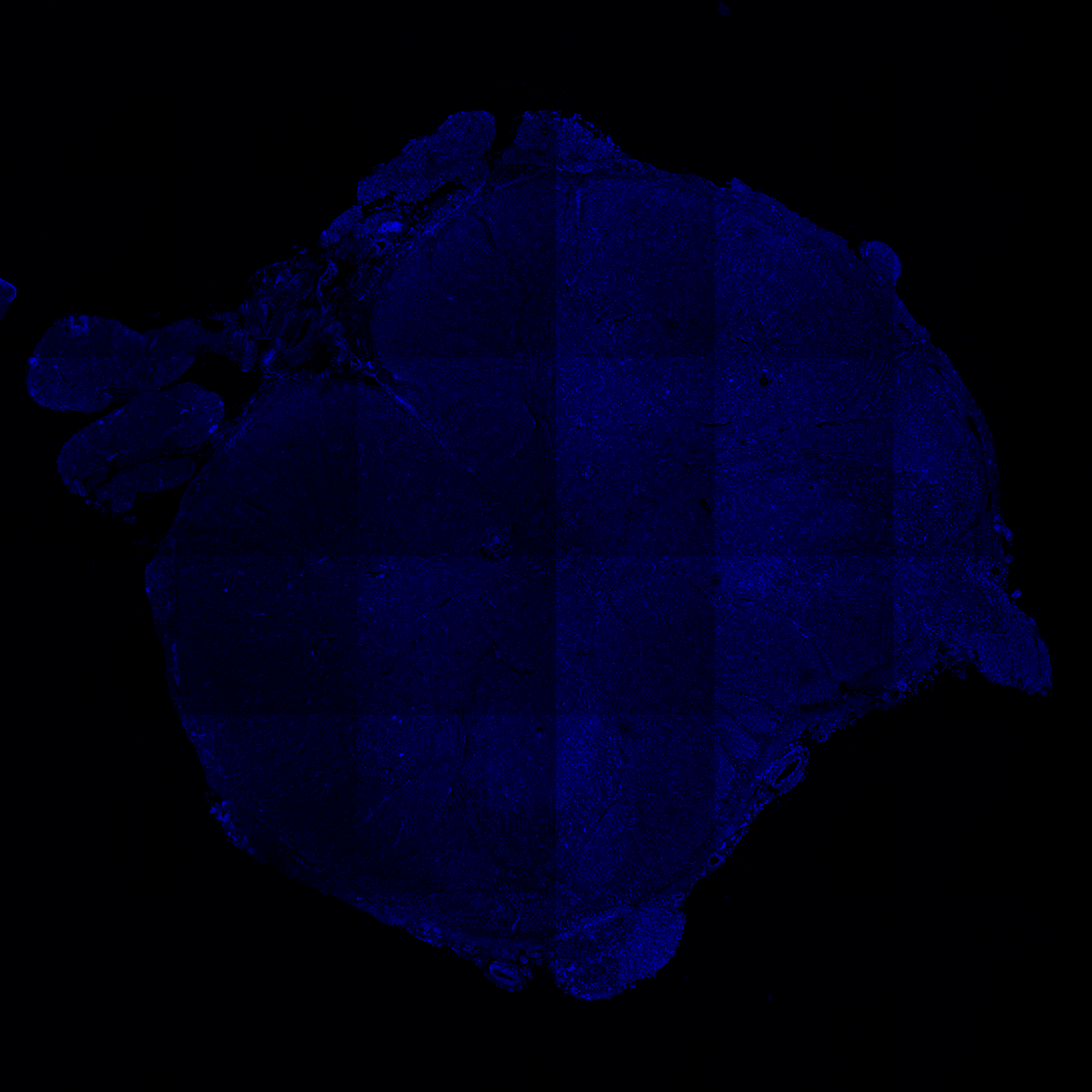

Supplement: Supplementary file 8 — Source data Fig. 4 [file 44321_2025_323_MOESM8_ESM.zip › Figure 4/4E/ALS3 pUbs65 NeuN DAPI.tif]

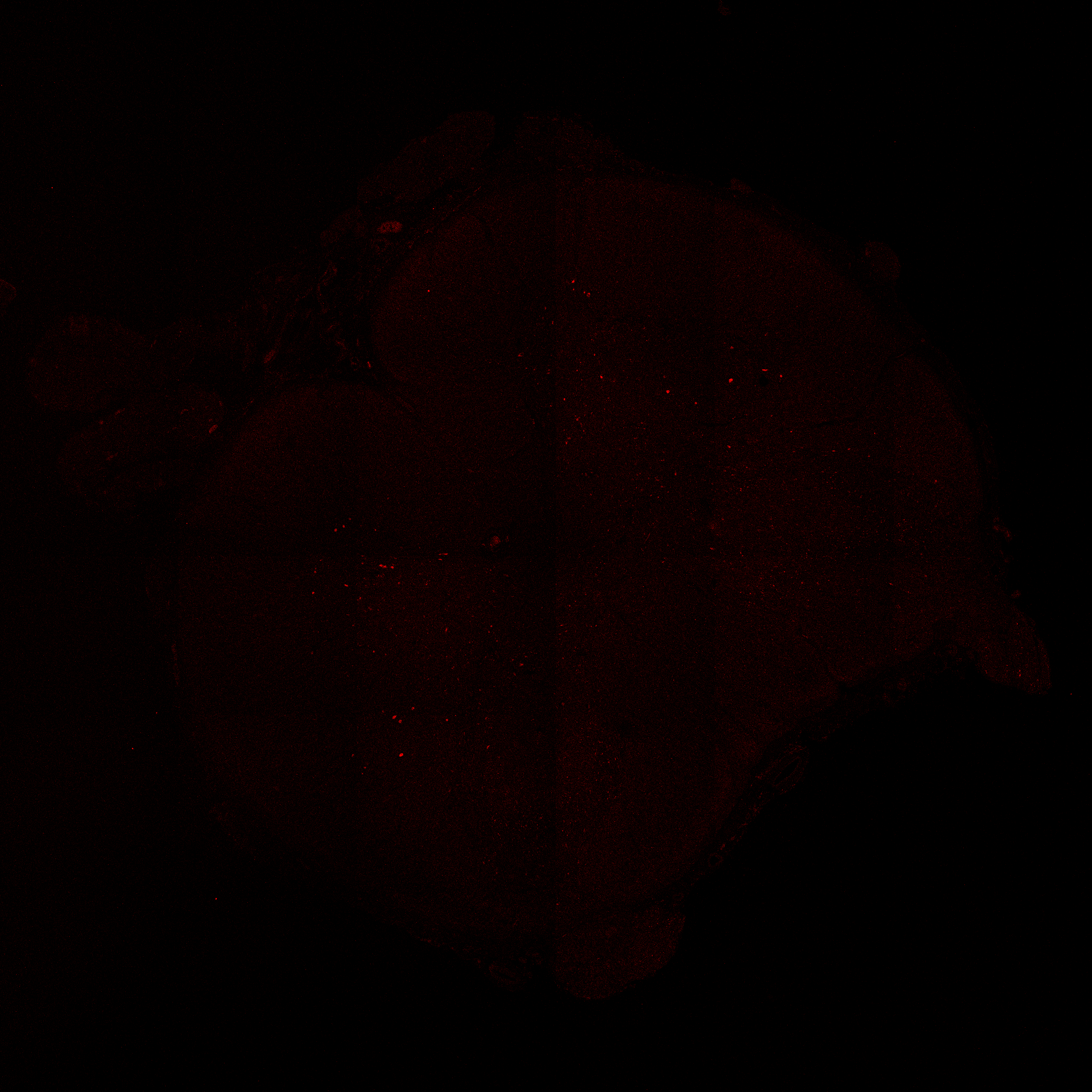

Supplement: Supplementary file 8 — Source data Fig. 4 [file 44321_2025_323_MOESM8_ESM.zip › Figure 4/4E/ALS3 pUbs65 NeuN pUbs65.tif]

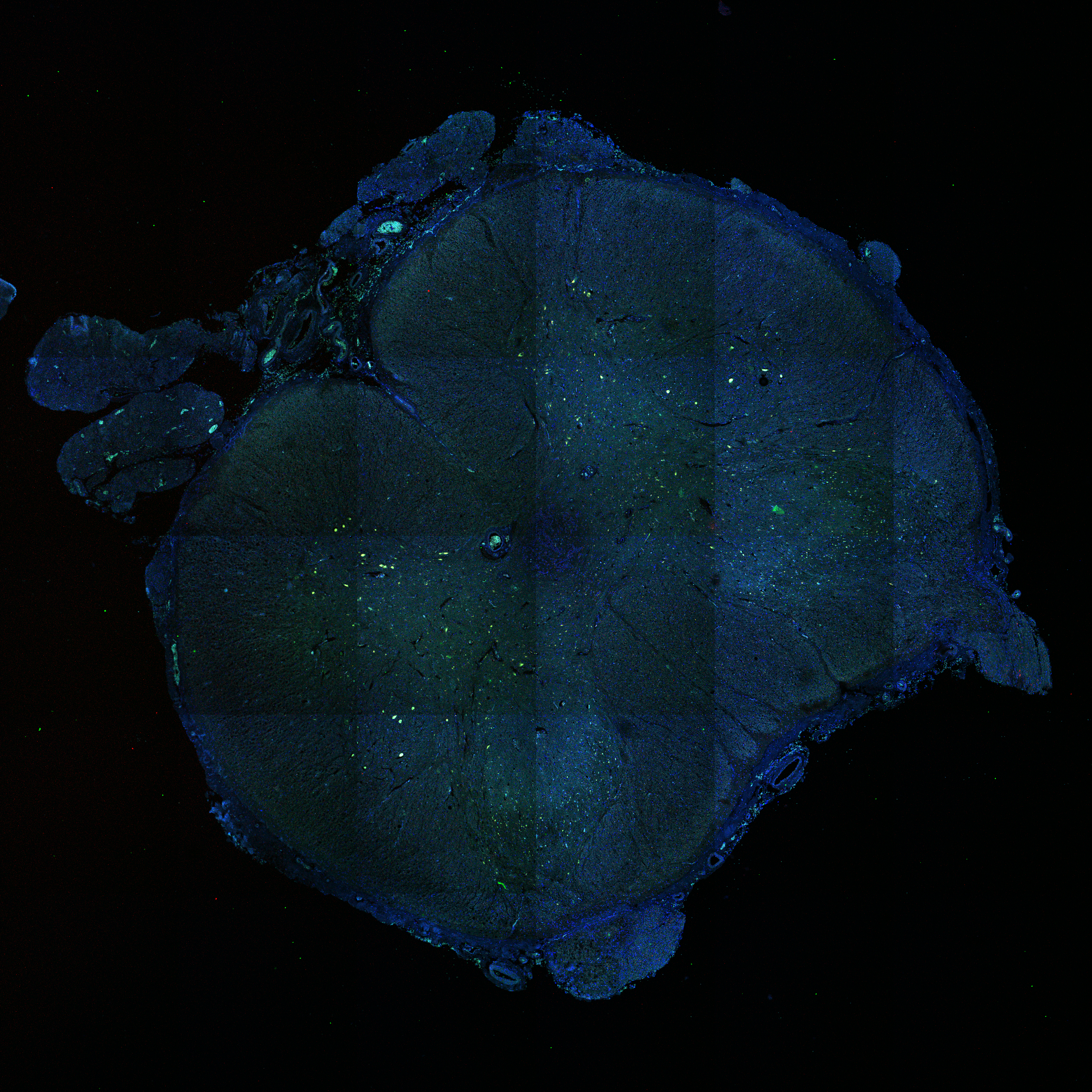

Supplement: Supplementary file 8 — Source data Fig. 4 [file 44321_2025_323_MOESM8_ESM.zip › Figure 4/4E/ALS3 pUbs65 NeuN.tif]

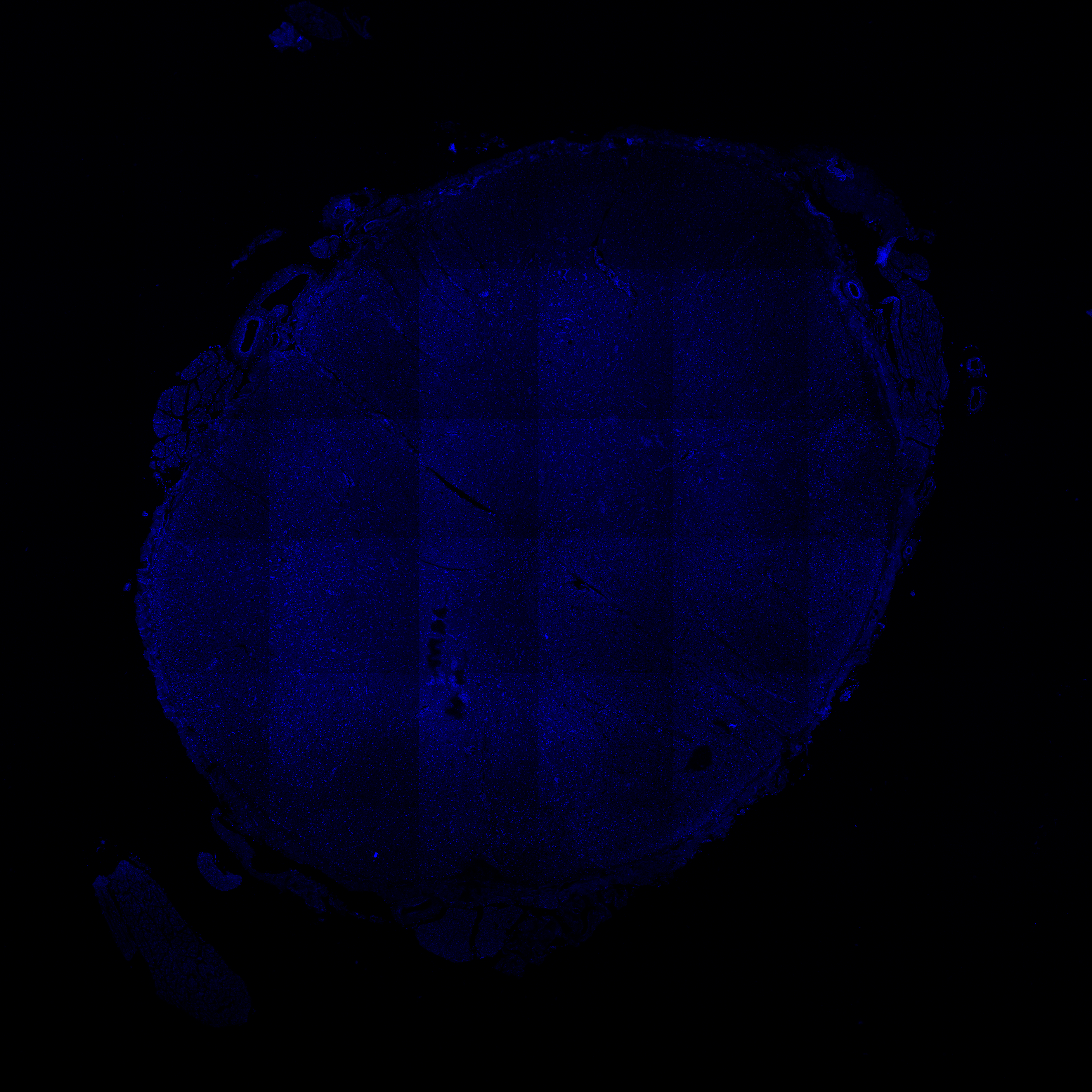

Supplement: Supplementary file 8 — Source data Fig. 4 [file 44321_2025_323_MOESM8_ESM.zip › Figure 4/4E/Ctl2 pUbs65 NeuN DAPI.tif]

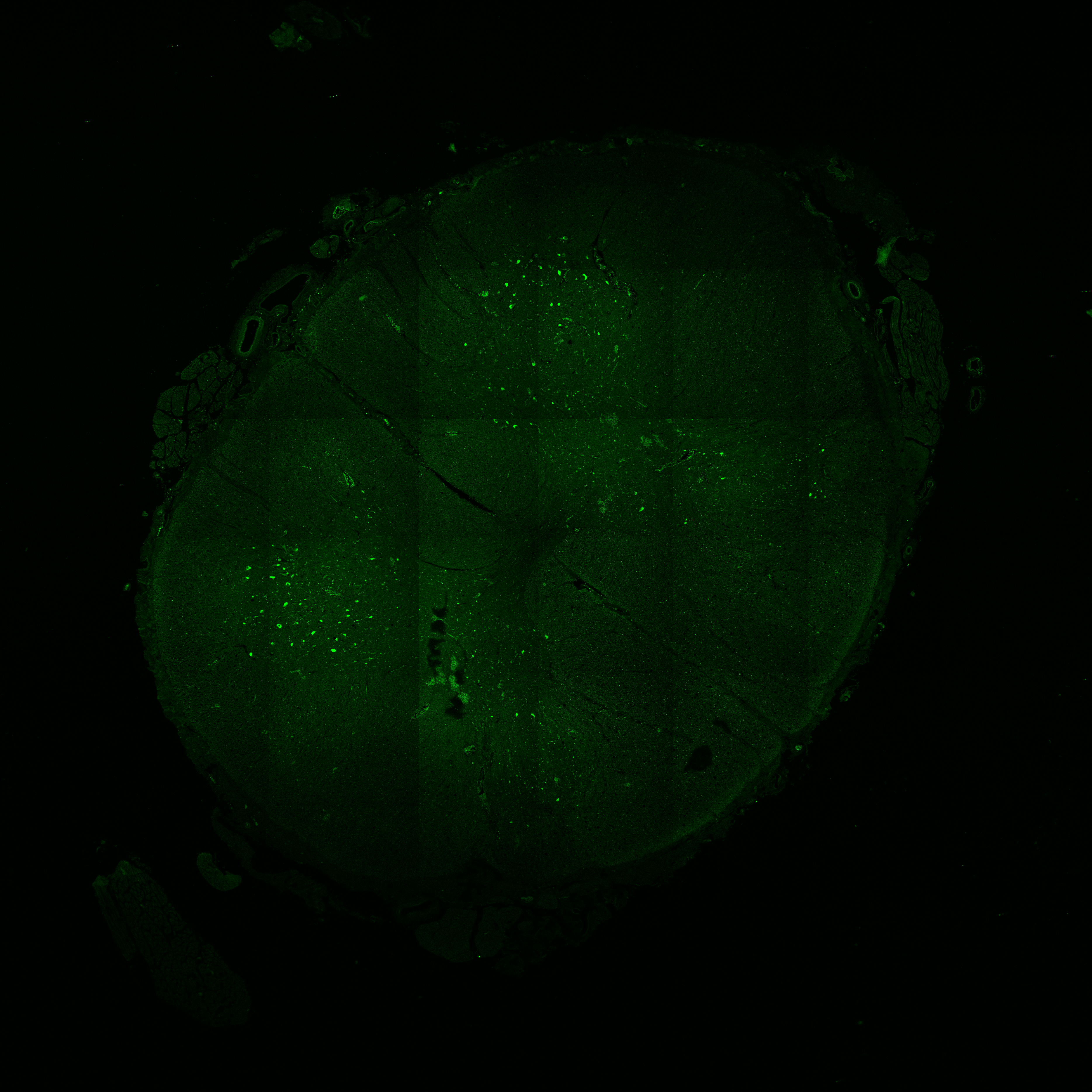

Supplement: Supplementary file 8 — Source data Fig. 4 [file 44321_2025_323_MOESM8_ESM.zip › Figure 4/4E/Ctl2 pUbs65 NeuN NeuN.tif]

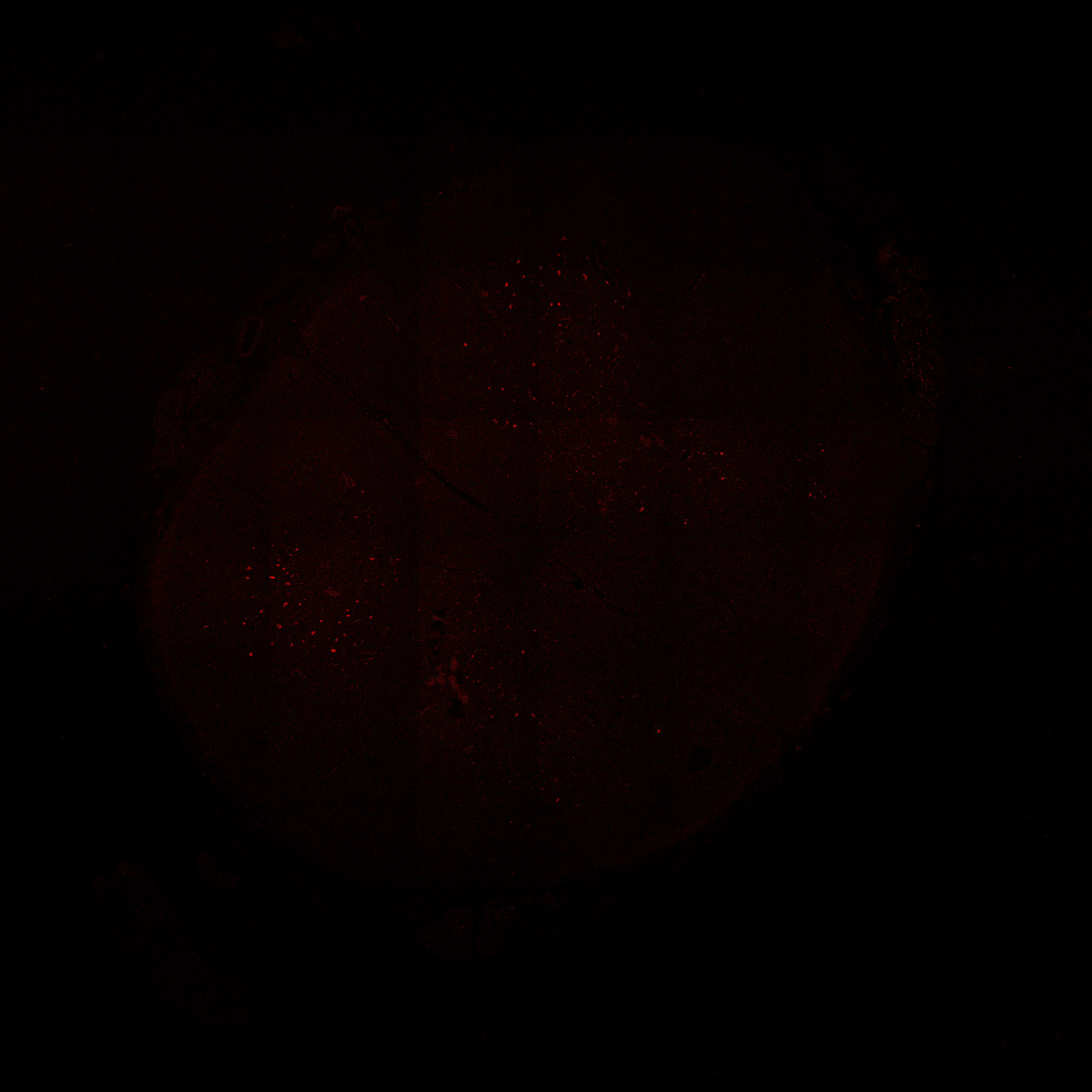

Supplement: Supplementary file 8 — Source data Fig. 4 [file 44321_2025_323_MOESM8_ESM.zip › Figure 4/4E/Ctl2 pUbs65 NeuN pUbs65.tif]

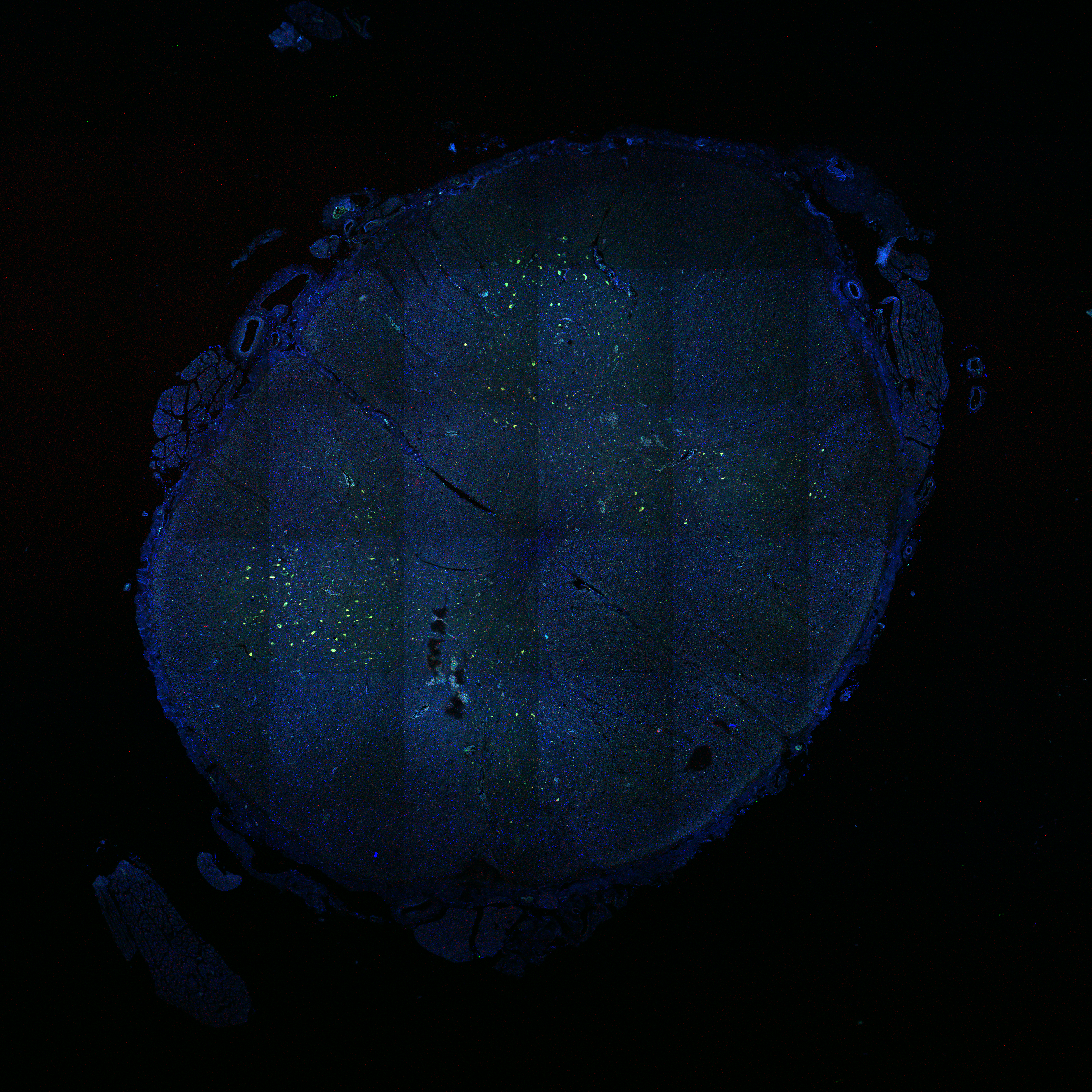

Supplement: Supplementary file 8 — Source data Fig. 4 [file 44321_2025_323_MOESM8_ESM.zip › Figure 4/4E/Ctl2 pUbs65 NeuN.tif]

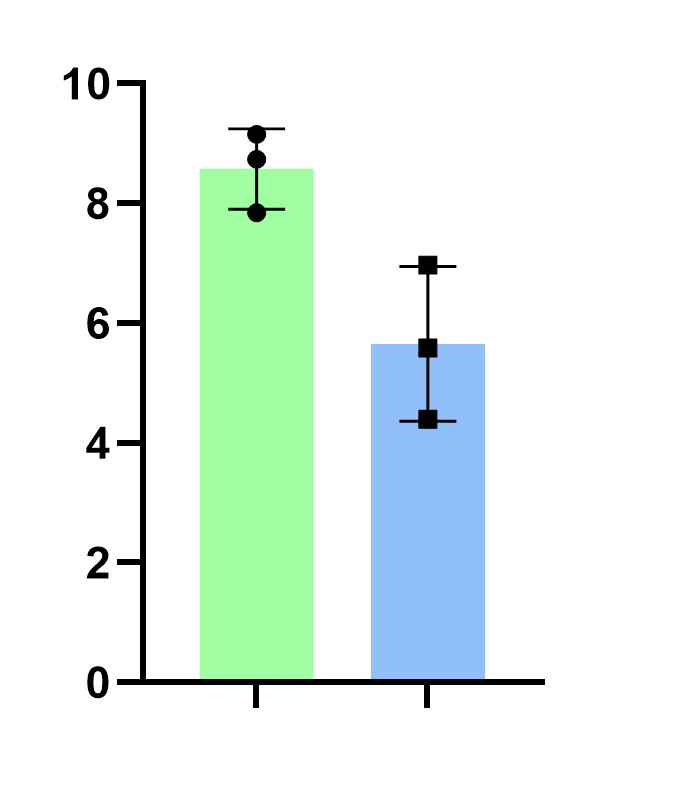

Supplement: Supplementary file 8 — Source data Fig. 4 [file 44321_2025_323_MOESM8_ESM.zip › Figure 4/4F/pUB.tif]

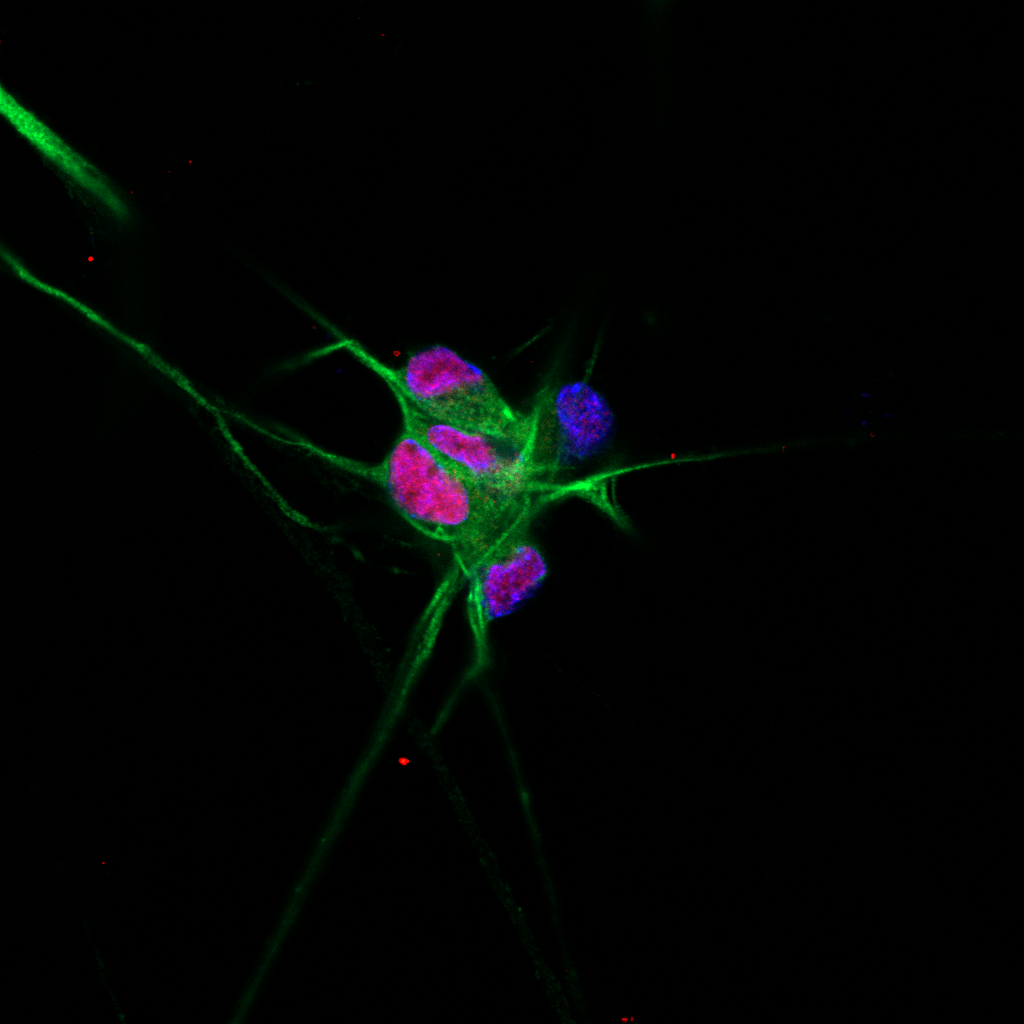

Supplement: Supplementary file 8 — Source data Fig. 4 [file 44321_2025_323_MOESM8_ESM.zip › Figure 4/4G/C9.tiff]

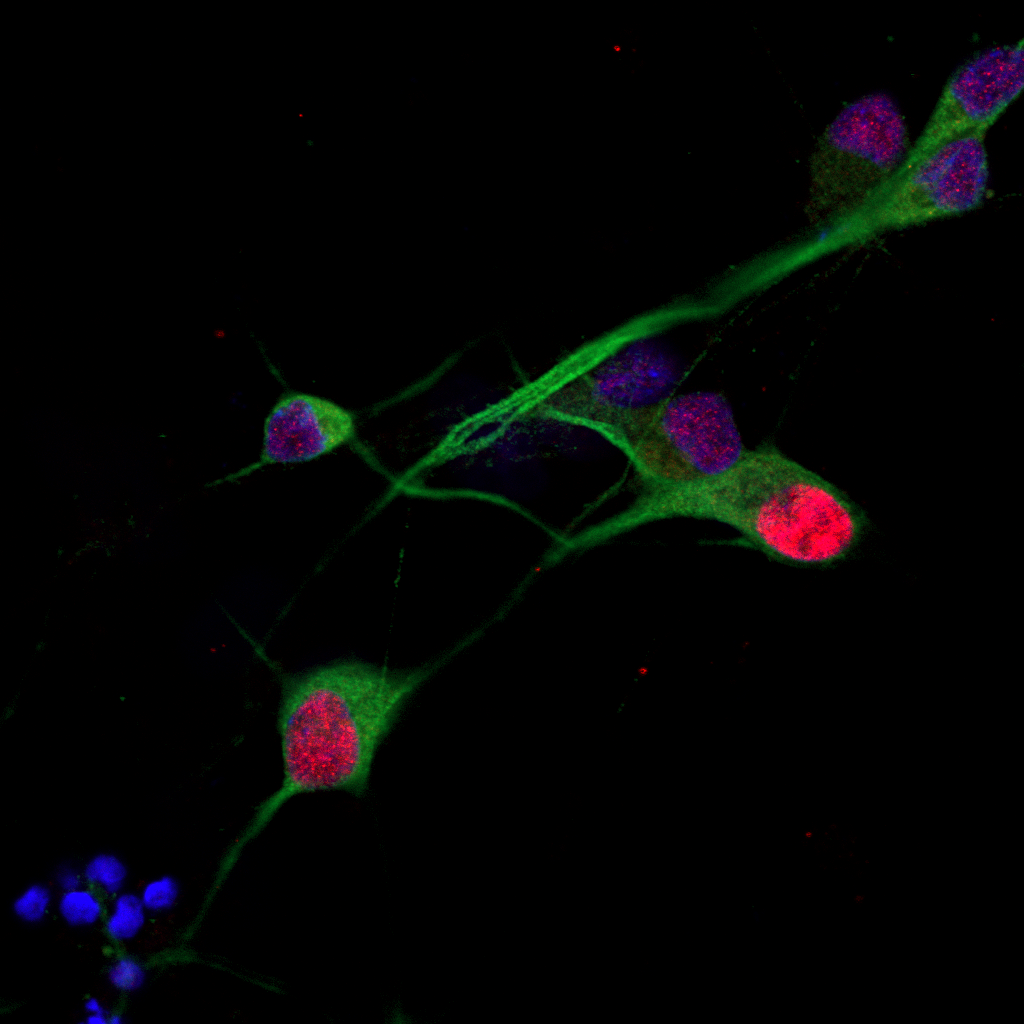

Supplement: Supplementary file 8 — Source data Fig. 4 [file 44321_2025_323_MOESM8_ESM.zip › Figure 4/4G/SOD1.tiff]

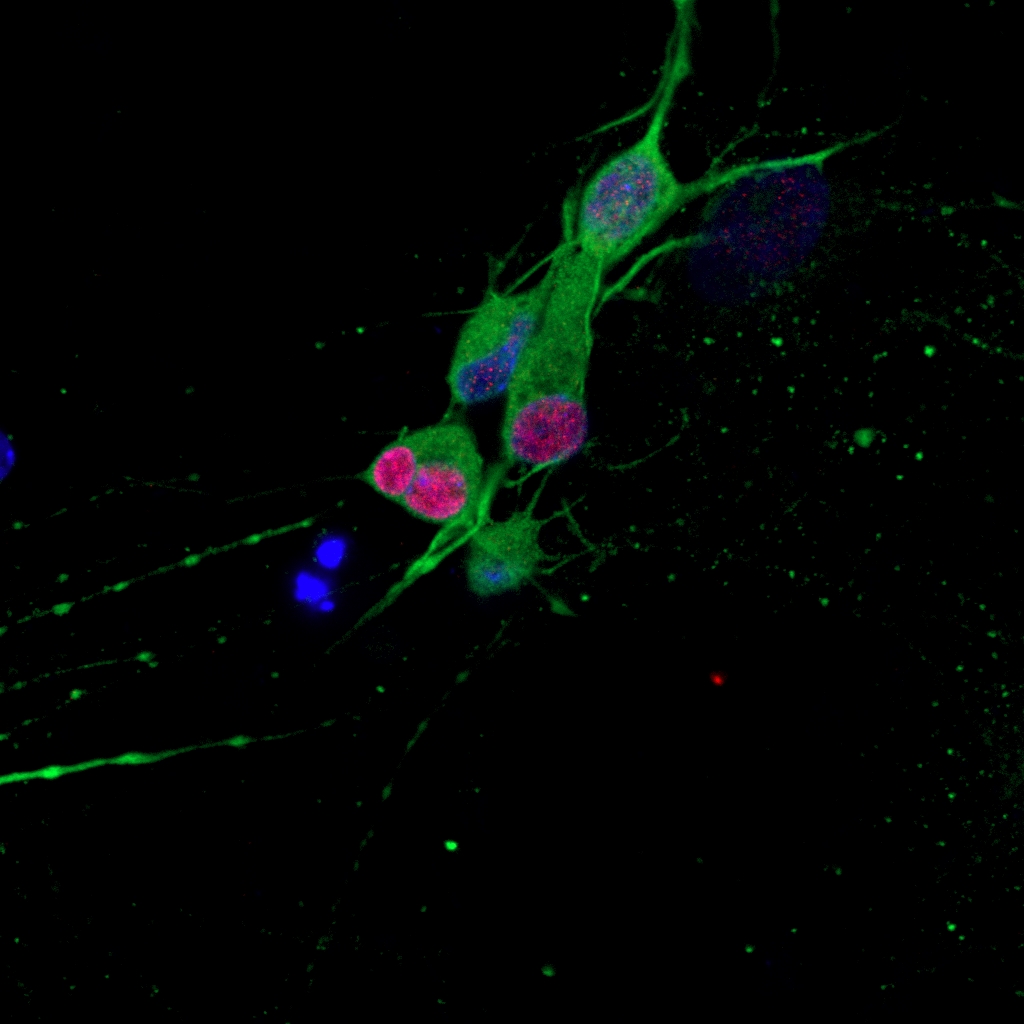

Supplement: Supplementary file 8 — Source data Fig. 4 [file 44321_2025_323_MOESM8_ESM.zip › Figure 4/4G/TDP43.jpg]

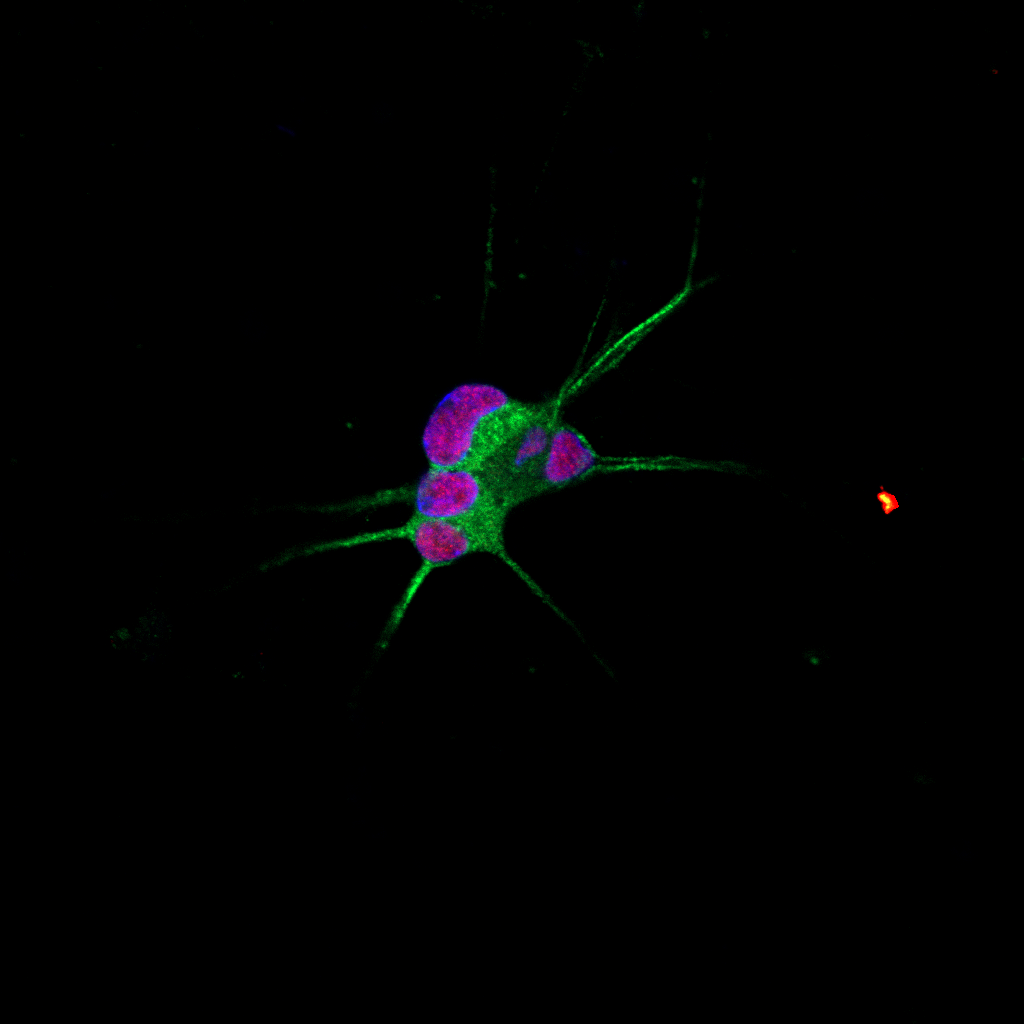

Supplement: Supplementary file 8 — Source data Fig. 4 [file 44321_2025_323_MOESM8_ESM.zip › Figure 4/4G/UC-12.tiff]

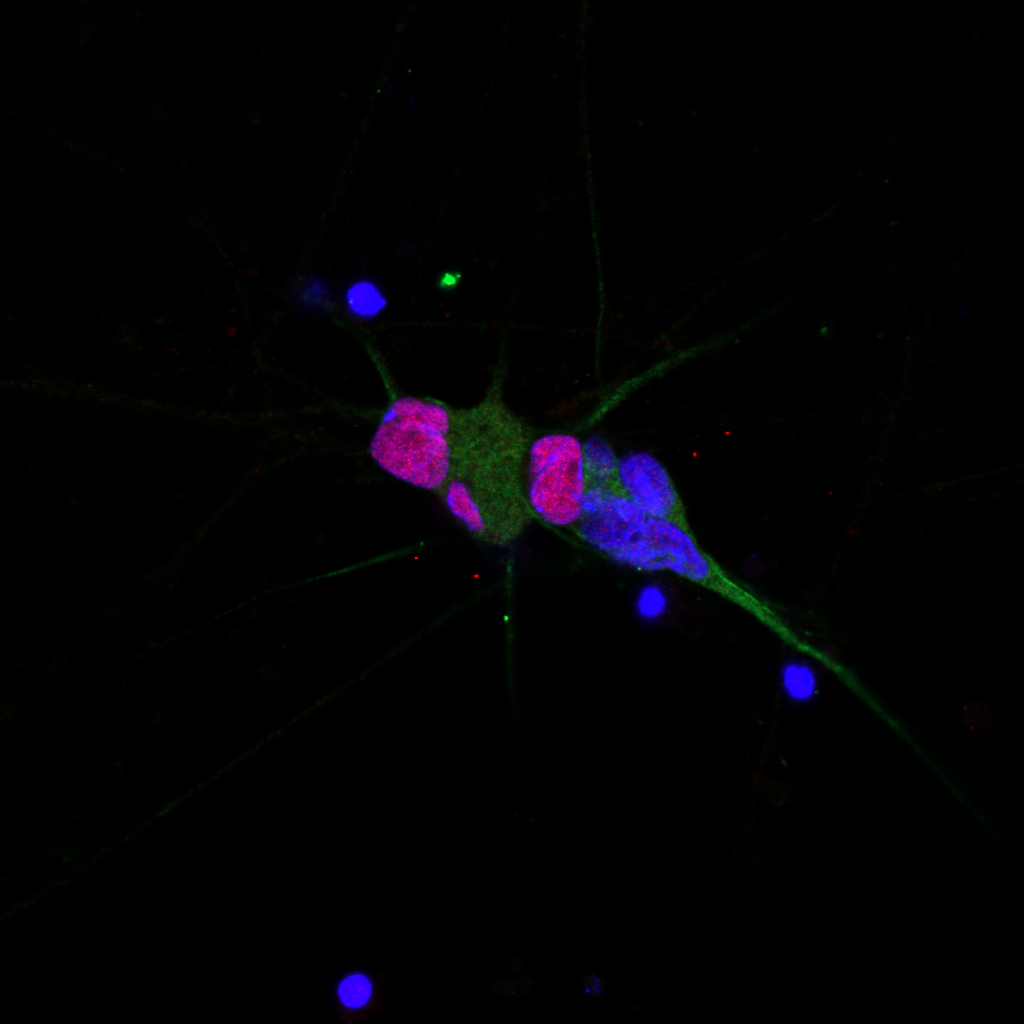

Supplement: Supplementary file 8 — Source data Fig. 4 [file 44321_2025_323_MOESM8_ESM.zip › Figure 4/4G/UC-H1.tif]

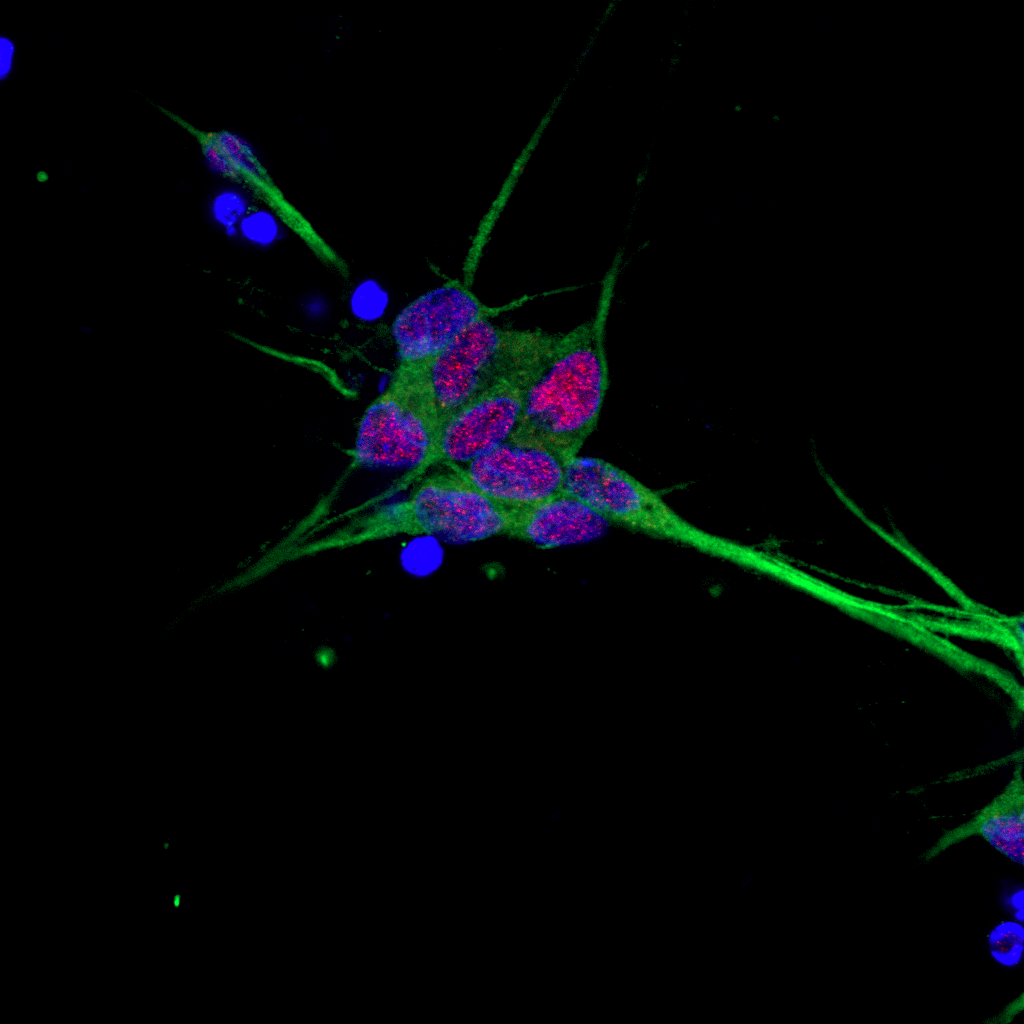

Supplement: Supplementary file 8 — Source data Fig. 4 [file 44321_2025_323_MOESM8_ESM.zip › Figure 4/4G/UC-H2.tiff]

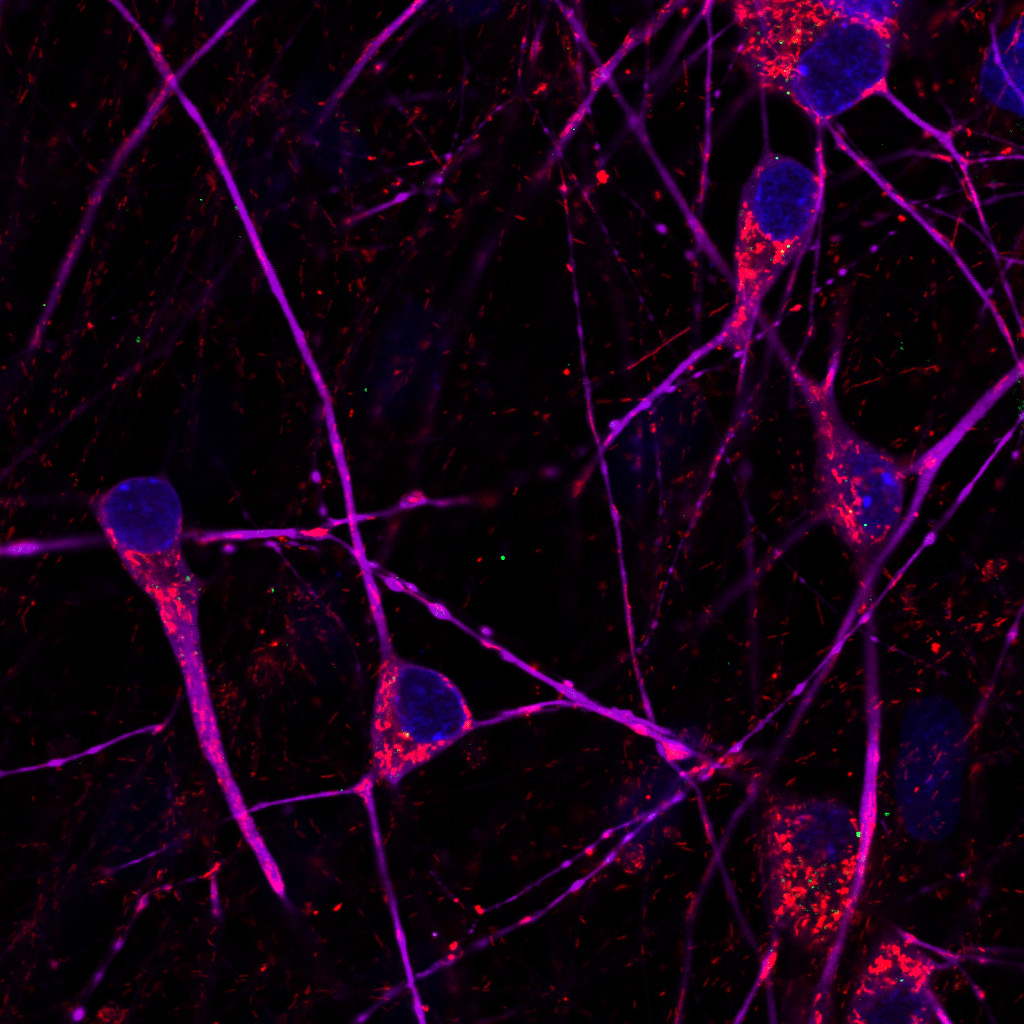

Supplement: Supplementary file 8 — Source data Fig. 4 [file 44321_2025_323_MOESM8_ESM.zip › Figure 4/4H/C9-Merge.tif]

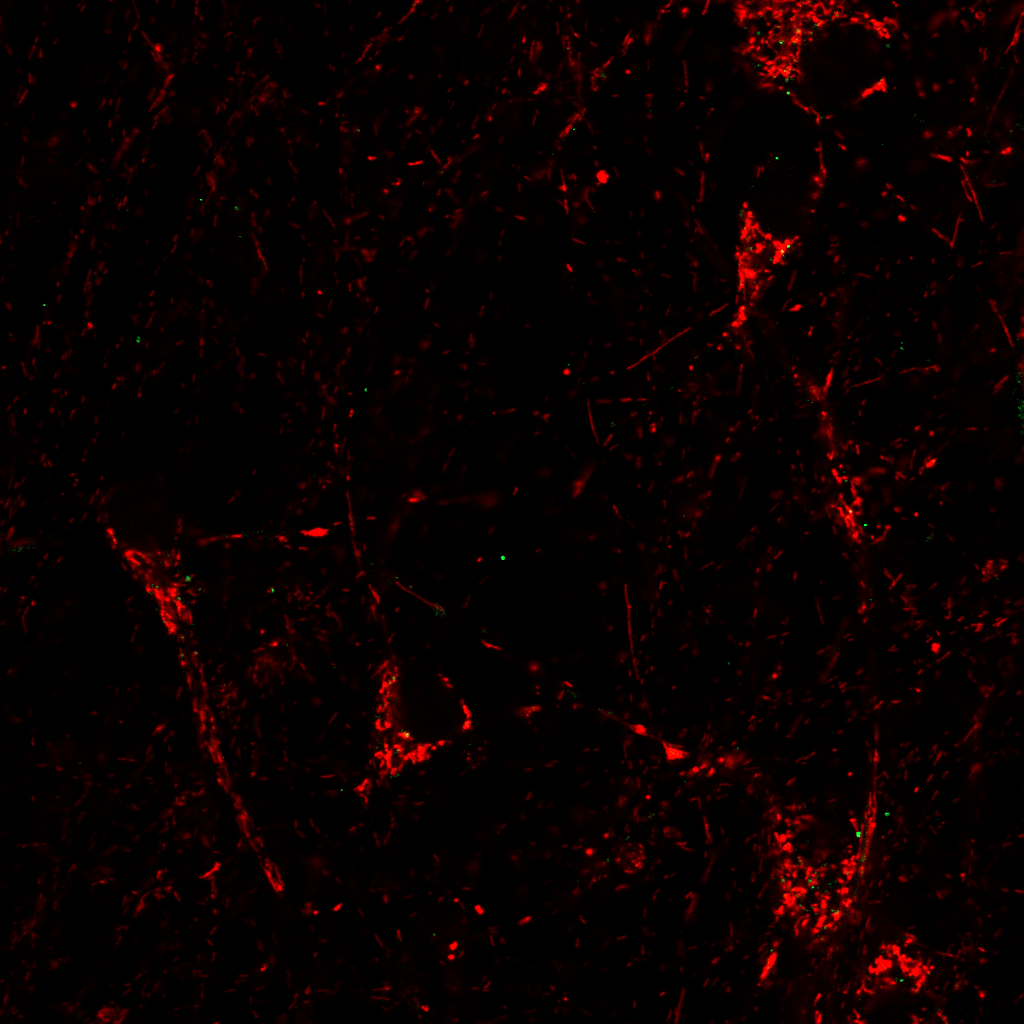

Supplement: Supplementary file 8 — Source data Fig. 4 [file 44321_2025_323_MOESM8_ESM.zip › Figure 4/4H/C9-MITO-LYSO.tif]

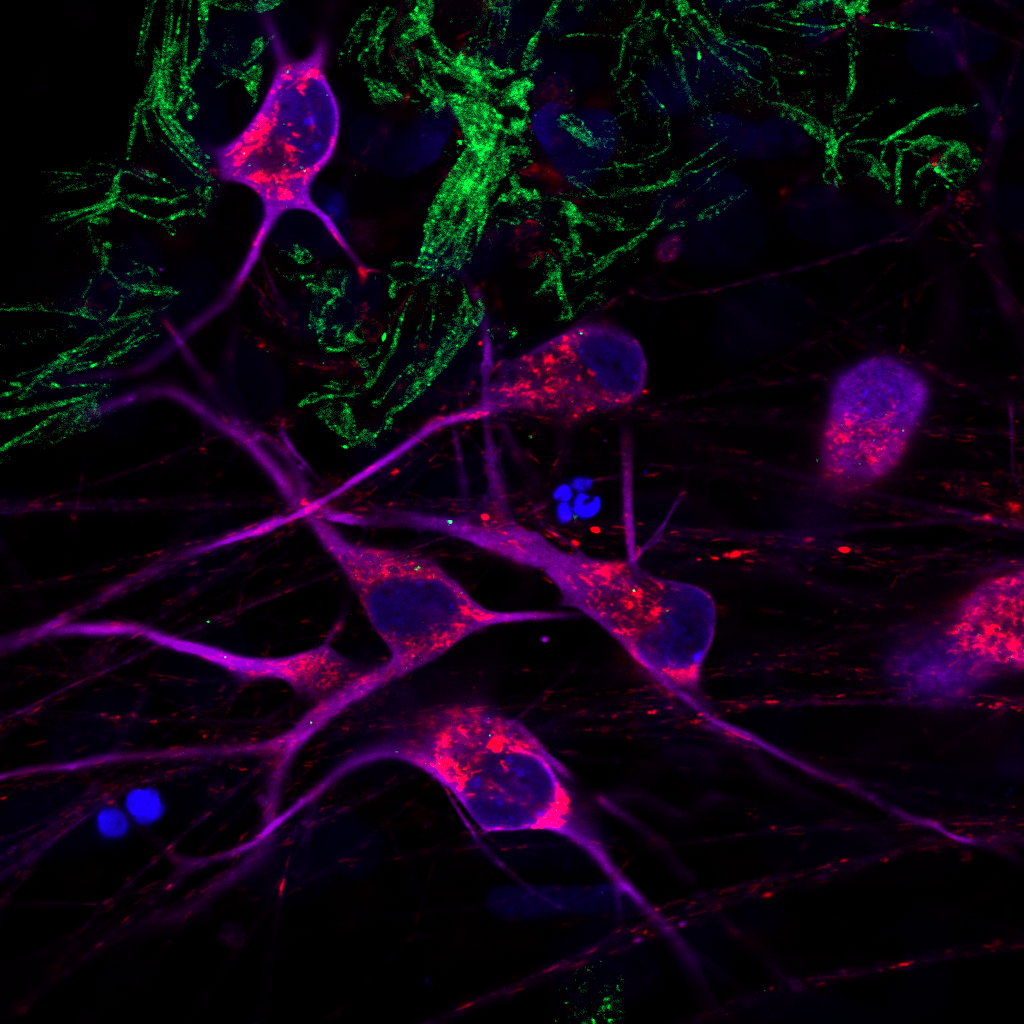

Supplement: Supplementary file 8 — Source data Fig. 4 [file 44321_2025_323_MOESM8_ESM.zip › Figure 4/4H/SOD1-Merge.tif]

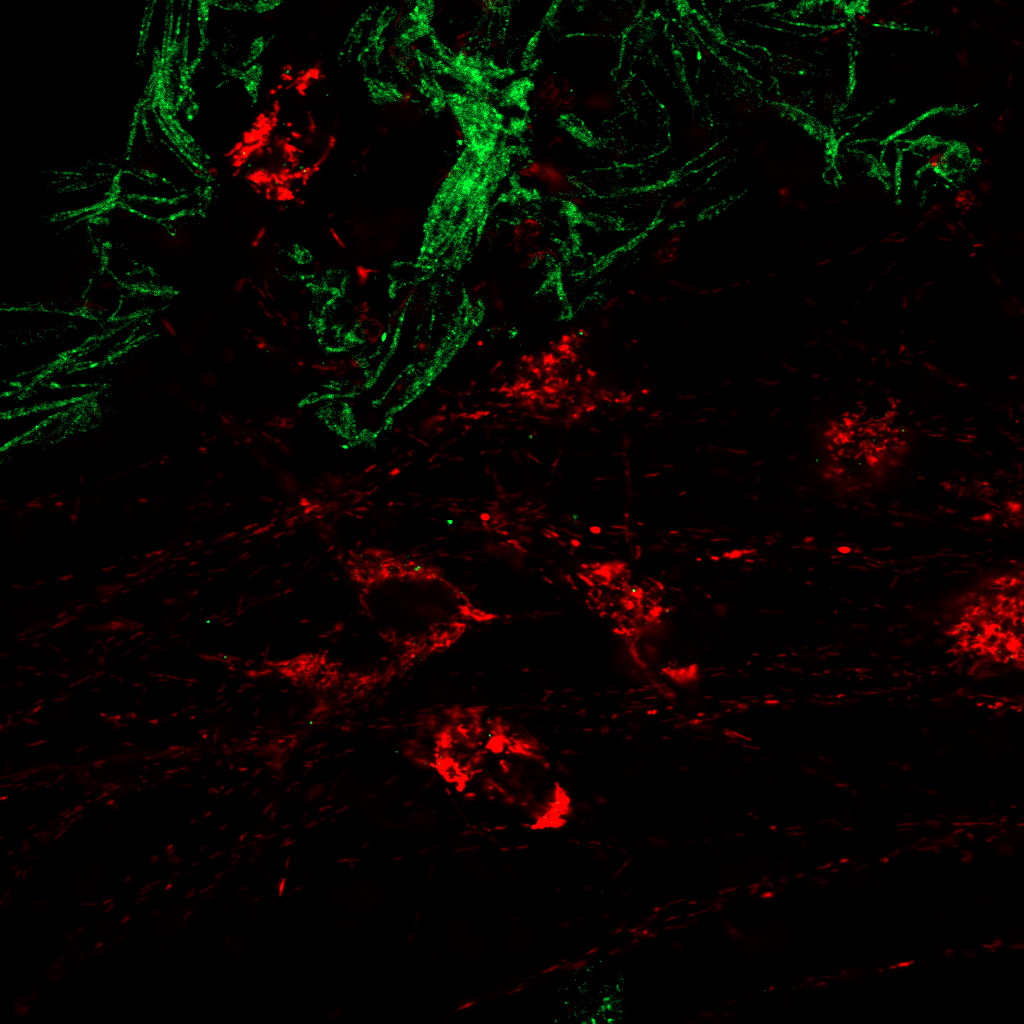

Supplement: Supplementary file 8 — Source data Fig. 4 [file 44321_2025_323_MOESM8_ESM.zip › Figure 4/4H/SOD1-MITO-LYSO.tif]

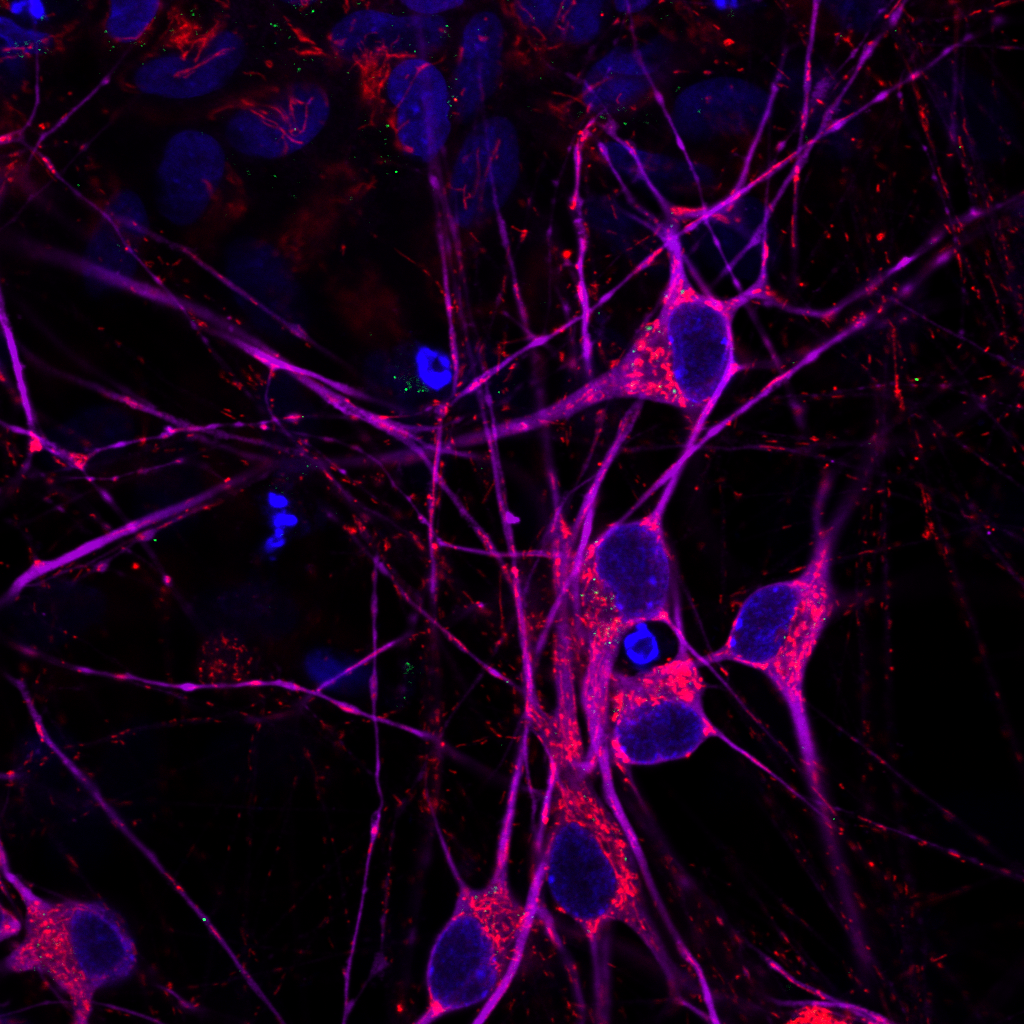

Supplement: Supplementary file 8 — Source data Fig. 4 [file 44321_2025_323_MOESM8_ESM.zip › Figure 4/4H/TDP-43-Merge.tif]

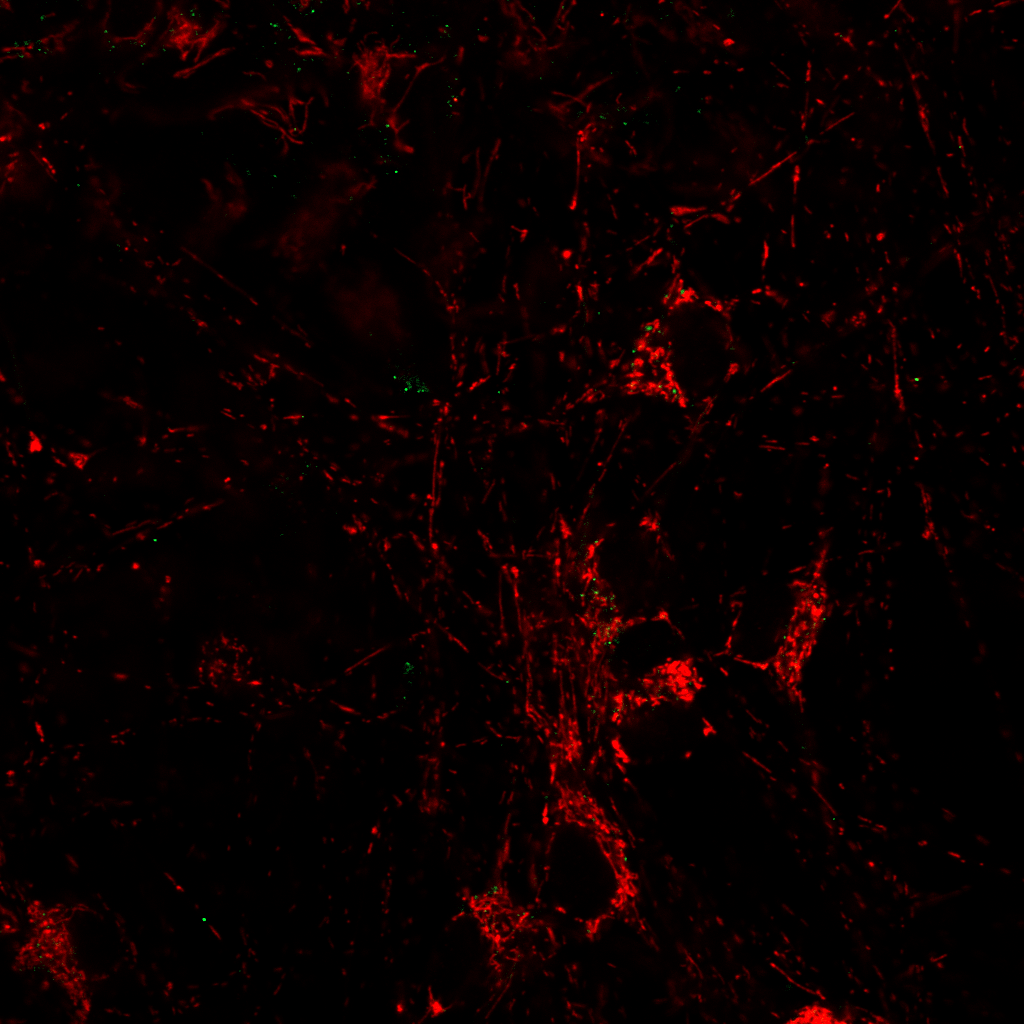

Supplement: Supplementary file 8 — Source data Fig. 4 [file 44321_2025_323_MOESM8_ESM.zip › Figure 4/4H/TDP-43-MITO-LYSO.tif]

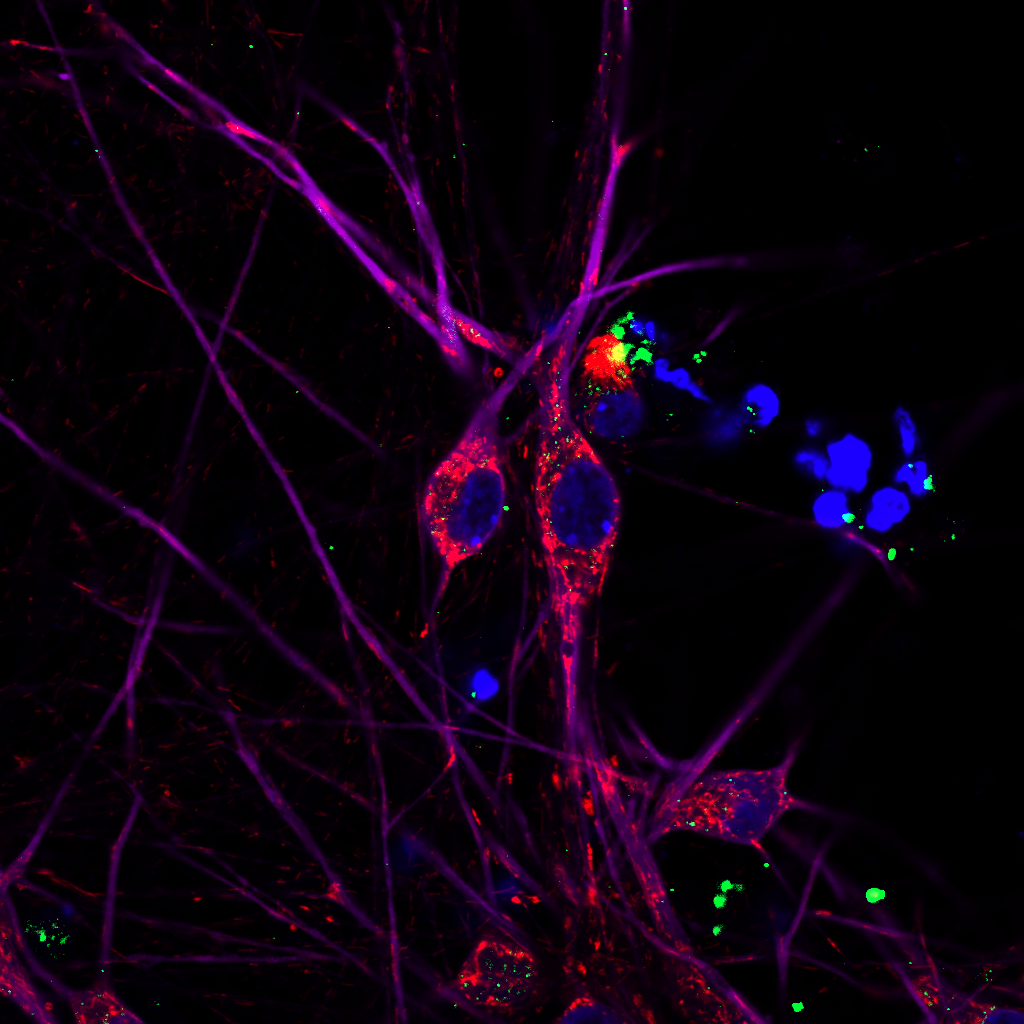

Supplement: Supplementary file 8 — Source data Fig. 4 [file 44321_2025_323_MOESM8_ESM.zip › Figure 4/4H/UC-12-Merge.tif]

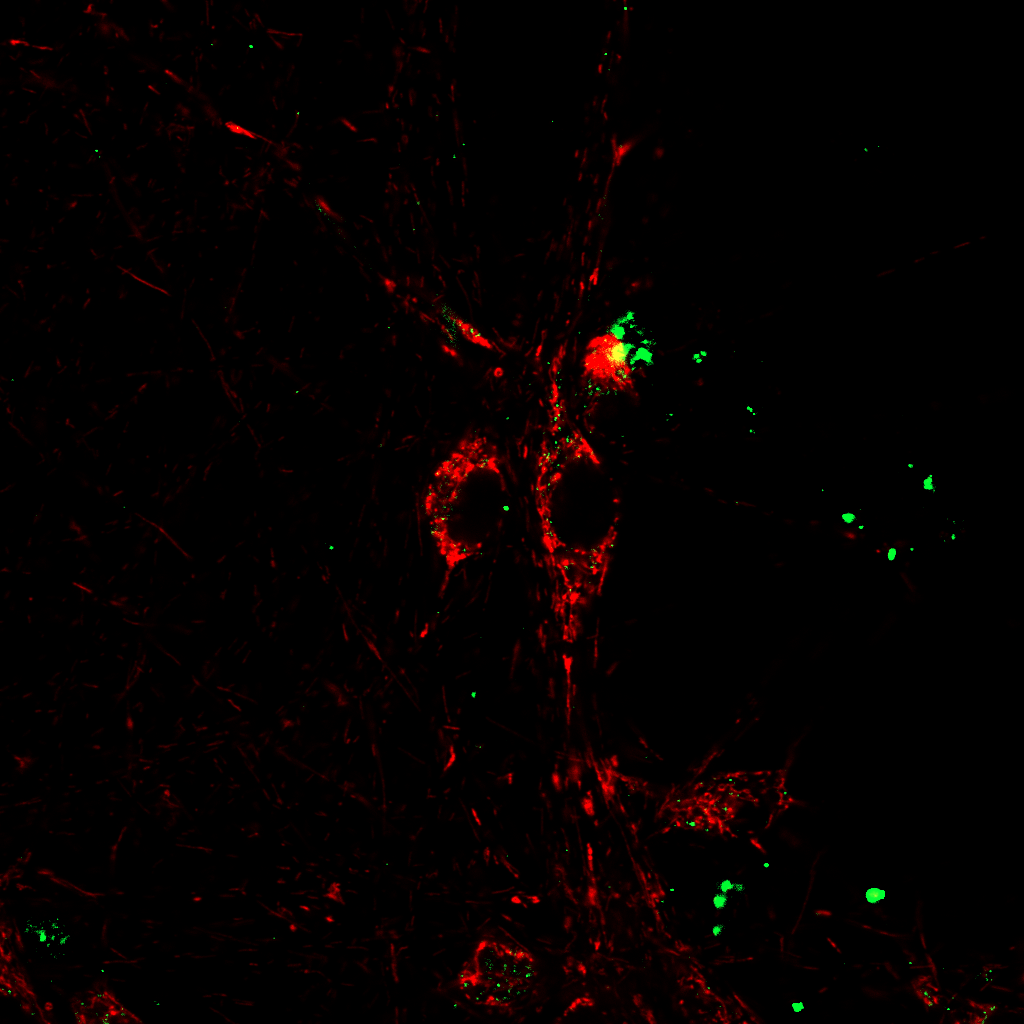

Supplement: Supplementary file 8 — Source data Fig. 4 [file 44321_2025_323_MOESM8_ESM.zip › Figure 4/4H/UC-12-MITO-LYSO.tif]

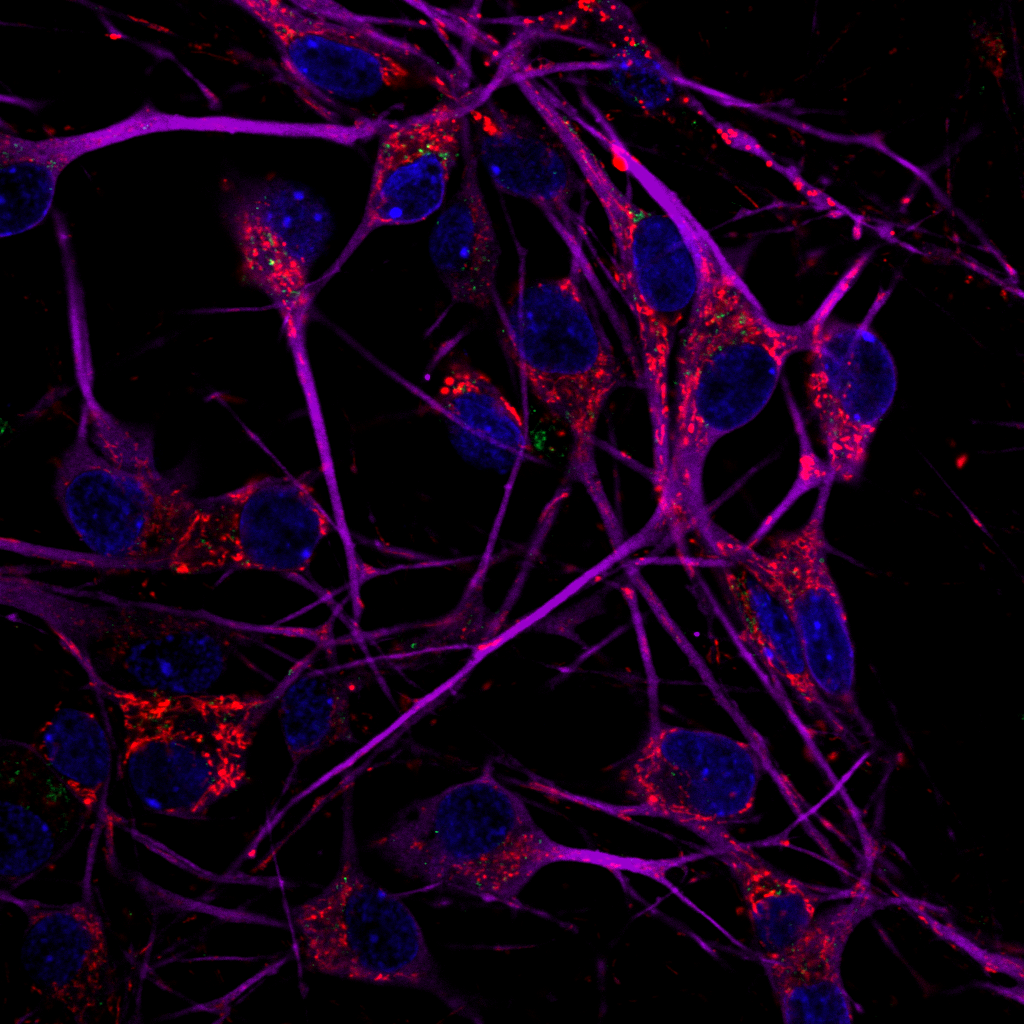

Supplement: Supplementary file 8 — Source data Fig. 4 [file 44321_2025_323_MOESM8_ESM.zip › Figure 4/4H/UC-H1-Merge.tif]

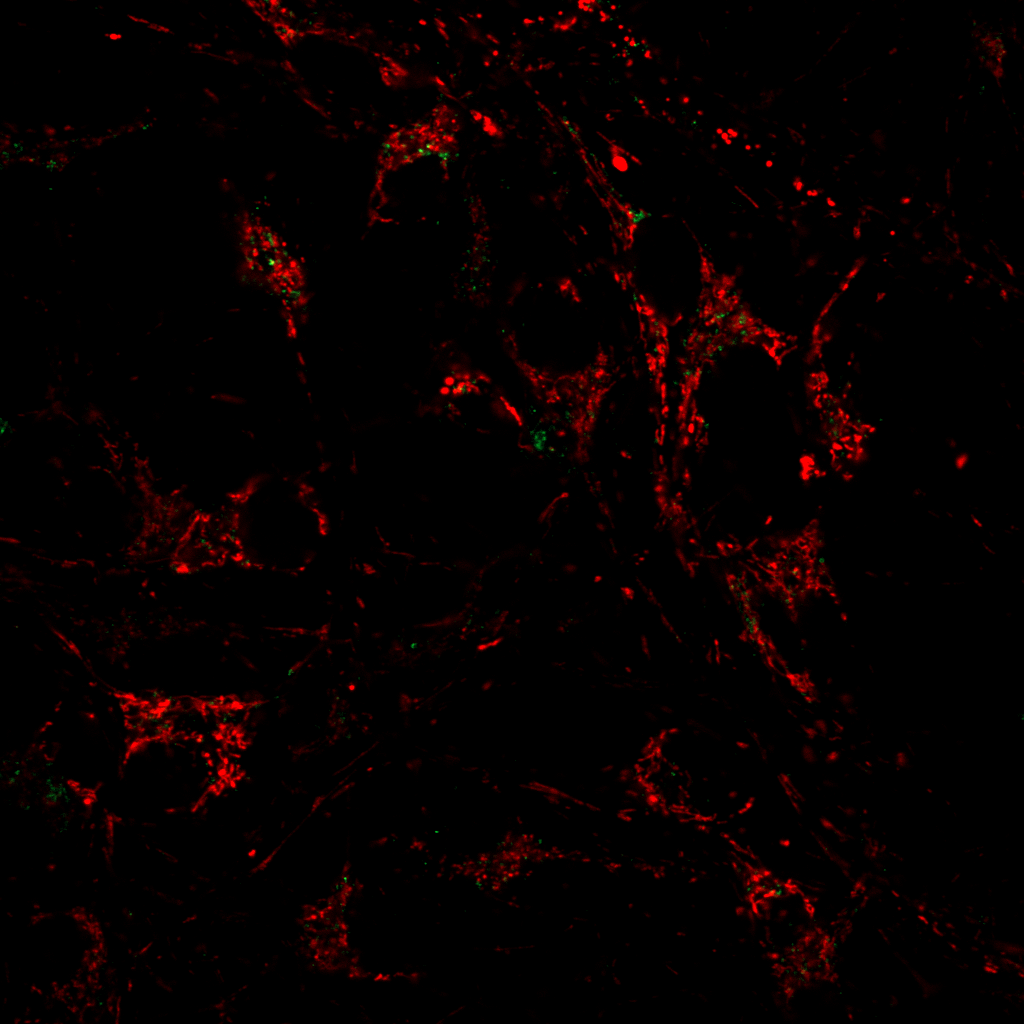

Supplement: Supplementary file 8 — Source data Fig. 4 [file 44321_2025_323_MOESM8_ESM.zip › Figure 4/4H/UC-H1-MITO-LYSO.tif]

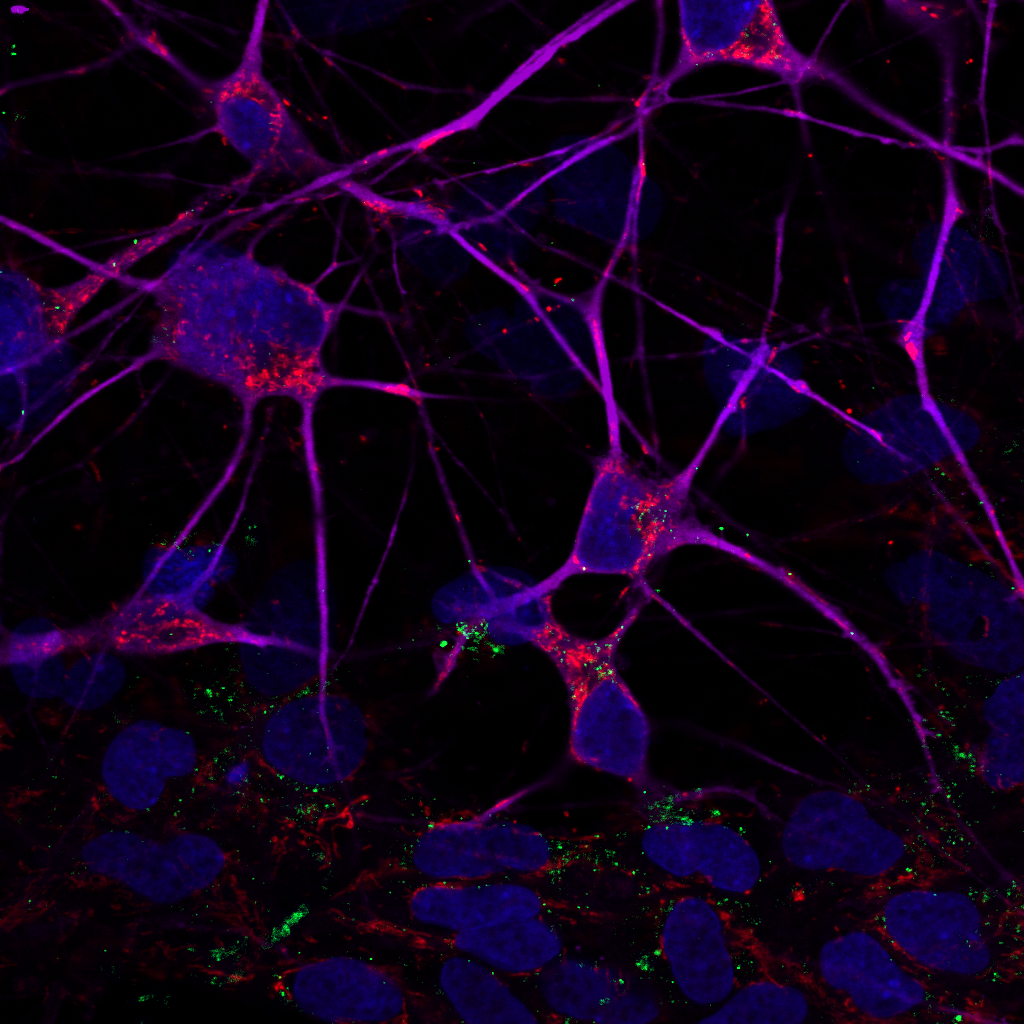

Supplement: Supplementary file 8 — Source data Fig. 4 [file 44321_2025_323_MOESM8_ESM.zip › Figure 4/4H/UC-H2-Merge.tif]

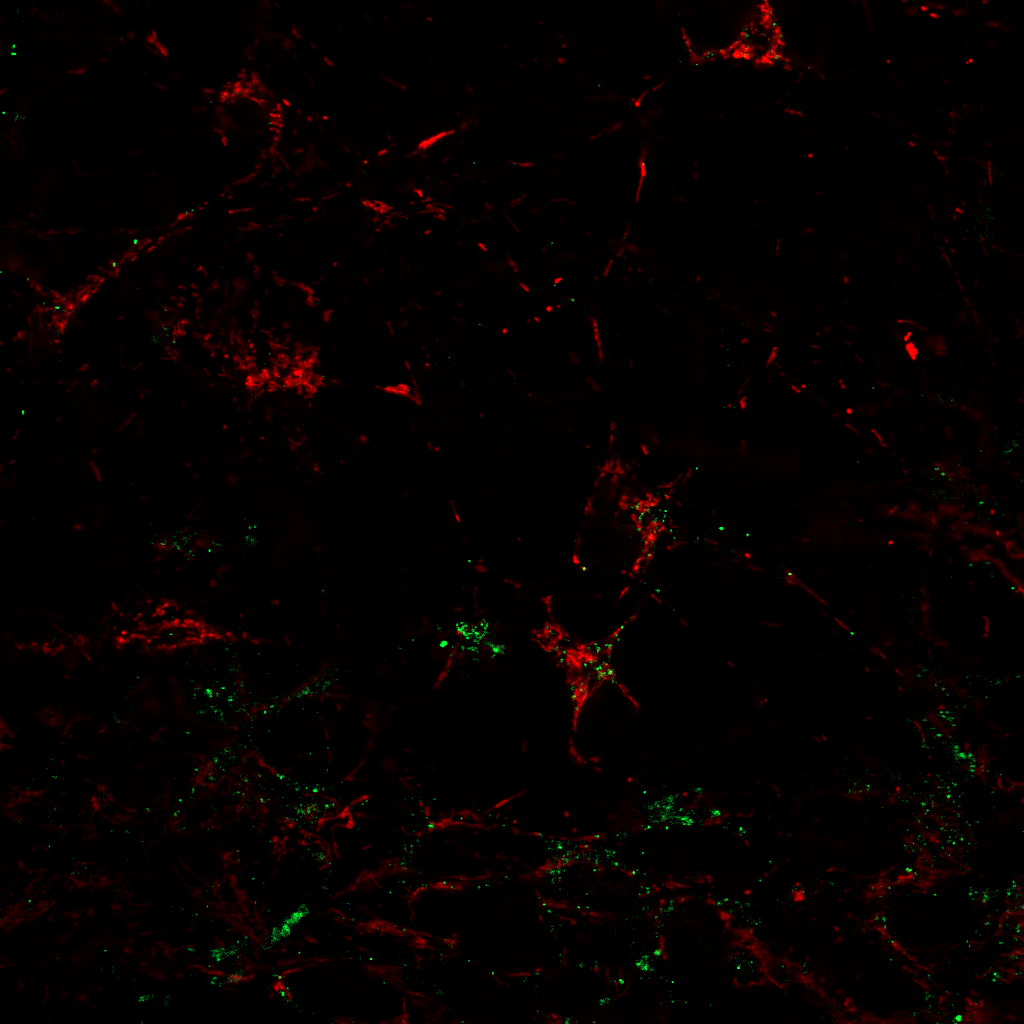

Supplement: Supplementary file 8 — Source data Fig. 4 [file 44321_2025_323_MOESM8_ESM.zip › Figure 4/4H/UC-H2-MITO-LYSO.tif]

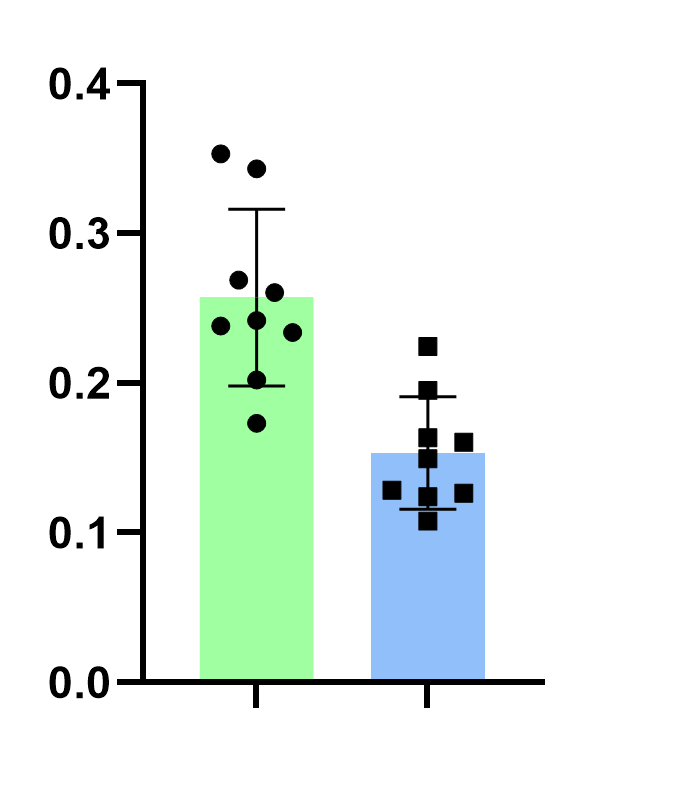

Supplement: Supplementary file 8 — Source data Fig. 4 [file 44321_2025_323_MOESM8_ESM.zip › Figure 4/4I/Mitophagy.tif]
