# Supplementary material for: Isoginkgetin antagonizes ALS pathologies in its animal and patient iPSC models via PINK1-Parkin-dependent mitophagy
Source: EMBO Mol Med. 2025 Oct 15;17(11):3139–73. doi: 10.1038/s44321-025-00323-2 (PMC12603167; doi:10.1038/s44321-025-00323-2)
Supplement: Supplementary file 7 — Source data Fig. 3 [file 44321_2025_323_MOESM7_ESM.zip › Figure 3/3E/biological repeat WB for analysis.pptx]

## Slide 1
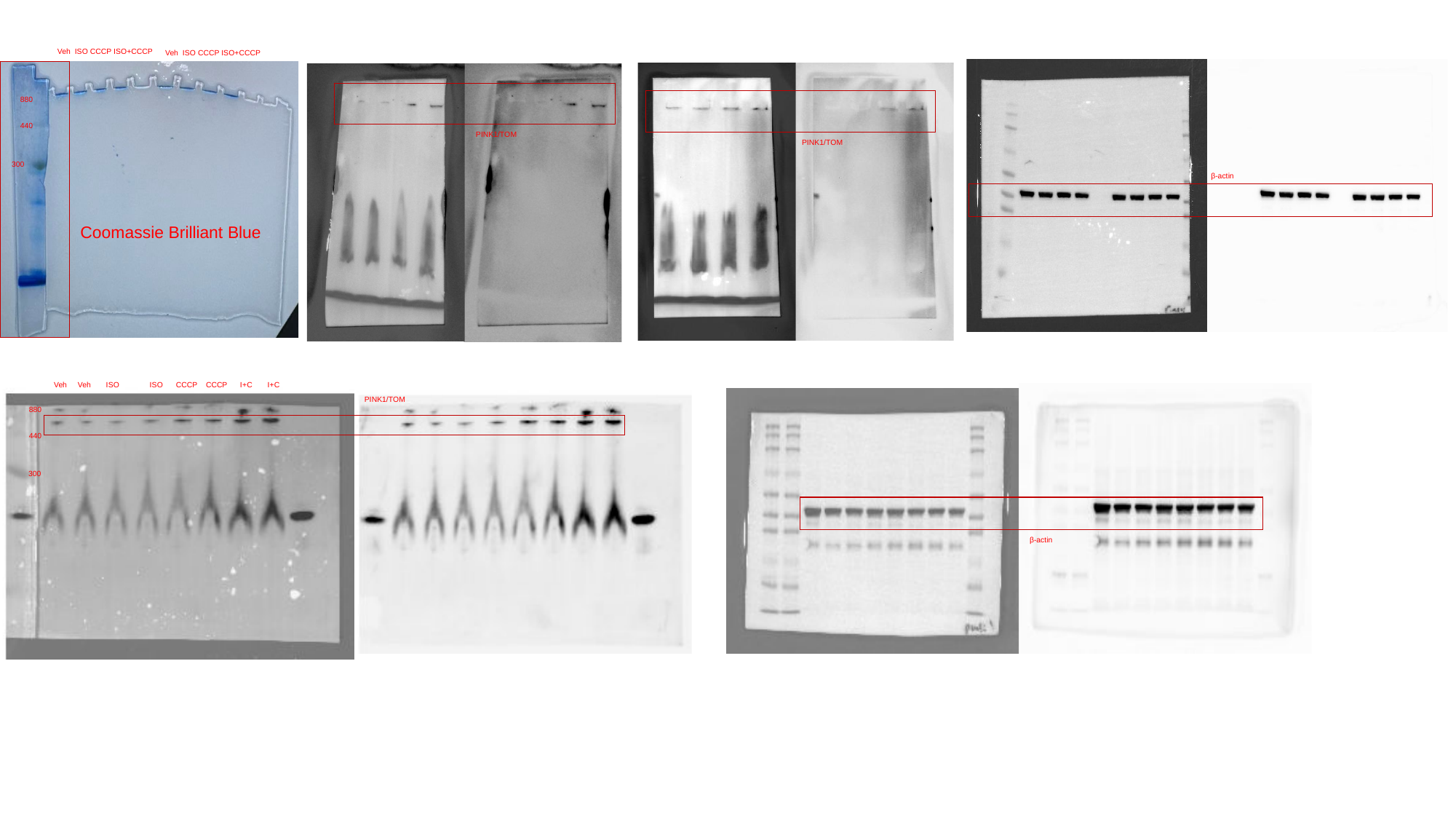

Veh ISO CCCP ISO+CCCP
Veh ISO CCCP ISO+CCCP
PINK1/TOM
PINK1/TOM
880
440
300
β-actin
Coomassie Brilliant Blue
Veh Veh ISO ISO CCCP CCCP I+C I+C
PINK1/TOM
880
440
300
β-actin
