# Supplementary figures and images for: Isoginkgetin antagonizes ALS pathologies in its animal and patient iPSC models via PINK1-Parkin-dependent mitophagy (part 2 of 3)
Source: EMBO Mol Med. 2025 Oct 15;17(11):3139–73. doi: 10.1038/s44321-025-00323-2 (PMC12603167; doi:10.1038/s44321-025-00323-2)

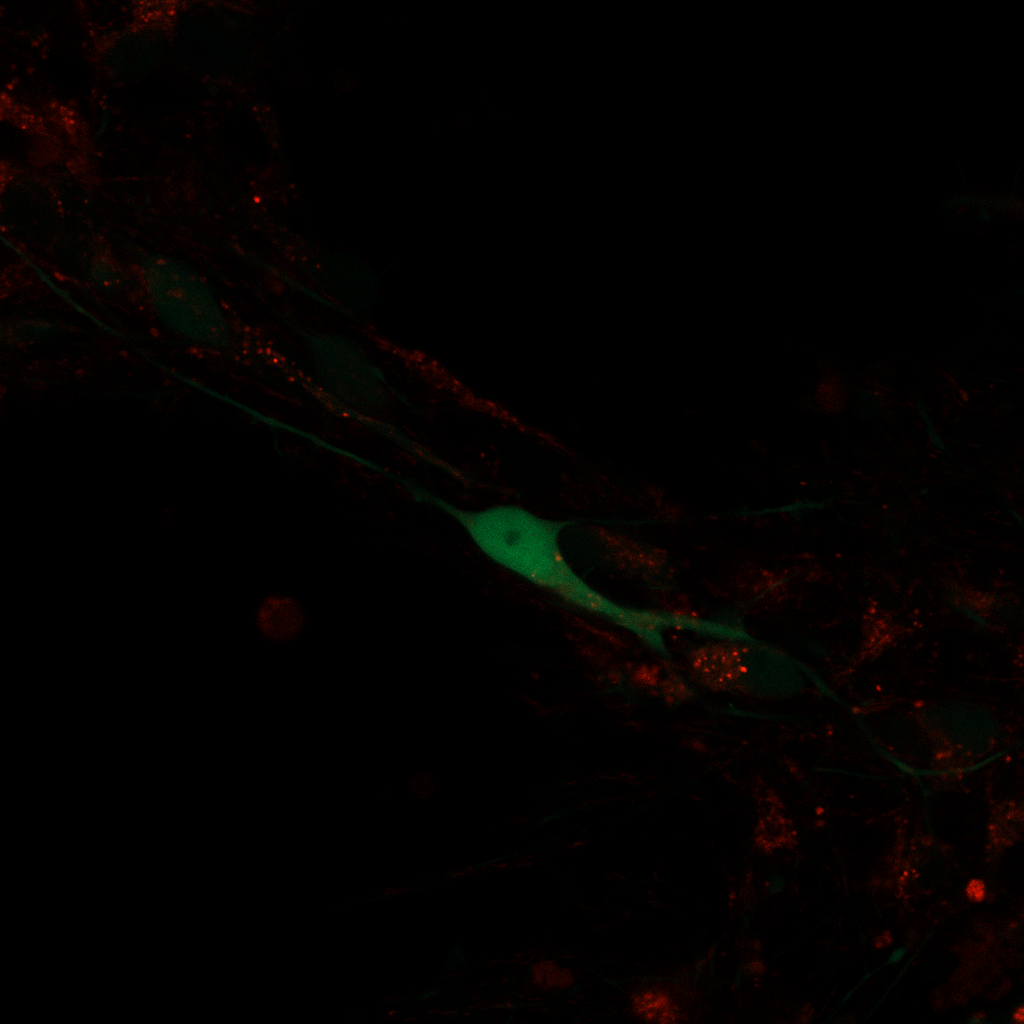

Supplement: Supplementary file 8 — Source data Fig. 4 [file 44321_2025_323_MOESM8_ESM.zip › Figure 4/4J/C9-SHOW-M.tif]

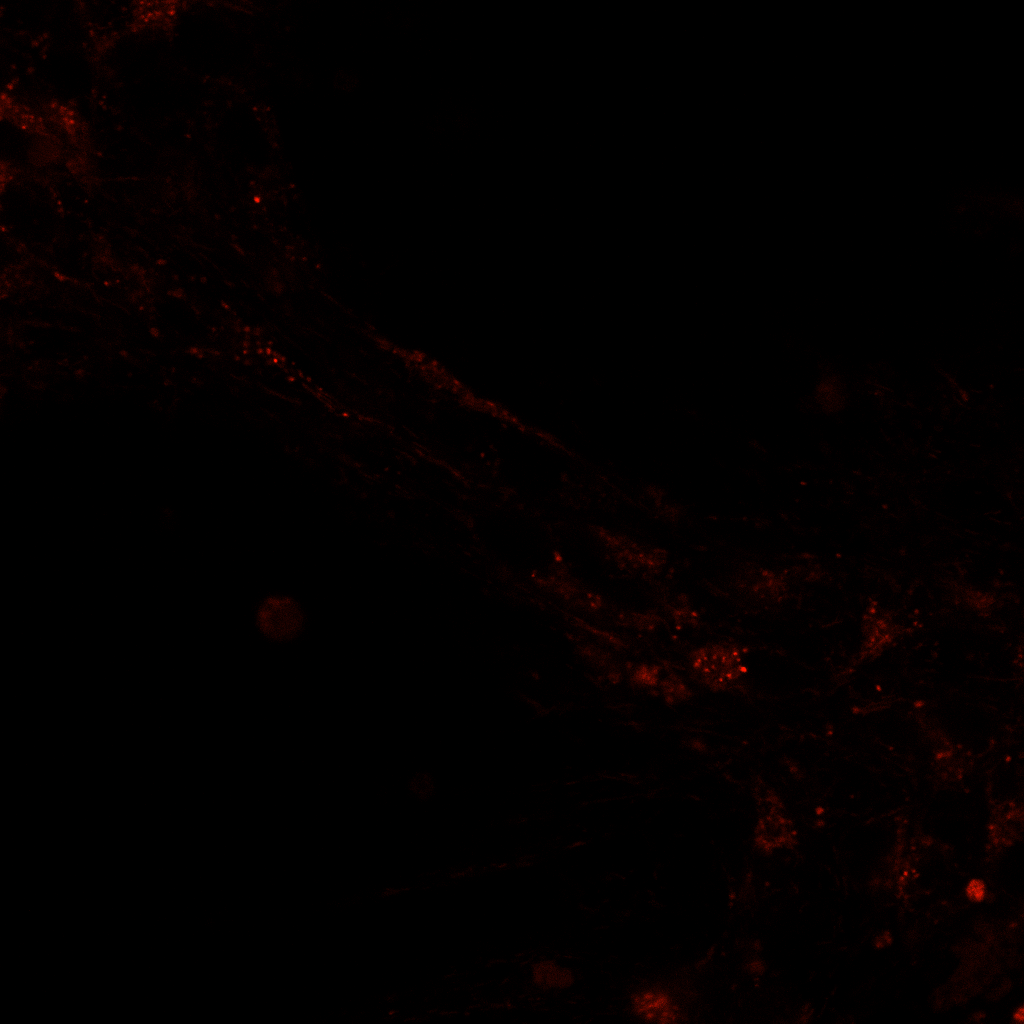

Supplement: Supplementary file 8 — Source data Fig. 4 [file 44321_2025_323_MOESM8_ESM.zip › Figure 4/4J/C9-SHOW-T.tif]

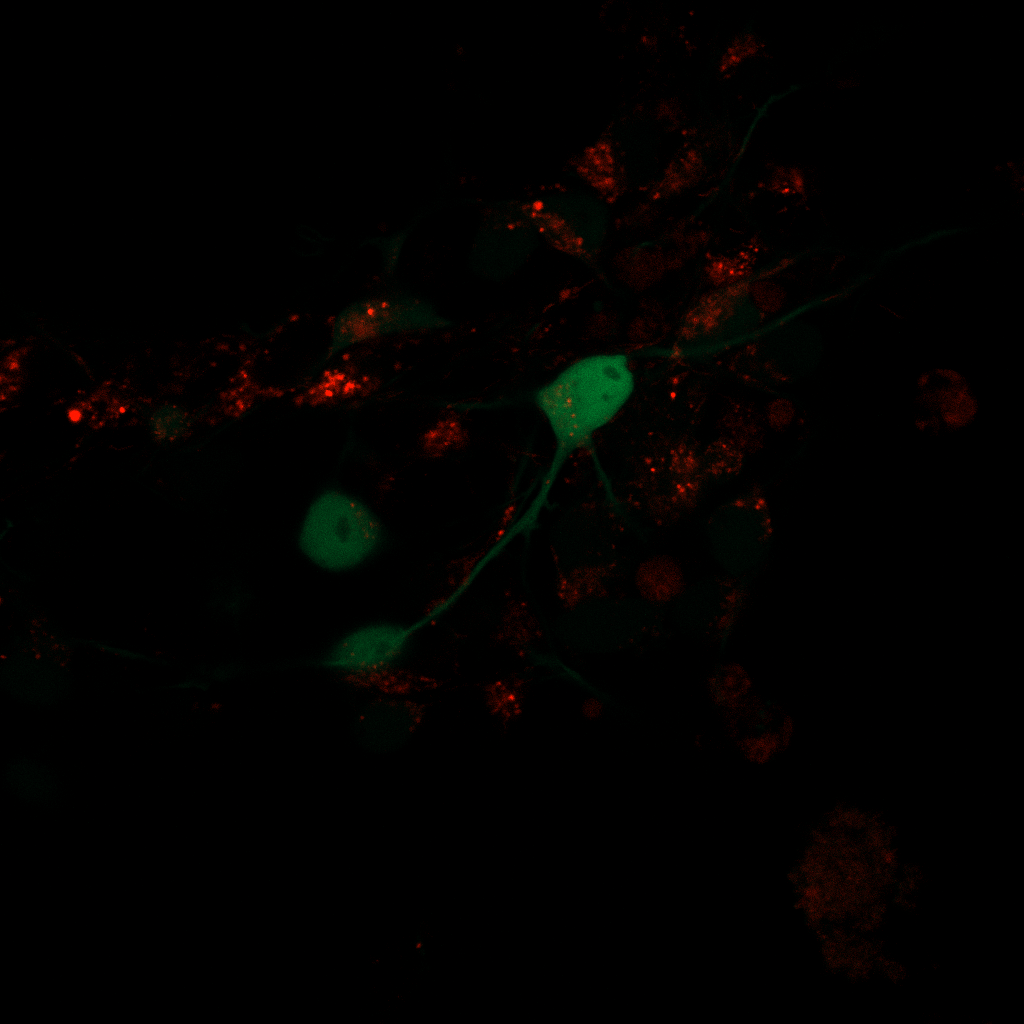

Supplement: Supplementary file 8 — Source data Fig. 4 [file 44321_2025_323_MOESM8_ESM.zip › Figure 4/4J/SOD1-SHOW-M.tif]

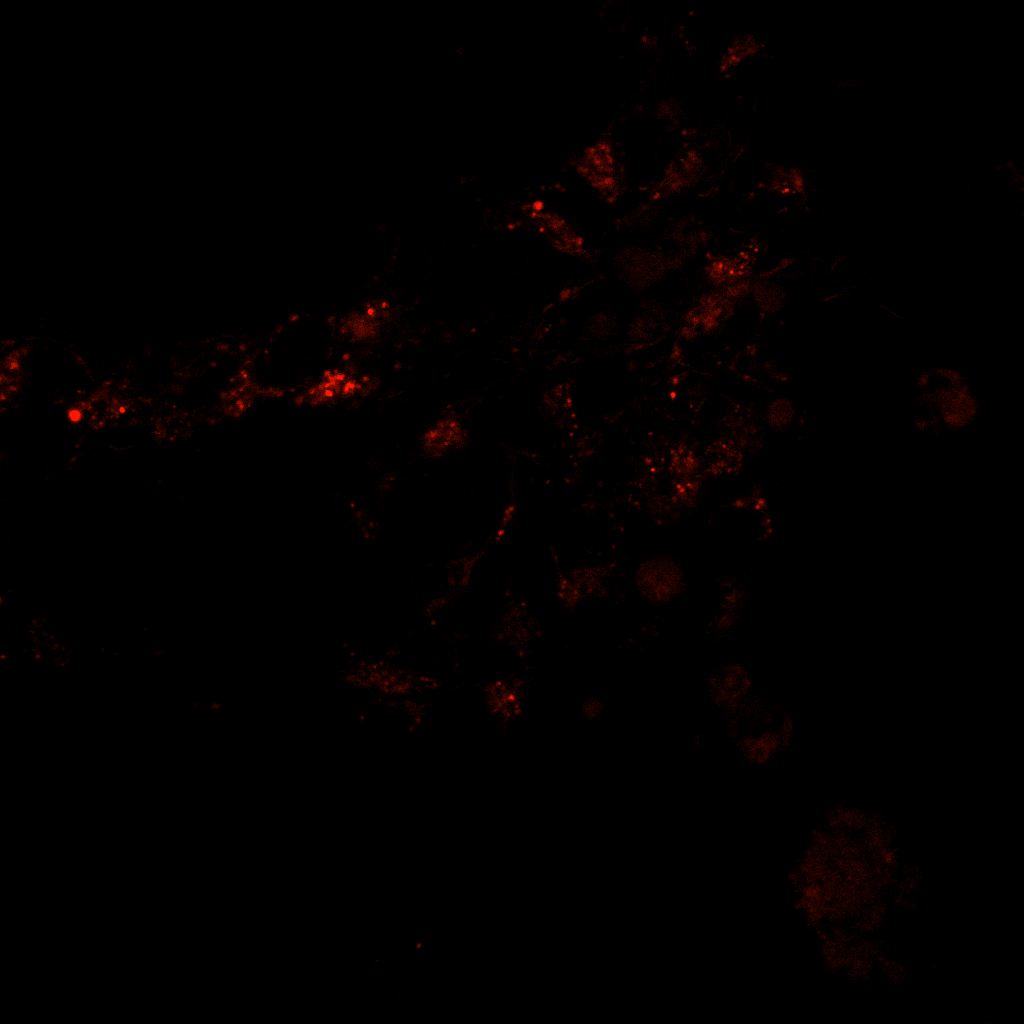

Supplement: Supplementary file 8 — Source data Fig. 4 [file 44321_2025_323_MOESM8_ESM.zip › Figure 4/4J/SOD1-SHOW-T.tif]

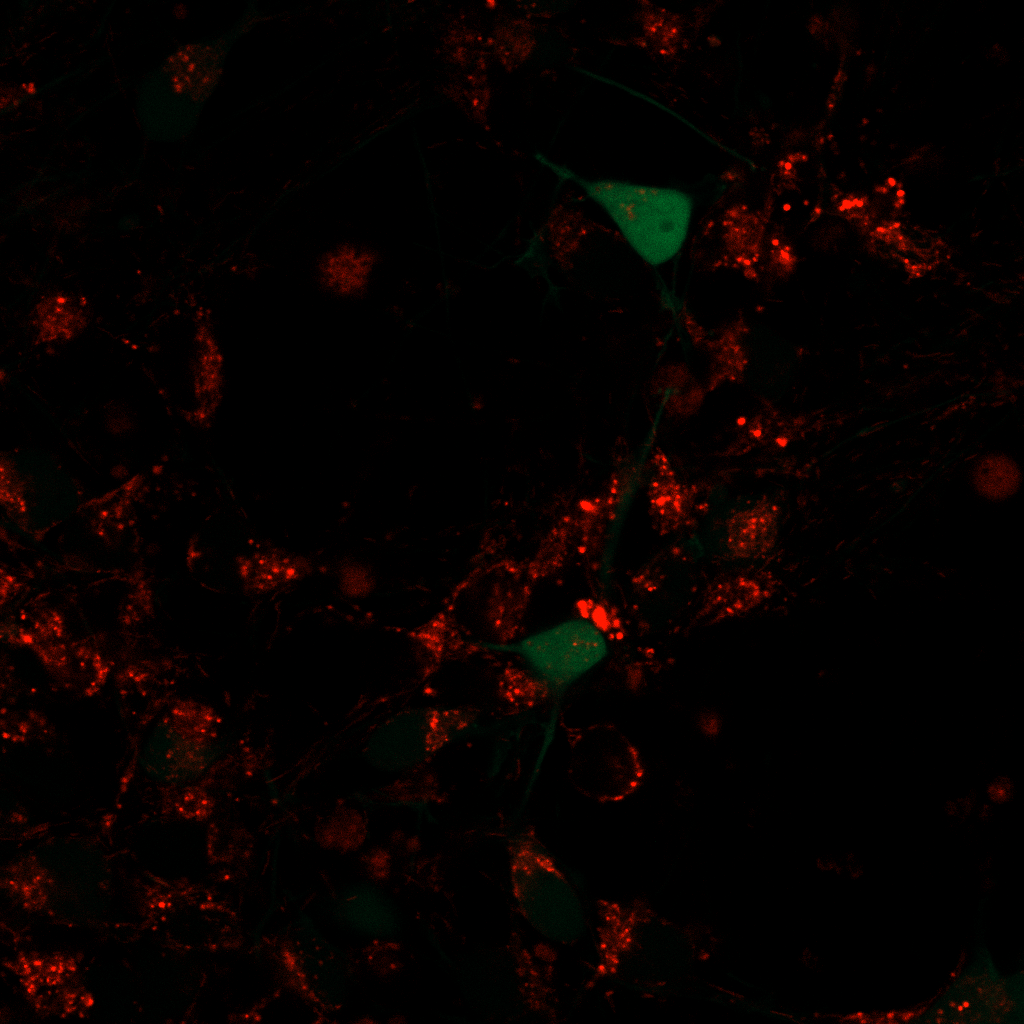

Supplement: Supplementary file 8 — Source data Fig. 4 [file 44321_2025_323_MOESM8_ESM.zip › Figure 4/4J/TDP-43-SHOW-M.tif]

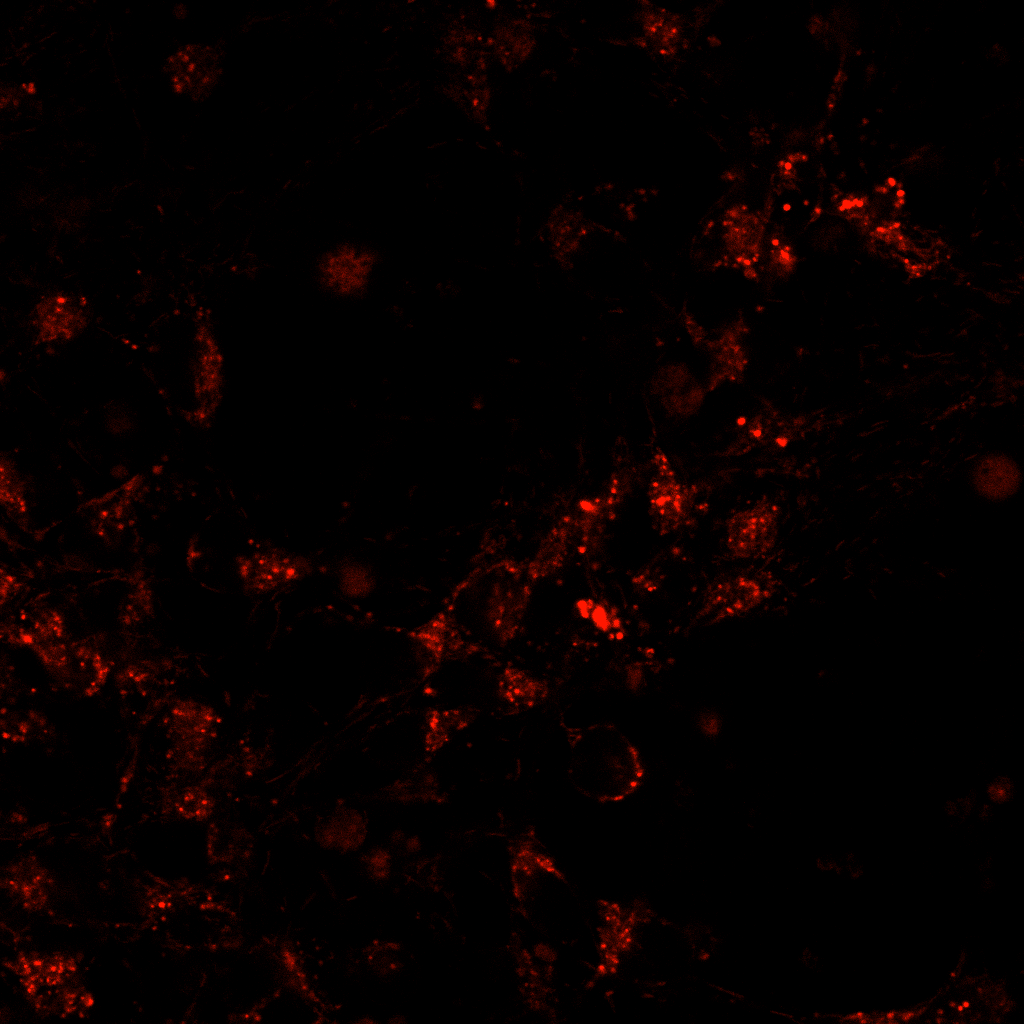

Supplement: Supplementary file 8 — Source data Fig. 4 [file 44321_2025_323_MOESM8_ESM.zip › Figure 4/4J/TDP-43-SHOW-T.tif]

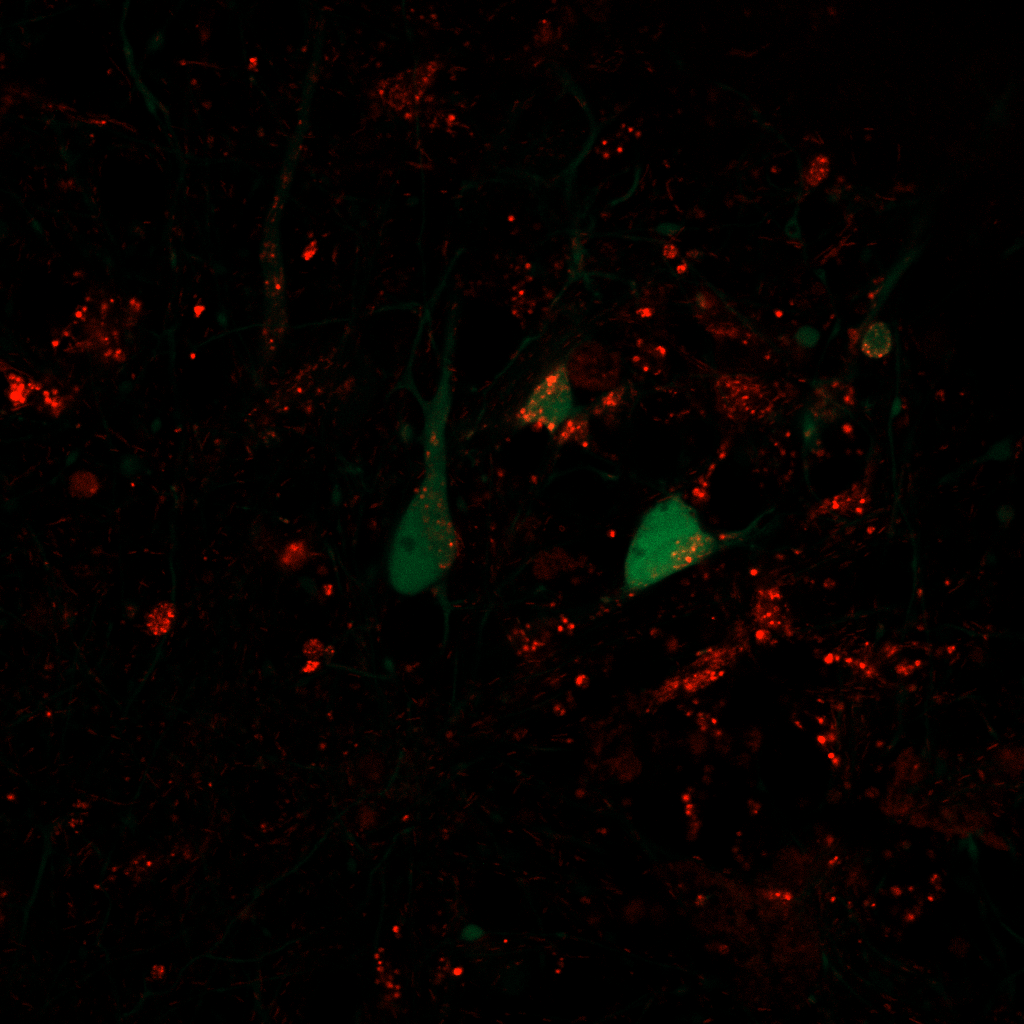

Supplement: Supplementary file 8 — Source data Fig. 4 [file 44321_2025_323_MOESM8_ESM.zip › Figure 4/4J/UC12-SHOW-M.tif]

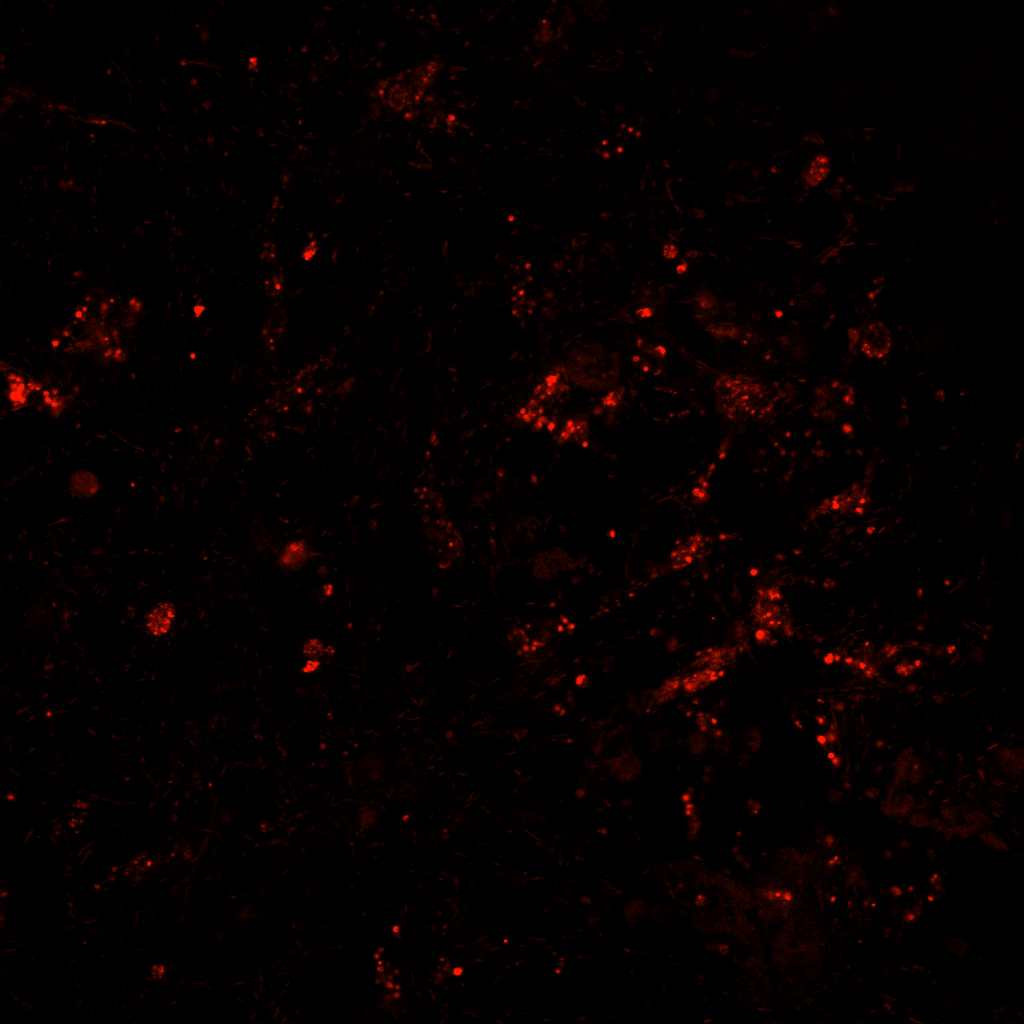

Supplement: Supplementary file 8 — Source data Fig. 4 [file 44321_2025_323_MOESM8_ESM.zip › Figure 4/4J/UC12-SHOW-T.tif]

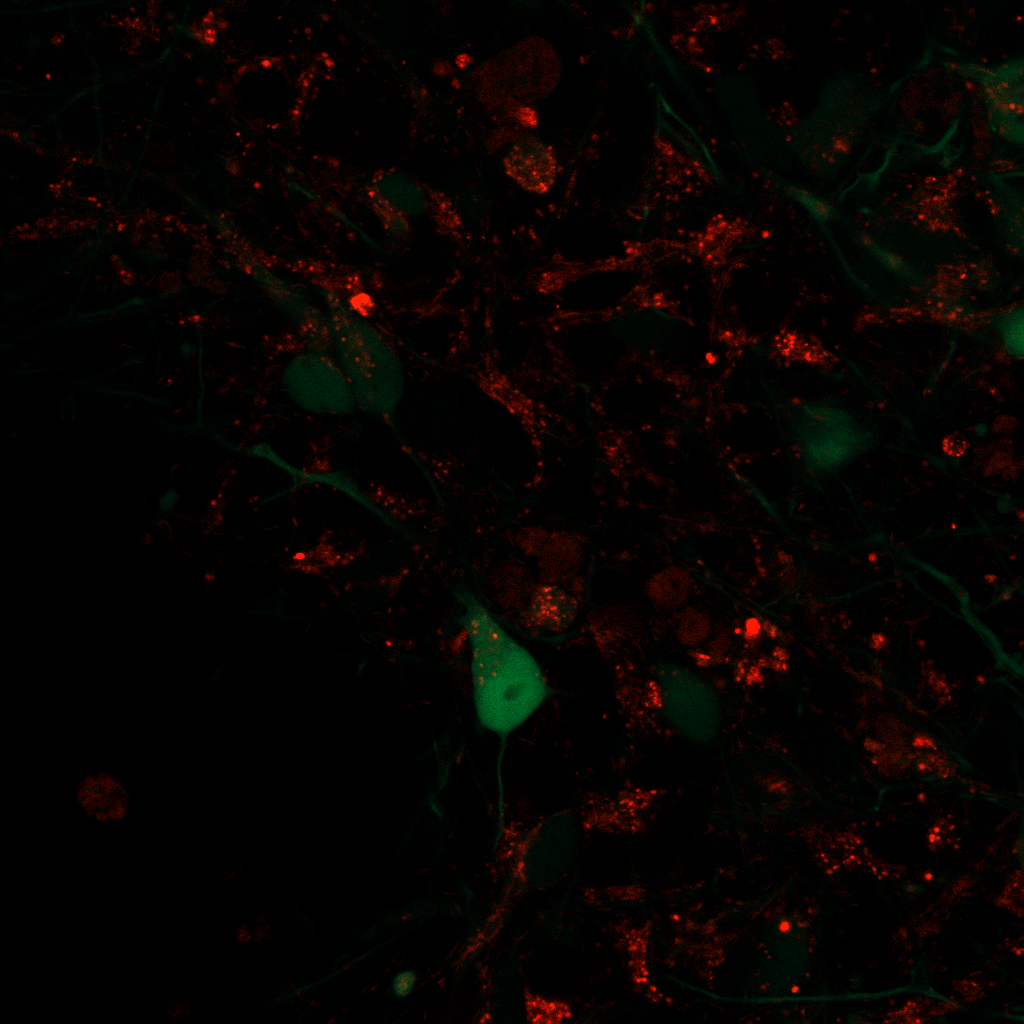

Supplement: Supplementary file 8 — Source data Fig. 4 [file 44321_2025_323_MOESM8_ESM.zip › Figure 4/4J/UC-H1-SHOW-M.tif]

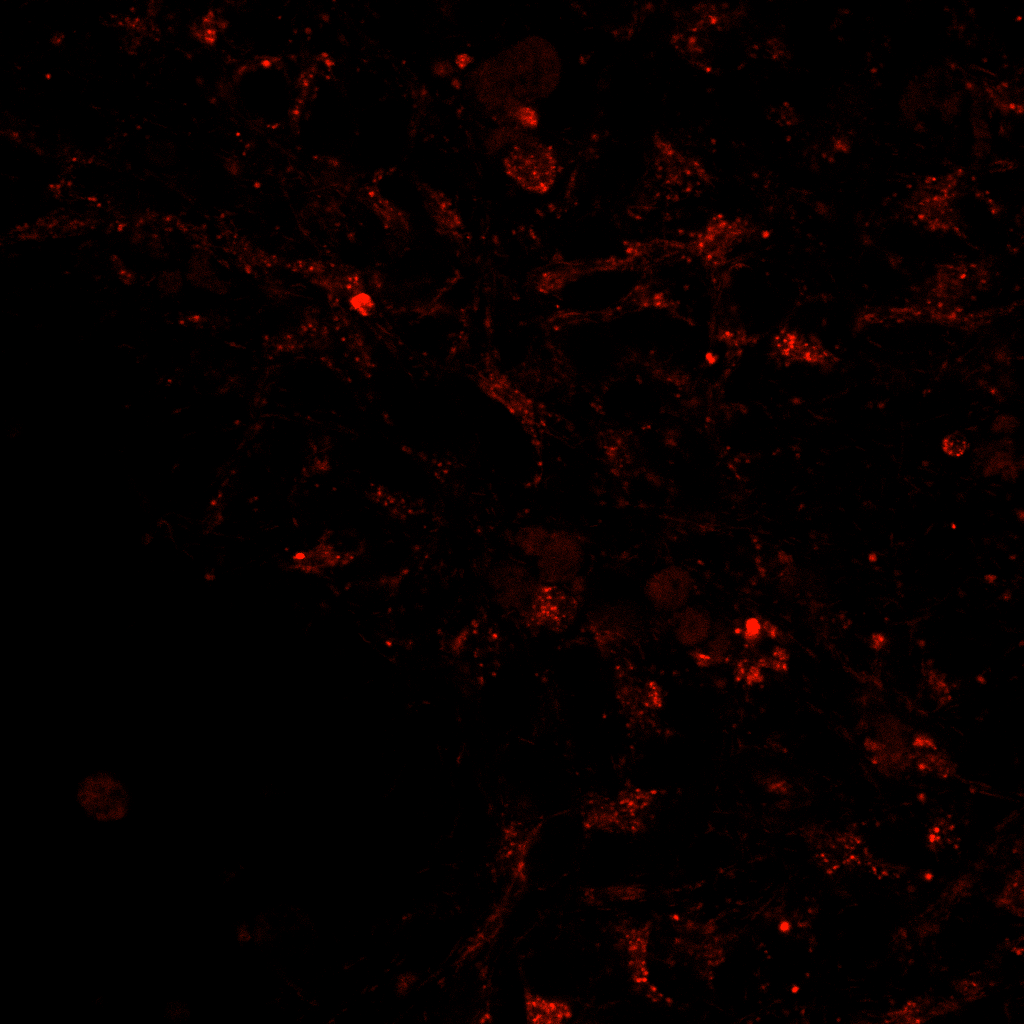

Supplement: Supplementary file 8 — Source data Fig. 4 [file 44321_2025_323_MOESM8_ESM.zip › Figure 4/4J/UC-H1-SHOW-T.tif]

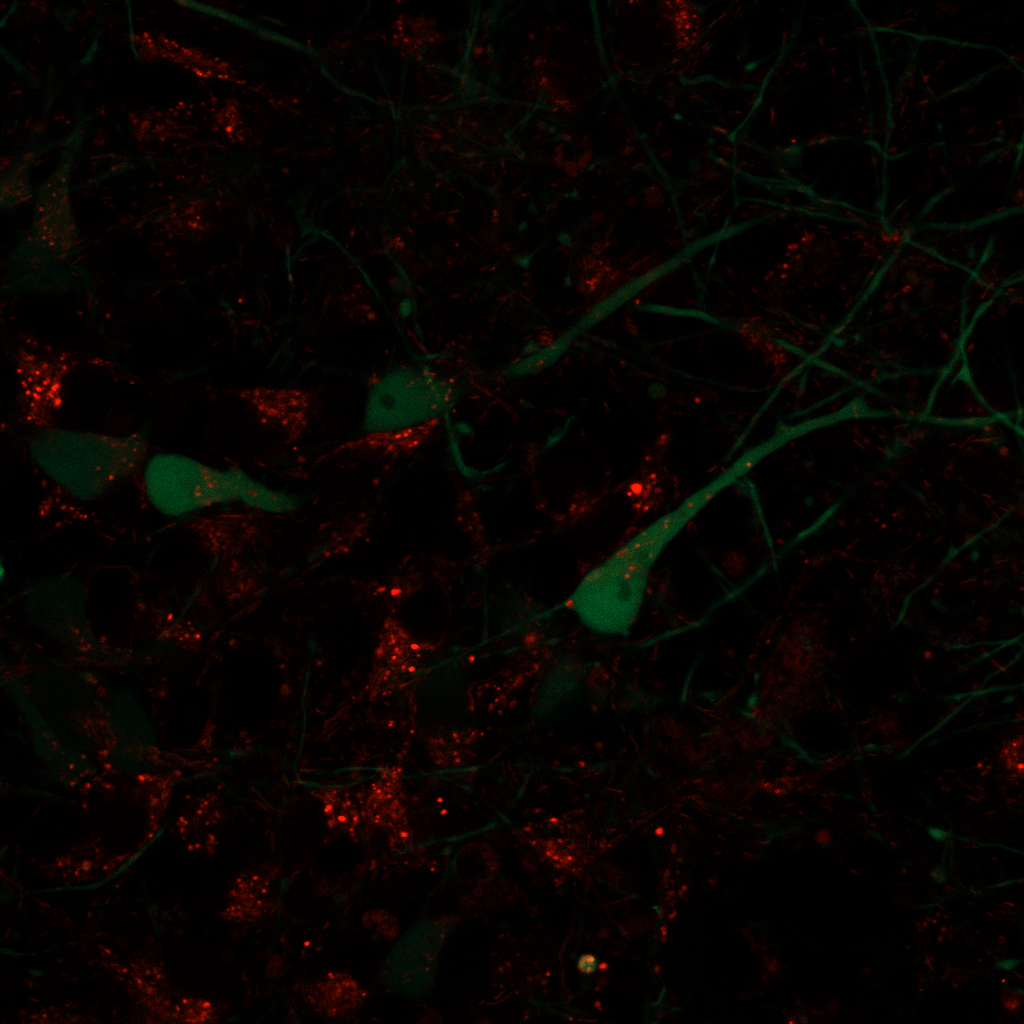

Supplement: Supplementary file 8 — Source data Fig. 4 [file 44321_2025_323_MOESM8_ESM.zip › Figure 4/4J/UC-H2-SHOW-M.tif]

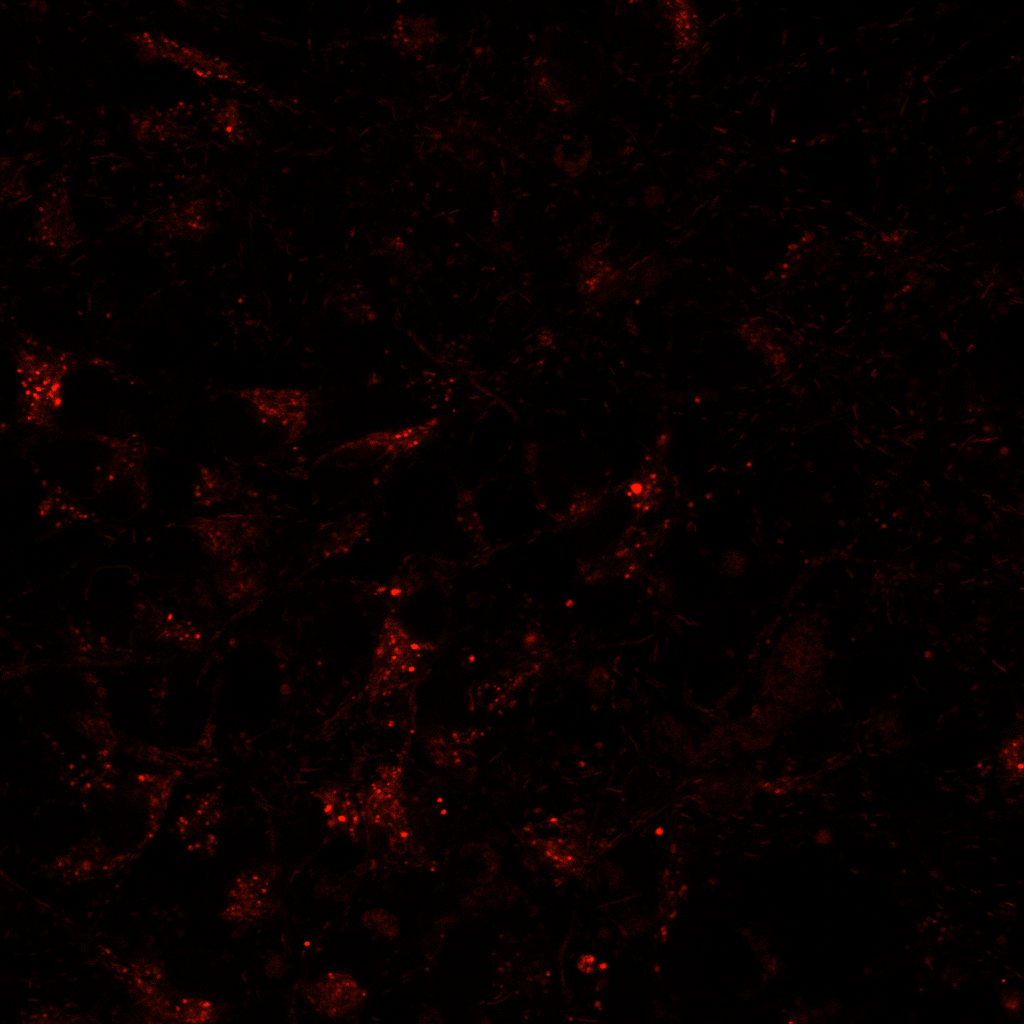

Supplement: Supplementary file 8 — Source data Fig. 4 [file 44321_2025_323_MOESM8_ESM.zip › Figure 4/4J/UC-H2-SHOW-T.tif]

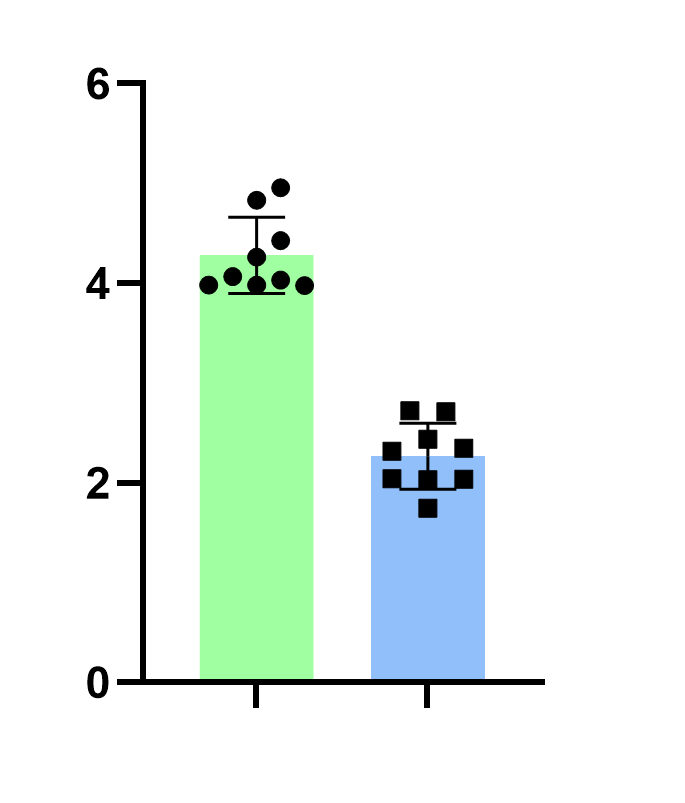

Supplement: Supplementary file 8 — Source data Fig. 4 [file 44321_2025_323_MOESM8_ESM.zip › Figure 4/4K/DOJINDO Mitophagy.tif]

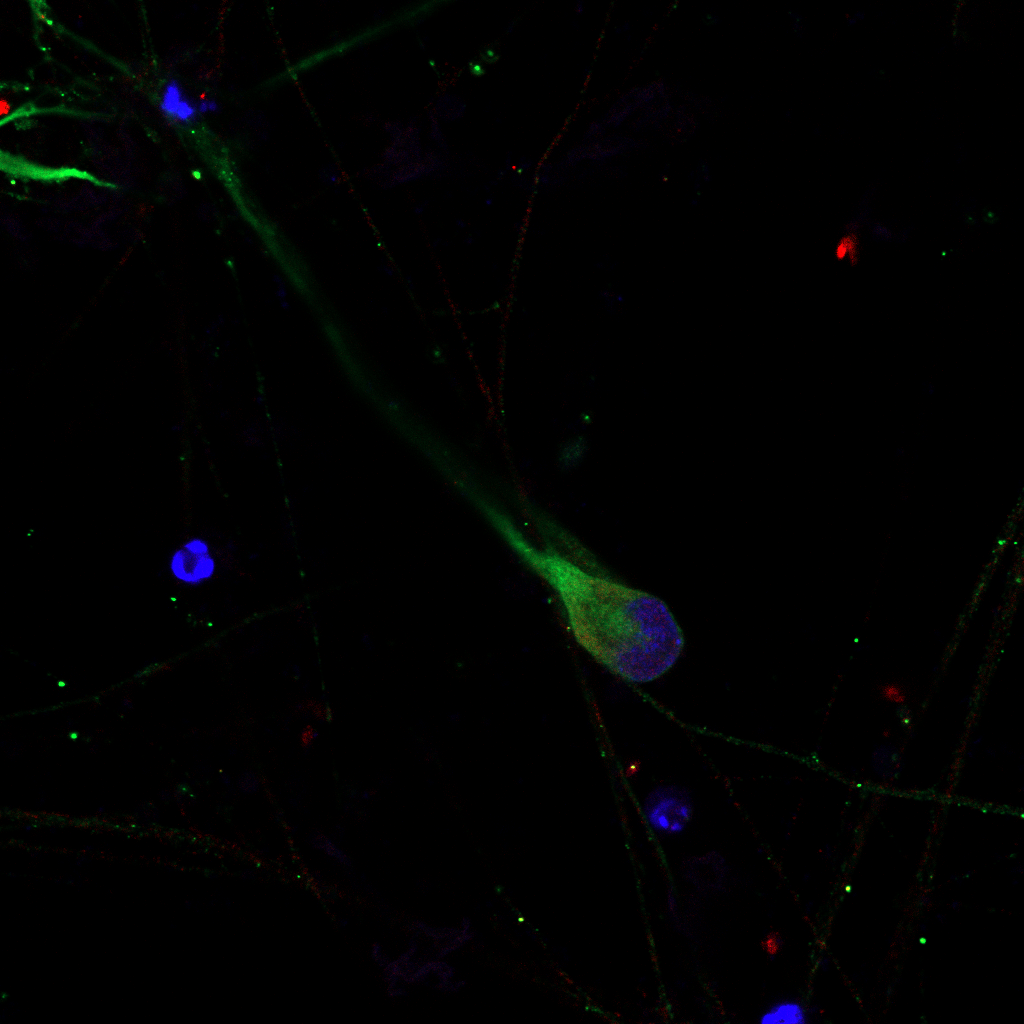

Supplement: Supplementary file 8 — Source data Fig. 4 [file 44321_2025_323_MOESM8_ESM.zip › Figure 4/4L/C9-Merge.tif]

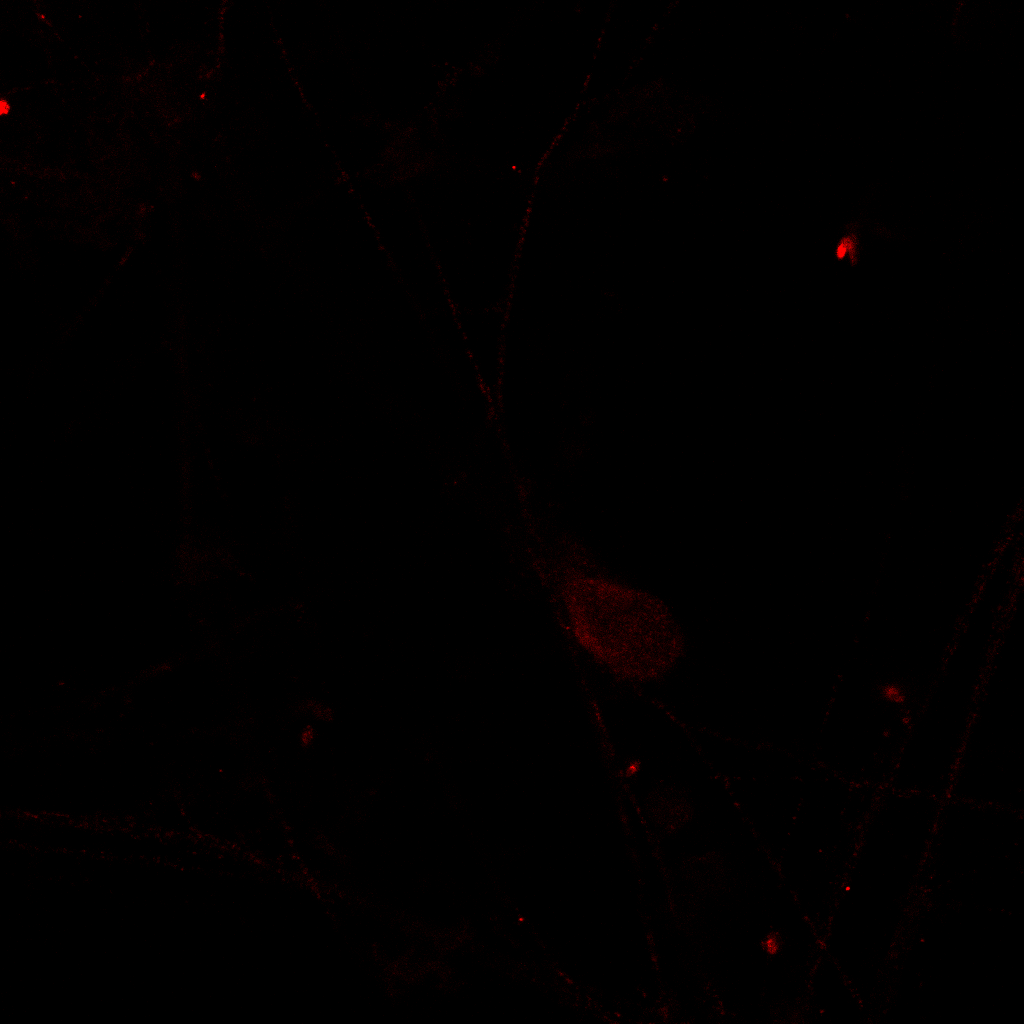

Supplement: Supplementary file 8 — Source data Fig. 4 [file 44321_2025_323_MOESM8_ESM.zip › Figure 4/4L/C9-pUb.tif]

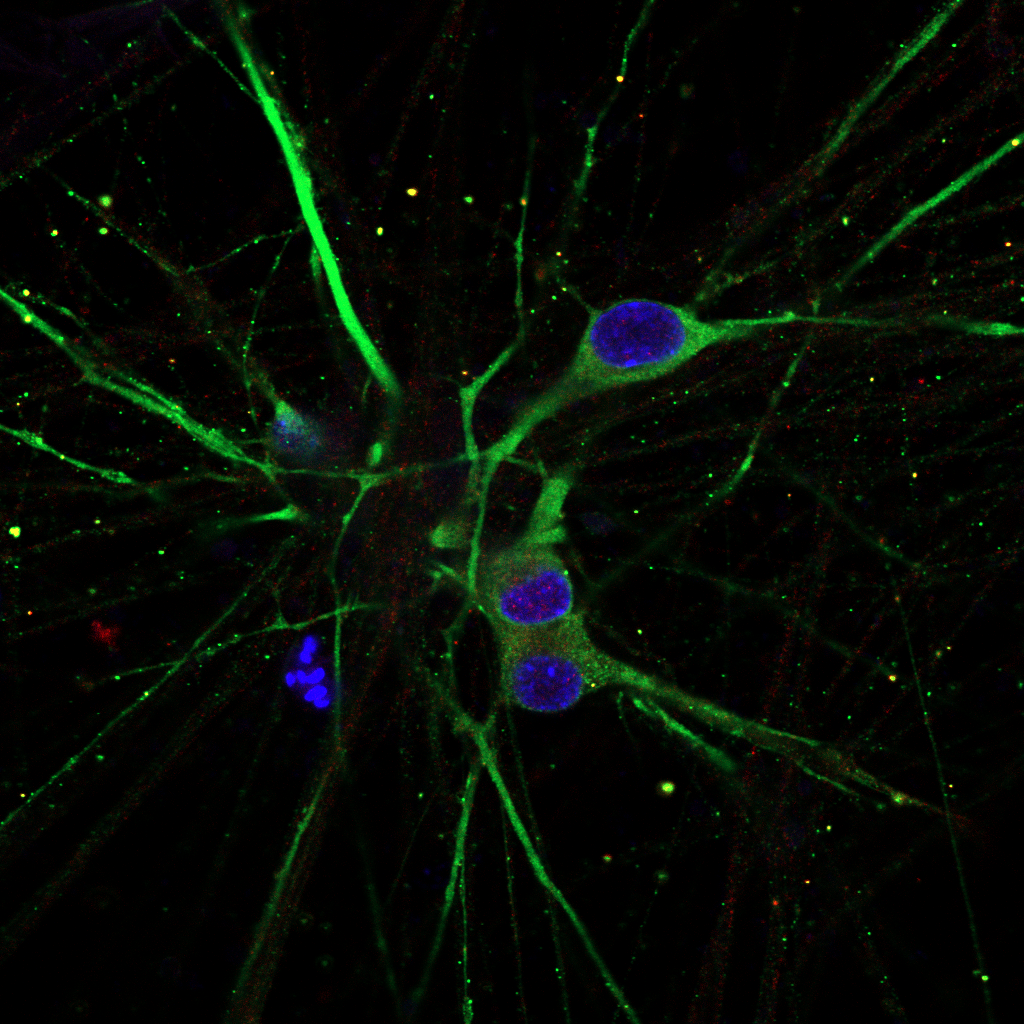

Supplement: Supplementary file 8 — Source data Fig. 4 [file 44321_2025_323_MOESM8_ESM.zip › Figure 4/4L/SOD1-Merge.tif]

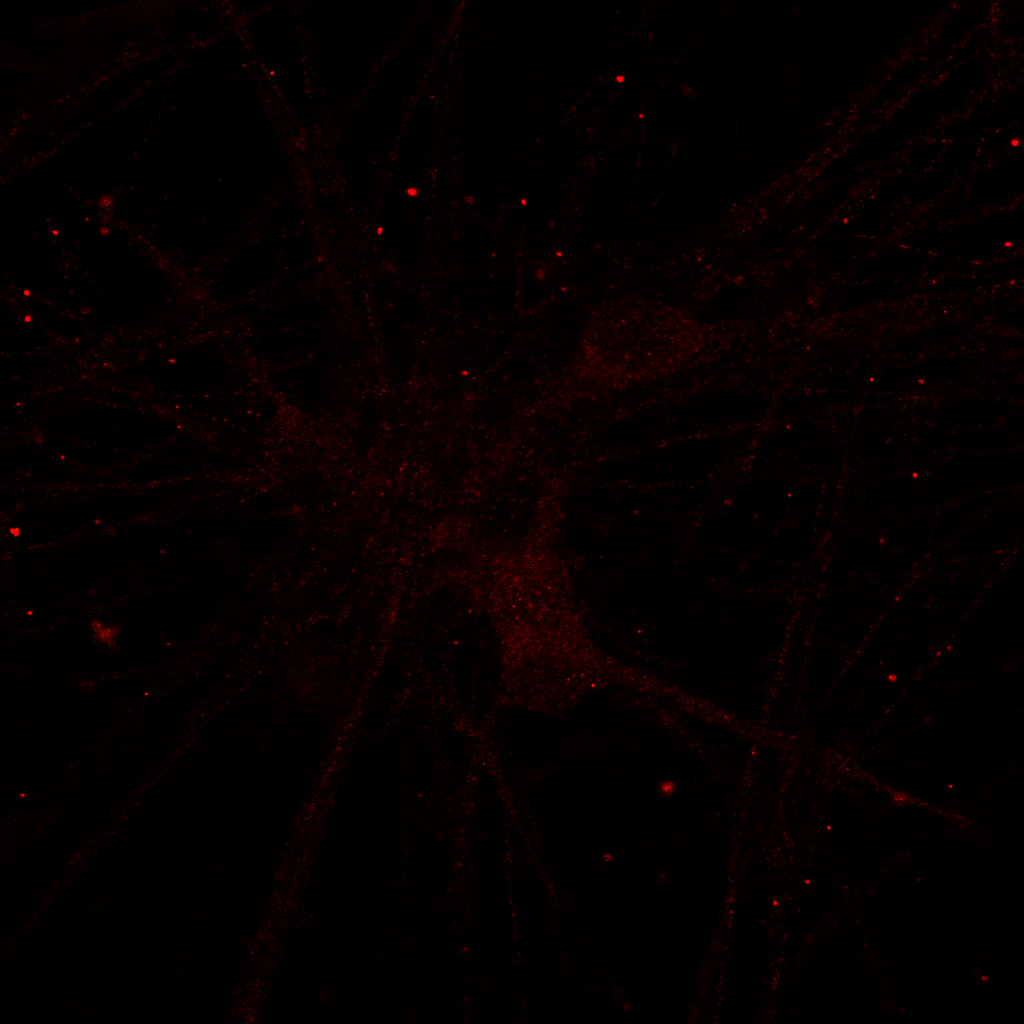

Supplement: Supplementary file 8 — Source data Fig. 4 [file 44321_2025_323_MOESM8_ESM.zip › Figure 4/4L/SOD1-pUb.tif]

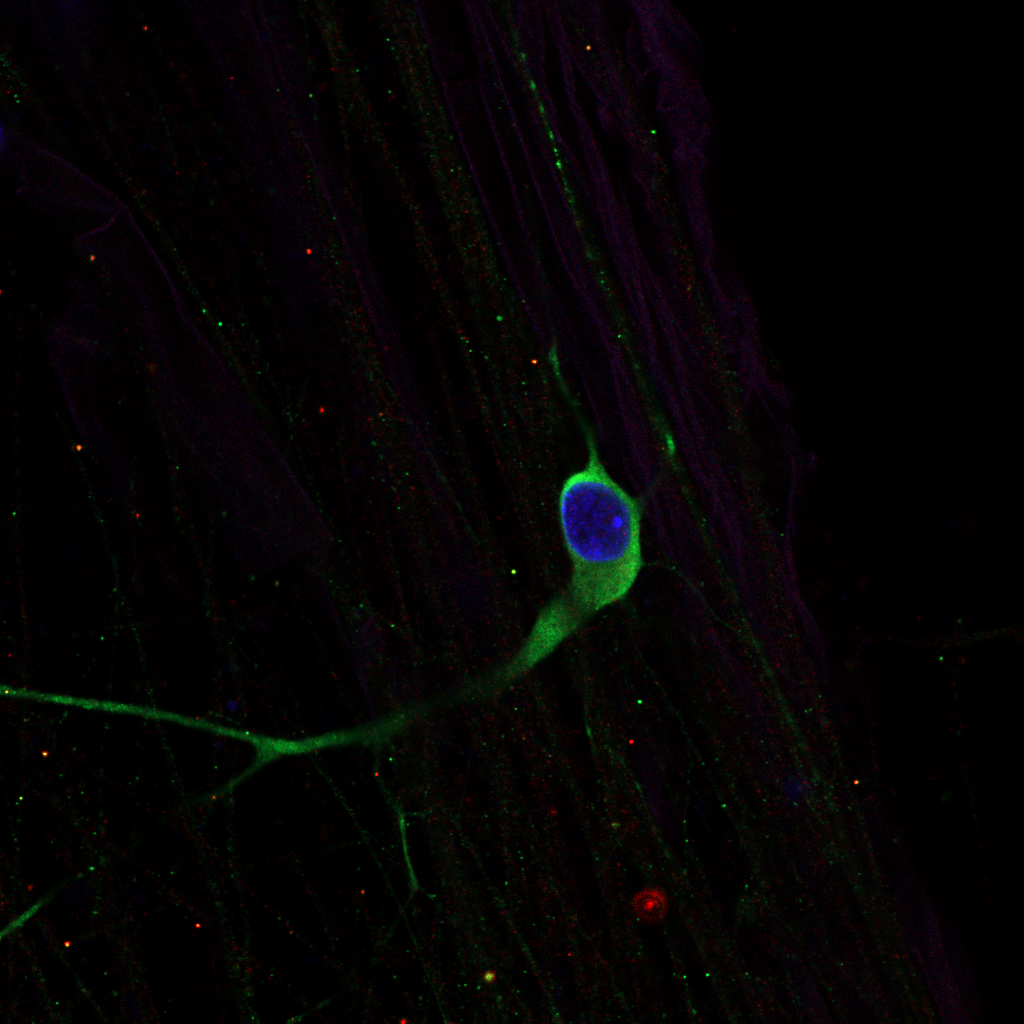

Supplement: Supplementary file 8 — Source data Fig. 4 [file 44321_2025_323_MOESM8_ESM.zip › Figure 4/4L/TDP43-Merge.tif]

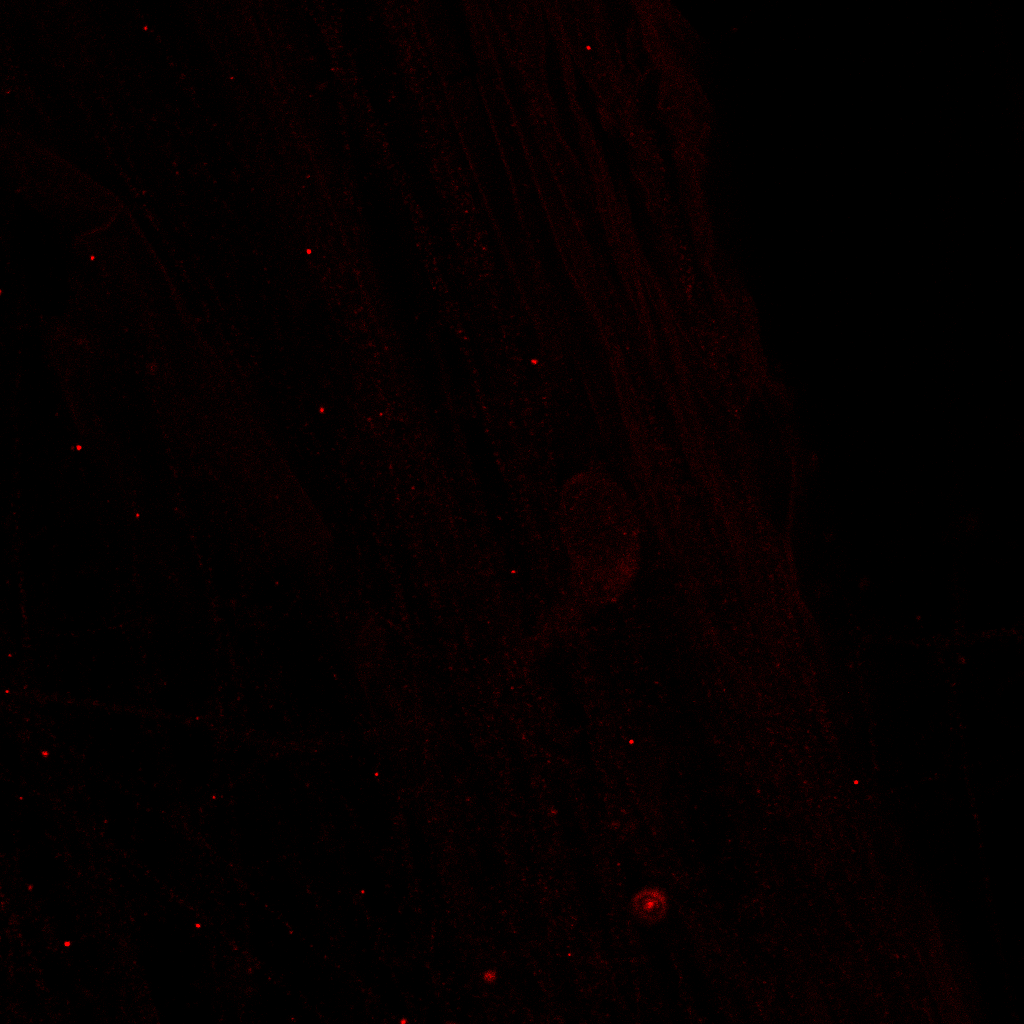

Supplement: Supplementary file 8 — Source data Fig. 4 [file 44321_2025_323_MOESM8_ESM.zip › Figure 4/4L/TDP43-pUb.tif]

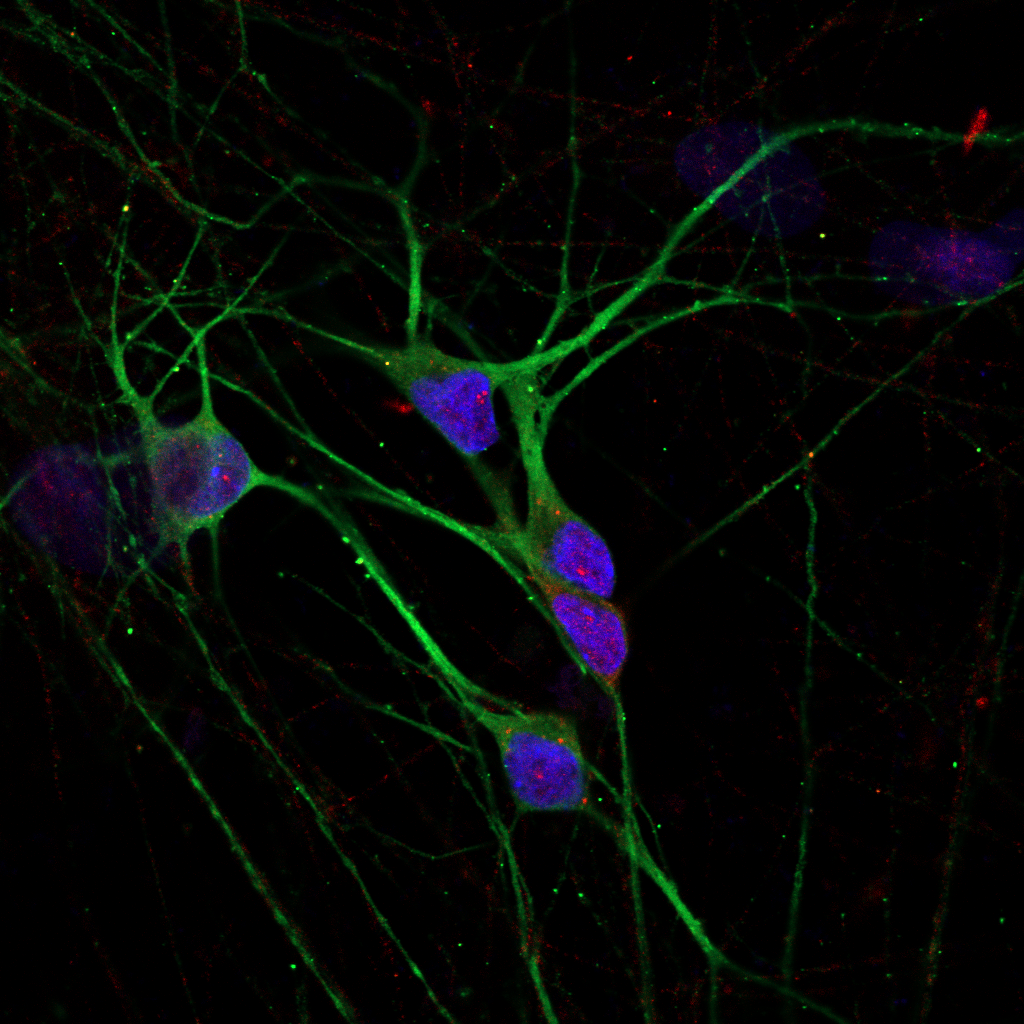

Supplement: Supplementary file 8 — Source data Fig. 4 [file 44321_2025_323_MOESM8_ESM.zip › Figure 4/4L/UC-12-Merge.tif]

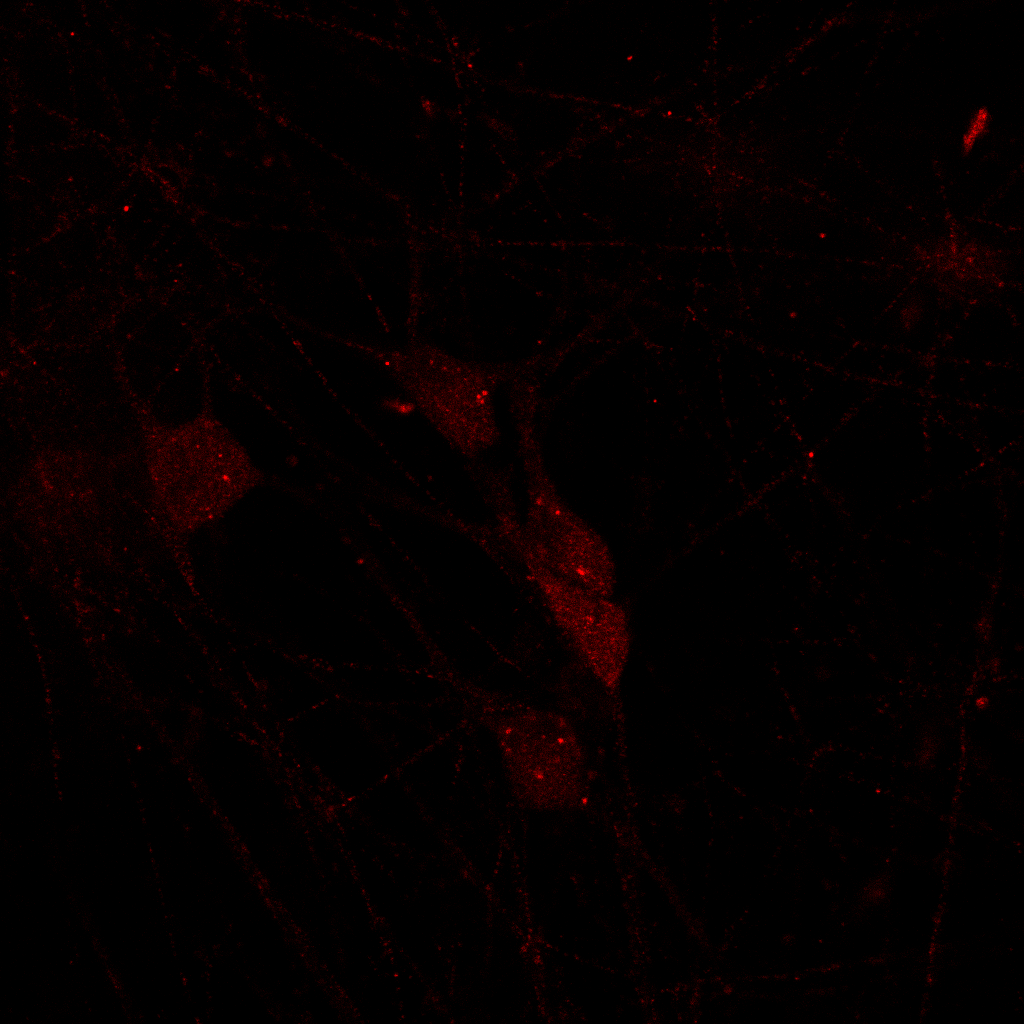

Supplement: Supplementary file 8 — Source data Fig. 4 [file 44321_2025_323_MOESM8_ESM.zip › Figure 4/4L/UC-12-pUb.tif]

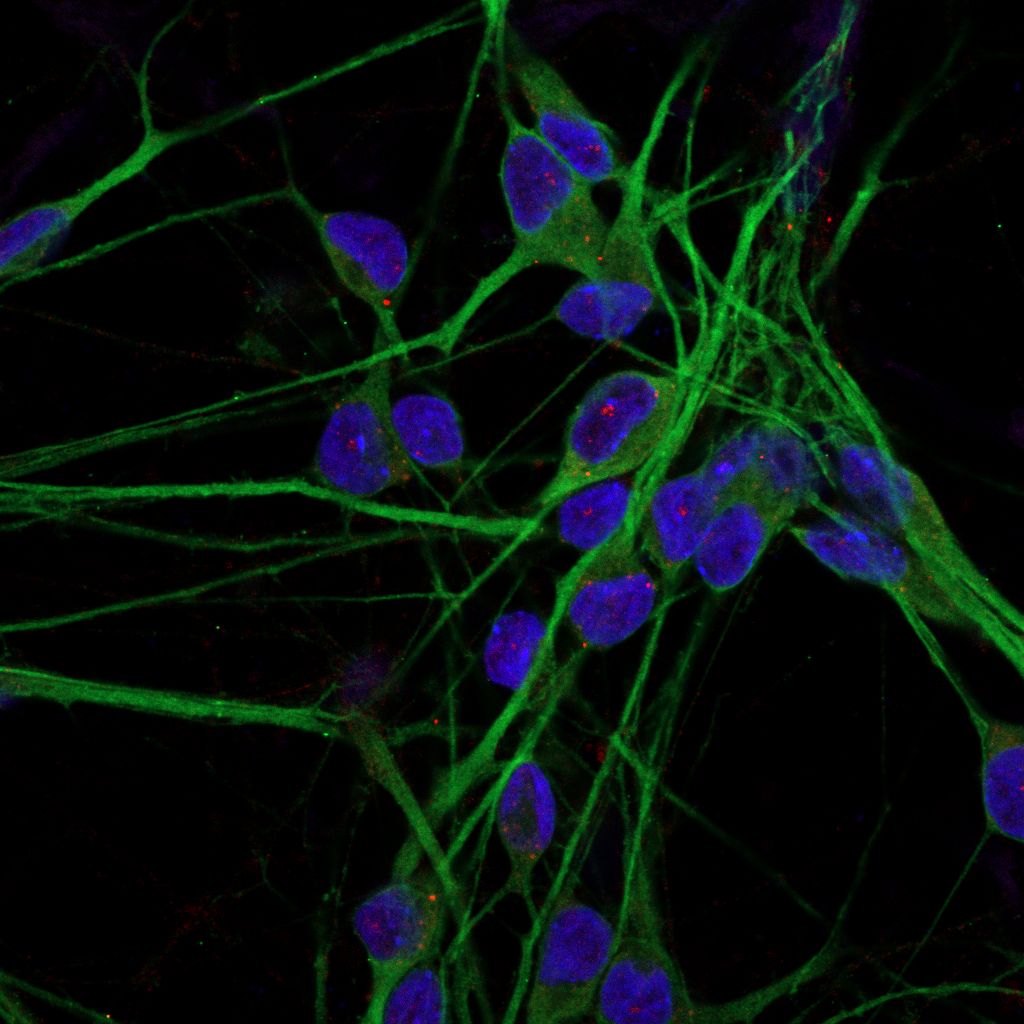

Supplement: Supplementary file 8 — Source data Fig. 4 [file 44321_2025_323_MOESM8_ESM.zip › Figure 4/4L/UC-H1-Merge.tif]

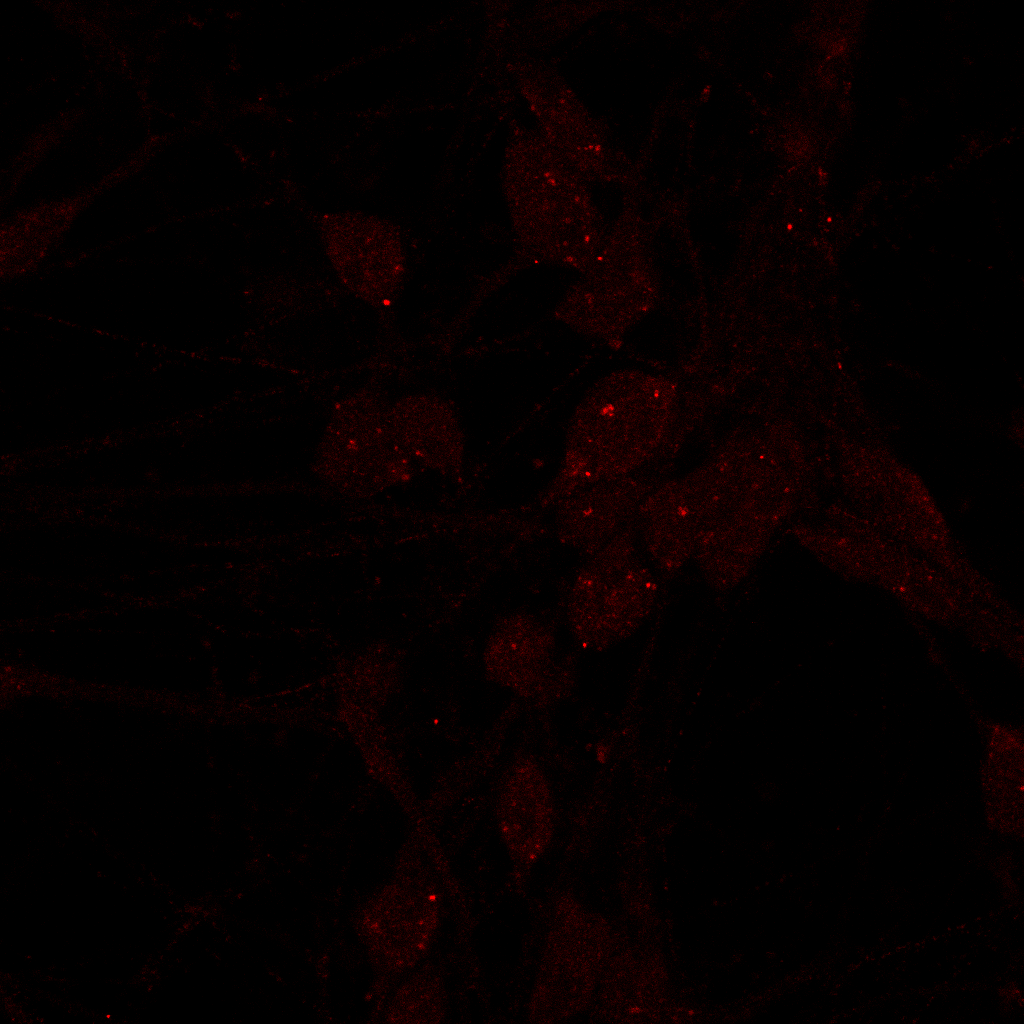

Supplement: Supplementary file 8 — Source data Fig. 4 [file 44321_2025_323_MOESM8_ESM.zip › Figure 4/4L/UC-H1-pUb.tif]

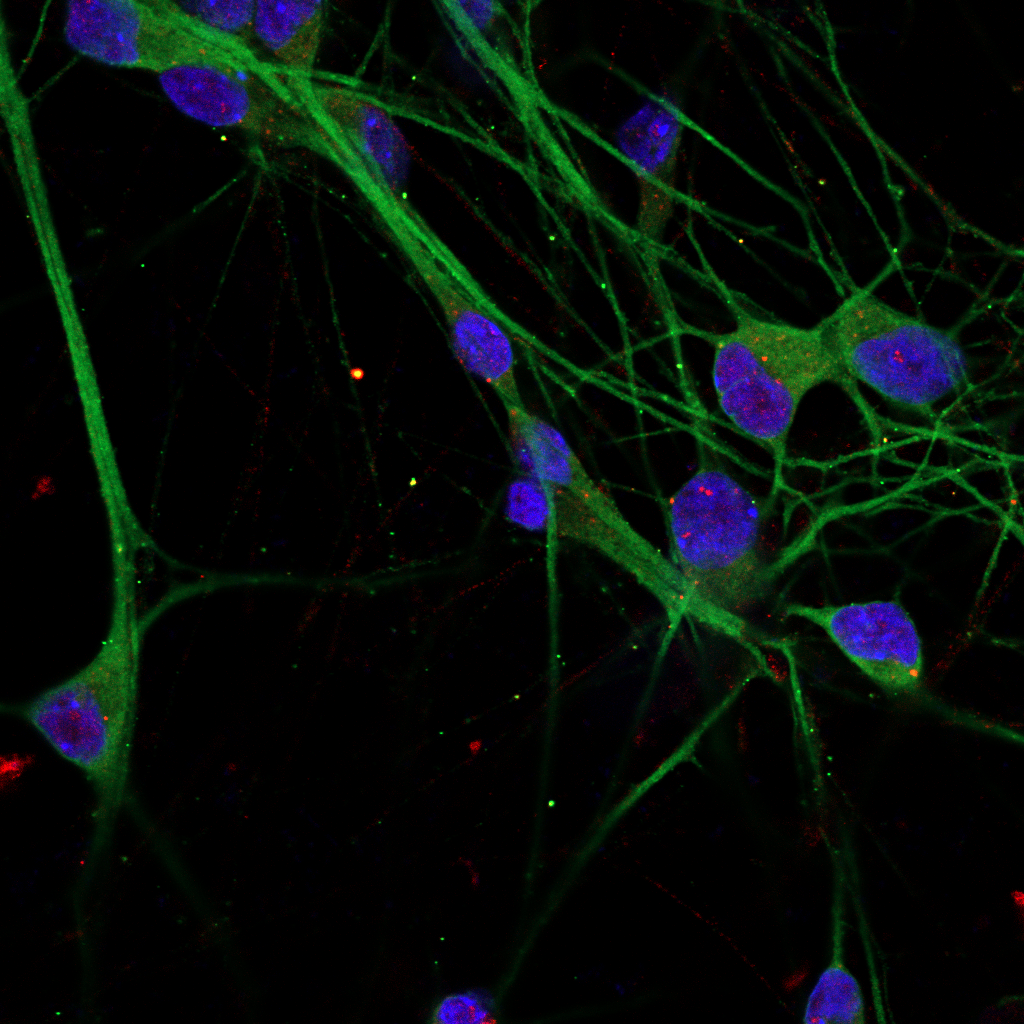

Supplement: Supplementary file 8 — Source data Fig. 4 [file 44321_2025_323_MOESM8_ESM.zip › Figure 4/4L/UC-H2-Merge.tif]

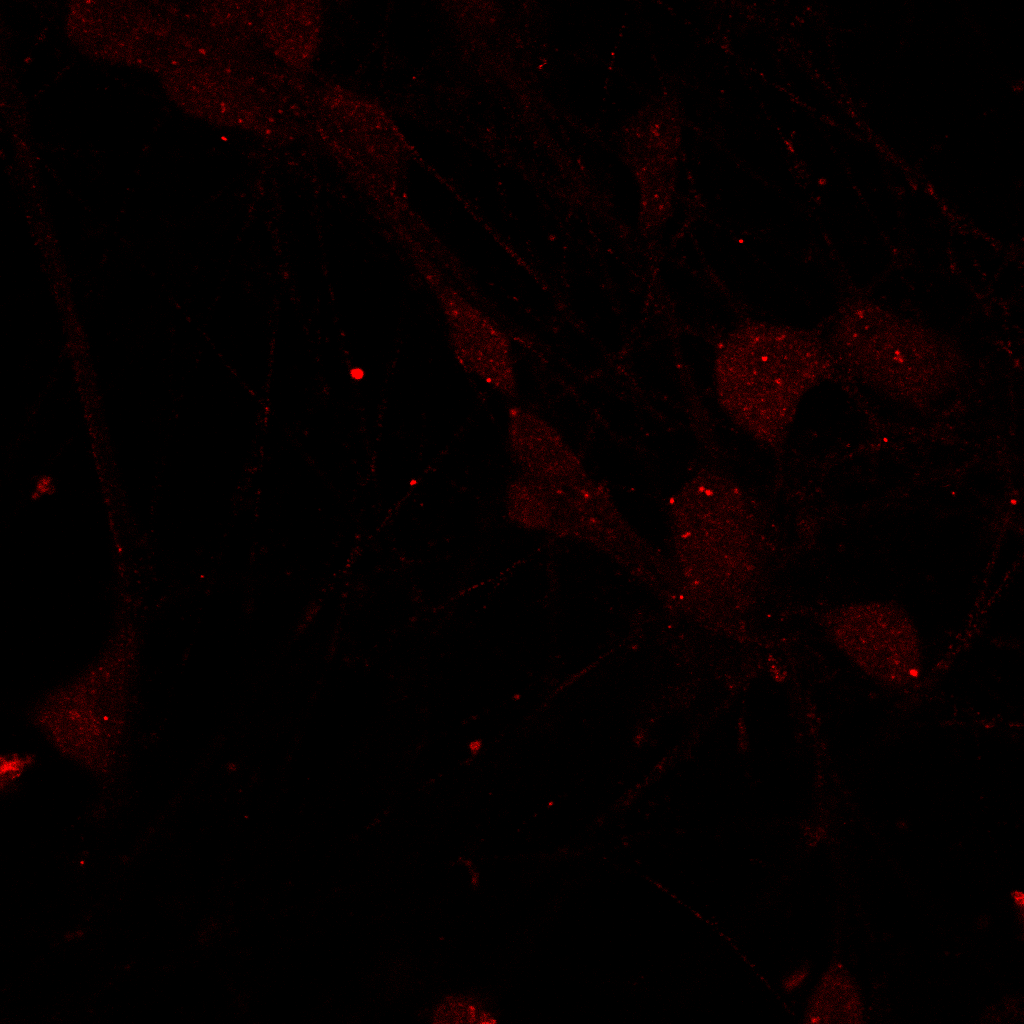

Supplement: Supplementary file 8 — Source data Fig. 4 [file 44321_2025_323_MOESM8_ESM.zip › Figure 4/4L/UC-H2-pUb.tif]

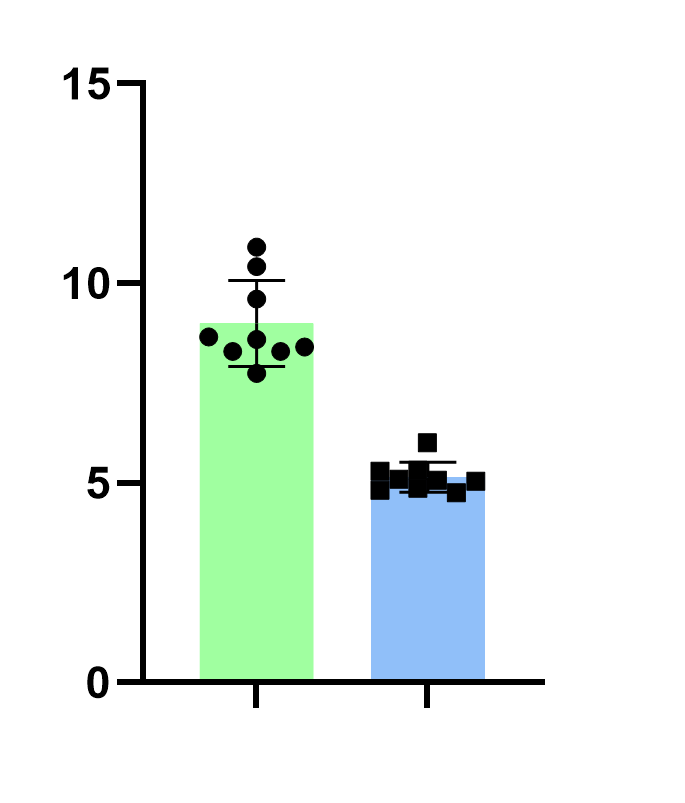

Supplement: Supplementary file 8 — Source data Fig. 4 [file 44321_2025_323_MOESM8_ESM.zip › Figure 4/4M/pUb.tif]

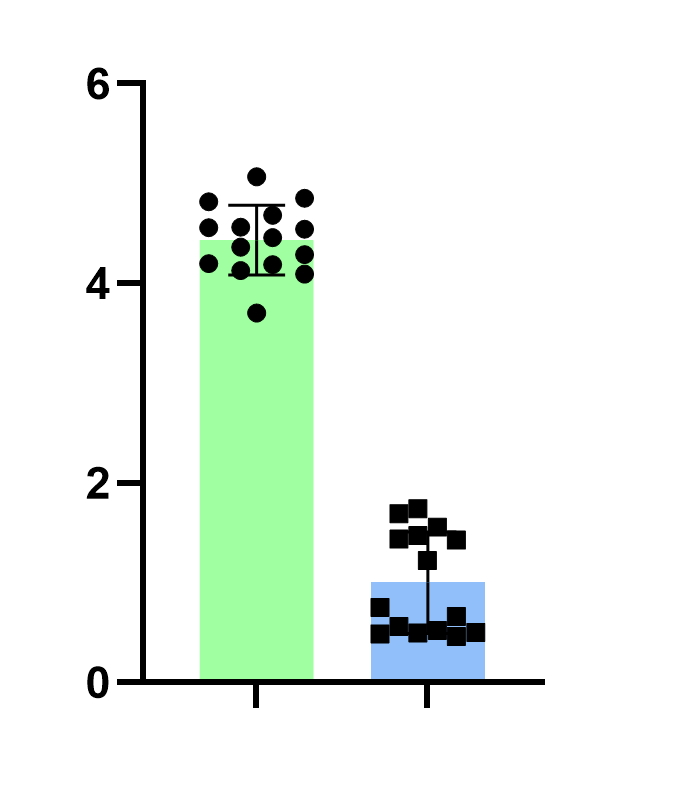

Supplement: Supplementary file 8 — Source data Fig. 4 [file 44321_2025_323_MOESM8_ESM.zip › Figure 4/4N/ATP.tif]

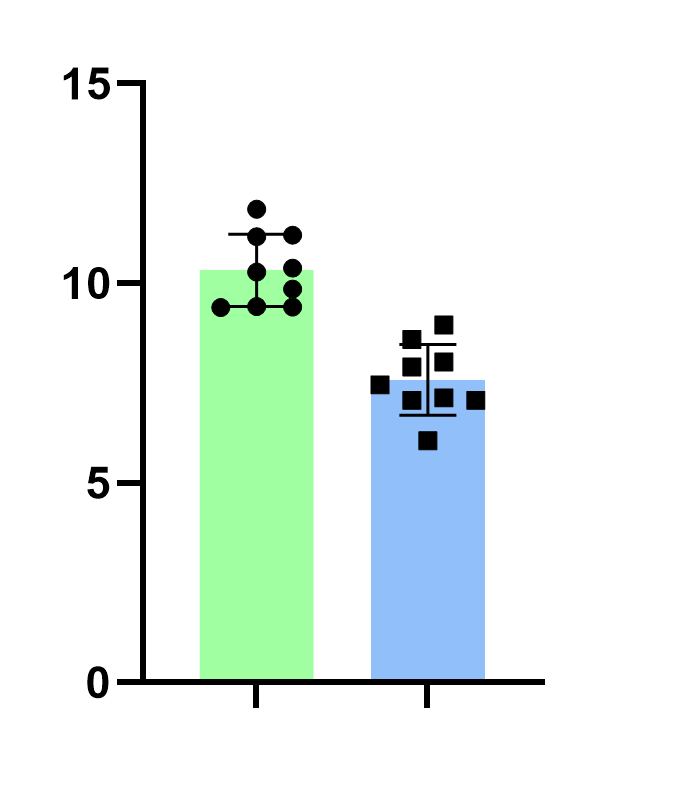

Supplement: Supplementary file 8 — Source data Fig. 4 [file 44321_2025_323_MOESM8_ESM.zip › Figure 4/4O/MMP.tif]

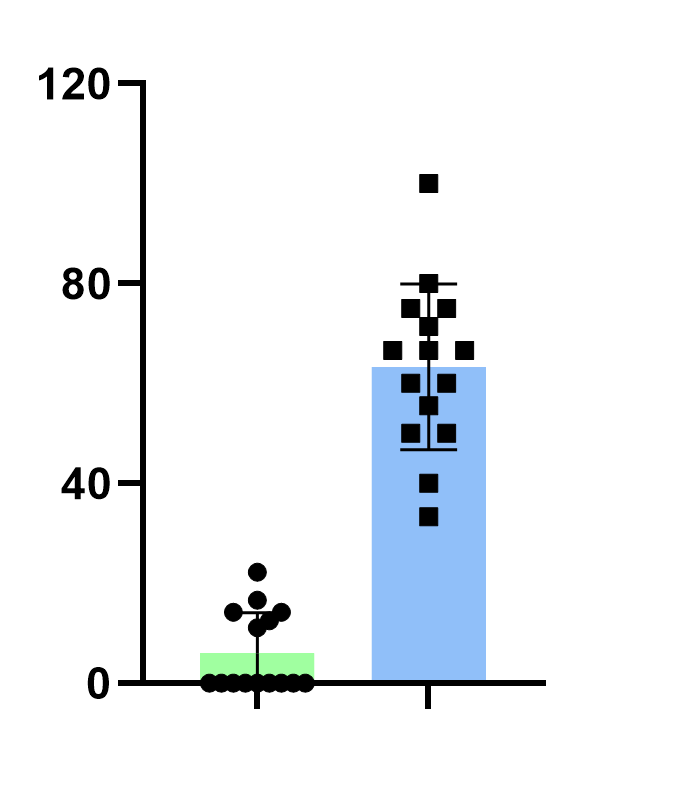

Supplement: Supplementary file 8 — Source data Fig. 4 [file 44321_2025_323_MOESM8_ESM.zip › Figure 4/4Q/%damaged mitochondria.tif]

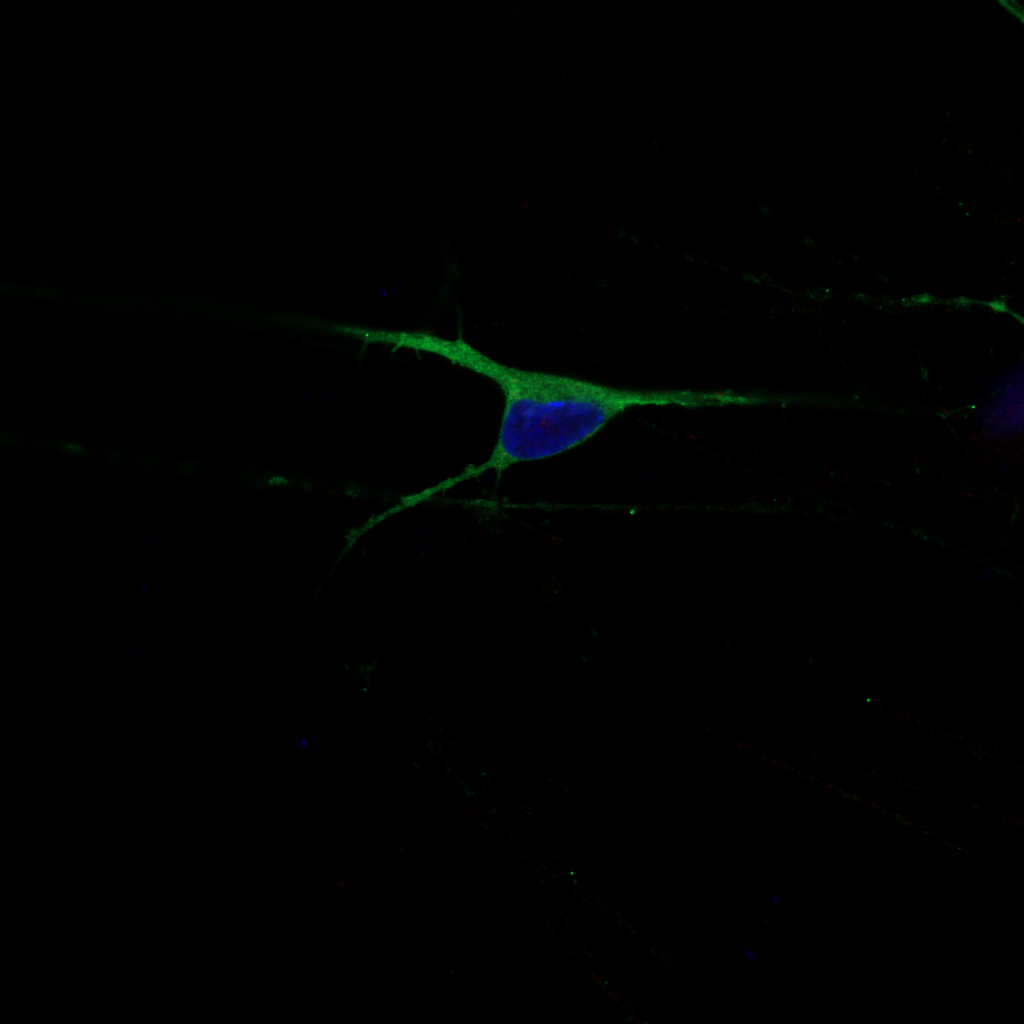

Supplement: Supplementary file 9 — Source data Fig. 5 [file 44321_2025_323_MOESM9_ESM.zip › Figure 5/5A/c9.tif]

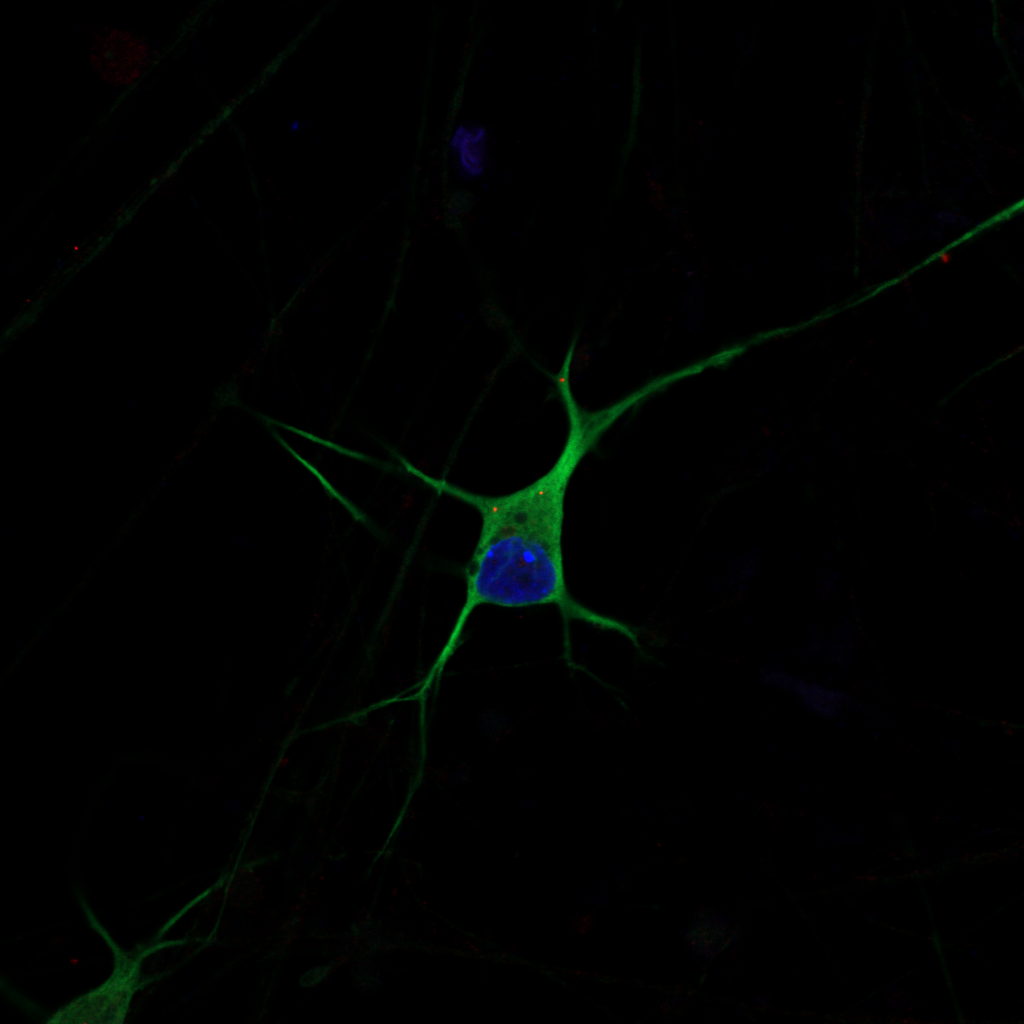

Supplement: Supplementary file 9 — Source data Fig. 5 [file 44321_2025_323_MOESM9_ESM.zip › Figure 5/5A/c9-iso.tif]

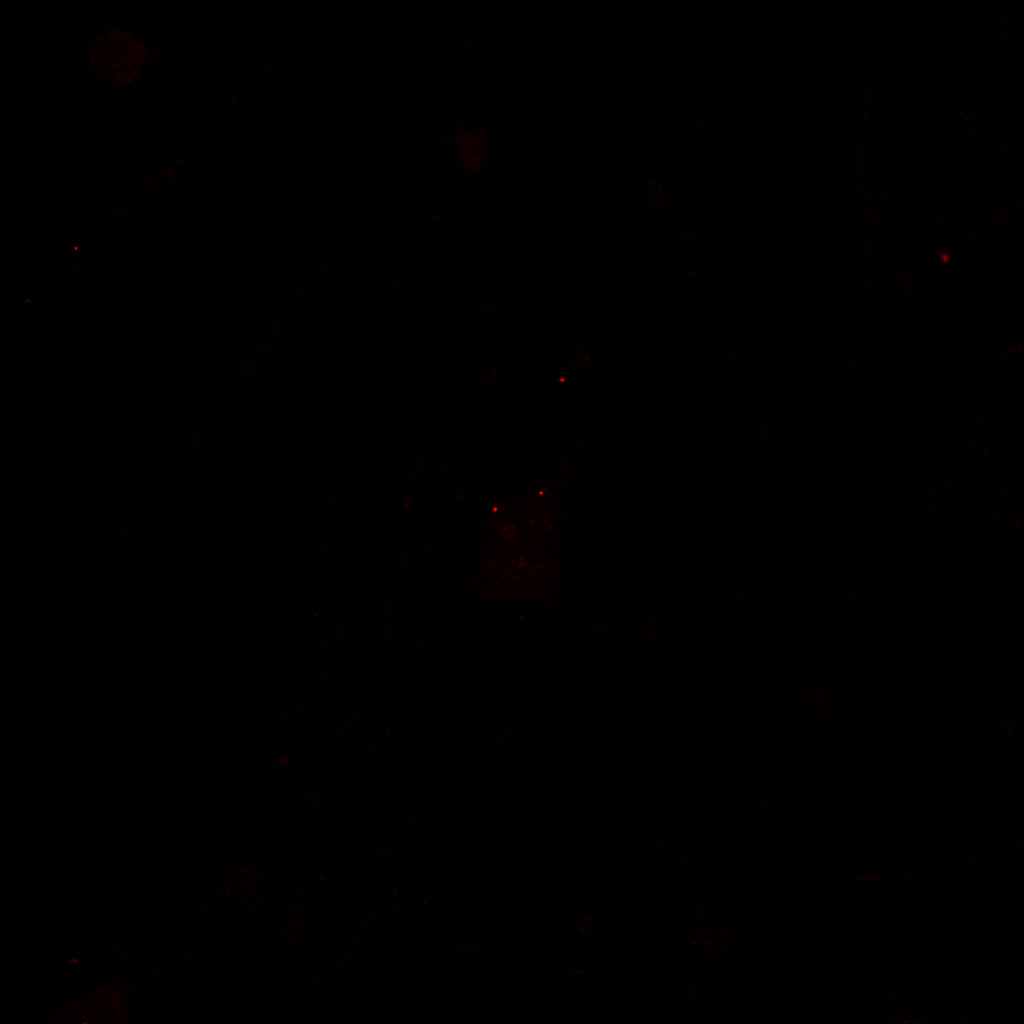

Supplement: Supplementary file 9 — Source data Fig. 5 [file 44321_2025_323_MOESM9_ESM.zip › Figure 5/5A/c9-iso-PUB.tif]

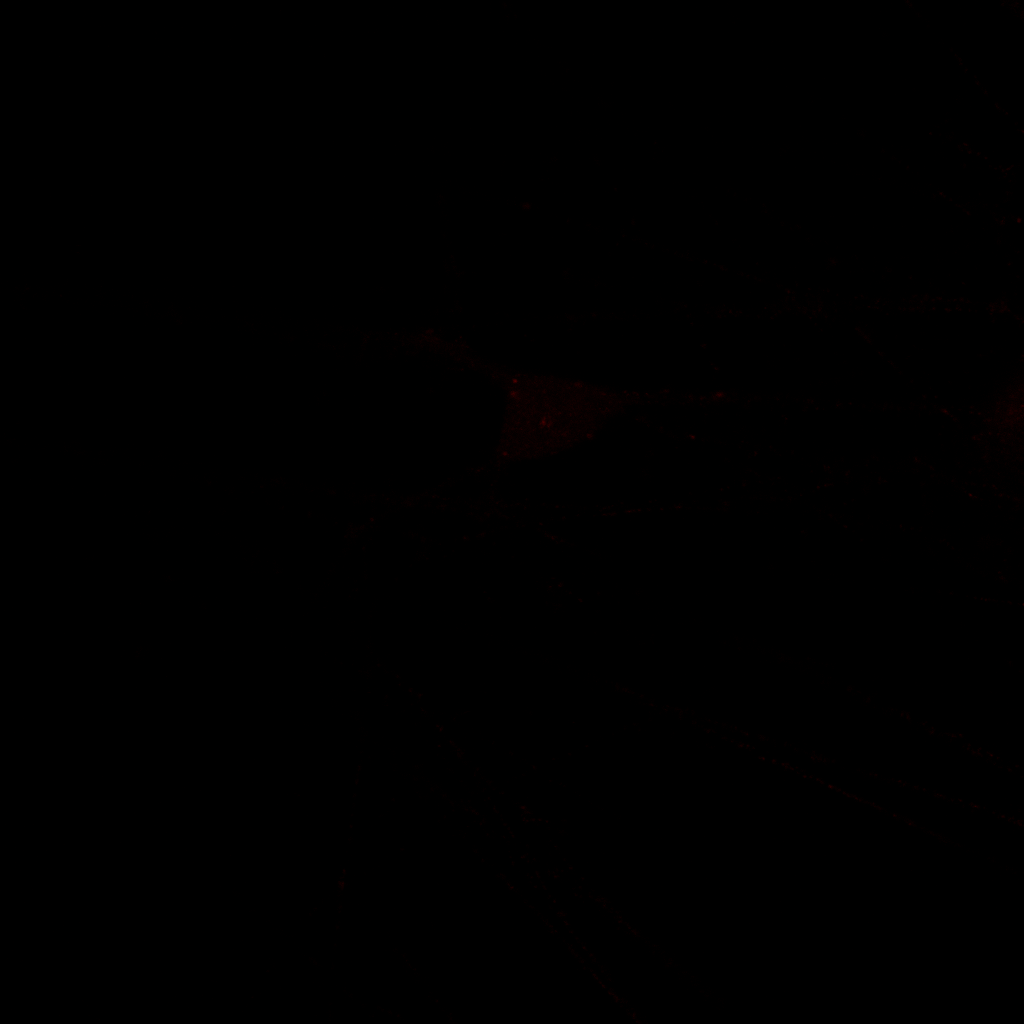

Supplement: Supplementary file 9 — Source data Fig. 5 [file 44321_2025_323_MOESM9_ESM.zip › Figure 5/5A/c9-PUB.tif]

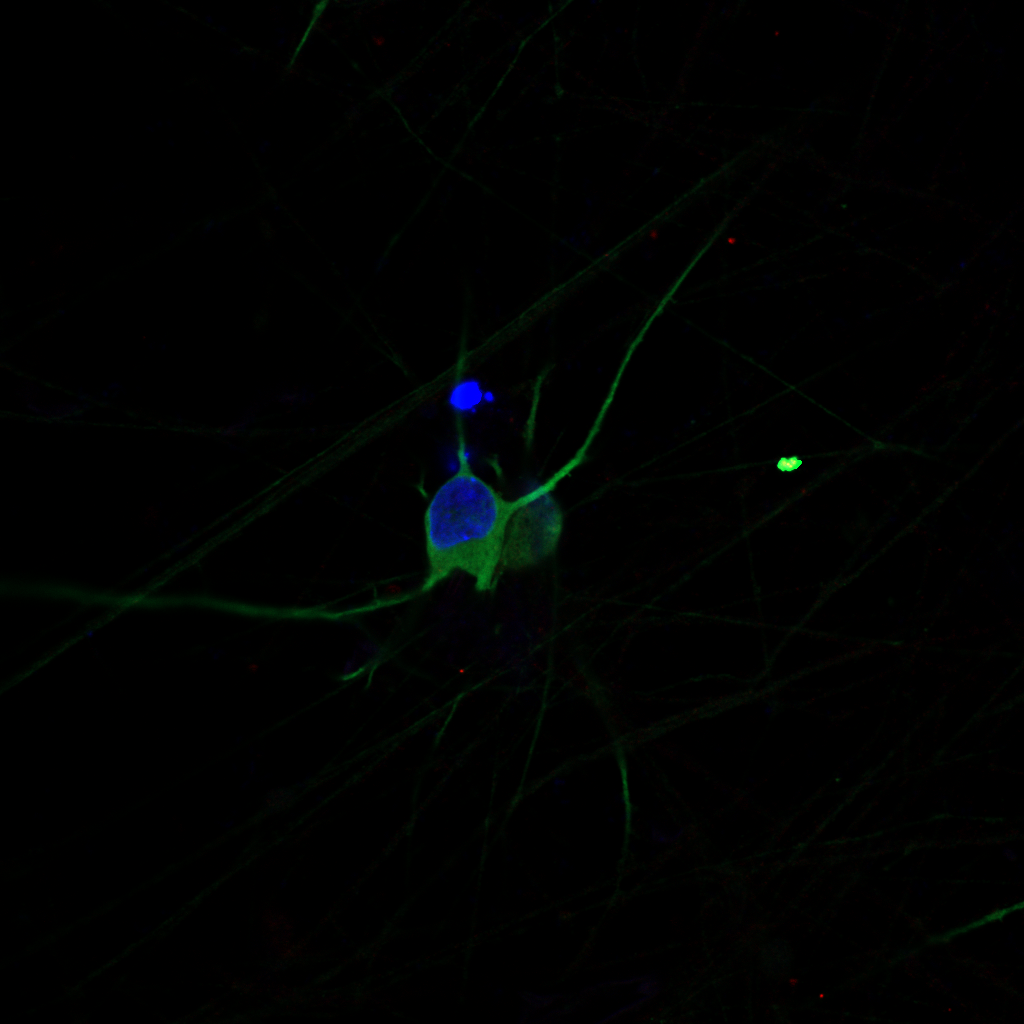

Supplement: Supplementary file 9 — Source data Fig. 5 [file 44321_2025_323_MOESM9_ESM.zip › Figure 5/5A/sod1.tif]

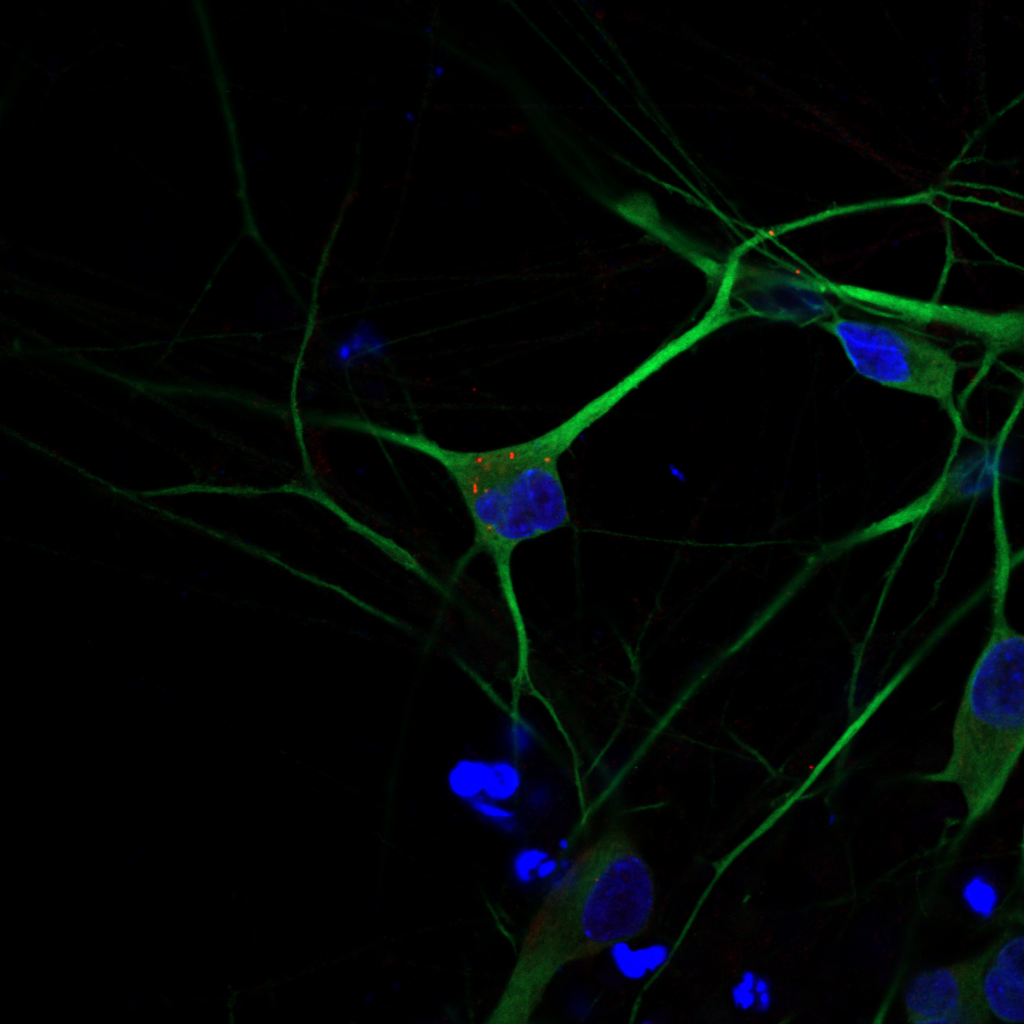

Supplement: Supplementary file 9 — Source data Fig. 5 [file 44321_2025_323_MOESM9_ESM.zip › Figure 5/5A/sod1-iso.tif]

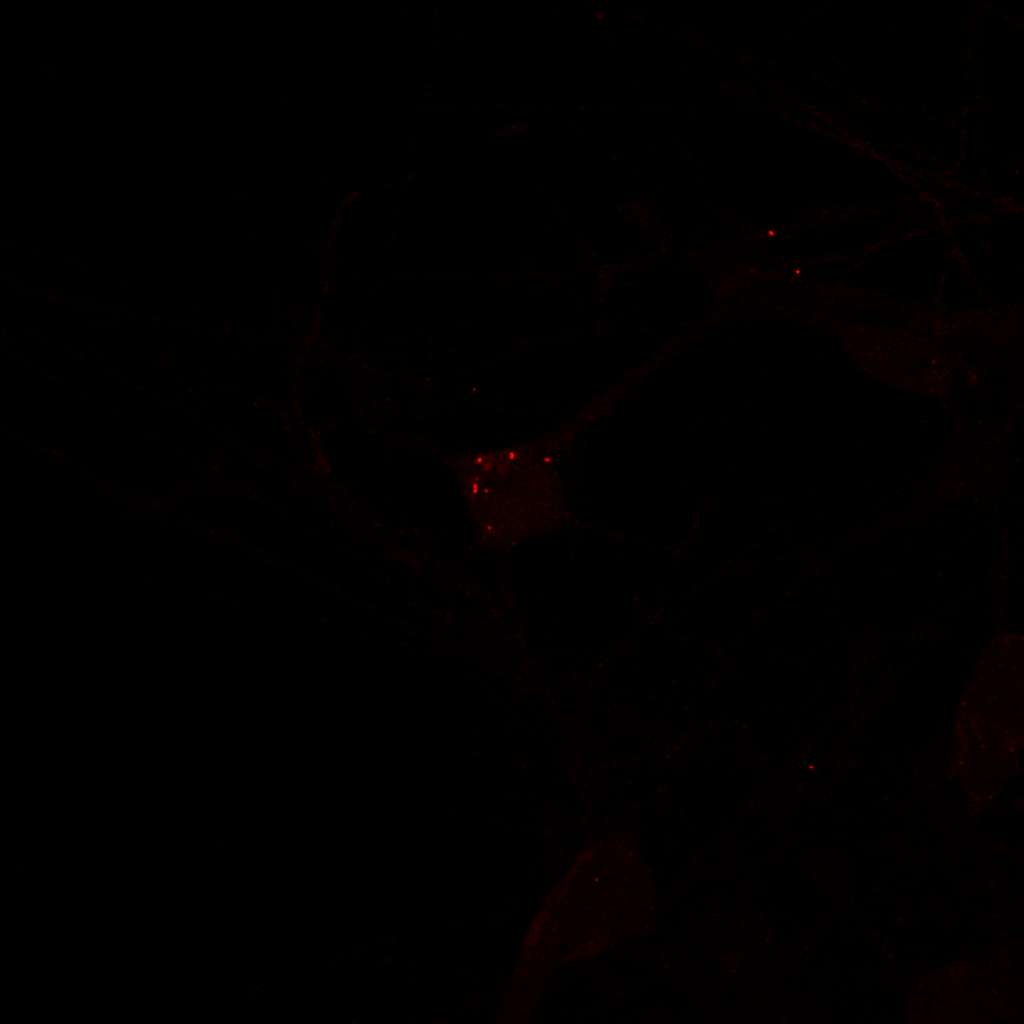

Supplement: Supplementary file 9 — Source data Fig. 5 [file 44321_2025_323_MOESM9_ESM.zip › Figure 5/5A/sod1-iso-PUB.tif]

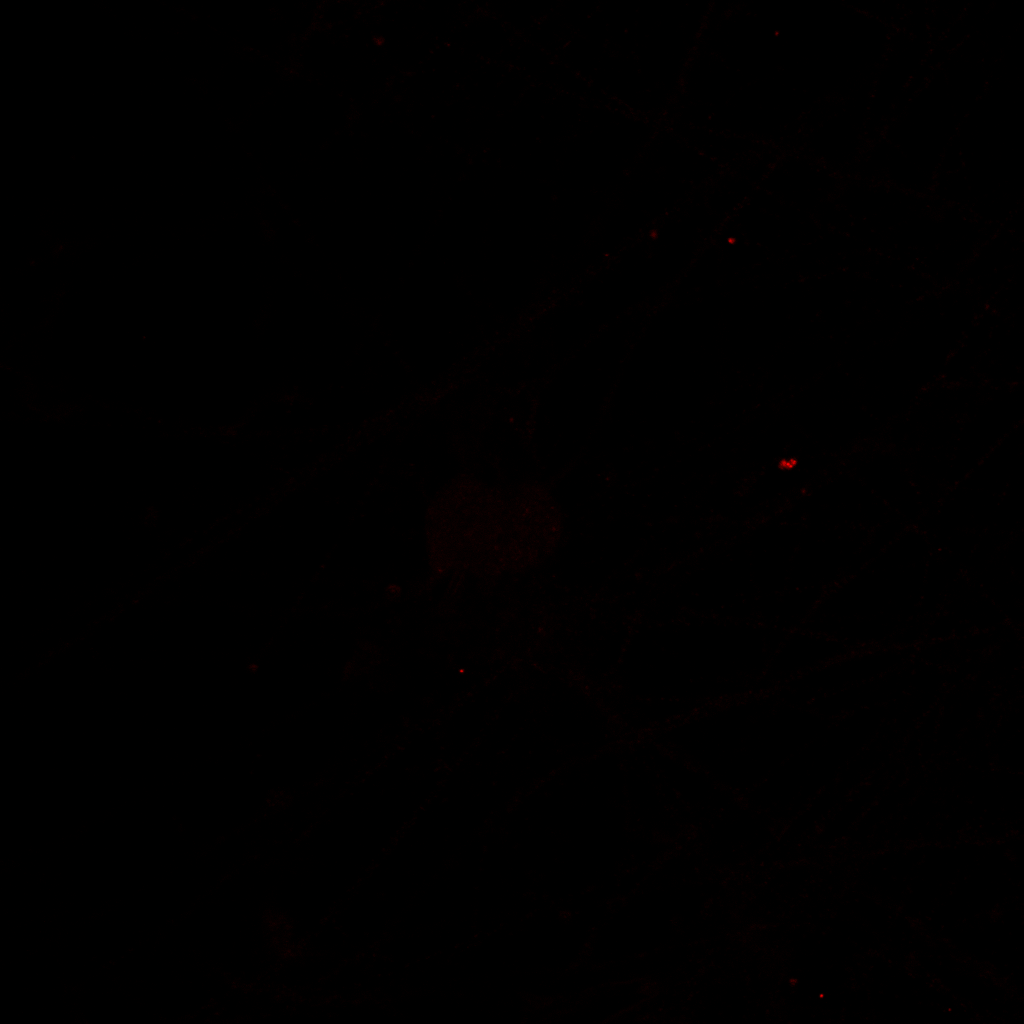

Supplement: Supplementary file 9 — Source data Fig. 5 [file 44321_2025_323_MOESM9_ESM.zip › Figure 5/5A/sod1-PUB.tif]

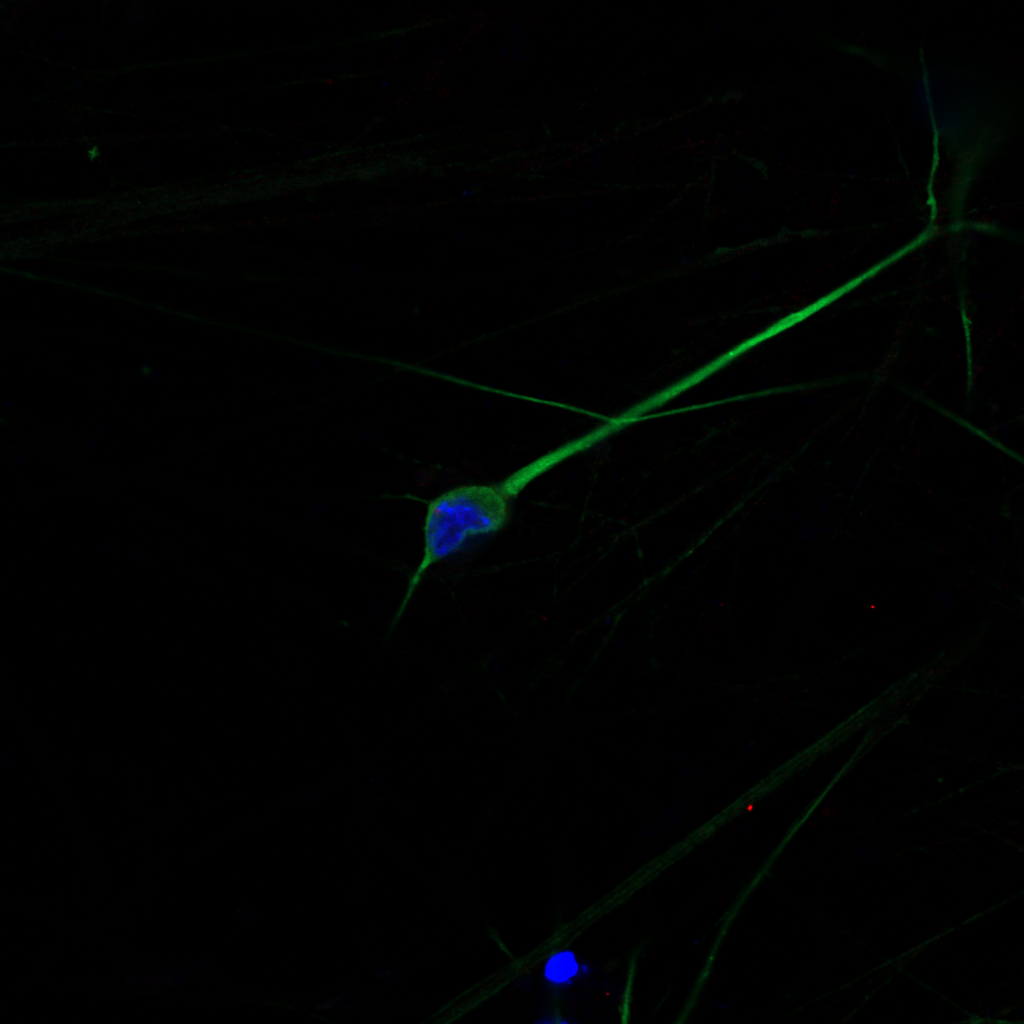

Supplement: Supplementary file 9 — Source data Fig. 5 [file 44321_2025_323_MOESM9_ESM.zip › Figure 5/5A/tdp-43.tif]

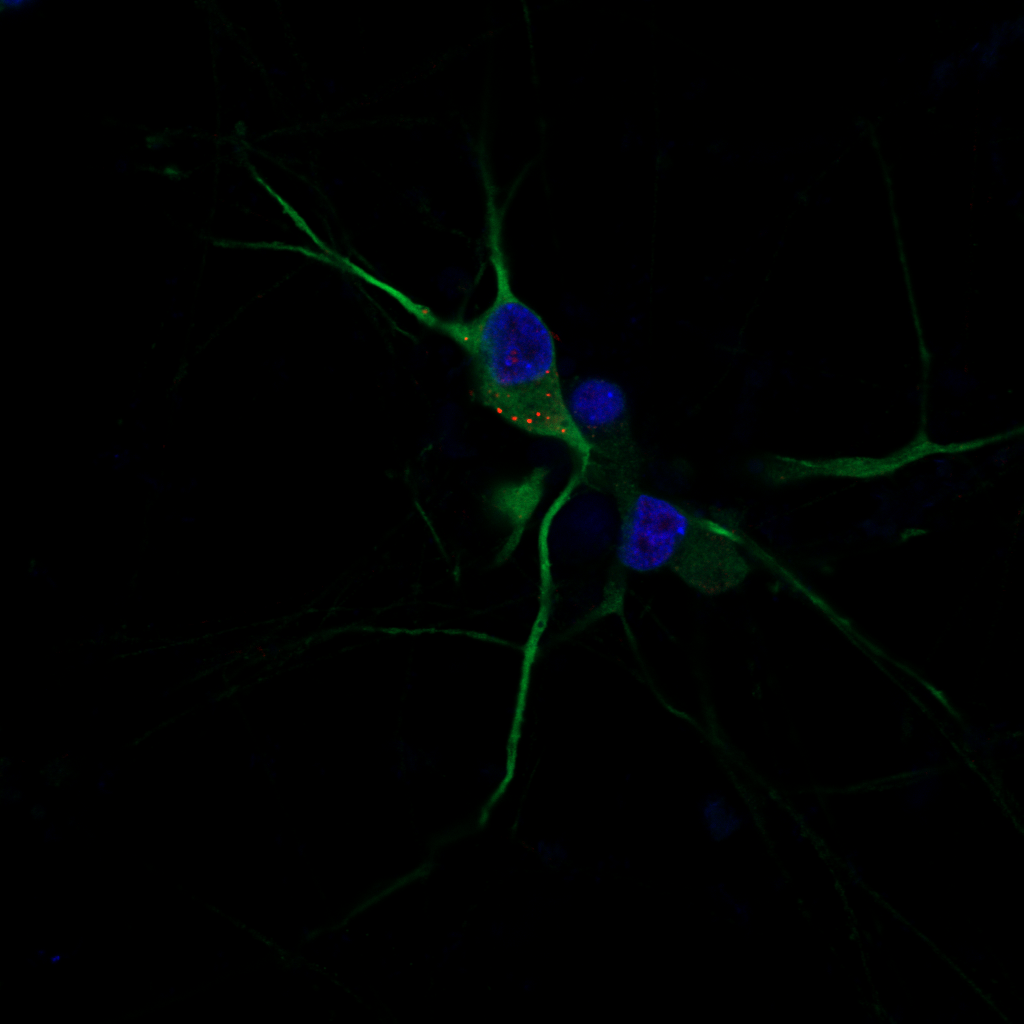

Supplement: Supplementary file 9 — Source data Fig. 5 [file 44321_2025_323_MOESM9_ESM.zip › Figure 5/5A/tdp-43-iso.tif]

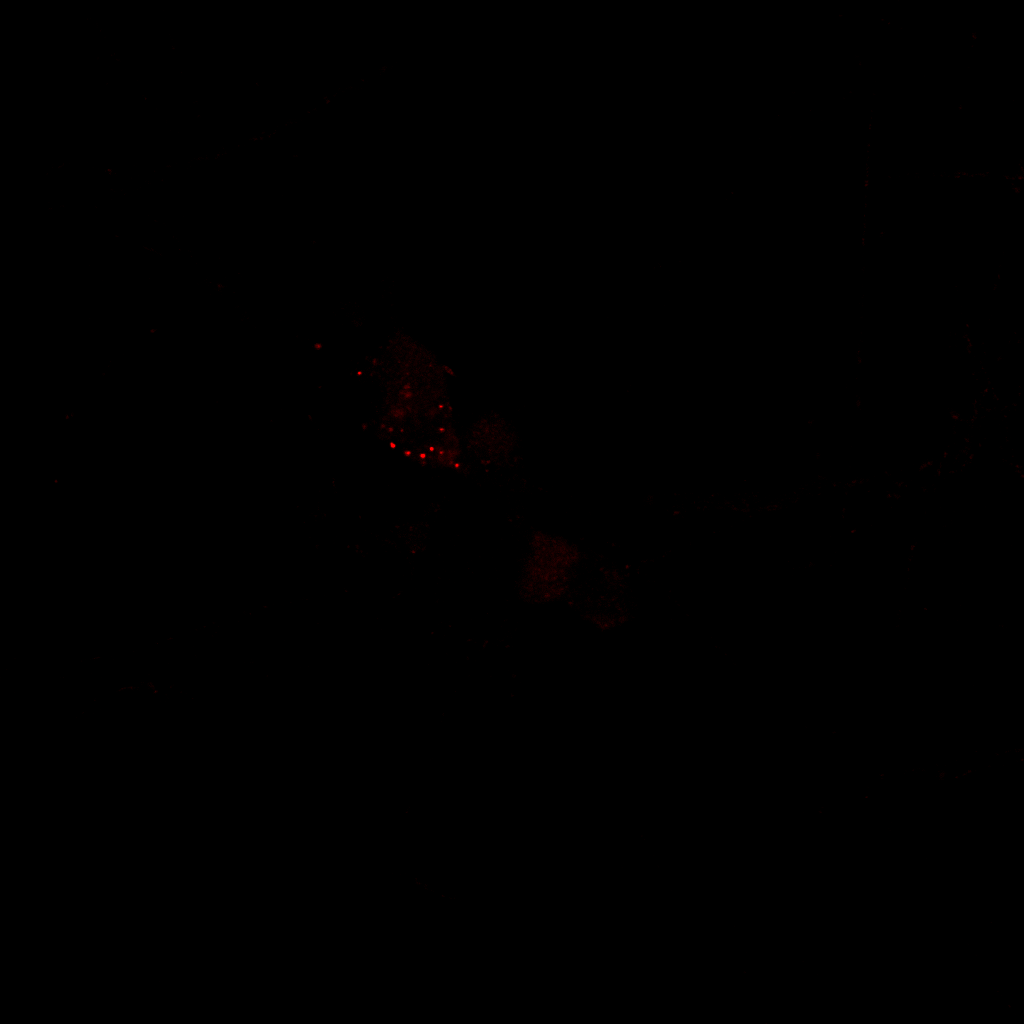

Supplement: Supplementary file 9 — Source data Fig. 5 [file 44321_2025_323_MOESM9_ESM.zip › Figure 5/5A/tdp-43-iso-PUB.tif]

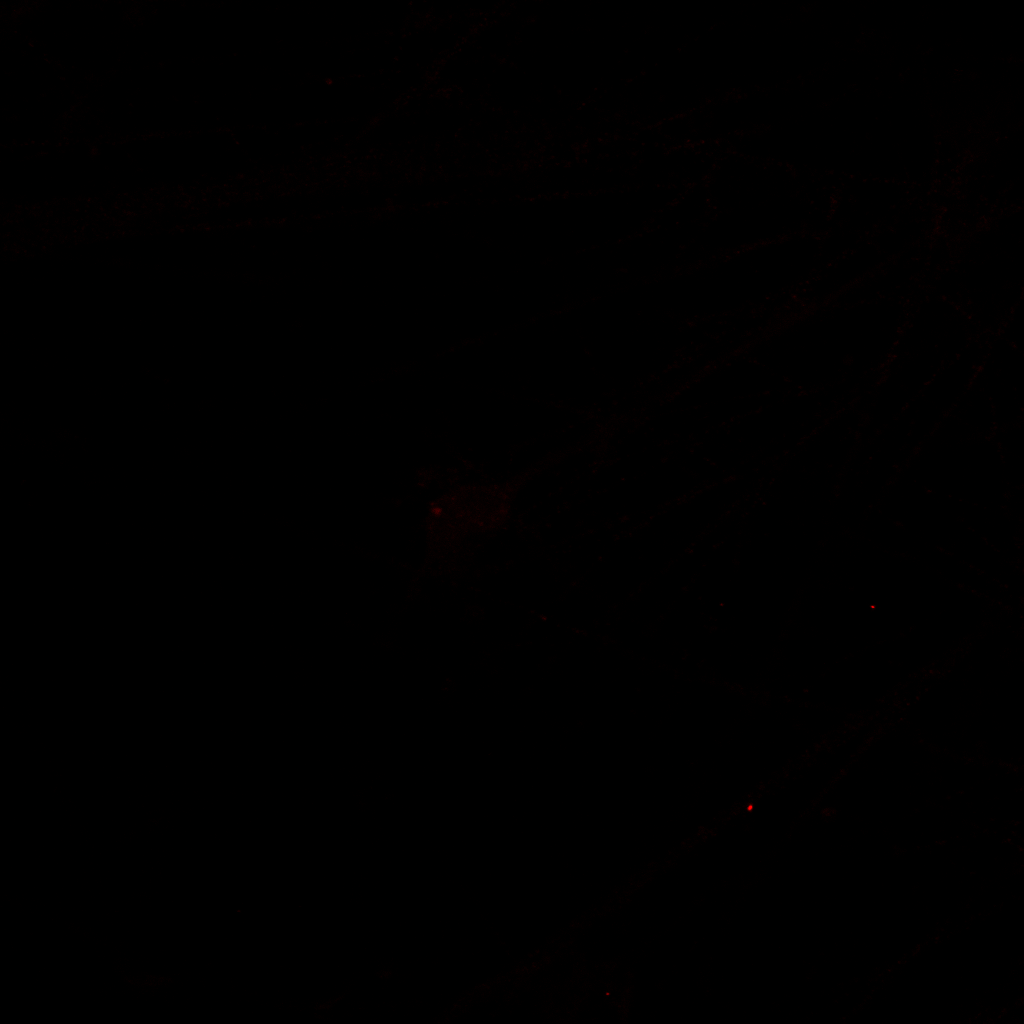

Supplement: Supplementary file 9 — Source data Fig. 5 [file 44321_2025_323_MOESM9_ESM.zip › Figure 5/5A/tdp-43-PUB.tif]

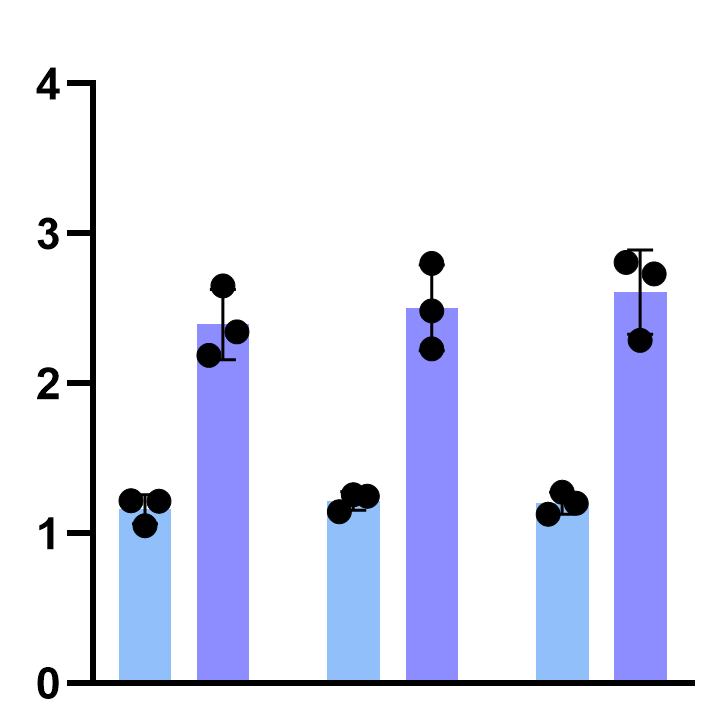

Supplement: Supplementary file 9 — Source data Fig. 5 [file 44321_2025_323_MOESM9_ESM.zip › Figure 5/5B/PUB.tif]

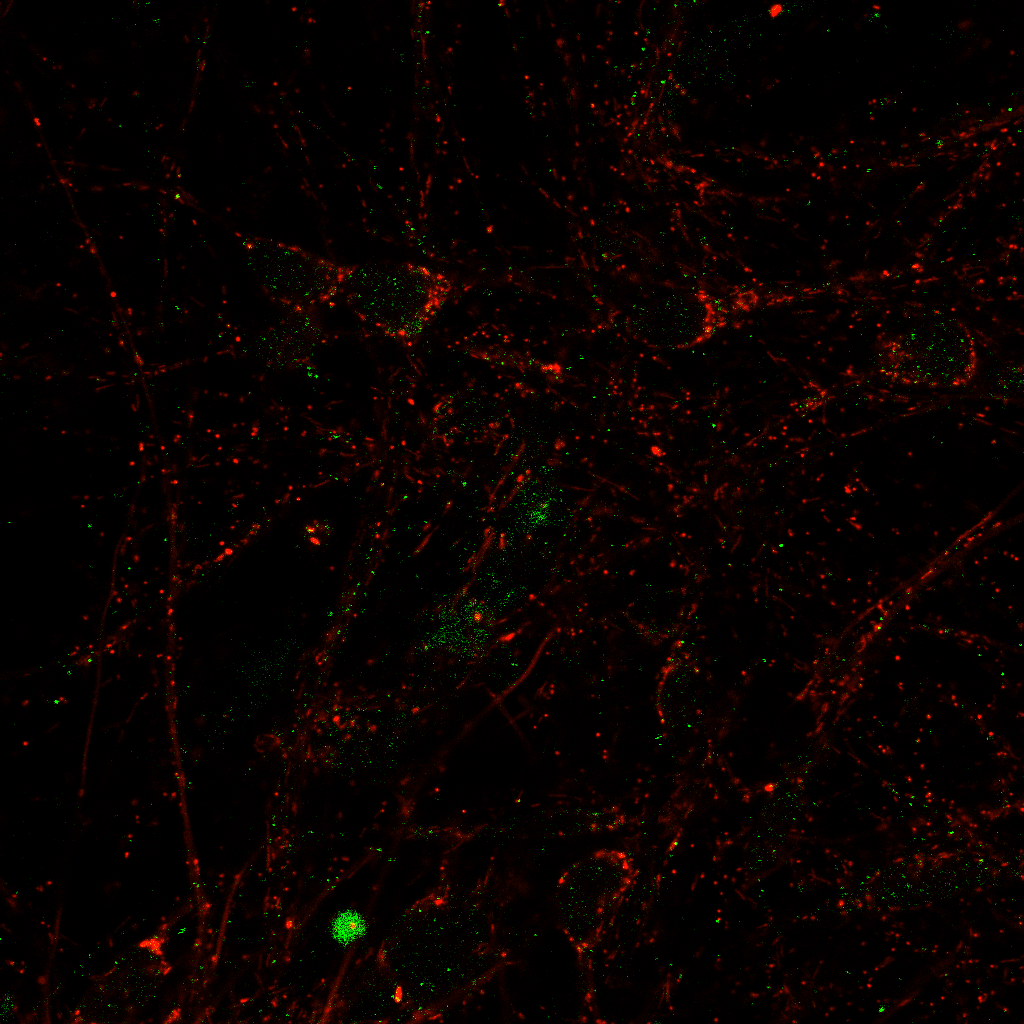

Supplement: Supplementary file 9 — Source data Fig. 5 [file 44321_2025_323_MOESM9_ESM.zip › Figure 5/5C/C9-ISO-SHOW-C.tif]

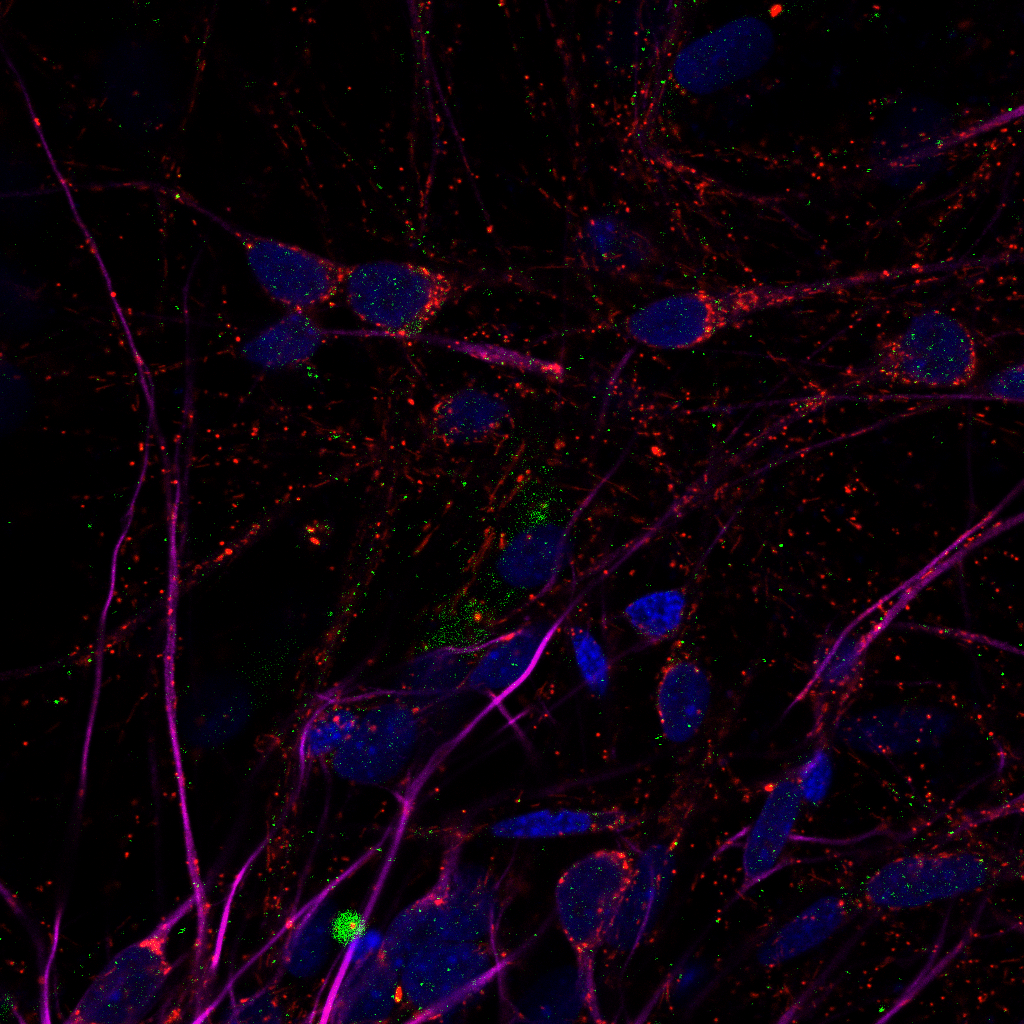

Supplement: Supplementary file 9 — Source data Fig. 5 [file 44321_2025_323_MOESM9_ESM.zip › Figure 5/5C/C9-ISO-SHOW-M.tif]

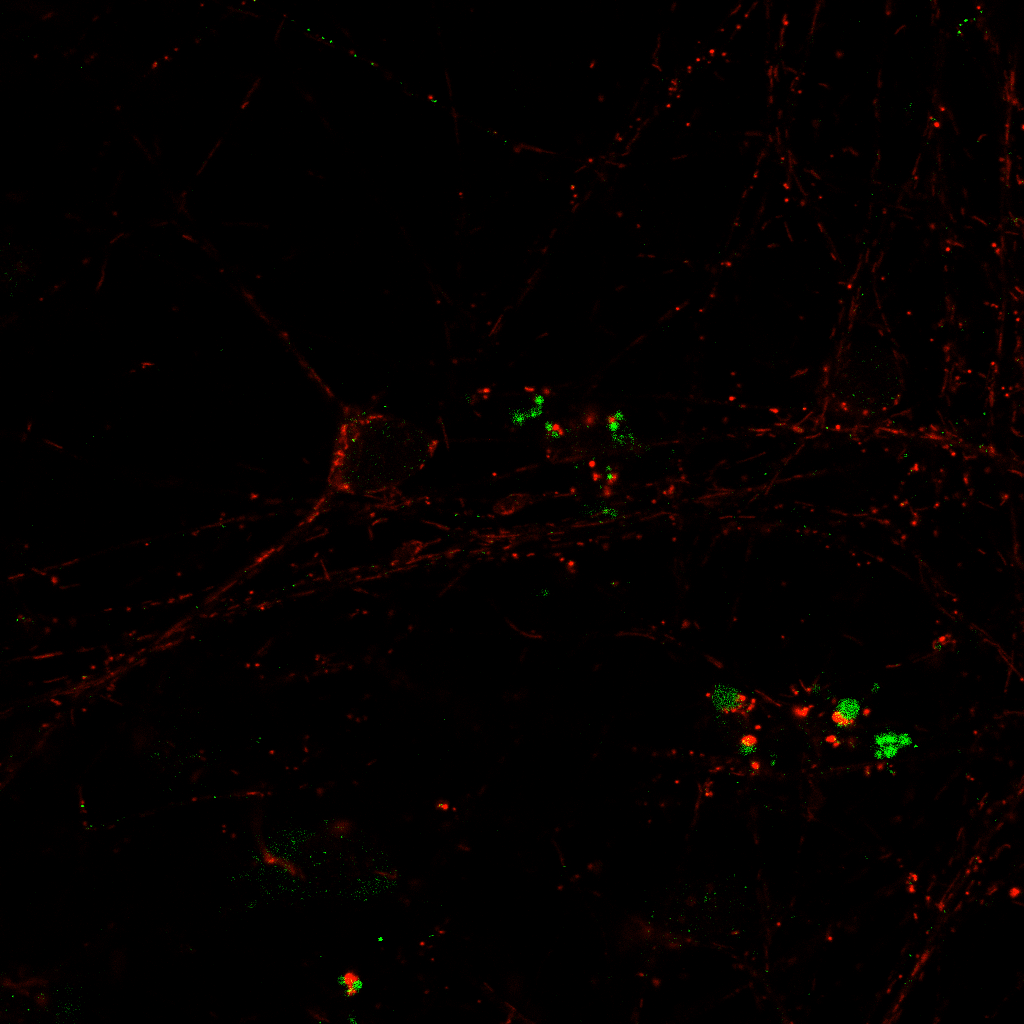

Supplement: Supplementary file 9 — Source data Fig. 5 [file 44321_2025_323_MOESM9_ESM.zip › Figure 5/5C/C9-VEH-SHOW-C.tif]

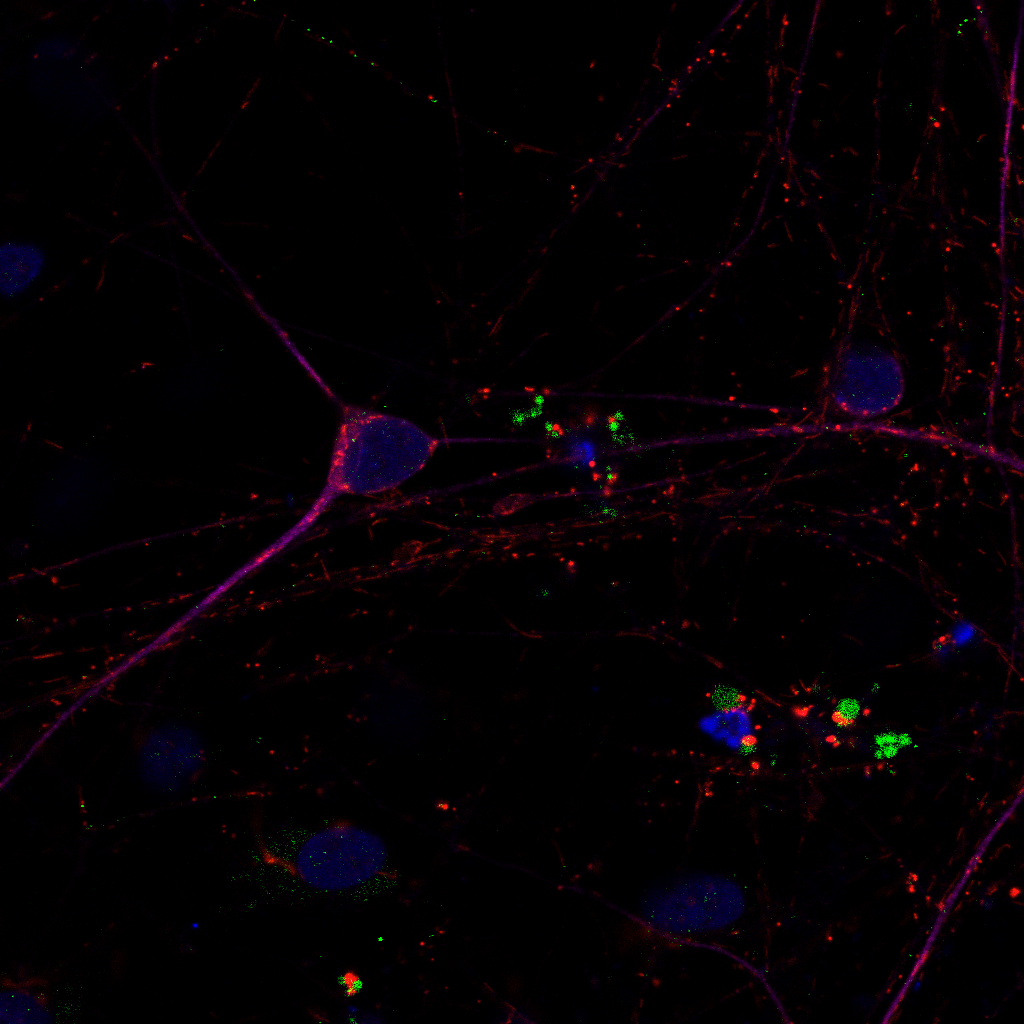

Supplement: Supplementary file 9 — Source data Fig. 5 [file 44321_2025_323_MOESM9_ESM.zip › Figure 5/5C/C9-VEH-SHOW-M.tif]

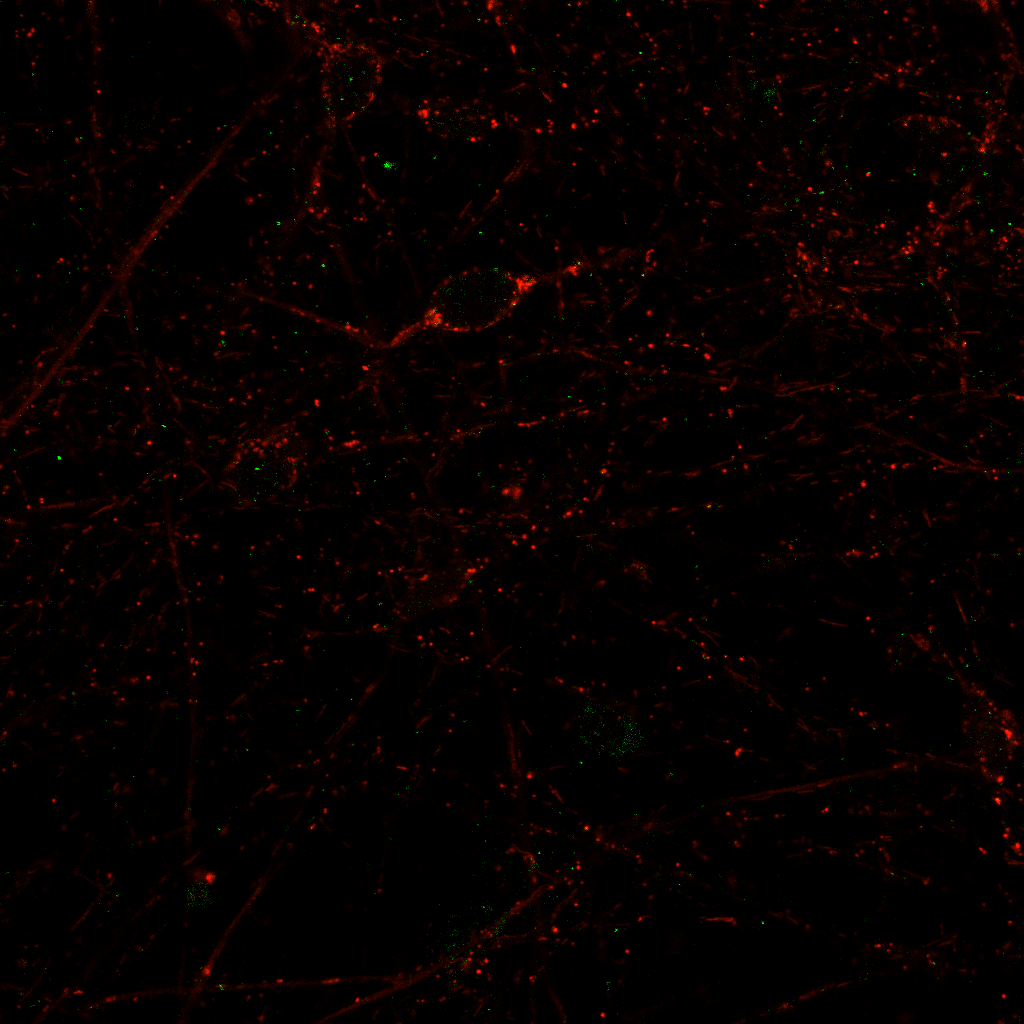

Supplement: Supplementary file 9 — Source data Fig. 5 [file 44321_2025_323_MOESM9_ESM.zip › Figure 5/5C/SOD1-ISO-SHOW-C.tif]

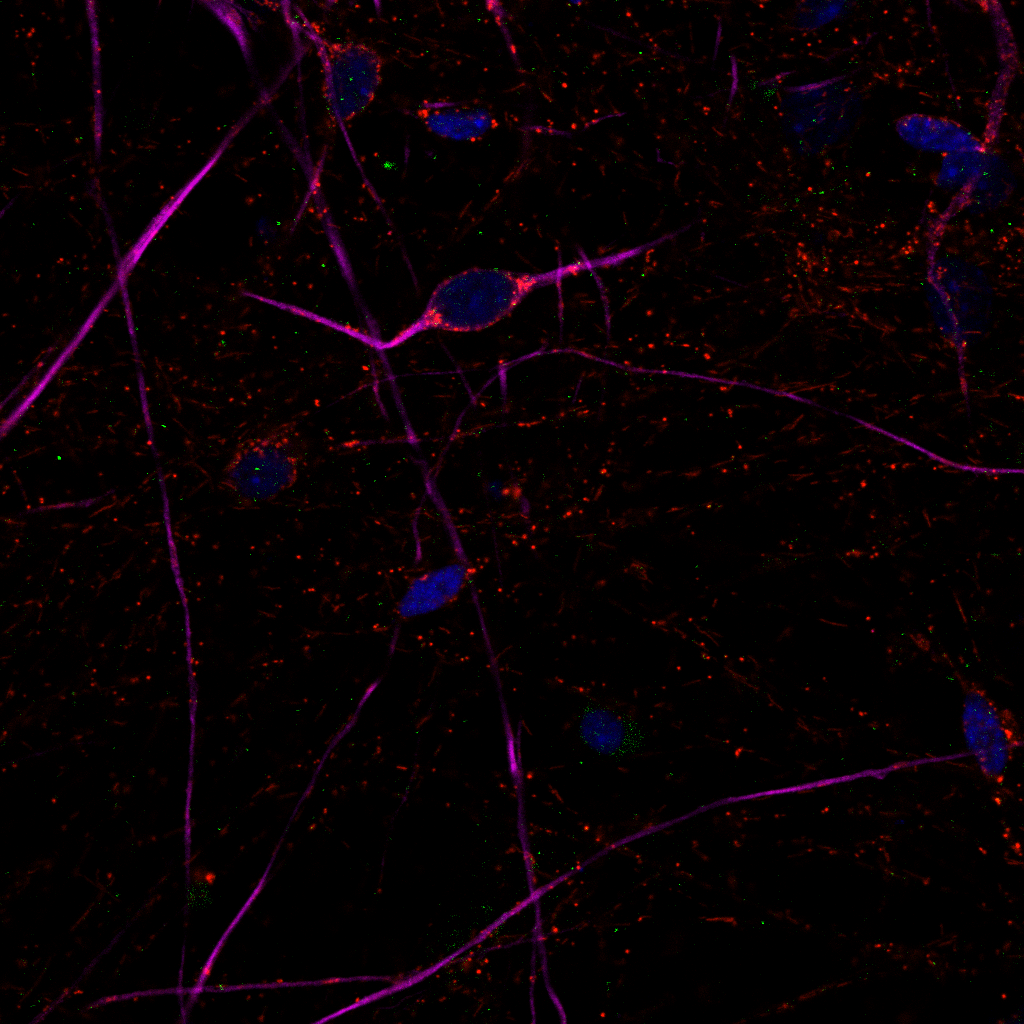

Supplement: Supplementary file 9 — Source data Fig. 5 [file 44321_2025_323_MOESM9_ESM.zip › Figure 5/5C/SOD1-ISO-SHOW-M.tif]

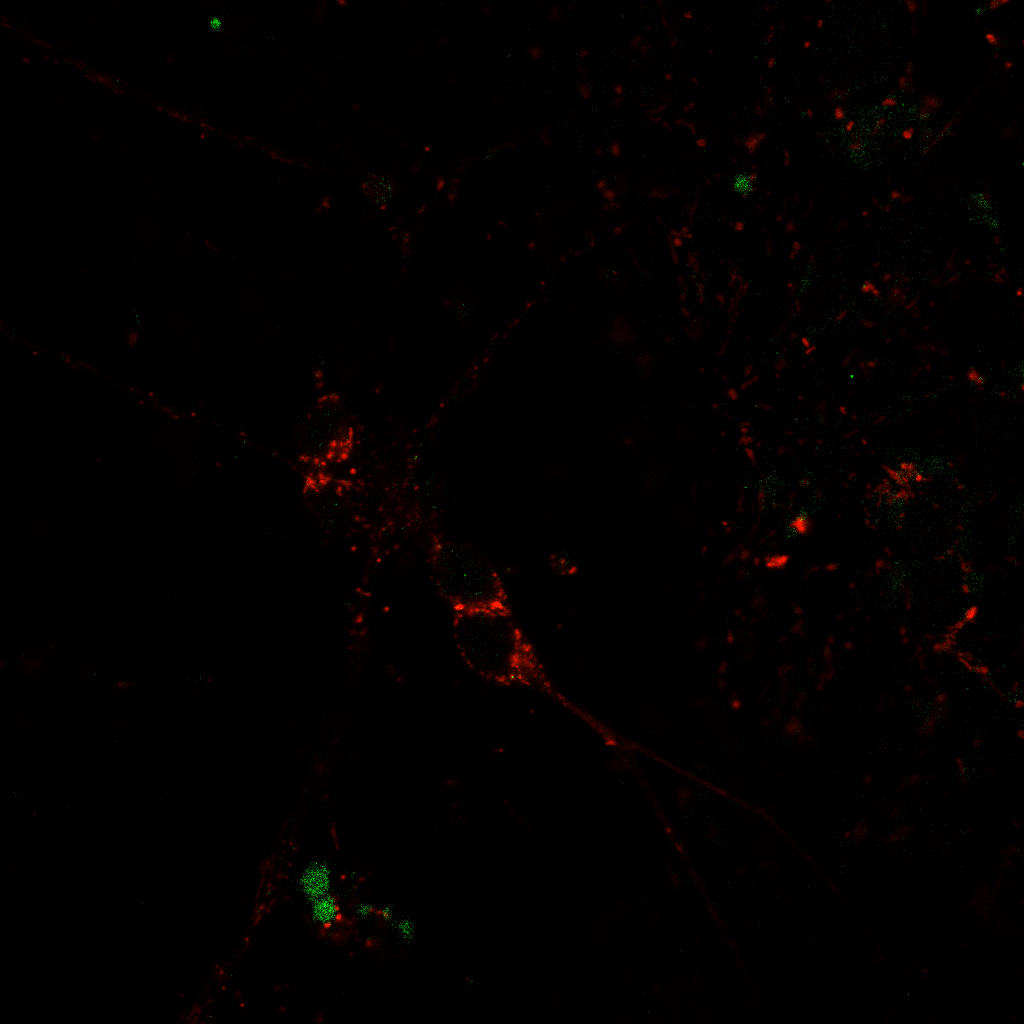

Supplement: Supplementary file 9 — Source data Fig. 5 [file 44321_2025_323_MOESM9_ESM.zip › Figure 5/5C/SOD1-VEH-SHOW-C.tif]

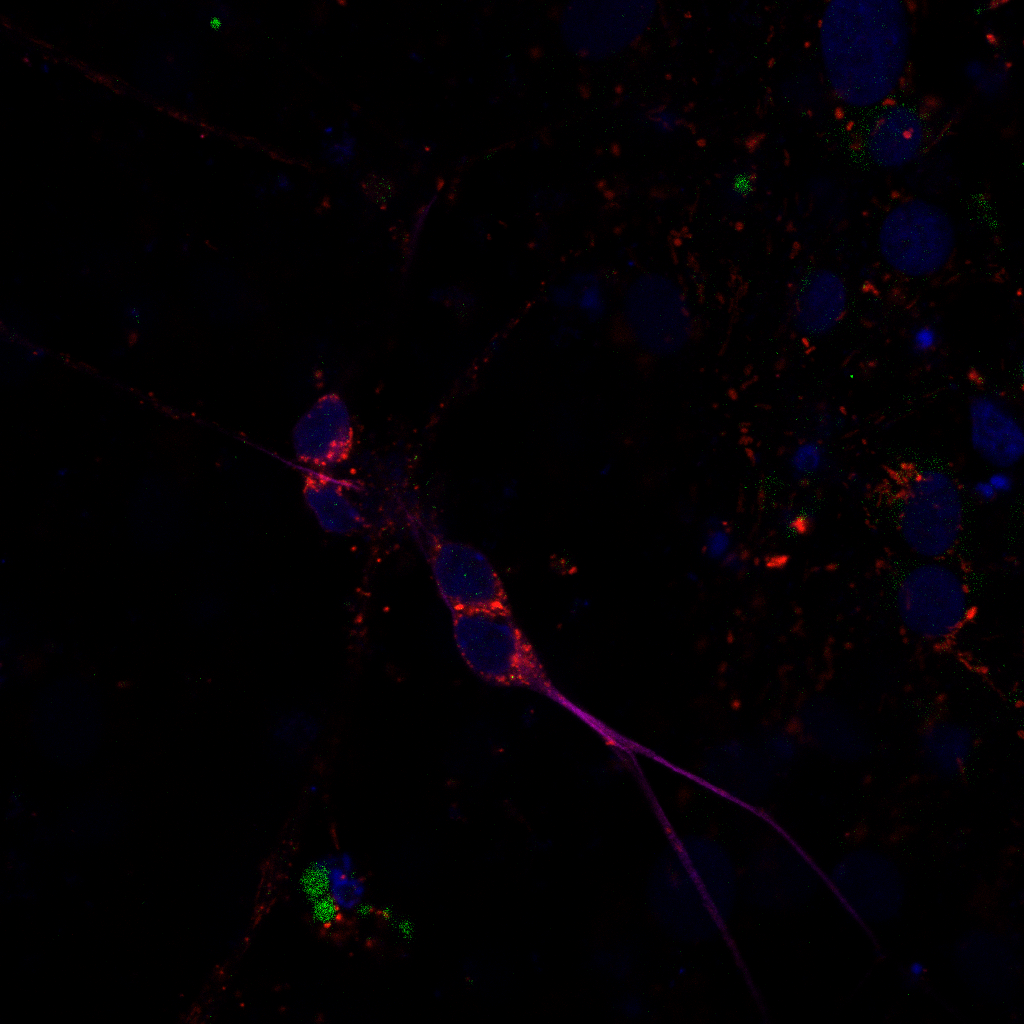

Supplement: Supplementary file 9 — Source data Fig. 5 [file 44321_2025_323_MOESM9_ESM.zip › Figure 5/5C/SOD1-VEH-SHOW-M.tif]

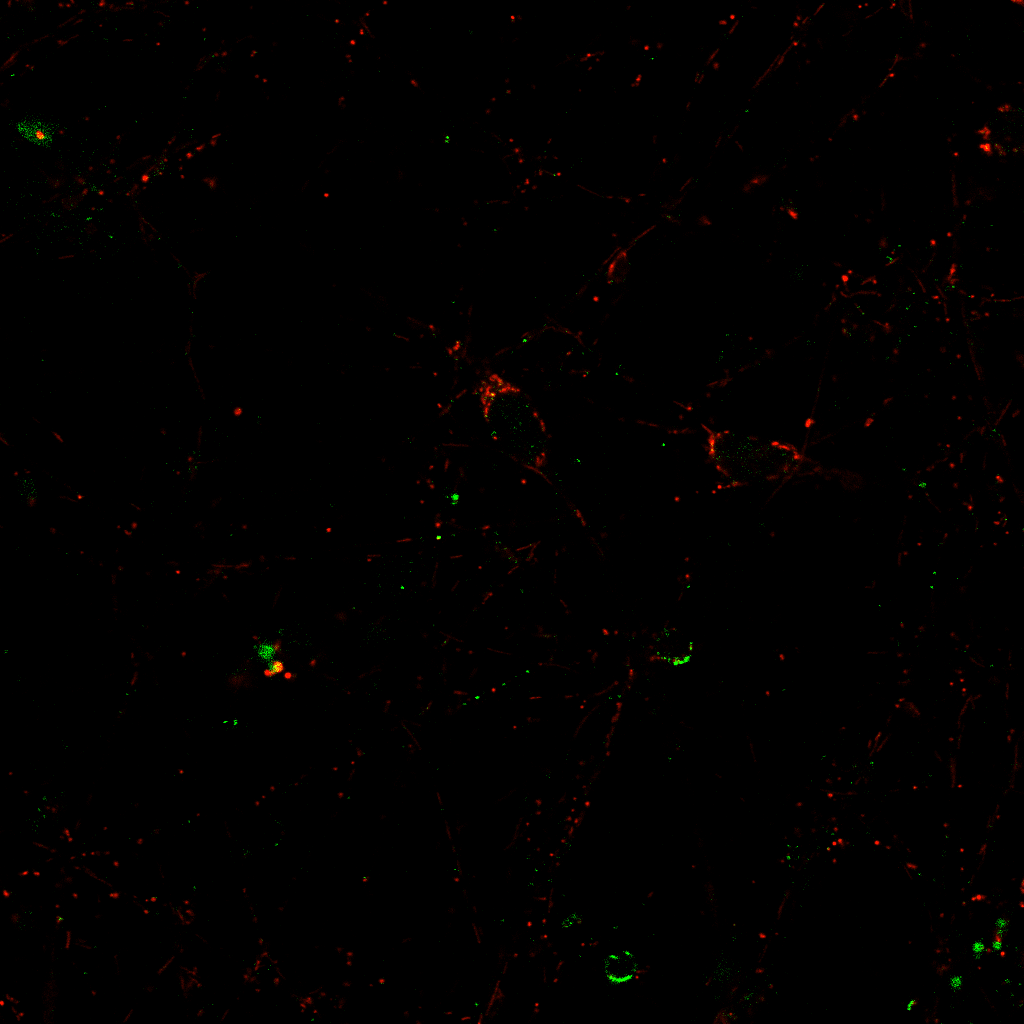

Supplement: Supplementary file 9 — Source data Fig. 5 [file 44321_2025_323_MOESM9_ESM.zip › Figure 5/5C/TDP-43-ISO-SHOW-C.tif]

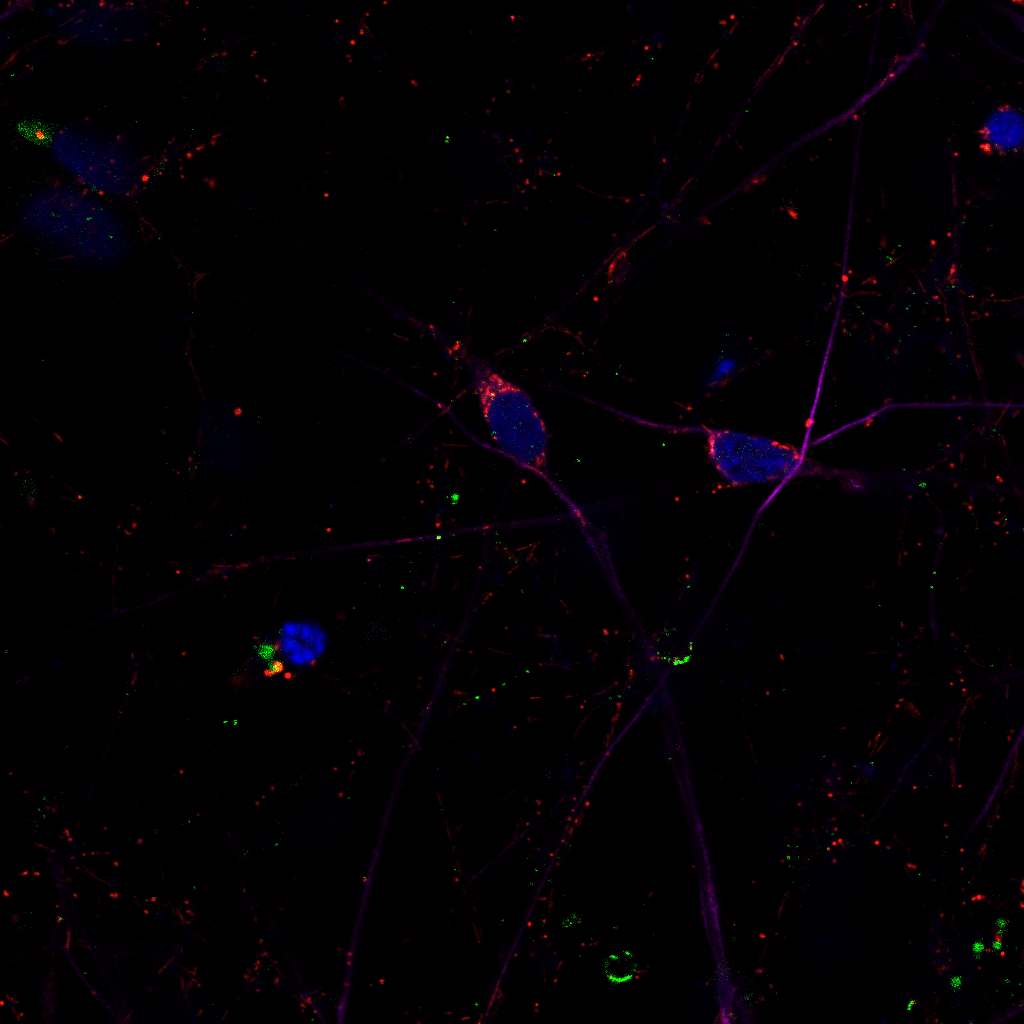

Supplement: Supplementary file 9 — Source data Fig. 5 [file 44321_2025_323_MOESM9_ESM.zip › Figure 5/5C/TDP-43-ISO-SHOW-M.tif]

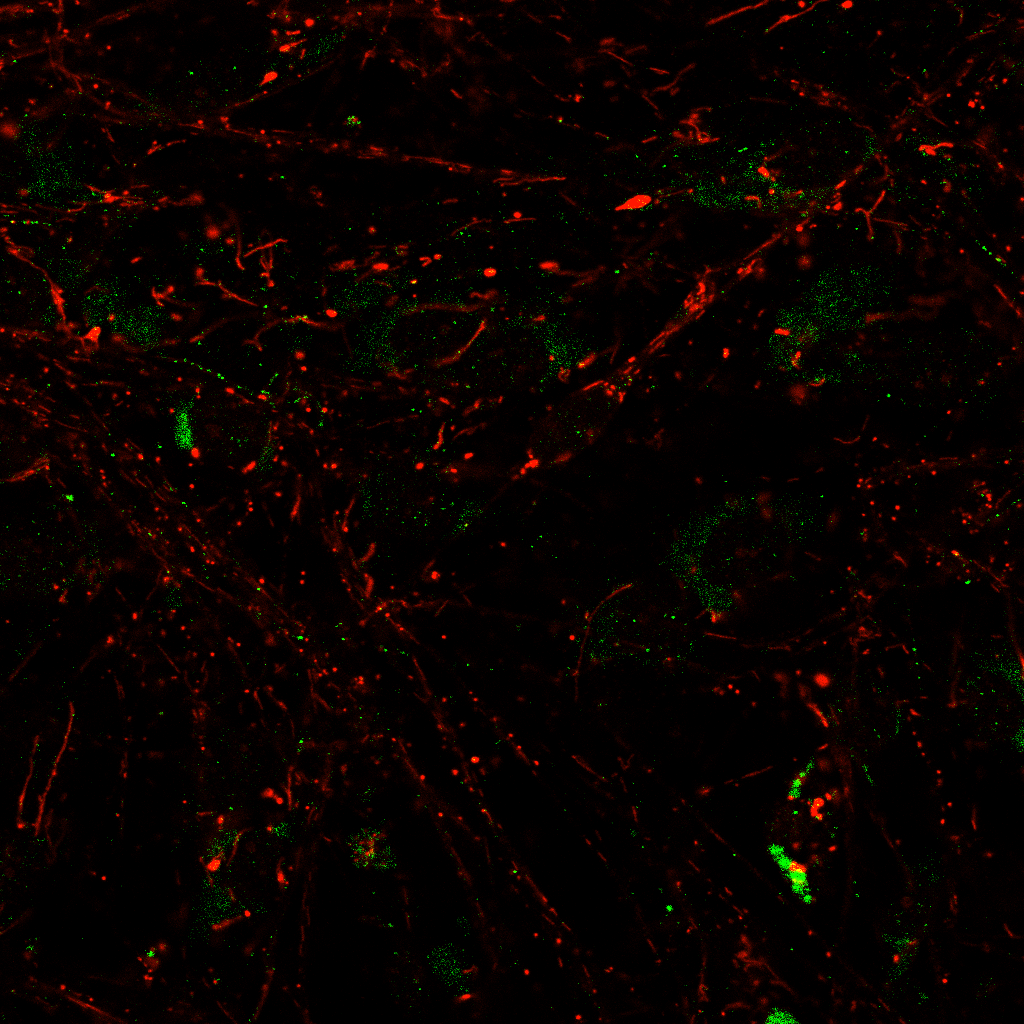

Supplement: Supplementary file 9 — Source data Fig. 5 [file 44321_2025_323_MOESM9_ESM.zip › Figure 5/5C/TDP-43-VEH-SHOW-C.tif]

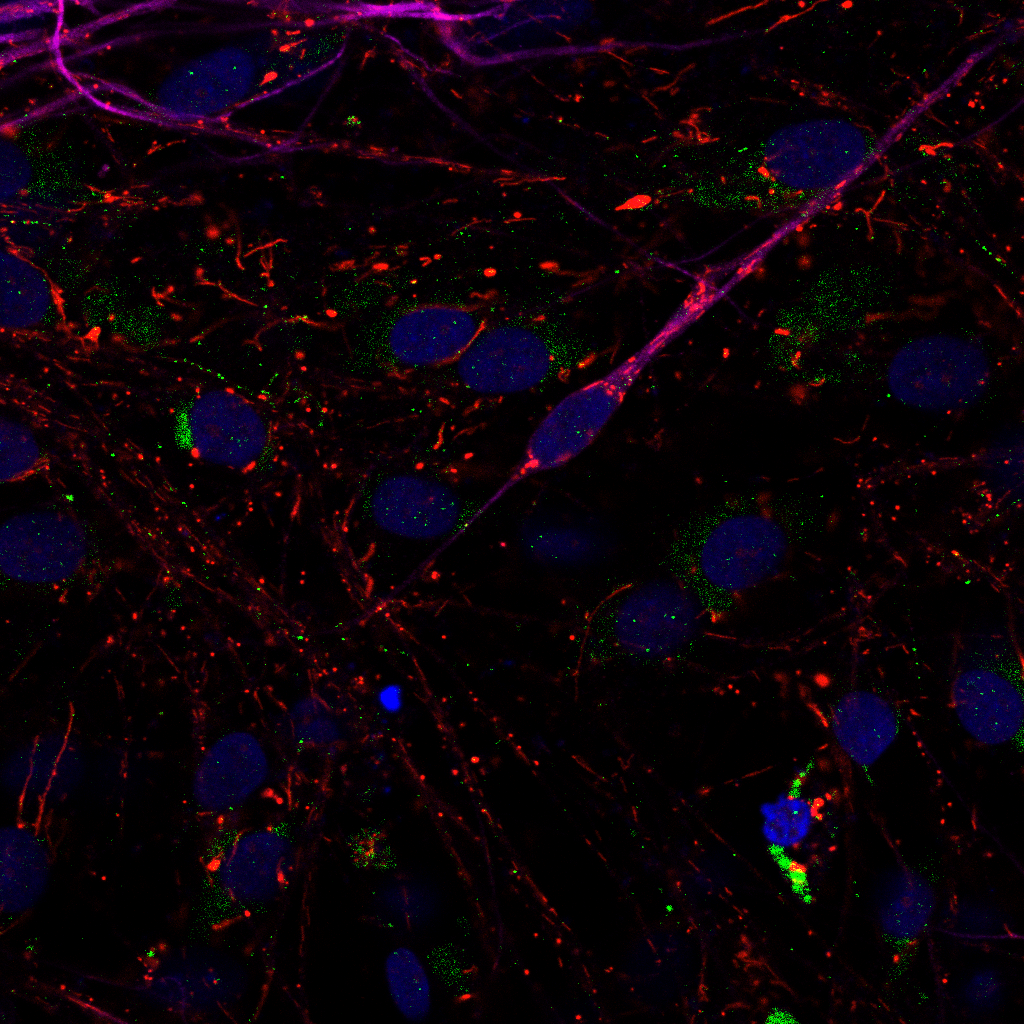

Supplement: Supplementary file 9 — Source data Fig. 5 [file 44321_2025_323_MOESM9_ESM.zip › Figure 5/5C/TDP-43-VEH-SHOW-M.tif]

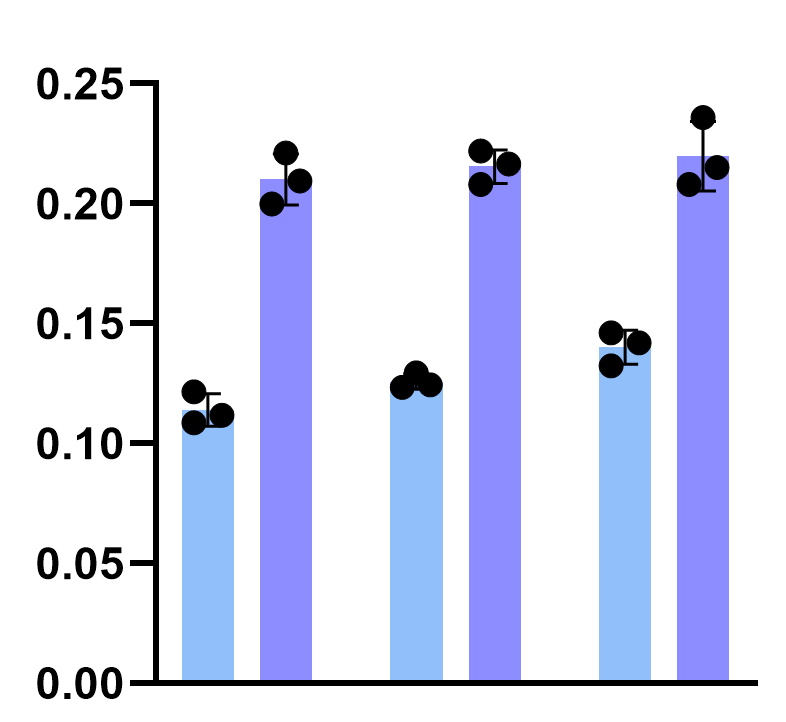

Supplement: Supplementary file 9 — Source data Fig. 5 [file 44321_2025_323_MOESM9_ESM.zip › Figure 5/5D/MITOPHAGY-P.tif]

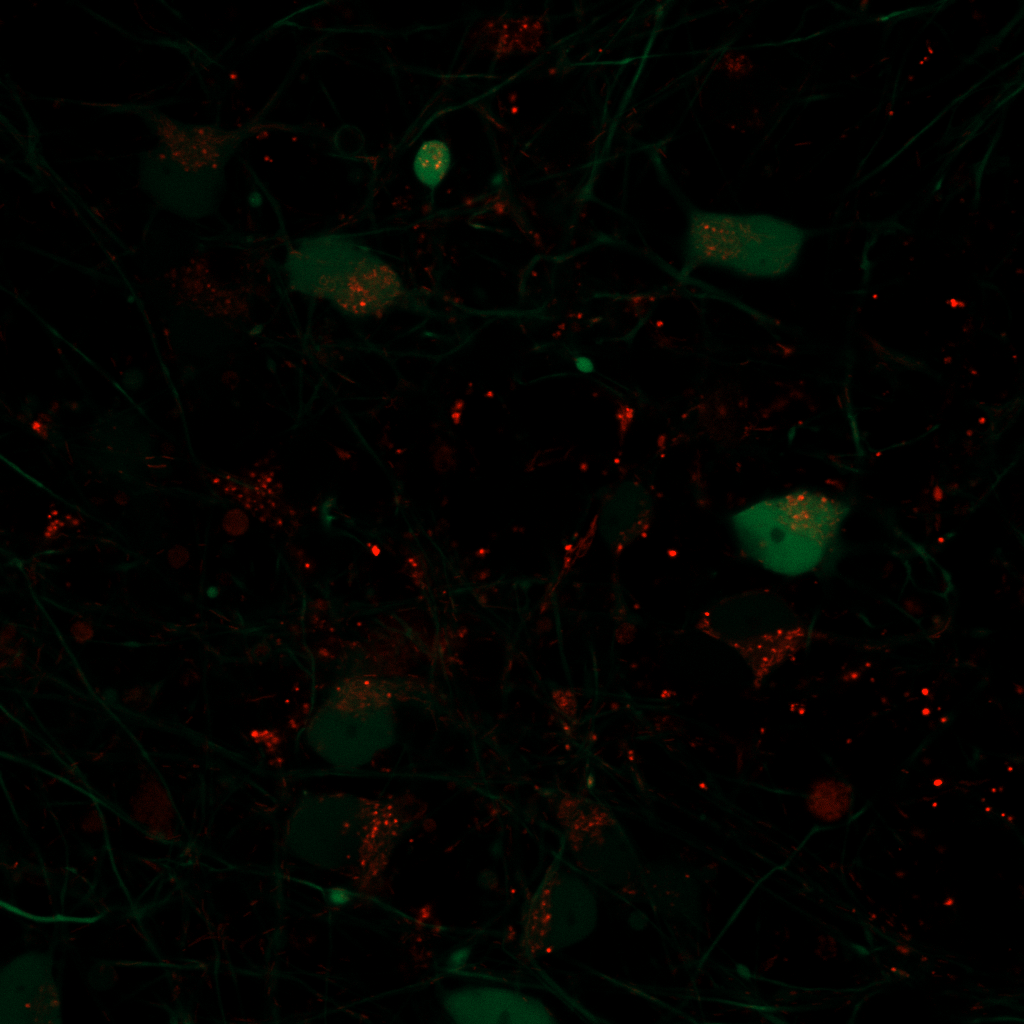

Supplement: Supplementary file 9 — Source data Fig. 5 [file 44321_2025_323_MOESM9_ESM.zip › Figure 5/5E/C9-ISO-SHOW-M.tif]

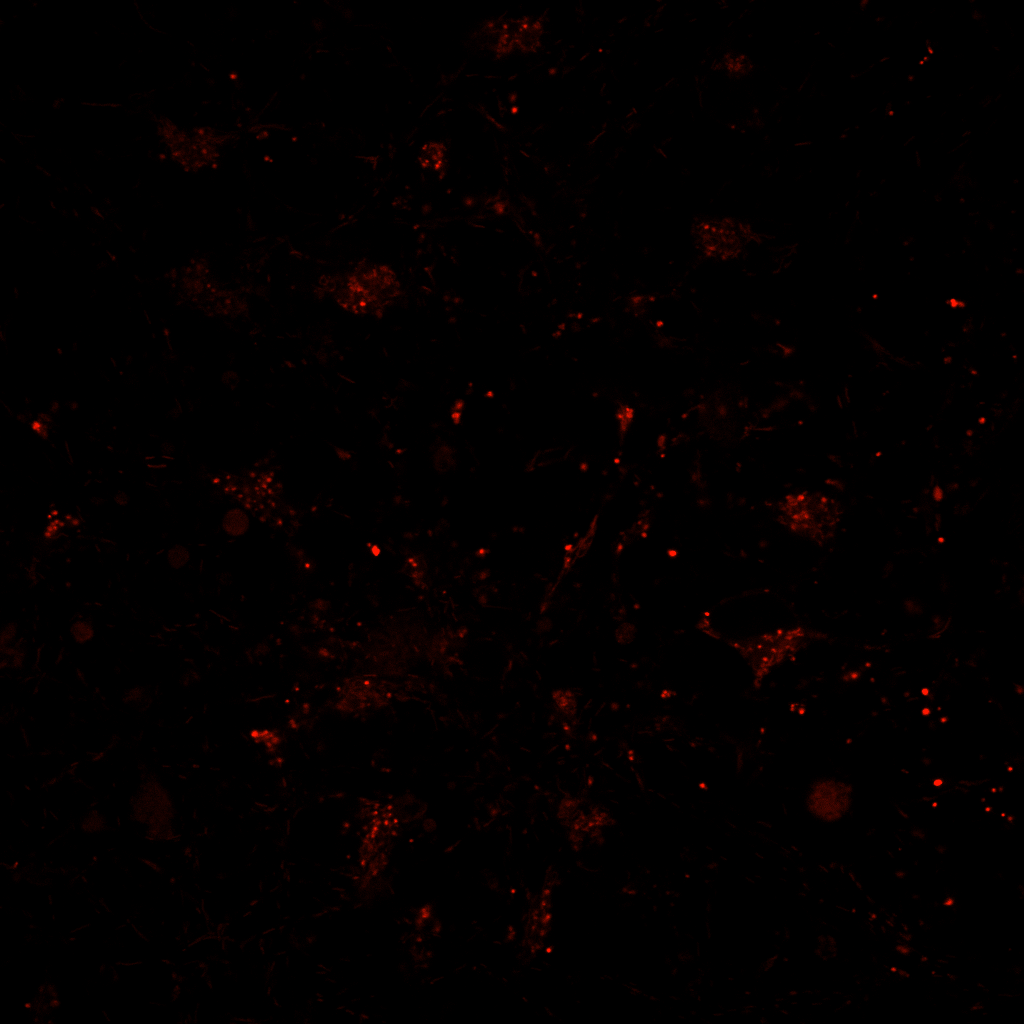

Supplement: Supplementary file 9 — Source data Fig. 5 [file 44321_2025_323_MOESM9_ESM.zip › Figure 5/5E/C9-ISO-SHOW-T.tif]

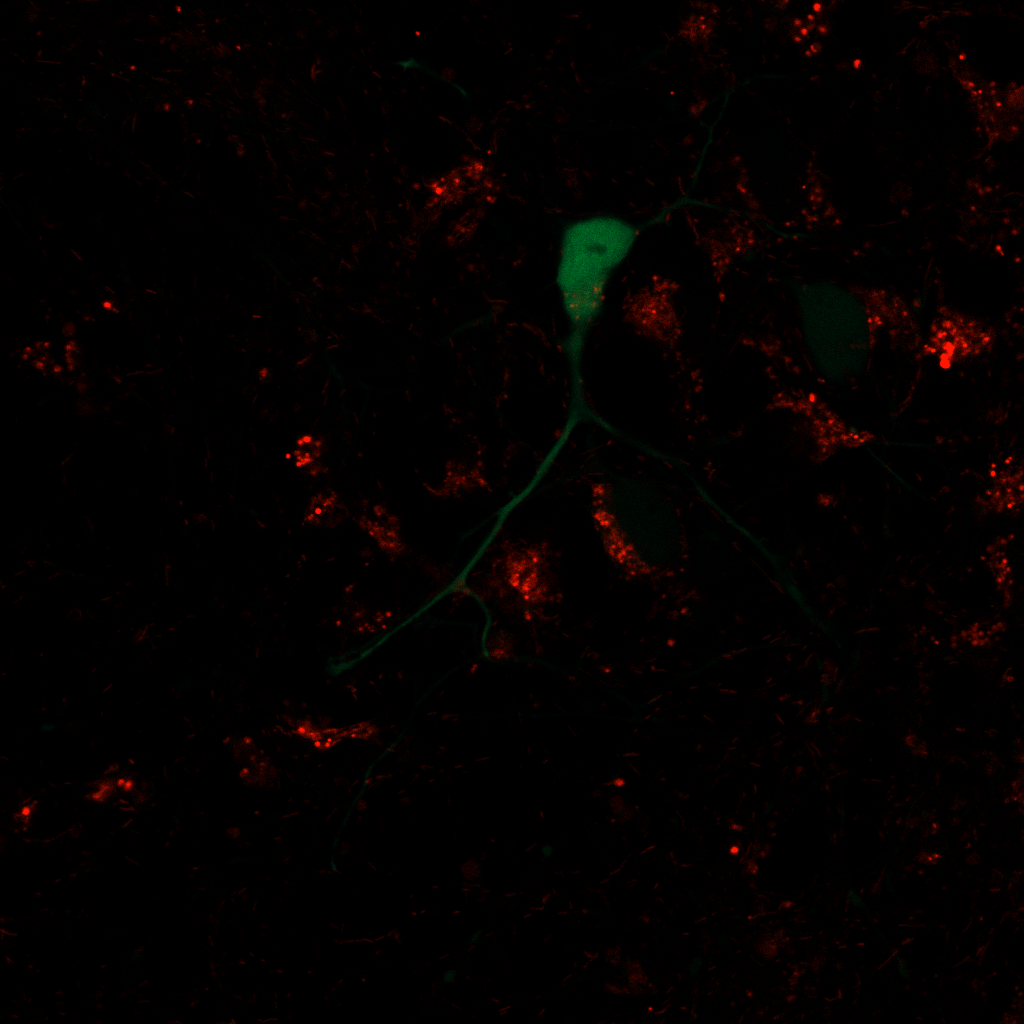

Supplement: Supplementary file 9 — Source data Fig. 5 [file 44321_2025_323_MOESM9_ESM.zip › Figure 5/5E/C9-VEH-SHOW-M.tif]

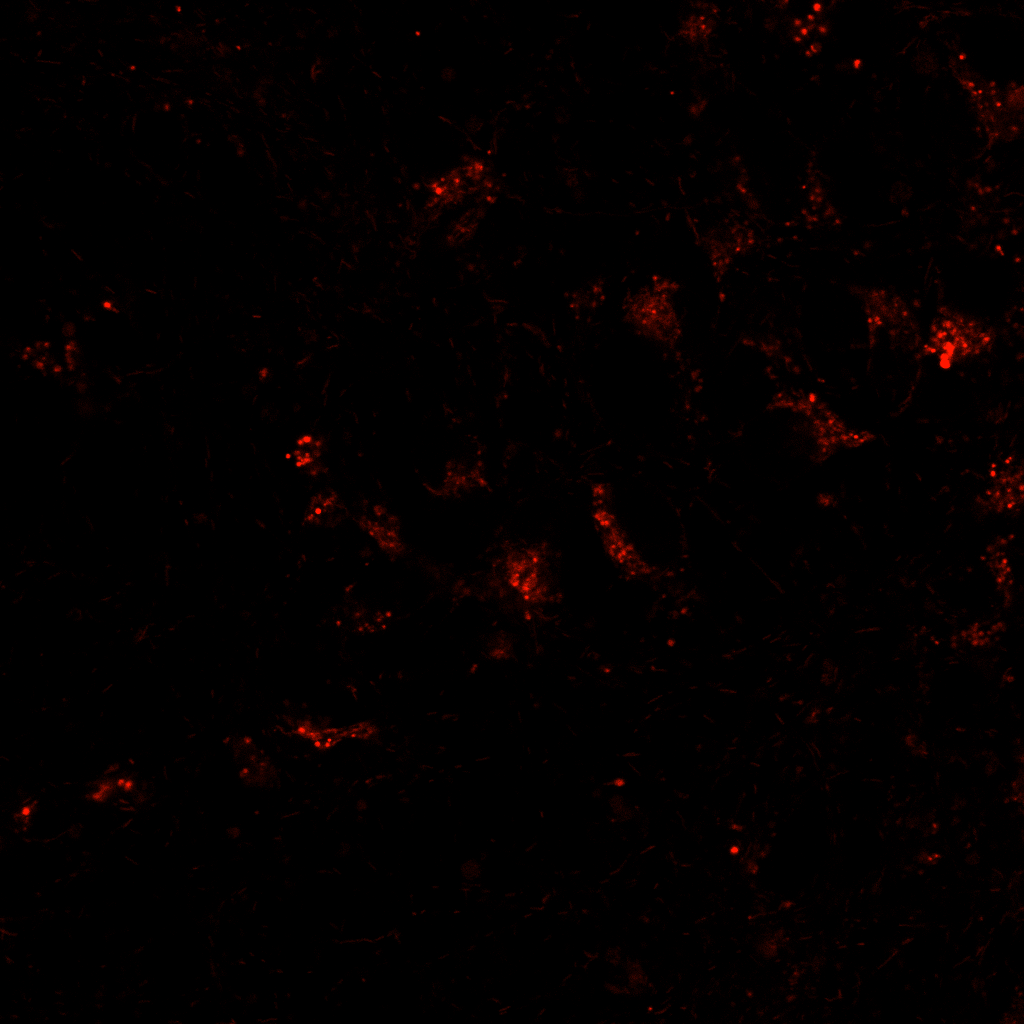

Supplement: Supplementary file 9 — Source data Fig. 5 [file 44321_2025_323_MOESM9_ESM.zip › Figure 5/5E/C9-VEH-SHOW-T.tif]

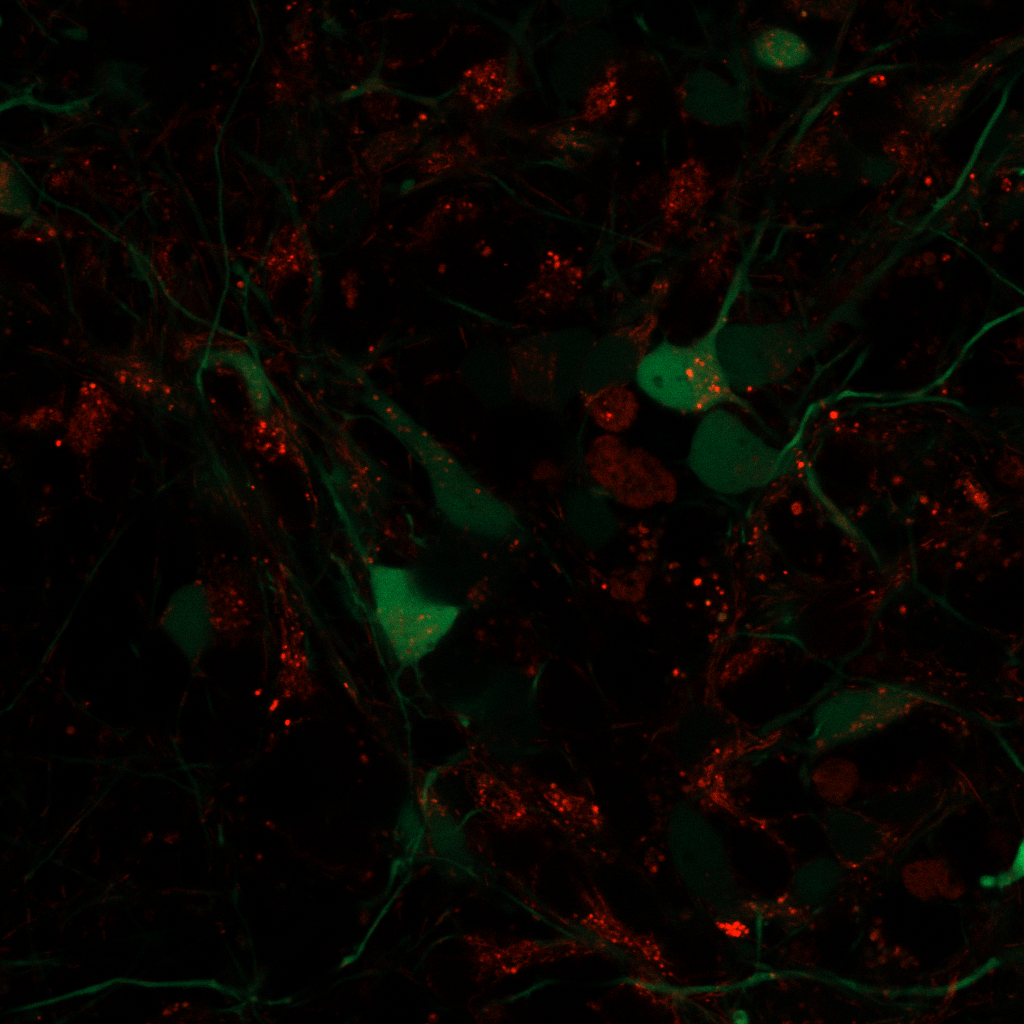

Supplement: Supplementary file 9 — Source data Fig. 5 [file 44321_2025_323_MOESM9_ESM.zip › Figure 5/5E/SOD1-ISO-SHOW-M.tif]

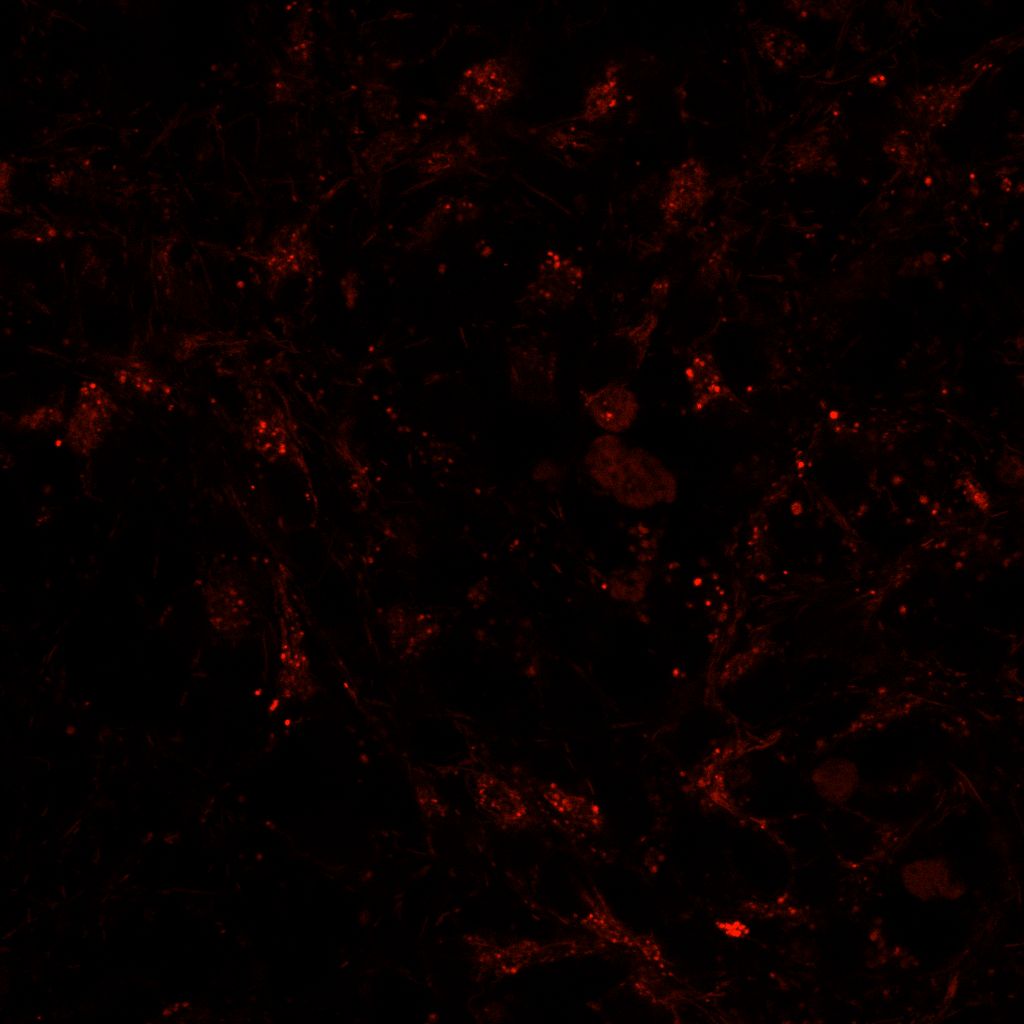

Supplement: Supplementary file 9 — Source data Fig. 5 [file 44321_2025_323_MOESM9_ESM.zip › Figure 5/5E/SOD1-ISO-SHOW-T.tif]

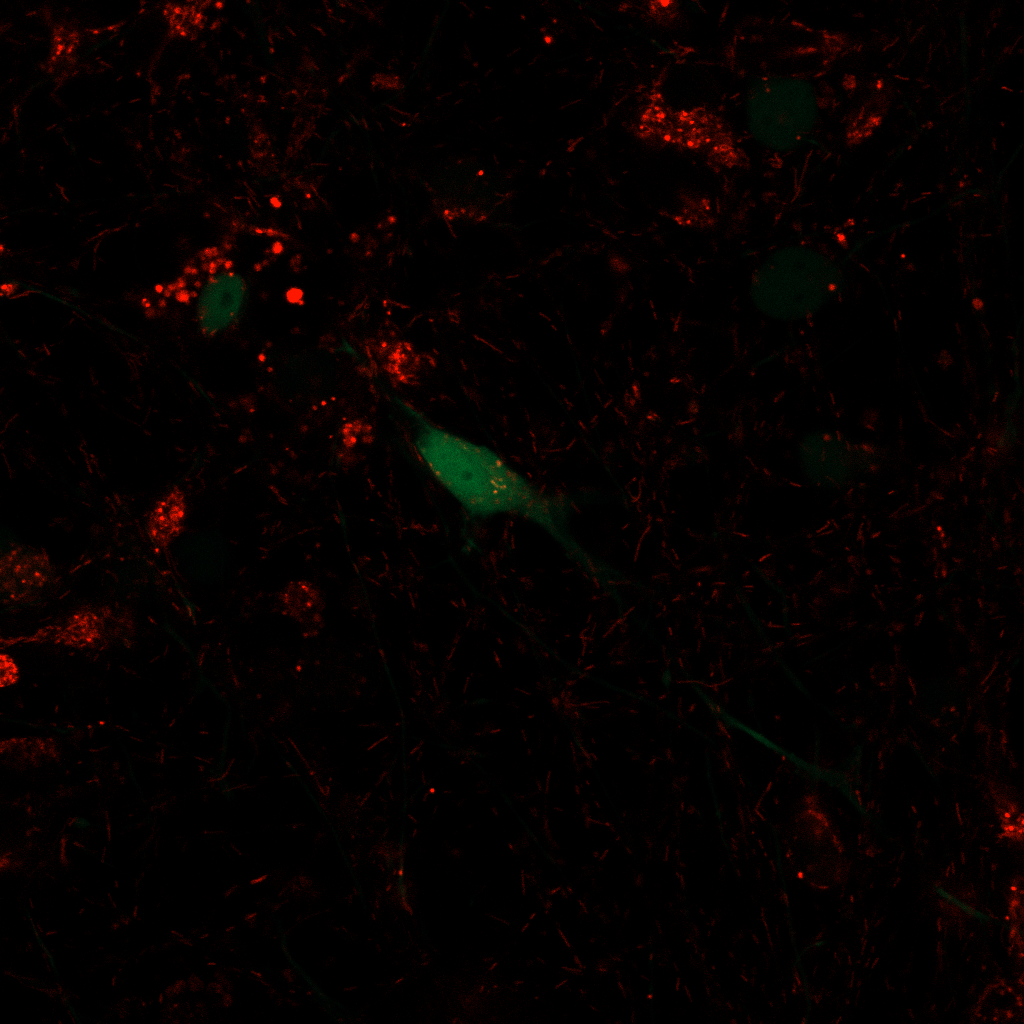

Supplement: Supplementary file 9 — Source data Fig. 5 [file 44321_2025_323_MOESM9_ESM.zip › Figure 5/5E/SOD1-VEH-SHOW-M.tif]

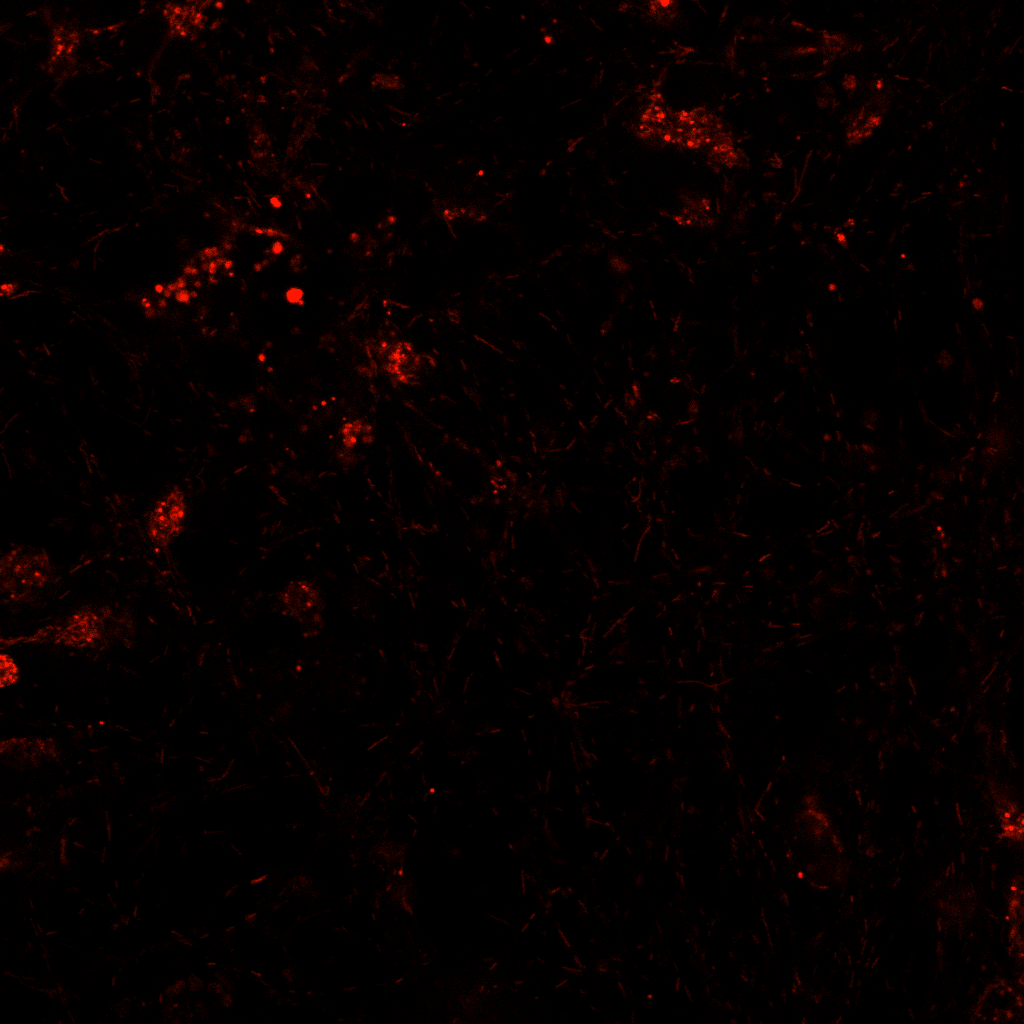

Supplement: Supplementary file 9 — Source data Fig. 5 [file 44321_2025_323_MOESM9_ESM.zip › Figure 5/5E/SOD1-VEH-SHOW-T.tif]

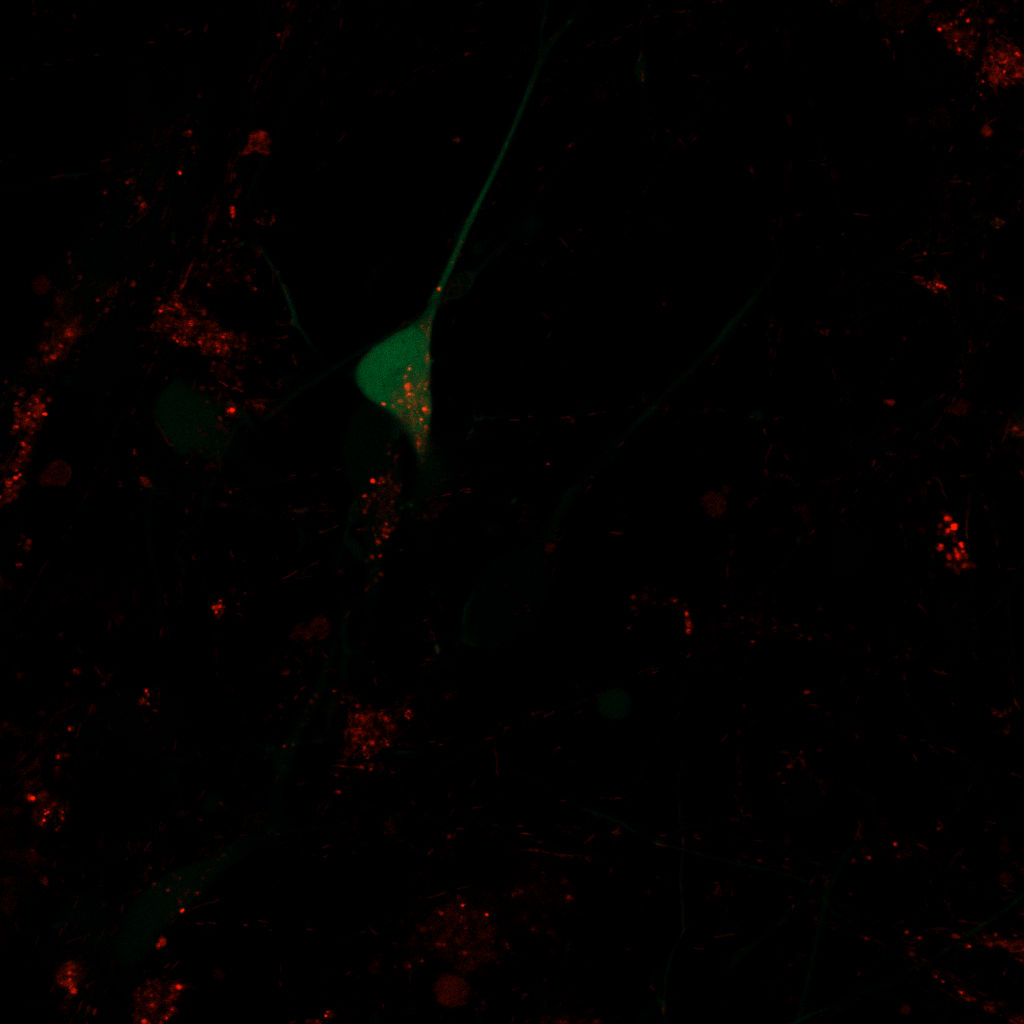

Supplement: Supplementary file 9 — Source data Fig. 5 [file 44321_2025_323_MOESM9_ESM.zip › Figure 5/5E/TDP-43-ISO-SHOW-M.tif]

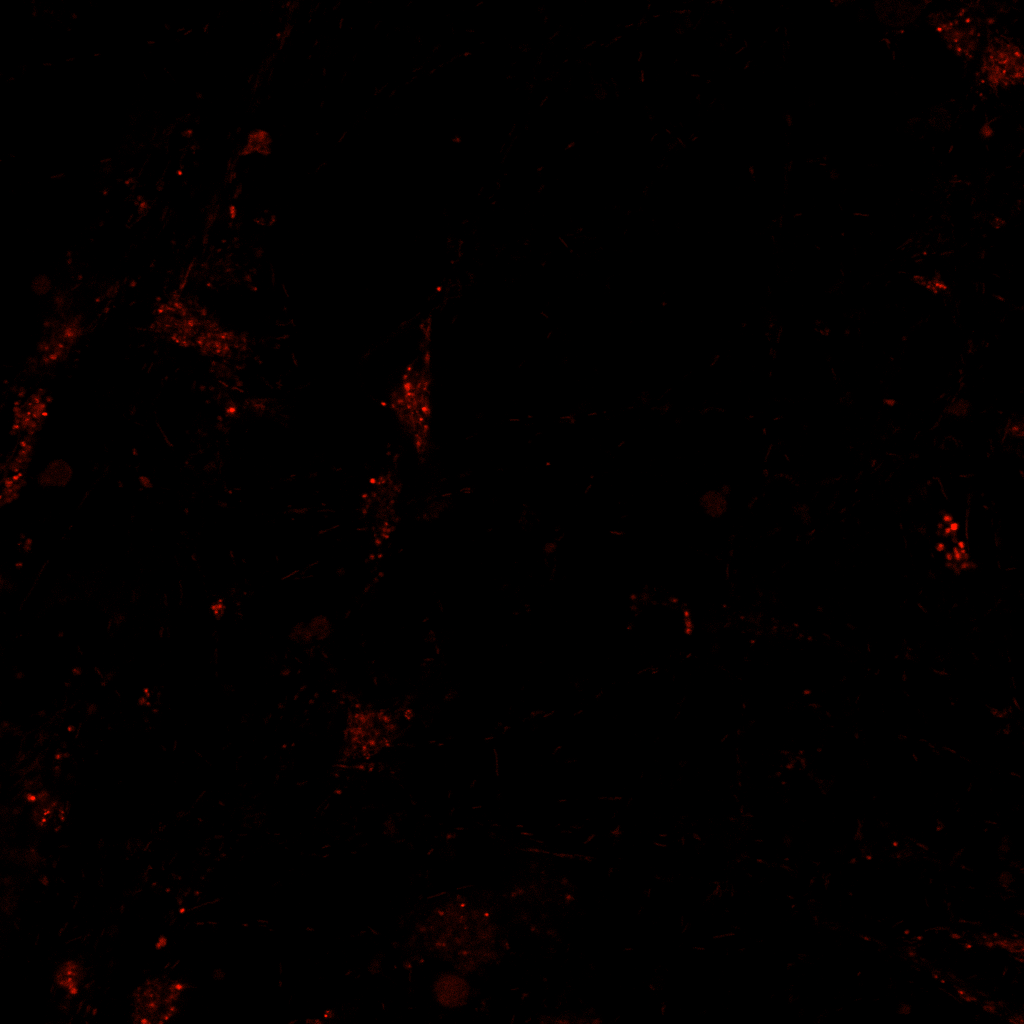

Supplement: Supplementary file 9 — Source data Fig. 5 [file 44321_2025_323_MOESM9_ESM.zip › Figure 5/5E/TDP-43-ISO-SHOW-T.tif]

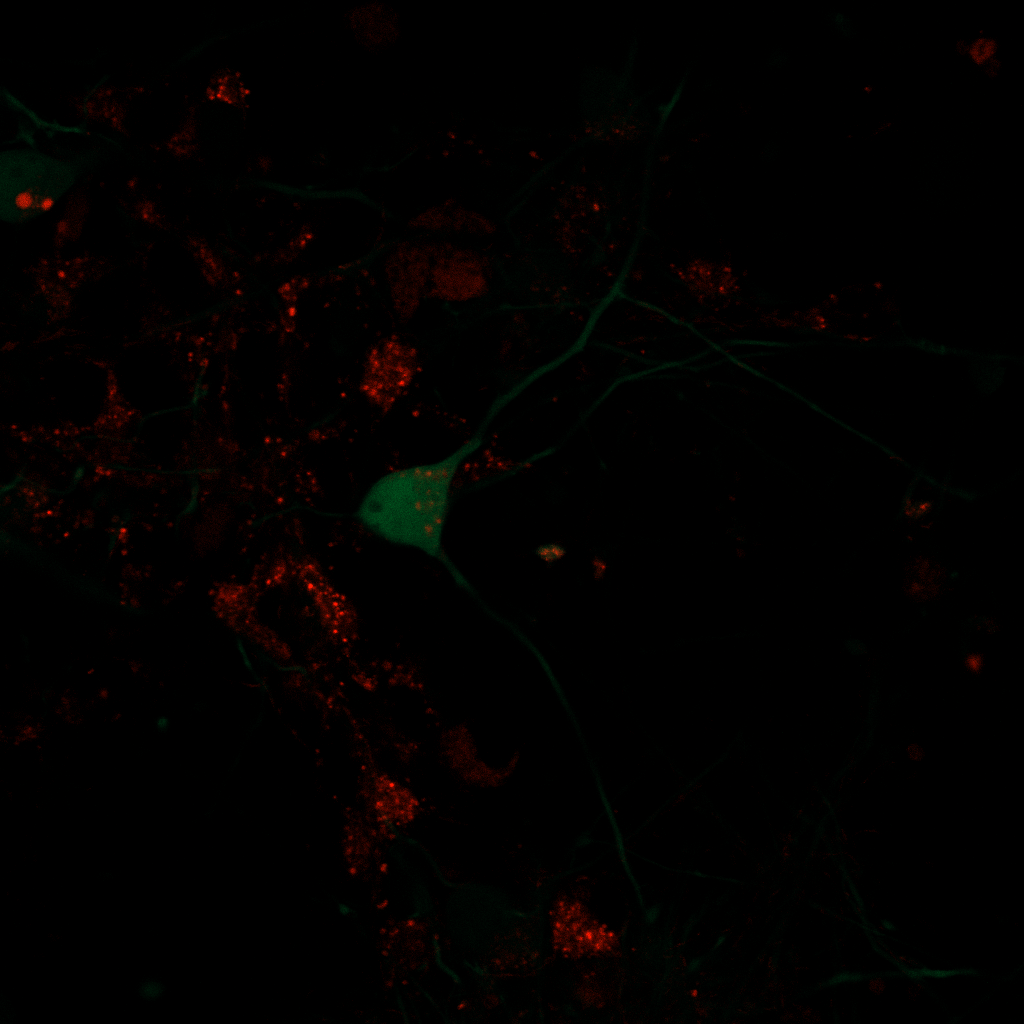

Supplement: Supplementary file 9 — Source data Fig. 5 [file 44321_2025_323_MOESM9_ESM.zip › Figure 5/5E/TDP-43-VEH-SHOW.tif]

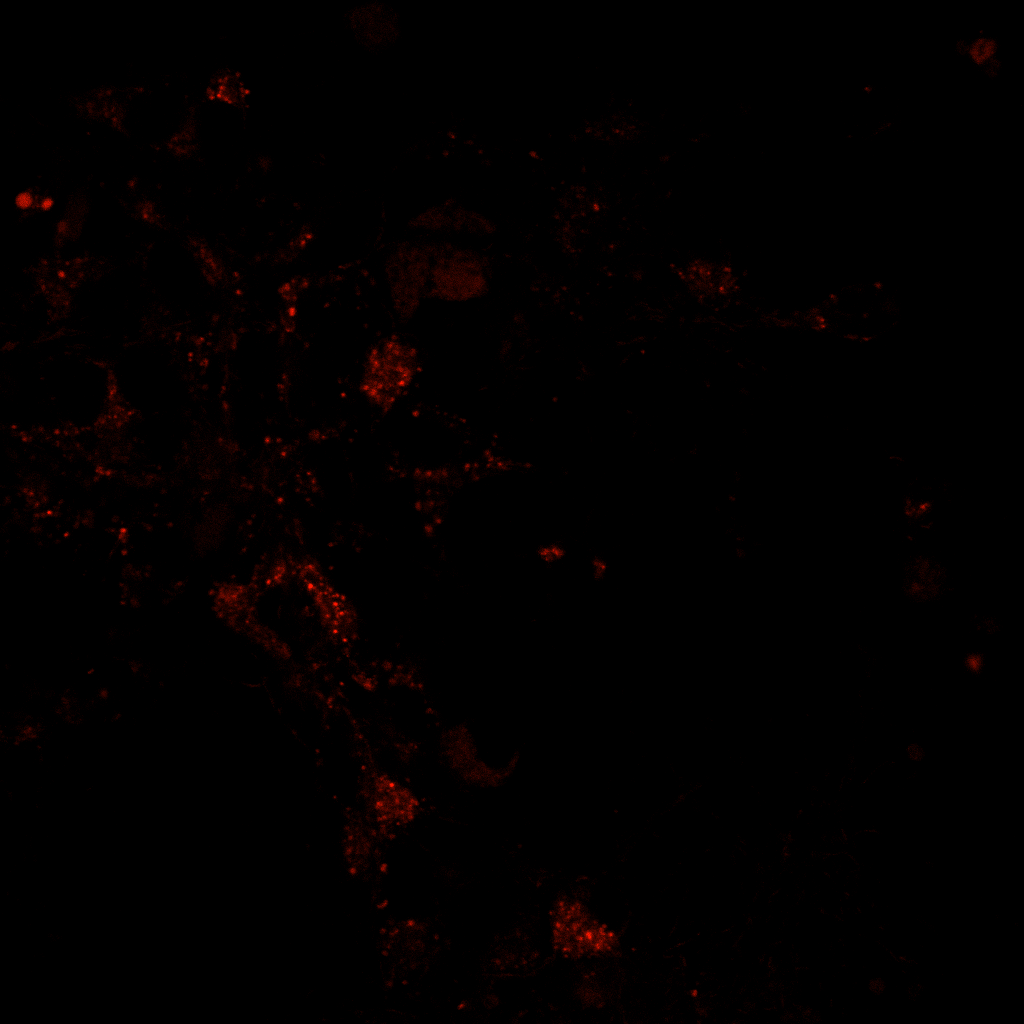

Supplement: Supplementary file 9 — Source data Fig. 5 [file 44321_2025_323_MOESM9_ESM.zip › Figure 5/5E/TDP-43-VEH-SHOW-T.tif]

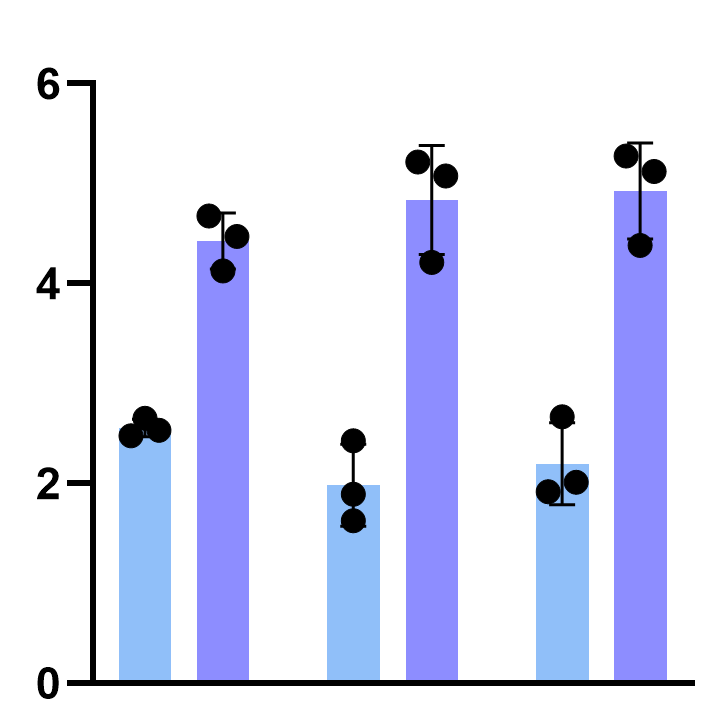

Supplement: Supplementary file 9 — Source data Fig. 5 [file 44321_2025_323_MOESM9_ESM.zip › Figure 5/5F/mitophagy.tif]

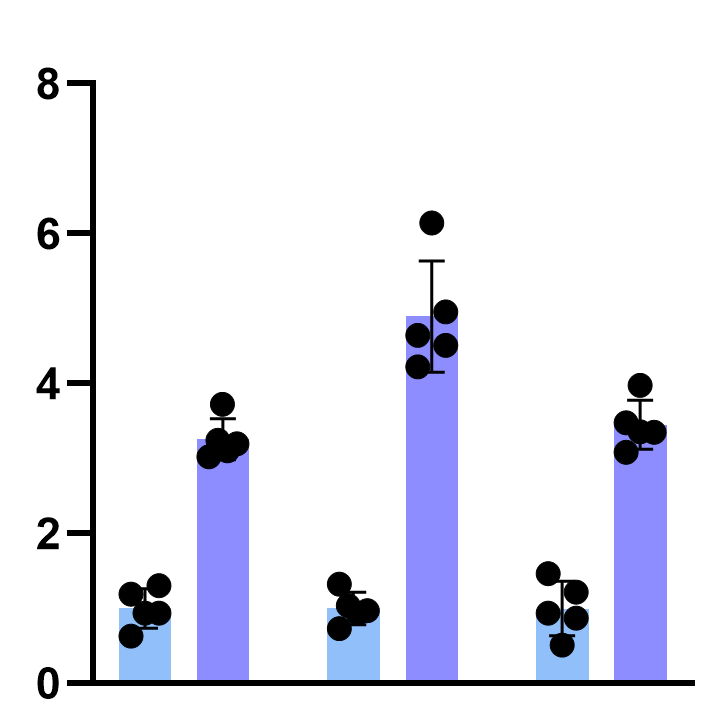

Supplement: Supplementary file 9 — Source data Fig. 5 [file 44321_2025_323_MOESM9_ESM.zip › Figure 5/5H/ATP.tif]

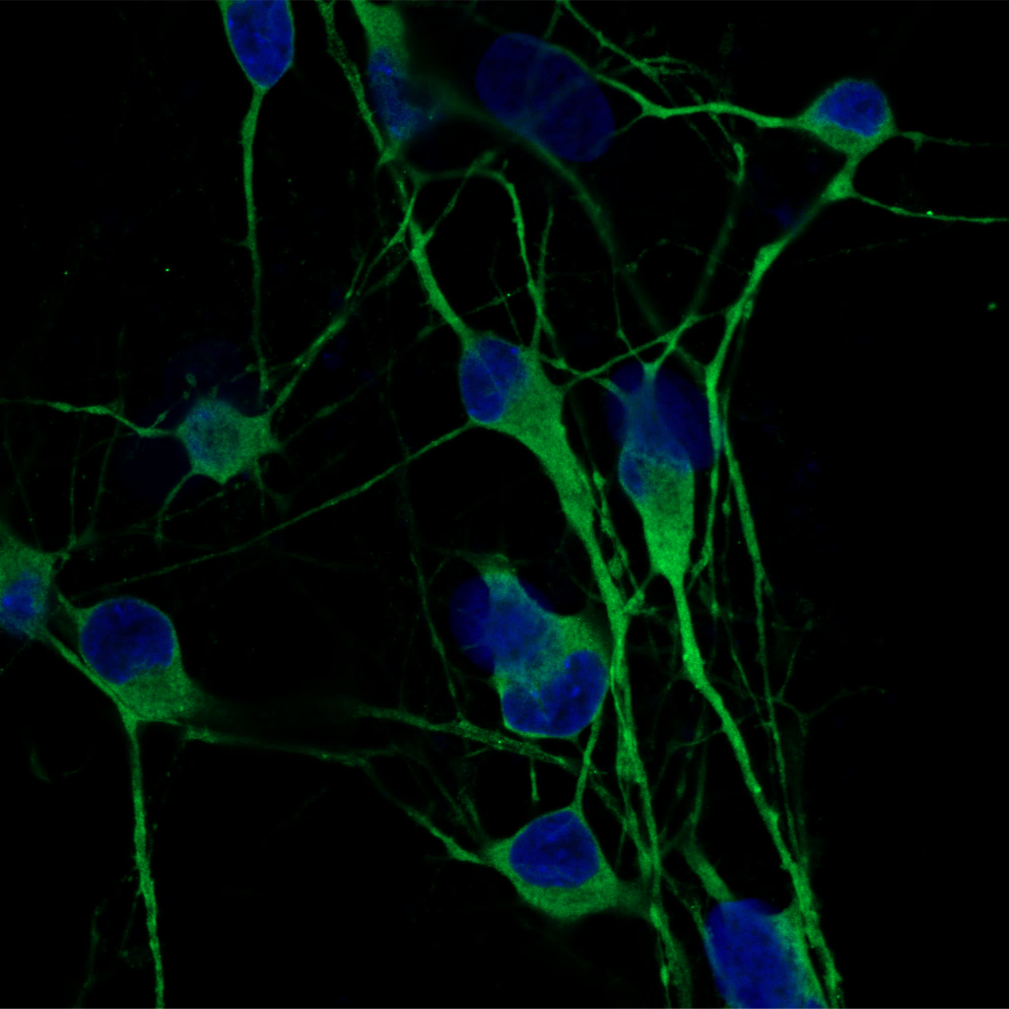

Supplement: Supplementary file 9 — Source data Fig. 5 [file 44321_2025_323_MOESM9_ESM.zip › Figure 5/5I/C9.tif]

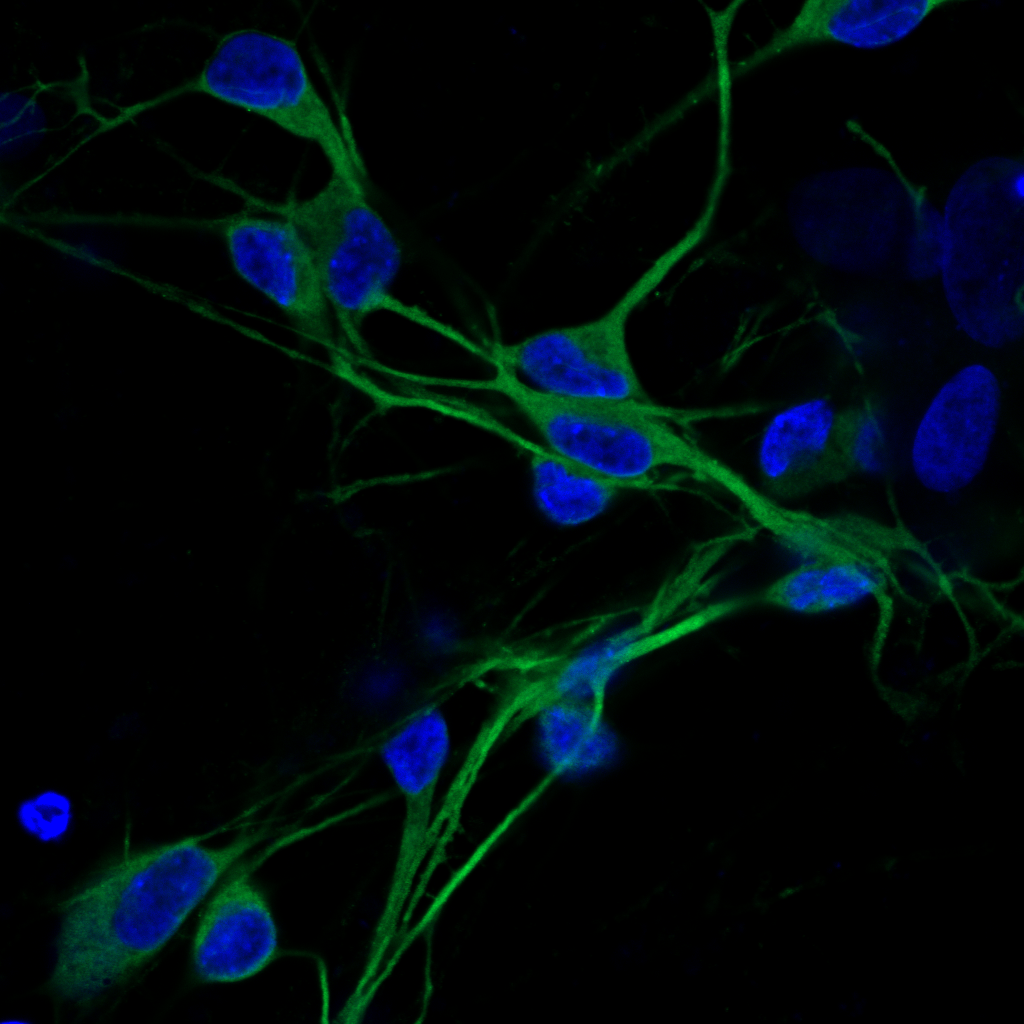

Supplement: Supplementary file 9 — Source data Fig. 5 [file 44321_2025_323_MOESM9_ESM.zip › Figure 5/5I/C9-ISO.tif]

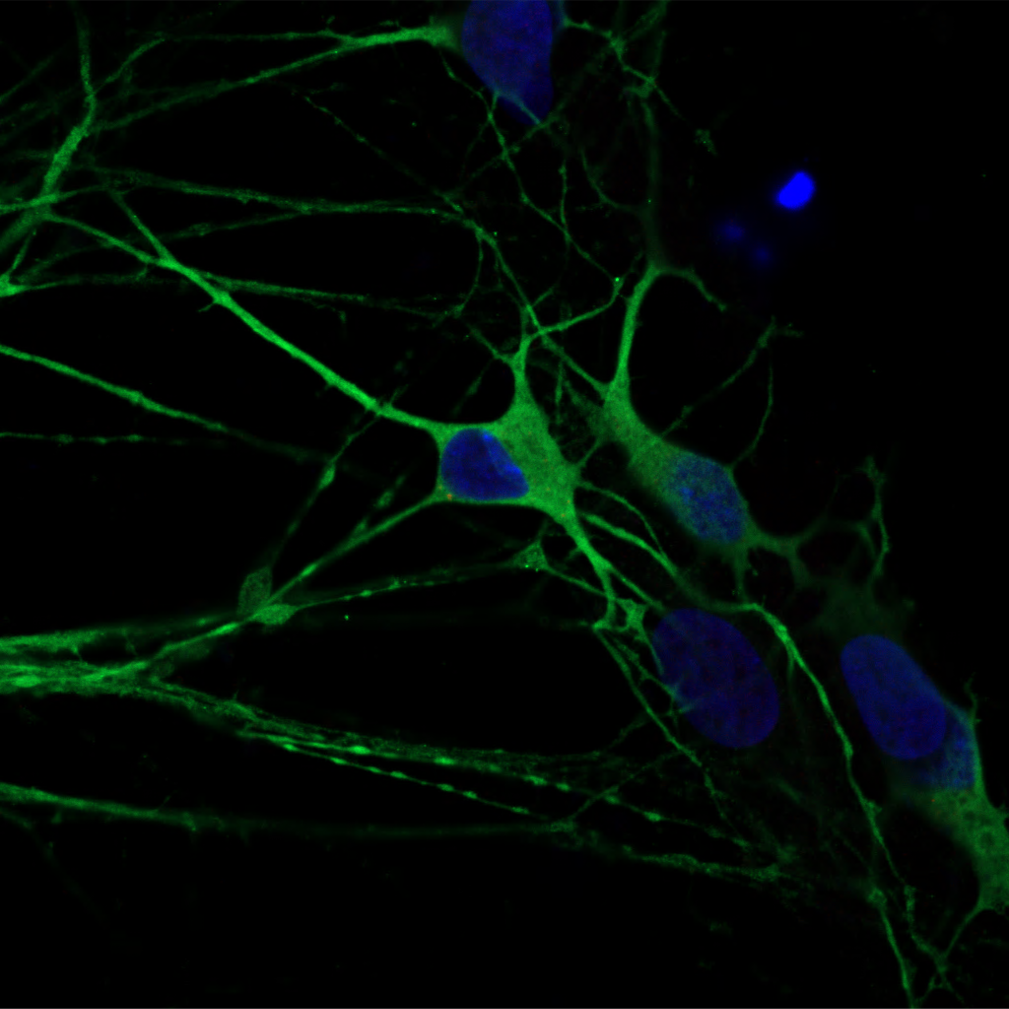

Supplement: Supplementary file 9 — Source data Fig. 5 [file 44321_2025_323_MOESM9_ESM.zip › Figure 5/5I/SOD1.tif]

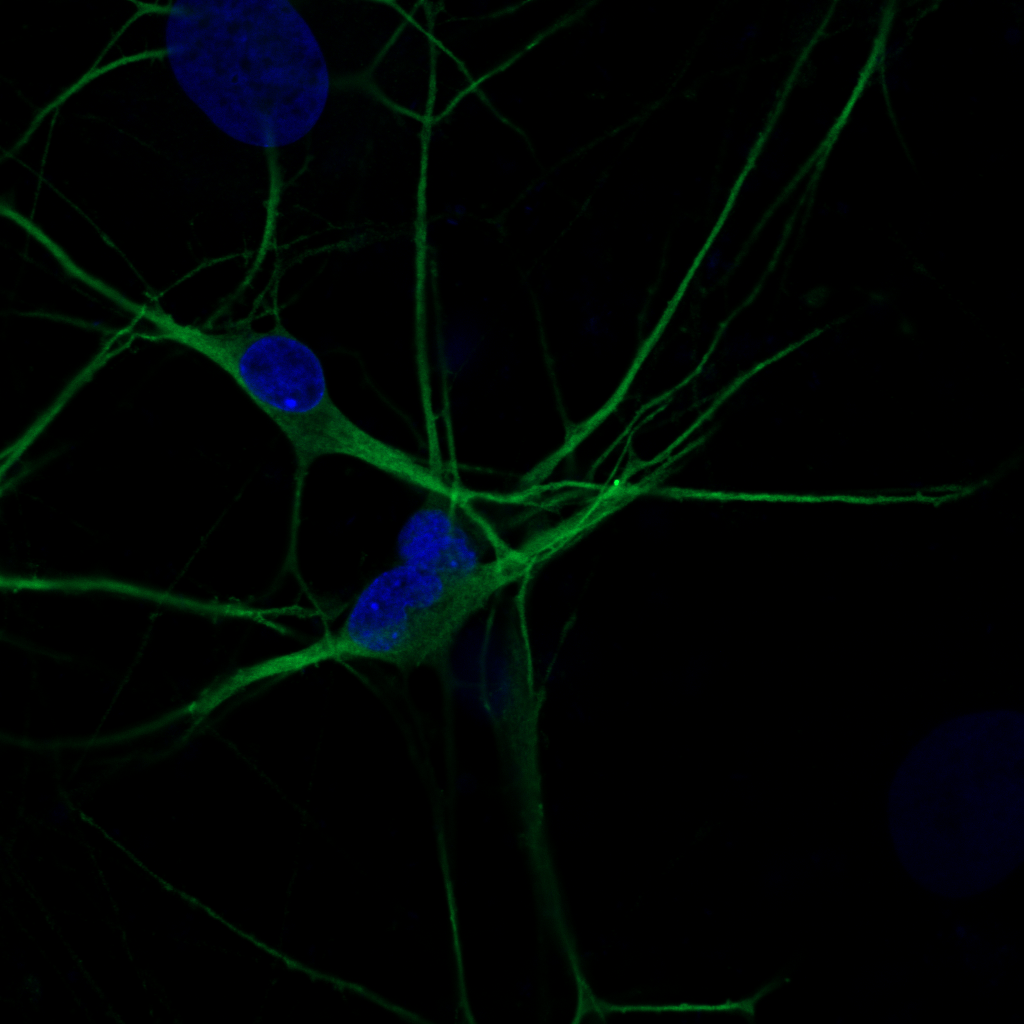

Supplement: Supplementary file 9 — Source data Fig. 5 [file 44321_2025_323_MOESM9_ESM.zip › Figure 5/5I/SOD1-ISO.tif]

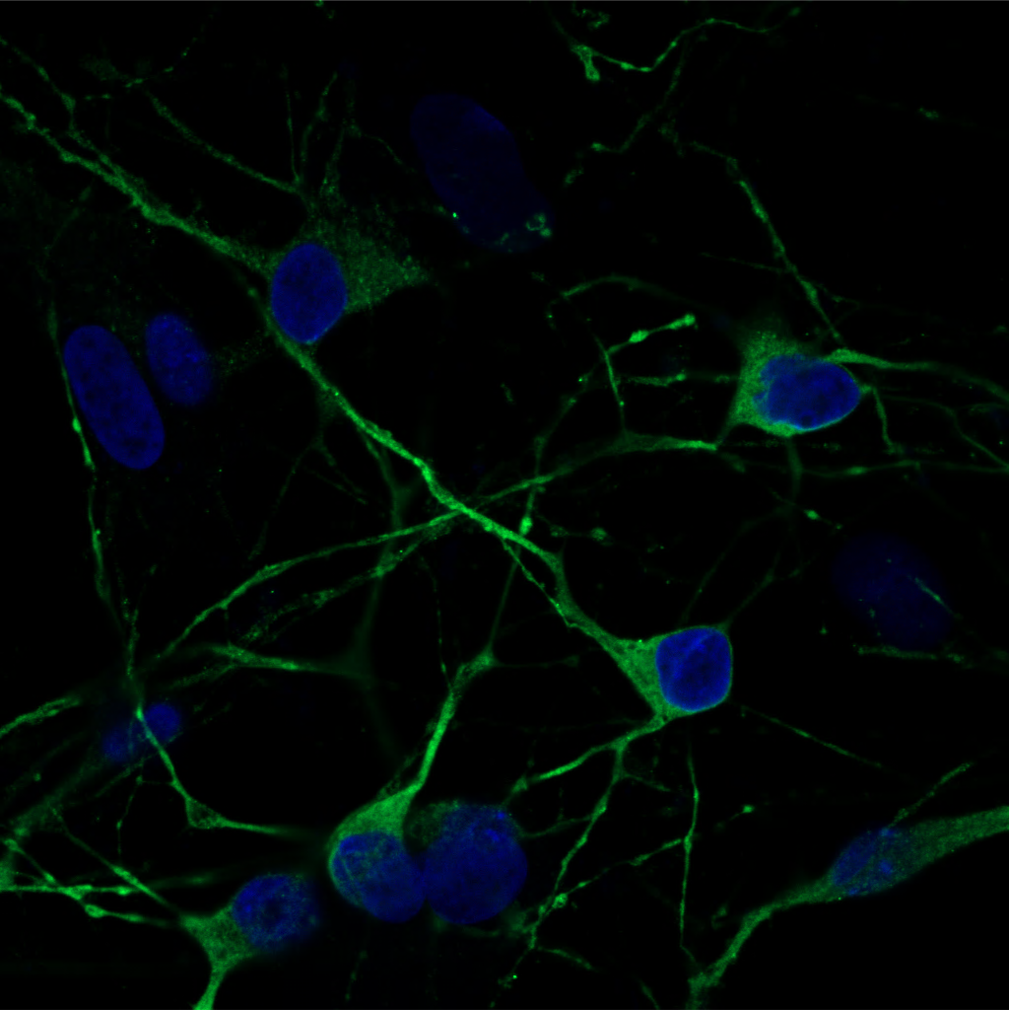

Supplement: Supplementary file 9 — Source data Fig. 5 [file 44321_2025_323_MOESM9_ESM.zip › Figure 5/5I/TDP-43.tif]

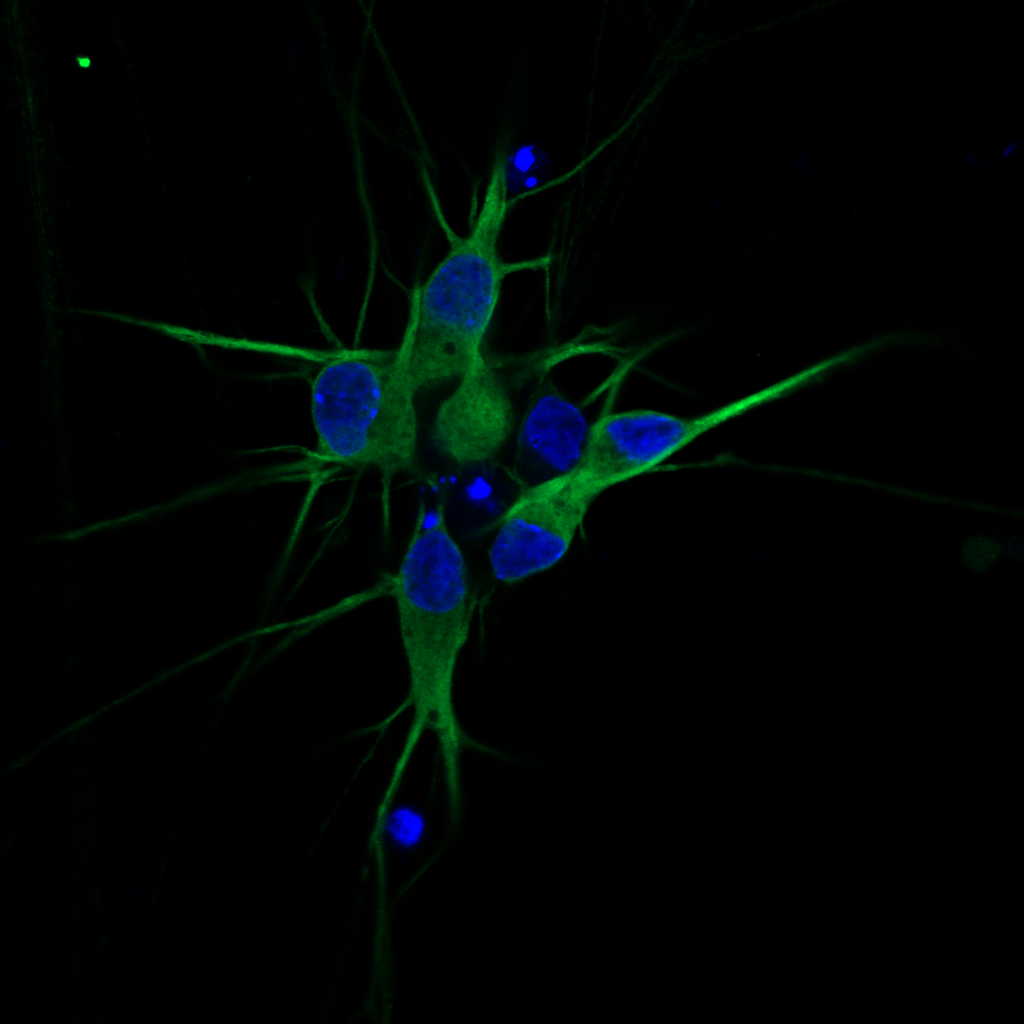

Supplement: Supplementary file 9 — Source data Fig. 5 [file 44321_2025_323_MOESM9_ESM.zip › Figure 5/5I/TDP43-ISO.tif]

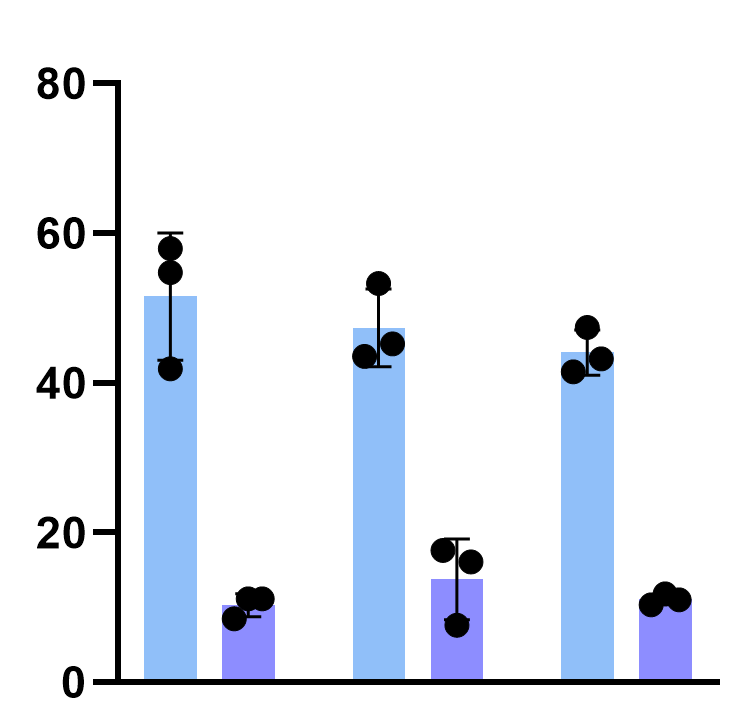

Supplement: Supplementary file 9 — Source data Fig. 5 [file 44321_2025_323_MOESM9_ESM.zip › Figure 5/5J/%BEADS.tif]

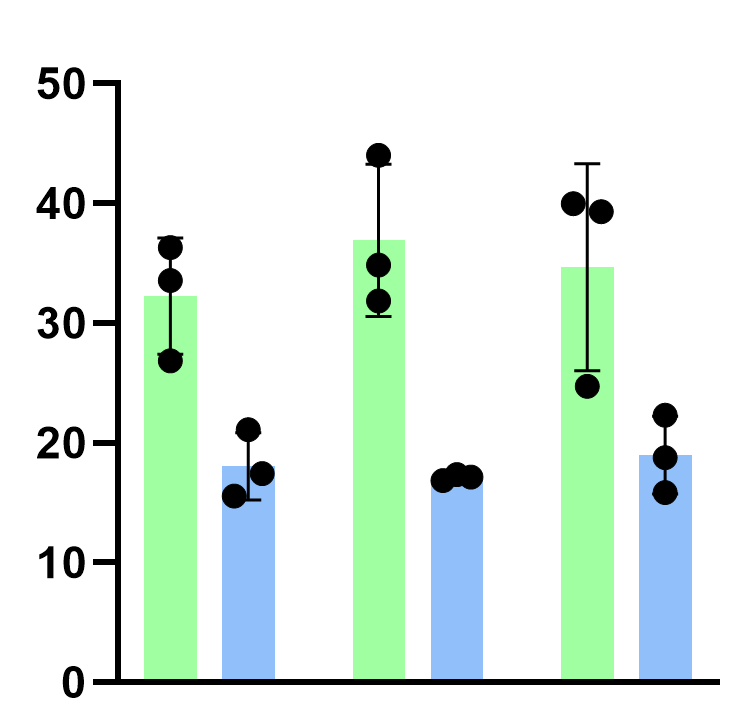

Supplement: Supplementary file 9 — Source data Fig. 5 [file 44321_2025_323_MOESM9_ESM.zip › Figure 5/5K/area.tif]

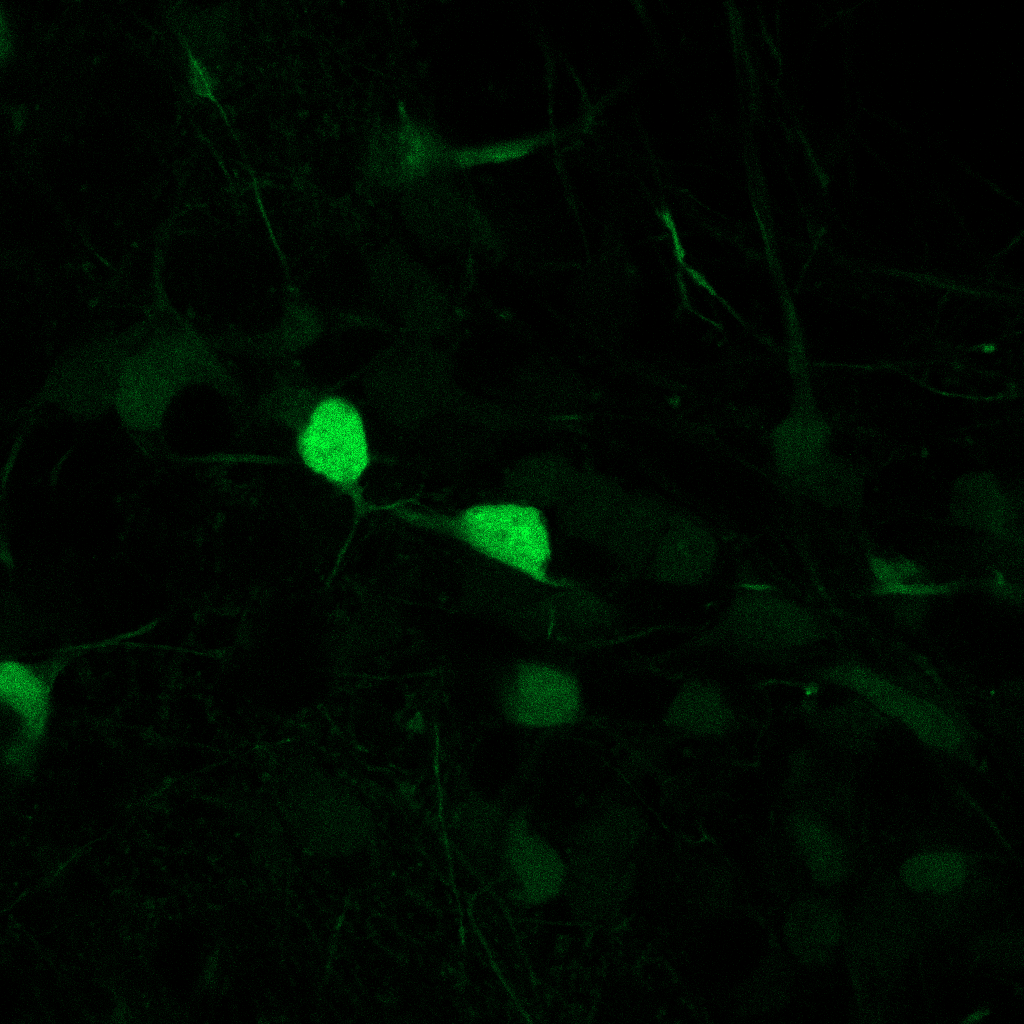

Supplement: Supplementary file 9 — Source data Fig. 5 [file 44321_2025_323_MOESM9_ESM.zip › Figure 5/5L/C9-ISO.tif]

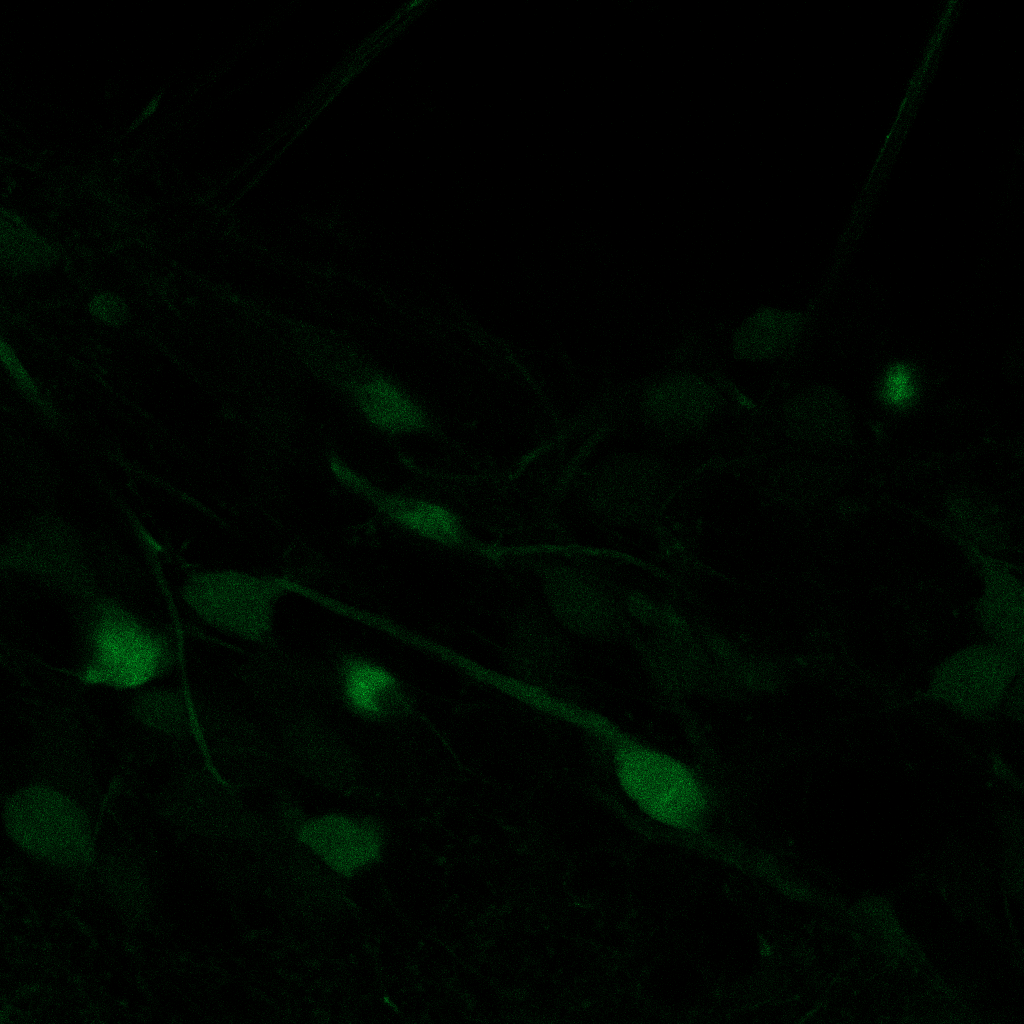

Supplement: Supplementary file 9 — Source data Fig. 5 [file 44321_2025_323_MOESM9_ESM.zip › Figure 5/5L/C9-VEH.tif]

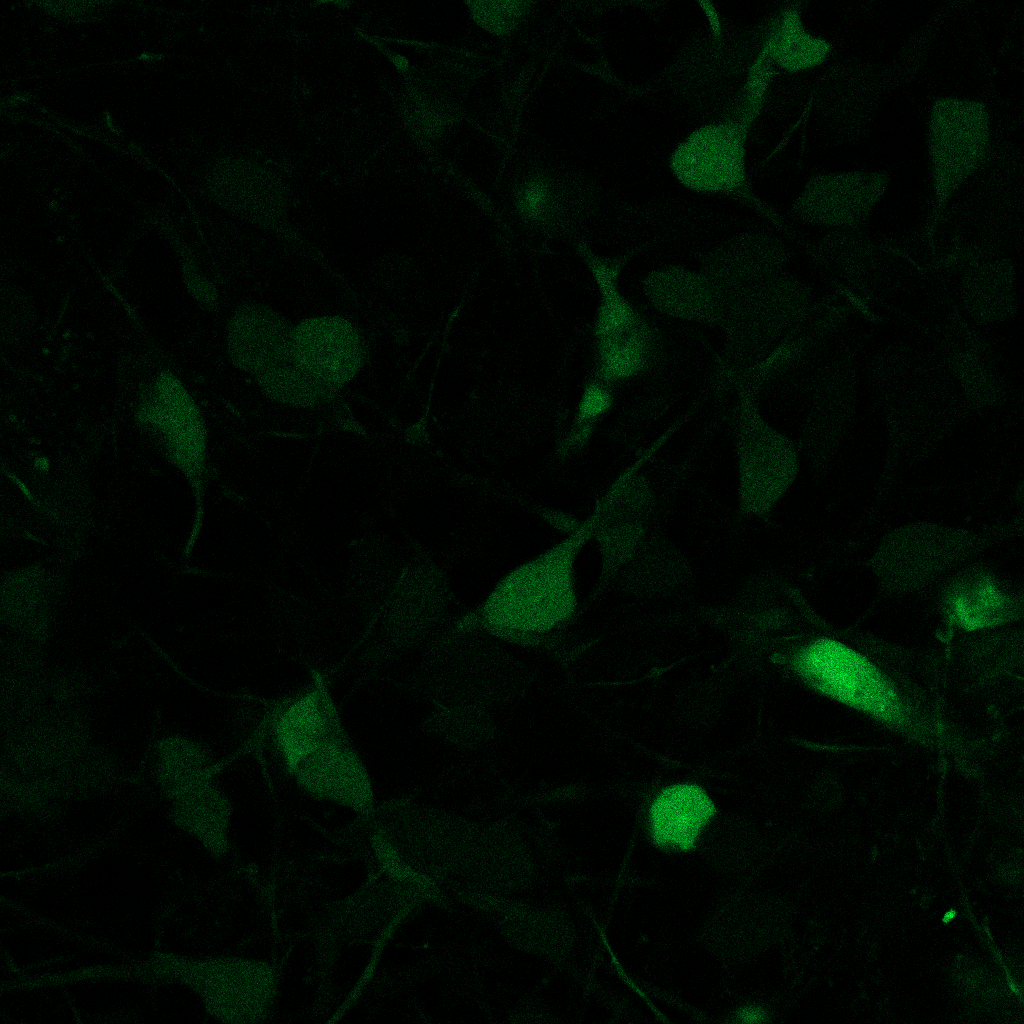

Supplement: Supplementary file 9 — Source data Fig. 5 [file 44321_2025_323_MOESM9_ESM.zip › Figure 5/5L/SOD1-ISO.tif]

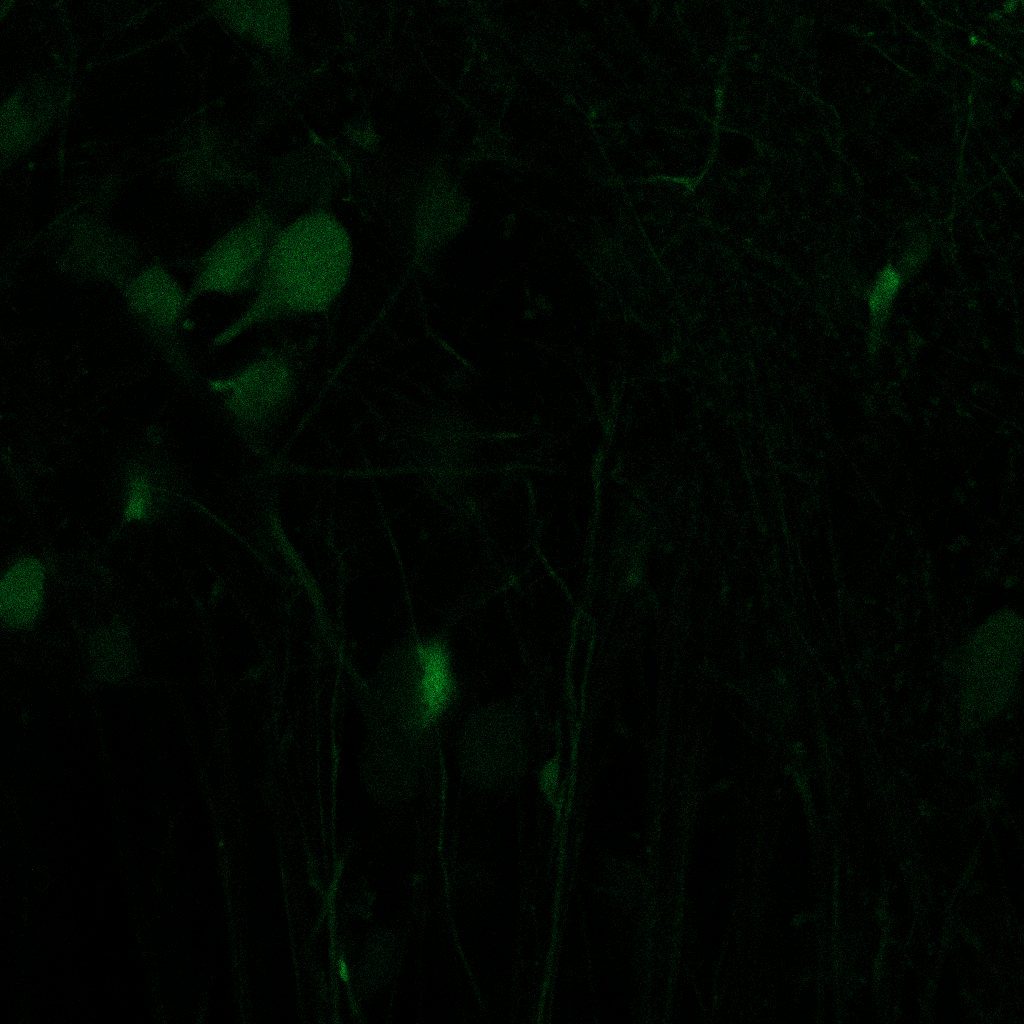

Supplement: Supplementary file 9 — Source data Fig. 5 [file 44321_2025_323_MOESM9_ESM.zip › Figure 5/5L/SOD1-VEH.tif]

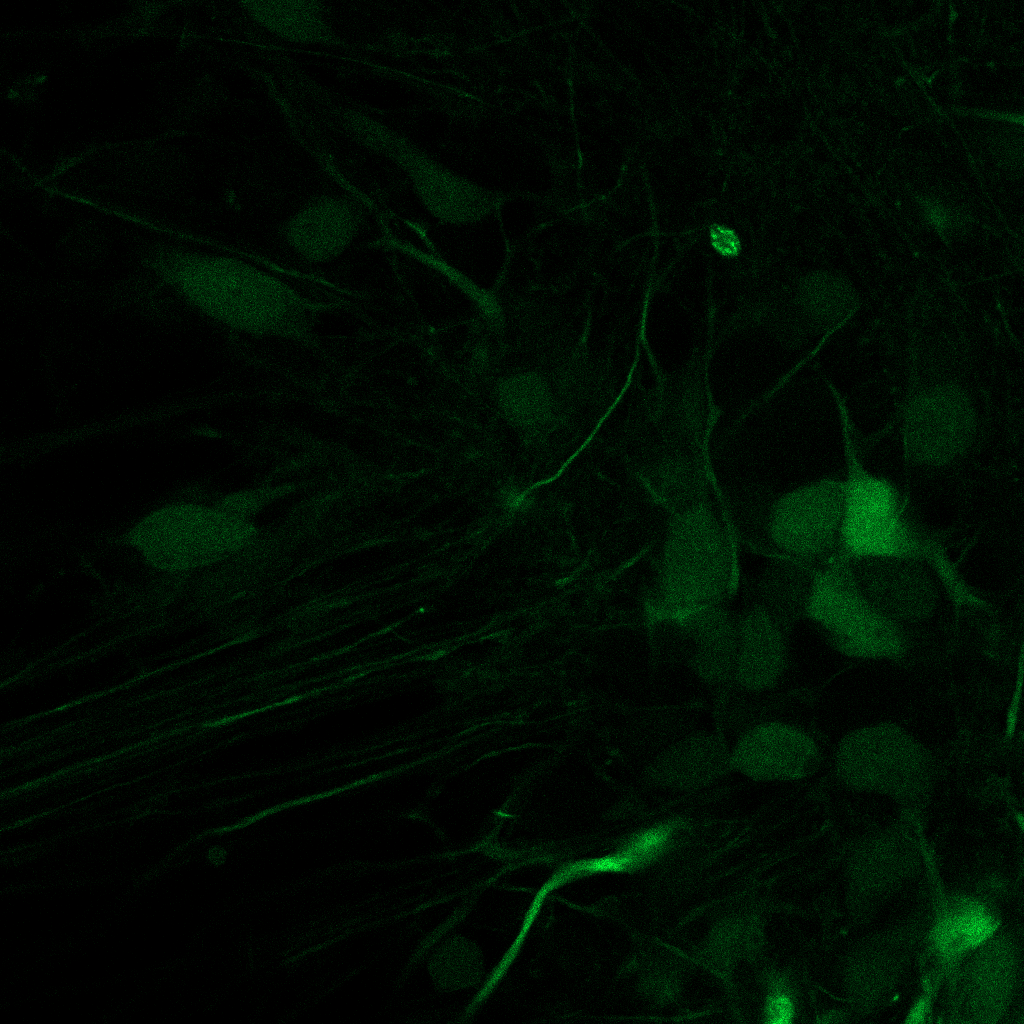

Supplement: Supplementary file 9 — Source data Fig. 5 [file 44321_2025_323_MOESM9_ESM.zip › Figure 5/5L/TDP-43-ISO.tif]

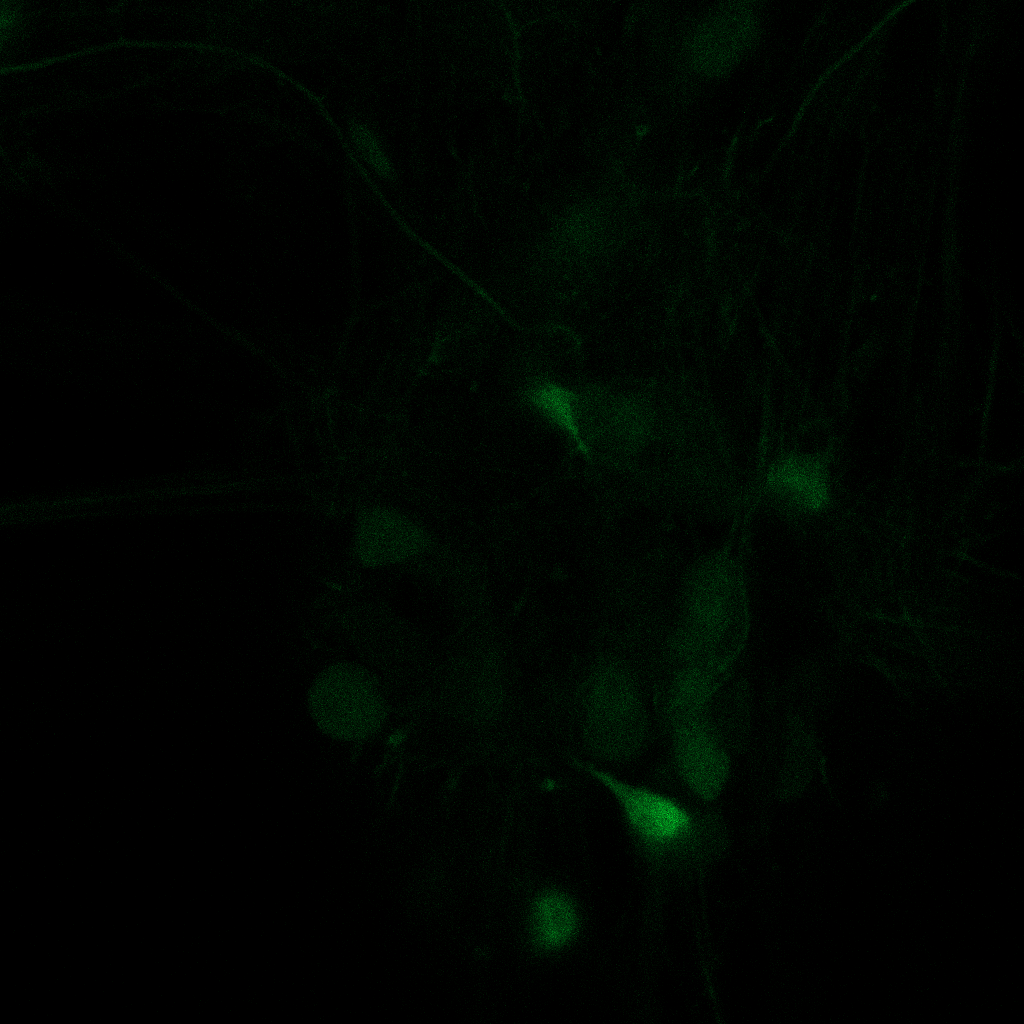

Supplement: Supplementary file 9 — Source data Fig. 5 [file 44321_2025_323_MOESM9_ESM.zip › Figure 5/5L/TDP-43-VEH.tif]

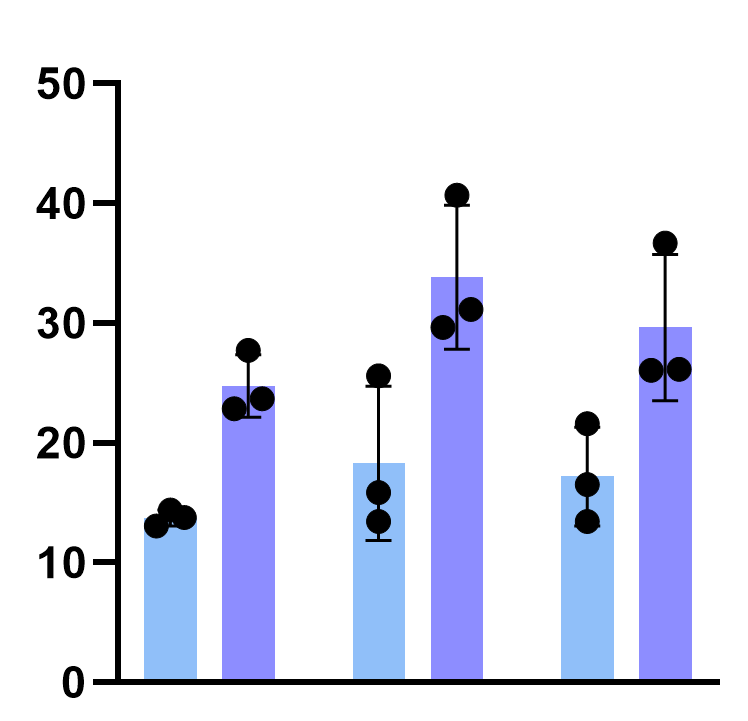

Supplement: Supplementary file 9 — Source data Fig. 5 [file 44321_2025_323_MOESM9_ESM.zip › Figure 5/5M/iso area.tif]

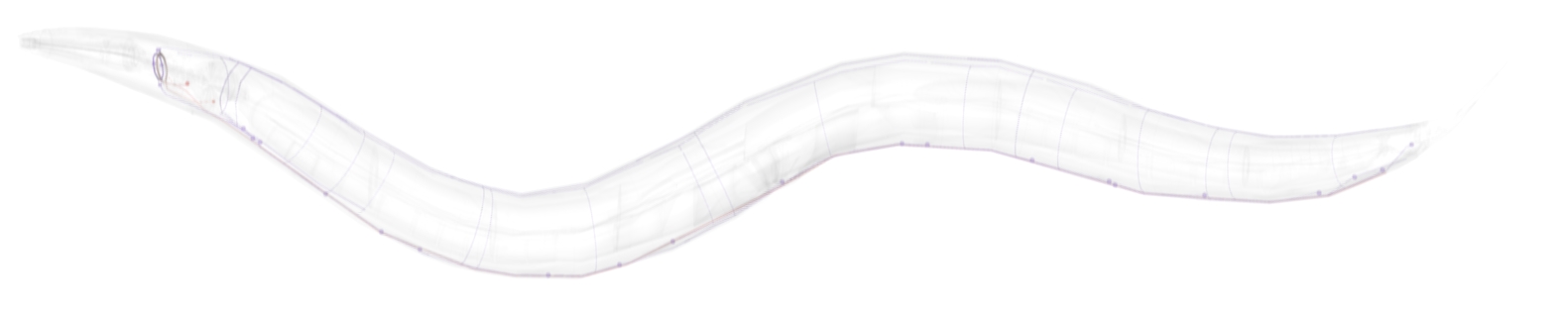

Supplement: Supplementary file 10 — Source data Fig. 6 [file 44321_2025_323_MOESM10_ESM.zip › Figure 6/6A/from wormbase WBGene00006762.jpg]

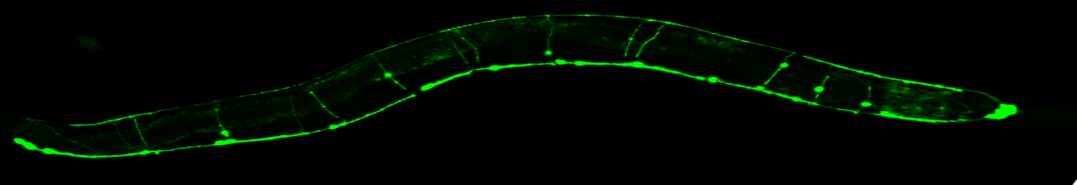

Supplement: Supplementary file 10 — Source data Fig. 6 [file 44321_2025_323_MOESM10_ESM.zip › Figure 6/6A/UNC25 GFP.tif]

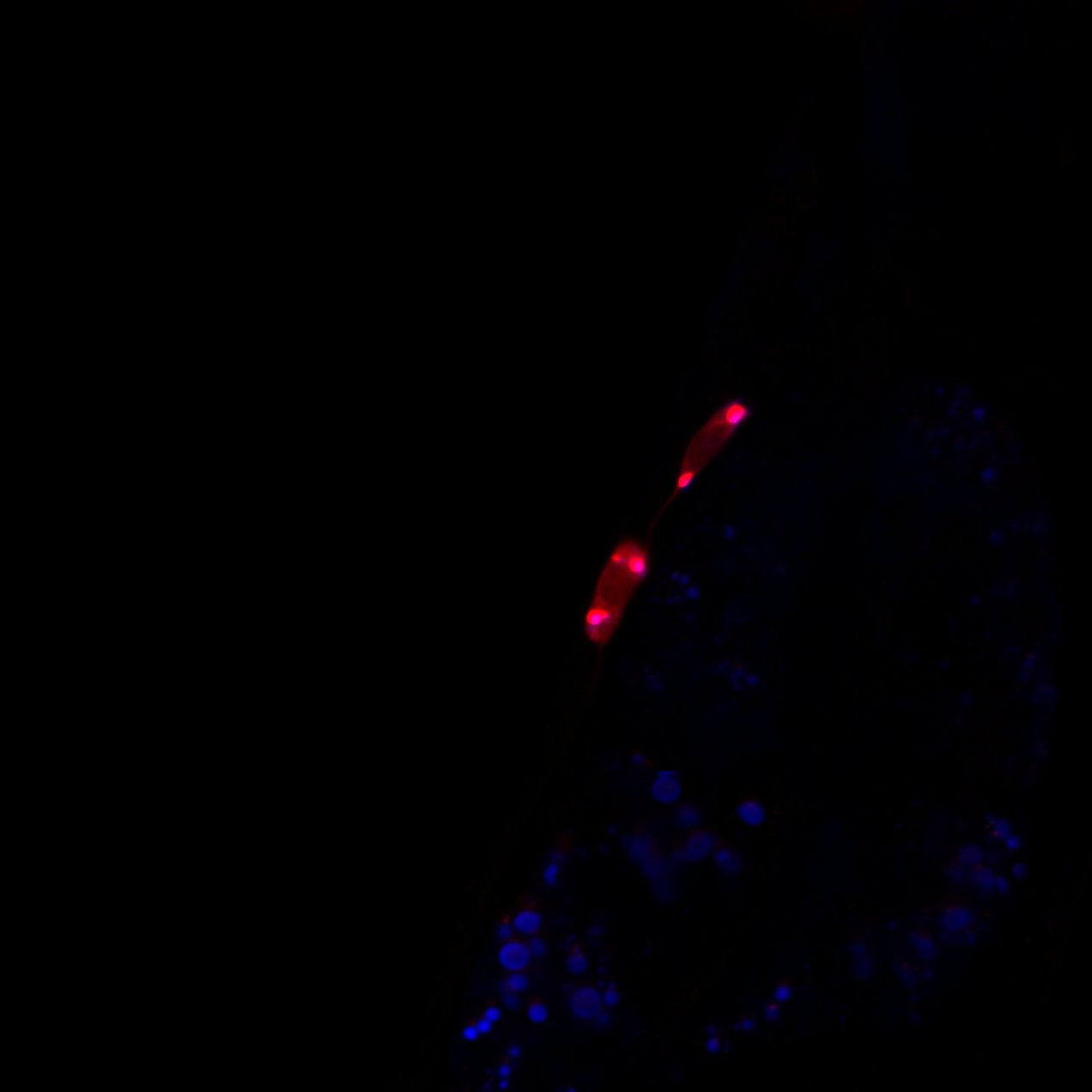

Supplement: Supplementary file 10 — Source data Fig. 6 [file 44321_2025_323_MOESM10_ESM.zip › Figure 6/6B-C/ISO+G93A/ISO+G93A Merge.jpg]

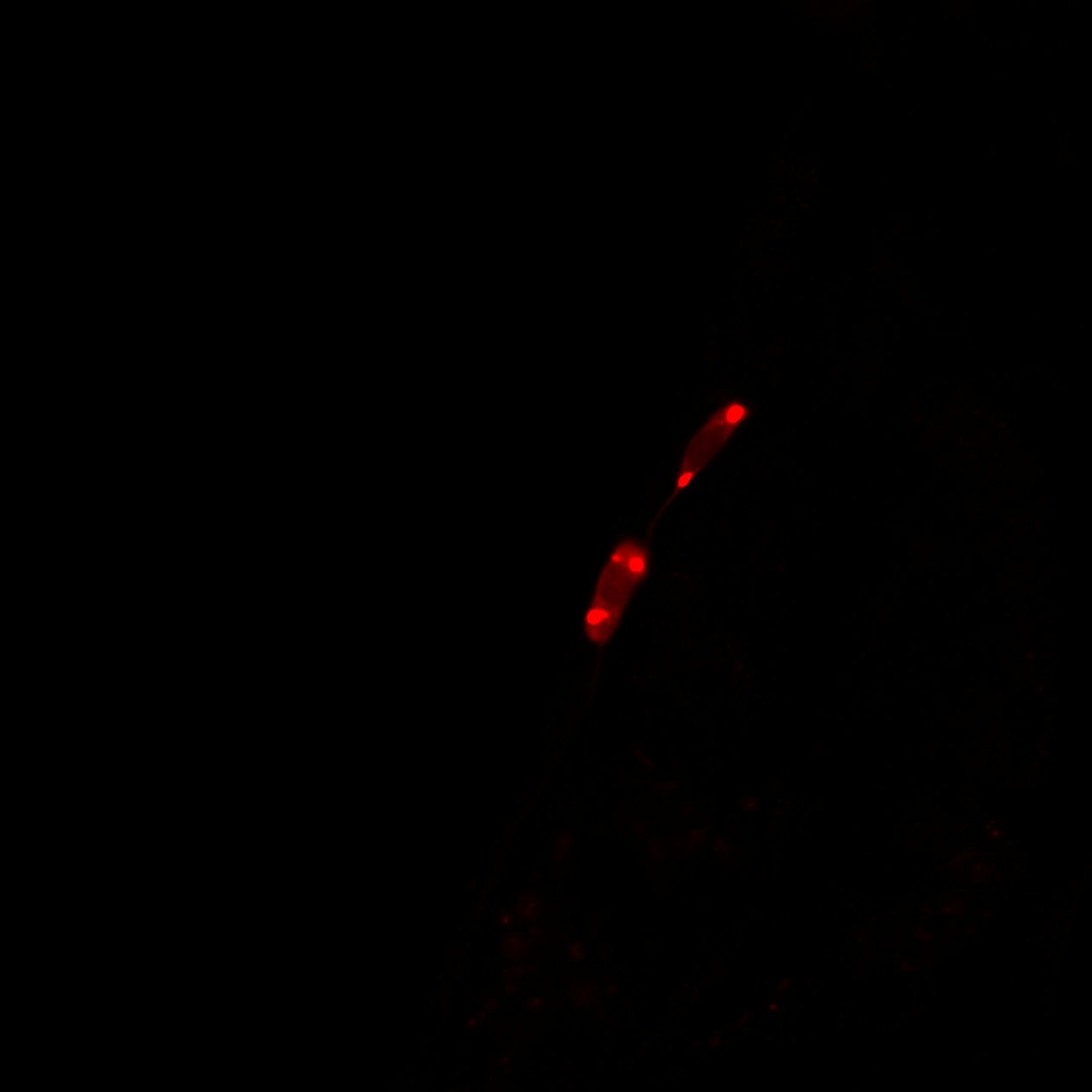

Supplement: Supplementary file 10 — Source data Fig. 6 [file 44321_2025_323_MOESM10_ESM.zip › Figure 6/6B-C/ISO+G93A/ISO+G93A-lgg-1-Dsred.jpg]

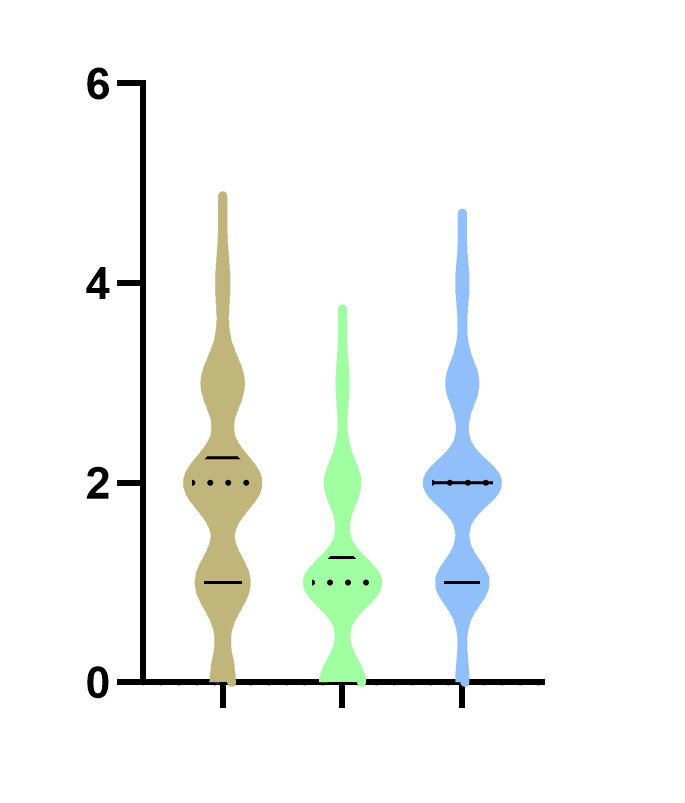

Supplement: Supplementary file 10 — Source data Fig. 6 [file 44321_2025_323_MOESM10_ESM.zip › Figure 6/6B-C/LGG-1-DCT-1.tif]

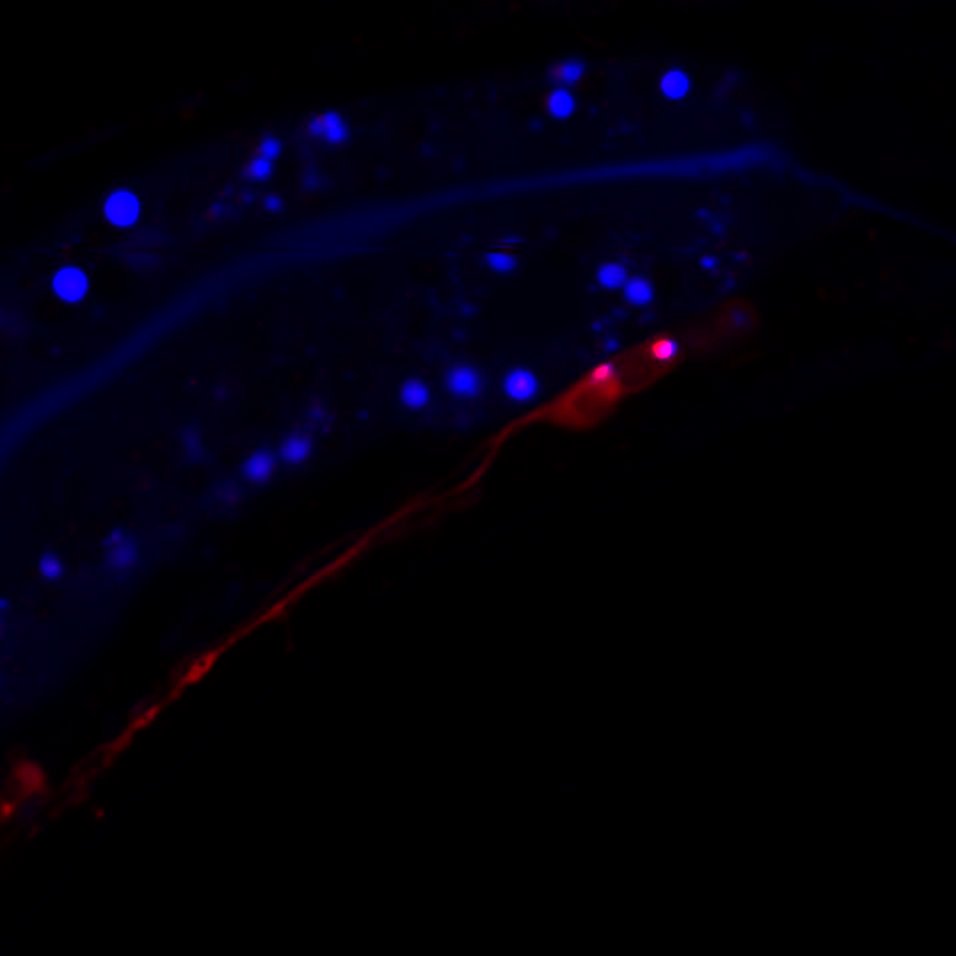

Supplement: Supplementary file 10 — Source data Fig. 6 [file 44321_2025_323_MOESM10_ESM.zip › Figure 6/6B-C/SOD1 G93A worm/G93A Merge.jpg]

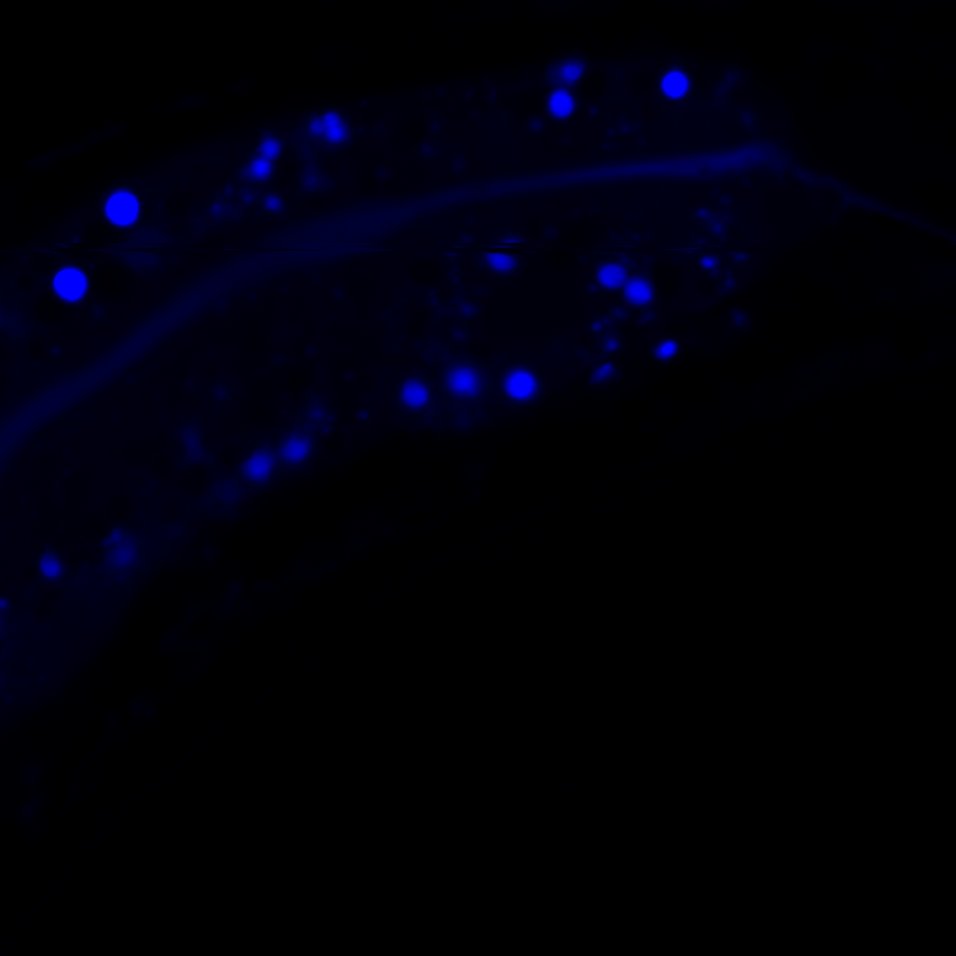

Supplement: Supplementary file 10 — Source data Fig. 6 [file 44321_2025_323_MOESM10_ESM.zip › Figure 6/6B-C/SOD1 G93A worm/G93A-dct-1-BFP.jpg]

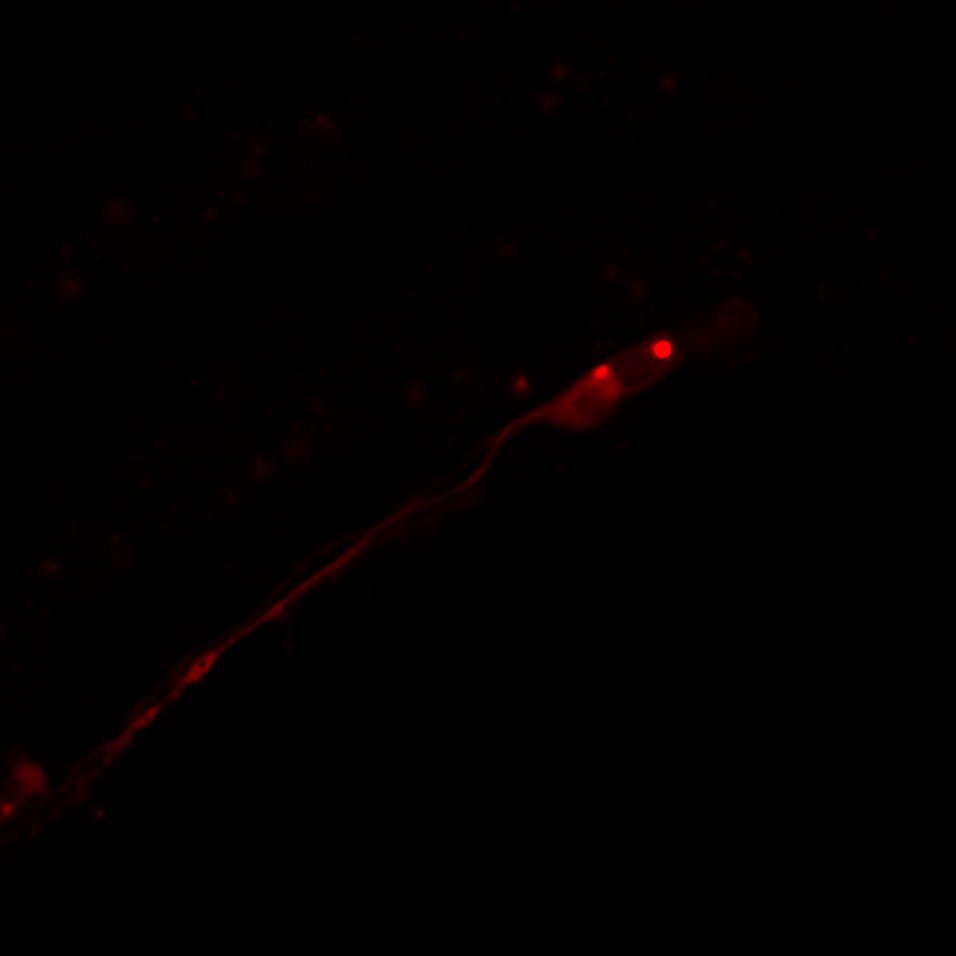

Supplement: Supplementary file 10 — Source data Fig. 6 [file 44321_2025_323_MOESM10_ESM.zip › Figure 6/6B-C/SOD1 G93A worm/G93A-lgg-1-Dsred.jpg]

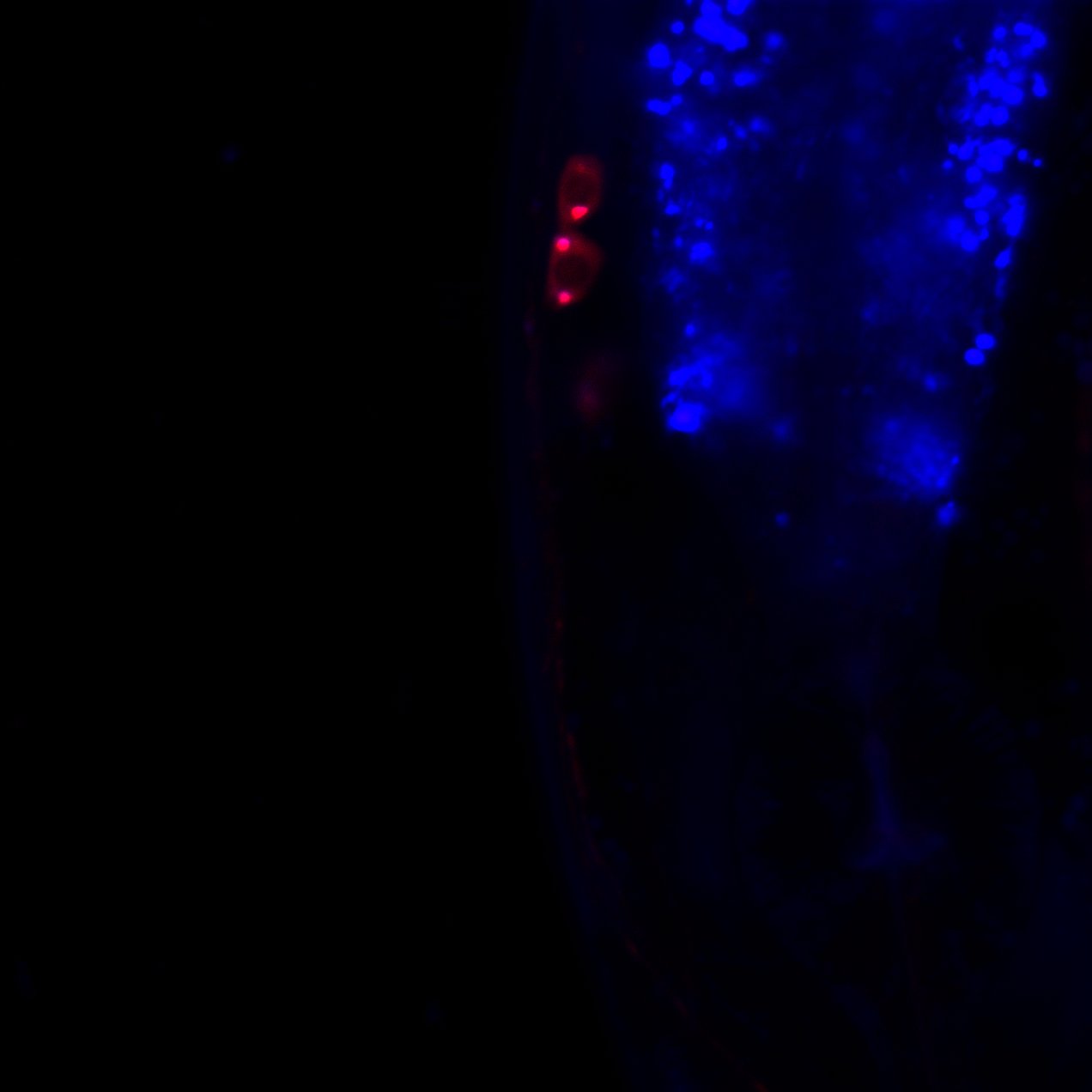

Supplement: Supplementary file 10 — Source data Fig. 6 [file 44321_2025_323_MOESM10_ESM.zip › Figure 6/6B-C/WT worm/WT Merge.jpg]

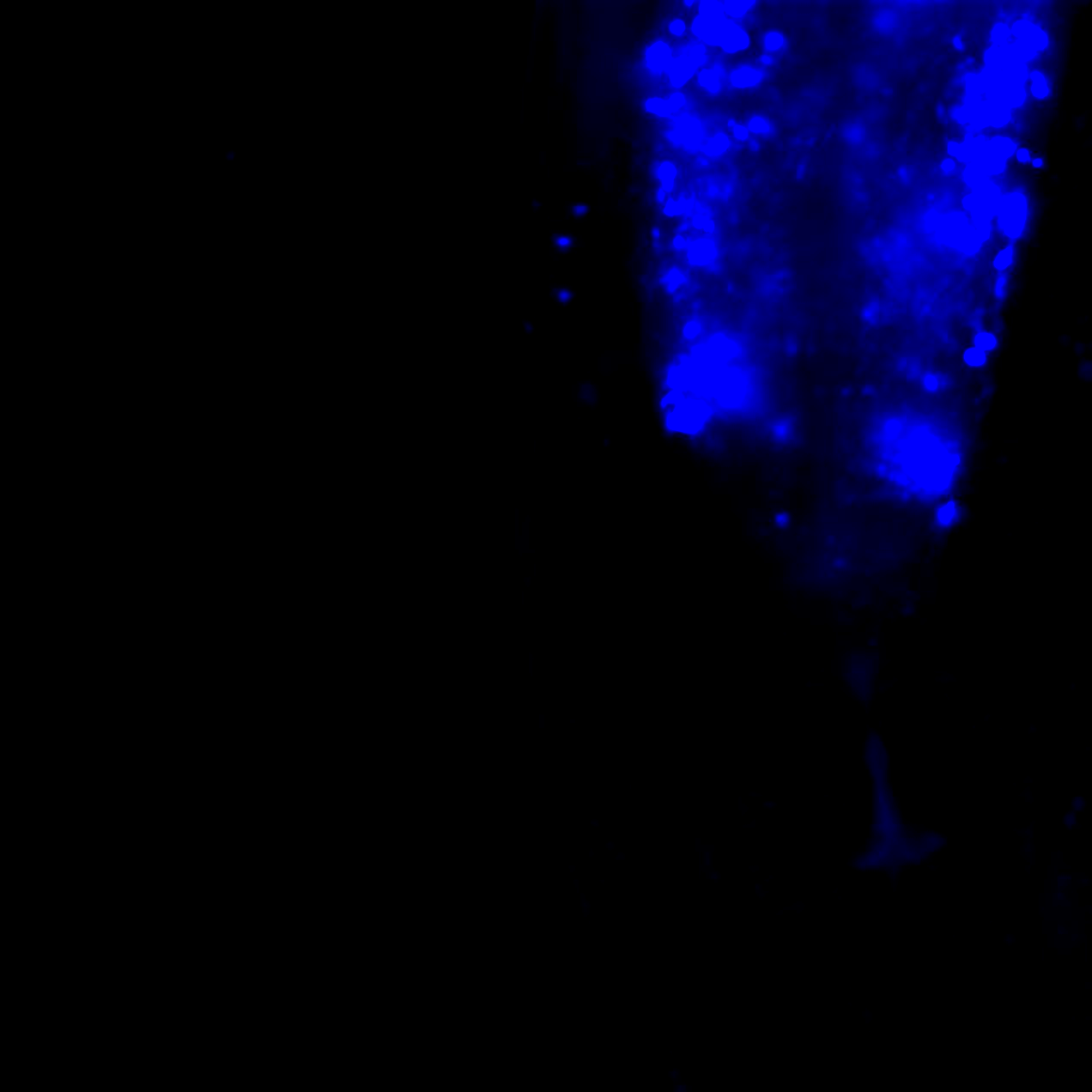

Supplement: Supplementary file 10 — Source data Fig. 6 [file 44321_2025_323_MOESM10_ESM.zip › Figure 6/6B-C/WT worm/WT-dct-1-BFP.tif]

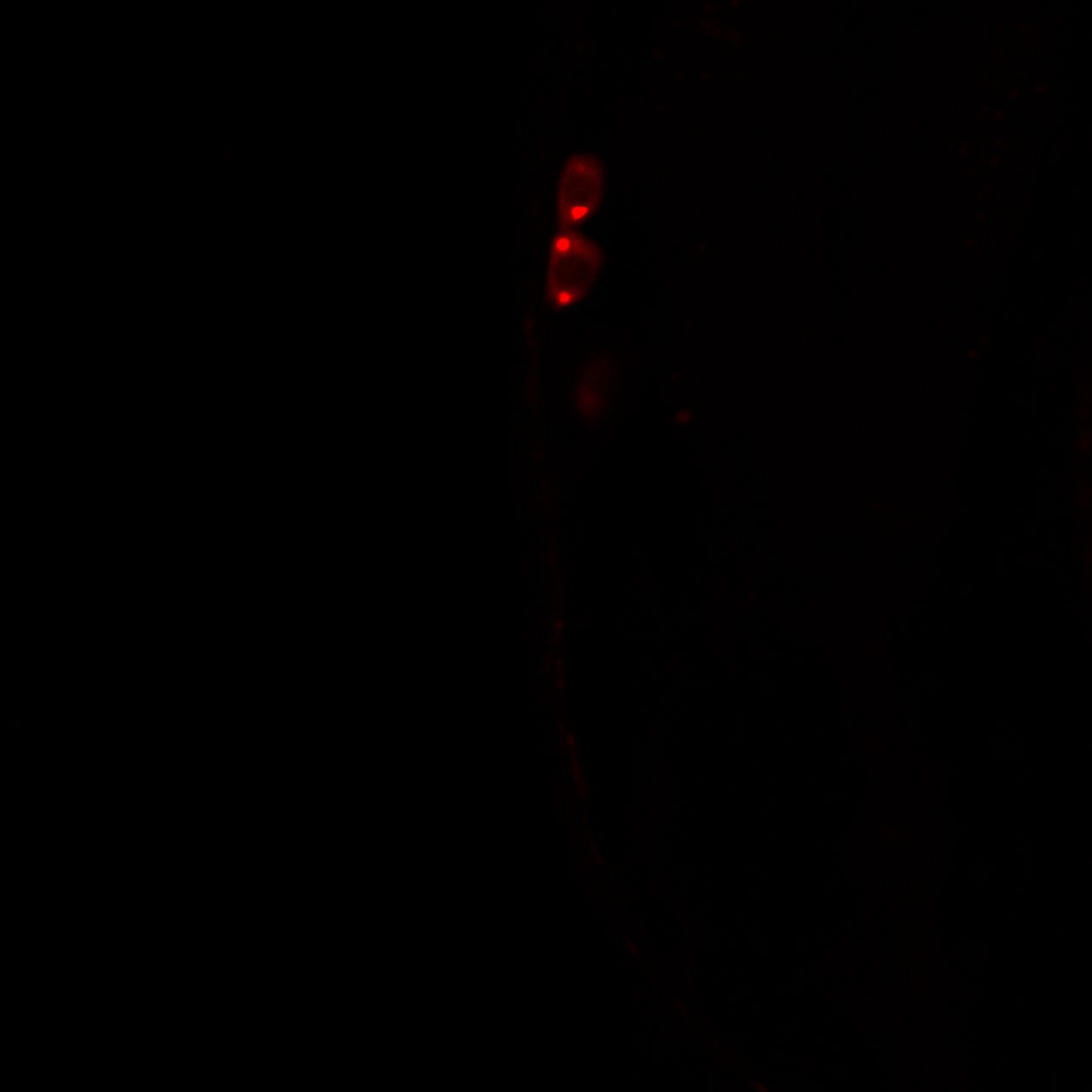

Supplement: Supplementary file 10 — Source data Fig. 6 [file 44321_2025_323_MOESM10_ESM.zip › Figure 6/6B-C/WT worm/WT-lgg-1-Dsred.jpg]

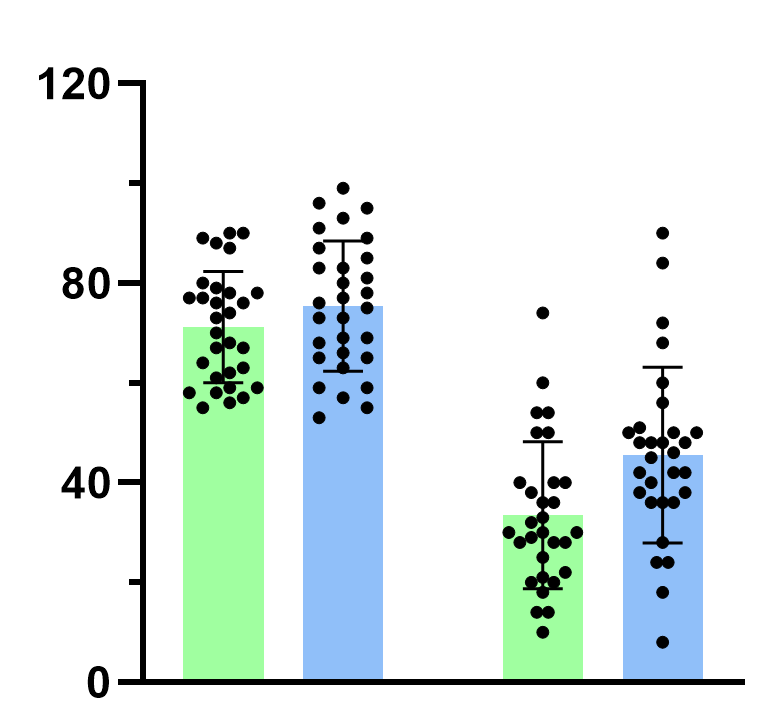

Supplement: Supplementary file 10 — Source data Fig. 6 [file 44321_2025_323_MOESM10_ESM.zip › Figure 6/6D/iso swimming.tif]

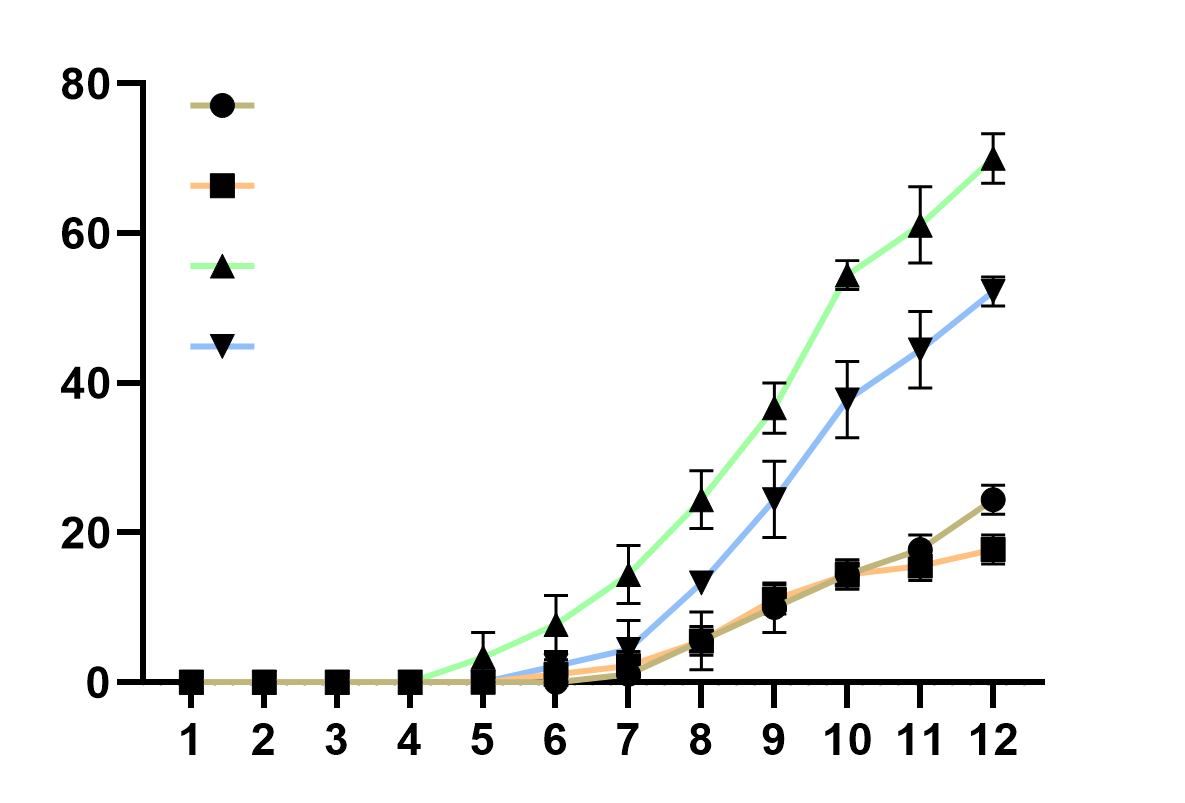

Supplement: Supplementary file 10 — Source data Fig. 6 [file 44321_2025_323_MOESM10_ESM.zip › Figure 6/6E/paralyzed.tif]

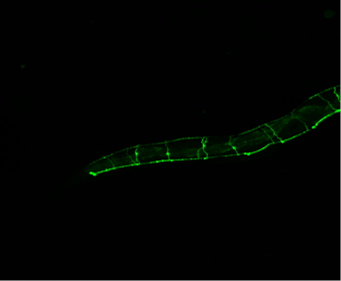

Supplement: Supplementary file 10 — Source data Fig. 6 [file 44321_2025_323_MOESM10_ESM.zip › Figure 6/6F/ISO-SOD1 G93A-LEFT.tif]

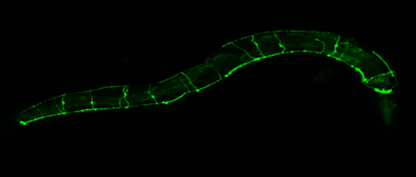

Supplement: Supplementary file 10 — Source data Fig. 6 [file 44321_2025_323_MOESM10_ESM.zip › Figure 6/6F/ISO-SOD1 G93A-Merge.tif]

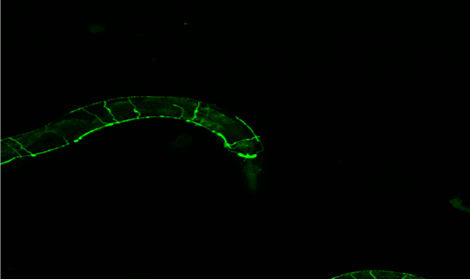

Supplement: Supplementary file 10 — Source data Fig. 6 [file 44321_2025_323_MOESM10_ESM.zip › Figure 6/6F/ISO-SOD1 G93A-RIGHT.tif]
