# Supplementary figures and images for: Isoginkgetin antagonizes ALS pathologies in its animal and patient iPSC models via PINK1-Parkin-dependent mitophagy (part 3 of 3)
Source: EMBO Mol Med. 2025 Oct 15;17(11):3139–73. doi: 10.1038/s44321-025-00323-2 (PMC12603167; doi:10.1038/s44321-025-00323-2)

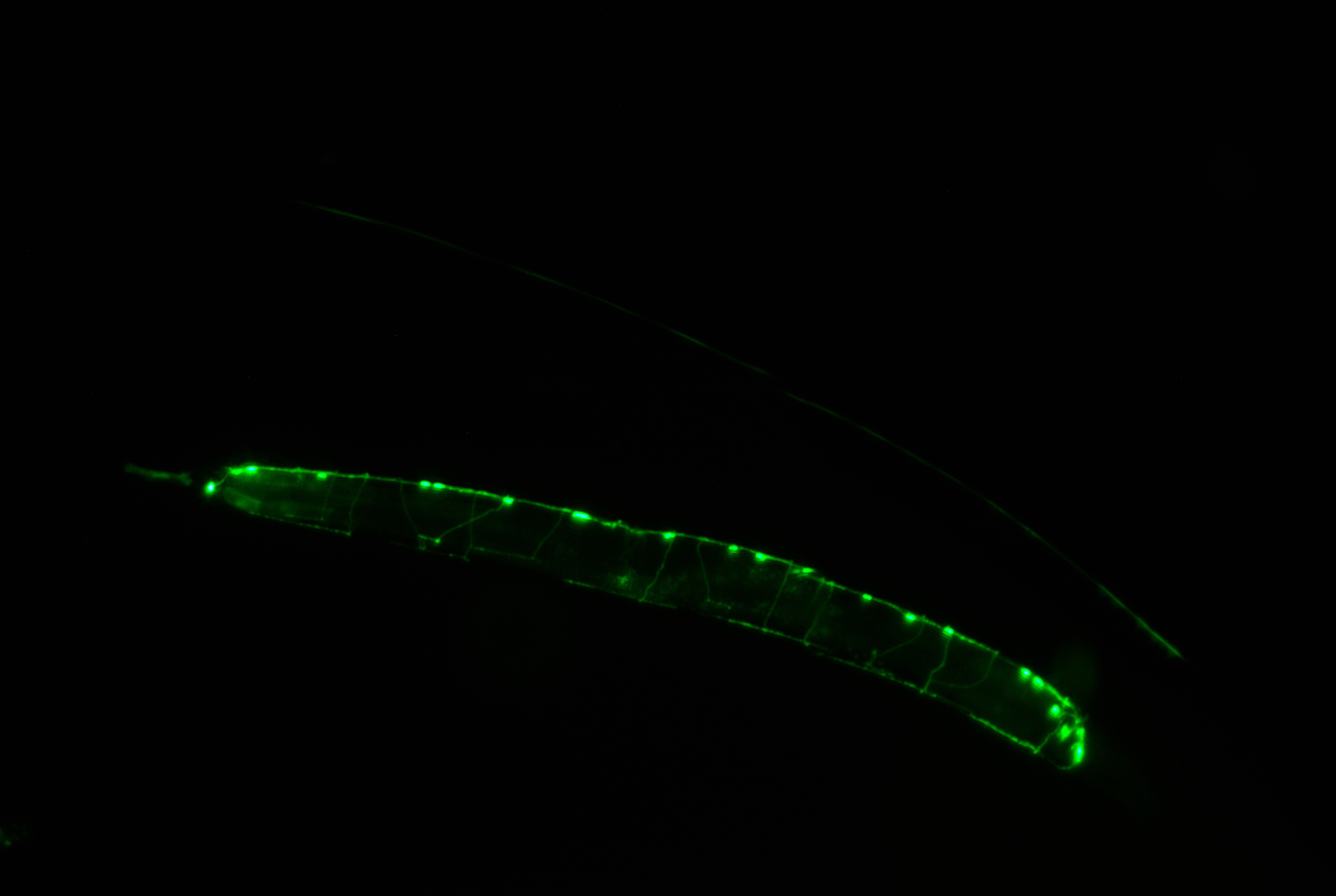

Supplement: Supplementary file 10 — Source data Fig. 6 [file 44321_2025_323_MOESM10_ESM.zip › Figure 6/6F/ISO-WT.jpg]

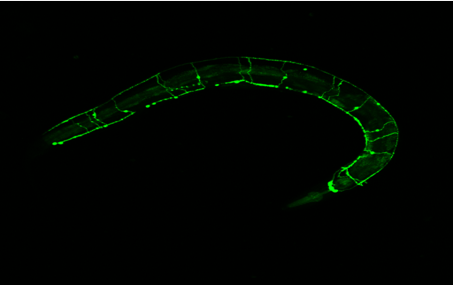

Supplement: Supplementary file 10 — Source data Fig. 6 [file 44321_2025_323_MOESM10_ESM.zip › Figure 6/6F/Veh-SOD1 G93A.tif]

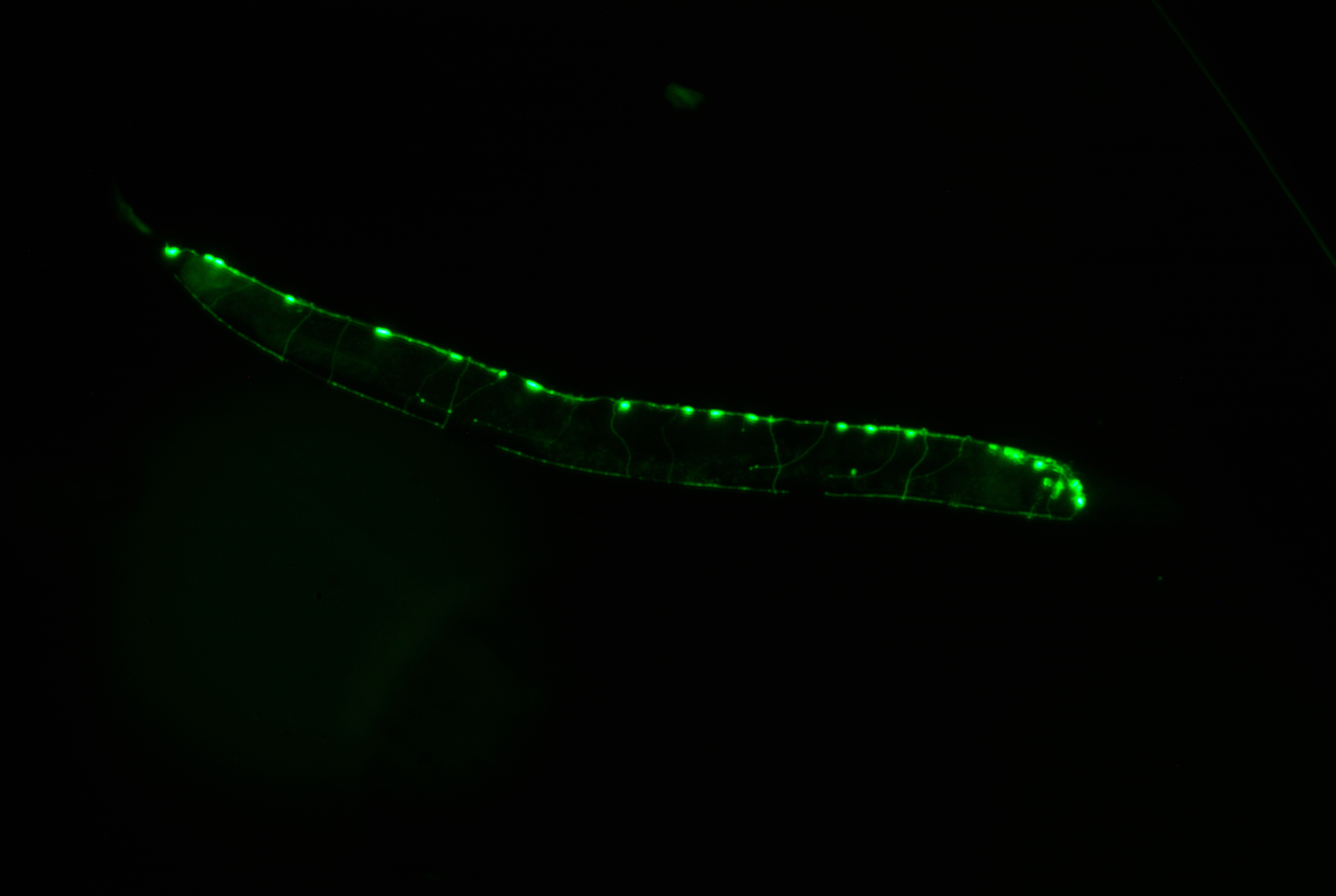

Supplement: Supplementary file 10 — Source data Fig. 6 [file 44321_2025_323_MOESM10_ESM.zip › Figure 6/6F/Veh-WT.jpg]

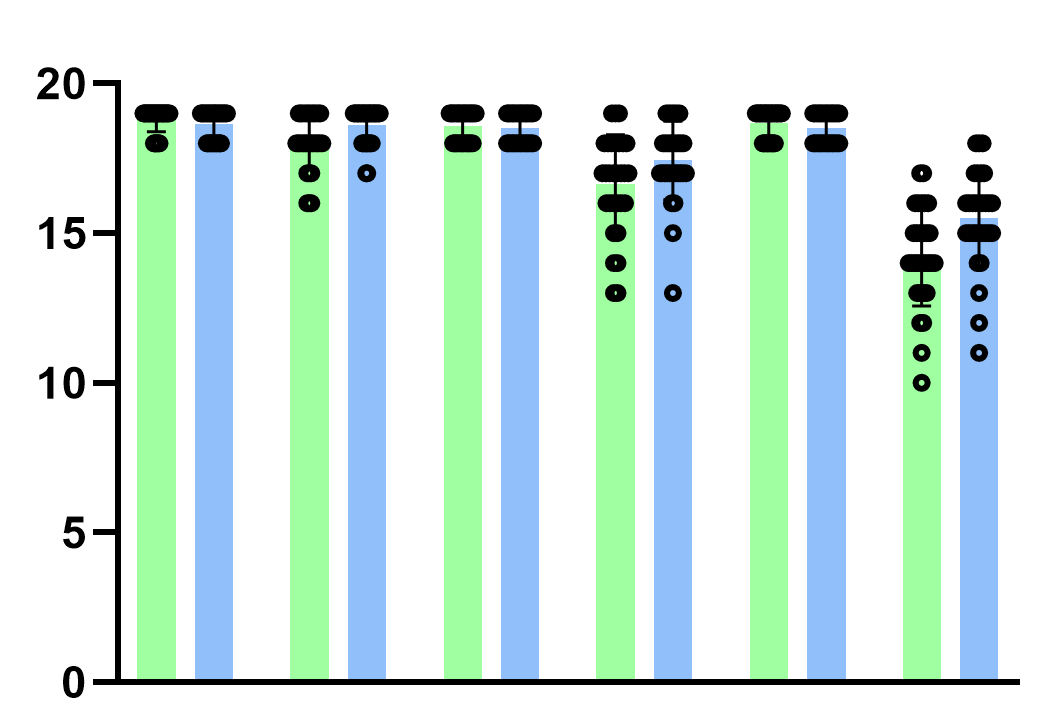

Supplement: Supplementary file 10 — Source data Fig. 6 [file 44321_2025_323_MOESM10_ESM.zip › Figure 6/6G/MNs.tif]

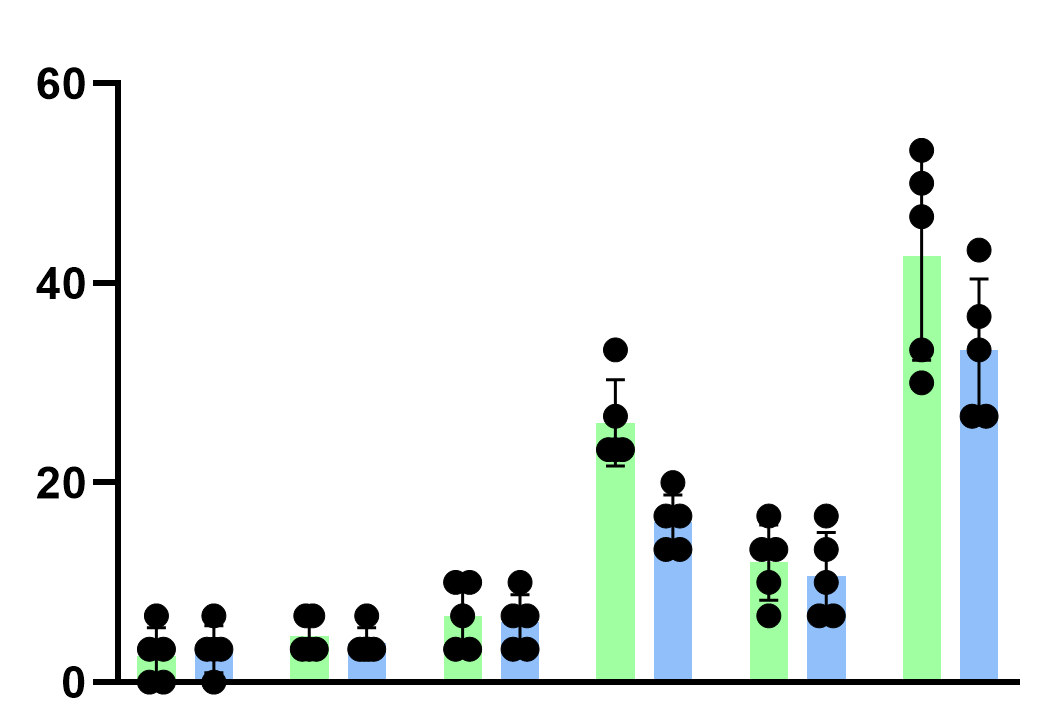

Supplement: Supplementary file 10 — Source data Fig. 6 [file 44321_2025_323_MOESM10_ESM.zip › Figure 6/6H/MN connection.tif]

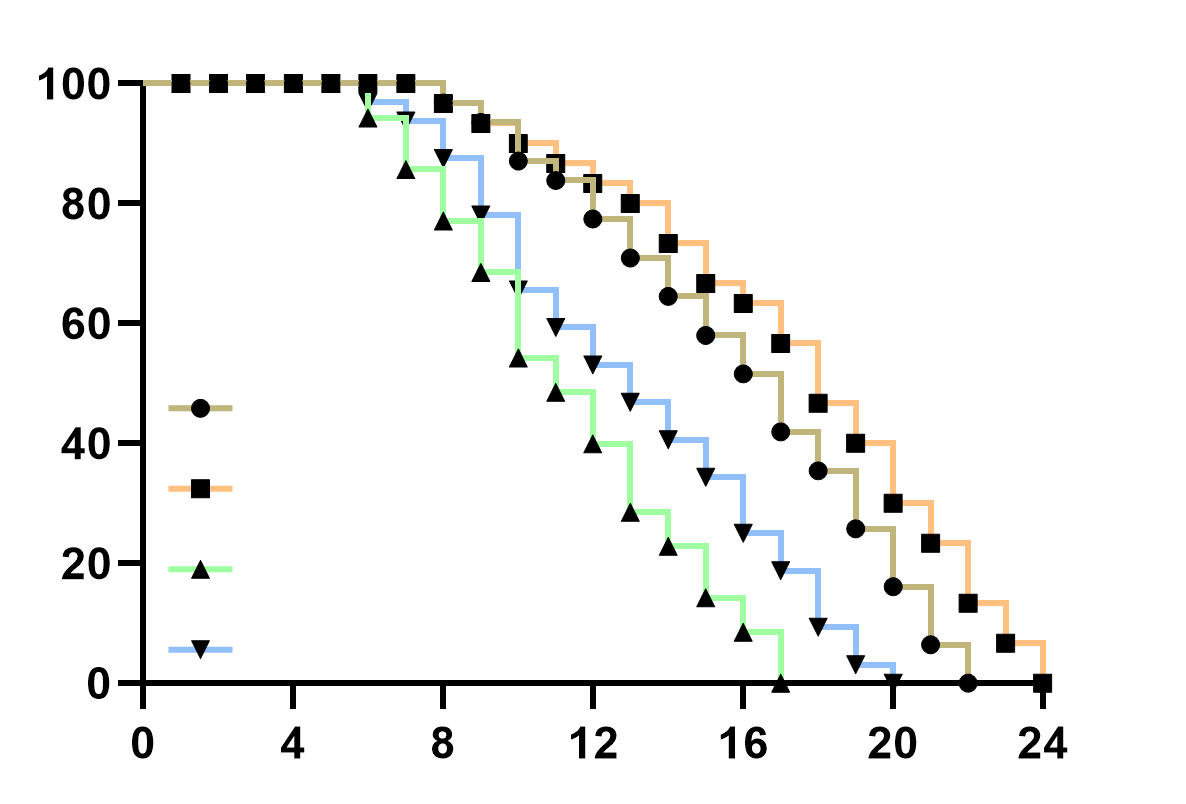

Supplement: Supplementary file 10 — Source data Fig. 6 [file 44321_2025_323_MOESM10_ESM.zip › Figure 6/6I/life spanlife span.tif]

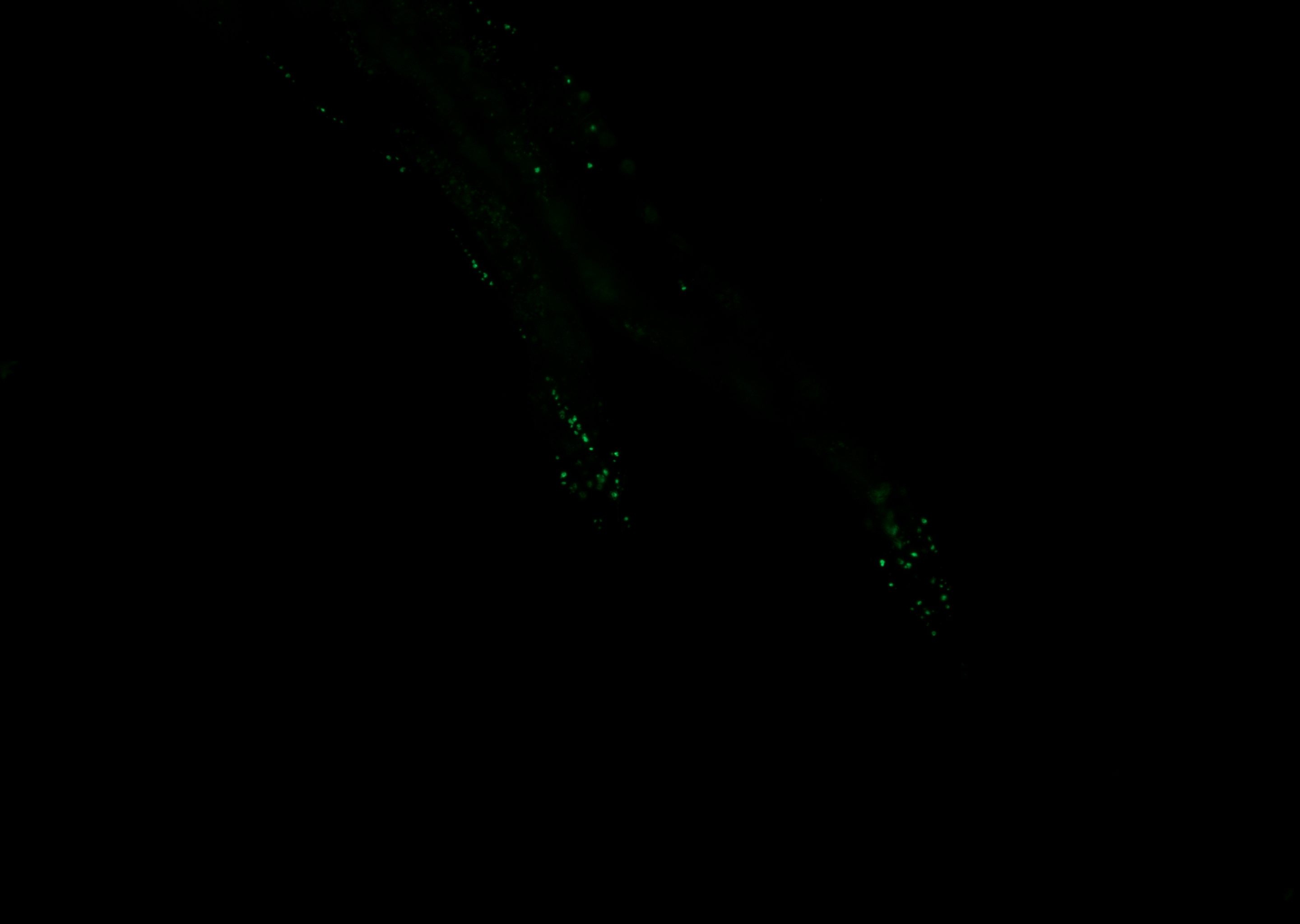

Supplement: Supplementary file 10 — Source data Fig. 6 [file 44321_2025_323_MOESM10_ESM.zip › Figure 6/6J/mtRosella-G93A-GFP.tif]

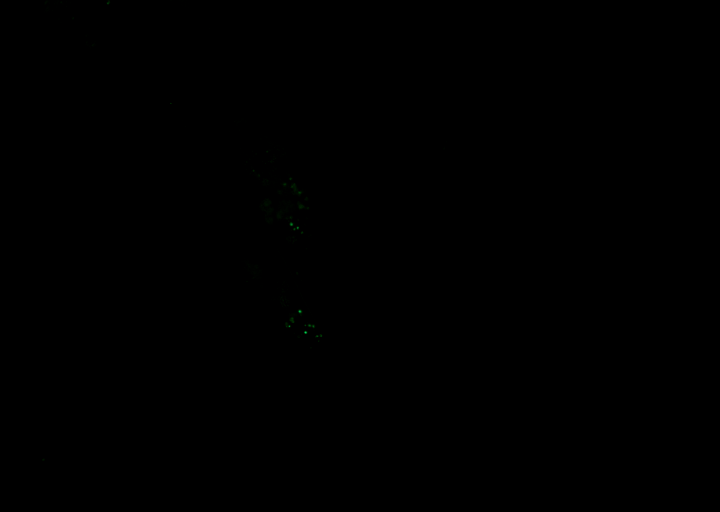

Supplement: Supplementary file 10 — Source data Fig. 6 [file 44321_2025_323_MOESM10_ESM.zip › Figure 6/6J/mtRosella-G93A-ISO-GFP.tif]

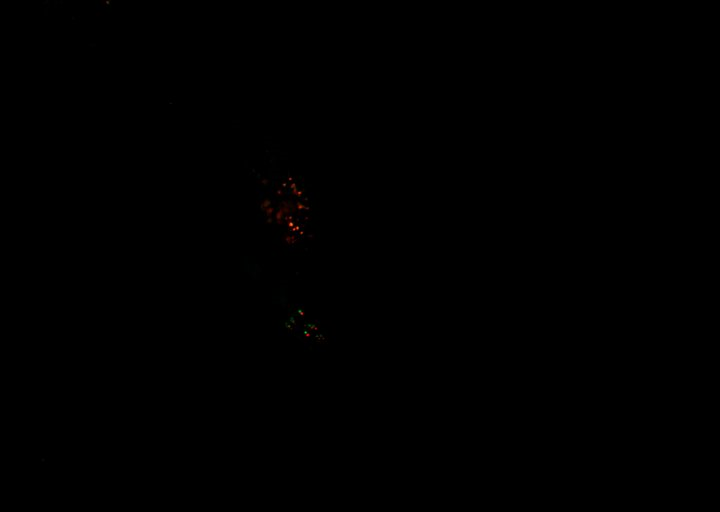

Supplement: Supplementary file 10 — Source data Fig. 6 [file 44321_2025_323_MOESM10_ESM.zip › Figure 6/6J/mtRosella-G93A-ISO-Merge.tif]

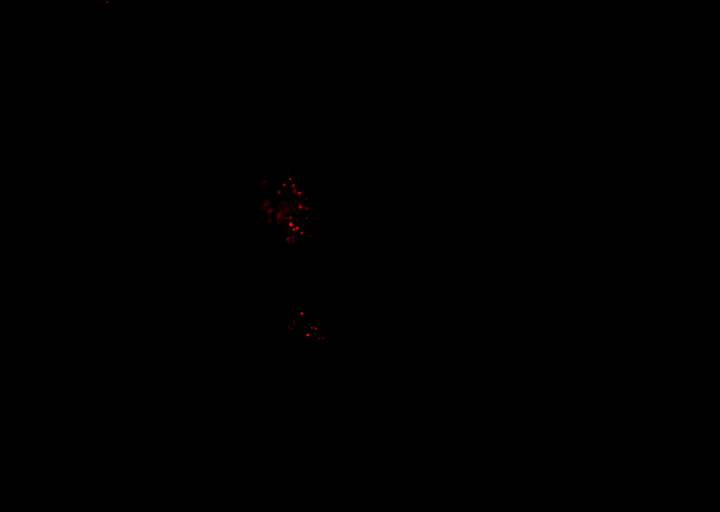

Supplement: Supplementary file 10 — Source data Fig. 6 [file 44321_2025_323_MOESM10_ESM.zip › Figure 6/6J/mtRosella-G93A-ISO-RFP.tif]

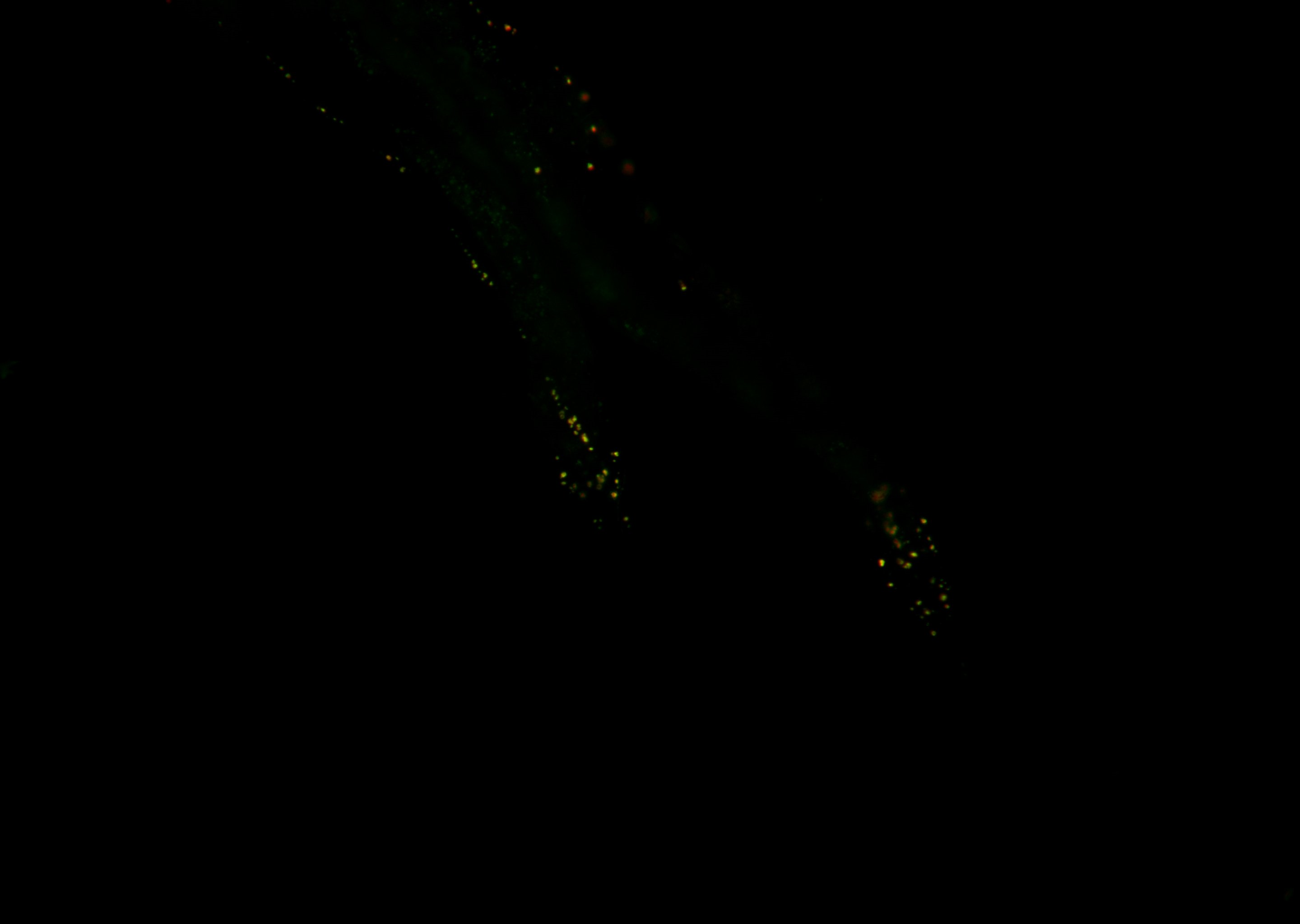

Supplement: Supplementary file 10 — Source data Fig. 6 [file 44321_2025_323_MOESM10_ESM.zip › Figure 6/6J/mtRosella-G93A-Merge.tif]

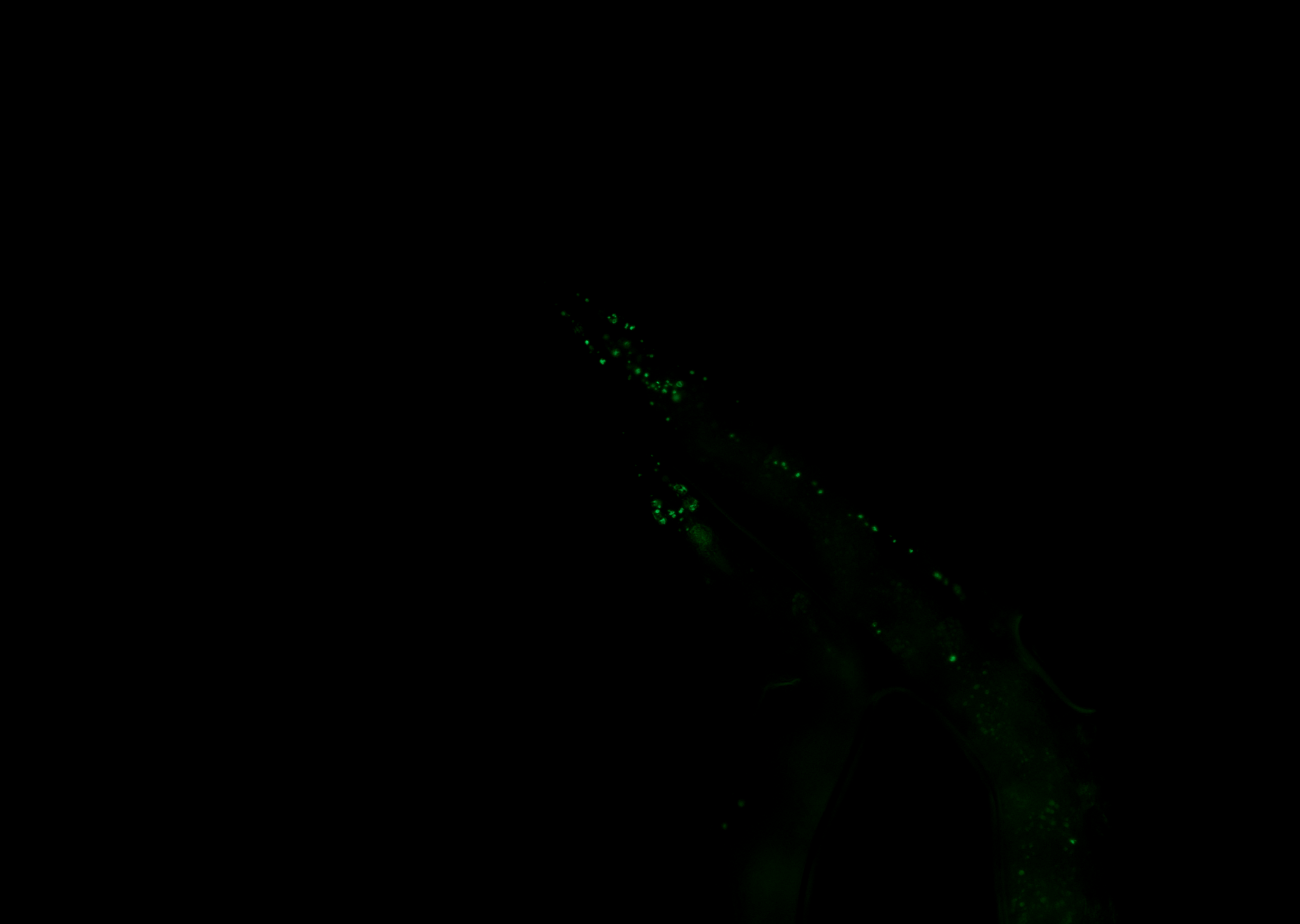

Supplement: Supplementary file 10 — Source data Fig. 6 [file 44321_2025_323_MOESM10_ESM.zip › Figure 6/6J/mtRosella-G93A-PDR1-GFP.tif]

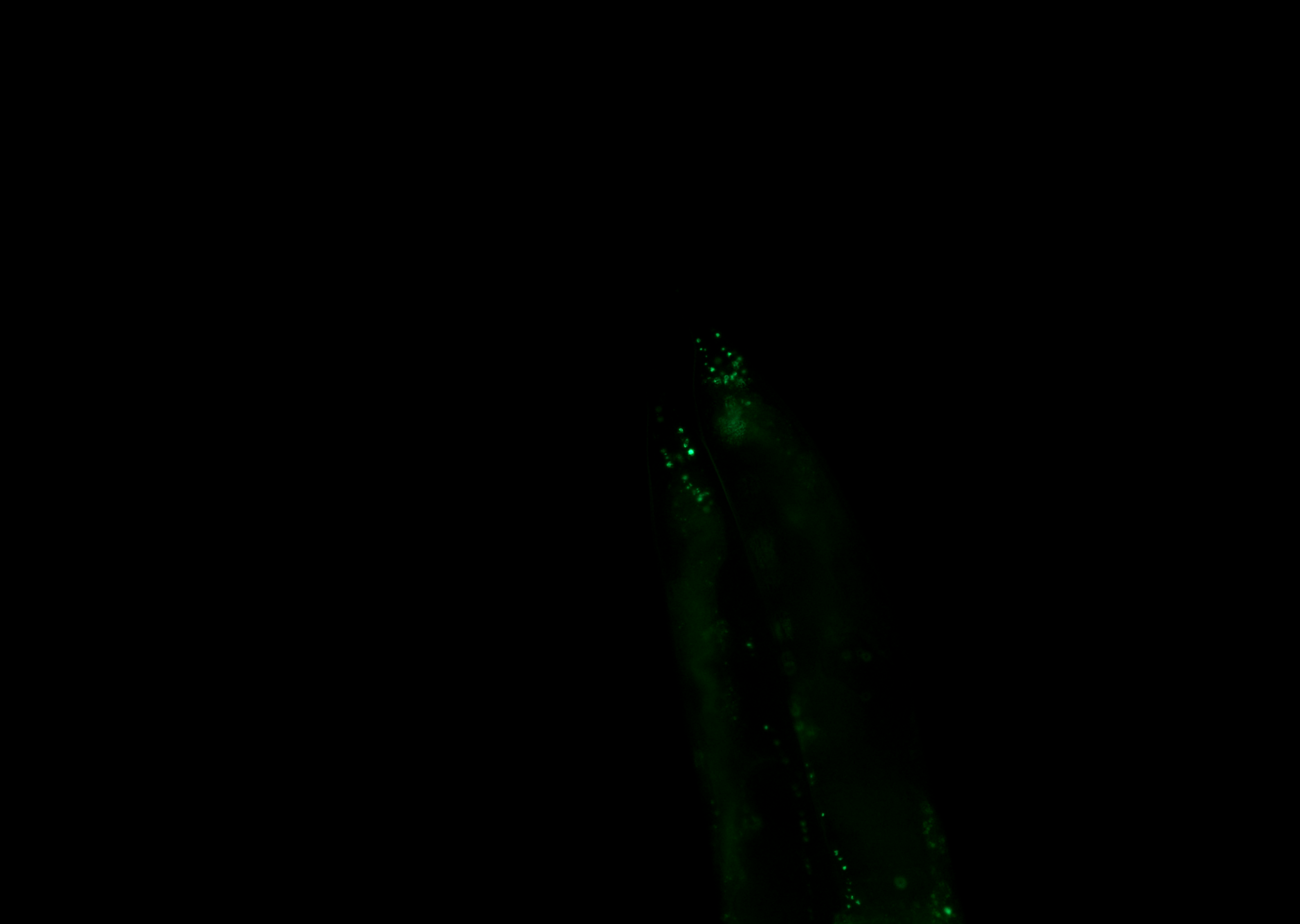

Supplement: Supplementary file 10 — Source data Fig. 6 [file 44321_2025_323_MOESM10_ESM.zip › Figure 6/6J/mtRosella-G93A-PDR1-ISO-GFP.tif]

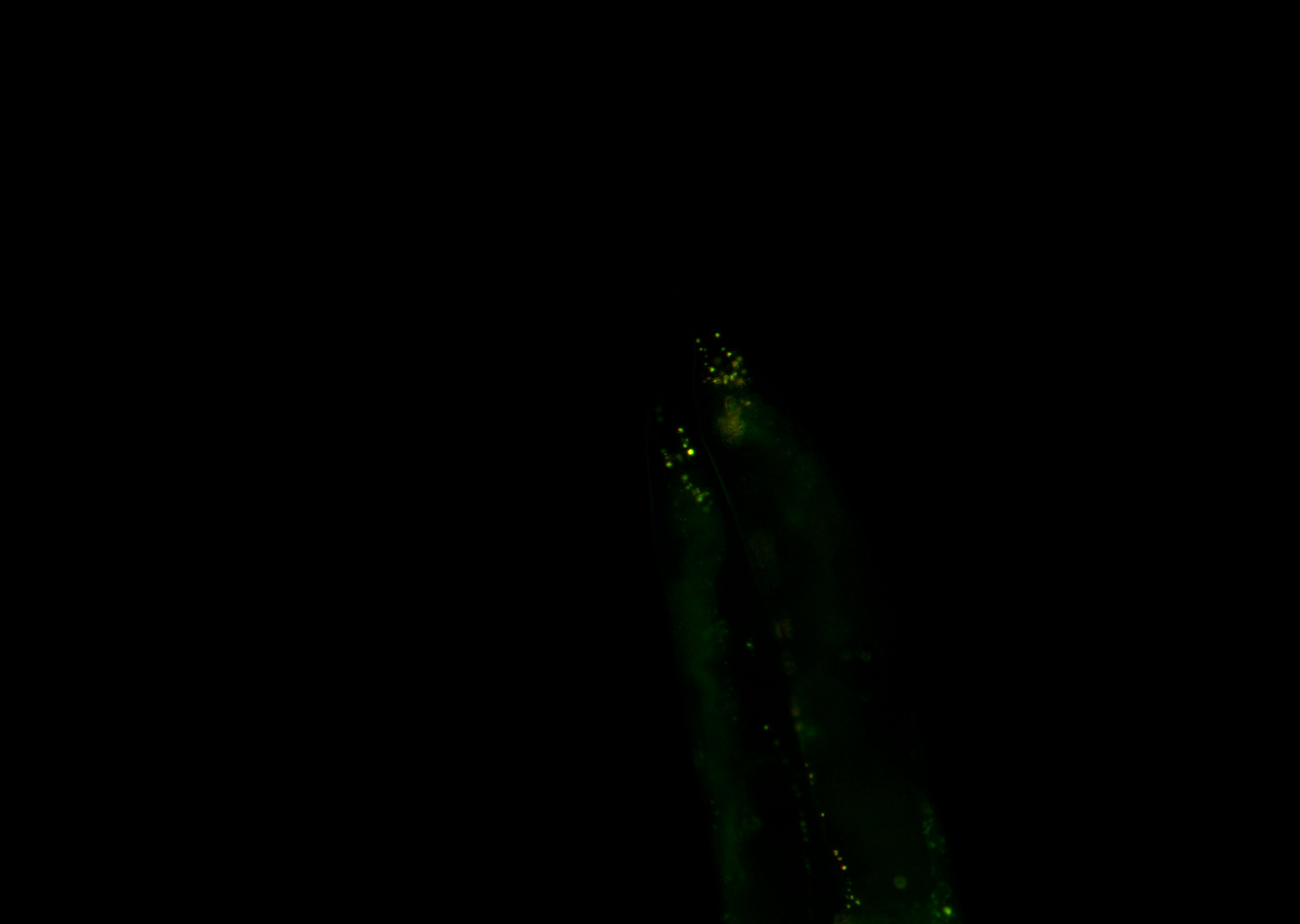

Supplement: Supplementary file 10 — Source data Fig. 6 [file 44321_2025_323_MOESM10_ESM.zip › Figure 6/6J/mtRosella-G93A-PDR1-ISO-Merge.tif]

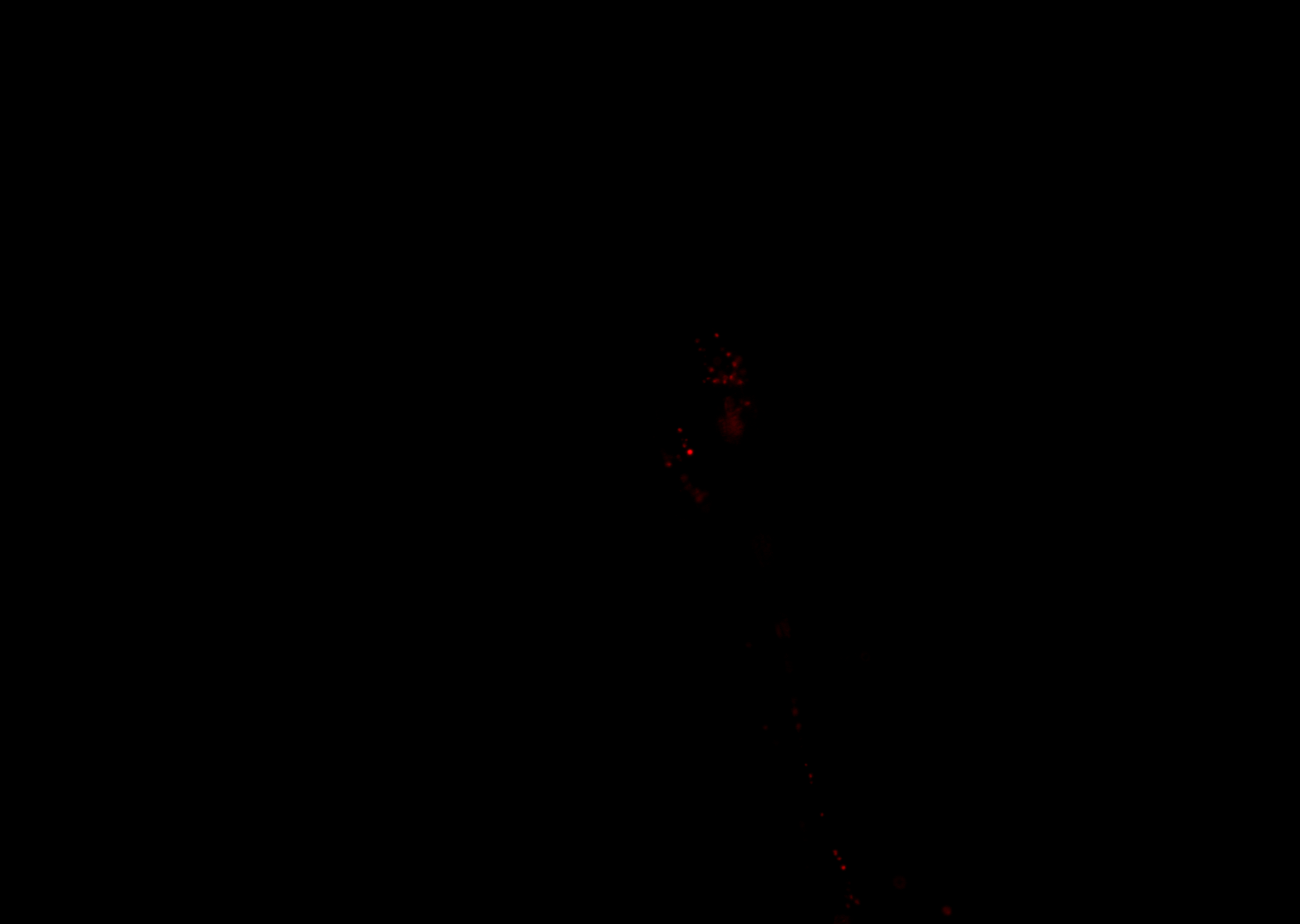

Supplement: Supplementary file 10 — Source data Fig. 6 [file 44321_2025_323_MOESM10_ESM.zip › Figure 6/6J/mtRosella-G93A-PDR1-ISO-RFP.tif]

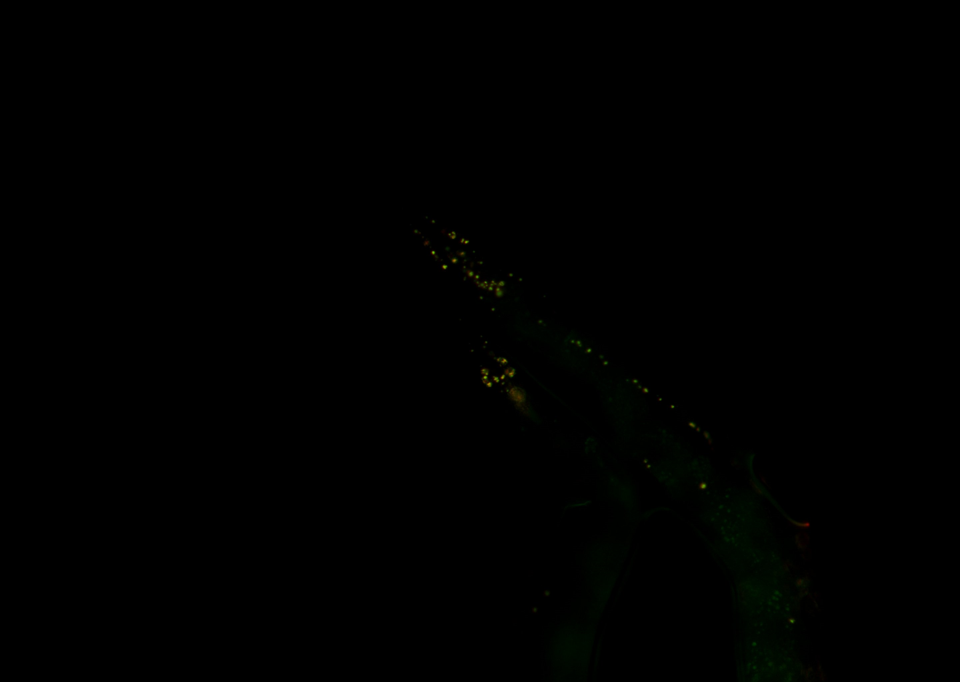

Supplement: Supplementary file 10 — Source data Fig. 6 [file 44321_2025_323_MOESM10_ESM.zip › Figure 6/6J/mtRosella-G93A-PDR1-Merge.tif]

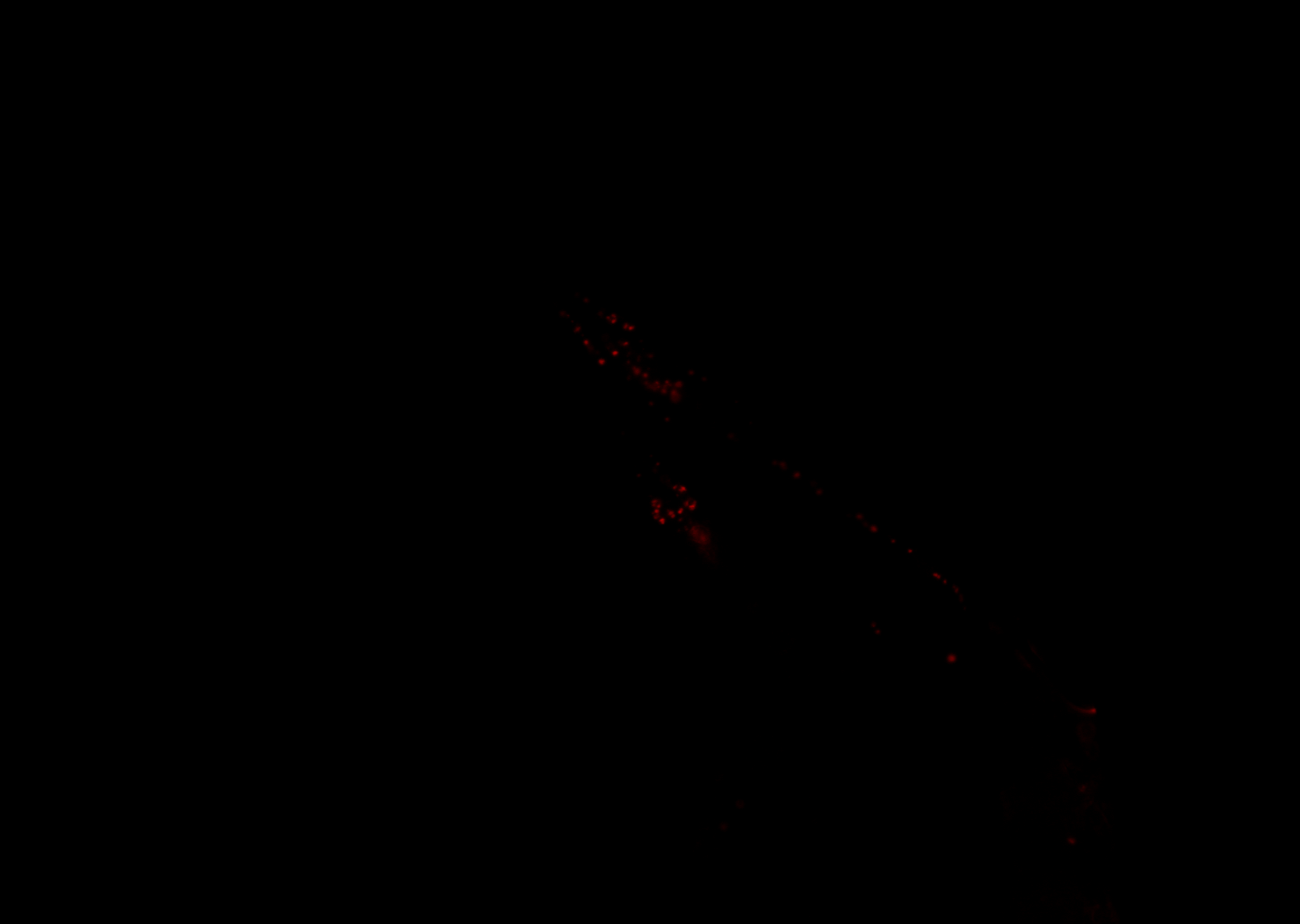

Supplement: Supplementary file 10 — Source data Fig. 6 [file 44321_2025_323_MOESM10_ESM.zip › Figure 6/6J/mtRosella-G93A-PDR1-RFP.tif]

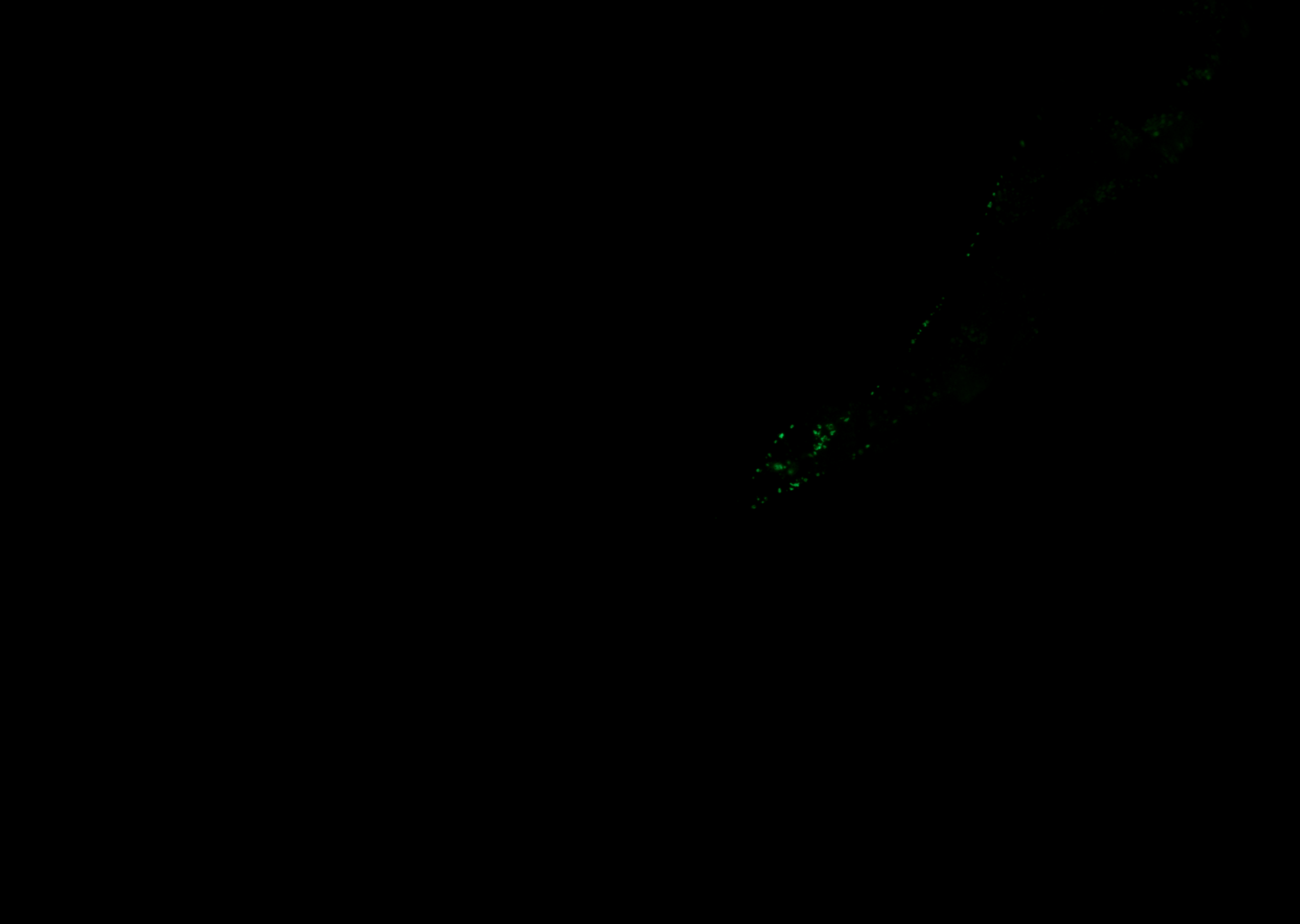

Supplement: Supplementary file 10 — Source data Fig. 6 [file 44321_2025_323_MOESM10_ESM.zip › Figure 6/6J/mtRosella-G93A-PINK1-GFP.tif]

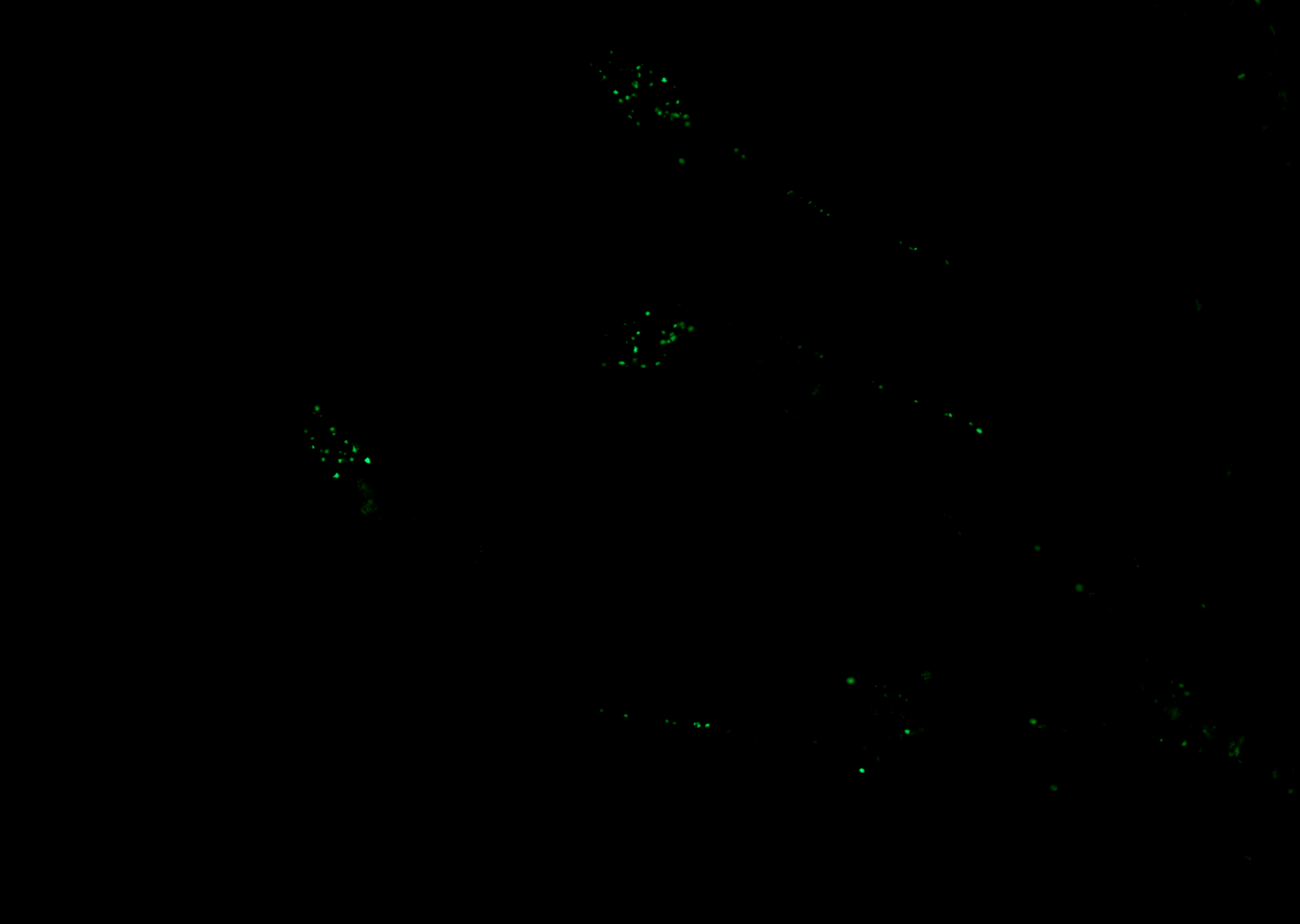

Supplement: Supplementary file 10 — Source data Fig. 6 [file 44321_2025_323_MOESM10_ESM.zip › Figure 6/6J/mtRosella-G93A-PINK1-ISO-GFP.tif]

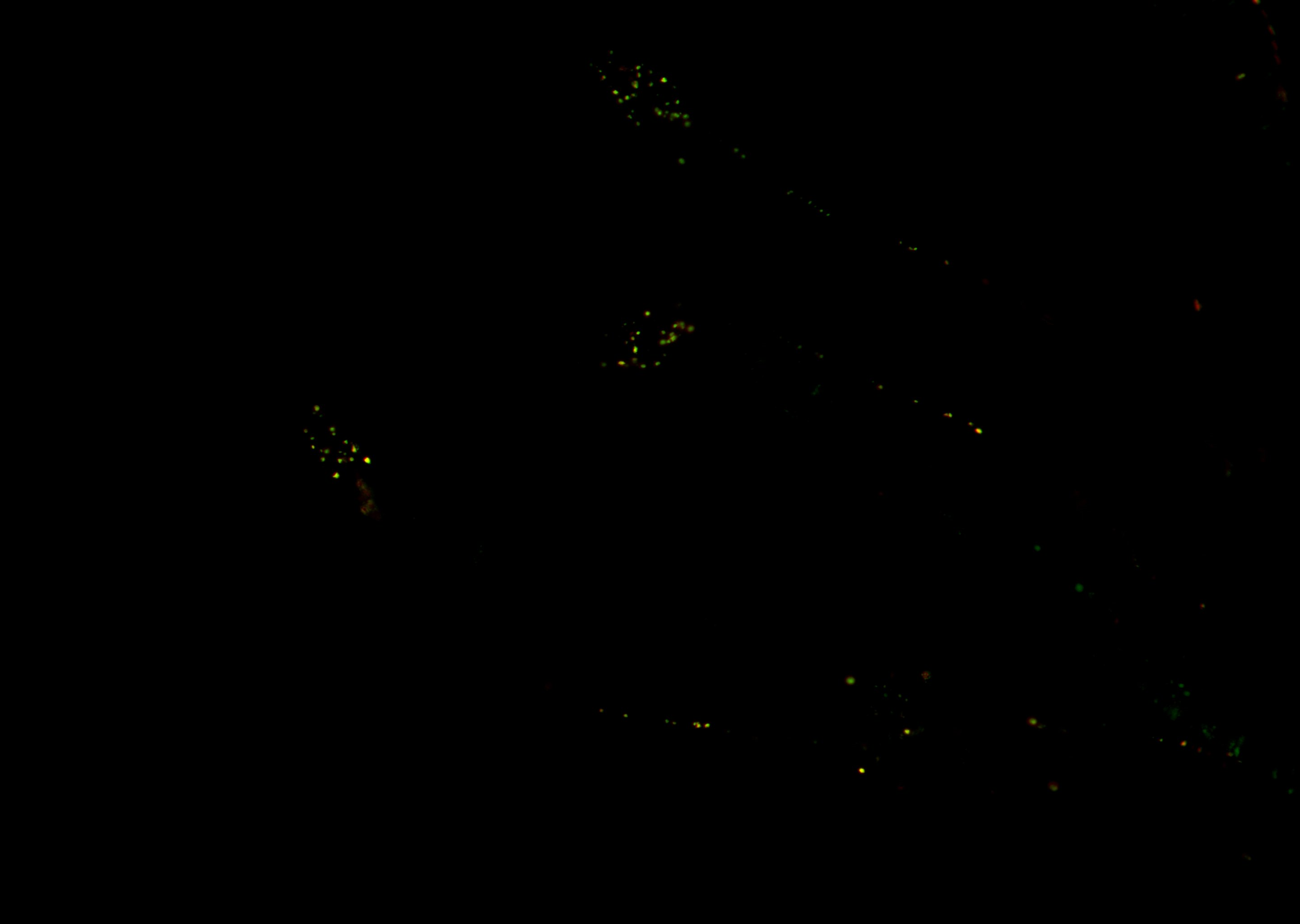

Supplement: Supplementary file 10 — Source data Fig. 6 [file 44321_2025_323_MOESM10_ESM.zip › Figure 6/6J/mtRosella-G93A-PINK1-ISO-Merge.tif]

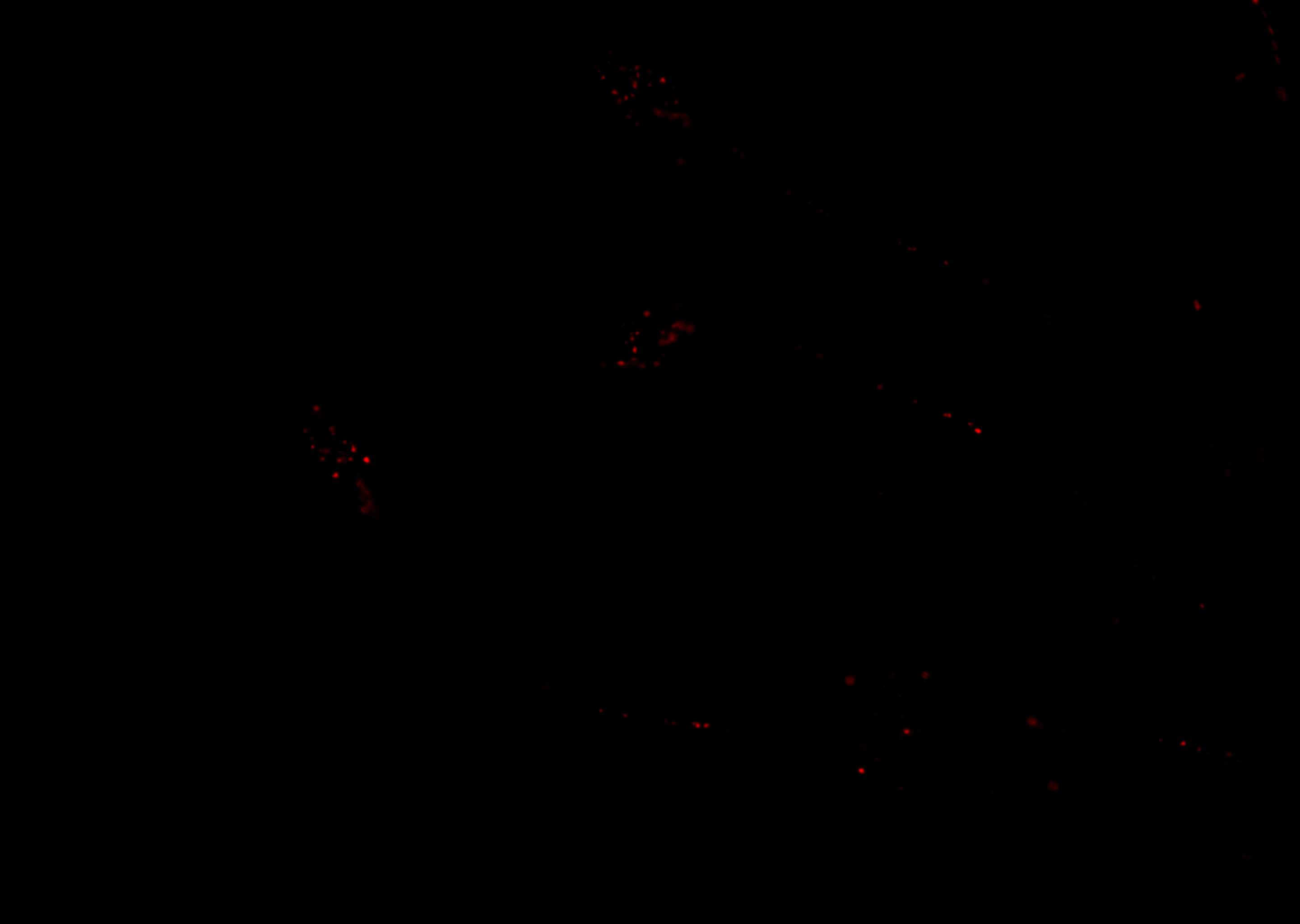

Supplement: Supplementary file 10 — Source data Fig. 6 [file 44321_2025_323_MOESM10_ESM.zip › Figure 6/6J/mtRosella-G93A-PINK1-ISO-RFP.tif]

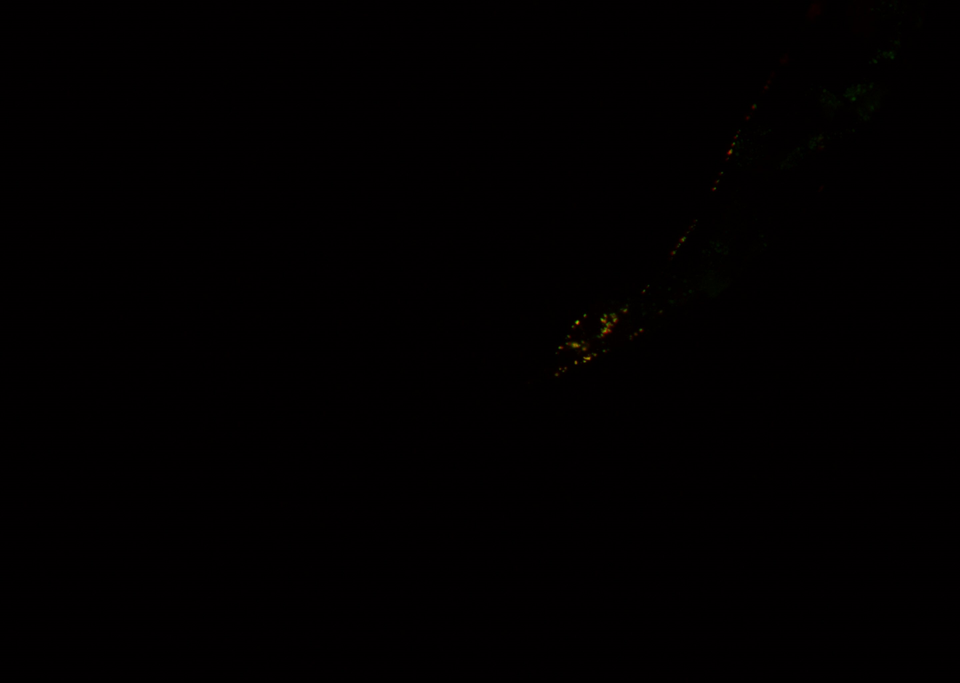

Supplement: Supplementary file 10 — Source data Fig. 6 [file 44321_2025_323_MOESM10_ESM.zip › Figure 6/6J/mtRosella-G93A-PINK1-Merge.tif]

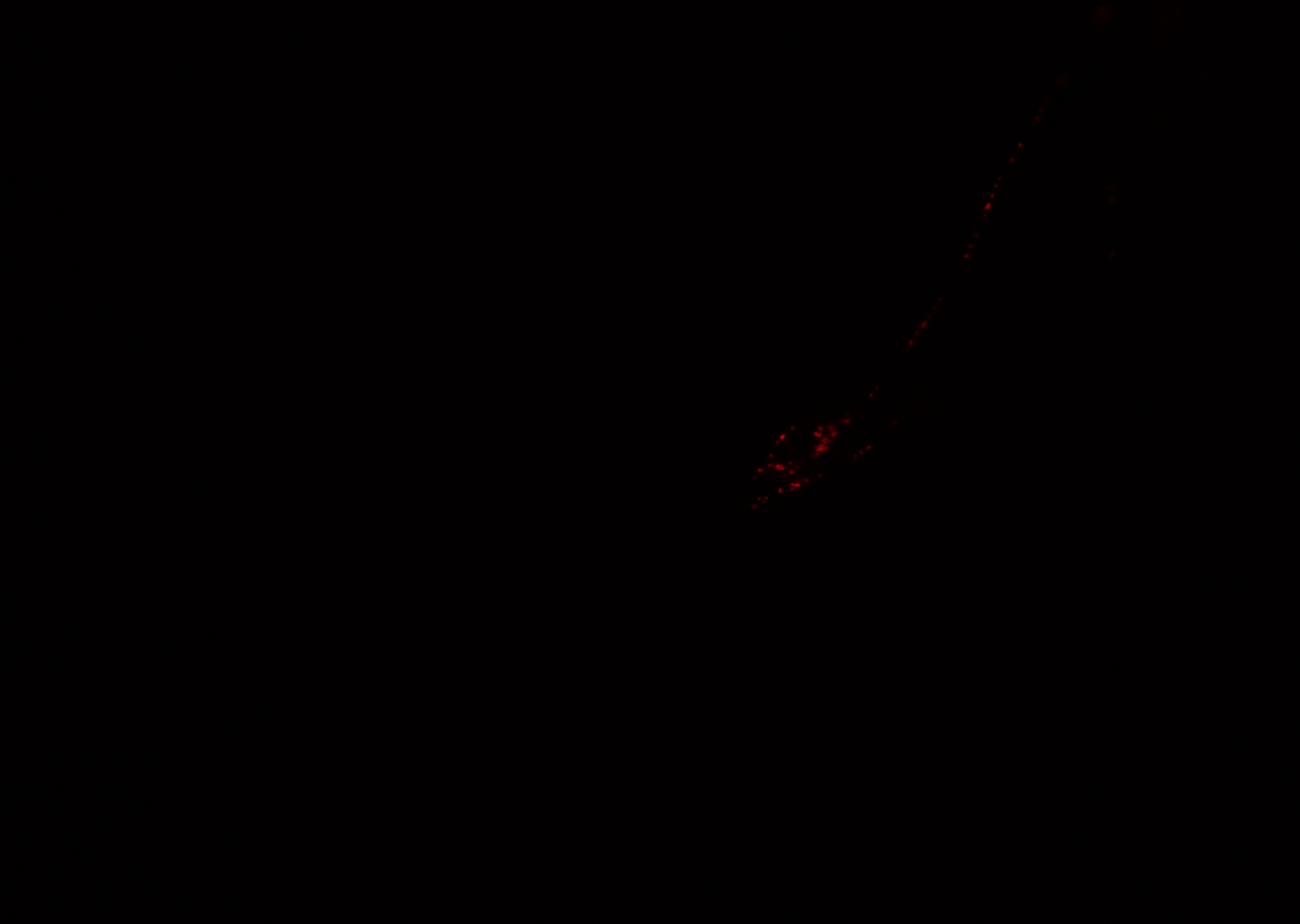

Supplement: Supplementary file 10 — Source data Fig. 6 [file 44321_2025_323_MOESM10_ESM.zip › Figure 6/6J/mtRosella-G93A-PINK1-RFP.tif]

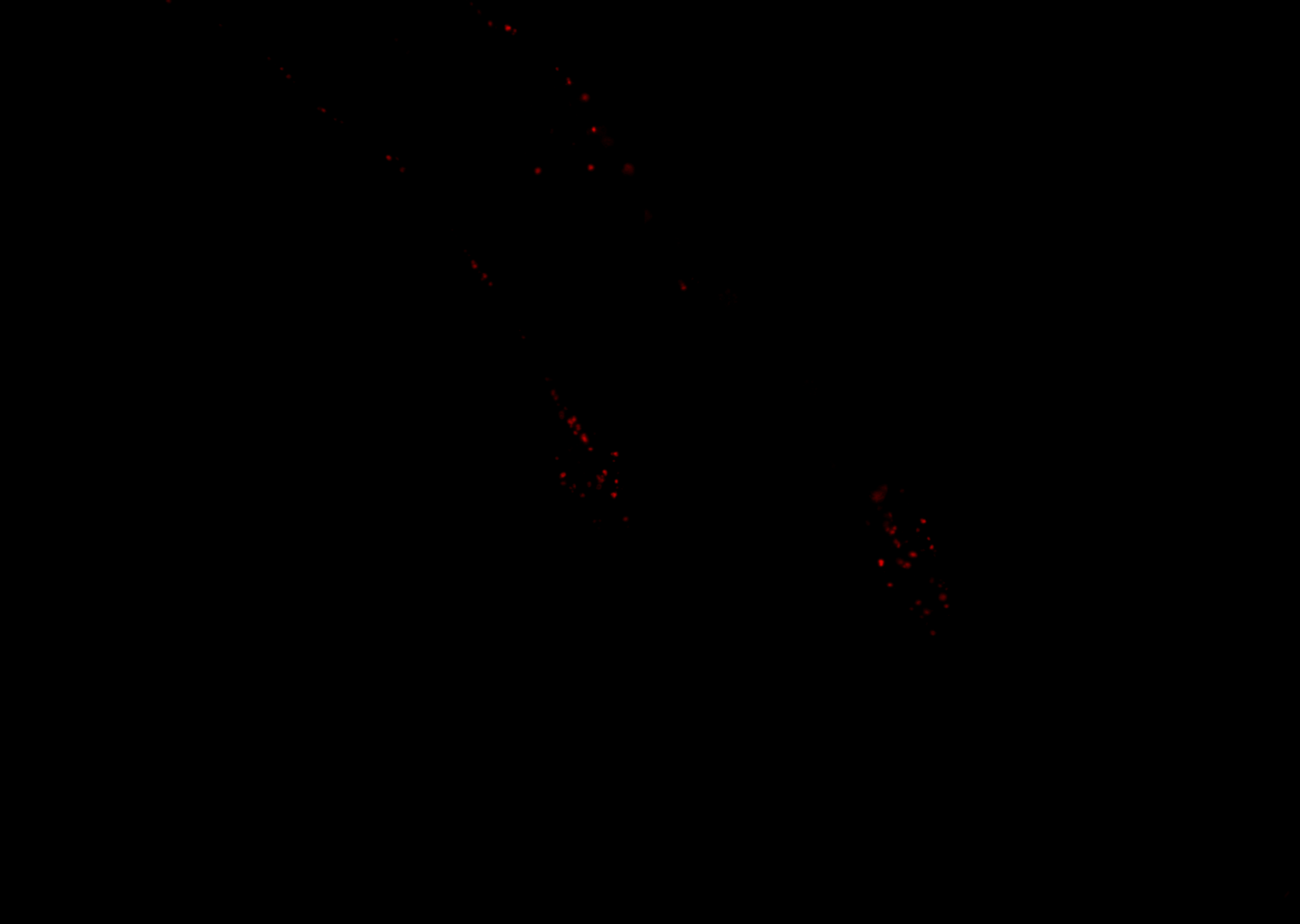

Supplement: Supplementary file 10 — Source data Fig. 6 [file 44321_2025_323_MOESM10_ESM.zip › Figure 6/6J/mtRosella-G93A-RFP.tif]

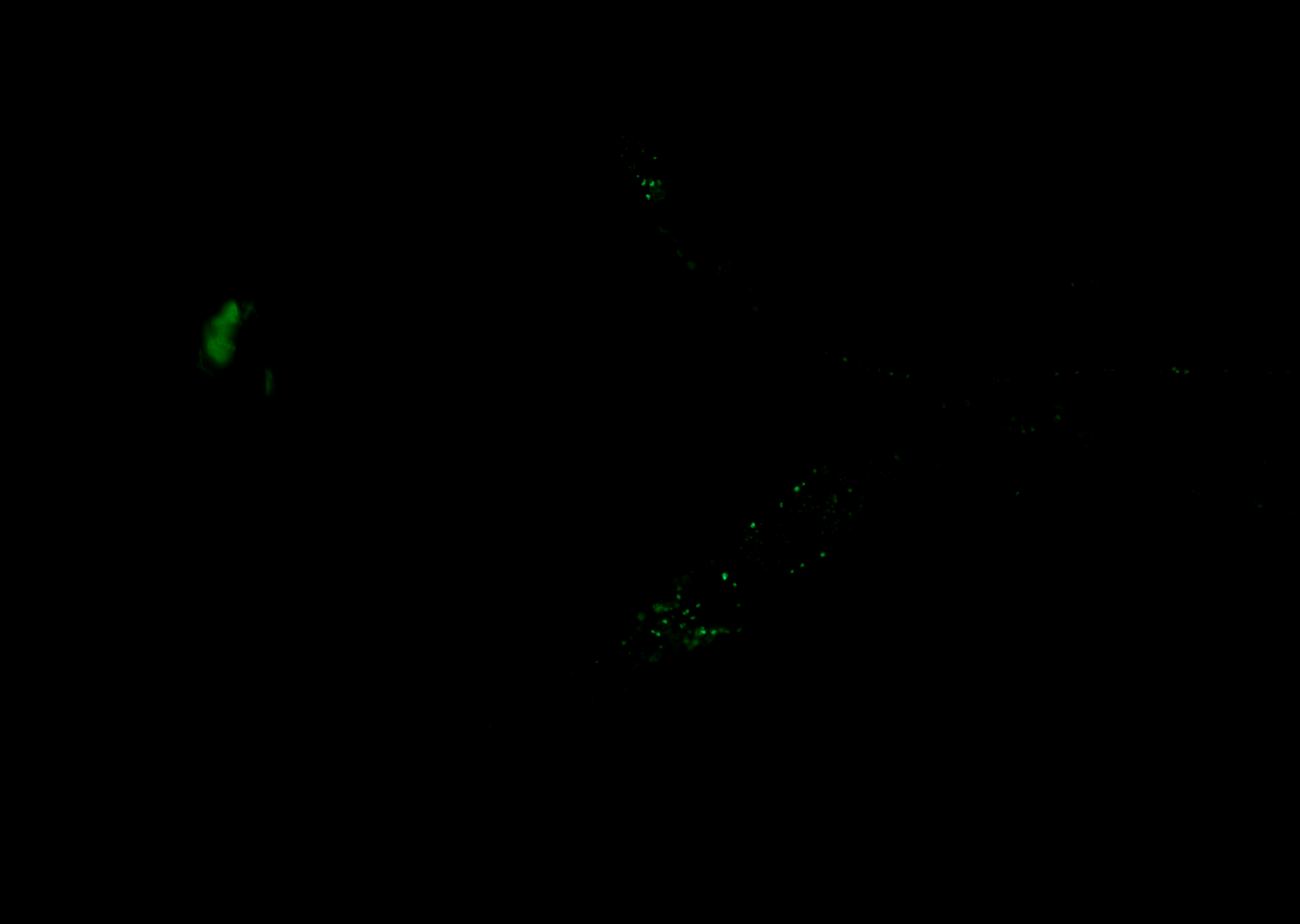

Supplement: Supplementary file 10 — Source data Fig. 6 [file 44321_2025_323_MOESM10_ESM.zip › Figure 6/6J/mtRosella-ISO-GFP.tif]

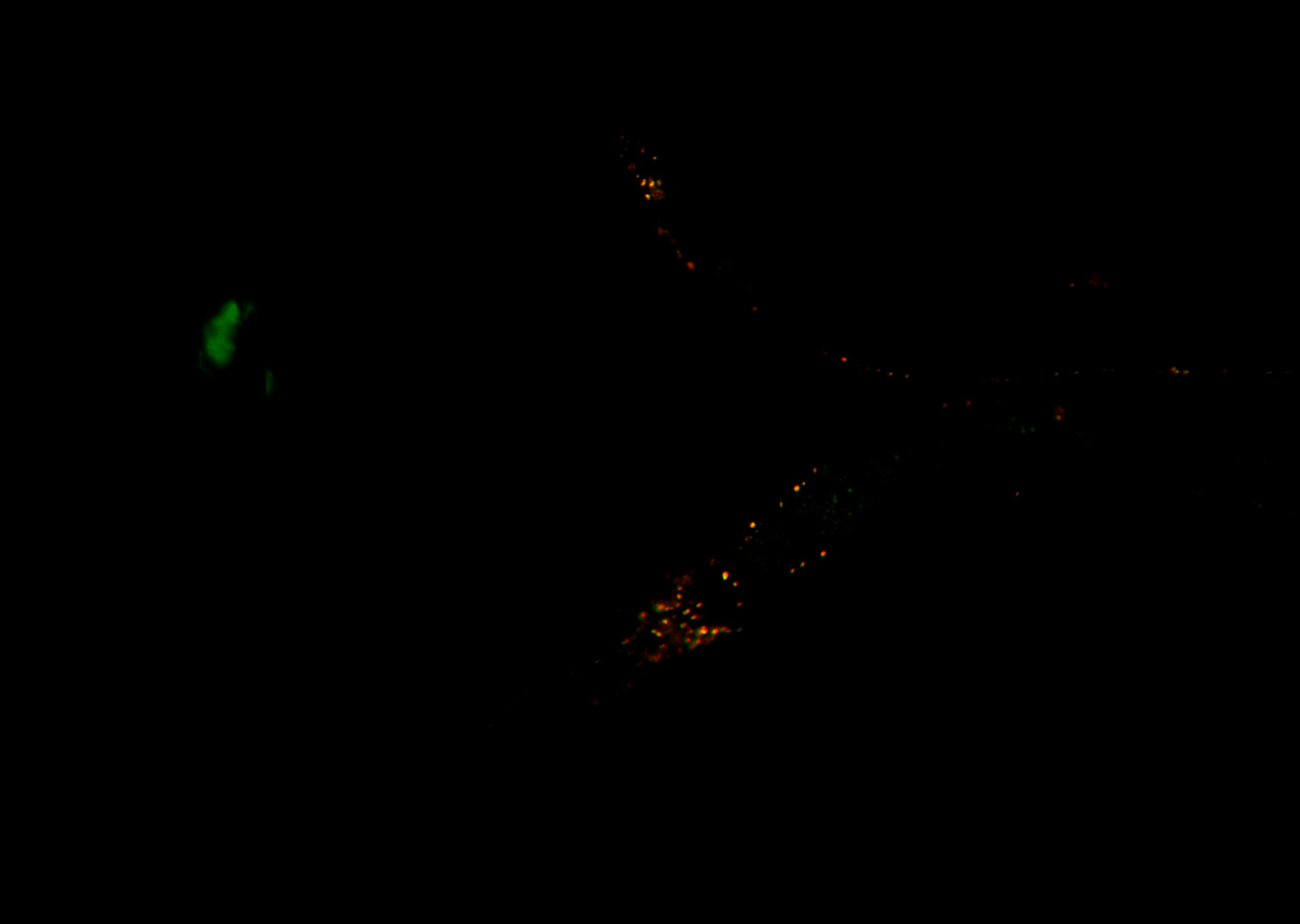

Supplement: Supplementary file 10 — Source data Fig. 6 [file 44321_2025_323_MOESM10_ESM.zip › Figure 6/6J/mtRosella-ISO-Merge.tif]

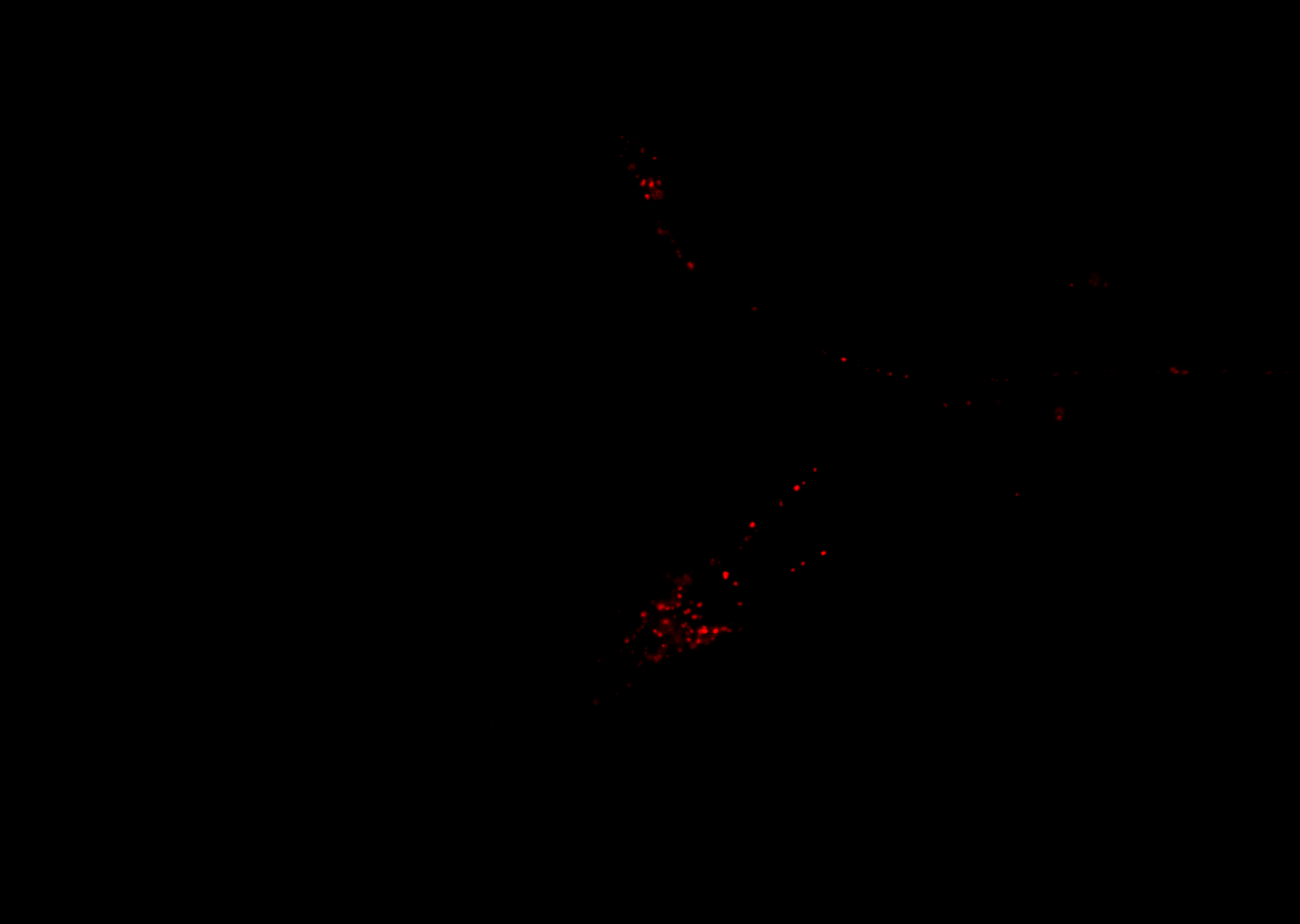

Supplement: Supplementary file 10 — Source data Fig. 6 [file 44321_2025_323_MOESM10_ESM.zip › Figure 6/6J/mtRosella-ISO-RFP.tif]

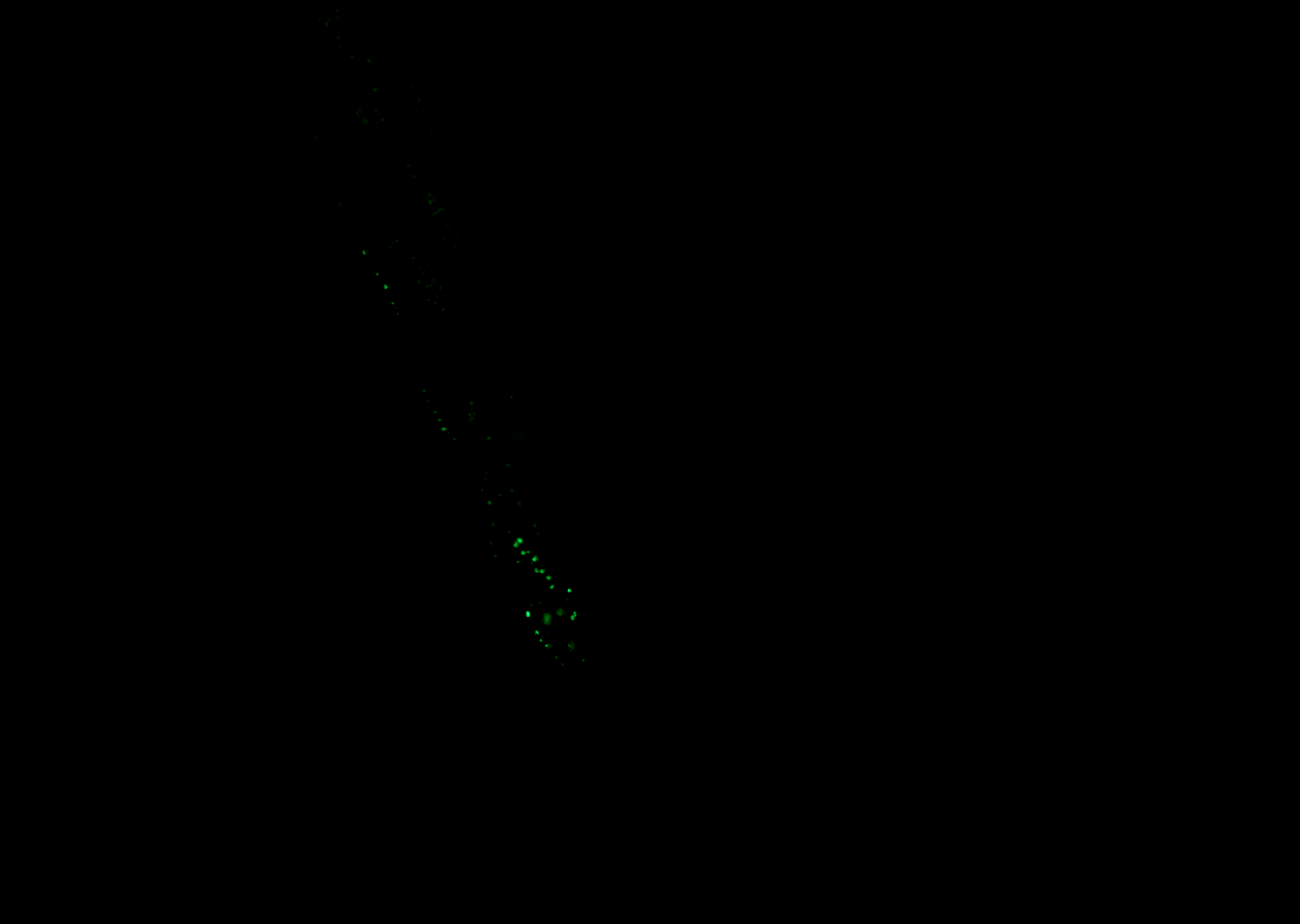

Supplement: Supplementary file 10 — Source data Fig. 6 [file 44321_2025_323_MOESM10_ESM.zip › Figure 6/6J/mtRosella-VEH-GFP.tif]

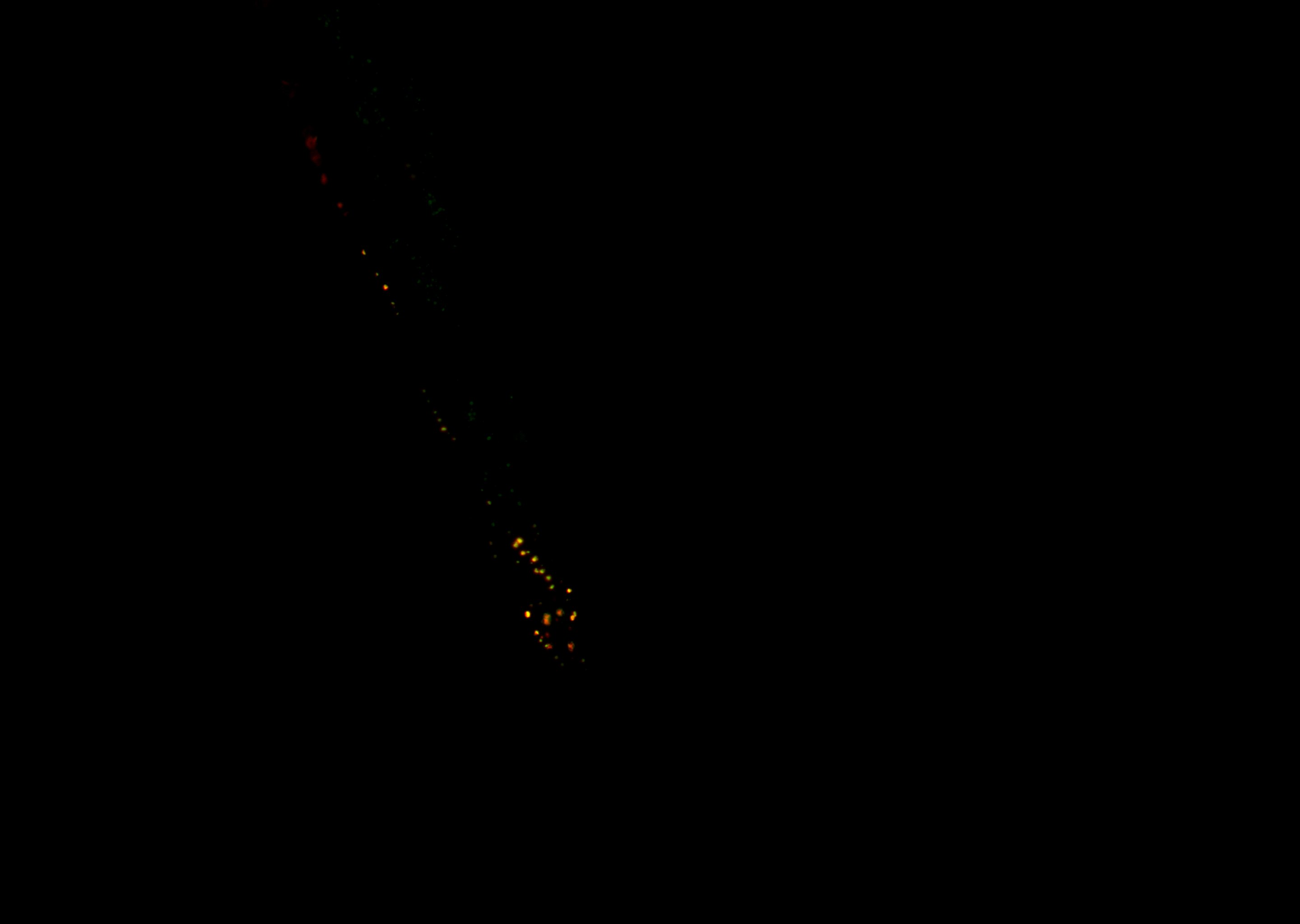

Supplement: Supplementary file 10 — Source data Fig. 6 [file 44321_2025_323_MOESM10_ESM.zip › Figure 6/6J/mtRosella-VEH-Merge.tif]

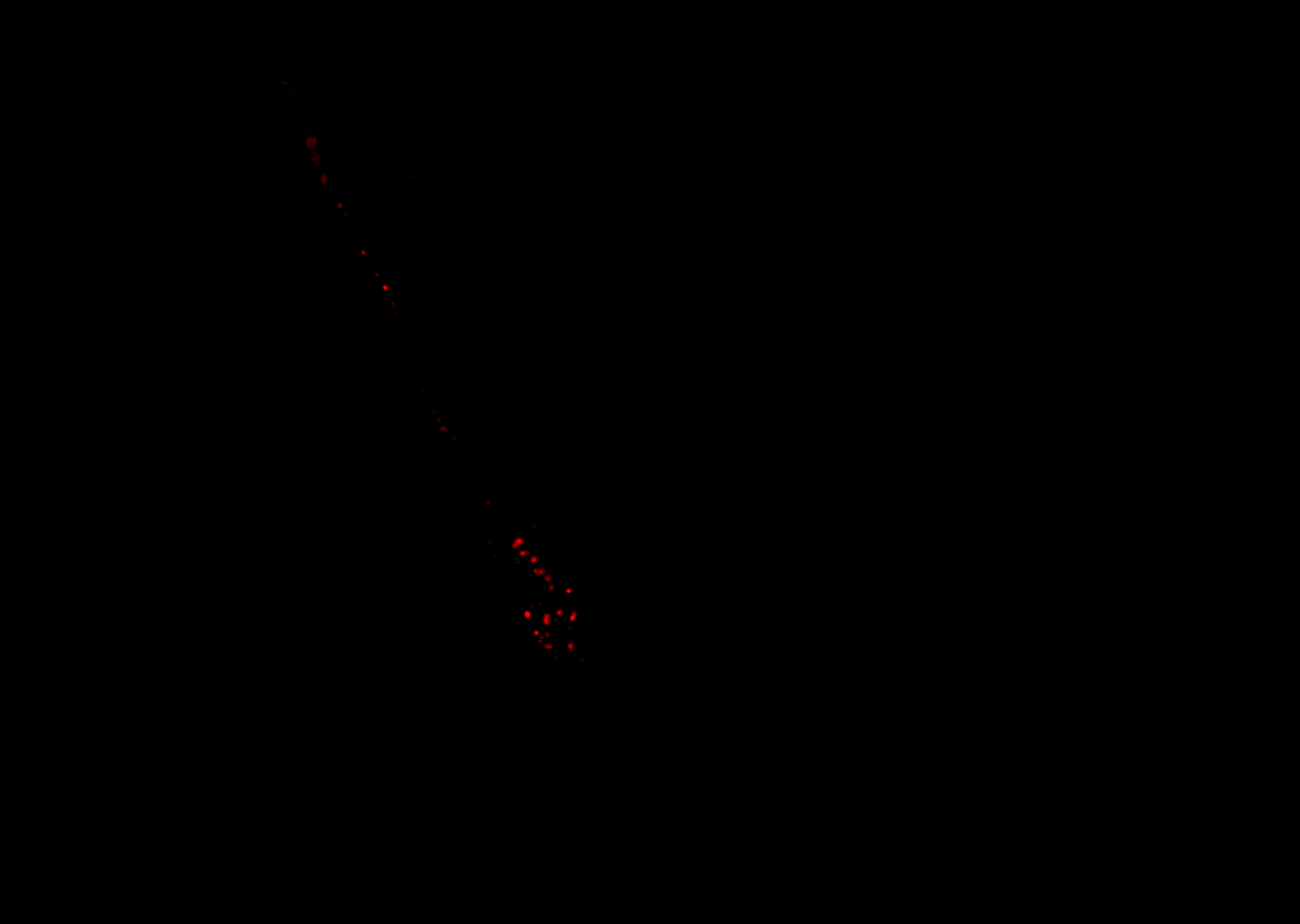

Supplement: Supplementary file 10 — Source data Fig. 6 [file 44321_2025_323_MOESM10_ESM.zip › Figure 6/6J/mtRosella-VEH-RFP.tif]

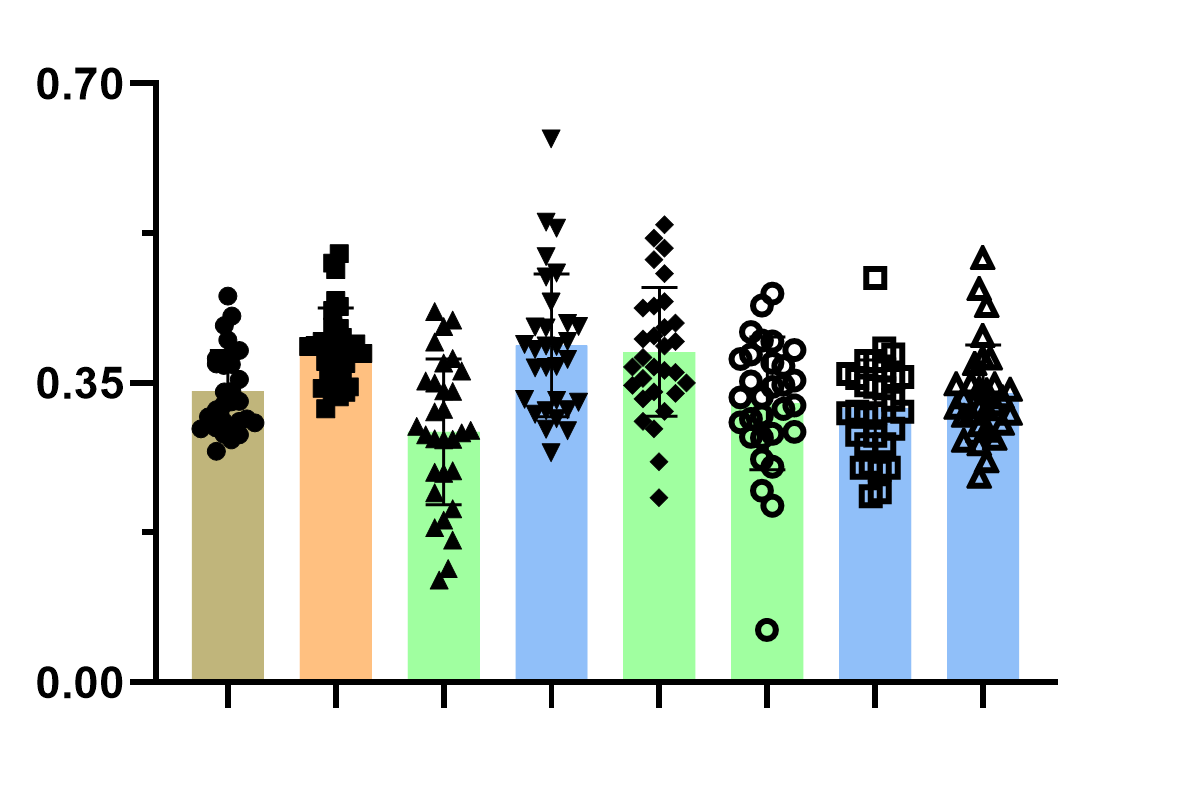

Supplement: Supplementary file 10 — Source data Fig. 6 [file 44321_2025_323_MOESM10_ESM.zip › Figure 6/6K/mtRosella.tif]

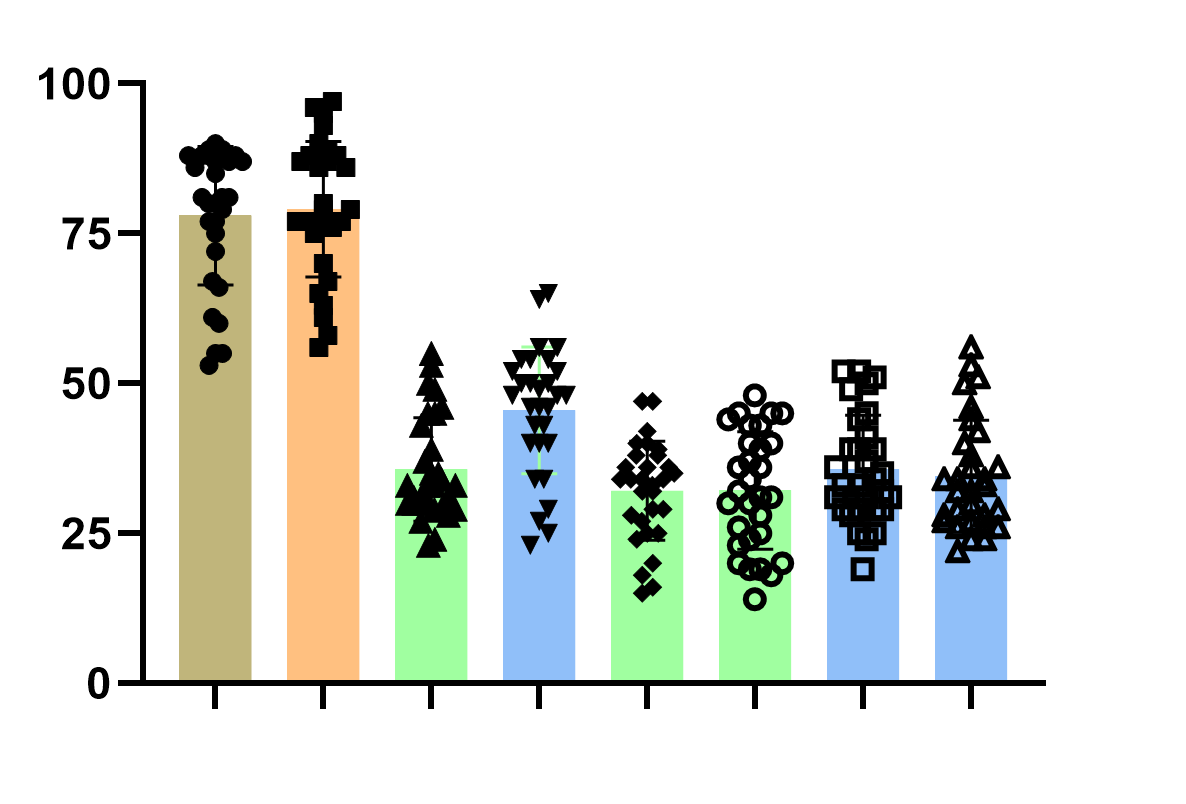

Supplement: Supplementary file 10 — Source data Fig. 6 [file 44321_2025_323_MOESM10_ESM.zip › Figure 6/6L/RNAi swimming.tif]

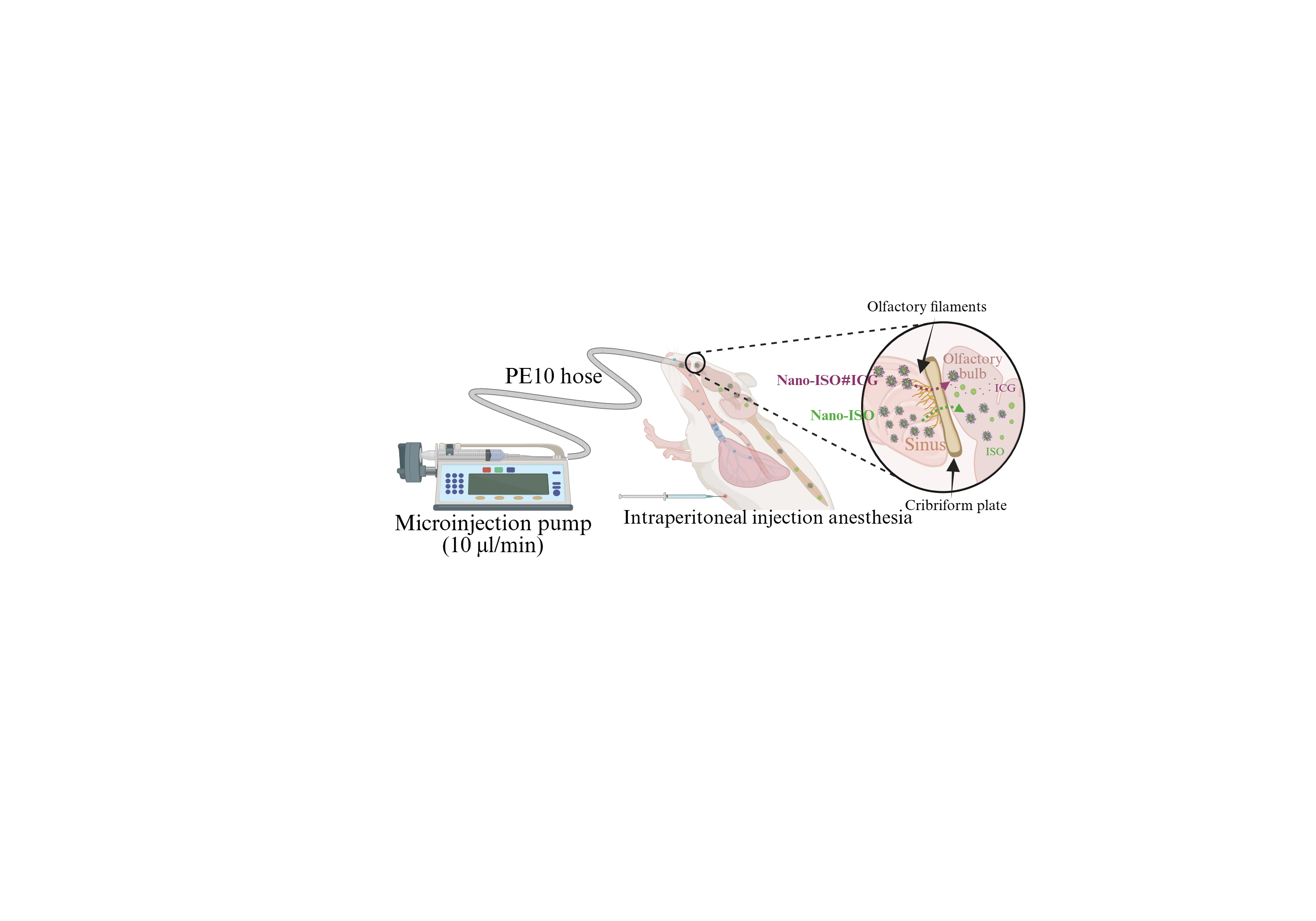

Supplement: Supplementary file 11 — Source data Fig. 7 [file 44321_2025_323_MOESM11_ESM.zip › Figure 7/7A/6A.png]

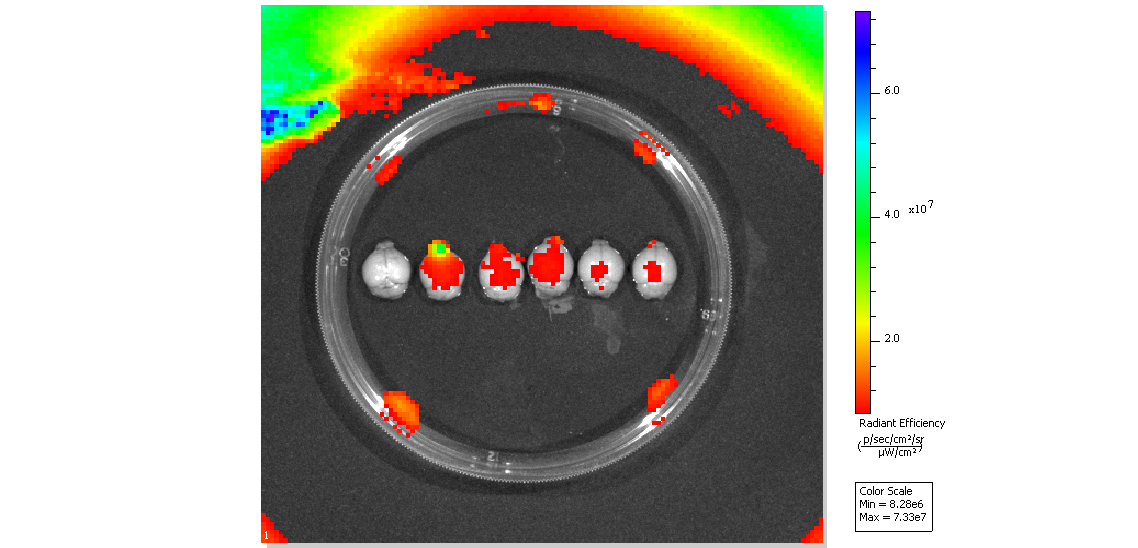

Supplement: Supplementary file 11 — Source data Fig. 7 [file 44321_2025_323_MOESM11_ESM.zip › Figure 7/7B/brain iso igc.tif]

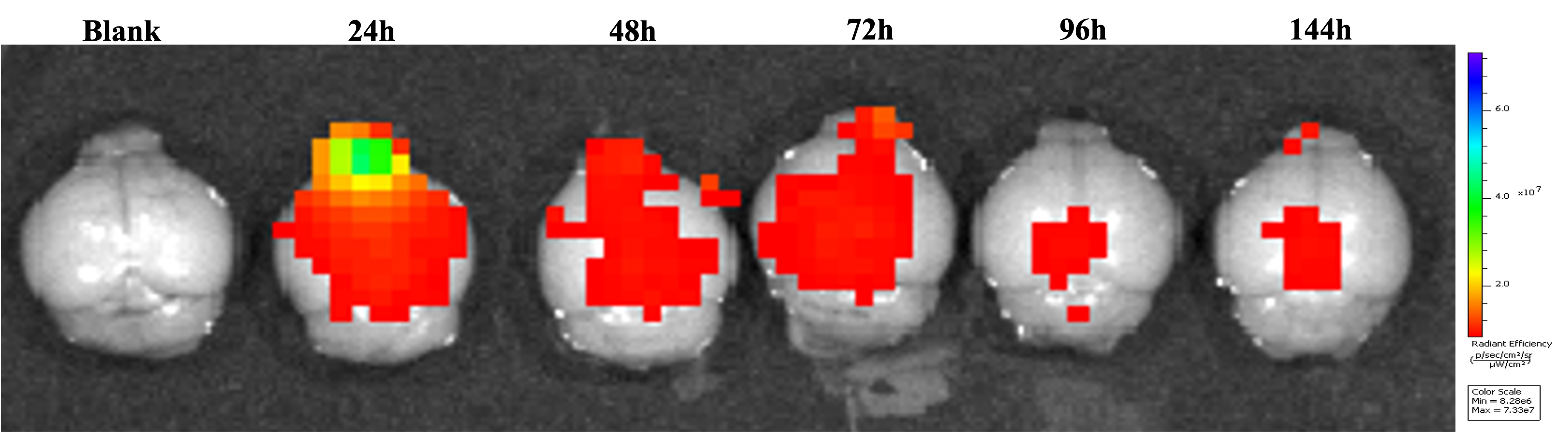

Supplement: Supplementary file 11 — Source data Fig. 7 [file 44321_2025_323_MOESM11_ESM.zip › Figure 7/7B/Selected show.jpg]

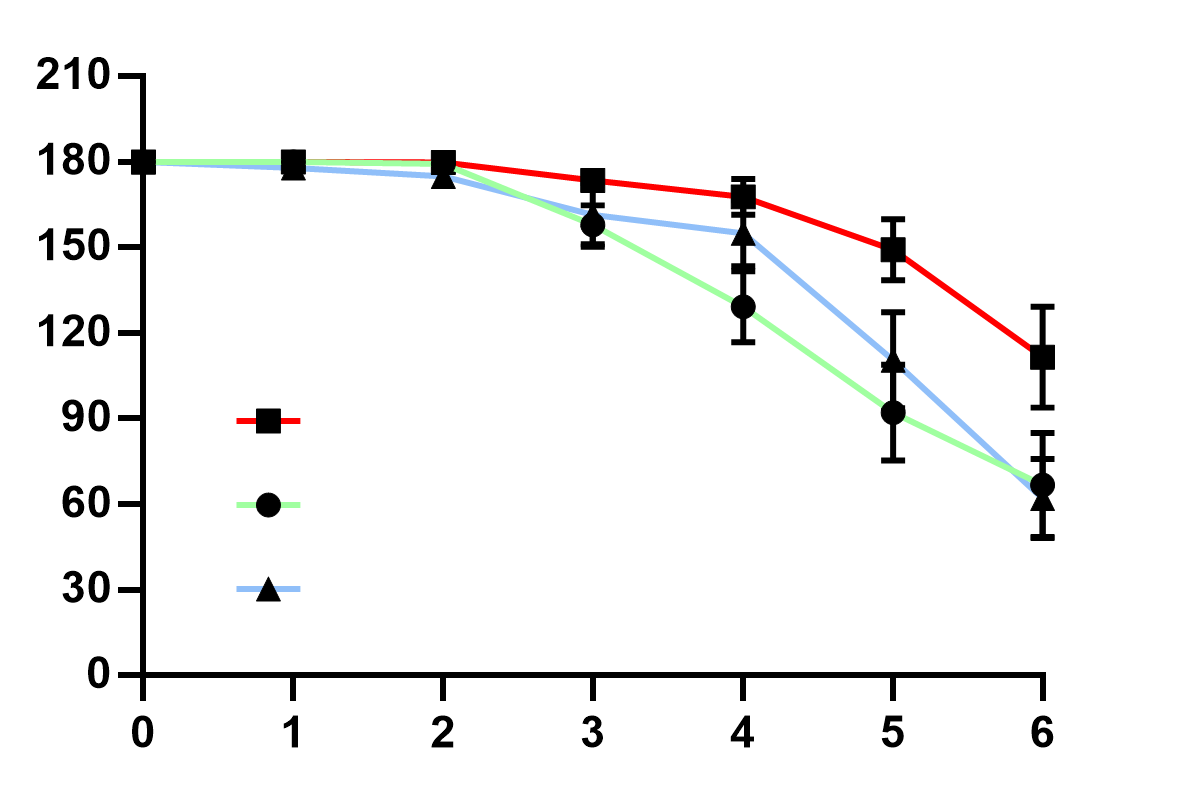

Supplement: Supplementary file 11 — Source data Fig. 7 [file 44321_2025_323_MOESM11_ESM.zip › Figure 7/7D-F/hanging.tif]

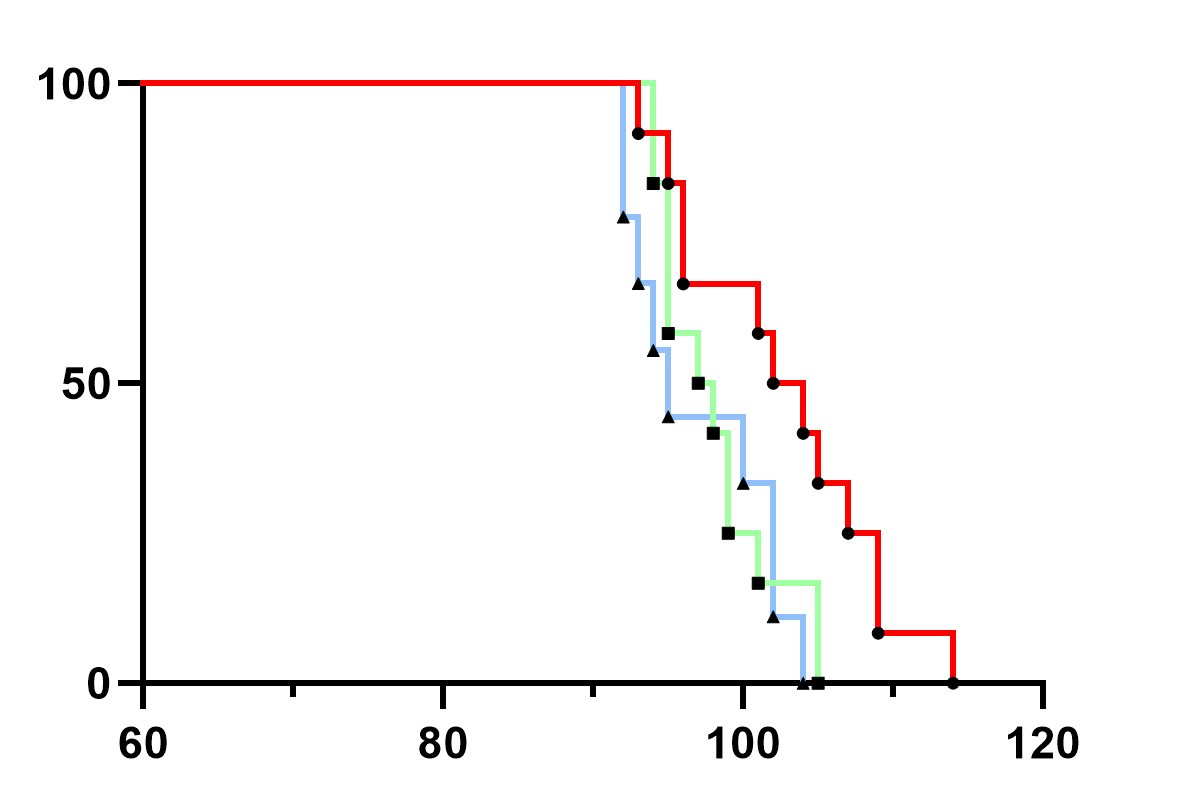

Supplement: Supplementary file 11 — Source data Fig. 7 [file 44321_2025_323_MOESM11_ESM.zip › Figure 7/7D-F/onset.tif]

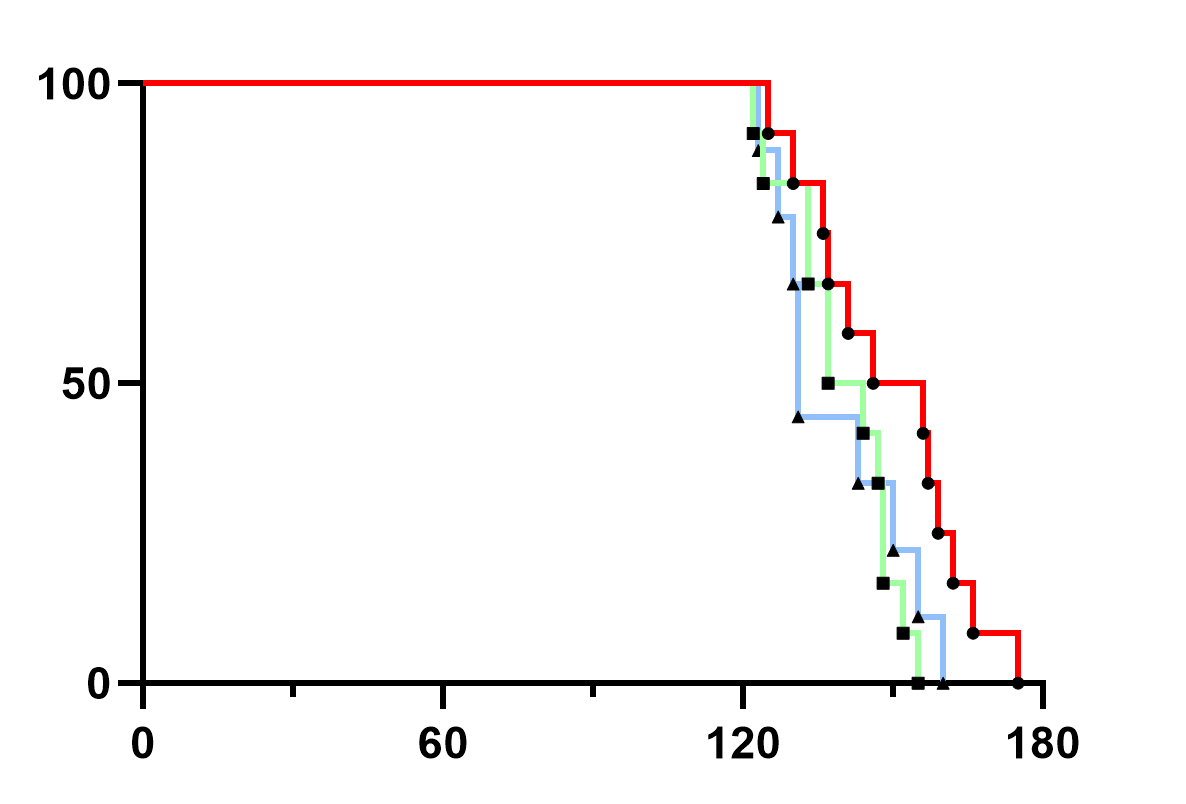

Supplement: Supplementary file 11 — Source data Fig. 7 [file 44321_2025_323_MOESM11_ESM.zip › Figure 7/7D-F/survival.tif]

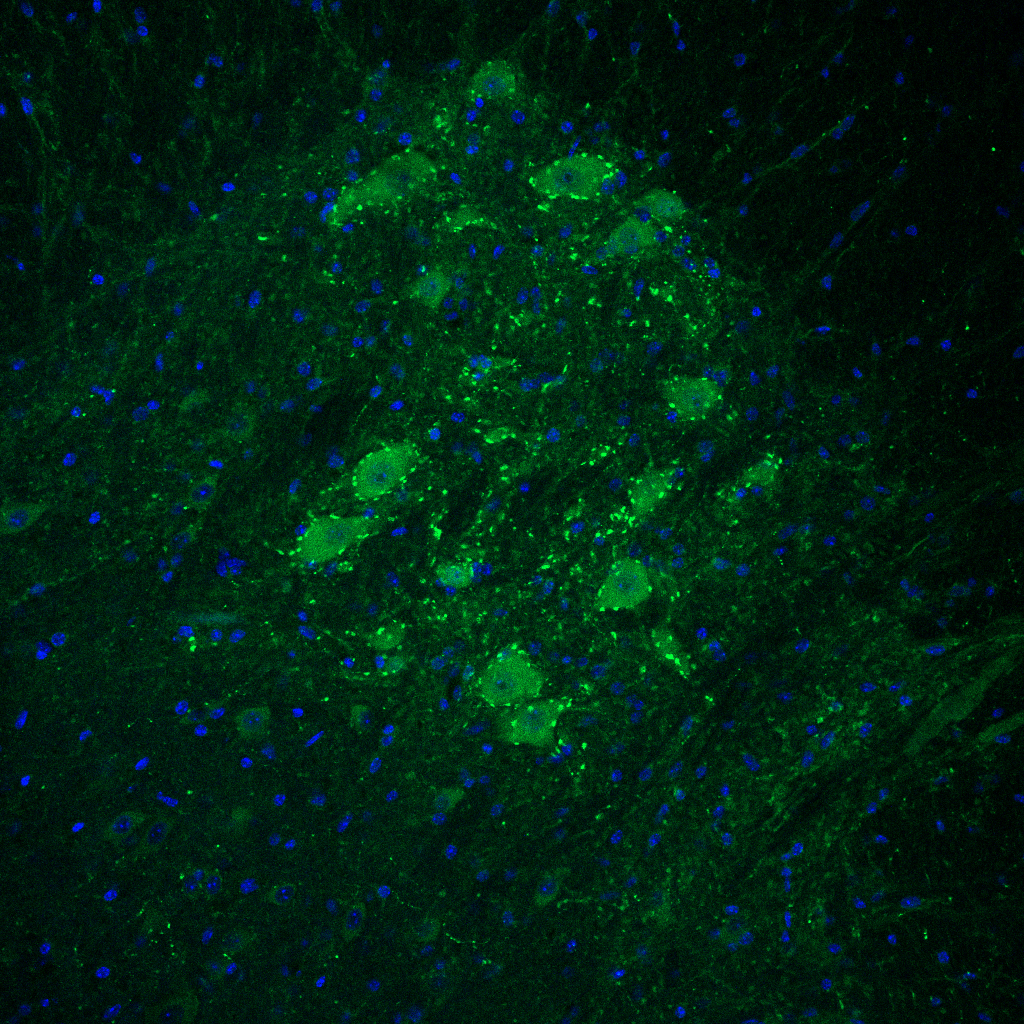

Supplement: Supplementary file 11 — Source data Fig. 7 [file 44321_2025_323_MOESM11_ESM.zip › Figure 7/7G/ISO CHAT 20X.tif]

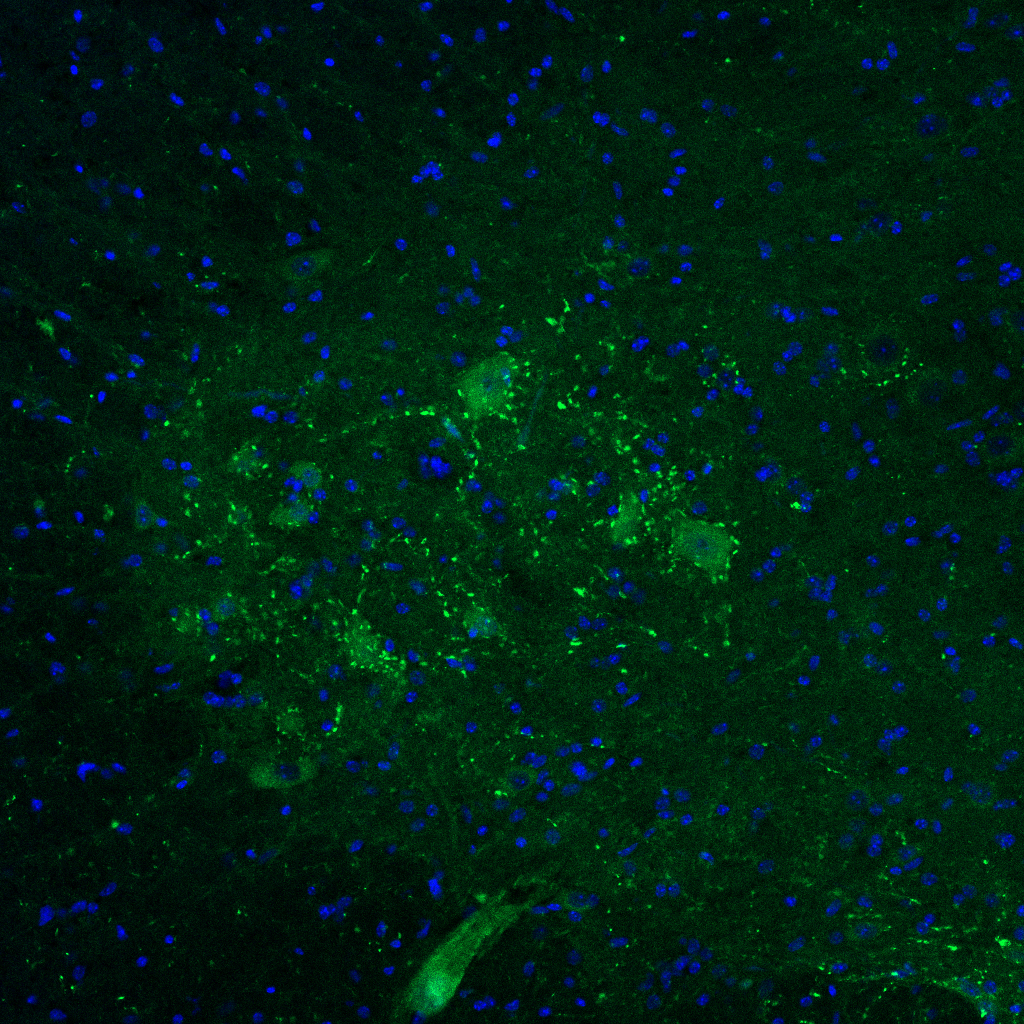

Supplement: Supplementary file 11 — Source data Fig. 7 [file 44321_2025_323_MOESM11_ESM.zip › Figure 7/7G/VEH CHAT 20X.tif]

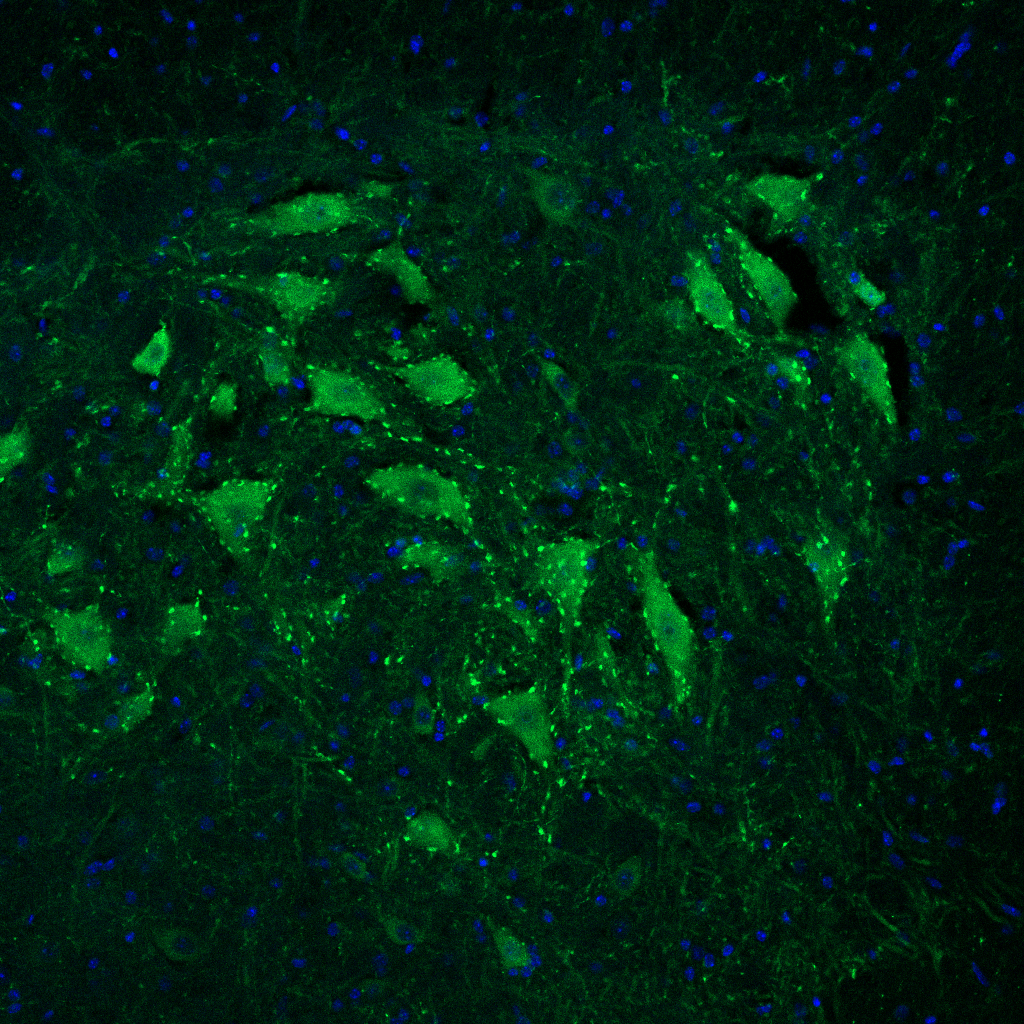

Supplement: Supplementary file 11 — Source data Fig. 7 [file 44321_2025_323_MOESM11_ESM.zip › Figure 7/7G/WT CHAT 20X.tif]

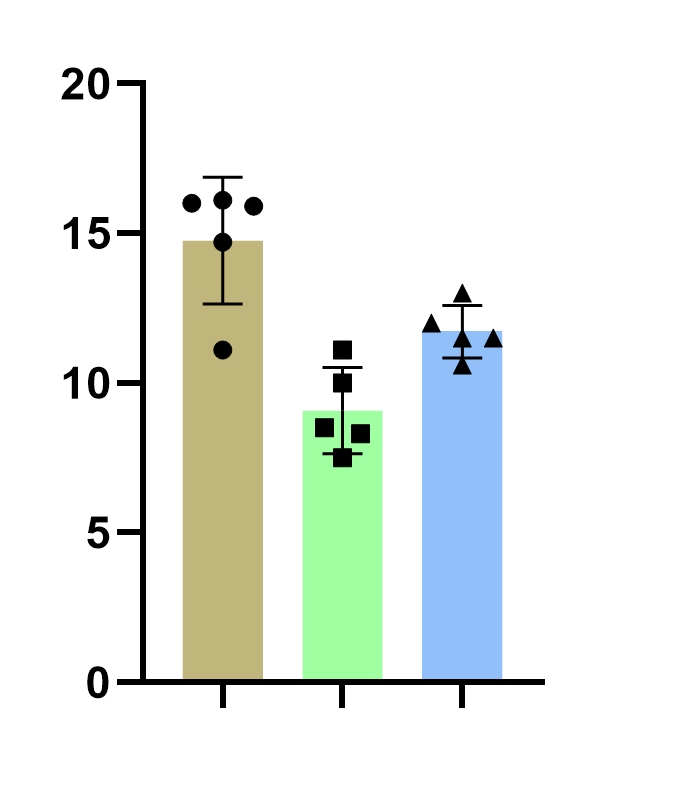

Supplement: Supplementary file 11 — Source data Fig. 7 [file 44321_2025_323_MOESM11_ESM.zip › Figure 7/7H/MN Number.tif]

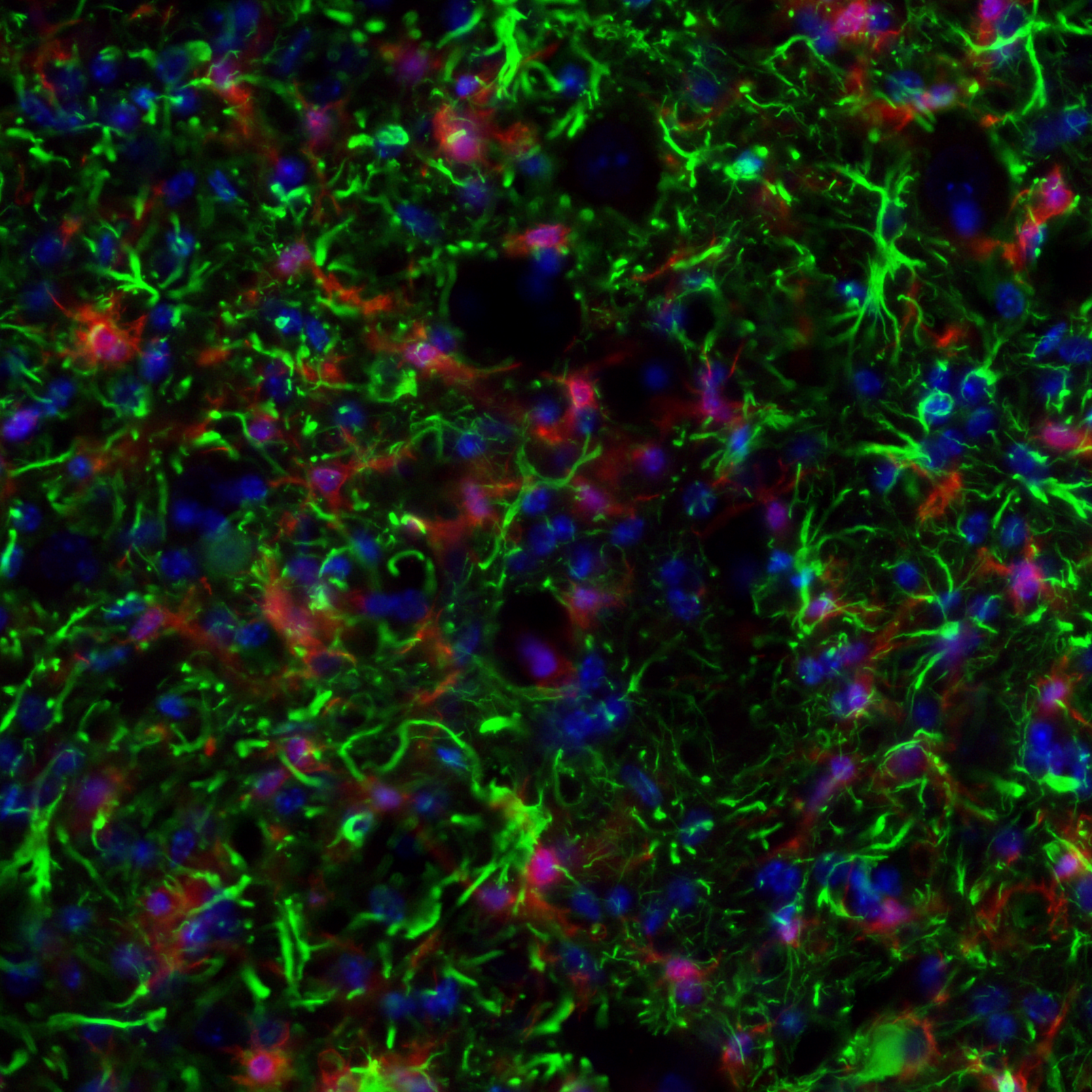

Supplement: Supplementary file 11 — Source data Fig. 7 [file 44321_2025_323_MOESM11_ESM.zip › Figure 7/7I/ISO IBA1488-GFAP555 40X 2_RGB.tif]

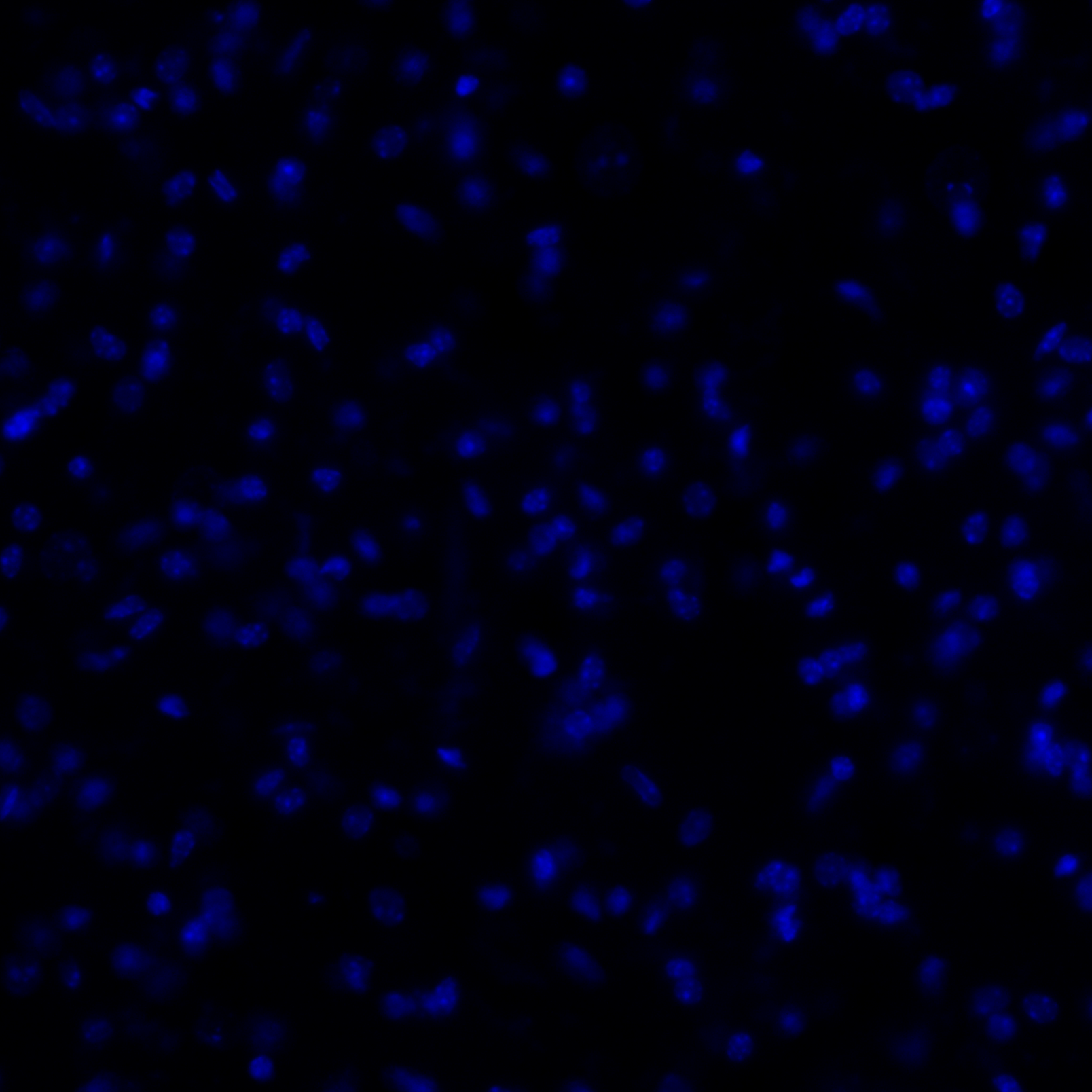

Supplement: Supplementary file 11 — Source data Fig. 7 [file 44321_2025_323_MOESM11_ESM.zip › Figure 7/7I/ISO IBA1488-GFAP555 40X 2_RGB_405.tif]

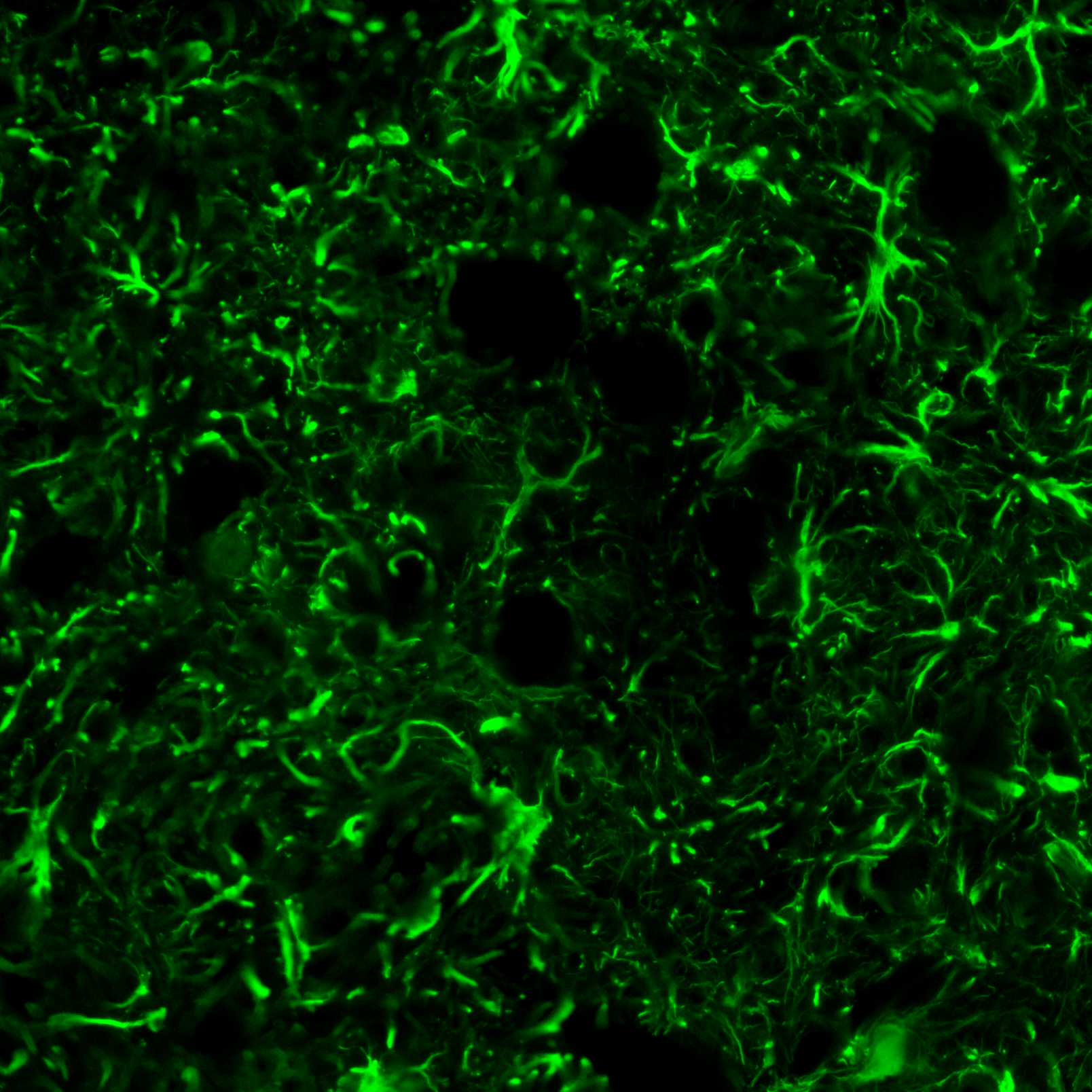

Supplement: Supplementary file 11 — Source data Fig. 7 [file 44321_2025_323_MOESM11_ESM.zip › Figure 7/7I/ISO IBA1488-GFAP555 40X 2_RGB_488.tif]

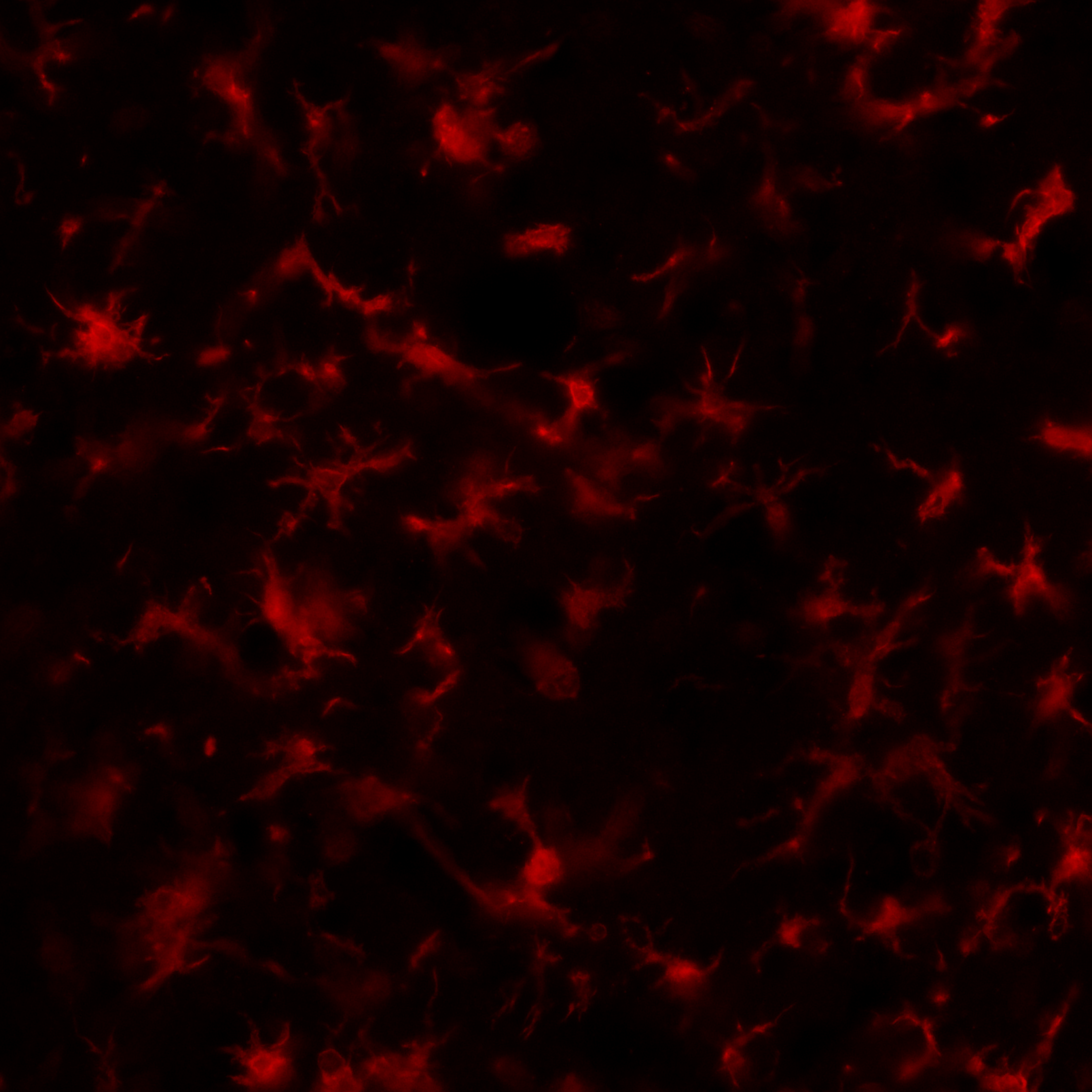

Supplement: Supplementary file 11 — Source data Fig. 7 [file 44321_2025_323_MOESM11_ESM.zip › Figure 7/7I/ISO IBA1488-GFAP555 40X 2_RGB_555.tif]

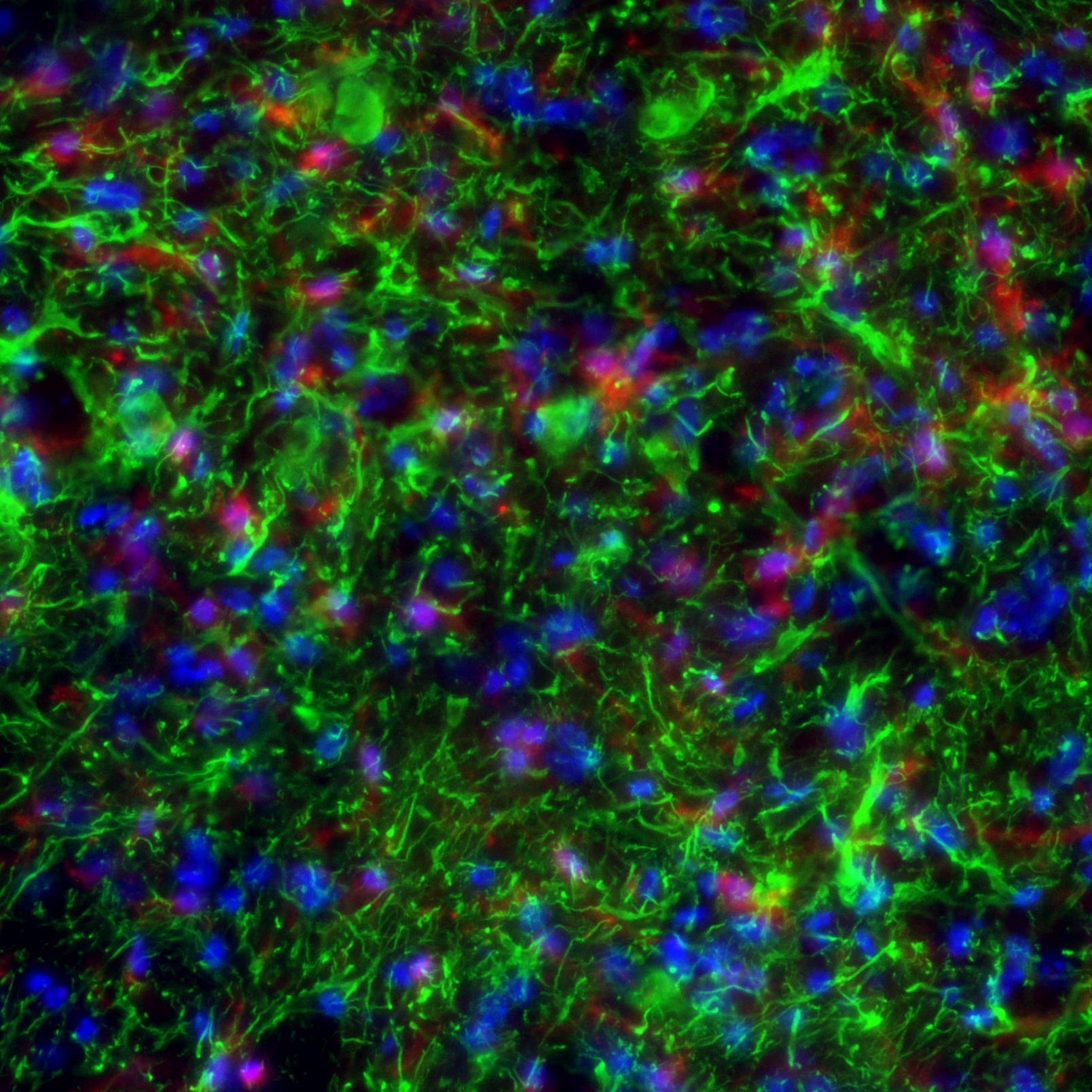

Supplement: Supplementary file 11 — Source data Fig. 7 [file 44321_2025_323_MOESM11_ESM.zip › Figure 7/7I/VEH IBA1488-GFAP1555 40X 4_RGB.tif]

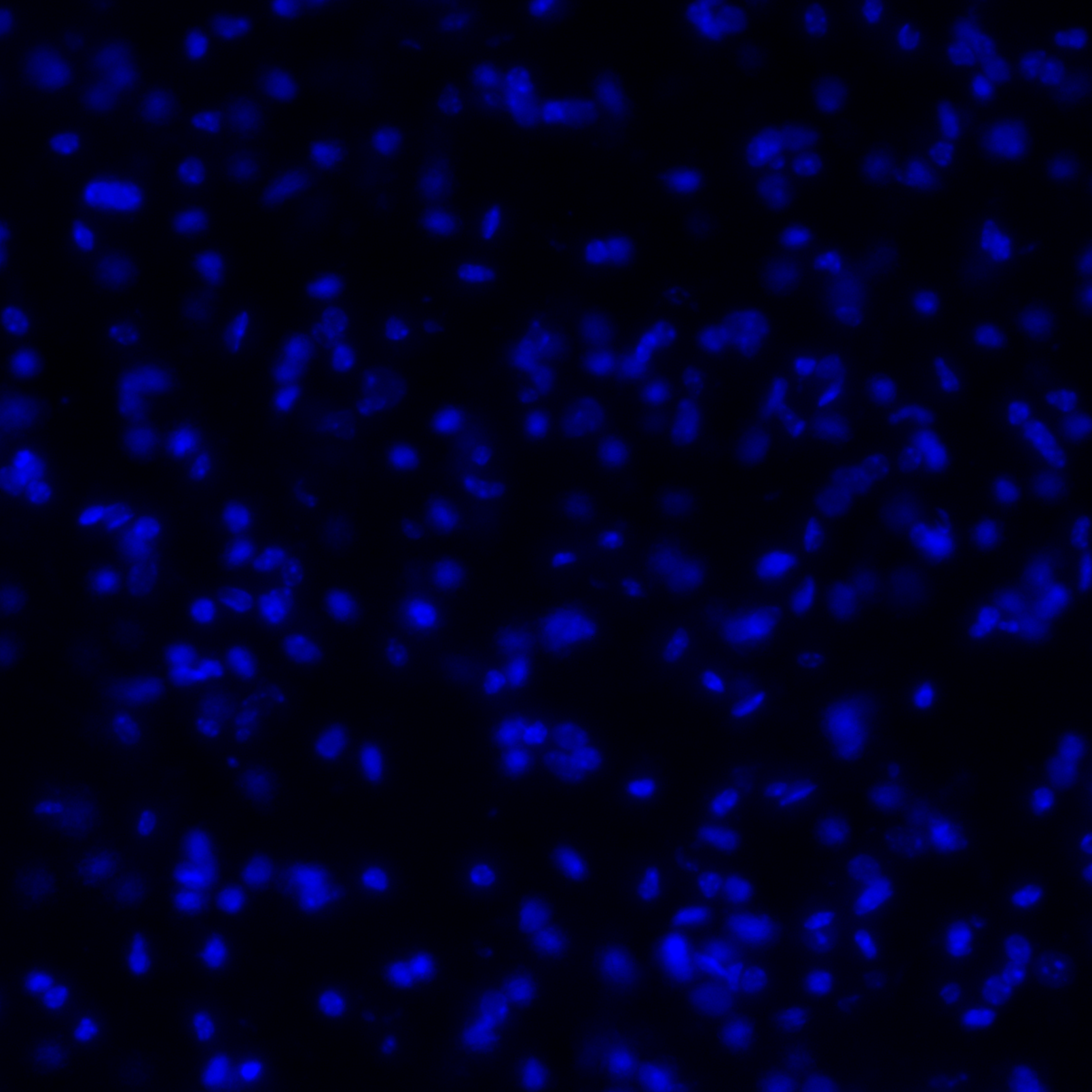

Supplement: Supplementary file 11 — Source data Fig. 7 [file 44321_2025_323_MOESM11_ESM.zip › Figure 7/7I/VEH IBA1488-GFAP1555 40X 4_RGB_405.tif]

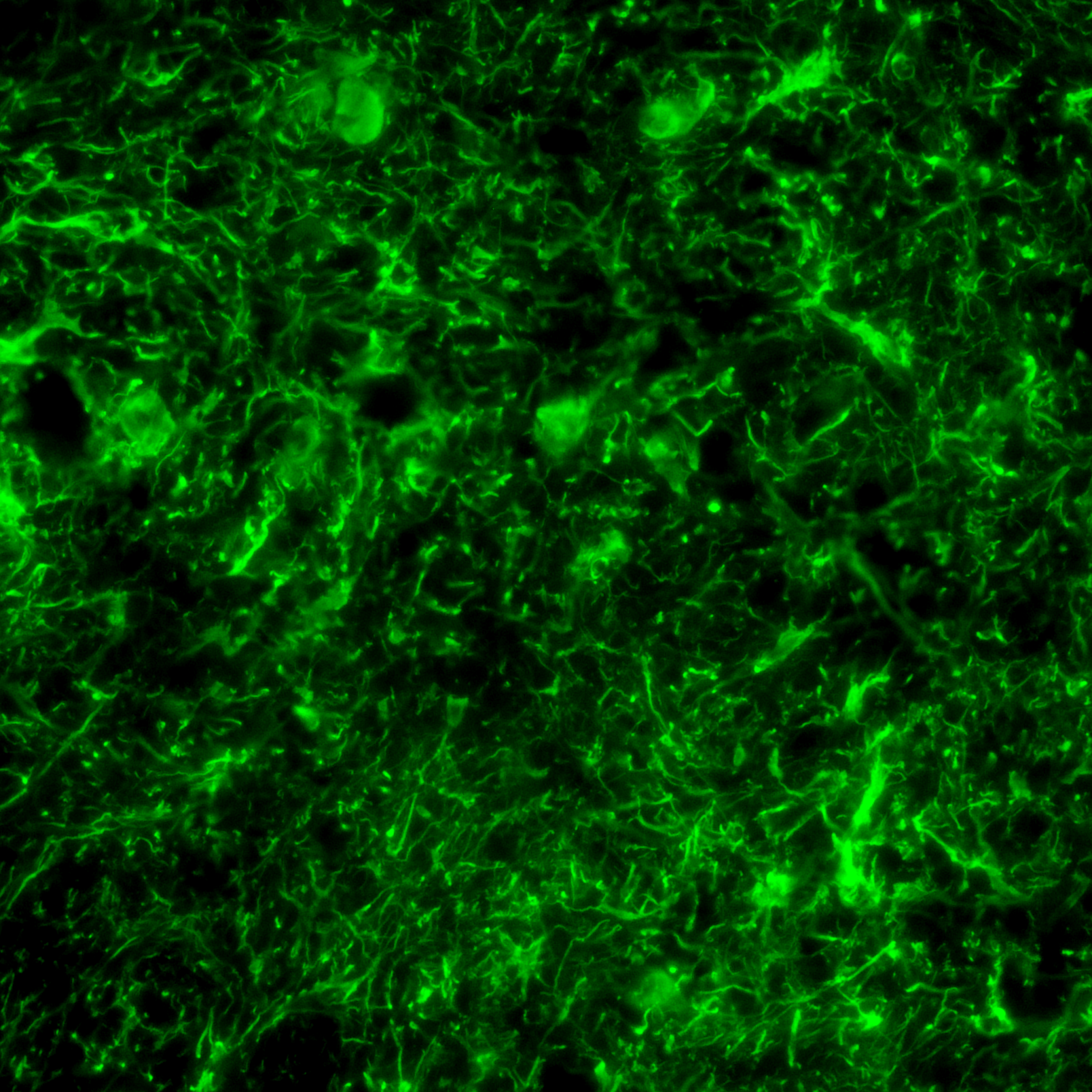

Supplement: Supplementary file 11 — Source data Fig. 7 [file 44321_2025_323_MOESM11_ESM.zip › Figure 7/7I/VEH IBA1488-GFAP1555 40X 4_RGB_488.tif]

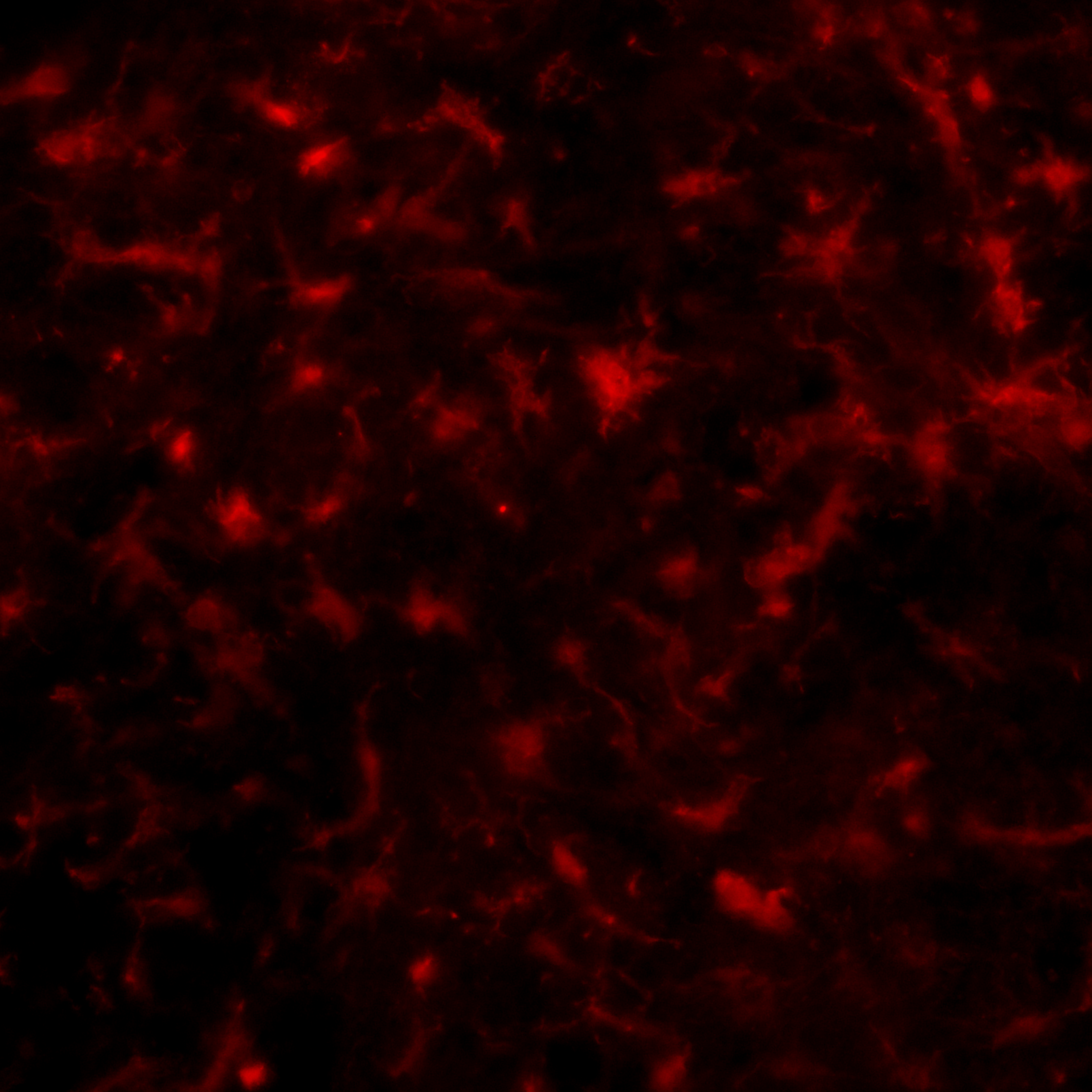

Supplement: Supplementary file 11 — Source data Fig. 7 [file 44321_2025_323_MOESM11_ESM.zip › Figure 7/7I/VEH IBA1488-GFAP1555 40X 4_RGB_555.tif]

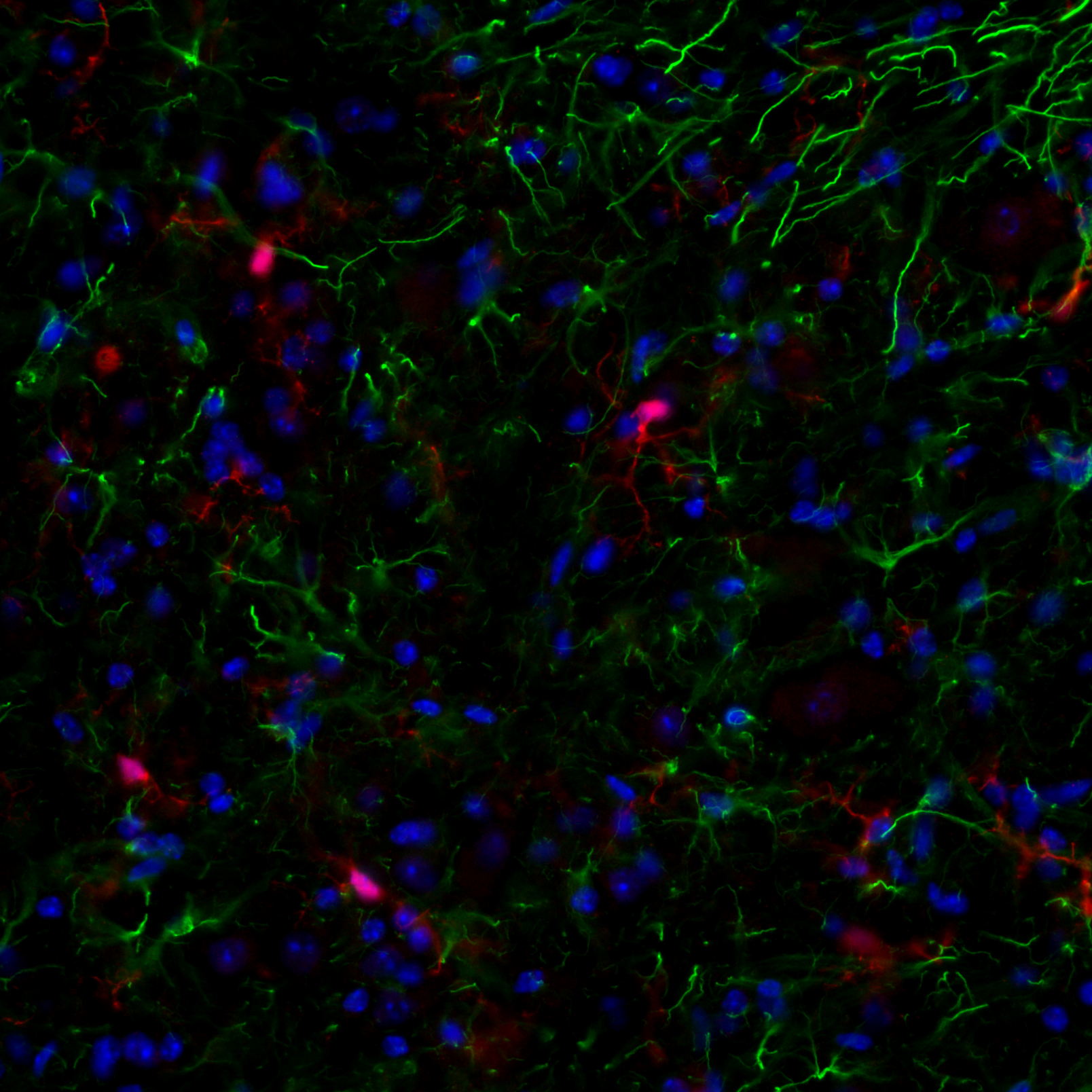

Supplement: Supplementary file 11 — Source data Fig. 7 [file 44321_2025_323_MOESM11_ESM.zip › Figure 7/7I/WT GFAP-IBA 40X 1_RGB.tif]

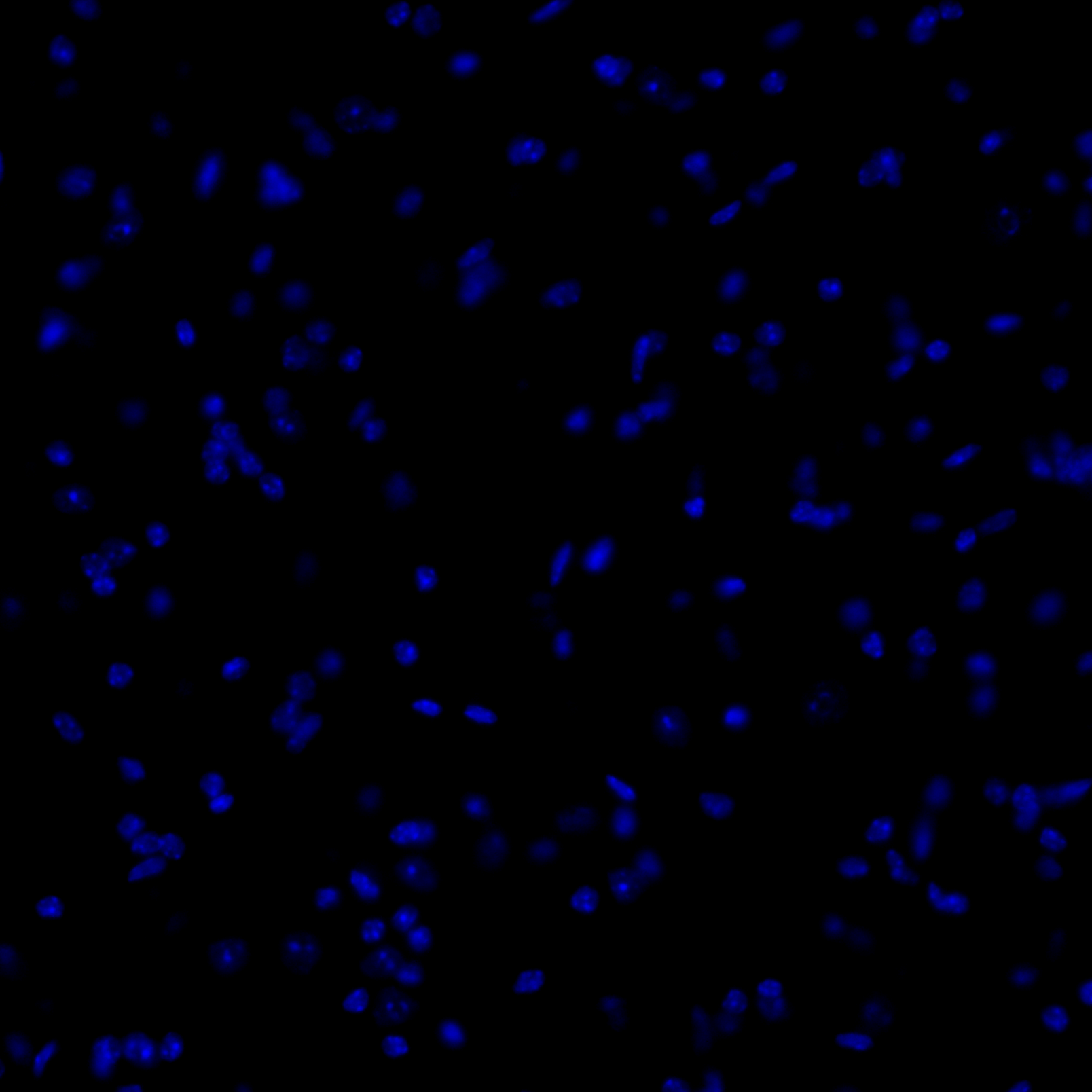

Supplement: Supplementary file 11 — Source data Fig. 7 [file 44321_2025_323_MOESM11_ESM.zip › Figure 7/7I/WT GFAP-IBA 40X 1_RGB_405.tif]

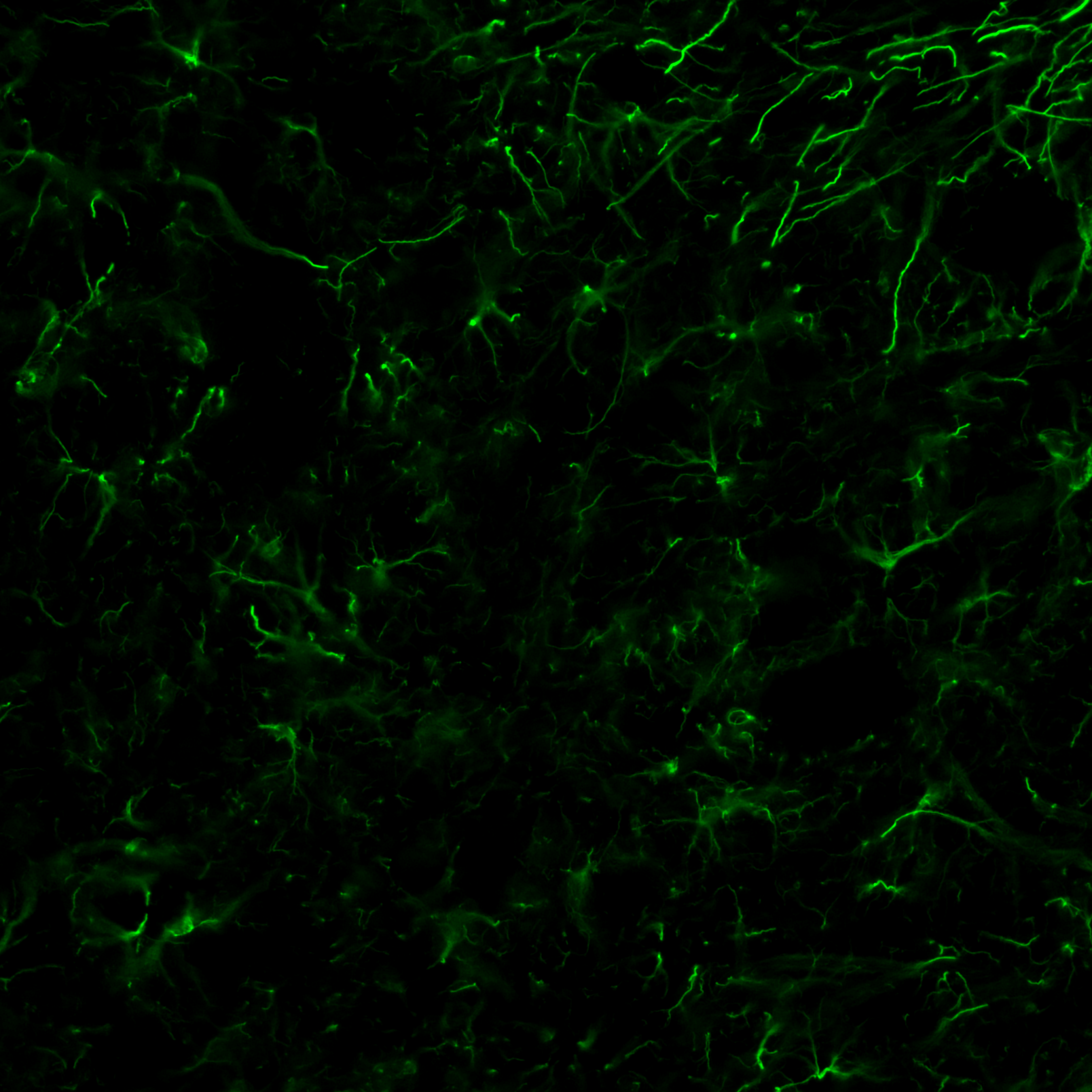

Supplement: Supplementary file 11 — Source data Fig. 7 [file 44321_2025_323_MOESM11_ESM.zip › Figure 7/7I/WT GFAP-IBA 40X 1_RGB_488.tif]

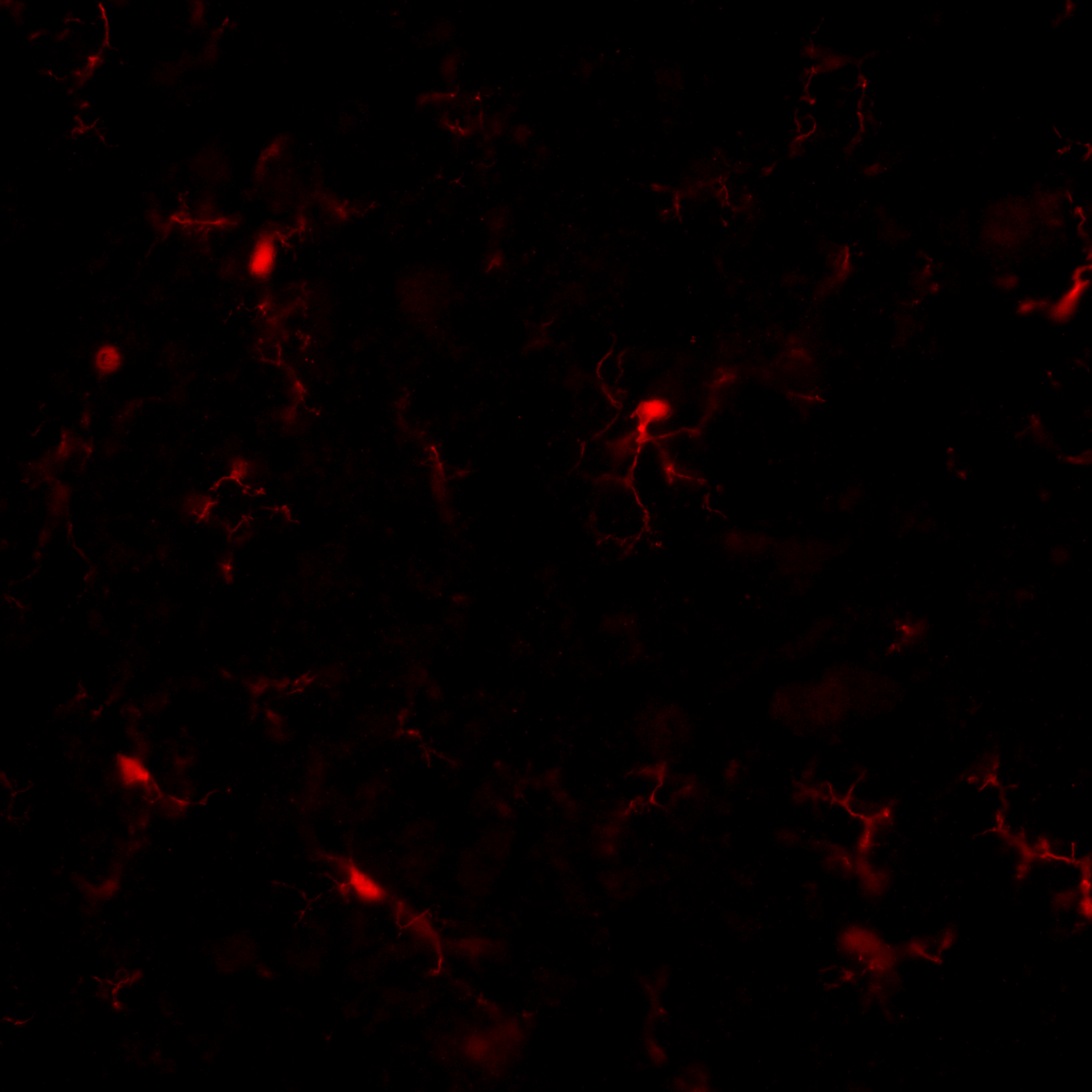

Supplement: Supplementary file 11 — Source data Fig. 7 [file 44321_2025_323_MOESM11_ESM.zip › Figure 7/7I/WT GFAP-IBA 40X 1_RGB_594.tif]

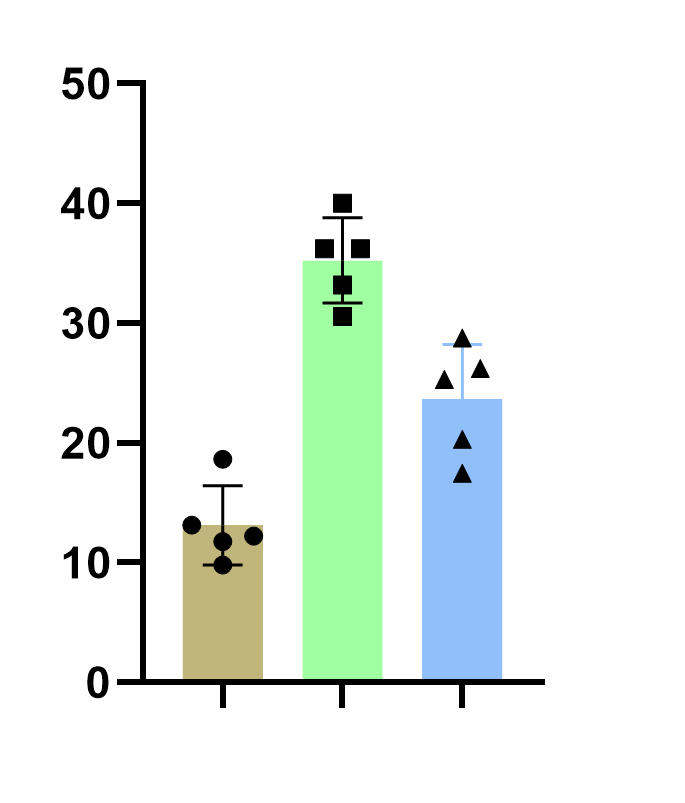

Supplement: Supplementary file 11 — Source data Fig. 7 [file 44321_2025_323_MOESM11_ESM.zip › Figure 7/7J/IBA1.tif]

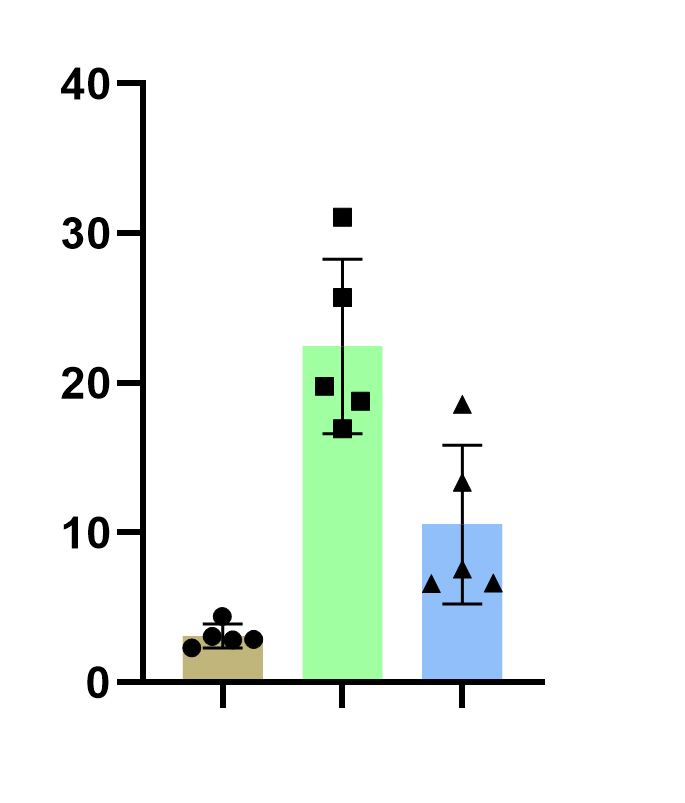

Supplement: Supplementary file 11 — Source data Fig. 7 [file 44321_2025_323_MOESM11_ESM.zip › Figure 7/7K/GFAP.tif]

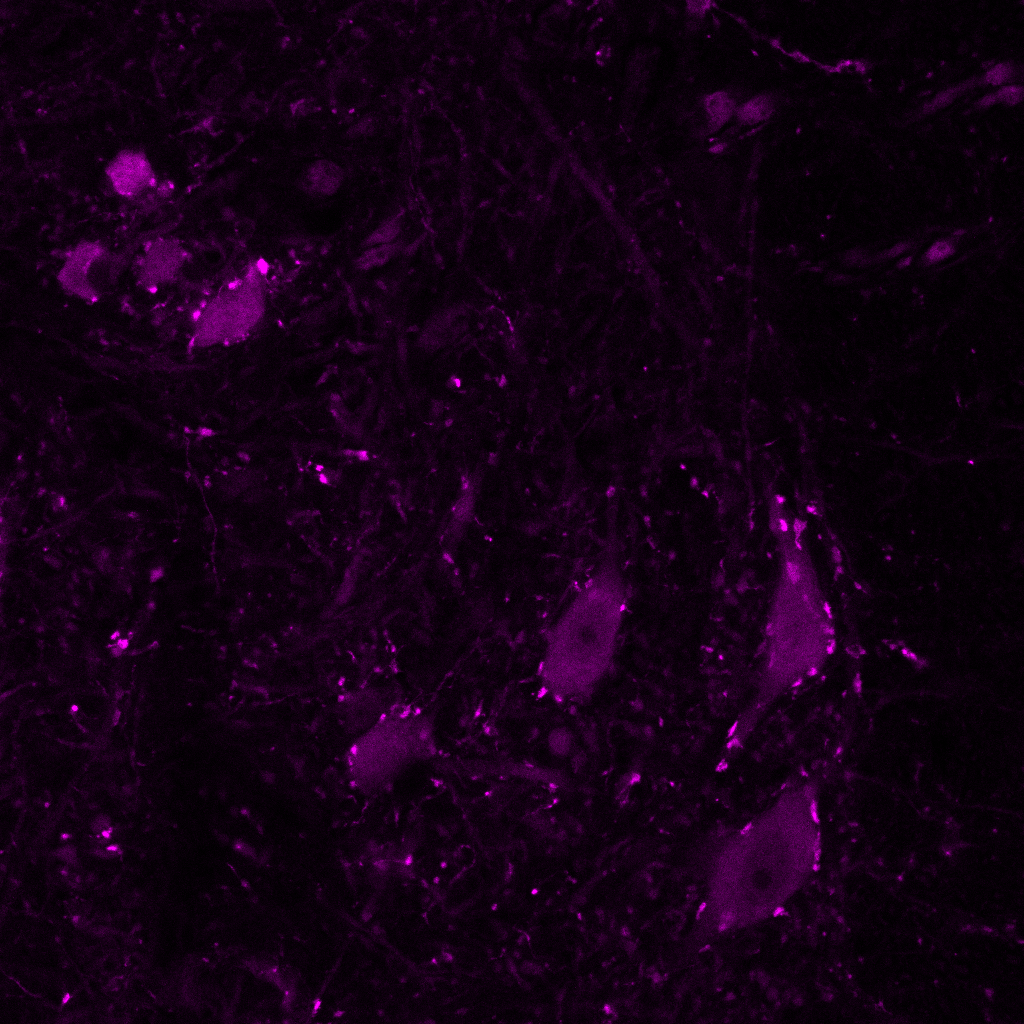

Supplement: Supplementary file 11 — Source data Fig. 7 [file 44321_2025_323_MOESM11_ESM.zip › Figure 7/7L/ISO LAMP1-MITO-CHAT 40X chat.tif]

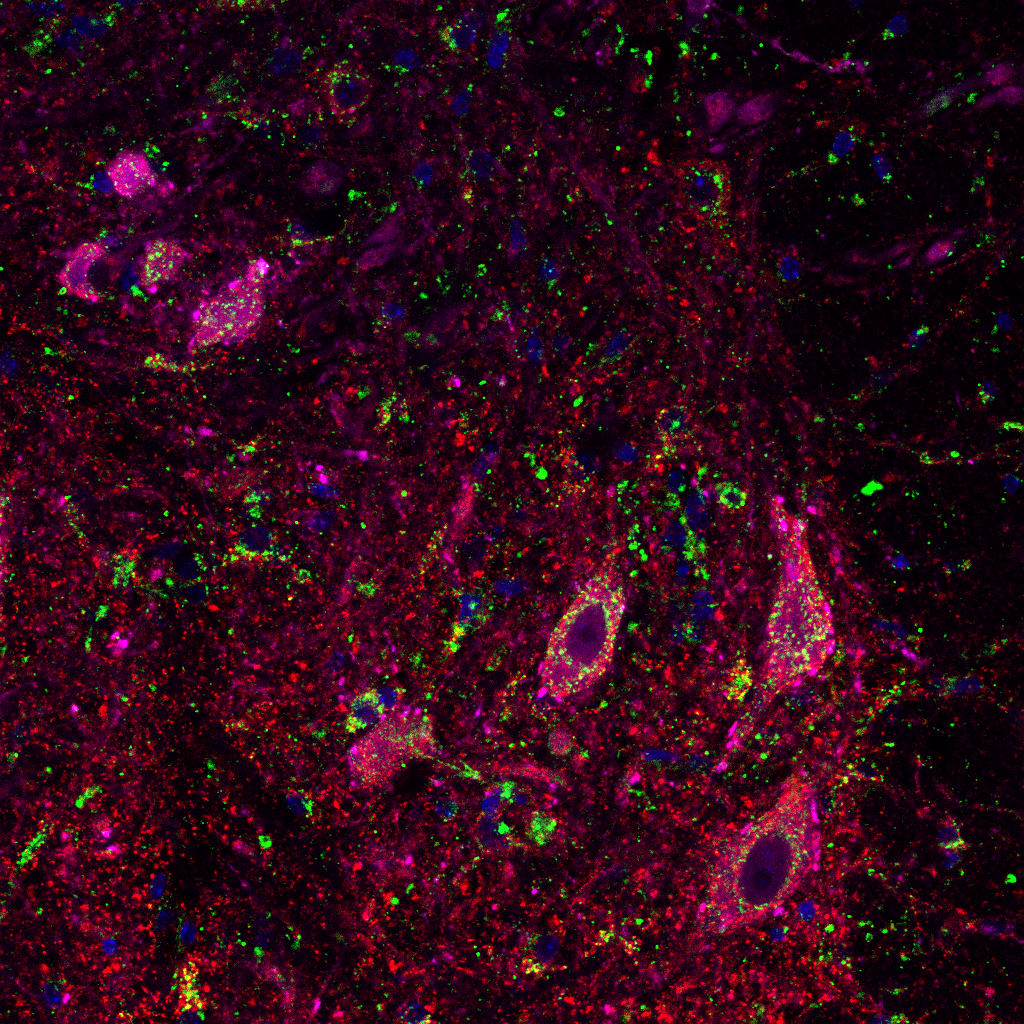

Supplement: Supplementary file 11 — Source data Fig. 7 [file 44321_2025_323_MOESM11_ESM.zip › Figure 7/7L/ISO LAMP1-MITO-CHAT 40X Merge.tif]

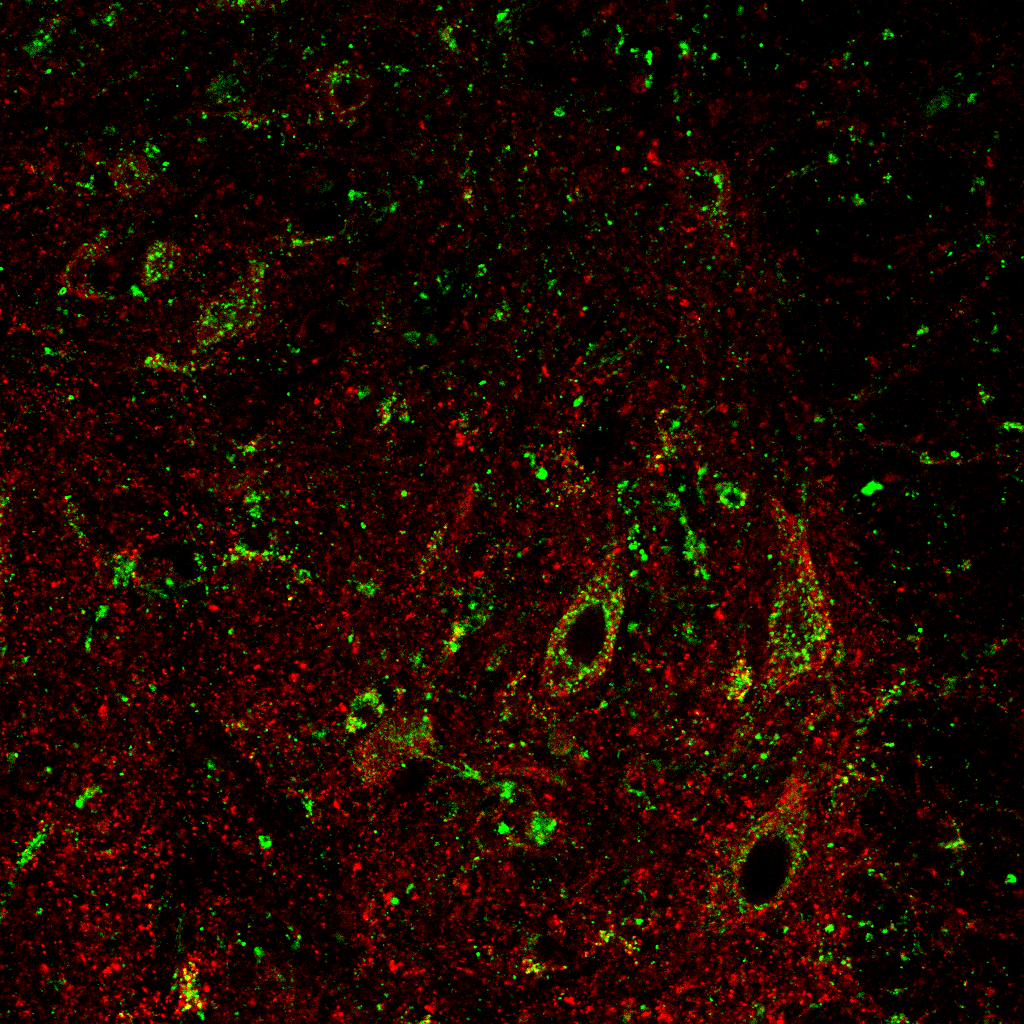

Supplement: Supplementary file 11 — Source data Fig. 7 [file 44321_2025_323_MOESM11_ESM.zip › Figure 7/7L/ISO LAMP1-MITO-CHAT.tif]

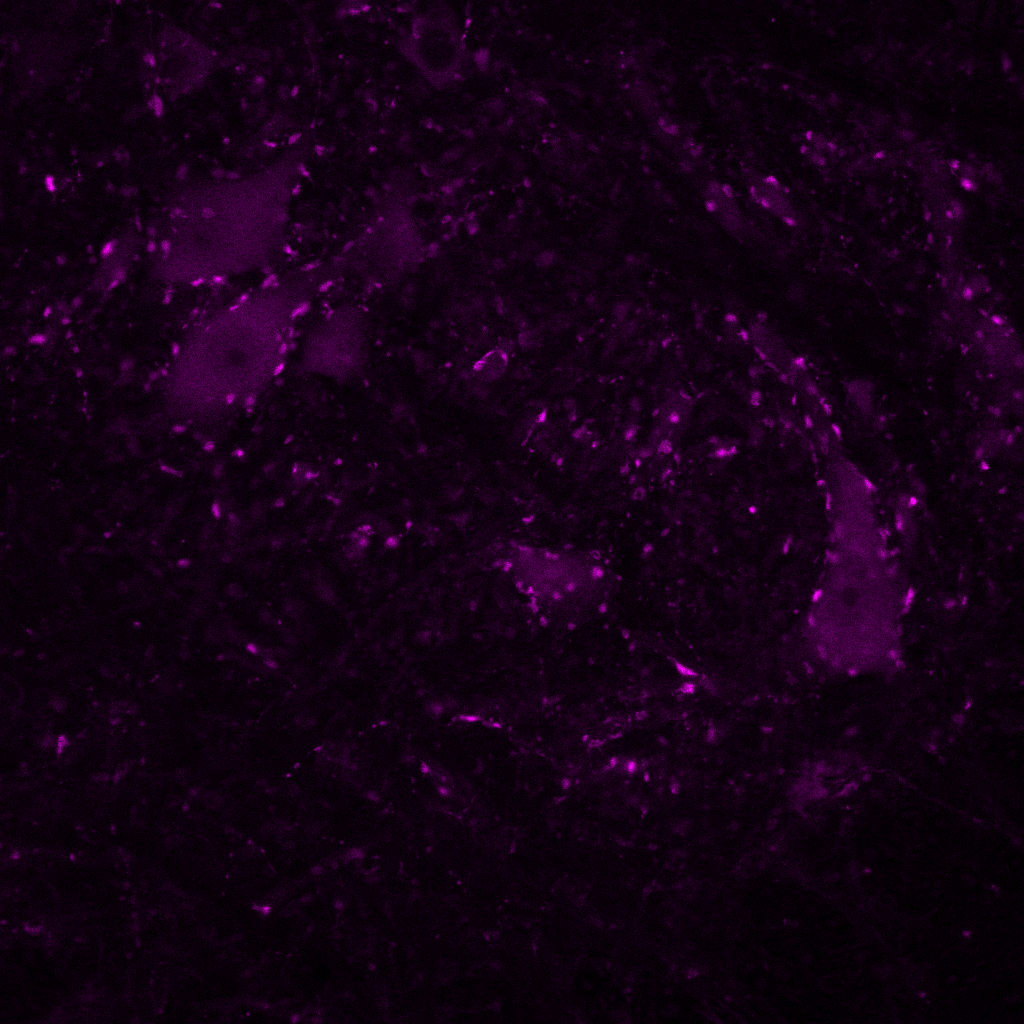

Supplement: Supplementary file 11 — Source data Fig. 7 [file 44321_2025_323_MOESM11_ESM.zip › Figure 7/7L/VEH LAMP1-MITO-CHAT 40X chat.tif]

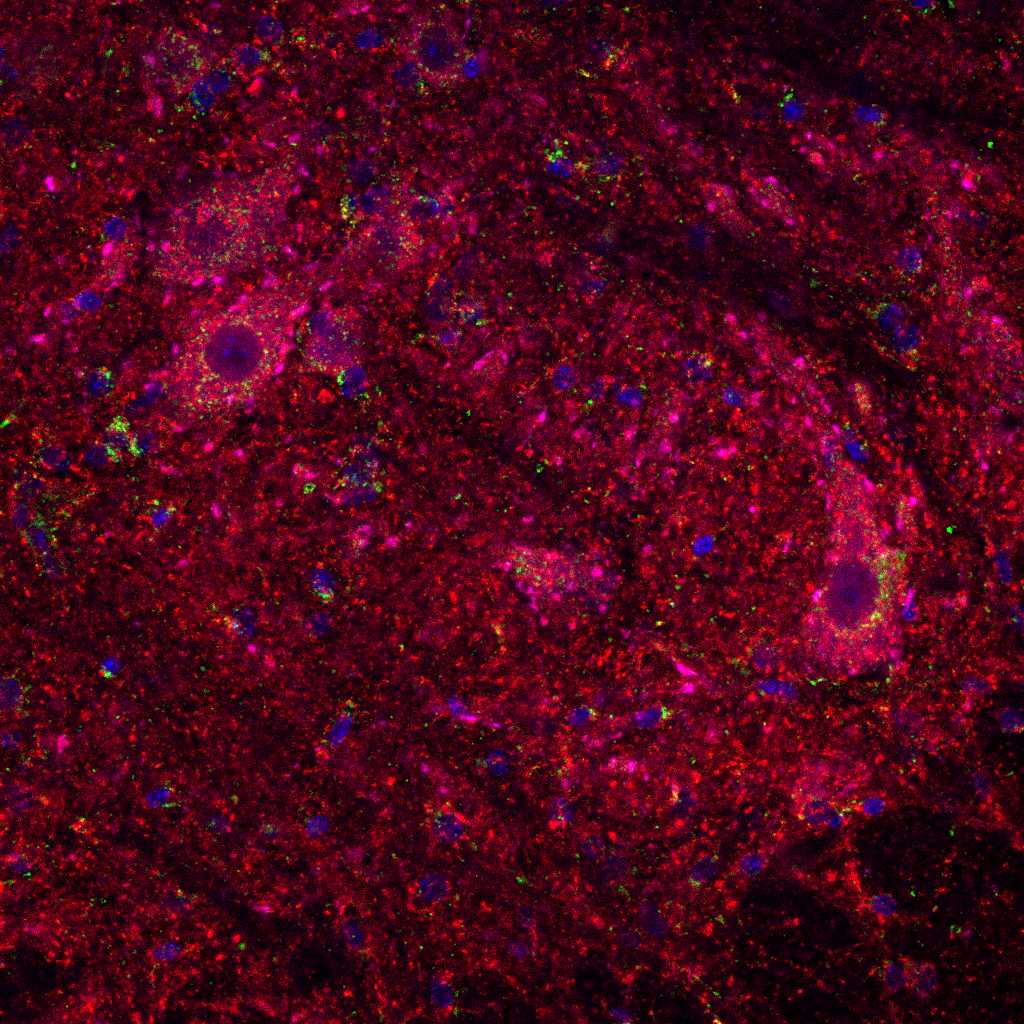

Supplement: Supplementary file 11 — Source data Fig. 7 [file 44321_2025_323_MOESM11_ESM.zip › Figure 7/7L/VEH LAMP1-MITO-CHAT 40X Merge.tif]

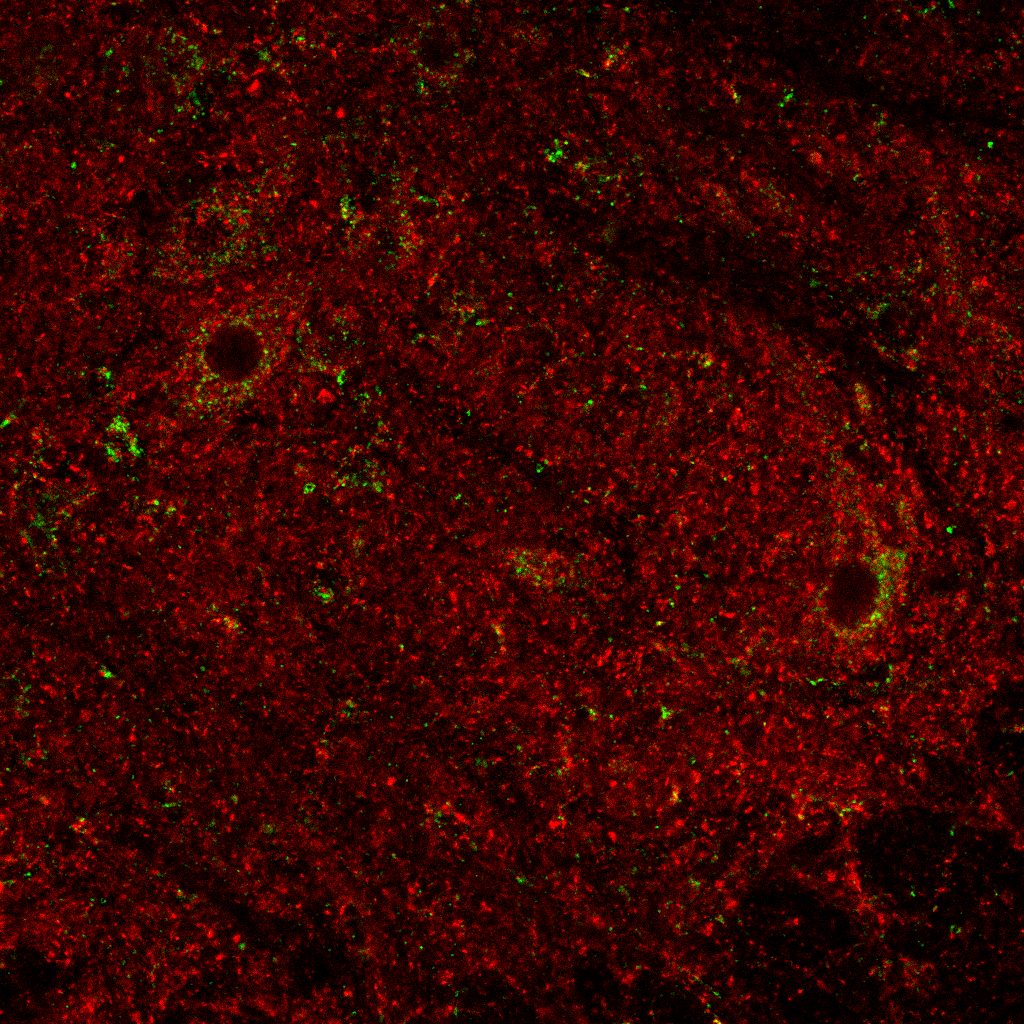

Supplement: Supplementary file 11 — Source data Fig. 7 [file 44321_2025_323_MOESM11_ESM.zip › Figure 7/7L/VEH LAMP1-MITO-CHAT.tif]

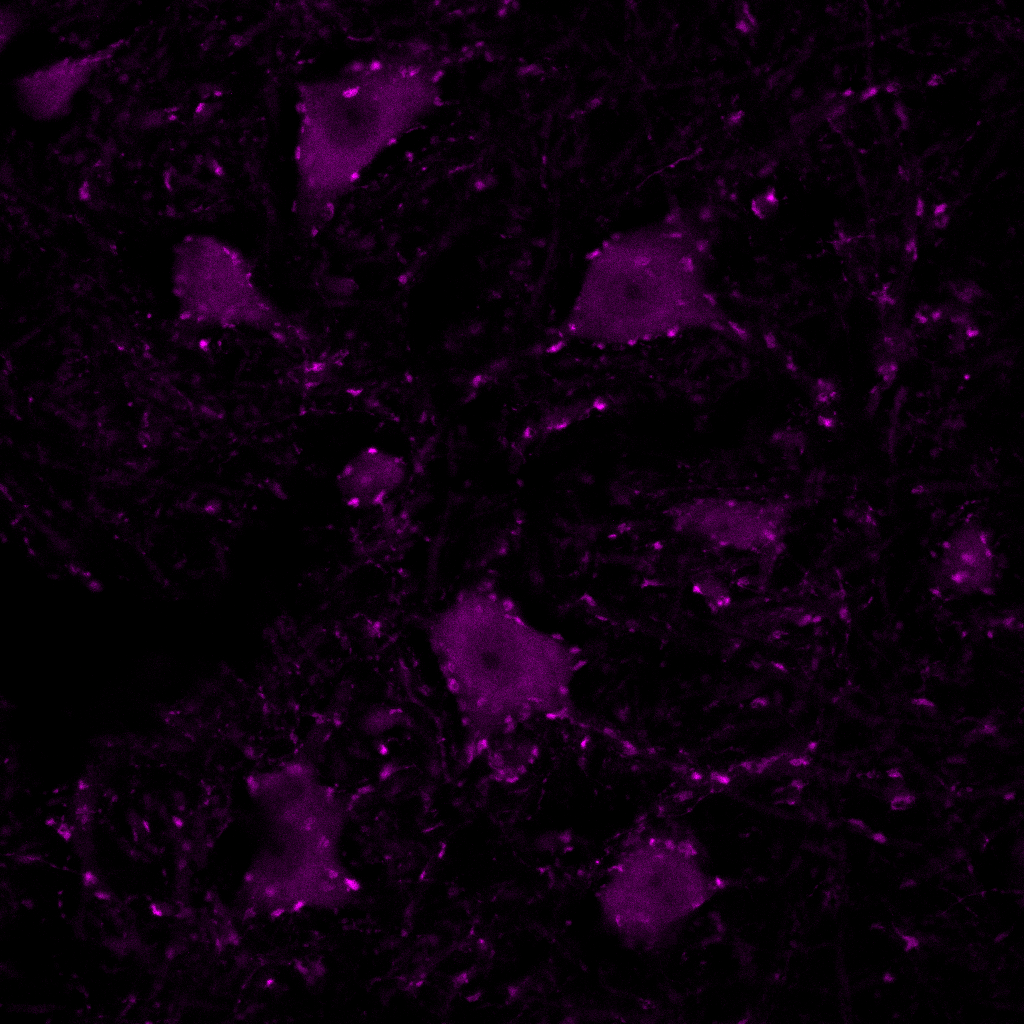

Supplement: Supplementary file 11 — Source data Fig. 7 [file 44321_2025_323_MOESM11_ESM.zip › Figure 7/7L/WT LAMP1-MITO-CHAT 40X chat.tif]

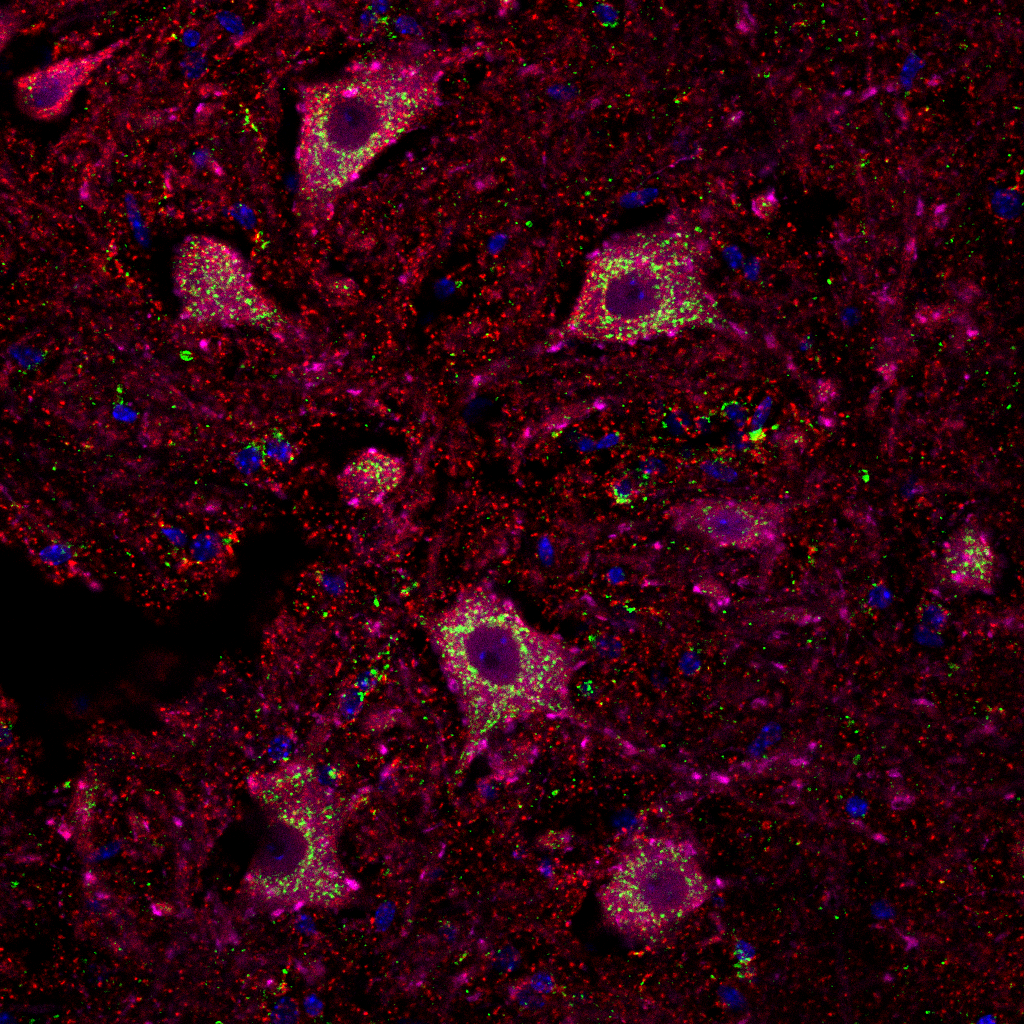

Supplement: Supplementary file 11 — Source data Fig. 7 [file 44321_2025_323_MOESM11_ESM.zip › Figure 7/7L/WT LAMP1-MITO-CHAT 40X Merge.tif]

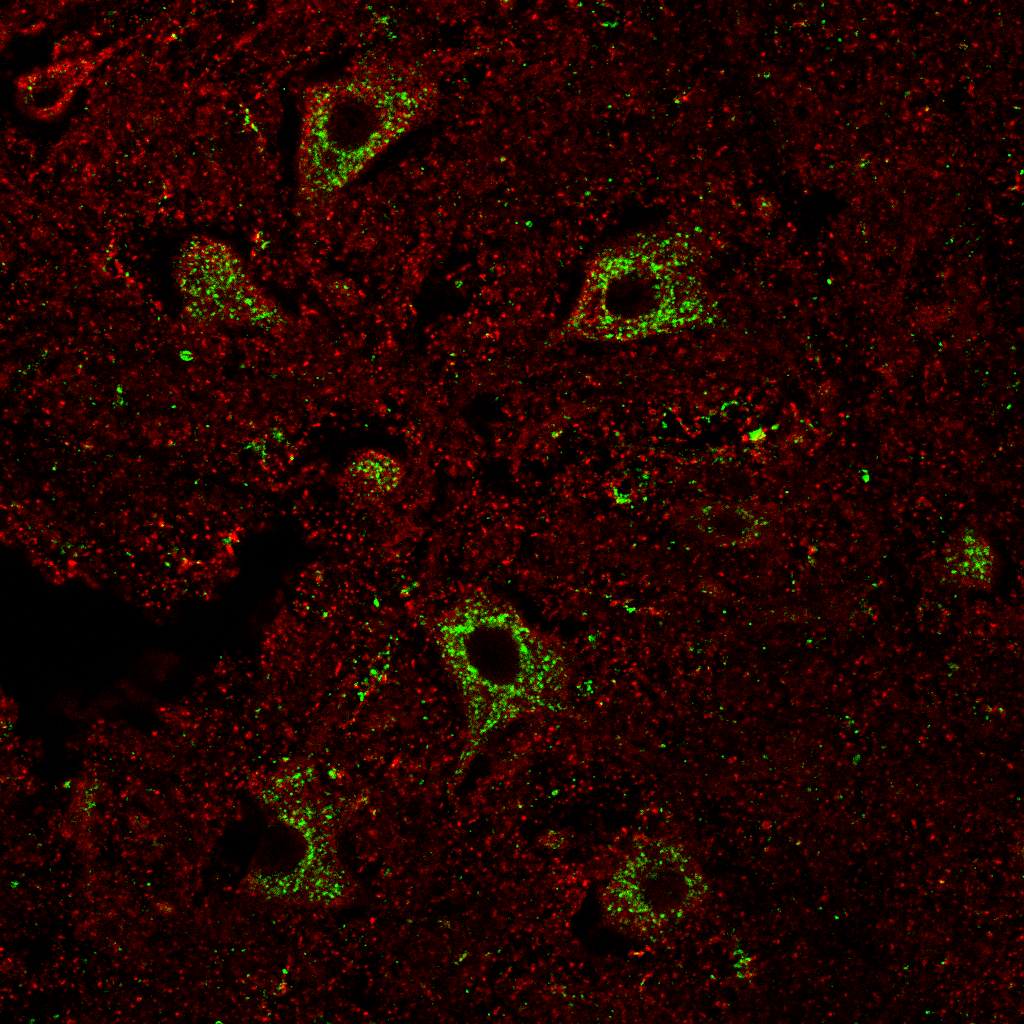

Supplement: Supplementary file 11 — Source data Fig. 7 [file 44321_2025_323_MOESM11_ESM.zip › Figure 7/7L/WT LAMP1-MITO-CHAT.tif]

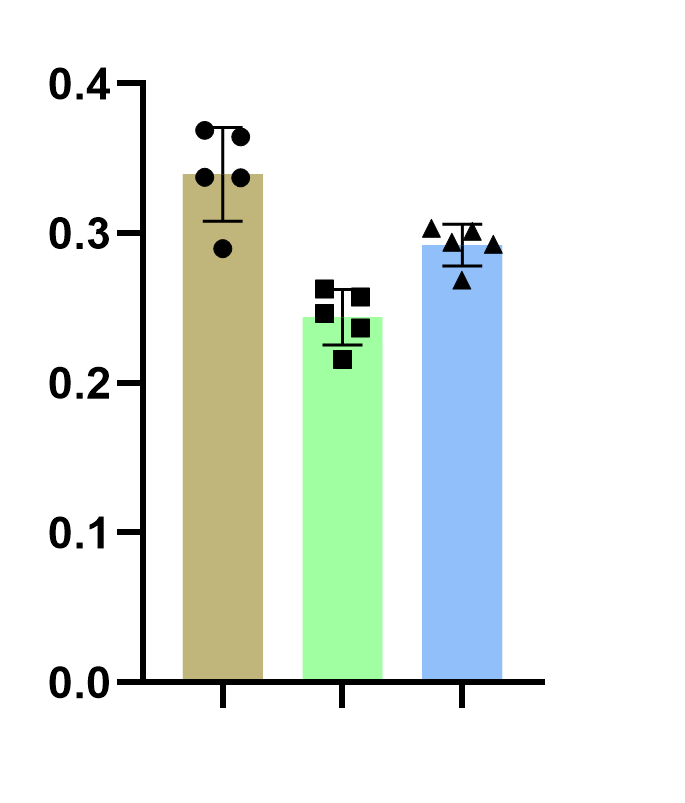

Supplement: Supplementary file 11 — Source data Fig. 7 [file 44321_2025_323_MOESM11_ESM.zip › Figure 7/7M/MTCO2-LAMP2.tif]

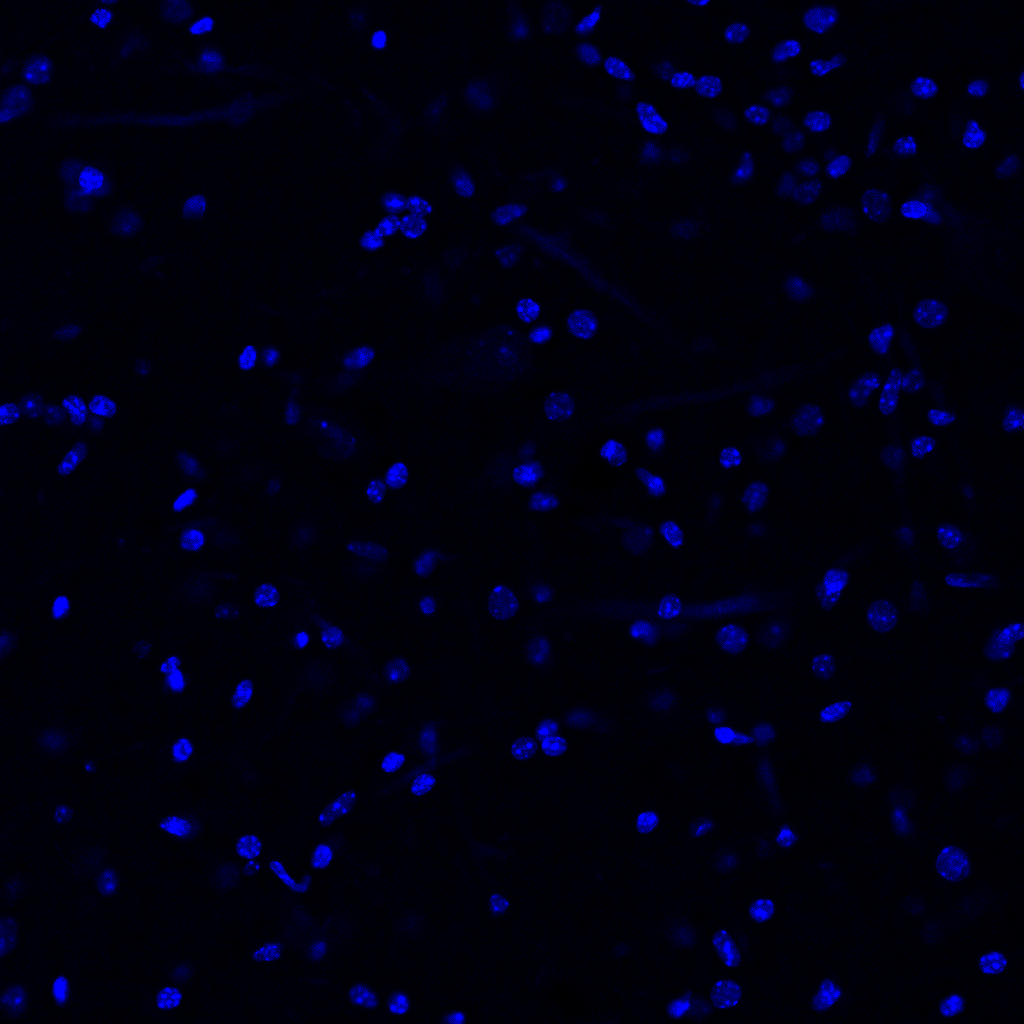

Supplement: Supplementary file 11 — Source data Fig. 7 [file 44321_2025_323_MOESM11_ESM.zip › Figure 7/7N/ISO PUB 40X 2-Image Export-34_c3 (1).tif]

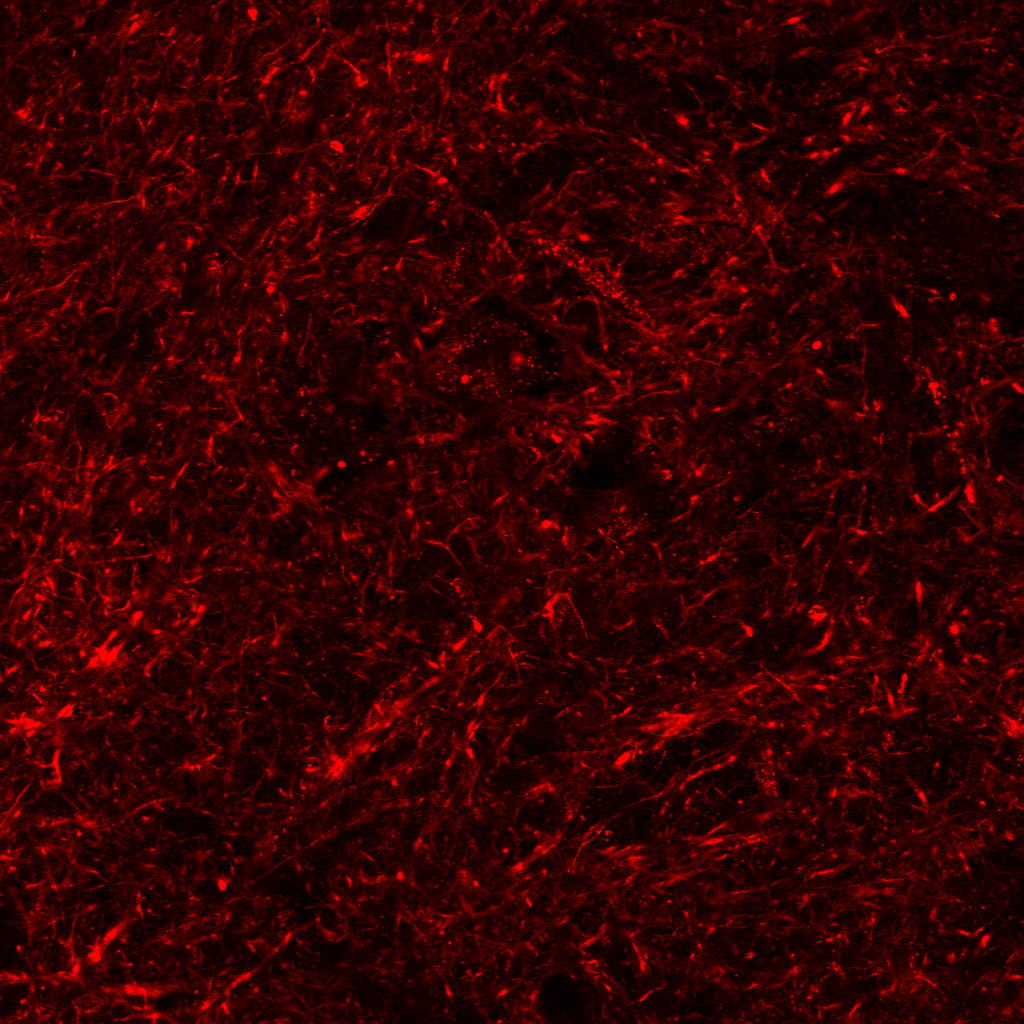

Supplement: Supplementary file 11 — Source data Fig. 7 [file 44321_2025_323_MOESM11_ESM.zip › Figure 7/7N/ISO PUB 40X 2-Image Export-34_c3 (2).tif]

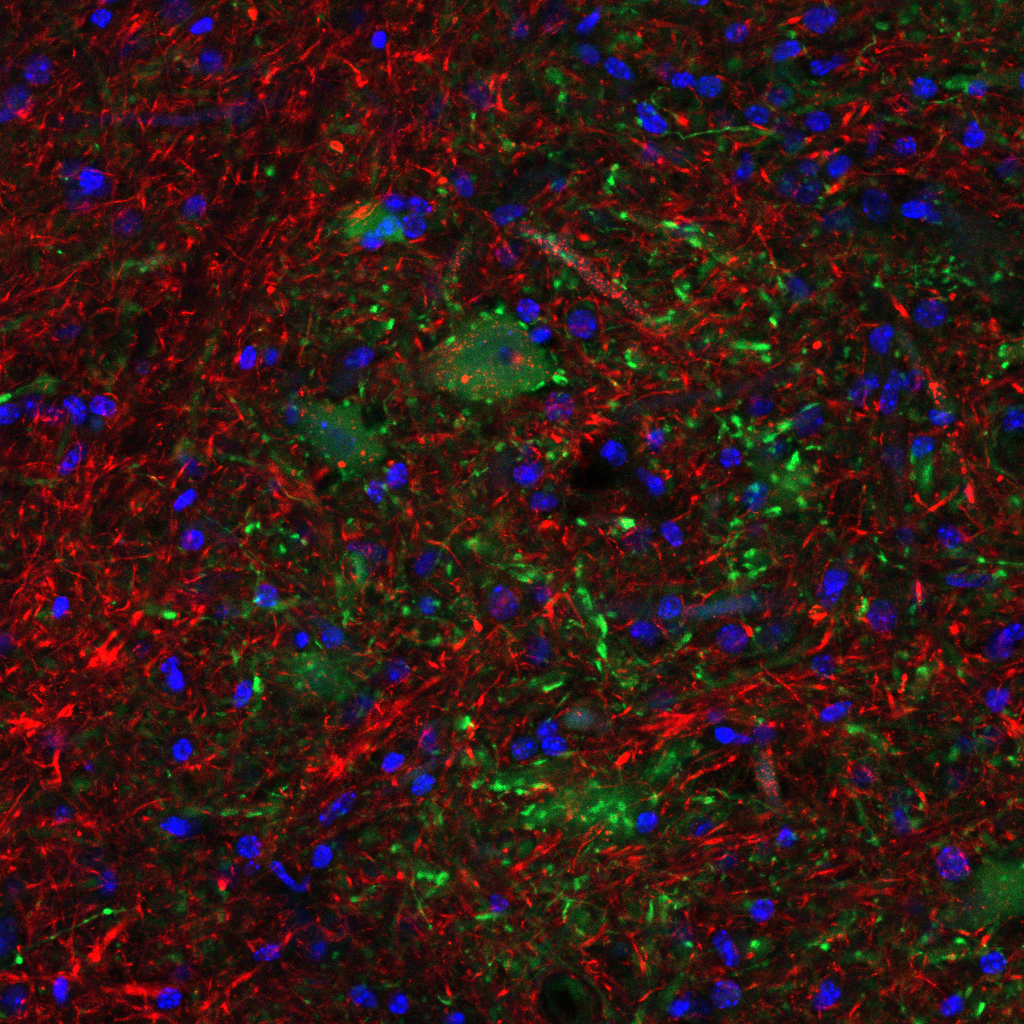

Supplement: Supplementary file 11 — Source data Fig. 7 [file 44321_2025_323_MOESM11_ESM.zip › Figure 7/7N/ISO PUB 40X 2-Image Export-34_c3 (3).tif]

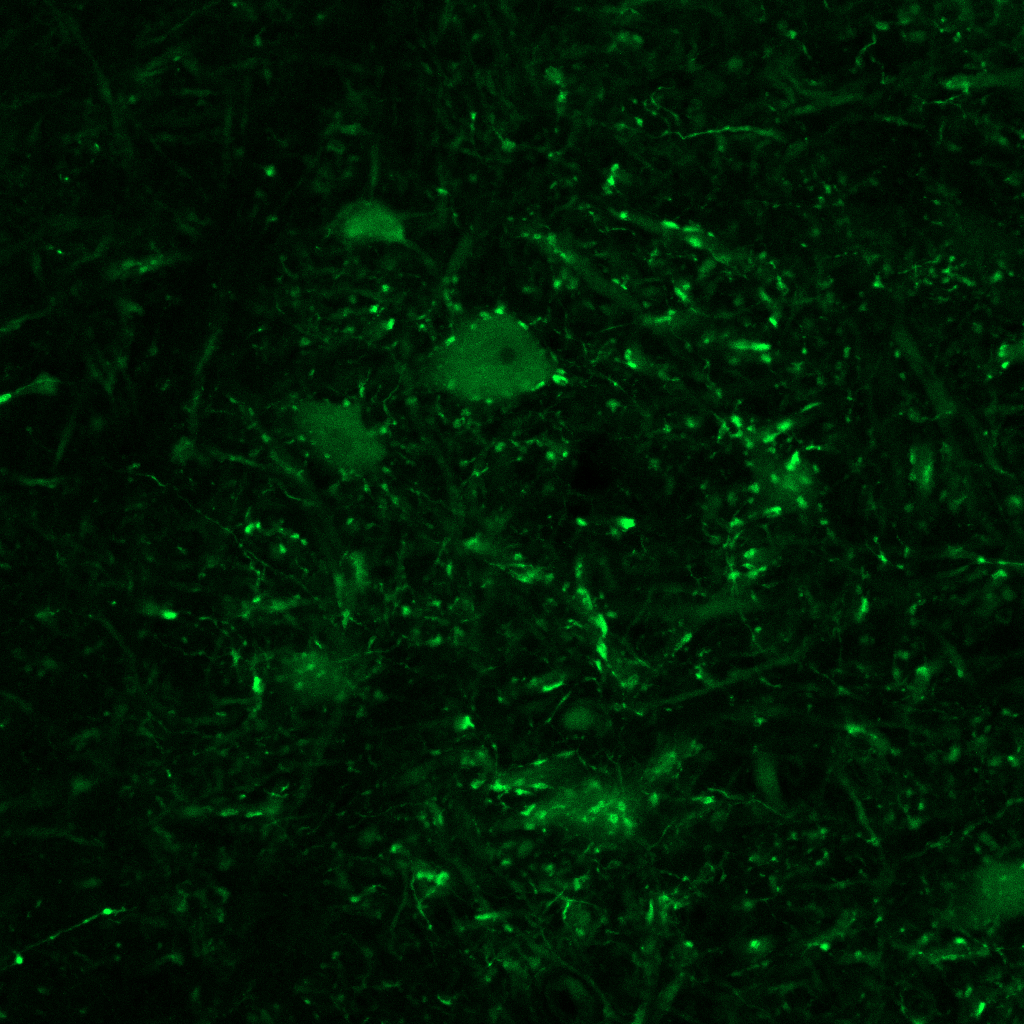

Supplement: Supplementary file 11 — Source data Fig. 7 [file 44321_2025_323_MOESM11_ESM.zip › Figure 7/7N/ISO PUB 40X 2-Image Export-34_c3 (4).tif]

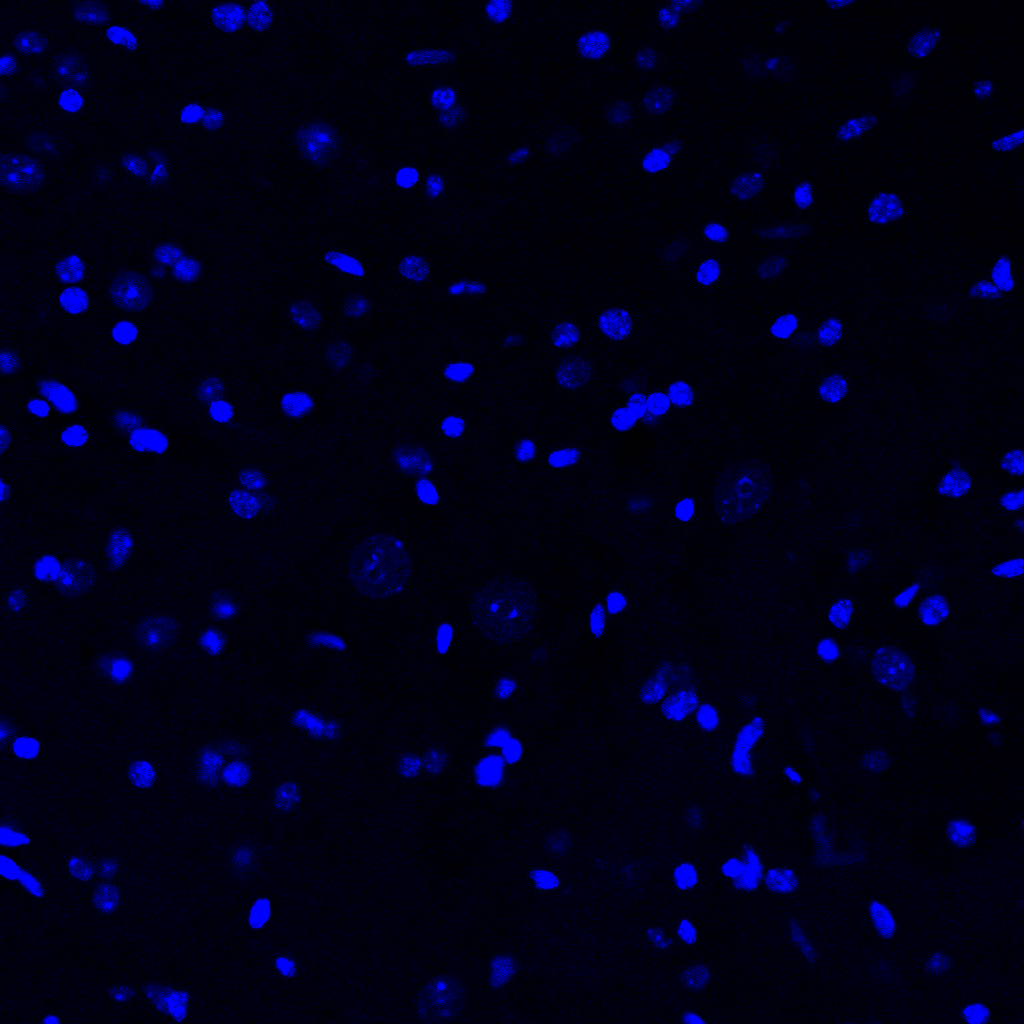

Supplement: Supplementary file 11 — Source data Fig. 7 [file 44321_2025_323_MOESM11_ESM.zip › Figure 7/7N/VEH PUB 40X 1-Image Export-13_c3 (1).tif]

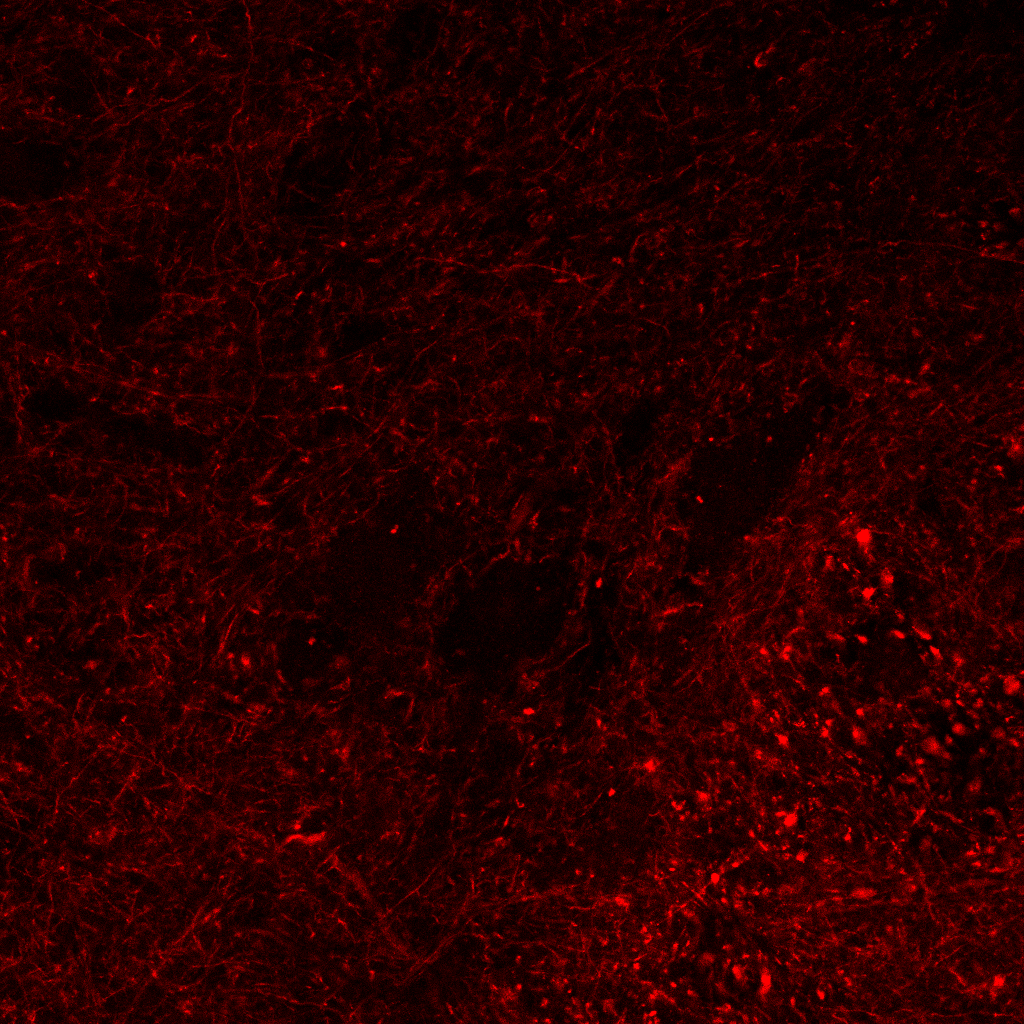

Supplement: Supplementary file 11 — Source data Fig. 7 [file 44321_2025_323_MOESM11_ESM.zip › Figure 7/7N/VEH PUB 40X 1-Image Export-13_c3 (2).tif]

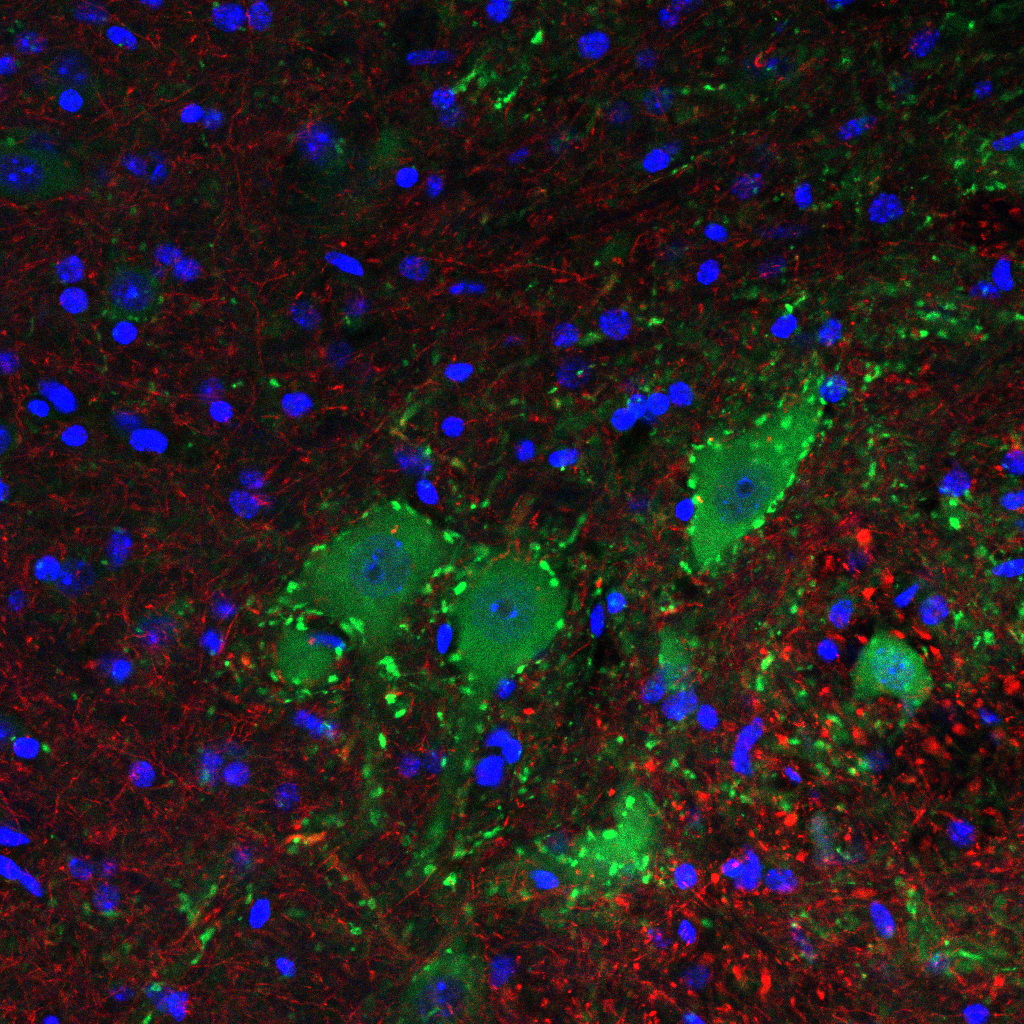

Supplement: Supplementary file 11 — Source data Fig. 7 [file 44321_2025_323_MOESM11_ESM.zip › Figure 7/7N/VEH PUB 40X 1-Image Export-13_c3 (3).tif]

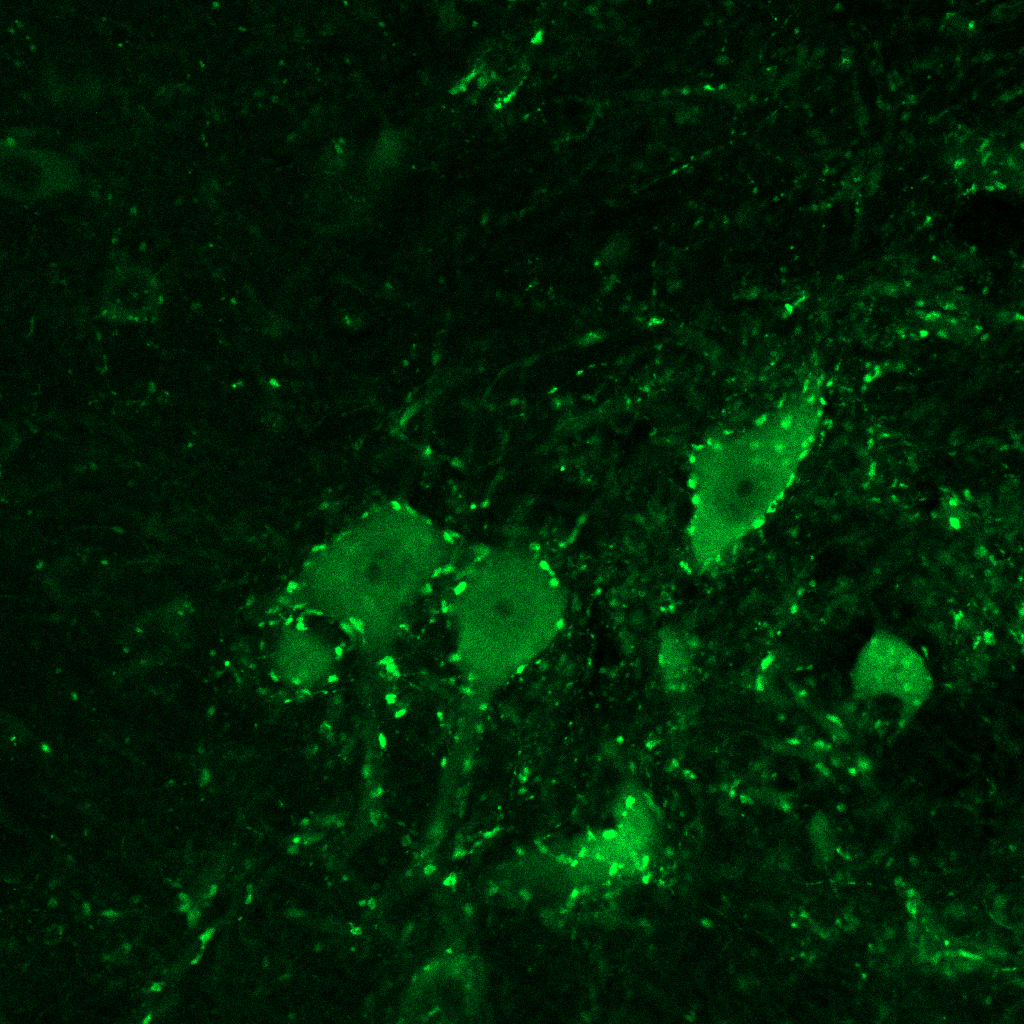

Supplement: Supplementary file 11 — Source data Fig. 7 [file 44321_2025_323_MOESM11_ESM.zip › Figure 7/7N/VEH PUB 40X 1-Image Export-13_c3 (4).tif]

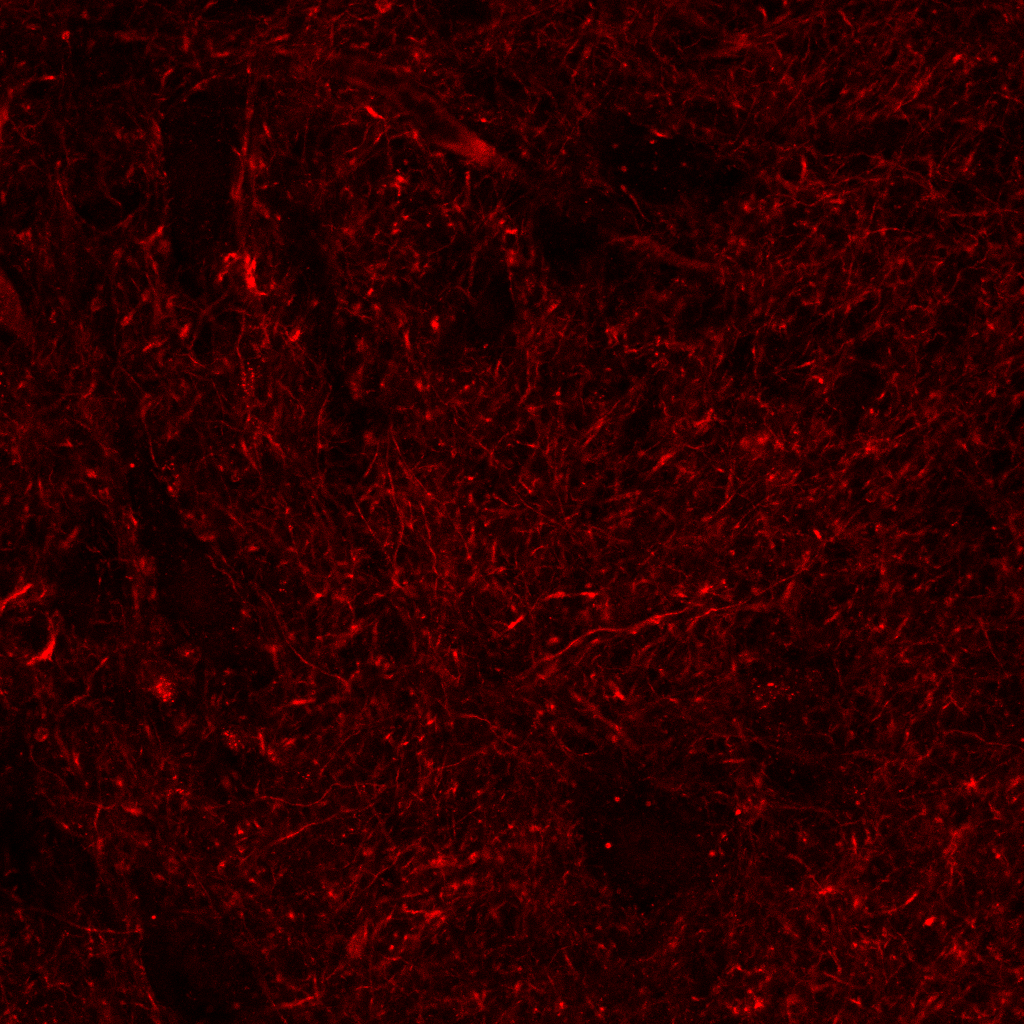

Supplement: Supplementary file 11 — Source data Fig. 7 [file 44321_2025_323_MOESM11_ESM.zip › Figure 7/7N/WT PUB 40X 1-Image Export-37_c1 (1).tif]

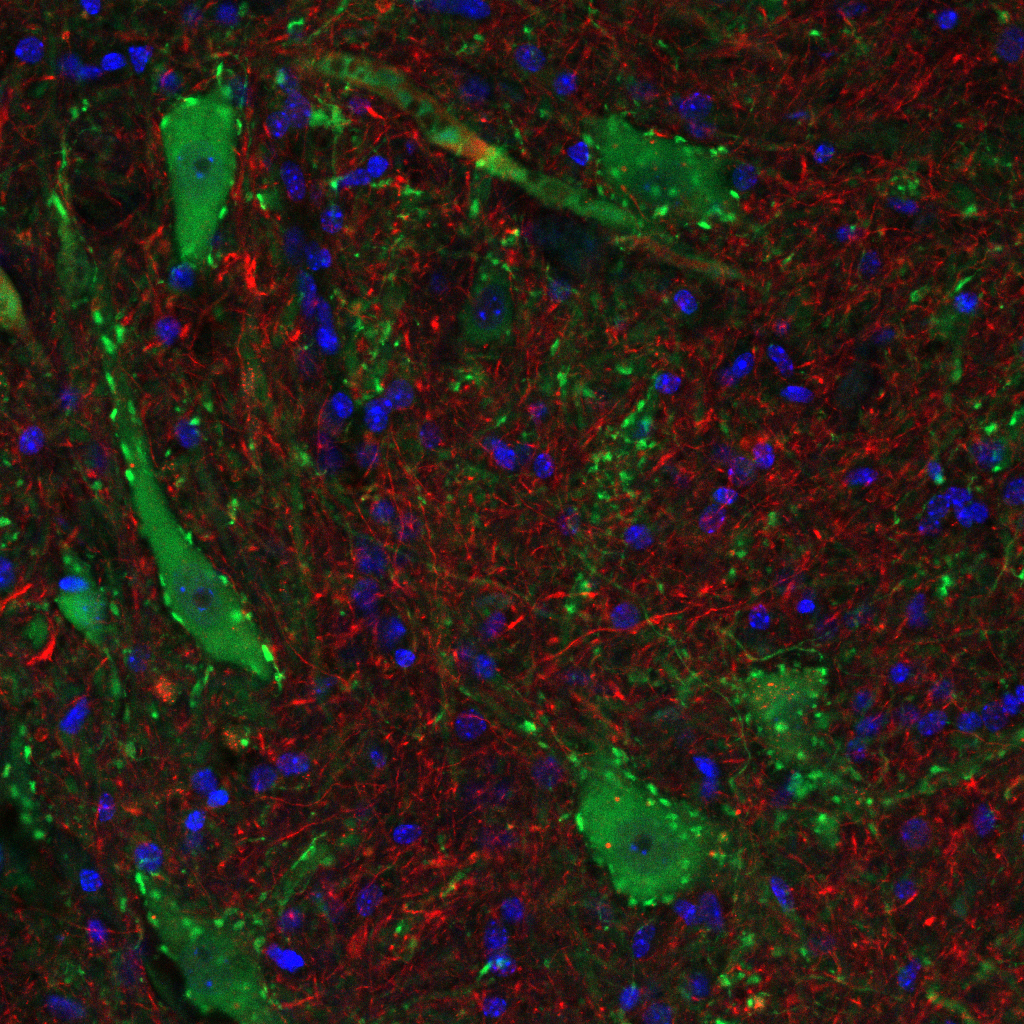

Supplement: Supplementary file 11 — Source data Fig. 7 [file 44321_2025_323_MOESM11_ESM.zip › Figure 7/7N/WT PUB 40X 1-Image Export-37_c1 (2).tif]

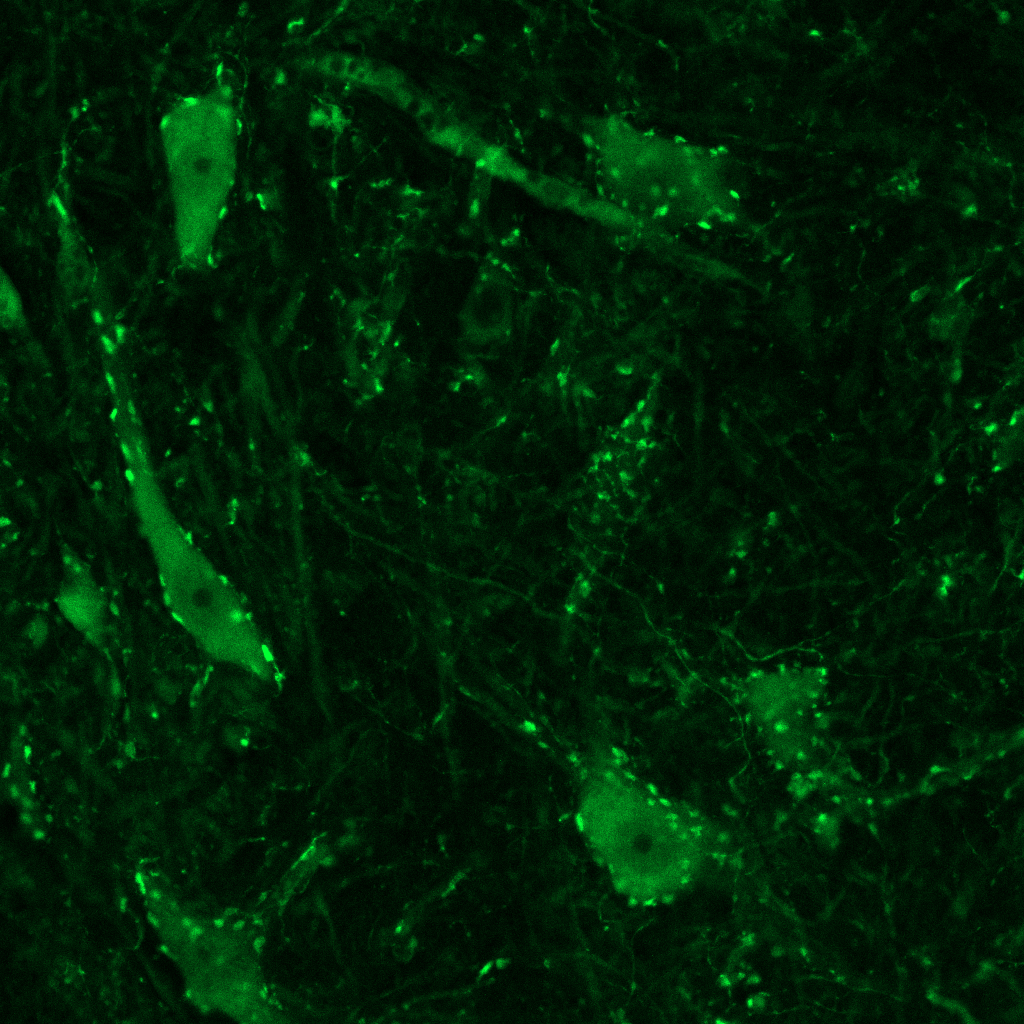

Supplement: Supplementary file 11 — Source data Fig. 7 [file 44321_2025_323_MOESM11_ESM.zip › Figure 7/7N/WT PUB 40X 1-Image Export-37_c1 (3).tif]

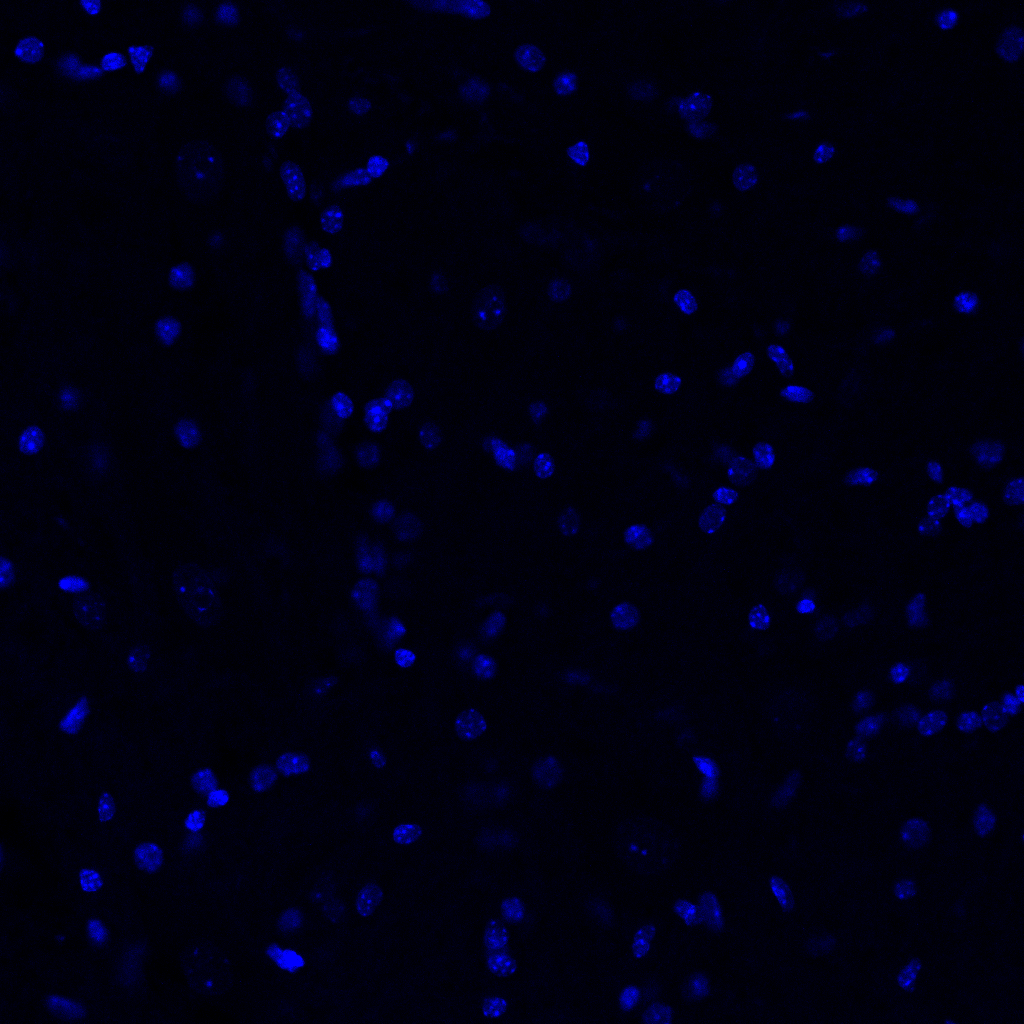

Supplement: Supplementary file 11 — Source data Fig. 7 [file 44321_2025_323_MOESM11_ESM.zip › Figure 7/7N/WT PUB 40X 1-Image Export-37_c1 (4).tif]

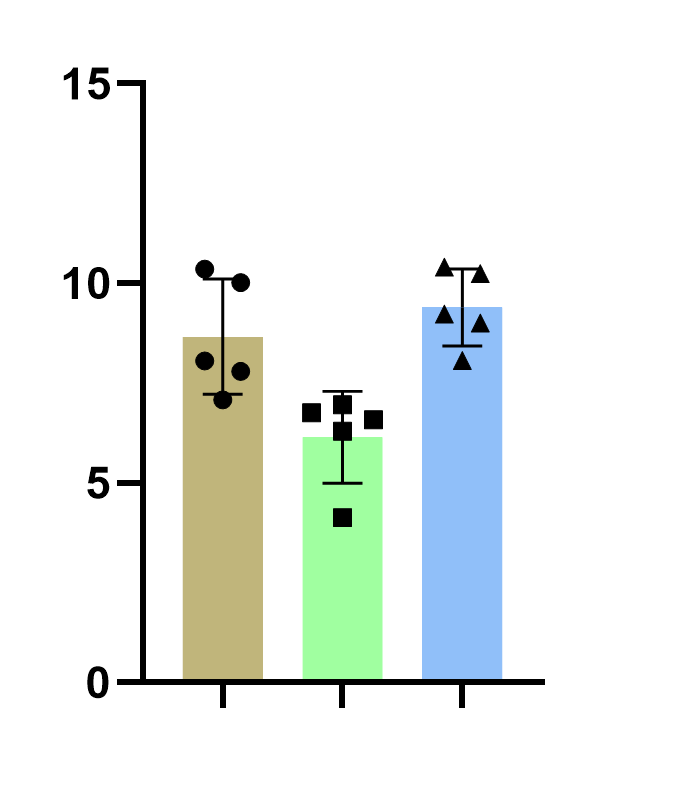

Supplement: Supplementary file 11 — Source data Fig. 7 [file 44321_2025_323_MOESM11_ESM.zip › Figure 7/7O/PUB.tif]

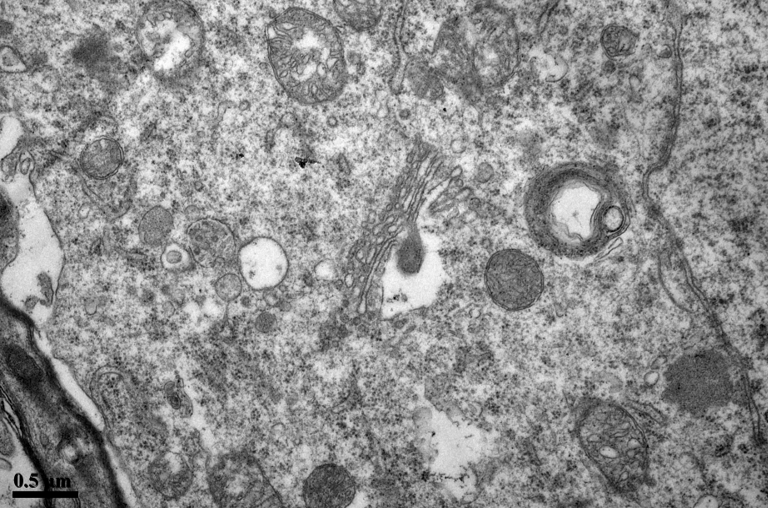

Supplement: Supplementary file 11 — Source data Fig. 7 [file 44321_2025_323_MOESM11_ESM.zip › Figure 7/7P/Nano-ISO.tif]

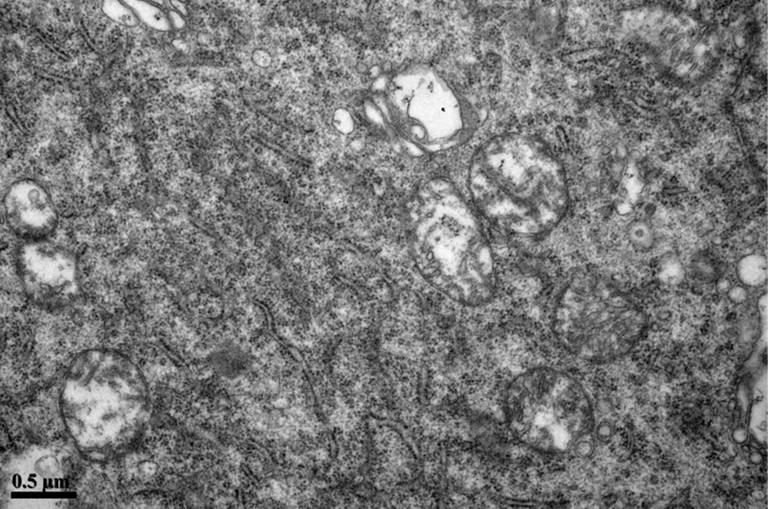

Supplement: Supplementary file 11 — Source data Fig. 7 [file 44321_2025_323_MOESM11_ESM.zip › Figure 7/7P/Veh.tif]

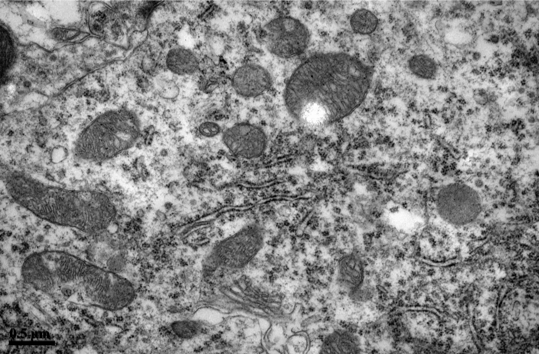

Supplement: Supplementary file 11 — Source data Fig. 7 [file 44321_2025_323_MOESM11_ESM.zip › Figure 7/7P/Wild Type.tif]

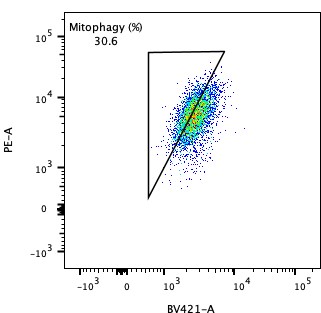

Supplement: Supplementary file 12 — Figure EV1 Source Data [file 44321_2025_323_MOESM12_ESM.zip › Figure EV1/EV1C/CCCP.jpg]

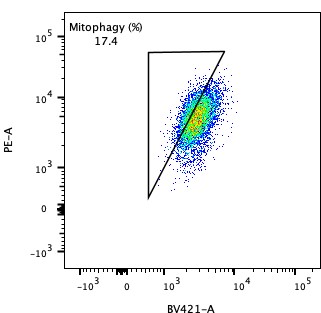

Supplement: Supplementary file 12 — Figure EV1 Source Data [file 44321_2025_323_MOESM12_ESM.zip › Figure EV1/EV1C/ISO.jpg]

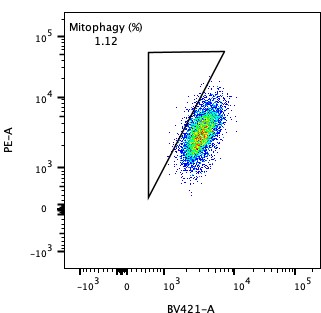

Supplement: Supplementary file 12 — Figure EV1 Source Data [file 44321_2025_323_MOESM12_ESM.zip › Figure EV1/EV1C/VEH.jpg]

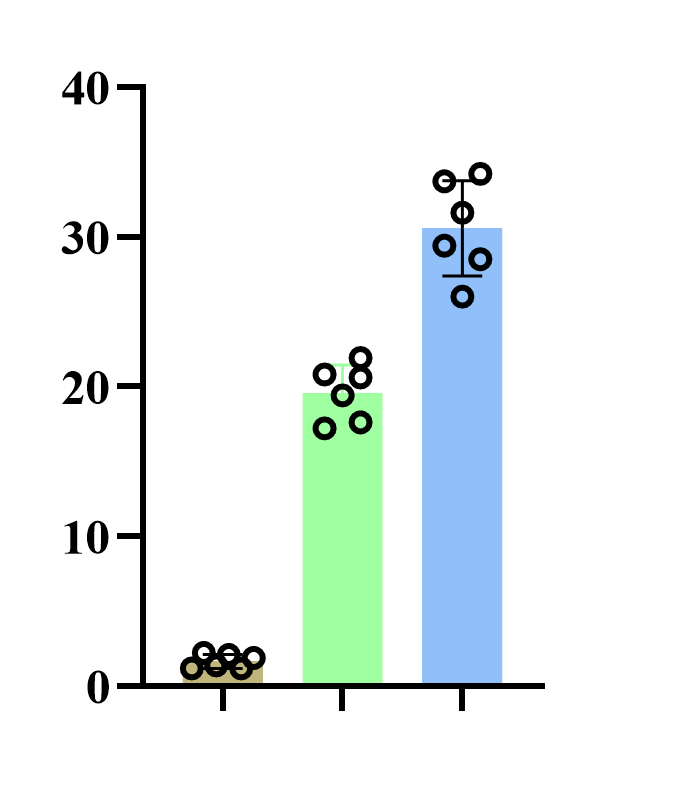

Supplement: Supplementary file 12 — Figure EV1 Source Data [file 44321_2025_323_MOESM12_ESM.zip › Figure EV1/EV1D/MKEIMA.tif]

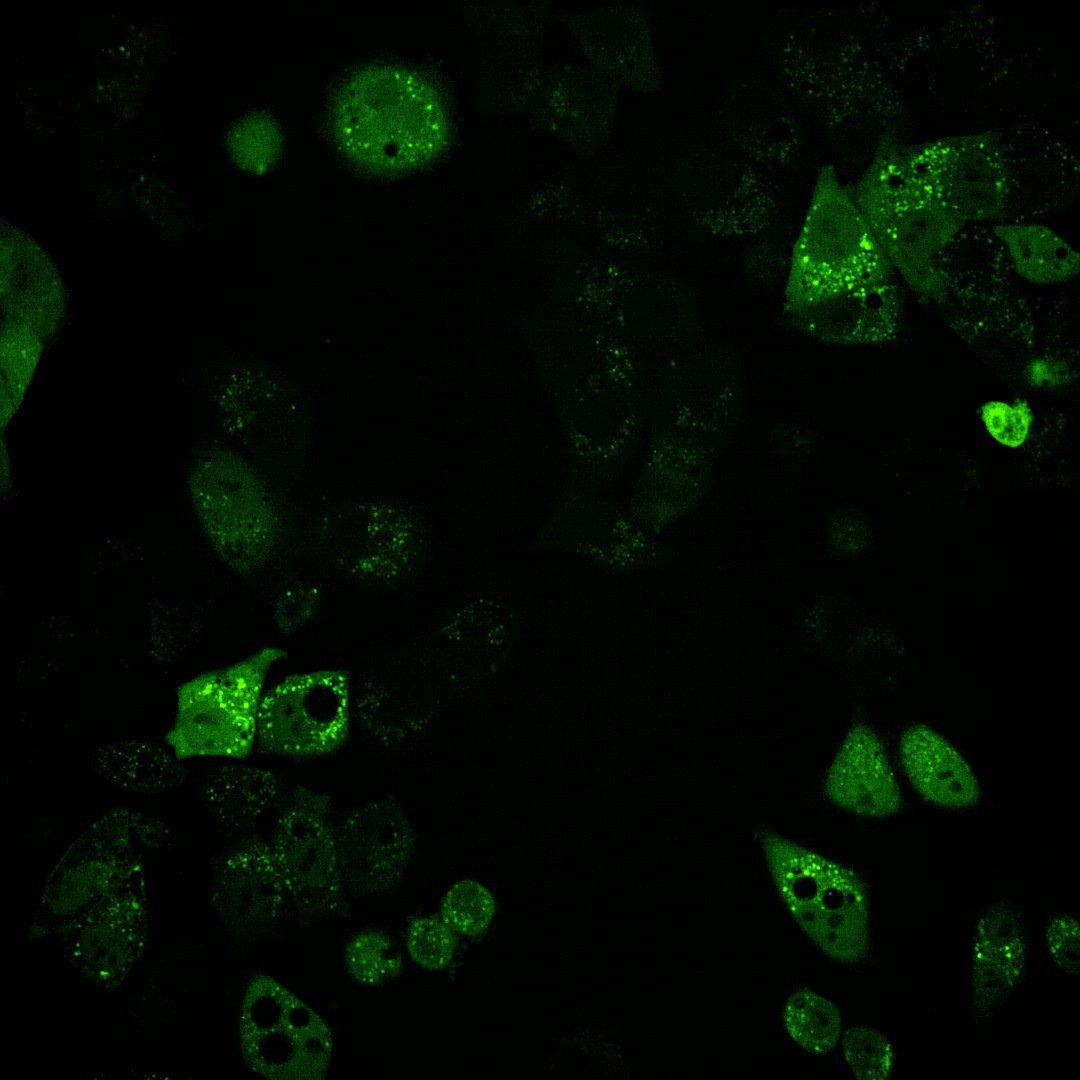

Supplement: Supplementary file 12 — Figure EV1 Source Data [file 44321_2025_323_MOESM12_ESM.zip › Figure EV1/EV1E/CCCP.tif]

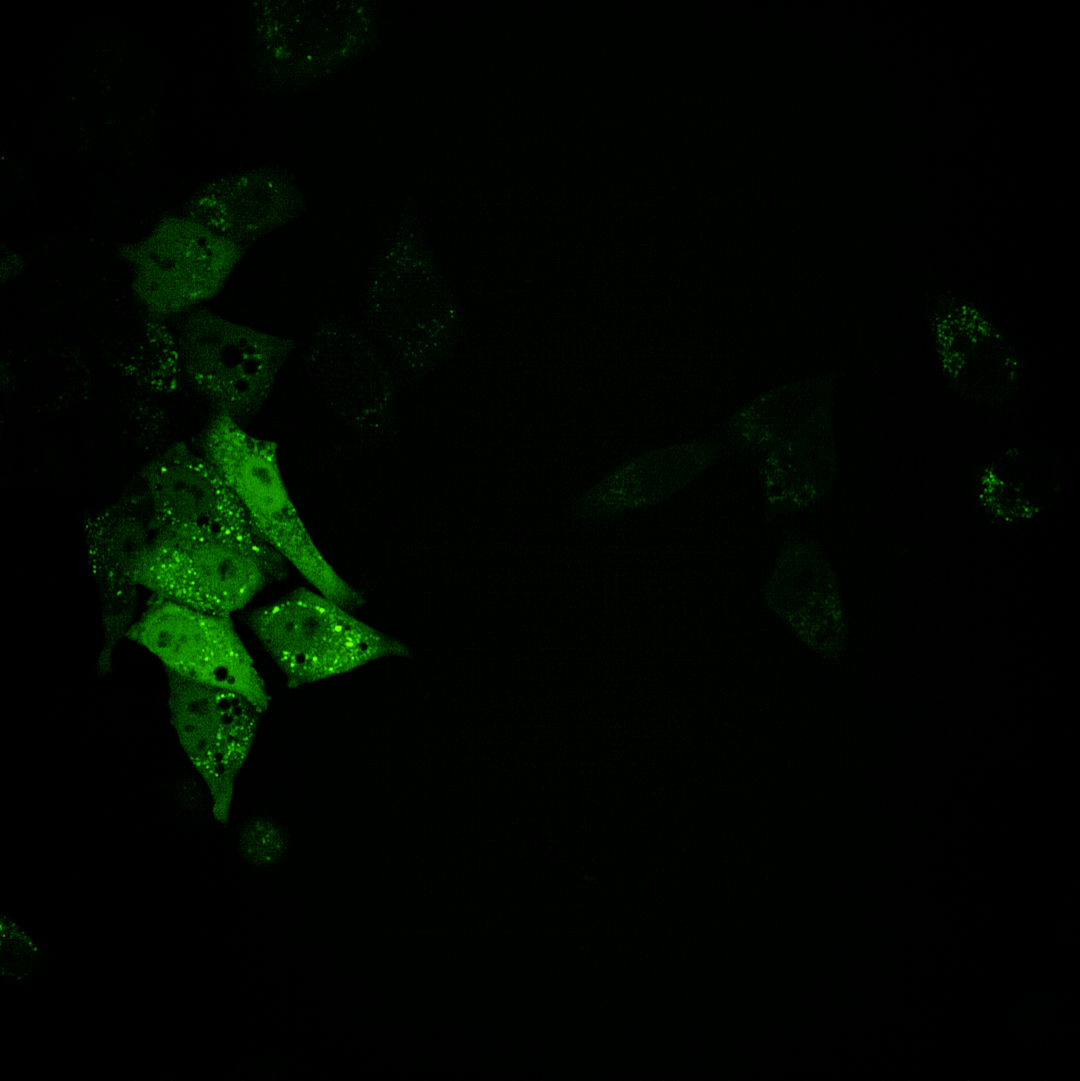

Supplement: Supplementary file 12 — Figure EV1 Source Data [file 44321_2025_323_MOESM12_ESM.zip › Figure EV1/EV1E/ISO.tif]

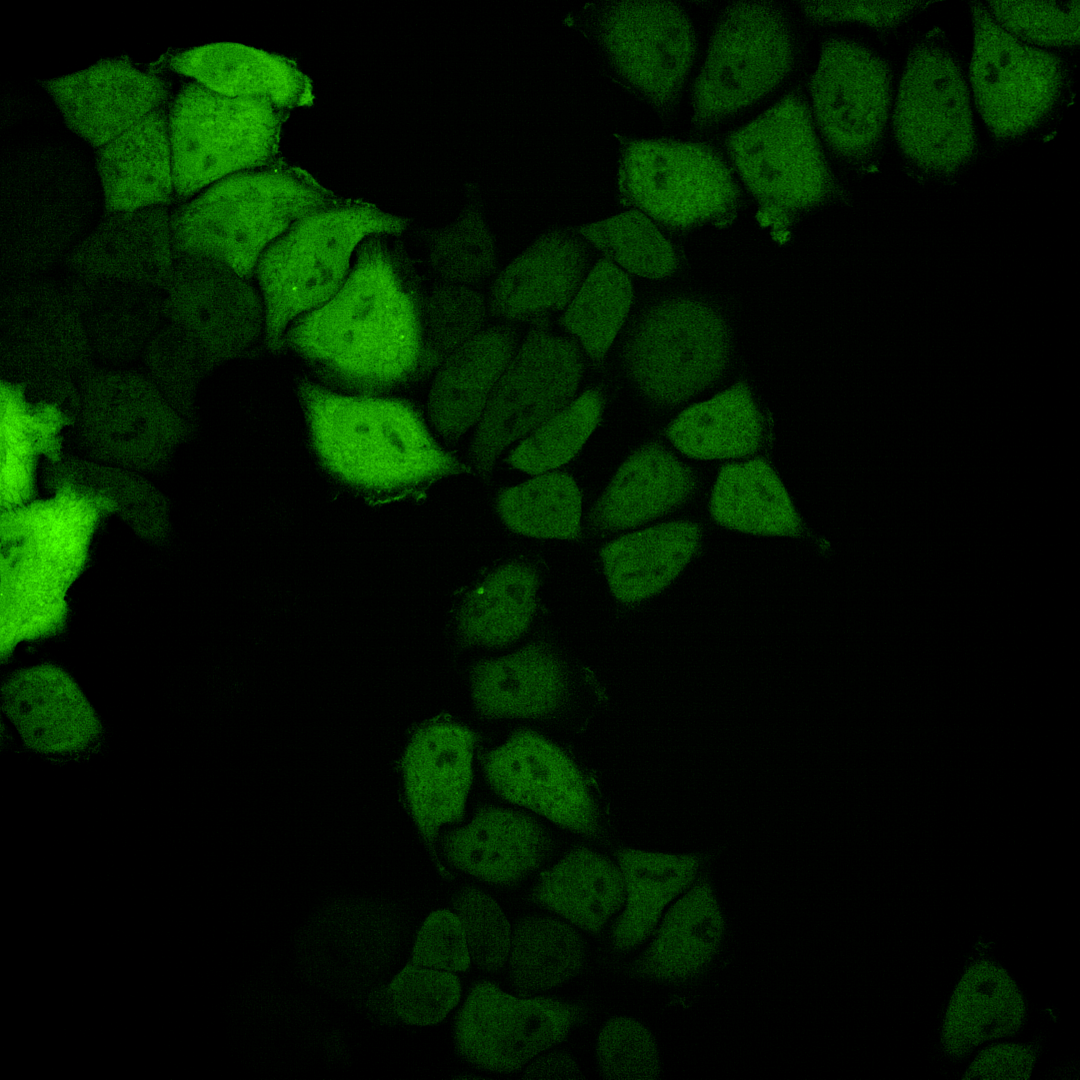

Supplement: Supplementary file 12 — Figure EV1 Source Data [file 44321_2025_323_MOESM12_ESM.zip › Figure EV1/EV1E/Veh.tif]

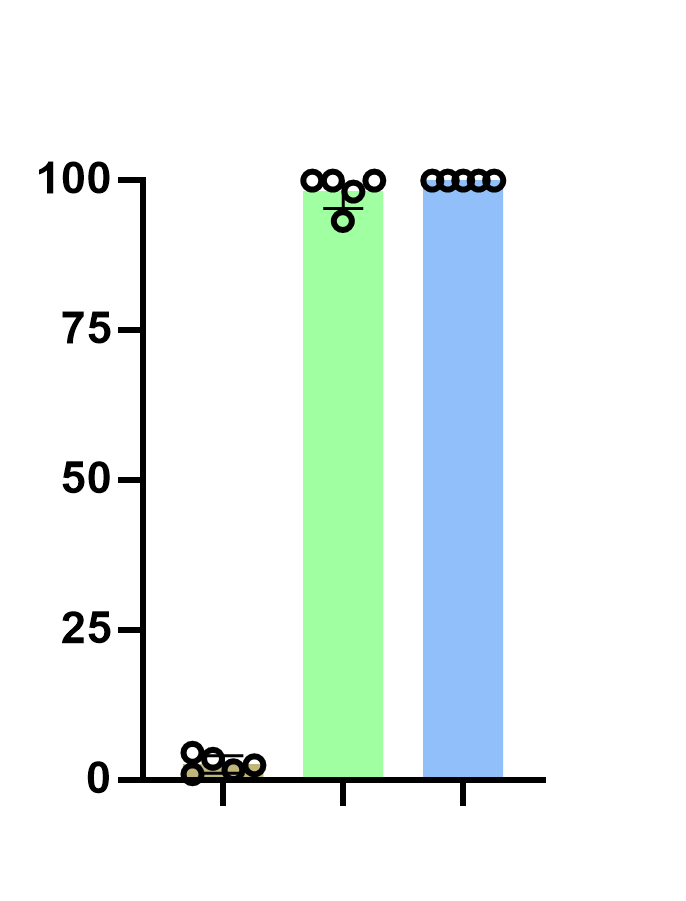

Supplement: Supplementary file 12 — Figure EV1 Source Data [file 44321_2025_323_MOESM12_ESM.zip › Figure EV1/EV1F/PARKIN.tif]

## Slide 1
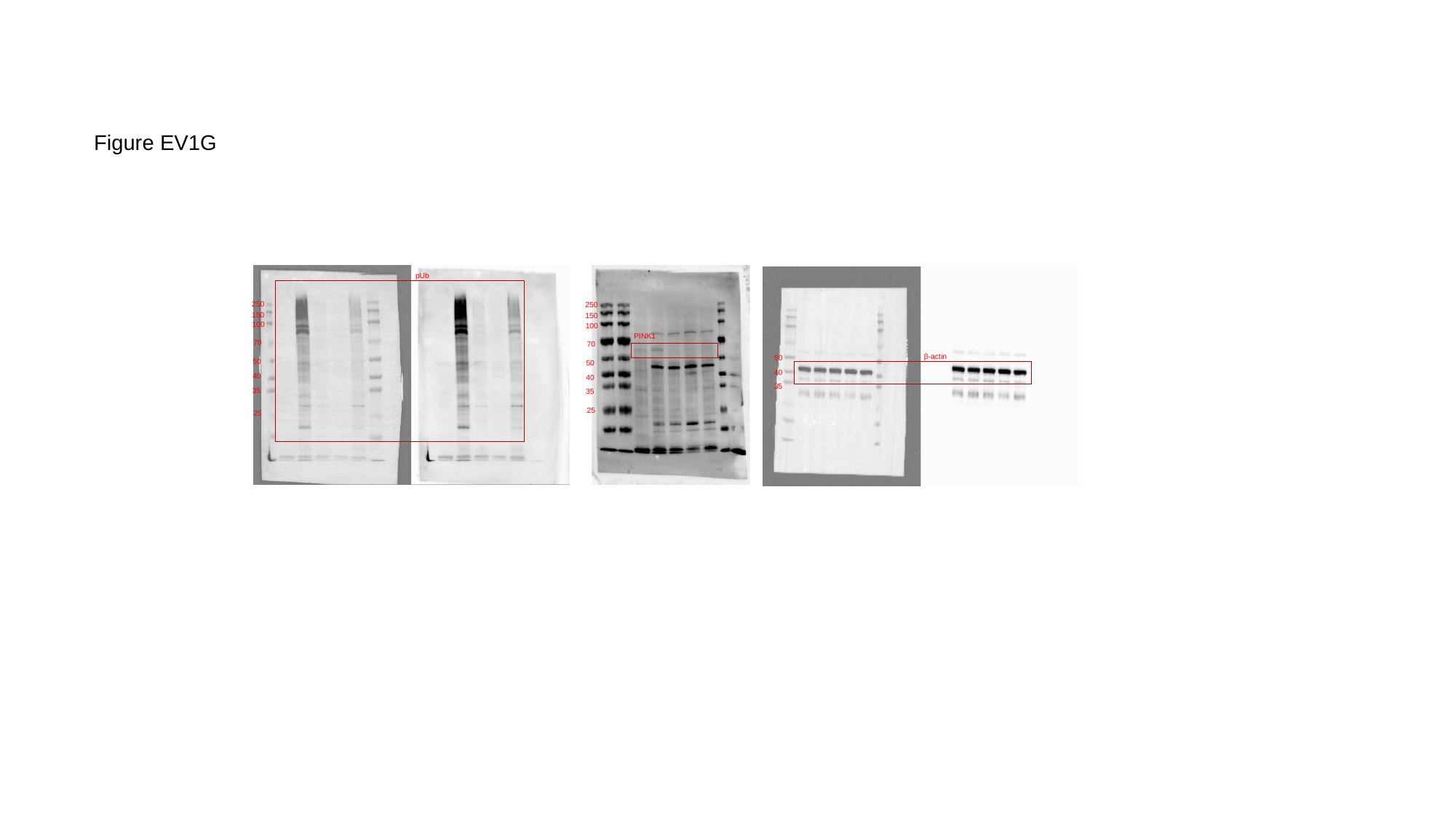

Figure EV1G
pUb
250
250
150
150
100
100
PINK1
70
70
β-actin
50
50
50
40
40
40
35
35
35
25
25

## Slide 2
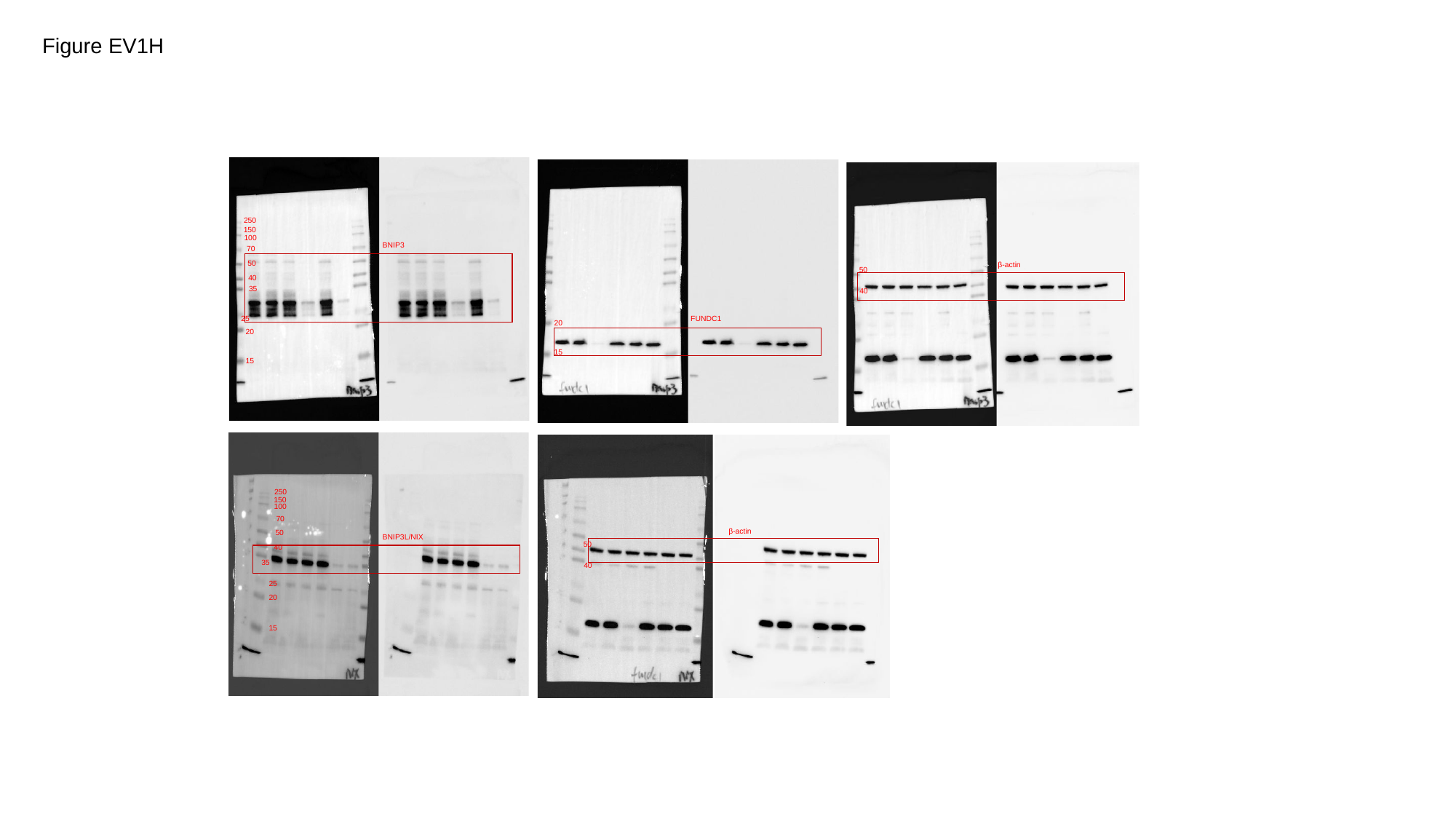

Figure EV1H
250
150
100
BNIP3
70
50
40
35
25
20
15
β-actin
50
40
FUNDC1
20
15
250
150
100
70
50
BNIP3L/NIX
40
35
25
20
15
β-actin
50
40

Supplement: Supplementary file 12 — Figure EV1 Source Data [file 44321_2025_323_MOESM12_ESM.zip › Figure EV1/EV1G-H/WB.pptx]

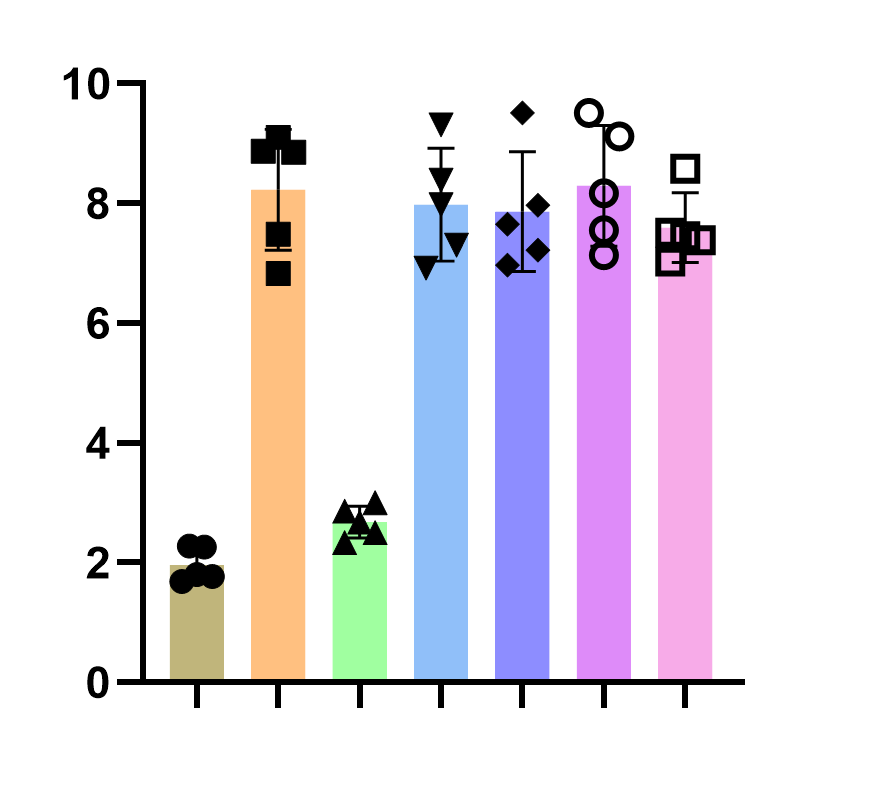

Supplement: Supplementary file 12 — Figure EV1 Source Data [file 44321_2025_323_MOESM12_ESM.zip › Figure EV1/EV1I/mitophagy.tif]

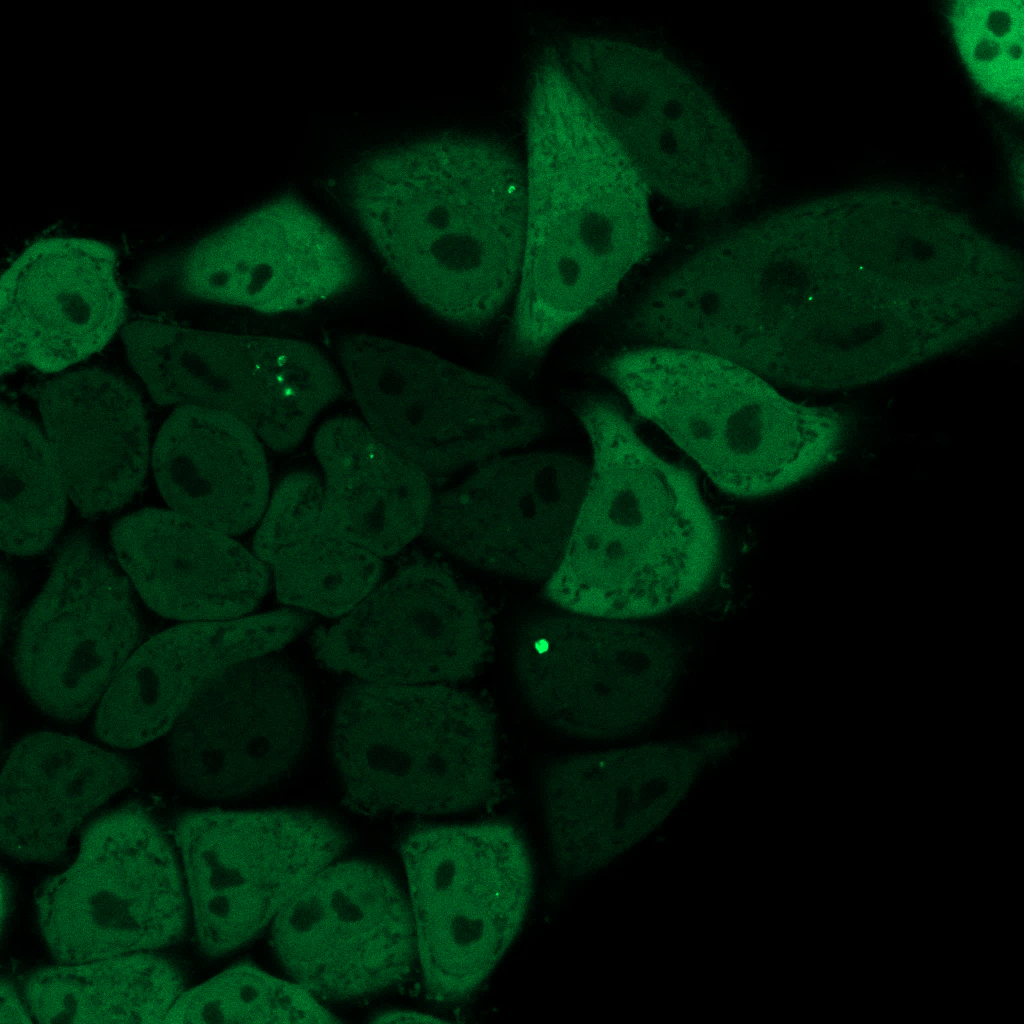

Supplement: Supplementary file 13 — Figure EV2 Source Data [file 44321_2025_323_MOESM13_ESM.zip › Figure EV2/EV2F/PRT-CCCP.tif]

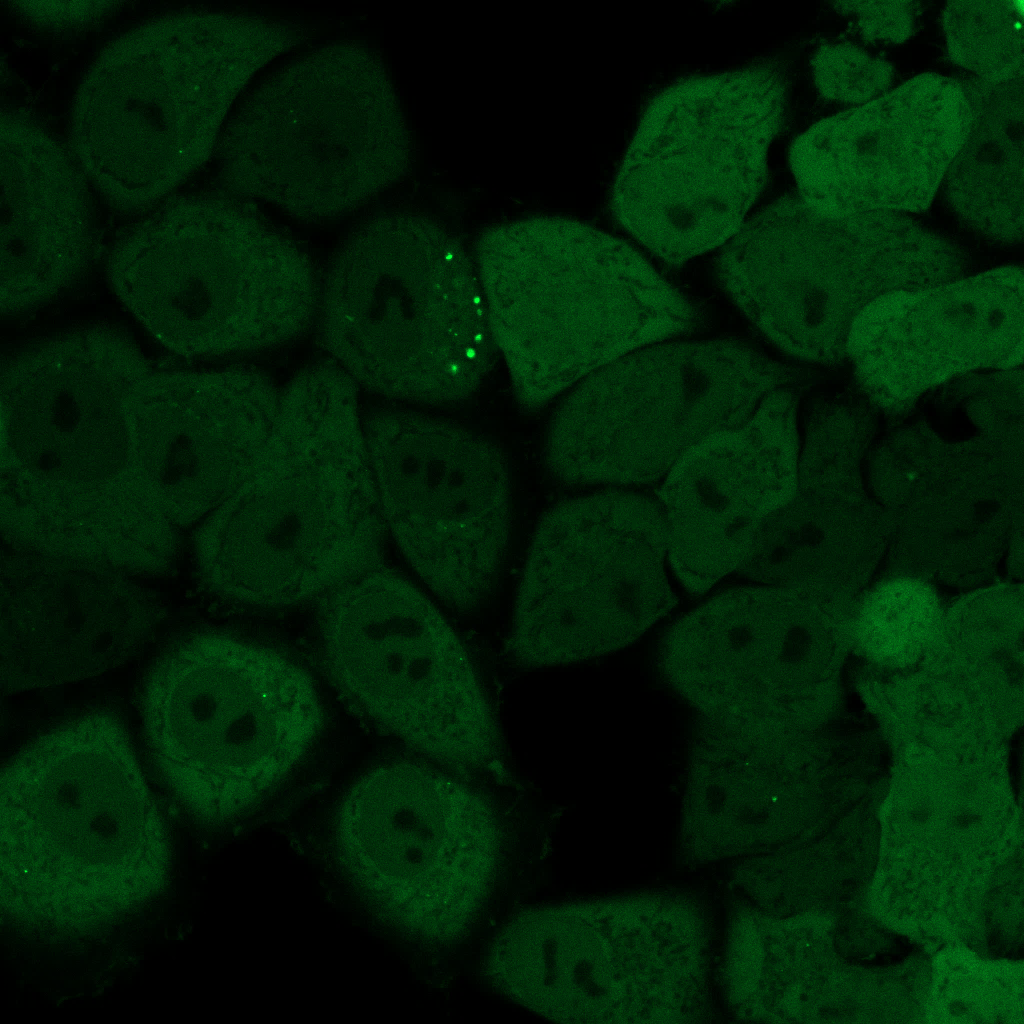

Supplement: Supplementary file 13 — Figure EV2 Source Data [file 44321_2025_323_MOESM13_ESM.zip › Figure EV2/EV2F/PRT-ISO.tif]

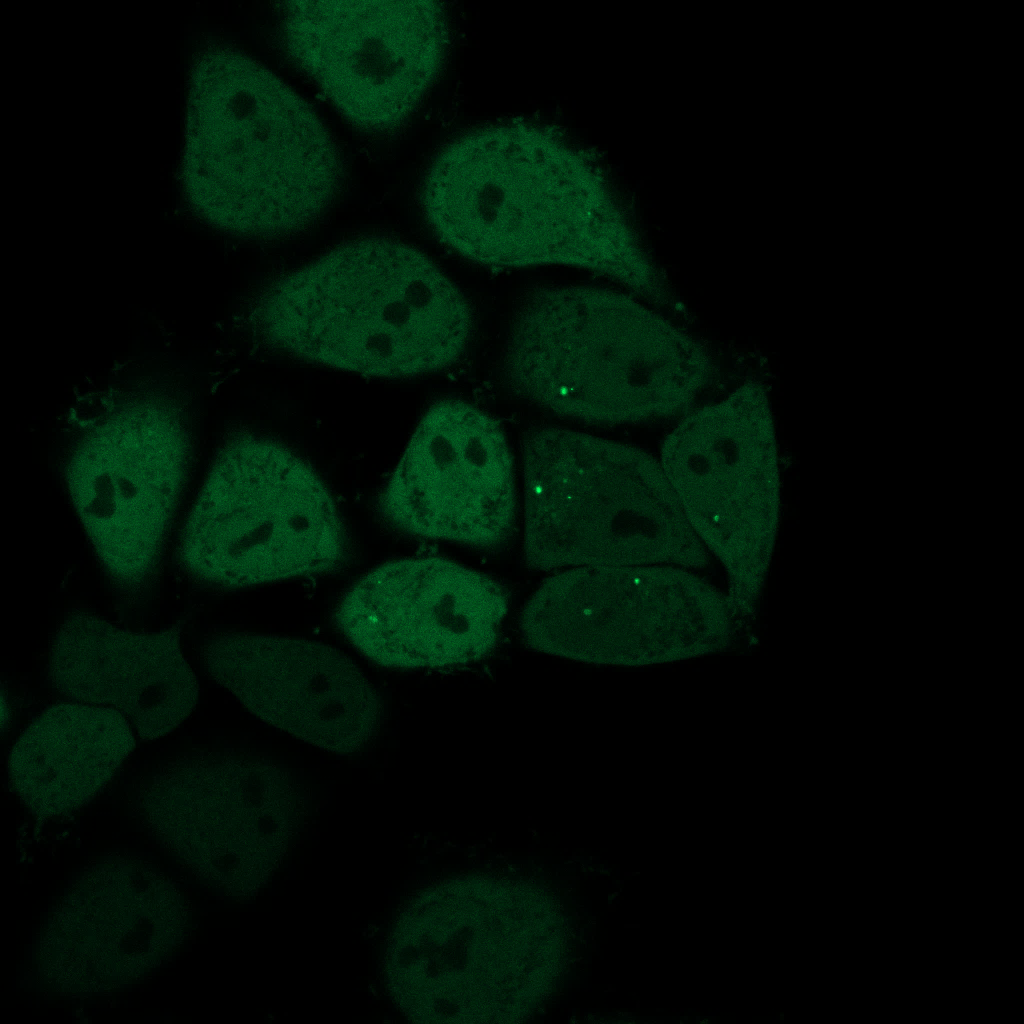

Supplement: Supplementary file 13 — Figure EV2 Source Data [file 44321_2025_323_MOESM13_ESM.zip › Figure EV2/EV2F/PRT-ISO+CCCP.tif]

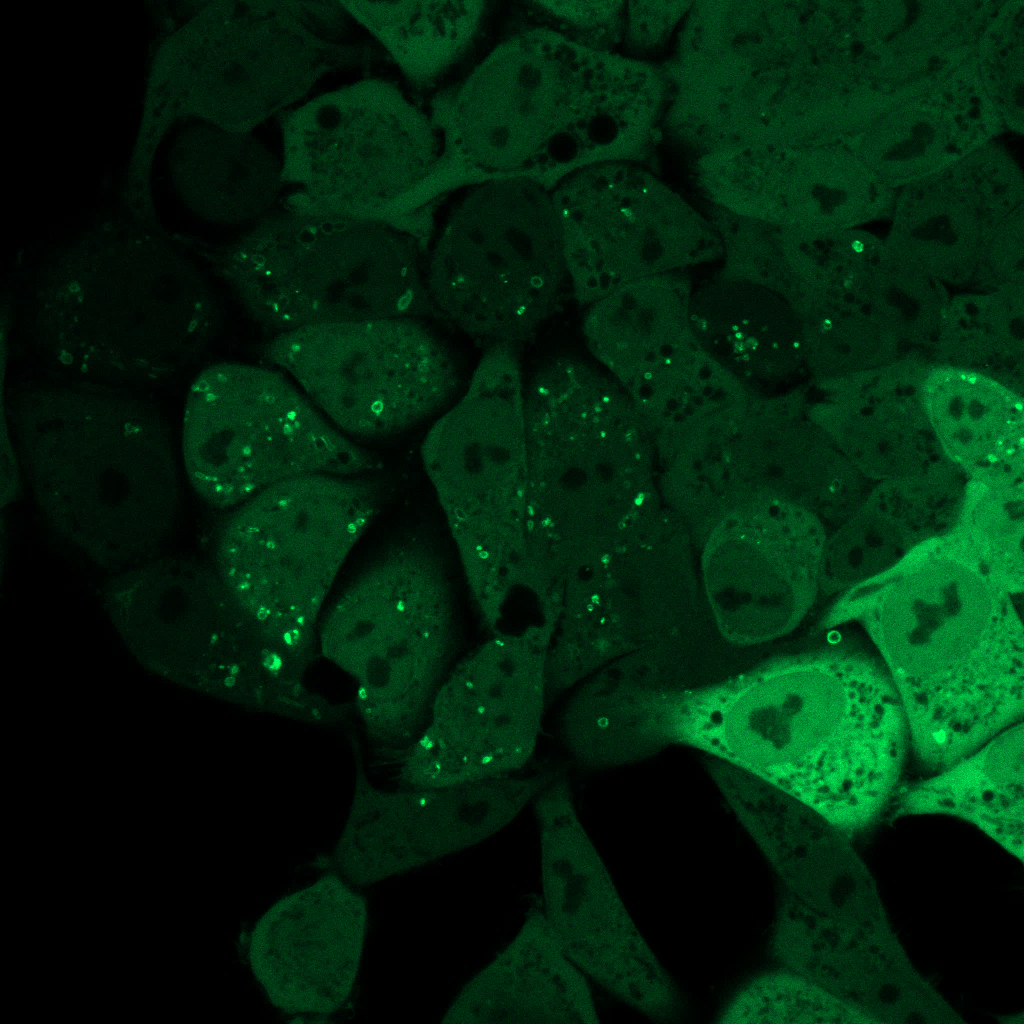

Supplement: Supplementary file 13 — Figure EV2 Source Data [file 44321_2025_323_MOESM13_ESM.zip › Figure EV2/EV2F/VEH-CCCP.tif]

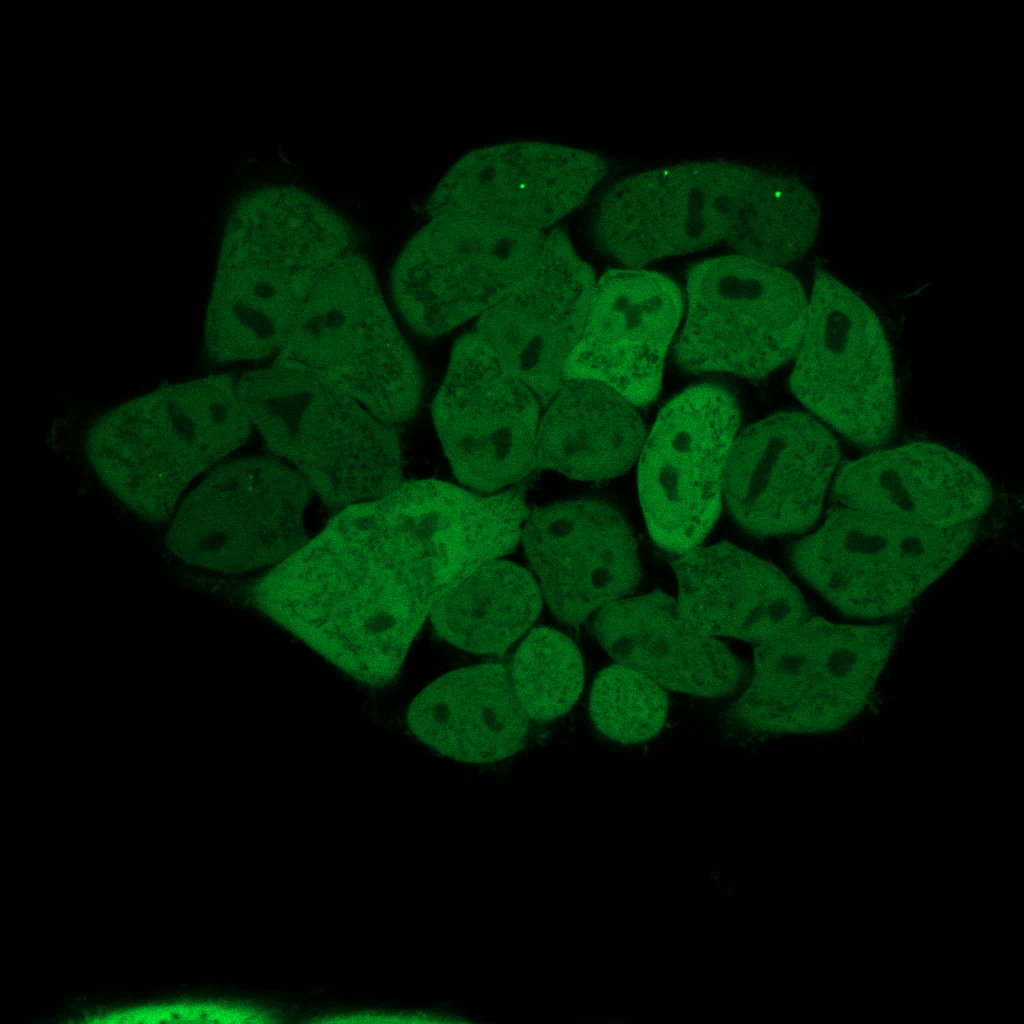

Supplement: Supplementary file 13 — Figure EV2 Source Data [file 44321_2025_323_MOESM13_ESM.zip › Figure EV2/EV2F/VEH-ISO.tif]

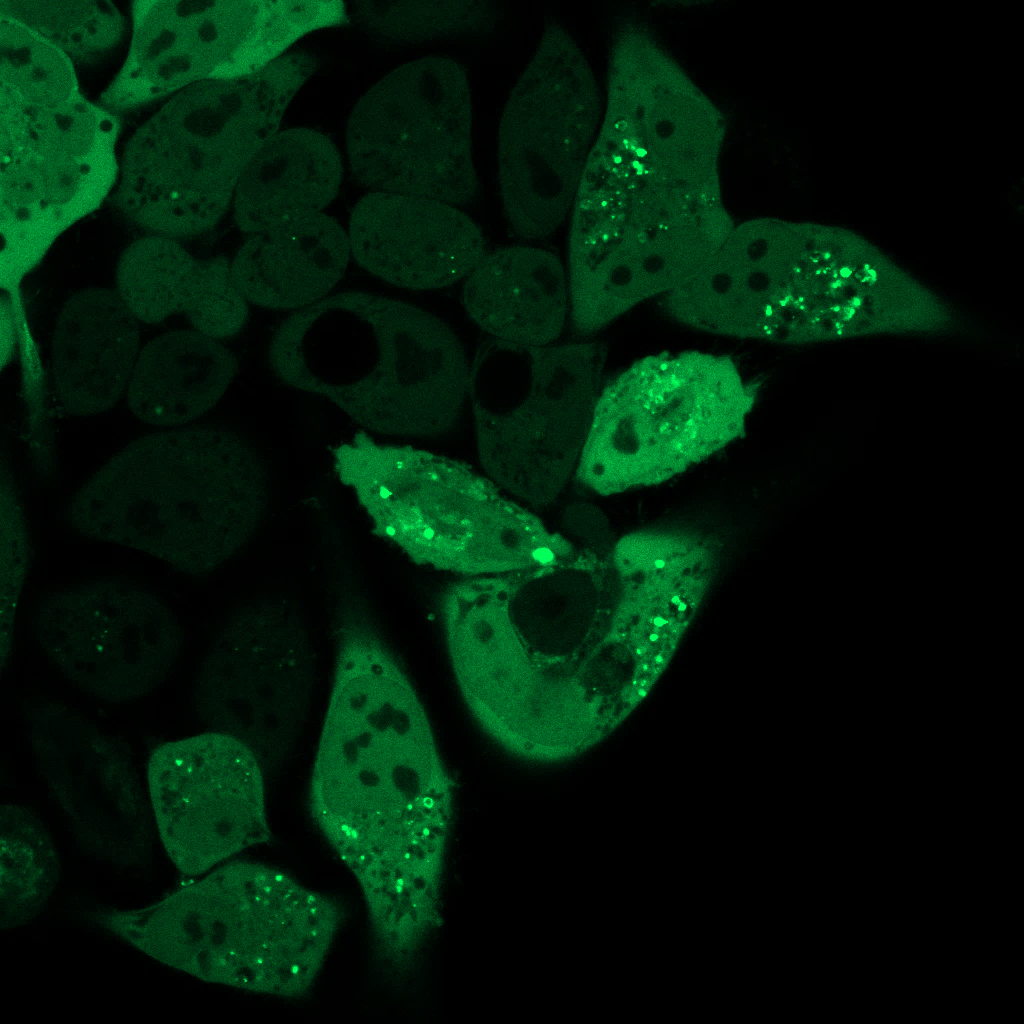

Supplement: Supplementary file 13 — Figure EV2 Source Data [file 44321_2025_323_MOESM13_ESM.zip › Figure EV2/EV2F/VEH-ISO+CCCP.tif]

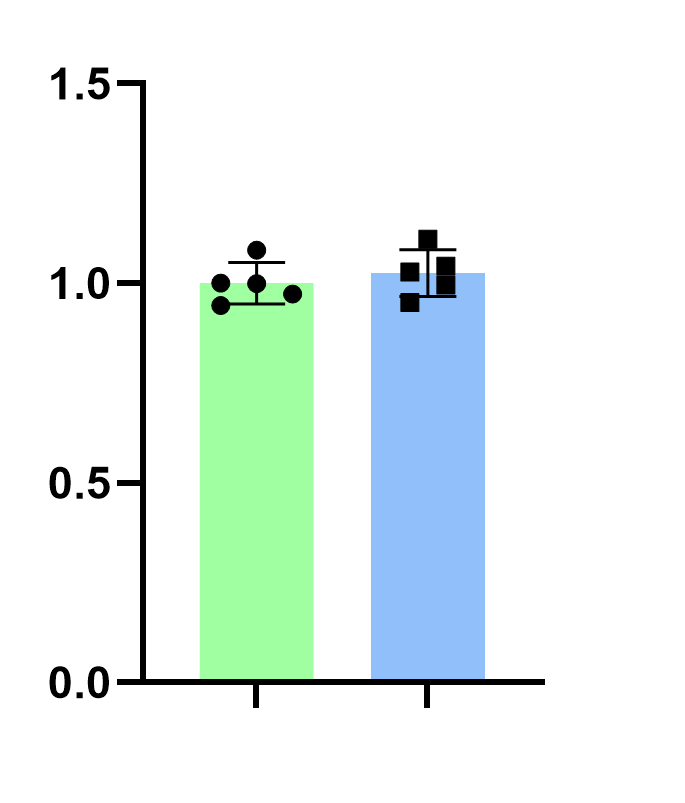

Supplement: Supplementary file 13 — Figure EV2 Source Data [file 44321_2025_323_MOESM13_ESM.zip › Figure EV2/EV2G/PINK1 mRNA.tif]

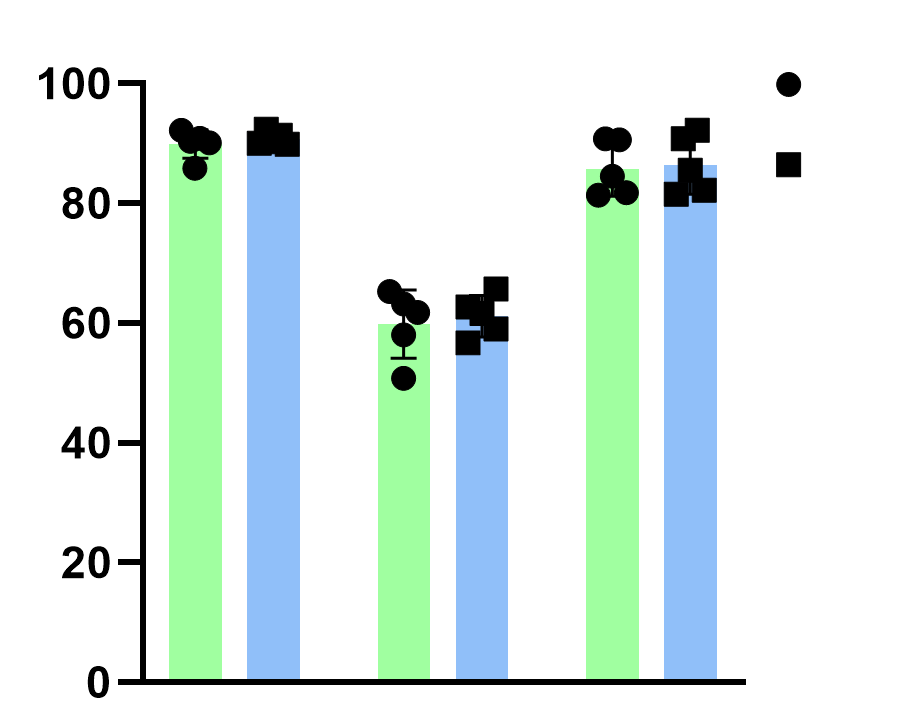

Supplement: Supplementary file 13 — Figure EV2 Source Data [file 44321_2025_323_MOESM13_ESM.zip › Figure EV2/EV2H/WASH MMP.tif]
