# Supplementary material for: Isoginkgetin antagonizes ALS pathologies in its animal and patient iPSC models via PINK1-Parkin-dependent mitophagy
Source: EMBO Mol Med. 2025 Oct 15;17(11):3139–73. doi: 10.1038/s44321-025-00323-2 (PMC12603167; doi:10.1038/s44321-025-00323-2)

1. **Blank solution (50% acetonitrile solution)**
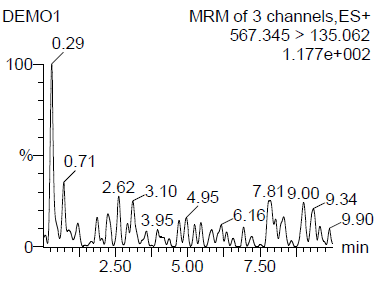

2. **Detection of standard solution (1 ng/mL Isoginkgetin)**
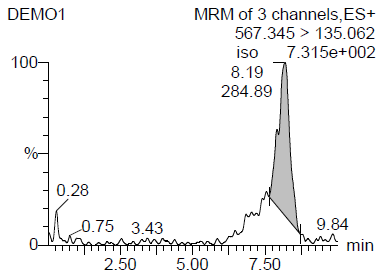

3. **Blank tissue samples (containing 1 ng/mL Isoginkgetin)**
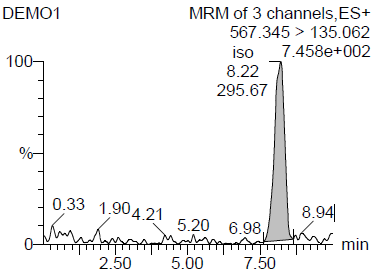

4. **Sample detection:**

**#24 h (10.8 ng/mL)**


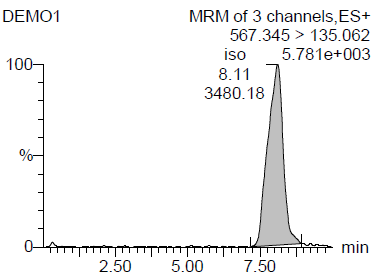


**#48 h (2.9 ng/mL)**


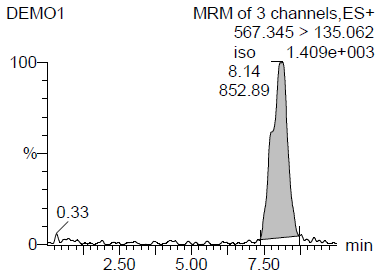


**#72 h (1.2 ng/mL)**


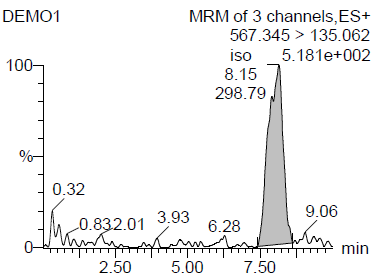


**#96 h (0.8 ng/mL)**


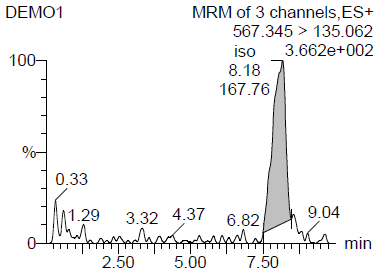


**#120 h (0.7 ng/mL)**


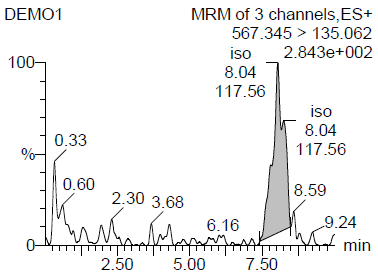


**#Nano-ISO (After being diluted approximately 1000 times and injected, a significant response can be detected)**


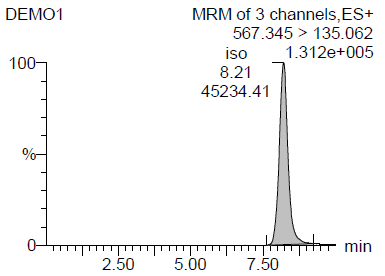

Supplement: Supplementary file 11 — Source data Fig. 7 [file 44321_2025_323_MOESM11_ESM.zip › Figure 7/7C/LC-MS-MS ISO BBB.docx]
