# Supplementary material for: Isoginkgetin antagonizes ALS pathologies in its animal and patient iPSC models via PINK1-Parkin-dependent mitophagy
Source: EMBO Mol Med. 2025 Oct 15;17(11):3139–73. doi: 10.1038/s44321-025-00323-2 (PMC12603167; doi:10.1038/s44321-025-00323-2)
Supplement: Supplementary file 12 — Figure EV1 Source Data [file 44321_2025_323_MOESM12_ESM.zip › Figure EV1/EV1A-B/Drug screen result.pptx]

## Slide 1
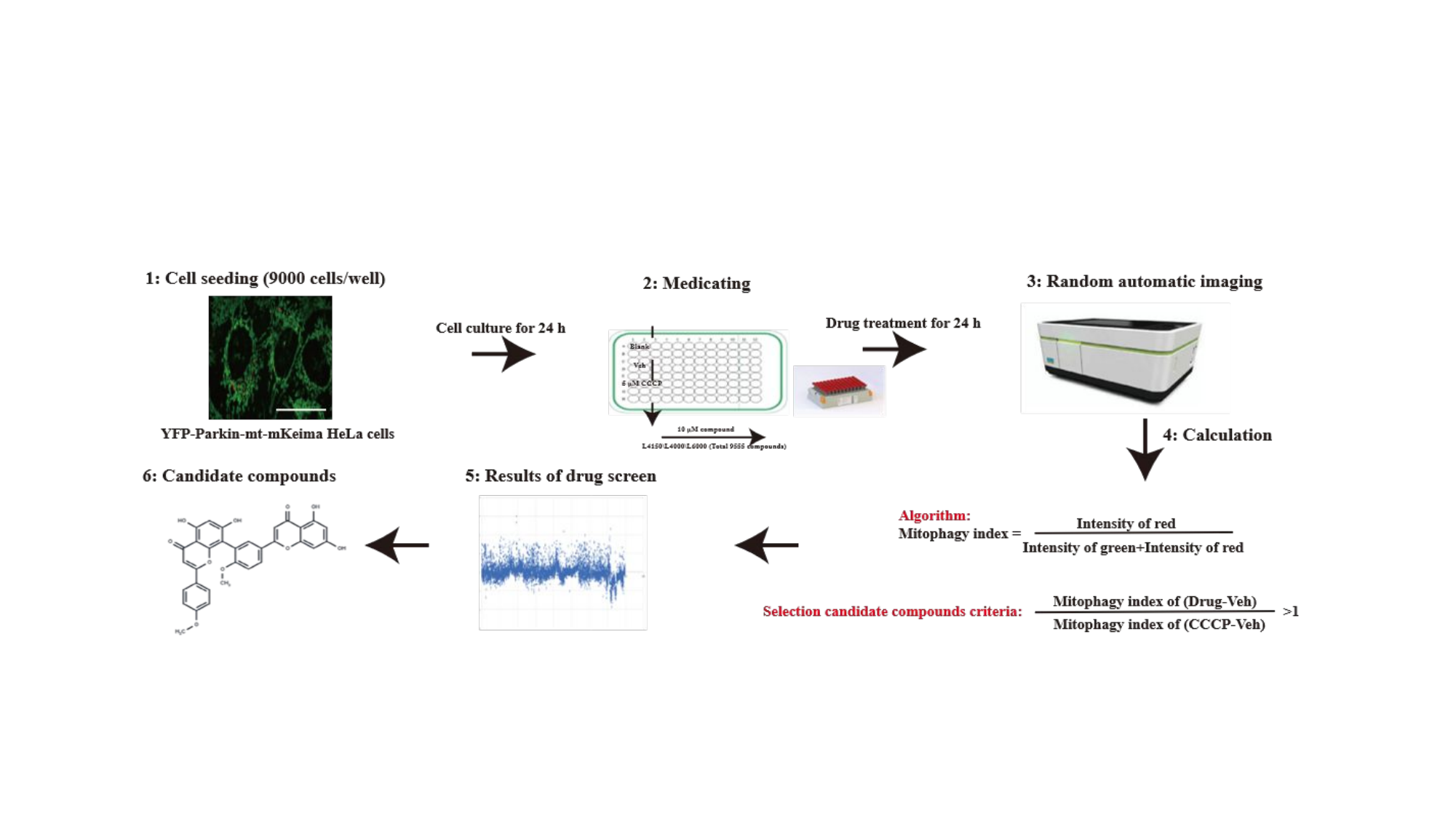

## Slide 2
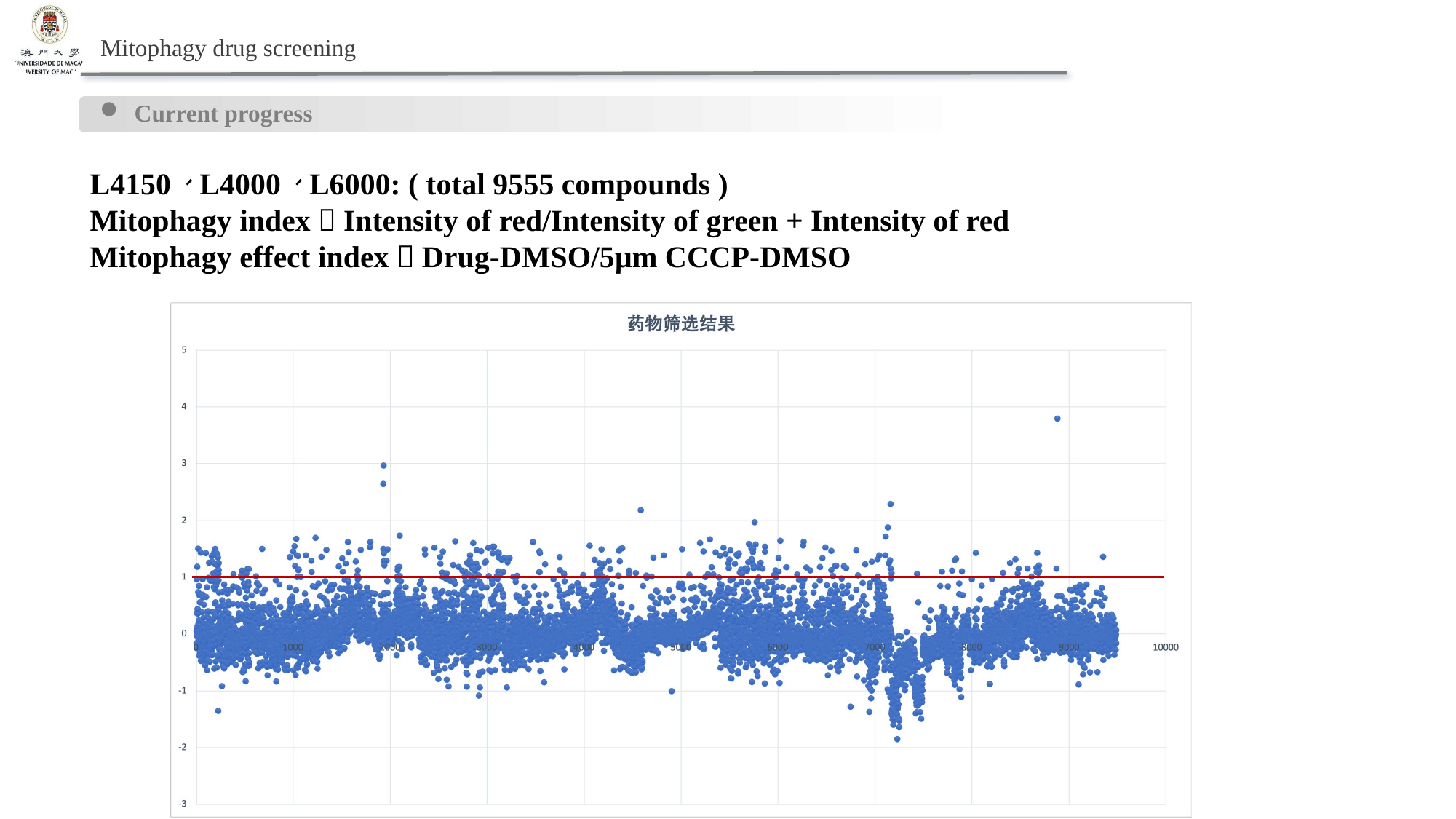

Mitophagy drug screening
Current progress
L4150、L4000、L6000: ( total 9555 compounds )
Mitophagy index：Intensity of red/Intensity of green + Intensity of red
Mitophagy effect index：Drug-DMSO/5μm CCCP-DMSO

## Slide 3
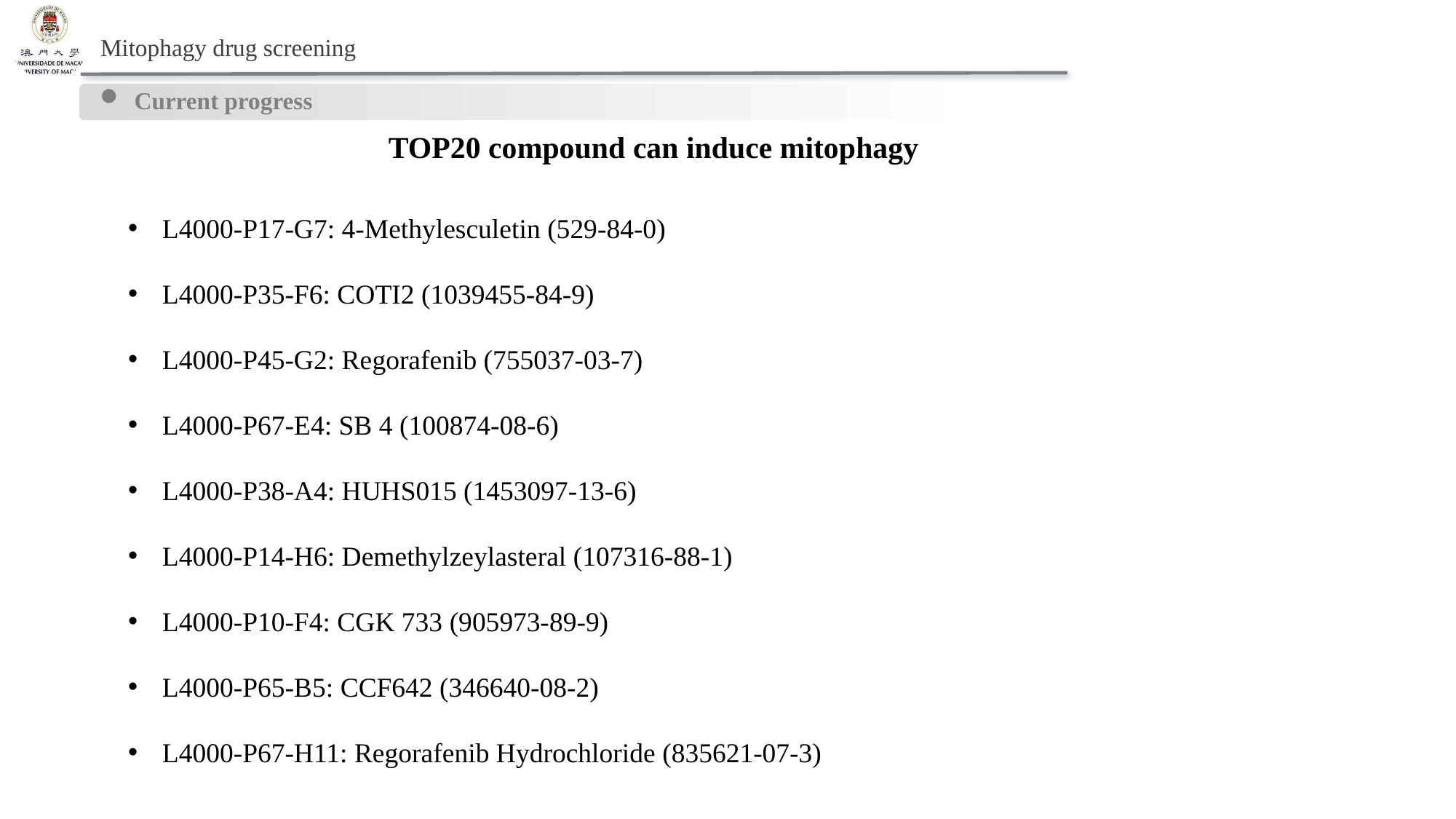

Mitophagy drug screening
Current progress
TOP20 compound can induce mitophagy
L4000-P17-G7: 4-Methylesculetin (529-84-0)
L4000-P35-F6: COTI2 (1039455-84-9)
L4000-P45-G2: Regorafenib (755037-03-7)
L4000-P67-E4: SB 4 (100874-08-6)
L4000-P38-A4: HUHS015 (1453097-13-6)
L4000-P14-H6: Demethylzeylasteral (107316-88-1)
L4000-P10-F4: CGK 733 (905973-89-9)
L4000-P65-B5: CCF642 (346640-08-2)
L4000-P67-H11: Regorafenib Hydrochloride (835621-07-3)

## Slide 4
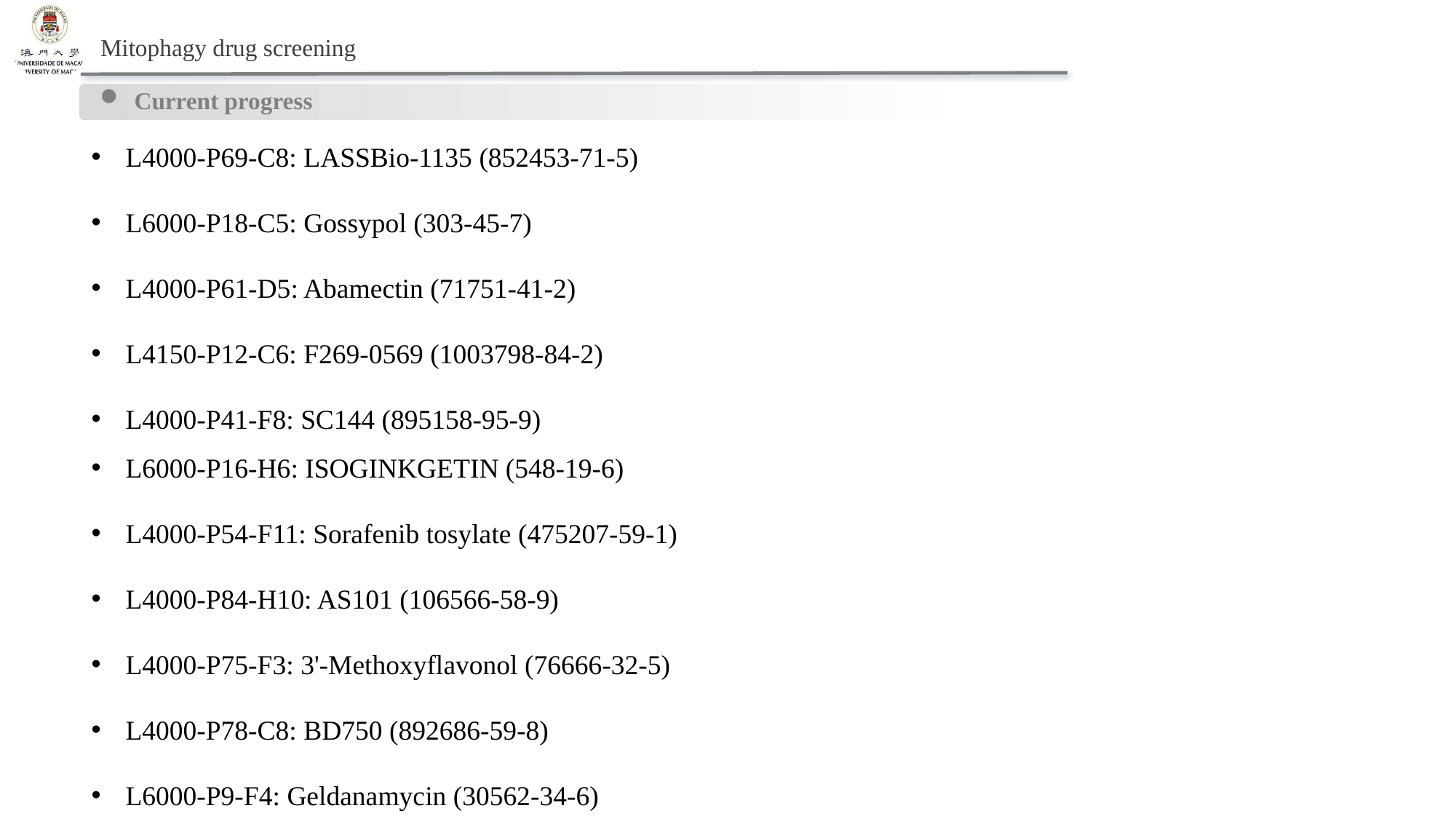

Mitophagy drug screening
Current progress
L4000-P69-C8: LASSBio-1135 (852453-71-5)
L6000-P18-C5: Gossypol (303-45-7)
L4000-P61-D5: Abamectin (71751-41-2)
L4150-P12-C6: F269-0569 (1003798-84-2)
L4000-P41-F8: SC144 (895158-95-9)
L6000-P16-H6: ISOGINKGETIN (548-19-6)
L4000-P54-F11: Sorafenib tosylate (475207-59-1)
L4000-P84-H10: AS101 (106566-58-9)
L4000-P75-F3: 3'-Methoxyflavonol (76666-32-5)
L4000-P78-C8: BD750 (892686-59-8)
L6000-P9-F4: Geldanamycin (30562-34-6)

## Slide 5
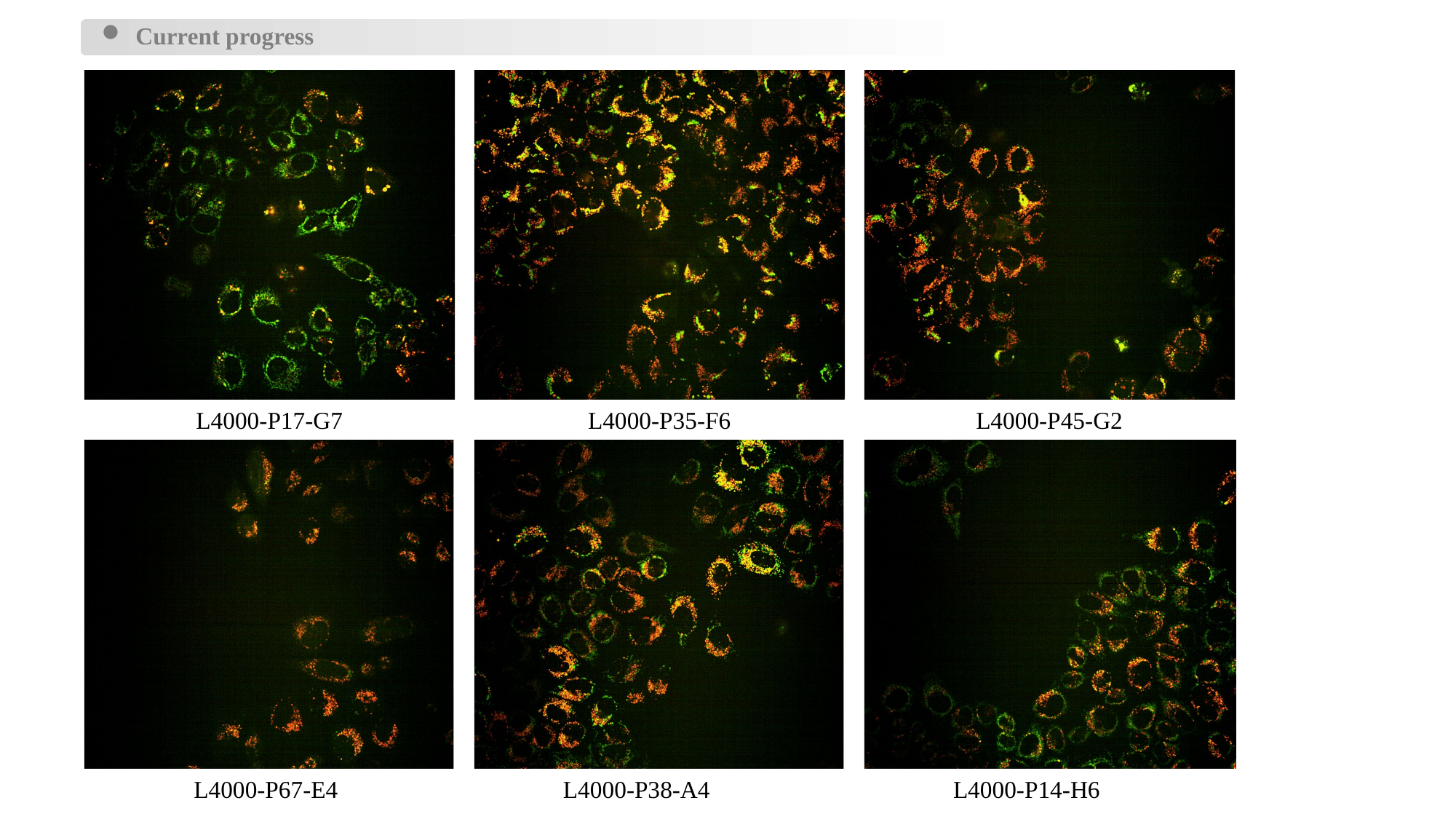

Current progress
L4000-P17-G7
L4000-P35-F6
L4000-P45-G2
L4000-P67-E4
L4000-P38-A4
L4000-P14-H6

## Slide 6
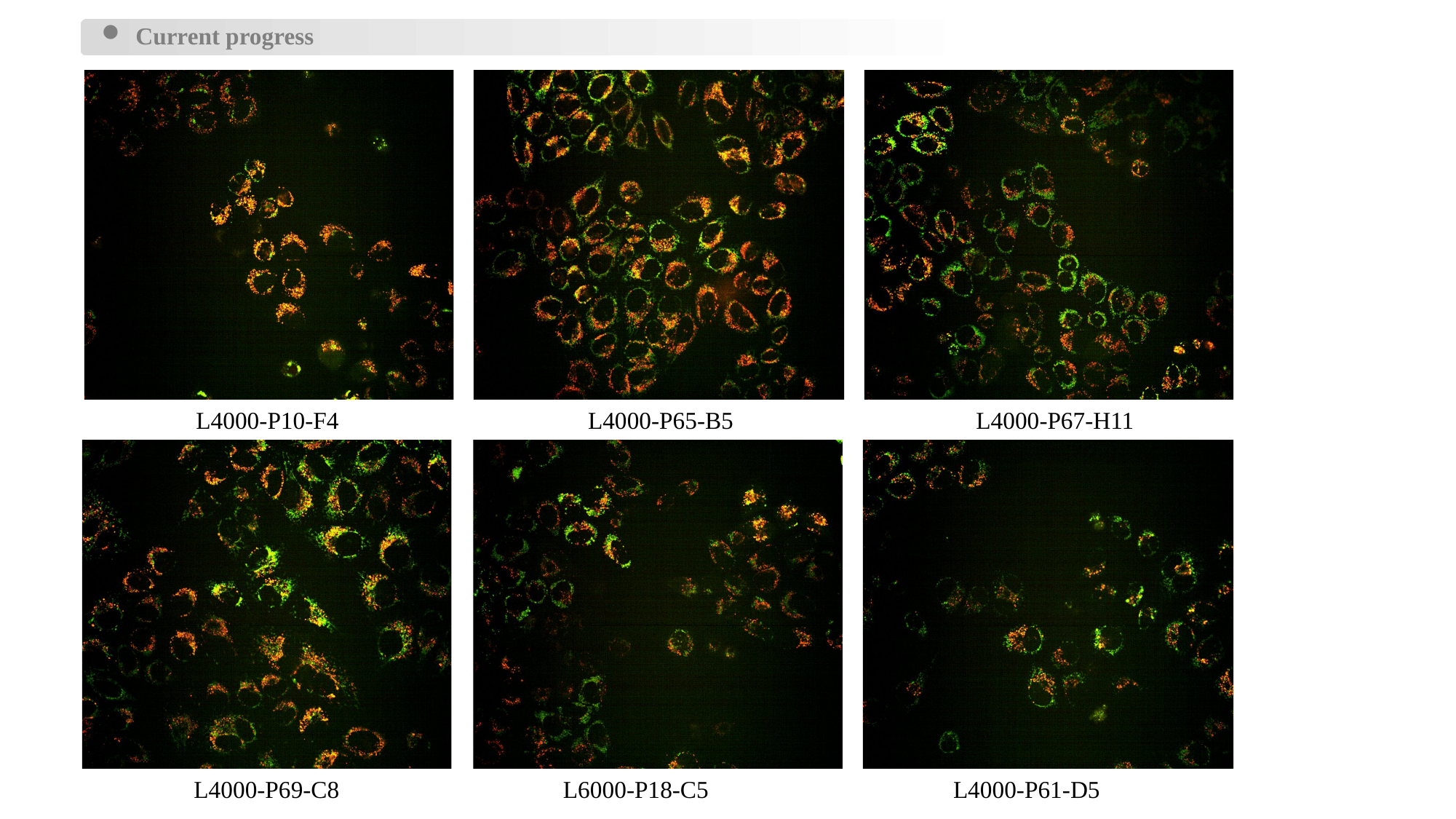

Current progress
L4000-P10-F4
L4000-P65-B5
L4000-P67-H11
L4000-P69-C8
L6000-P18-C5
L4000-P61-D5

## Slide 7
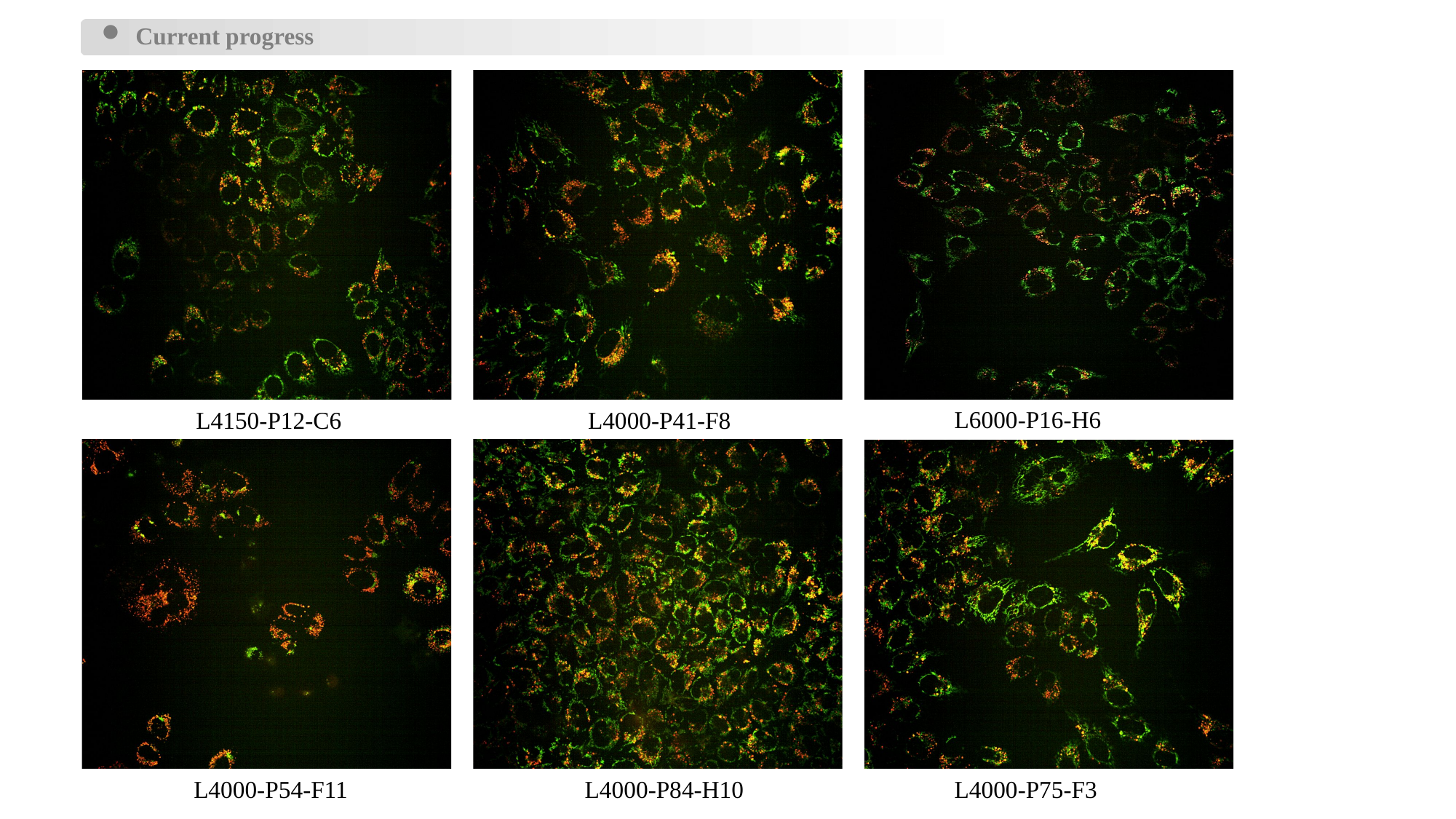

Current progress
L6000-P16-H6
L4150-P12-C6
L4000-P41-F8
L4000-P54-F11
L4000-P84-H10
L4000-P75-F3

## Slide 8
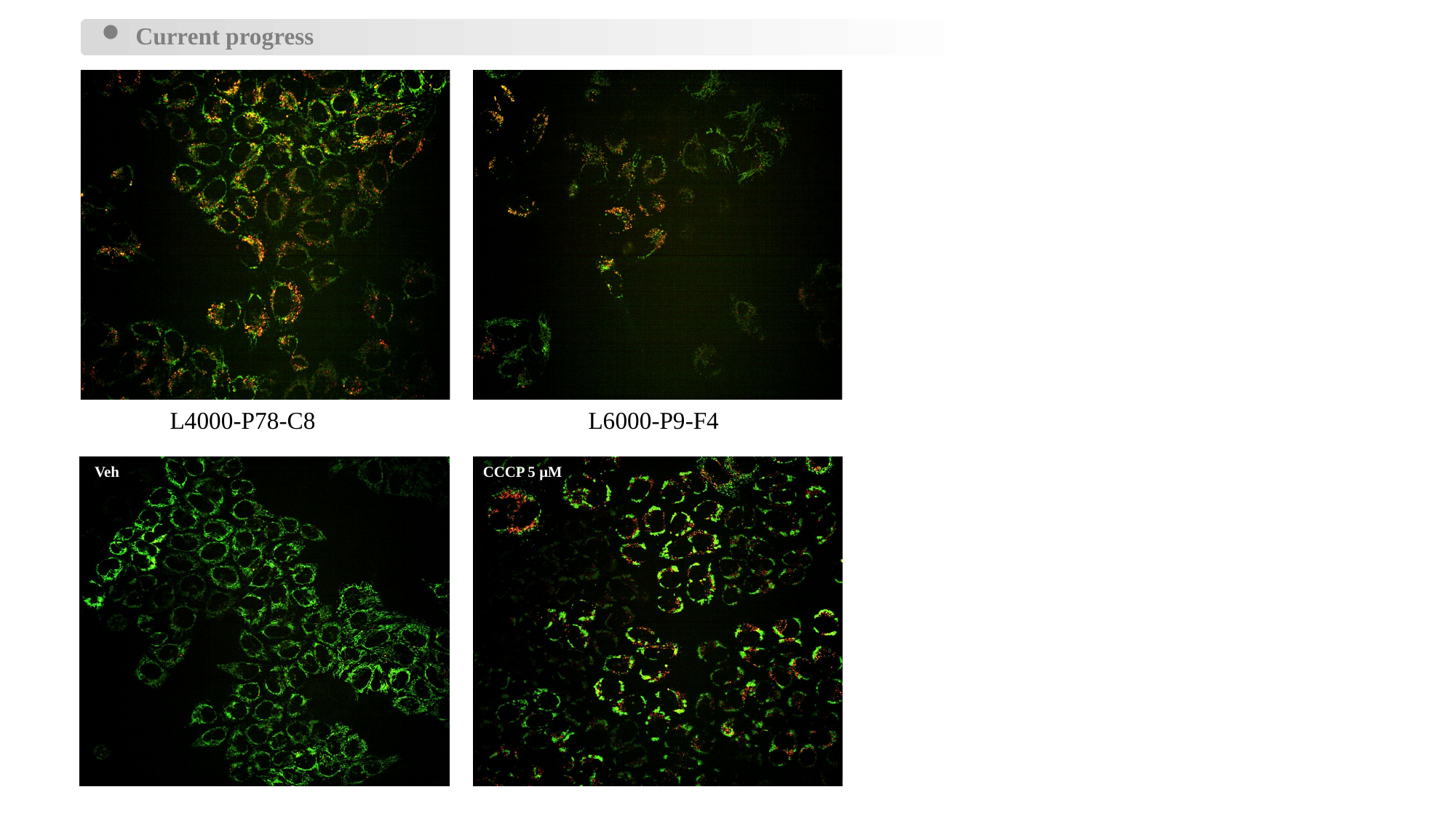

Current progress
L4000-P78-C8
L6000-P9-F4
Veh
CCCP 5 μM
