# Supplementary material for: Isoginkgetin antagonizes ALS pathologies in its animal and patient iPSC models via PINK1-Parkin-dependent mitophagy
Source: EMBO Mol Med. 2025 Oct 15;17(11):3139–73. doi: 10.1038/s44321-025-00323-2 (PMC12603167; doi:10.1038/s44321-025-00323-2)
Supplement: Supplementary file 13 — Figure EV2 Source Data [file 44321_2025_323_MOESM13_ESM.zip › Figure EV2/EV2A-B-C-D-E-I-J/WB.pptx]

## Slide 1
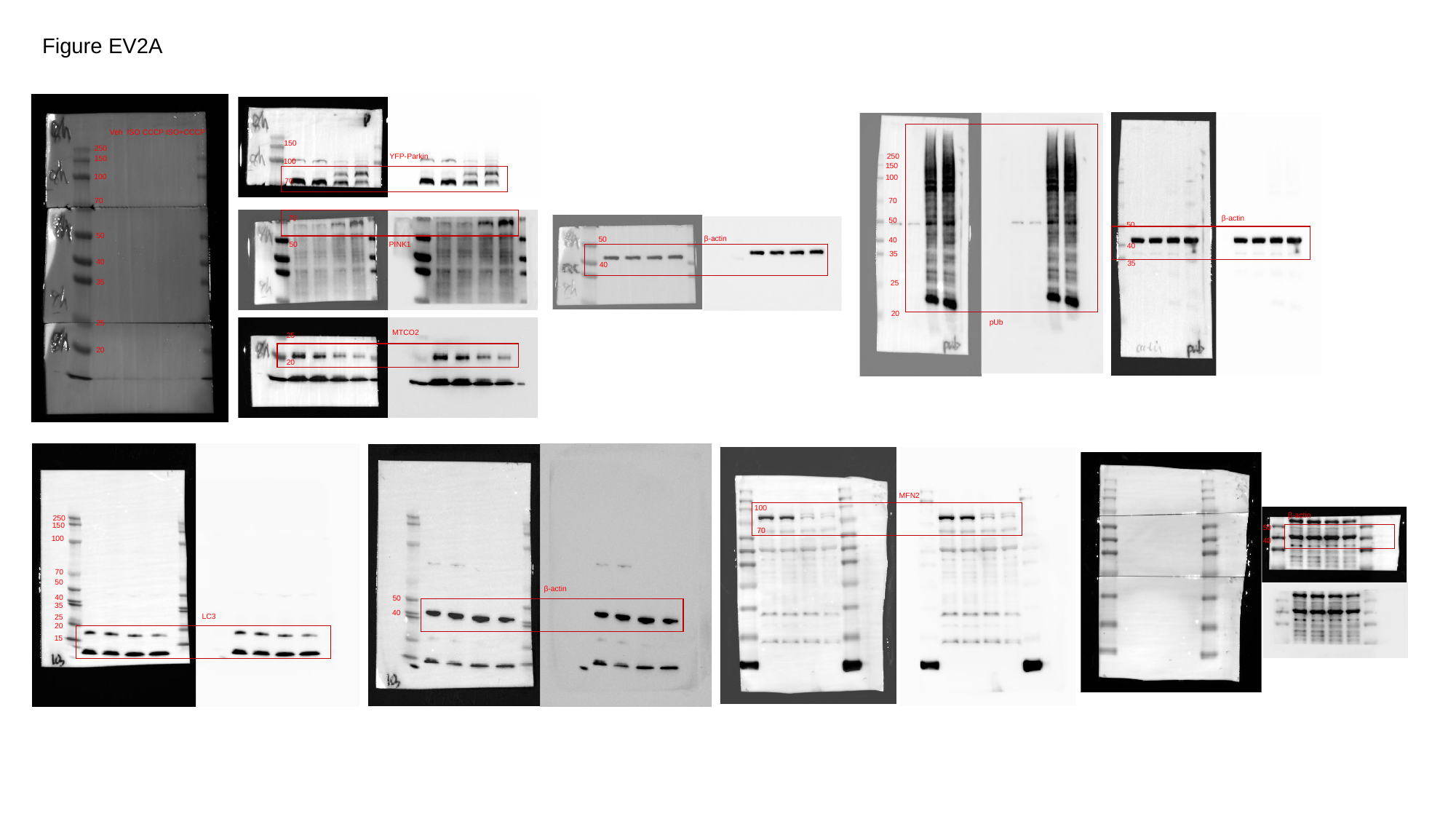

Figure EV2A
Veh ISO CCCP ISO+CCCP
250
YFP-Parkin
150
100
70
50
β-actin
PINK1
40
35
25
MTCO2
20
150
100
70
70
50
50
40
25
20
β-actin
50
40
35
250
150
100
70
50
40
35
25
20
pUb
250
150
100
70
50
40
35
LC3
25
20
15
β-actin
50
40
MFN2
100
β-actin
50
70
40

## Slide 2
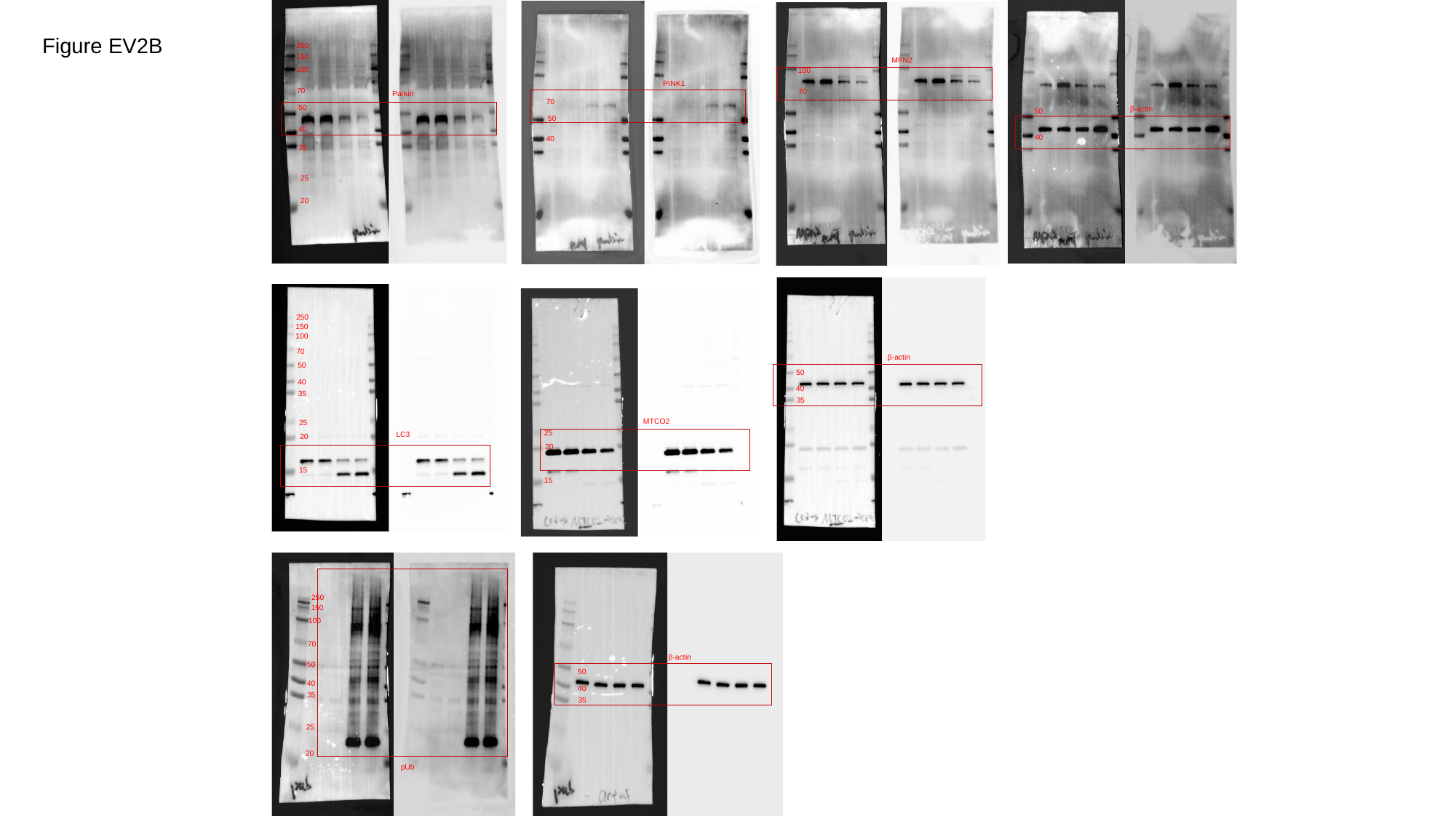

β-actin
50
40
PINK1
70
50
40
250
150
100
70
Parkin
50
40
35
25
20
Figure EV2B
MFN2
100
70
β-actin
50
40
35
250
150
100
70
50
40
35
25
LC3
20
15
MTCO2
25
20
15
250
150
100
70
β-actin
50
50
40
40
35
35
25
20
pUb

## Slide 3
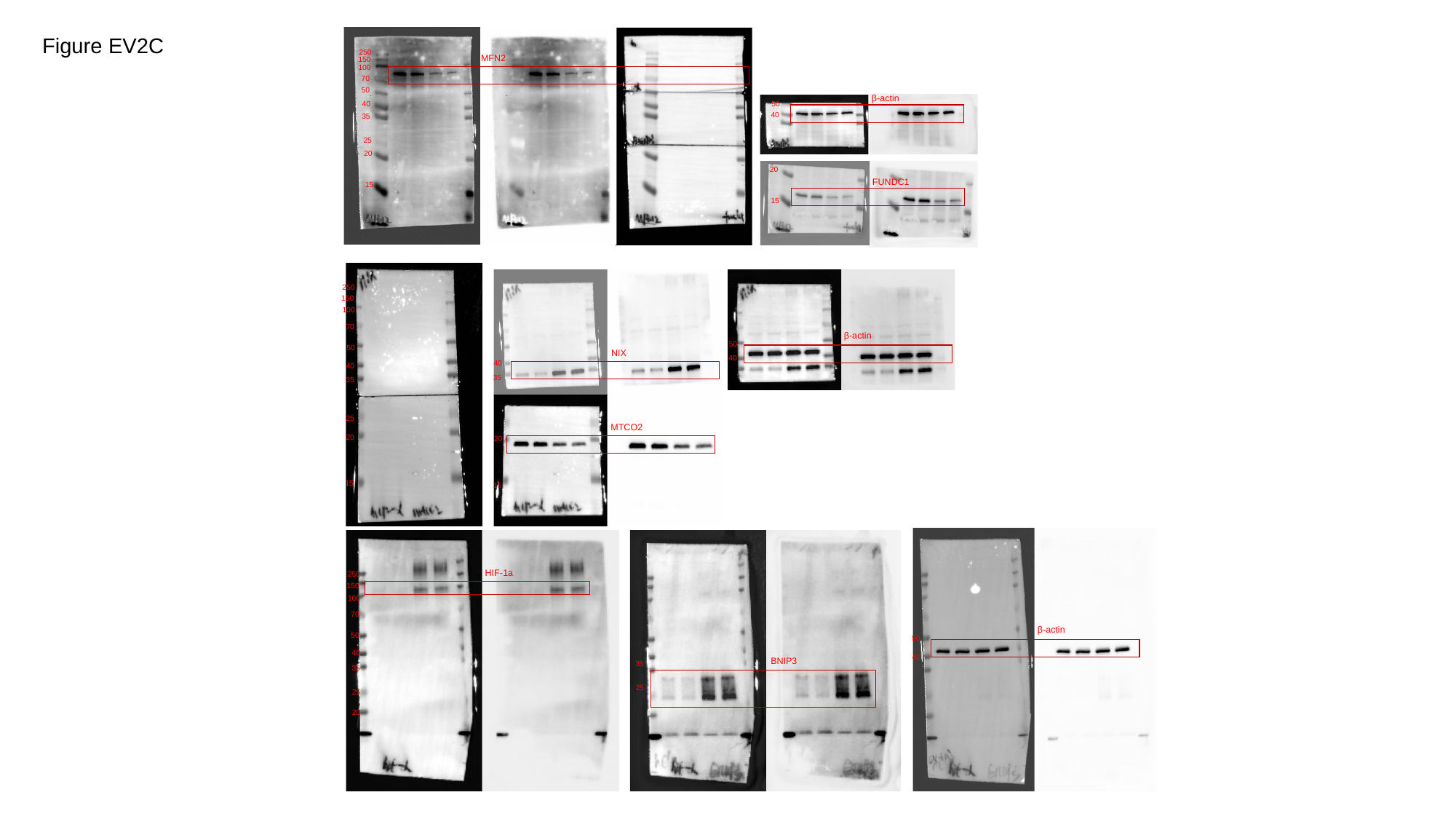

Figure EV2C
250
MFN2
150
100
70
50
40
35
25
20
15
β-actin
50
40
20
FUNDC1
15
250
150
100
70
50
NIX
40
40
35
35
25
MTCO2
20
20
15
15
β-actin
50
40
β-actin
50
40
HIF-1a
250
150
100
70
50
40
BNIP3
35
35
25
25
20

## Slide 4
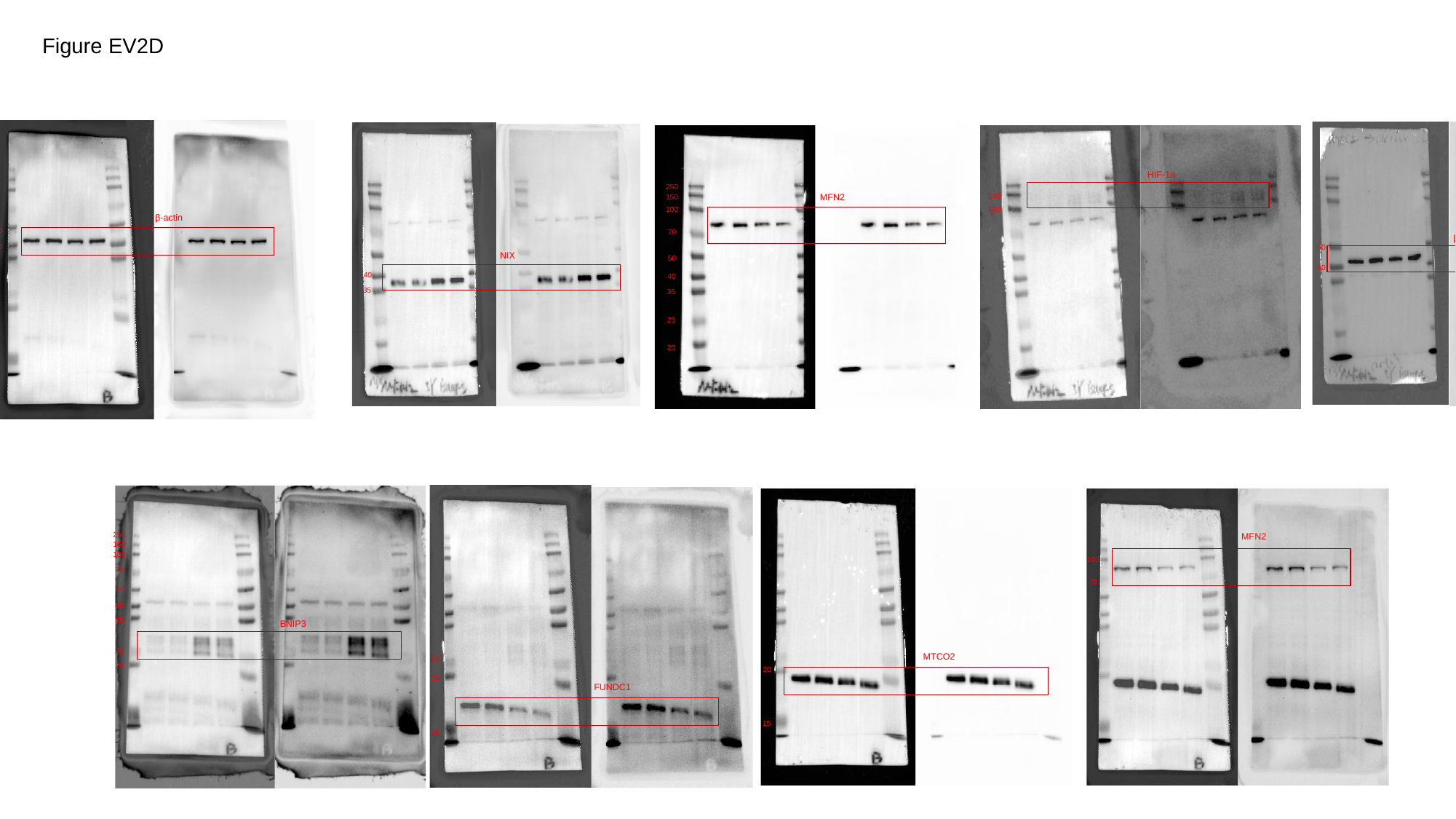

Figure EV2D
β-actin
50
40
β-actin
50
40
NIX
40
35
HIF-1a
250
150
MFN2
150
100
100
70
50
40
35
25
20
BNIP3
250
150
100
70
50
40
35
25
20
25
20
FUNDC1
15
MFN2
100
70
MTCO2
20
15

## Slide 5
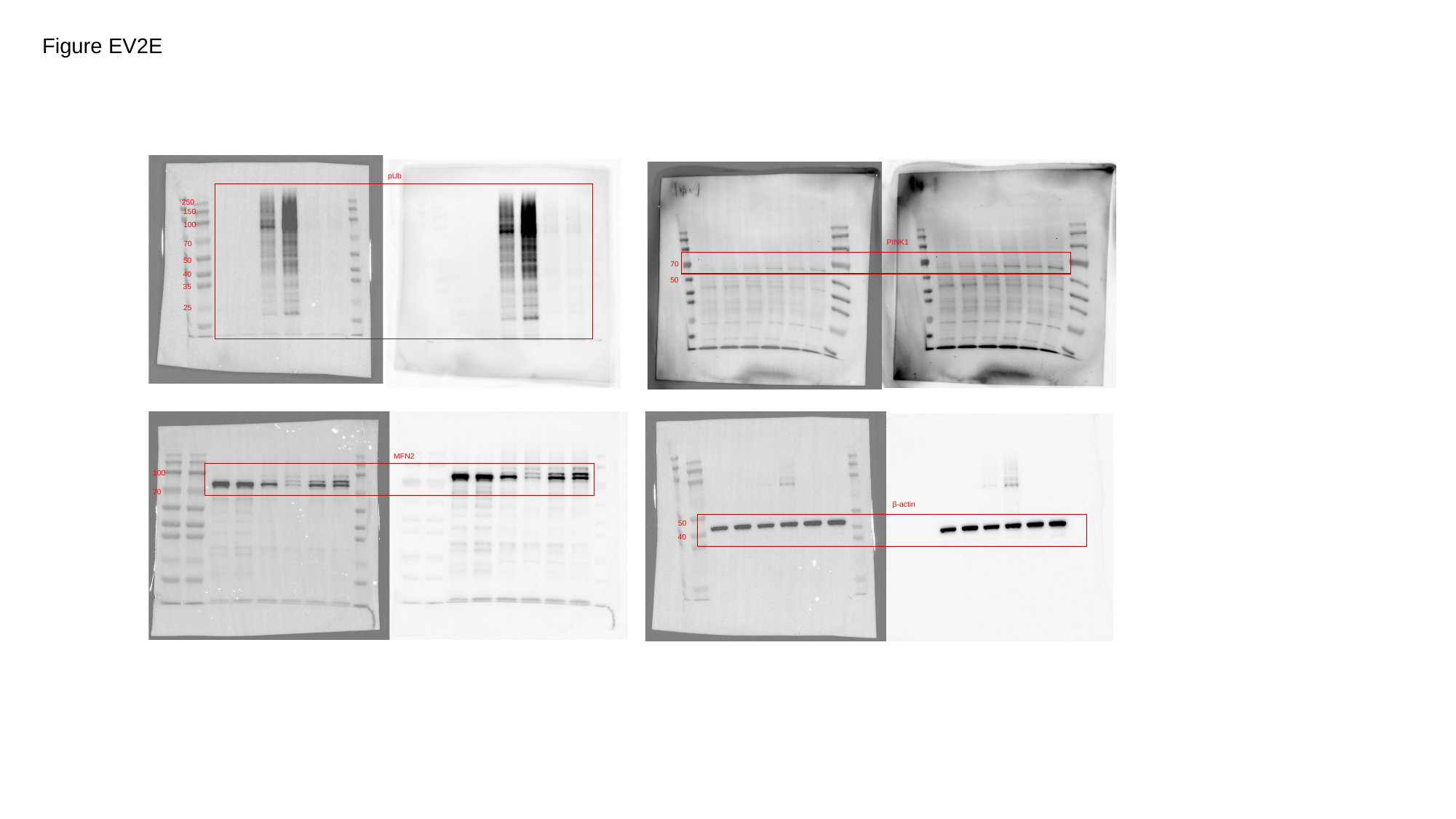

Figure EV2E
pUb
250
150
100
PINK1
70
50
70
40
50
35
25
MFN2
100
70
β-actin
50
40

## Slide 6
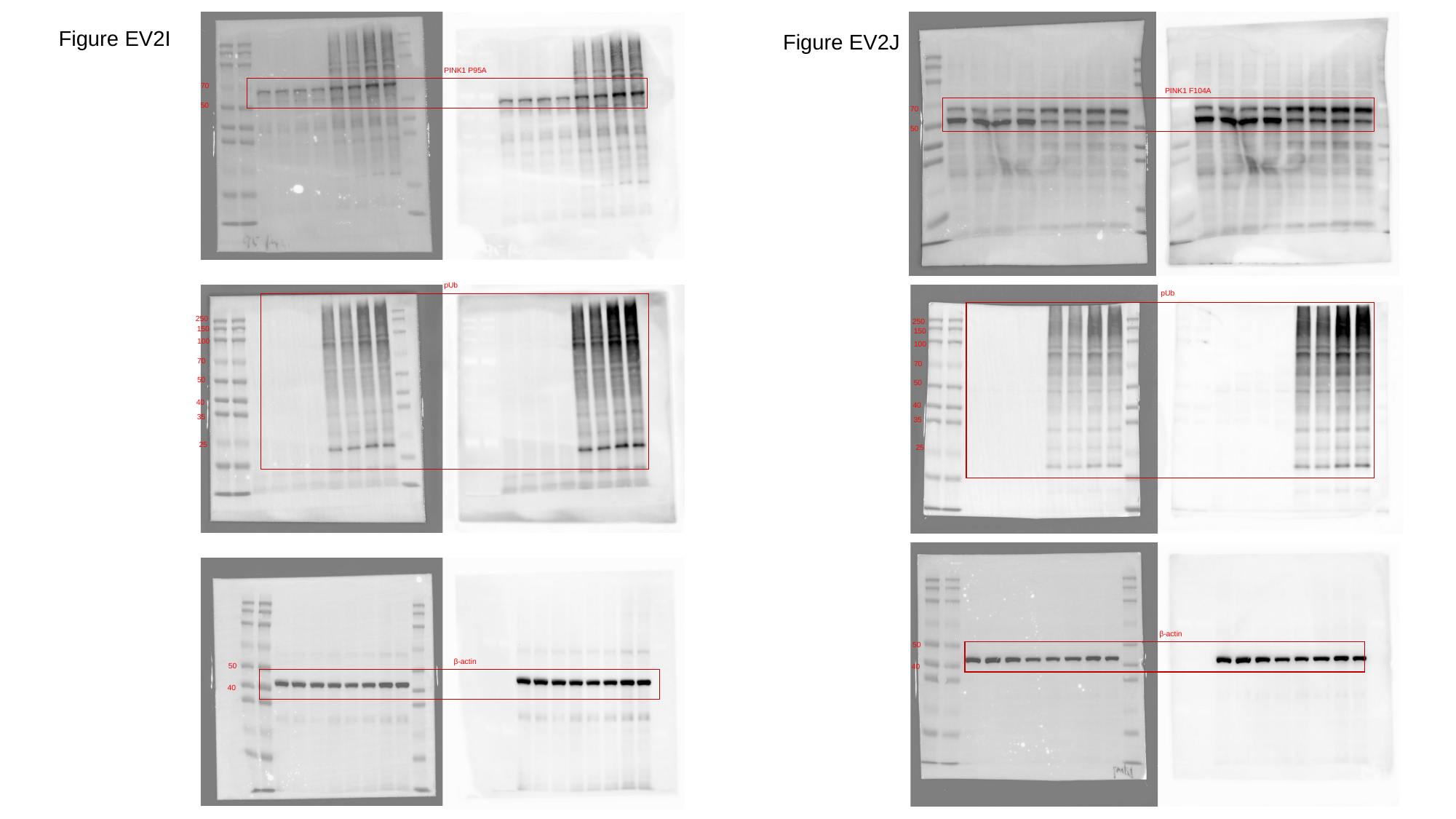

PINK1 P95A
70
50
pUb
250
150
100
70
50
40
35
25
β-actin
50
40
PINK1 F104A
70
50
pUb
250
150
100
70
50
40
35
25
β-actin
50
40
Figure EV2I
Figure EV2J
