# Supplementary material for: Isoginkgetin antagonizes ALS pathologies in its animal and patient iPSC models via PINK1-Parkin-dependent mitophagy
Source: EMBO Mol Med. 2025 Oct 15;17(11):3139–73. doi: 10.1038/s44321-025-00323-2 (PMC12603167; doi:10.1038/s44321-025-00323-2)
Supplement: Supplementary file 14 — Expanded View Figures [file 44321_2025_323_MOESM14_ESM.pdf]

Expanded View Figures

Figure EV1. High-content drug screening identifies ISO as a potent PINK1-Parkin mitophagy agonist.

(**A**) Flowchart for high-content drug screening. Scale bar, 25 μm. (**B**) Representative images of Top 20 mitophagy inducers. Scale bar, 50 μm. (**C**) YFP-Parkin-mt-mKeima HeLa cells were treated with ISO (10 μM) for 24 h. Flow cytometry was used to detect mt-mKeima indicated mitophagy signals. (**D**) The statistical results showed that ISO (10 μM) activated mitophagy ($n = 5$; five biological repeats). (**E**) Representative images of Parkin translocation induced by ISO (10 μM). Scale bar, 15 μm. (**F**) Quantification of (**E**) showed that ISO (10 μM) induced substantial Parkin translocation ($n = 5$; five biological repeats). (**G**) Three siRNAs targeting *PINK1* were transfected into YFP-Parkin-mt-mKeima HeLa cells with Lipo3000, and after 72 h transfection, CCCP was added into cells to induce PINK1-dependent mitophagy. Western blotting showed that PINK1 was successfully knocked down, and the expression of pSer65-Ub was also significantly inhibited. (**H**) siRNAs targeting *BNIP3*, *NIX*, *FUNDC1* and *BNIP3/NIX* double knock-down (KD) were transfected into YFP-Parkin-mt-mKeima HeLa cells with Lipo3000, and after 72 h transfection, Western blotting showed that each receptor protein was successfully knocked down. (**I**) Only *PINK1* KD in YFP-Parkin-mt-mKeima HeLa cells can block 10 μM ISO (8 h) induced mitophagy signals ($n = 5$; five biological repeats). Data are presented as the mean ± SD. Exact *P* values are reported in Appendix Table S1. One-way ANOVA followed by Dunnett's multiple comparisons test. Source data are available online for this figure.

© The Author(s)

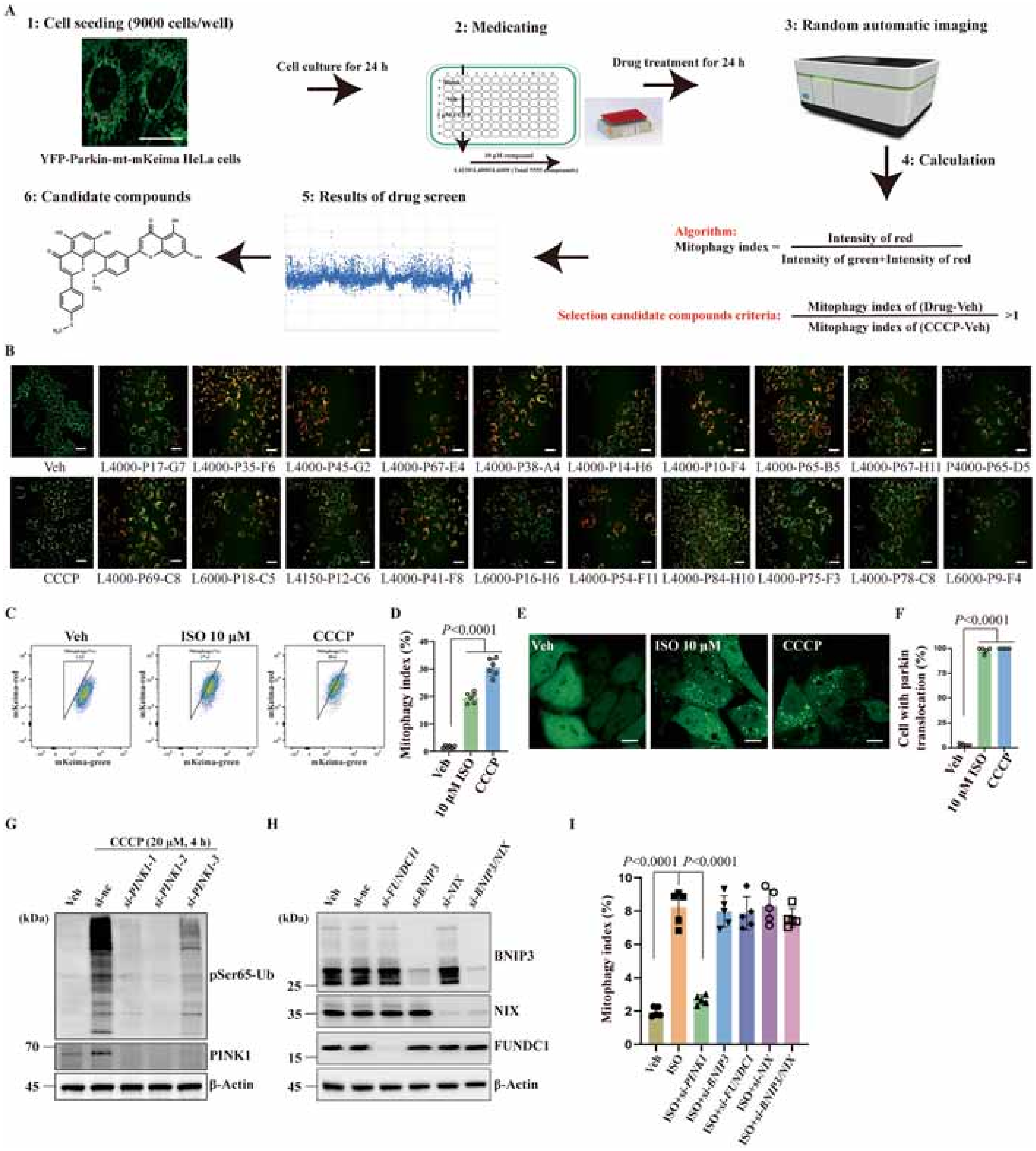
A
1: Cell seeding (9000 cells/well)
YFP-Parkin-mt-mKeima HeLa cells
Cell culture for 24 h
2: Medicating
Drug treatment for 24 h
3: Random automatic imaging
4: Calculation
Algorithm:
Mitophagy index = Intensity of red / Intensity of green+Intensity of red
Selection candidate compounds criteria: Mitophagy index of (Drug-Veh) / Mitophagy index of (CCCP-Veh) >1
5: Results of drug screen
6: Candidate compounds
B
Veh
L4000-P17-G7
L4000-P35-F6
L4000-P45-G2
L4000-P67-E4
L4000-P38-A4
L4000-P14-H6
L4000-P10-F4
L4000-P65-B5
L4000-P67-H11
P4000-P65-D5
CCCP
L4000-P69-C8
L6000-P18-C5
L4150-P12-C6
L4000-P41-F8
L6000-P16-H6
L4000-P54-F11
L4000-P84-H10
L4000-P75-F3
L4000-P78-C8
L6000-P9-F4
C
Veh
ISO 10 μM
CCCP
mKeima-red
mKeima-green
D
P<0.0001
Mitophagy index (%)
Veh
10 μM ISO
CCCP
E
Veh
ISO 10 μM
CCCP
F
P<0.0001
Cell with parkin translocation (%)
Veh
10 μM ISO
CCCP
G
CCCP (20 μM, 4 h)
Veh
si-nc
si-PINK1-1
si-PINK1-2
si-PINK1-3
(kDa)
pSer65-Ub
70
PINK1
45
β-Actin
H
Veh
si-nc
si-FUNDC1
si-BNIP3
si-NIX
si-BNIP3/NIX
(kDa)
BNIP3
25
35
NIX
15
FUNDC1
45
β-Actin
I
P<0.0001
P<0.0001
Mitophagy index (%)
Veh
ISO
ISO+si-PINK1
ISO+si-BNIP3
ISO+si-FUNDC1
ISO+si-NIX
ISO+si-BNIP3/NIX

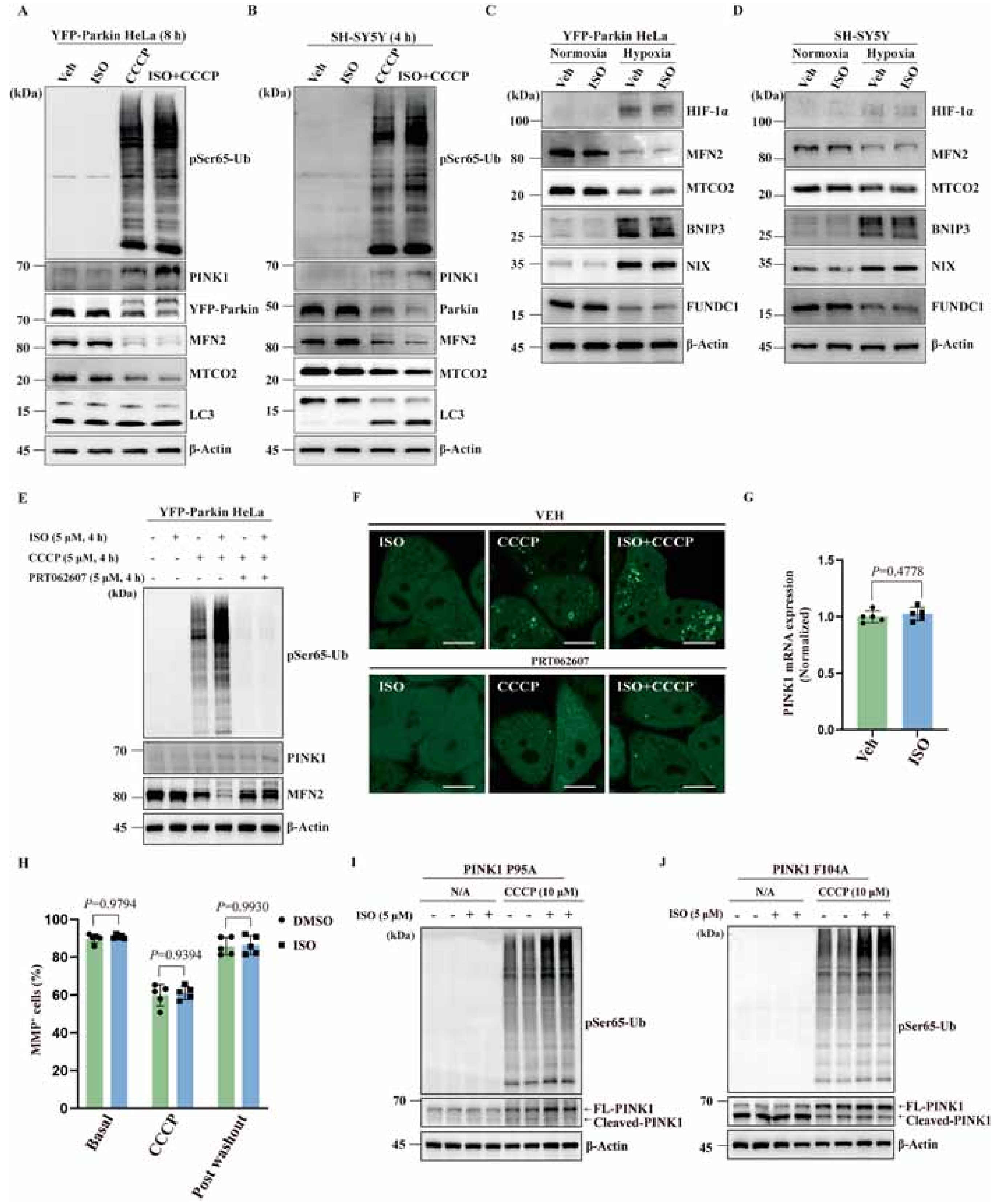
A
YFP-Parkin HeLa (8 h)
Veh
ISO
CCCP
ISO+CCCP
pSer65-Ub
PINK1
YFP-Parkin
MFN2
MTCO2
LC3
β-Actin
B
SH-SY5Y (4 h)
Parkin
C
YFP-Parkin HeLa
Normoxia
Hypoxia
HIF-1α
BNIP3
NIX
FUNDC1
D
SH-SY5Y
E
ISO (5 μM, 4 h)
CCCP (5 μM, 4 h)
PRT062607 (5 μM, 4 h)
F
VEH
PRT062607
G
P=0.4778
PINK1 mRNA expression (Normalized)
H
P=0.9794
P=0.9394
P=0.9930
DMSO
MMP+ cells (%)
Basal
Post washout
I
PINK1 P95A
N/A
CCCP (10 μM)
ISO (5 μM)
FL-PINK1
Cleaved-PINK1
J
PINK1 F104A

 © The Author(s)

Figure EV2. ISO promotes mitophagy via mediating PINK1 function.

(**A**) 5 μM ISO treatment for 8 h can promote CCCP-induced PINK1-Parkin-dependent mitophagy protein expression, mitochondrial membrane protein degradation and LC3 lipidation in YFP-Parkin HeLa cells. (**B**) 5 μM ISO treatment for 4 h can promote CCCP-induced PINK1-Parkin-dependent mitophagy protein expression, mitochondrial membrane protein degradation and LC3 lipidation in SH-SY5Y cells. (**C**) 5 μM ISO treatment for 24 h cannot promote hypoxia-induced receptor-mediated mitophagy protein expression in YFP-Parkin HeLa cells. (**D**) 5 μM ISO treatment for 24 h cannot promote hypoxia-induced receptor-mediated mitophagy protein expression in SH-SY5Y cells. (**E**) Immunoblotting showed that PRT, a PINK1 inhibitor, prevented the enhancing effect of ISO on CCCP-induced pSer65-Ub expression and MFN2 degradation, and Parkin translocation (**F**). (**G**) Relative mRNA expression levels of PINK1 in YFP-Parkin-PINK1-Myc HeLa cells between the Veh and ISO (5 μM, 4 h) group ($n = 5$; five biological repeats). (**H**) In a CCCP washout experiment, MMP was quantified by flow cytometry with TMRE probe at the indicated timepoints. Quantification of the TMRE intensity showed that ISO stabilized PINK1 without causing MMP collapse ($n = 5$; five biological repeats). (**I**, **J**) Immunoblotting showed that ISO was still able to increase pSer65-Ub expression in YFP-Parkin HeLa cells transfected with PINK1 P95A and F104A mutation plasmids. Data are presented as the mean ± SD. Exact *P* values are reported in Appendix Table S1. Unpaired *t* test in (**G**). Two-way ANOVA followed by Tukey's multiple comparisons test in (**H**). Source data are available online for this figure.

© The Author(s)

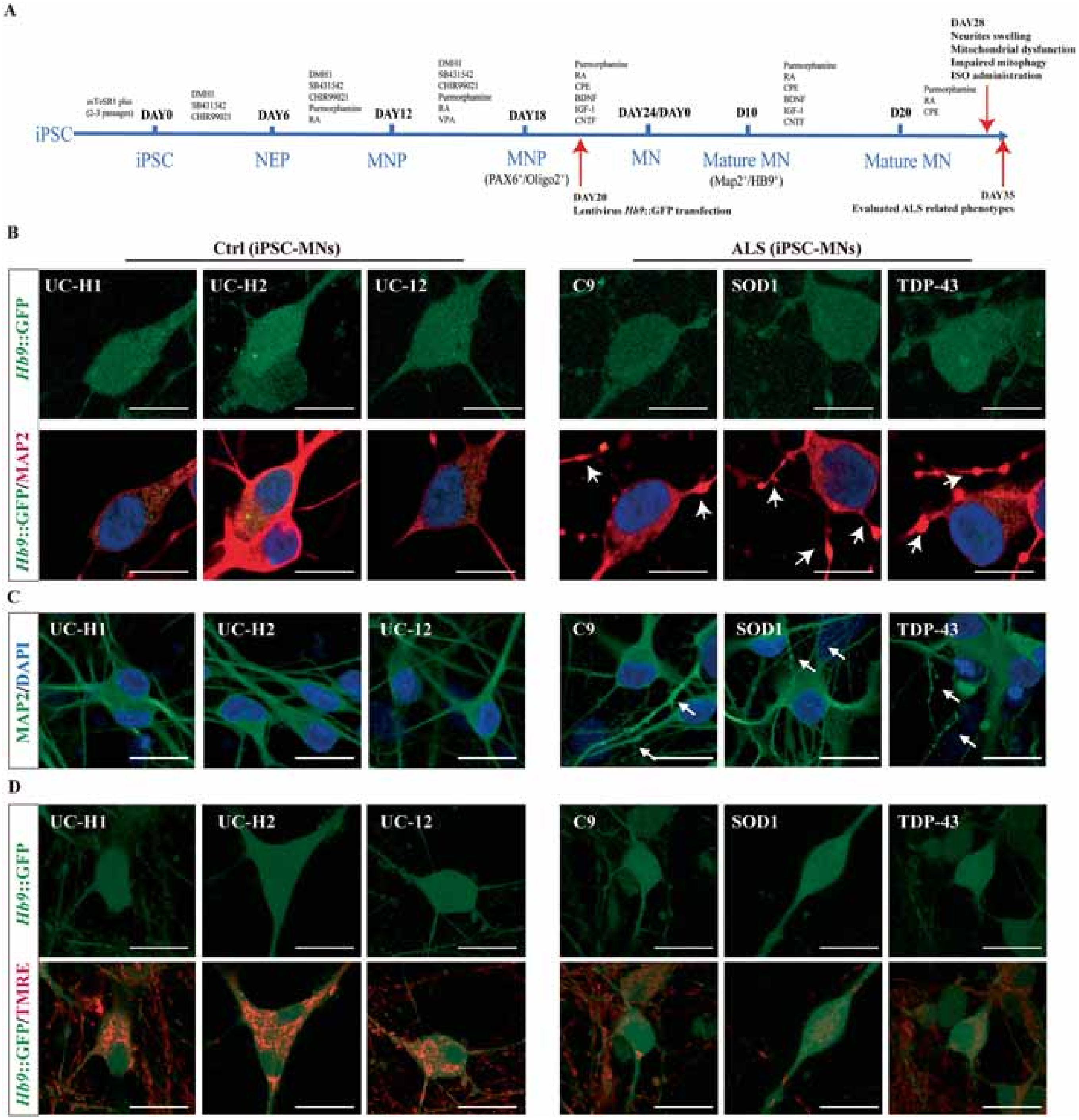

Figure EV3. ALS-related phenotypes in three ALS patient iPSC lines derived MNs.

(**A**) Schematic of the MN differentiation protocol. (**B**) $OLIG2^{+}$ motor neuron progenitors (MNPs) were transfected with lentivirus *Hb9*::GFP and further induced to differentiate into MNs. Immunostaining showed $MAP2^{+}/GFP^{+}$ MNs were successfully differentiated, and neurites swelling with bead-like structures appeared in ALS MNs (white arrow) on day 28 at stage 5. Scale bars, 15 μm. (**C**) Representative images of neurites with bead-like structures (white arrow) in $MAP2^{+}$ MNs on day 28 at stage 5. Scale bars, 20 μm. (**D**) Representative images of TMRE staining of GFP^{+} MNs showed that MMP was significantly reduced in ALS *Hb9*::GFP^{+} MNs on day 28 at stage 5. Scale bars, 20 μm.

 © The Author(s)

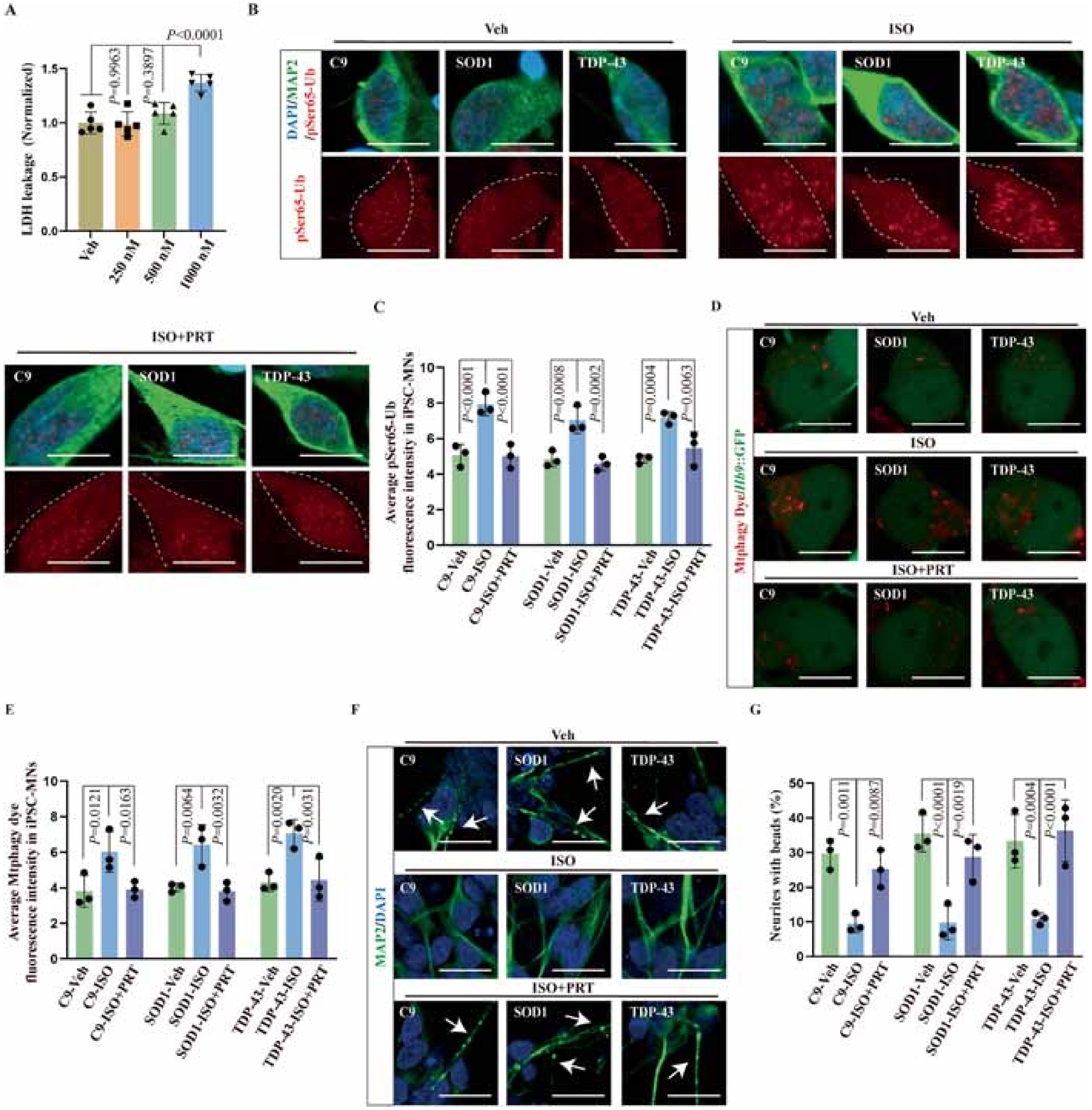

Figure EV4. PRT abolishes the beneficial effects of ISO in three ALS patient iPSC-derived MNs.

(**A**) LDH leakage assay determined upon a working concentration of ISO (without neurotoxicity) of 0.5 μM (n = 5; three biological repeats). (**B**, **C**) ISO increased pSer65-Ub expression in three ALS patient iPSC lines derived MAP2+ MNs and PRT, a PINK1 inhibitor blocked the effect of ISO on increasing pSer65-Ub expression. MAP2+ motor neurons are marked with white dotted borders. Scale bar, 15 μm. Each point represents the average value of 10 MNs for each iPSC-derived MNs; three biological replicates for each group. (**D**) Representative living cell images of the mtphagy dye in three ALS patient iPSC-derived MNs (*Hb9*::GFP+). Scale bars, 15 μm. (**E**) Quantification of (D) the fluorescence intensity demonstrating that ISO enhanced mitophagy in three ALS patient iPSC-derived *Hb9*::GFP+ MNs, and PRT blocked this effect. Each point represents the average value of 10 MNs for each iPSC-derived MNs; three biological replicates for each group. (**F**) Representative images of neurites with swelling bead-like structures (white arrow) in three ALS patient iPSC lines derived MAP2+ MNs. Scale bars, 50 μm. (**G**) Quantification of (**F**) showing that ISO decreased neurites with swelling bead-like structures in three ALS patient iPSC line-derived MAP2+ MNs after 7 days of treatment and PRT abolished this effect. Each point represents the average value of 10 images for each iPSC-derived MNs; three biological replicates for each group. Data are presented as the mean ± SD. Exact *P* values are reported in Appendix Table S1. One-way ANOVA followed by Dunnett's multiple comparisons test in (**A**). Two-way ANOVA followed by Tukey's multiple comparisons test in (**C**, **E**, **G**).

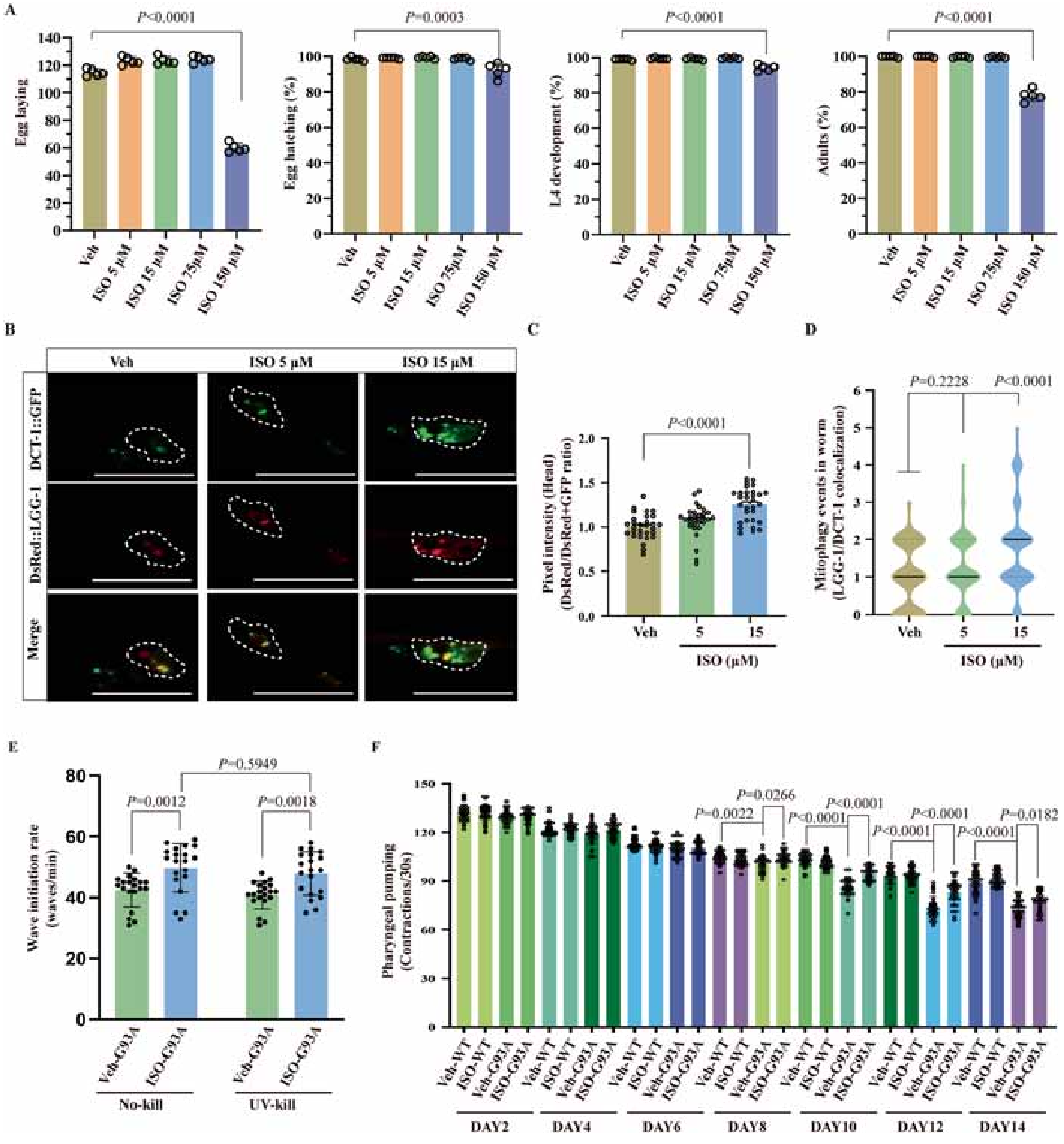

Figure EV5. ISO induces neuronal mitophagy and ameliorates ALS-related phenotypes in *C. elegans*.

(**A**) Effects of ISO (5 to 150 μM) on the egg laying rate, egg hatching rate (L1), L4 development rate, and the adulthood rate (Adult day 1) in the WT N2 nematodes. Toxicity tests revealed a safe concentration range of ISO between 0 and 75 μM (n = 5; 10 worms for each biological repeats). (**B**) Representative images showing the LGG-1 and DCT-1 co-localization in DMSO or ISO treatment group on adult day 1. *unc119*[+] neurons are marked with white dotted borders. Scale bar, 5 μm. (**C**, **D**) Statistical results of mt-Rosella (**C**) and LGG-1/DCT-1 (**D**) transgenic worms showed that 15 μM ISO activated neuronal mitophagy (n = 30 neurons from 30 worms for each group). (**E**) UV-killed OP50 experiment showing that the protective effect of ISO on the motility of SOD1 G93A worms was independent of microbial metabolism (n = 20 worms for each group). (**F**) Effect of ISO on pharyngeal pumping speed from adult day 2 to day 14 in WT (N2) and SOD1 G93A worms. The results showed that ISO did not restrict the nematode diet (n = 30 worms for each group). Data are presented as the mean ± SD. Exact P values are reported in Appendix Table S1. One-way ANOVA followed by Dunnett's multiple comparisons test in (**A**, **C**, **D**, **F**). Two-way ANOVA followed by Tukey's multiple comparisons test in (**E**).

 © The Author(s)

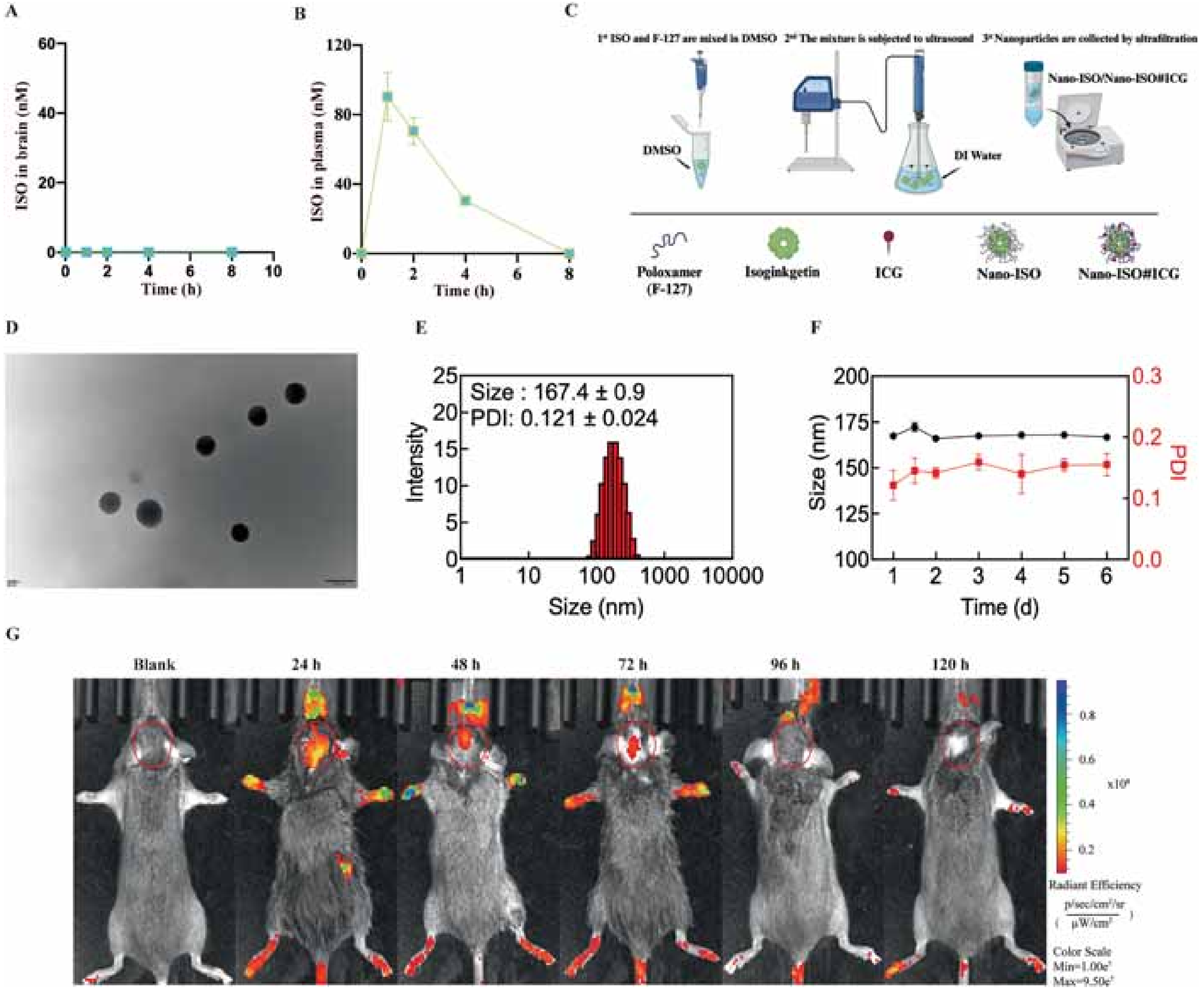

Figure EV6. Synthesis and characterization of self-assembled Nano-ISO.

(**A**, **B**) ISO concentrations in brain tissue (**A**) and plasma (**B**) detected by the UPLC-MS/MS assay after a single intraperitoneal injection of ISO ($n = 3$; three biological repeats). (**C**) Schematic illustration of the synthesis process of Nano-ISO and Nano-ISO#ICG. (**D**) TEM images of Nano-ISO in aqueous solutions at pH 7.4 for 24 h. (**E**) The size value and PDI of Nano-ISO were detected by particle size analyzer. (**F**) Within 6 days, the size of the Nano-ISO particles decreased gradually, and the PDI value increased gradually ($n = 3$; three biological repeats). (**G**) Representative IVIS images of the distribution of Nano-ISO#ICG over time in the mice after a single intranasal administration.

© The Author(s)

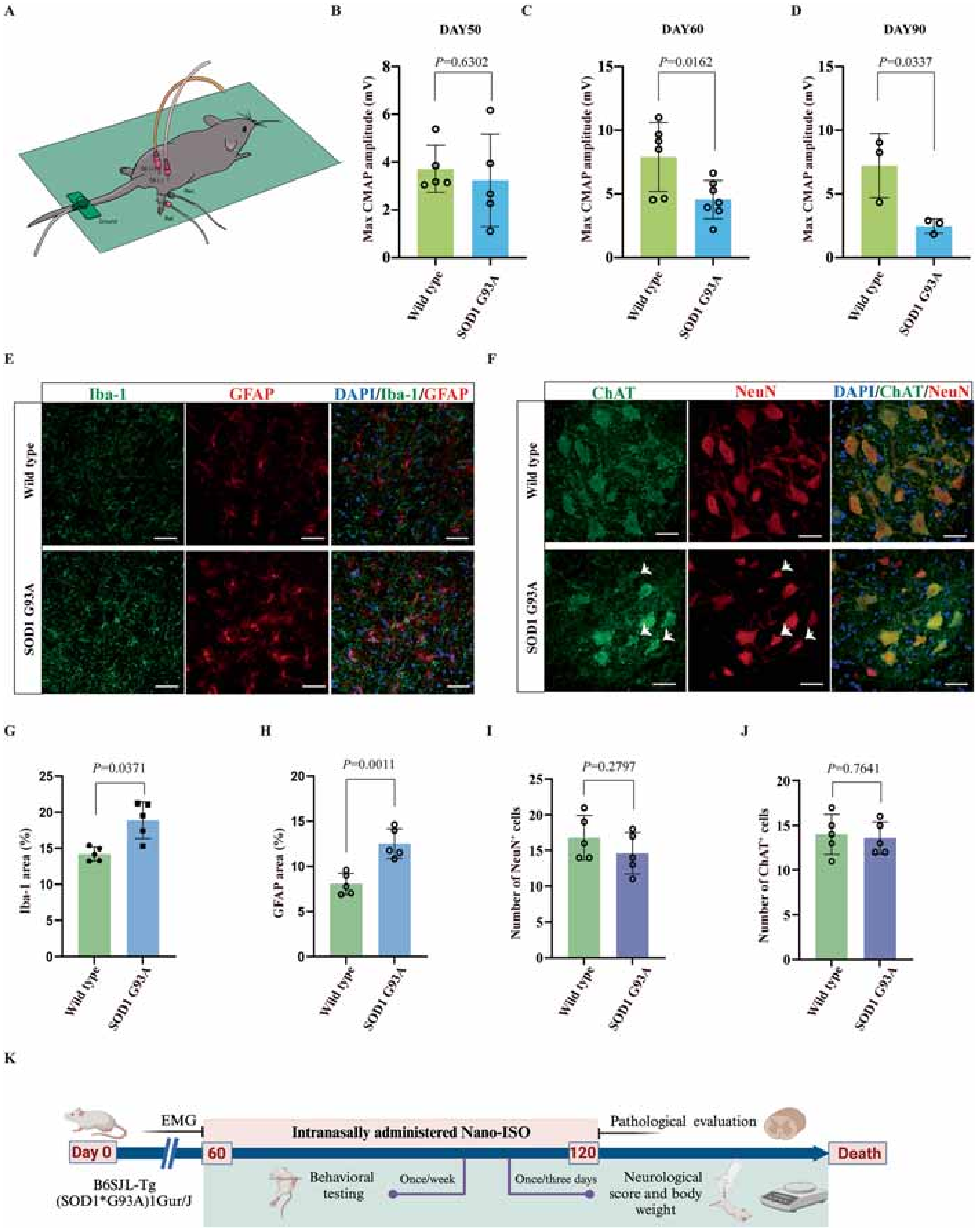
A
B
DAY50
P=0.6302
Max CMAP amplitude (mV)
Wild type
SOD1 G93A
C
DAY60
P=0.0162
D
DAY90
P=0.0337
E
Iba-1
GFAP
DAPI/Iba-1/GFAP
Wild type
SOD1 G93A
F
ChAT
NeuN
DAPI/ChAT/NeuN
G
P=0.0371
Iba-1 area (%)
H
P=0.0011
GFAP area (%)
I
P=0.2797
Number of NeuN+ cells
J
P=0.7641
Number of ChAT+ cells
K
EMG
Intranasally administered Nano-ISO
Pathological evaluation
Day 0
60
120
Death
B6SJL-Tg
(SOD1*G93A)1Gur/J
Behavioral testing
Once/week
Once/three days
Neurological score and body weight

Figure EV7. SOD1 G93A mice exhibit neurogenic damage and neuroinflammation at Day 60.

(**A**) Schematic diagram of electrophysiological test in mice. (**B–D**) Statistical results of the CMAP amplitude of gastrocnemius muscle by stimulating sciatic nerve in SOD1 G93A mouse and WT group on Day 50 (**B**), Day 60 (**C**), and Day 90 (**D**). The maximum CMAP amplitude was noted to decrease significantly in SOD1 G93A mice on day 60, $n = 5$ mice/group on day 50; $n = 6$ mice for WT and $n = 7$ mice for G93A on day 60; $n = 3$ mice/group on day 90. (**E**) Representative images of microglia and astrocyte activation revealed by Iba-I staining and GFAP staining. Scale bar, 25 µm. (**F**) Representative images of ChAT and NeuN staining. White arrows indicate motor neurons with shrunken cell bodies. Scale bar, 25 µm. (**G**, **H**) Statistical results of Iba-I staining and GFAP staining in (**E**) demonstrating that both the microglia and astrocyte activation were detected in the ventral horn of lumbar spinal cord of SOD1 G93A mice on day 60. Each point represents the average value of 10 images for each mouse, $n = 5$ mice/group. (**I**, **J**) Statistical results of the number of MNs by NeuN staining and ChAT staining. No loss of MNs was detected in the ventral horn of lumbar spinal cord of SOD1 G93A mice on day 60. Each point represents the average value of 10 images for each mouse, $n = 5$ mice/group. (**K**) Schematic diagram of the exploration of Nano-ISO therapeutic effects in SOD1 G93A mice. Data are presented as the mean ± SD. Exact *P* values are reported in Appendix Table S1. Unpaired *t* test.

© The Author(s)
